# tRNA m^1^A modification ensures HSPC production via modulating Nrf1 translation in zebrafish

**DOI:** 10.1038/s44319-026-00805-5

**Published:** 2026-05-27

**Authors:** Zhenkun Dong, Panfeng Li, Mengyao Liu, Sifeng Wang, Weiyi Lai, Yining Liu, Hailin Wang, Ang Li, Lu Wang

**Affiliations:** 1 State Key Laboratory of Experimental Hematology, National Clinical Research Center for Blood Diseases, Haihe Laboratory of Cell Ecosystem, Institute of Hematology & Blood Diseases Hospital, Chinese Academy of Medical Sciences & Peking Union Medical College, Tianjin, China; 2 Shandong Provincial Key Laboratory of Animal Cell and Developmental Biology, School of Life Sciences, Shandong University, Qingdao, China; 3 Hunan Provincial Key Laboratory of Regional Hereditary Birth Defects Prevention and Control, Changsha Hospital for Maternal and Child Health Care Affiliated to Hunan Normal University, Changsha, China; 4 State Key Laboratory of Environmental Chemistry and Ecotoxicology, Research Center for Eco-Environmental Sciences, Chinese Academy of Sciences, Beijing, China; 5 State Key Laboratory of Membrane Biology, Institute of Zoology, Institute for Stem Cell and Regeneration, Chinese Academy of Sciences, University of Chinese Academy of Sciences, Beijing, China; 6 Tianjin Institutes of Health Science, Tianjin, China

**Keywords:** RNA Biology, Stem Cells & Regenerative Medicine, Translation & Protein Quality

## Abstract

During embryogenesis, nascent hematopoietic stem and progenitor cells (HSPCs) arise from hemogenic endothelium via endothelial-to-hematopoietic transition (EHT). While this process is orchestrated by multiple intrinsic factors, the role of tRNA-mediated translational control during EHT remains poorly understood. Here, we identify tRNA m^1^A58 as a predominant tRNA modification in specifying HSPC fate in zebrafish embryos. Depletion of *trmt61a* compromises HSPC production in the aorta-gonad-mesonephros (AGM) region, accompanied by attenuated tRNA m^1^A58 levels and evident *p53*-dependent apoptosis. Mechanistically, Trmt61a-mediated tRNA m^1^A58 modification enhances translation efficiency of nuclear respiratory factor 1 (Nrf1), a key regulator of mitochondrial biogenesis. Consequently, *trmt61a* deficiency leads to mitochondrial dysfunction and compromises cell survival, ultimately impairing HSPC production. Our findings establish that tRNA m^1^A58 modification is essential for HSPC generation by supporting translation efficiency, providing new insights into improved strategies for in vitro HSPC induction.

## Synopsis

Trmt61a-mediated tRNA m^1^A58 modification participates in HSPC generation by promoting Nrf1 protein translation, thereby governing mitochondrial biogenesis and facilitating HSPC generation during embryonic hematopoiesis.

- Trmt61a-mediated tRNA m1A58 modification is required for embryonic HSPC generation in zebrafish.
- Loss of *trmt61a* impairs translation efficiency, selectively affecting codon-biased transcripts including Nrf1.
- Reduced Nrf1 translation disrupts mitochondrial biogenesis and triggers p53-dependent apoptosis, leading to HSPC defects.

## Abstract (web summary)

Trmt61a-mediated tRNA m^1^A58 modification participates in HSPC generation by promoting Nrf1 protein translation, thereby governing mitochondrial biogenesis and facilitating HSPC generation during embryonic hematopoiesis.

## Graphical abstract

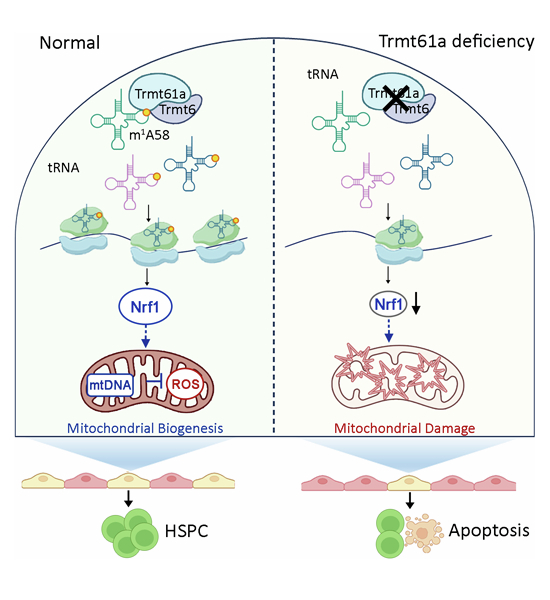

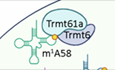

## Introduction

Hematopoietic stem and progenitor cells (HSPCs) maintain lifelong blood cell production through a precisely balanced triad of self-renewal, differentiation, and quiescence. During vertebrate embryogenesis, the earliest HSPCs are derived from hemogenic endothelial cells (HECs), located in the ventral wall of dorsal aorta (VDA) in the aorta-gonad-mesonephros (AGM) region, through endothelial-to-hematopoietic transition (EHT) (Boisset et al, 2010; Clements and Traver, 2013; Kissa and Herbomel, 2010). The process demonstrates significantly elevated ribosome biogenesis activity (Liu et al, 2024), revealing a heightened demand for protein synthesis during EHT. However, how proteome quality and specificity are remodeled to instruct this precise fate transition remains unknown.

tRNA modifications constitute a key layer of translational control (Schultz and Kothe, 2024). tRNA emerges as the most extensively modified RNA species (Cappannini et al, 2024), and these modifications regulate tRNA stability, translation fidelity and efficiency, tRNA processing and maturation, as well as the biogenesis of tRNA fragments (Lin et al, 2018; Muthukumar et al, 2024; Ontiveros et al, 2020; Tuorto et al, 2015; Zhu et al, 2024). One of the most prevalent modifications is N1-methyladenosine (m^1^A). Among them, m^1^A58 is a conserved modification located at position 58 in the T-loop of tRNAs and plays an important role in maintaining tRNA structural stability and proper function (Degut et al, 2016; Saikia et al, 2010; Xiong et al, 2018). In cytoplasmic tRNAs, m^1^A58 modification is catalyzed by TRMT61A/TRMT6 complex (MTC) (Ozanick et al, 2005), a heterotetrameric complex comprising two structurally homologous yet evolutionarily divergent subunits (Oerum et al, 2017). TRMT6 primarily mediates substrate recognition, enabling TRMT61A to catalyze methylation of the target adenosine (Finer-Moore et al, 2015). tRNA m^1^A58 modification has been implicated in diverse biological processes, including T cell expansion and anti-tumor activity, metabolism, and HSC homeostasis, primarily through translational modulation of key regulatory proteins (Liu et al, 2022; Miao et al, 2025; Wang et al, 2021; Zuo et al, 2024). Despite these insights, the understanding of tRNA m^1^A58 in embryonic hematopoiesis remains incomplete.

In this study, we demonstrate that Trmt61a-mediated tRNA m^1^A58 modification is indispensable for embryonic HSPC generation in zebrafish. Loss of tRNA m^1^A58 modification reduces Nrf1 translation efficiency, leading to impaired mitochondrial biogenesis and apoptosis in AGM EC, ultimately compromising HSPC production. Our finding sheds light on a pivotal mechanism through which tRNA m^1^A58 modification orchestrates translational regulation during HSPC ontogeny.

## Results

### Trmt61a is essential for HSPC generation in zebrafish

**Figure 1:**
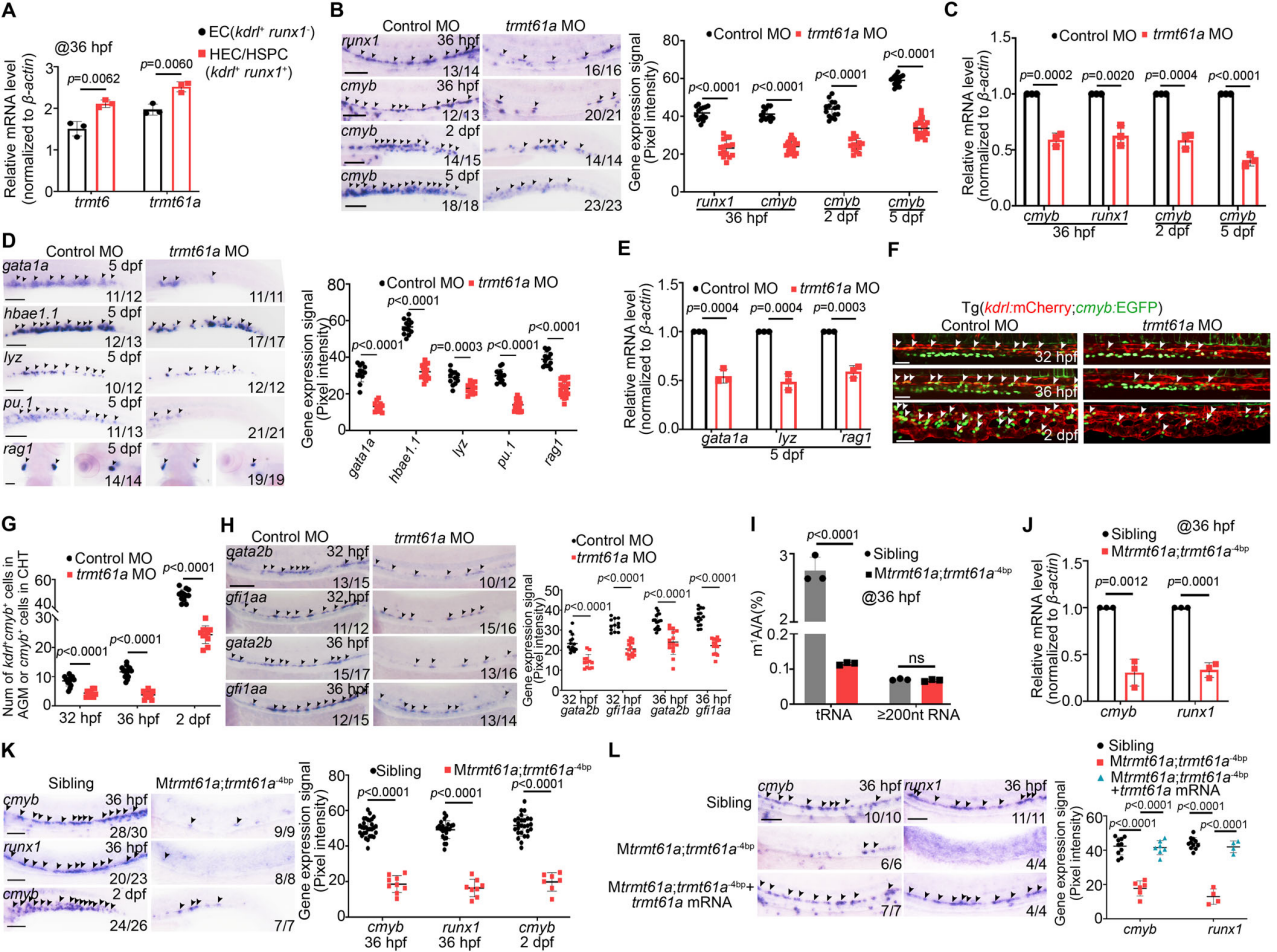
*trmt61a* deficiency leads to abnormal hematopoietic development. (**A**) qPCR analysis showing the relative mRNA expression of *trmt6*, and *trmt61a* at 36 hpf in sorted cells, including *kdrl*^+^*runx1*^-^ EC, *kdrl*^+^*runx1*^+^ HEC/HSPC (three biological replicates). (**B**) WISH results (left) with quantification (right) showing *runx1* expression at 36 hpf in the AGM region, *cmyb* expression at 36 hpf in the AGM region, at 2 dpf and 5 dpf in the CHT region in control MO- and *trmt61a* MO-injected embryos (three biological replicates). (**C**) qPCR results showing *runx1* and *cmyb* expression in the AGM region (36 hpf) or CHT region (2 dpf and 5 dpf) in control MO- and *trmt61a* MO-injected embryos (three biological replicates). (**D**) WISH results (left) and quantification (right) showing the expression of *gata1a*, *hbae1.1*, *lyz*, *pu.1*, and *rag1* at 5 dpf in CHT region of control MO- and *trmt61a* MO-injected embryos (three biological replicates). (**E**) qPCR analysis showing the relative mRNA expression of *gata1a*, *lyz* and *rag1* in CHT region of control MO- and *trmt61a* MO-injected embryos at 5 dpf (three biological replicates). (**F**, **G**) Confocal imaging (**F**) and statistical data (**G**) showing HECs and emerging HSPCs (white arrowheads) in the AGM region at 32 hpf and 36 hpf, and HSPCs (white arrowheads) in CHT region at 2 dpf in control MO- and *trmt61a* MO-injected embryos under Tg(*kdrl*:mCherry;*cmyb*:EGFP) background (three biological replicates). (**H**) WISH results (left) and quantification (right) showing the expression of *gata2b* and *gfi1aa* at 32 hpf and 36 hpf in control MO- and *trmt61a* MO-injected embryos (three biological replicates). (**I**) Quantification of m^1^A/A ratio from siblings and M*trmt61a*; *trmt61a*^-4bp^ embryos at 36 hpf by UHPLC-MS/MS (three biological replicates). (**J**) qPCR analysis showing the relative expression of *cmyb* and *runx1* in AGM region of siblings and M*trmt61a*; *trmt61a*^-4bp^ embryos at 36 hpf (three biological replicates). (**K**) WISH results (left) and quantification (right) showing the expression of HSPC markers *cmyb* and *runx1* in the AGM region at 36 hpf, and *cmyb* expression in the CHT region at 2 dpf, in siblings and M*trmt61a*;*trmt61a*^-4bp^ embryos (three biological replicates). (**L**) WISH results (left) and statistical analysis (right) showing the expression of *cmyb* and *runx1* in siblings, M*trmt61a*;*trmt61a*^-4bp^ embryos and *trmt61a* mRNA-injected M*trmt61a*;*trmt61a*^-4bp^ embryos at 36 hpf (three biological replicates). Error bars represent mean ± SD. Two-tailed unpaired Student’s *t* test (**A**–**E**,** G**–**L**). Arrowheads denote marker gene-positive signals (**B**, **D**, **H**,** K**, **L**). The numbers indicating the number of embryos with representative phenotype/total number of embryos in each group (**B**, **D**, **H**, **K**, **L**). Scale bars: 100 μm (**B**, **D**, **H**, **K**, **L**); 50 μm (**F**). Source data are available online for this figure.

To determine the functional involvement of m^1^A58 modification in hematopoiesis, we first examined the level of tRNA modifications in the AGM region of zebrafish embryos at 36 hours post fertilization (hpf, the timing of HSPC generation), and revealed abundant tRNA m^1^A modification (Appendix Fig. S1A). Then, we assessed the expression of MTC genes, *trmt61a* and *trmt6*, during zebrafish embryogenesis. Whole-mount in situ hybridization (WISH) showed maternal and persistent zygotic expression of both genes, consistent with the presence of m^1^A modification (Appendix Fig. S1B). Analysis of the published single-cell transcriptomic data from endothelial and hematopoietic cells in the AGM at 36 hpf (Xia et al, 2023; data ref: Xia et al, 2023) further revealed high expression of *trmt61a* and *trmt6* in hemogenic endothelium and HSPC, compared with EC (Appendix Fig. S1C). Subsequently, real-time quantitative PCR (qPCR) analysis of the sorted ECs (*kdrl*^+^*runx1*^-^) and HECs/HSPCs (*kdrl*^+^*runx1*^+^) from Tg(*kdrl*:mCherry;*runx1*:en-GFP) embryos at 36 hpf confirmed enrichment of both genes in HECs/HSPCs (Fig. 1A; Appendix Fig. S1D,E), supporting their functional relevance in embryonic hematopoiesis.

To directly examine the role of m^1^A58 modification, we first knocked down the endogenous expression of *trmt61a* using an ATG-targeting morpholino (*trmt61a* MO) (Appendix Fig. S2A). Western blotting and immunofluorescence confirmed substantial reduction of Trmt61a protein (Appendix Fig. S2B,C). Notably, *trmt61a*-deficient embryos exhibited no apparent defects in general morphology (Appendix Fig. S2D), including neurogenesis (*elavl3* and *gad1b*), somite development (*myod1*), and blood vessel development (*kdrl* and *fli1a*) (Appendix Fig. S2E). Subsequently, we sought to investigate whether Trmt61a is required for embryonic hematopoiesis. Primitive erythroid (*gata1a*) and myeloid progenitor genes (*pu.1*) remained unaffected (Appendix Fig. S2F–H). In contrast, definitive hematopoiesis was obviously impaired in *trmt61a* morphants. WISH and qPCR showed significant reductions in *runx1* and *cmyb* expression (HSPC markers) in the AGM and CHT at 36 hpf, 2 days post fertilization (dpf), and 5 dpf (Fig. 1B,C). Additionally, the differentiation towards erythroid (labeled by *gata1a* and *hbae1.1*), myeloid (*pu.1* and *lyz*), and lymphoid (*rag1*) lineages was also compromised (Fig. 1D,E). Using Tg(*kdrl*:mCherry;*cmyb*:EGFP) embryos, we found the number of HECs and emerging HSPCs in the AGM region in *trmt61a* morphants at 32 hpf and 36 hpf was significantly decreased, followed by reduced *cmyb*^+^ HSPCs in the CHT at 2 dpf (Fig. 1F,G). These findings suggest that *trmt61a* deficiency disrupts HSPC generation. Consistently, the expression of HEC markers (*gata2b* and *gfi1aa*) was decreased in morphants (Fig. 1H), whereas arterial markers (*dltc* and *dll4*) remained unchanged (Appendix Fig. S2I). Thus, Trmt61a is required for HSPC production but is dispensable for arterial development.

To ascertain the gene-specificity of the observed HSPC phenotype, we conducted rescue experiments via overexpression of engineered *trmt61a* mRNA, escaping from *trmt61a* MO blocking (Appendix Fig. S2A). Western blotting verified robust Trmt61a overexpression following mRNA injection (Appendix Fig. S2J). Both WISH and live imaging showed that the overexpression of *trmt61a* efficiently restored the expression of *runx1* and *cmyb* and the number of *kdrl*^+^*cmyb*^+^ HEC/HSPCs in *trmt61a* morphants at 36 hpf (Appendix Fig. S2K,L).

Collectively, these results demonstrate that Trmt61a is essential for embryonic HSPC development.

### Knockout of *trmt61a* abrogates HSPC production

To genetically confirm the role of Trmt61a in HSPC generation, we generated a *trmt61a* mutant using CRISPR/Cas9 technology. A 4-bp deletion was introduced into exon 2, resulting in truncation of the protein from 305 to 95 amino acids (Appendix Fig. S3A). Zygotic homozygous *trmt61a* mutants (Z*trmt61a*) were obtained by cross-mating *trmt61a*^*+/*-4bp^ adult zebrafish; however, these mutants failed to survive to adulthood. Western blotting showed that maternal T*rmt61a* protein persisted in Z*trmt61a* up to 4 dpf (Appendix Fig. S3B). Notably, the Z*trmt61a* exhibited relatively normal HSPC development (Appendix Fig. S3C), which were contrasted with the HSPC phenotype observed in *trmt61a* morphants, likely due to maternal contributions.

To eliminate maternal *trmt61a*, we employed a CRISPR/Cas9-mediated oocyte-specific conditional knockout strategy (Zhang et al, 2021). Constructs containing *U6*-driven *trmt61a* sgRNAs tagged with EGFP and I-Sce1 was injected into one-cell embryos under the Tg(*zpc*:*zcas9*) background, in which Cas9 is expressed specifically in oocytes (Appendix Fig. S4A). After screening, we identified an adult female founder fish capable of oocyte gene editing. Cross-mating this female with male *trmt61a*^*+/*-4bp^ fish generated maternal-zygotic *trmt61a* mutants (M*trmt61a*; *trmt61a*^-4bp^, <1% proportion) carrying frameshift mutations in EGFP-positive offspring (Appendix Fig. S4B).

WISH and immunofluorescence confirmed complete loss of *trmt61a* expression, while vascular development was normal (Appendix Fig. S4C–E). Global tRNA m^1^A modification level was significantly reduced in M*trmt61a*; *trmt61a*^-4bp^ embryos (Fig. 1I). Similar to the *trmt61a* morphants, *runx1* and *cmyb* expression in the AGM and CHT was reduced significantly in M*trmt61a*; *trmt61a*^-4bp^ embryos (Fig. 1J,K). Importantly, *trmt61a* mRNA overexpression restored HSPC production in M*trmt61a*; *trmt61a*^-4bp^ embryos (Fig. 1L), confirming that the hematopoietic defects were specifically caused by *trmt61a* loss.

Taken together, generation of maternal-zygotic *trmt61a* mutants confirms the essential role of Trmt61a in HSPC development.

### Trmt61a/Trmt6-mediated tRNA m^1^A58 methylation sustains HSPC generation in a cell-autonomous manner

**Figure 2:**
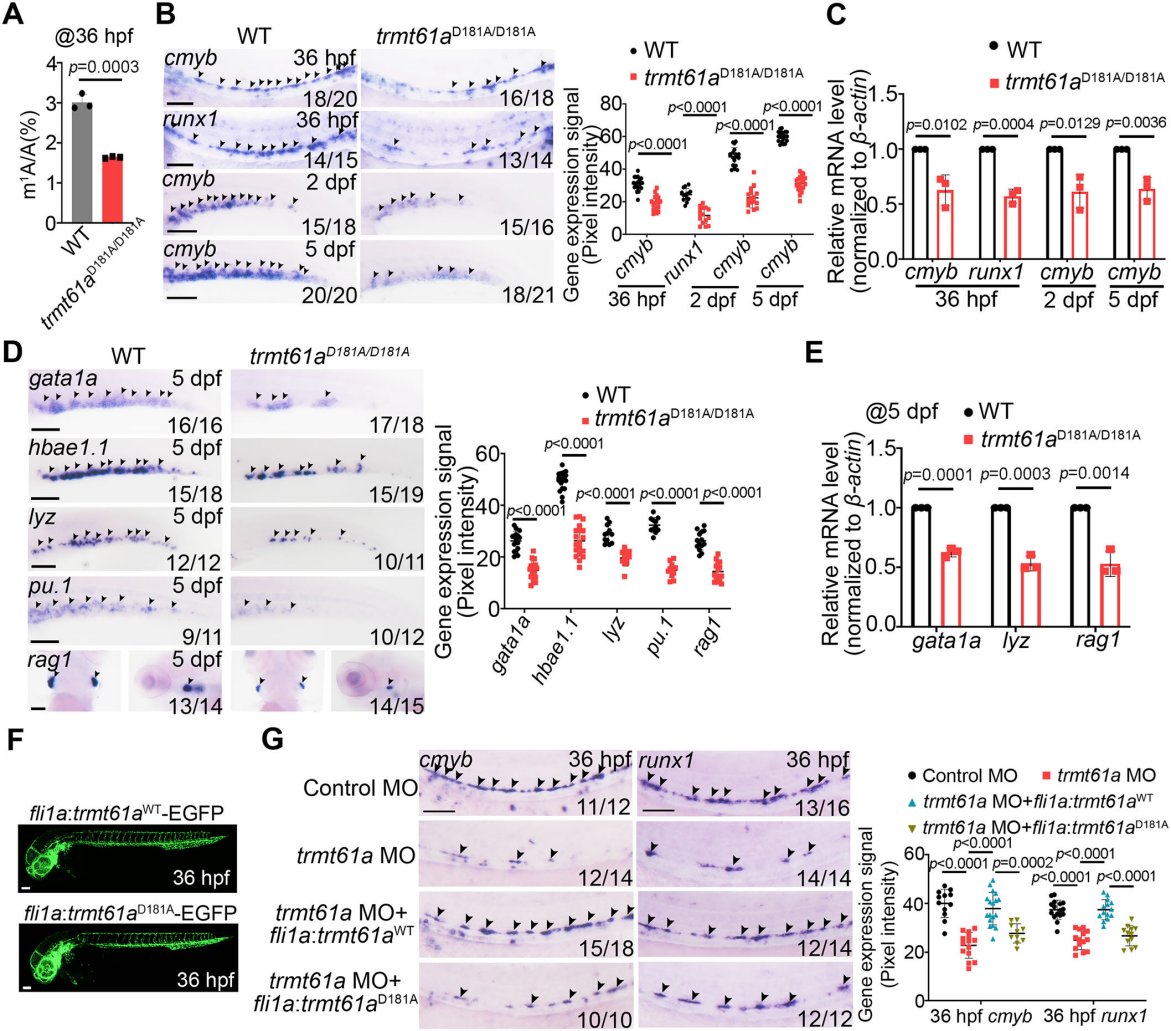
*trmt61a*-mediated tRNA m^1^A58 modification is essential for HSPC production. (**A**) Quantification of m^1^A/A ratio in total tRNA isolated from WT and *trmt61a*^D181A/D181A^ embryos at 36 hpf by UHPLC-MS/MS (three biological replicates). (**B**, **C**) WISH results (**B**, left) with quantification (**B**, right) and qPCR (**C**) showing the expression of *cmyb* and *runx1* in AGM region at 36 hpf, *cmyb* in the CHT region at 2 dpf and 5 dpf in WT and *trmt61a*^D181A/D181A^ embryos (three biological replicates). (**D**) WISH (left) and quantification (right) showing the expression of *gata1a*, *hbae1.1*, *lyz*, *pu.1* and *rag1* at 5 dpf in WT and *trmt61a*^D181A/D181A^ embryos (three biological replicates). (**E**) qPCR analysis showing the relative expression of *gata1a*, *lyz* and *rag1* in WT and *trmt61a*^D181A/D181A^ embryos at 5 dpf (three biological replicates). (**F**) Representative fluorescence images of *fli1a*: *trmt61a*^WT^-EGFP and *fli1a*: *trmt61a*^D181A^-EGFP. (**G**) WISH (left) and quantification (right) showing the expression of *cmyb* and *runx1* in control morphants, *trmt61a* morphants, Tg (*fli1a*: *trmt61a*^WT^-EGFP) embryos injected with *trmt61a* MO, and Tg (*fli1a*: *trmt61a*^D181A^-EGFP) embryos injected with *trmt61a* MO at 36 hpf (three biological replicates). Error bars represent mean ± SD. Two-tailed unpaired Student’s *t* test (**A**–**E**, **G**). Arrowheads denote marker gene-positive signals (**B**, **D**, **G**). The numbers indicating the number of embryos with representative phenotype/total number of embryos in each group (**B**, **D**, **G**). Scale bars: 100 μm (**B**, **D**, **F**, **G**). Source data are available online for this figure.

To determine whether the role of Trmt61a in HSPC generation depends on its enzymatic activity, protein sequence analysis was performed, and showed that Trmt61a was highly conserved across species, with the key catalytic residue Asp181 fully preserved (Finer-Moore et al, 2015) (Appendix Fig. S5A, B). To directly assess catalytic dependency, we generated a catalytic-dead knock-in line *Ki*(*trmt61a*^*D181A*^-EGFP), using CRISPR/Cas9-mediated approach (Li et al, 2015) (Appendix Fig. S5C; “Methods”). The specificity of knock-in events was validated by immunofluorescence, which revealed EGFP co-localization with Trmt61a-positive cells (Appendix Fig. S5D). Western blot analysis further demonstrated that catalytic-dead Trmt61a protein was expressed at levels comparable to wild-type embryos and remained sensitive to *trmt61a* MO-injection (Appendix Fig. S5E). Subsequently, ultra-high performance liquid chromatography coupled to mass spectrometry (UHPLC-MS/MS) analysis demonstrated a marked reduction in global tRNA m^1^A level in *trmt61a*^D181A/D181A^ embryos (Fig. 2A), indicating that Asp181 is a major catalytic residue in zebrafish. Notably, residual m^1^A signals remained detectable, suggesting that additional residues of Trmt61a may also contribute to tRNA m^1^A58 catalysis.

Morphologically, the *trmt61a*^D181A/D181A^ embryos developed normally and showed no obvious abnormalities including the nervous system, muscle development, vascular development, or arterial specification (Appendix Fig. S5F–H). We then examined HSPC development in the *trmt61a*^D181A/D181A^ embryos, and found attenuated expression of *runx1* and/or *cmyb* in the AGM (at 36 hpf) and CHT (at 2 dpf and 5 dpf) (Fig. 2B,C). Erythroid, myeloid, and lymphoid differentiation was also impaired (Fig. 2D,E). Similarly, the M*trmt61a*; *trmt61a*^D181A^ embryos showed decreased m^1^A level and hematopoietic defects (Appendix Fig. S5I–K). These results indicate that Trmt61a promotes HSPC generation, depending on its catalytic effect on tRNA m^1^A58 modification.

Next, to further determine the synergistic function of different components within the m^1^A58 MTC, we then blocked the expression of the RNA-binding component Trmt6 (Appendix Fig. S6A). WISH, qPCR and live imaging showed the impaired HSPC generation upon *trmt6* deficiency (Appendix Fig. S6B–D), consistent with that in *trmt61a*-deficient embryos. Moreover, the decreased expression of *runx1* and *cmyb* in the *trmt*6 morphants was rescued by *trmt6* mRNA (escaping from *trmt6* MO blocking) injection (Appendix Fig. S6E). These findings demonstrate that Trmt6 and Trmt61a cooperatively regulate HSPC development through their combined role as a heterotetrameric complex.

Finally, to determine whether this m^1^A58 modification operates intrinsically within ECs, we applied *fli1a* promoter-driven overexpression of *trmt61a*^WT^ or *trmt61a*^D181A^ transcripts (escaping from *trmt61a* MO blocking) (Fig. 2F). EC-specific rescue experiments demonstrated that *trmt61a*^WT^, but not the catalytic-dead *trmt61a*^D181A^ variant, significantly restored *runx1* and *cmyb* expression in the *trmt61a*-deficient embryos (Fig. 2G). Together, these data support that Trmt61a regulates HSPC generation in EC-autonomous and m^1^A58-dependent manners.

### Trmt61a deficiency compromises HSPC production through *p53*-dependent apoptosis

**Figure 3:**
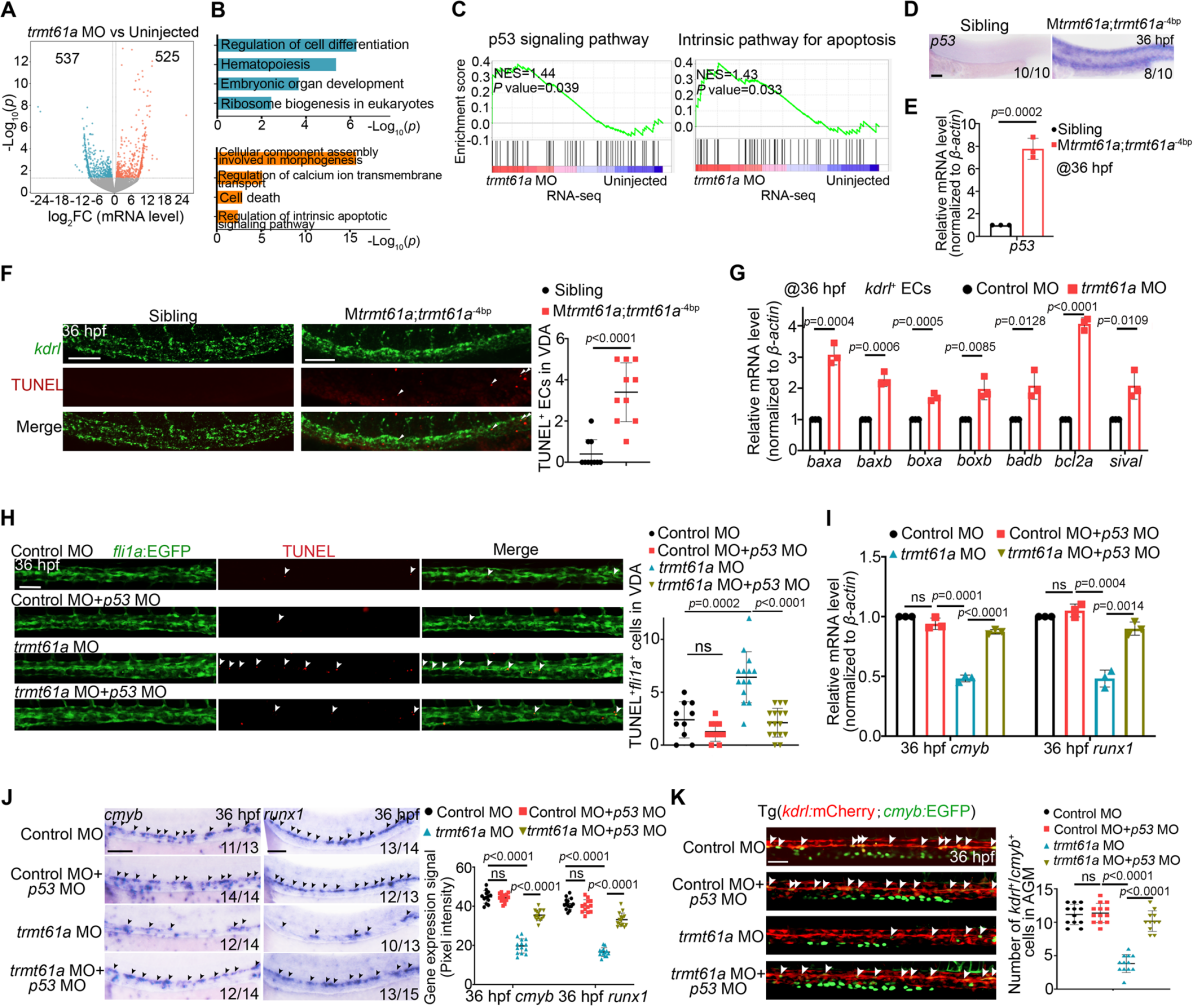
Trmt61a deficiency activates p53-dependent apoptosis. (**A**) Volcano plots of RNAseq data at 36 hpf showing the differentially expressed genes between uninjected embryos and *trmt61a* morphants (two biological replicates). The *P* value was calculated using the Wald test. (**B**) Representative gene ontology biological process categories enriched in downregulated (upper panel) and upregulated mRNAs (lower panel) in *trmt61a* morphants (two biological replicates). The *P* value was calculated using the Hypergeometric test. (**C**) GSEA enrichment analysis of RNAseq showing p53 signaling pathway and intrinsic pathway for apoptosis were enriched in *trmt61a* morphants (two biological replicates). The *P* value was calculated using the Permutation test. (**D**, **E**) WISH (**D**) and qPCR (**E**) showing the expression of *p53* in sibling and M*trmt61a*;*trmt61a*^-4bp^ embryos at 36 hpf (three biological replicates). (**F**) TUNEL results (left) and statistical analysis (right) showing increased proportion of apoptotic ECs (TUNEL^+^ *kdrl*^+^; white arrowheads) in the ventral wall of dorsal aorta (VDA) in M*trmt61a*;*trmt61a*^-4bp^ embryos, compared with that in siblings at 36 hpf (three biological replicates). (**G**) qPCR analysis showing the relative mRNA expression of apoptosis-related genes in *kdrl*^+^ ECs sorted from control MO- and *trmt61a* MO-injected embryos at 36 hpf (three biological replicates). (**H**) TUNEL results (left) and statistical analysis (right) showing the number of apoptotic ECs (TUNEL^+^ *fli1a*^+^; white arrowheads) in the VDA in control MO-, control+*p53* MOs-, *trmt61a* MO-, and *trmt61a* + *p53* MOs-injected embryos at 36 hpf (three biological replicates). (**I**) qPCR analysis showing the relative expression of *cmyb* and *runx1* in control MO-, control+*p53* MOs-, *trmt61a* MO-, and *trmt61a* + *p53* MOs-injected embryos at 36 hpf (three biological replicates). (**J**) WISH (left) and quantification (right) showing the expression of *cmyb* and *runx1* in control MO-, control+*p53* MOs-, *trmt61a* MO-, and *trmt61a* + *p53* MOs-injected embryos at 36 hpf. Arrowheads denote *runx1* or *cmyb* positive signals (three biological replicates). (**K**) Confocal imaging (left) and statistical data (right) showing the number of HECs and emerging HSPCs (white arrowheads) in AGM region at 36 hpf in control MO-, control+*p53* MOs-, *trmt61a* MO-, and *trmt61a* + *p53* MOs-injected embryos under Tg(*kdrl*:mCherry;*cmyb*:EGFP) background (three biological replicates). Error bars represent mean ± SD. Two-tailed unpaired Student’s *t* test (**E**–**K**). The numbers indicating the number of embryos with representative phenotype/total number of embryos in each group (**D**, **J**). Scale bars: 100 μm (**D**, **J**); 50 μm (**F**, **H**, **K**). Source data are available online for this figure.

To investigate the mechanism underlying impaired HSPC generation, we conducted transcriptomic analysis on *kdrl*^+^ ECs from the AGM region of uninjected embryos and *trmt61a* morphants at 36 hpf (Appendix Fig. S7A). Differentially expressed genes (DEG) analysis identified 537 downregulated and 525 upregulated transcripts in *trmt61a*-deficient embryos (Fig. 3A). Gene ontology (GO) enrichment analysis revealed that downregulated genes were associated with hematopoiesis, while upregulated genes were primarily linked to apoptotic pathways (Fig. 3B). Consistent with these findings, gene set enrichment analysis (GSEA) confirmed the significant upregulation of *p53*-related signaling and the intrinsic pathway for apoptosis (Fig. 3C).

Additionally, WISH and qPCR validated altered expression of *p53* in M*trmt61a*; *trmt61a*^-4bp^ embryos (Fig. 3D,E), indicating that the *trmt61a* loss activates *p53*-mediated apoptosis. TUNEL assays demonstrated significantly increased apoptosis in the VDA of *trmt61a* knockout embryos (Fig. 3F), accompanied by increased expression of apoptosis-related genes (*baxa*, *baxb*, *boxa*, *boxb*, *badb*, *bcl2a*, and *sival*) (Fig. 3G). These findings indicated that the observed HEC/HSPC defects were attributable to altered apoptosis.

To test this, we performed *p53* loss-of-function analysis. TUNEL results showed reduced apoptotic signals in the VDA of *trmt61a*-deficient embryos following *p53* knockdown (Fig. 3H). Critically, WISH, qPCR, and live imaging results revealed that *p53* knockdown restored HSPC production in *trmt61a*-deficient embryos (Fig. 3I–K). These results indicate that *trmt61a* deficiency impairs HSPC generation primarily through *p53*-dependent apoptosis.

### Trmt61a deficiency impairs mitochondrial biogenesis via Nrf1 downregulation

**Figure 4:**
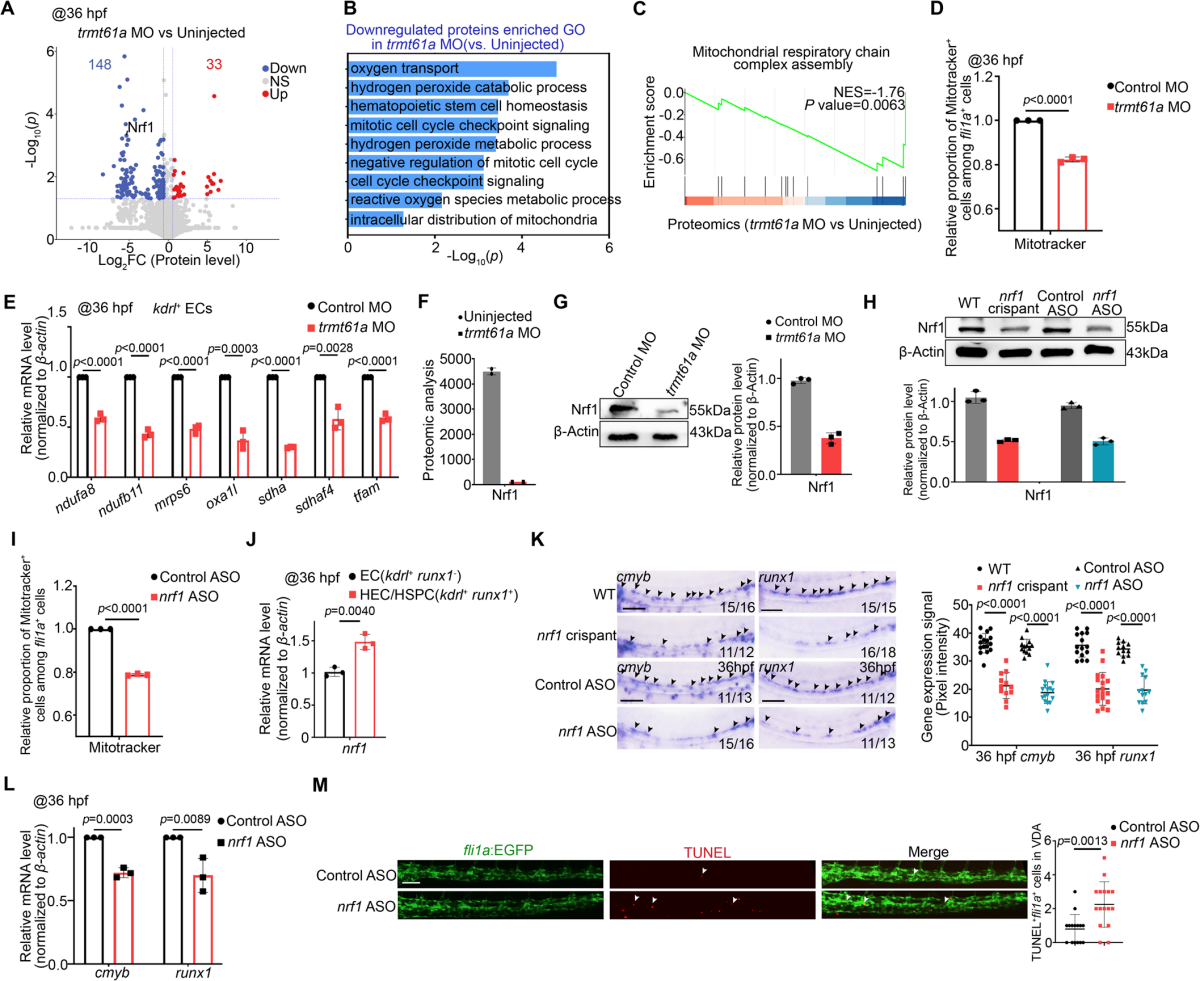
Nrf1-mediated mitochondrial homeostasis is essential for HSPC development. (**A**) Volcano plots of proteomic data at 36 hpf showing the differentially expressed proteins in *trmt61a* morphants and uninjected embryos. Red dots represent genes with increased protein levels, while blue dots represent genes with decreased protein levels in *trmt61a* morphants (two biological replicates). (**B**) Representative gene ontology biological process categories enriched in downregulated proteins in *trmt61a* morphants. (**C**) GSEA enrichment analysis of proteomics showing mitochondrial respiratory chain complex assembly was enriched in *trmt61a* morphants. (**D**) Mitochondrial content in *fli1a*^+^ ECs in *trmt61a* morphants and control MO-injected embryos at 36 hpf (three biological replicates). (**E**) qPCR analysis showing the relative expression of mitochondrial-related genes in *kdrl*^+^ ECs from control MO- and *trmt61a* MO-injected embryos at 36 hpf (three biological replicates). (**F**) Proteomic data analysis showing the Nrf1 protein level in *trmt61a* morphants and uninjected embryos (two biological replicates). (**G**) Western blotting results (left) and quantification (right, three technical replicates) showing Nrf1 protein level in control MO- and *trmt61a* MO-injected embryos at 36 hpf (*n* = 30 embryos). (**H**) Western blotting of Nrf1 protein (upper) and quantification (lower, three technical replicates) in WT, *nrf1* crispants, and control ASO- and *nrf1* ASO-injected embryos at 36 hpf (*n* = 30 embryos). (**I**) Mitochondrial content was detected using mitotracker in *fli1a*^+^ ECs in *nrf1-*deficient (*nrf1* ASO) and control ASO-injected embryos at 36 hpf (three biological replicates). (**J**) qPCR analysis showing the relative mRNA expression of *nrf1* in *kdrl*^+^*runx1*^-^ EC, *kdrl*^+^*runx1*^+^ HEC/HSPC at 36 hpf. (three biological replicates). (**K**) WISH (left) and quantification (right) showing the expression of *cmyb* and *runx1* in WT, *nrf1* crispants, and control ASO- and *nrf1* ASO-injected embryos at 36 hpf. Arrowheads denote *runx1* or *cmyb* positive signals (three biological replicates). The numbers indicating the number of embryos with representative phenotype/total number of embryos in each group. Scale bars: 100 μm. (**L**) qPCR analysis showing the relative mRNA expression of *cmyb* and *runx1* in control ASO- and *nrf1* ASO-injected embryos at 36 hpf (three biological replicates). (**M**) TUNEL results (left) and statistical analysis (right) showing the number of apoptotic ECs (TUNEL^+^ *fli1a*^+^; white arrowheads) in the VDA in control ASO-, or *nrf1* ASO-injected embryos at 36 hpf. Scale bars: 50 μm (three biological replicates). Error bars represent mean ± SD. Two-tailed unpaired Student’s *t* test (**D**, **E**, **I**–**M**). Source data are available online for this figure.

Given the crucial role of Trmt61a in translation control (Sun et al, 2023), we next performed proteomic analysis of *kdrl*^+^ ECs in AGM regions from uninjected embryos and *trmt61a* morphants at 36 hpf (Appendix Fig. S7A). Proteomic analysis revealed that *trmt61a* knockdown resulted in 148 downregulated and 33 upregulated proteins (Fig. 4A), indicating reduced global protein synthesis. This finding was corroborated by puromycin incorporation assay, which demonstrated decreased level of newly synthesized protein (Appendix Fig. S7B). Specifically, the downregulated proteins were significantly enriched in pathways related to oxygen transport, hydrogen peroxide catabolism, reactive oxygen species (ROS) metabolic and mitochondria dynamics (Fig. 4B), indicative of disrupted mitochondrial function. GSEA further revealed downregulation of the mitochondrial respiratory chain complex assembly in *trmt61a*-deficient embryos (Fig. 4C). Furthermore, flow cytometry analysis confirmed reduced mitochondrial content in ECs (Fig. 4D), while qPCR demonstrated decreased expression of mitochondrial biogenesis-related genes (Fig. 4E). These findings suggest impaired mitochondrial biogenesis in ECs following *trmt61a* deficiency.

Importantly, proteomic analysis revealed significantly reduced Nrf1 protein levels upon *trmt61a* deficiency (Fig. 4F), a finding confirmed by western blotting (Fig. 4G). Of note, NRF1 serves as a master regulator of mitochondrial biogenesis (Hu et al, 2022; Zhao et al, 2023). Downregulation of NRF1 contributes to mitochondrial dysfunction (Wang et al, 2006). Consequently, we hypothesized that *trmt61a* deficiency causes Nrf1 loss, which contributes to the impaired mitochondrial biogenesis during HSPC generation. To test this, we performed loss-of-function analysis. Nrf1 deficiency, either in antisense oligonucleotides (ASO)-injected embryos or in transient mutants (i.e., crispant), similarly led to decreased Nrf1 protein (Fig. 4H), diminished expression of mitochondrial biogenesis-related genes (Appendix Fig. S7C) and reduced mitochondrial content relative to control embryos (Fig. 4I).

Next, we sought to determine whether *nrf1* regulates HSPC generation. WISH results showed ubiquitous expression of *nrf1* in zebrafish embryos from 1-cell stage to 2 dpf (Appendix Fig. S7D). However, integration of scRNA transcriptomic data (Xia et al, 2023; data ref: Xia et al, 2023) with qPCR analysis revealed progressive *nrf1* upregulation during the transition from ECs to HECs and then to HSPCs (Fig. 4J; Appendix Fig. S7E), suggesting a potential role for Nrf1 in definitive hematopoiesis. Notably, *nrf1* deficiency phenocopied *trmt61a* loss, causing pronounced downregulation of HSPC genes (*runx1* and *cmyb*) at 36 hpf (Fig. 4K,L) and elevated apoptotic signal in ECs in the AGM region (Fig. 4M).

Taken together, these findings demonstrate that *trmt61a* deficiency impairs HSPC production primarily via Nrf1-dependent disruption of mitochondrial biogenesis.

### Trmt61a facilitates Nrf1 translation to maintain HSPC generation

**Figure 5:**
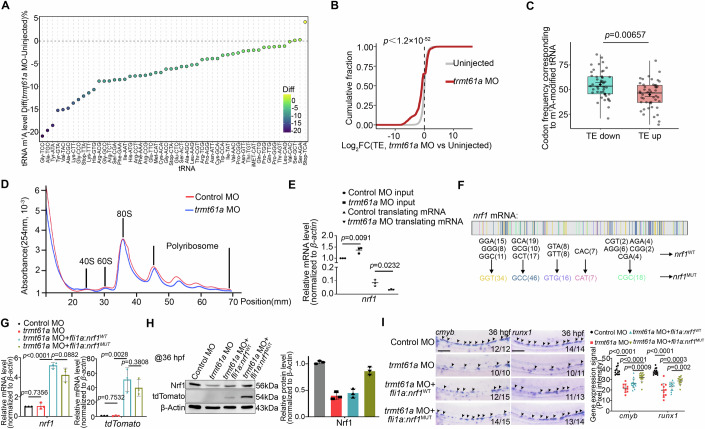
Trmt61a regulates the translation efficiency of Nrf1 in an m^1^A58-dependent manner. (**A**) The percentage decrease in tRNA-m1A58 levels for different tRNA species at 36 hpf, in *trmt61a* morphants relative to uninjected embryos (two biological replicates). (**B**) Cumulative distribution of translation efficiency (TE) between *trmt61a* morphants and uninjected embryos at 36 hpf (two biological replicates). Statistics by Wilcoxon test. (**C**) Frequency of codons corresponding to m^1^A58-modified tRNAs (*n* = 47) in the genes with decreased- or increased translation efficiency, respectively. Statistics by Two-tailed paired Student’s *t* test. (**D**) Polysome profiling showing the representative trace of ribosomes extracted from trunk region in control MO- and *trmt61a* MO-injected embryos at 36 hpf (three biological replicates). (**E**) Ribosome occupancy on input and translating *nrf1* mRNAs was measured by qPCR in both control MO- and *trmt61a* MO-injected embryos at 36 hpf (three biological replicates). (**F**) Schematic diagram of the codon switch assay. The upper panel represents the coding sequence of *nrf1*, with the positions of substituted codons indicated in different colors; the middle panel lists the codons and counts to be replaced (*nrf1*^WT^); and the lower panel displays the synonymous codons and counts after substitution (*nrf1*^MUT^). (**G**) The mRNA level of *nrf1* and *tdTomato* at 36 hpf in control MO-, *trmt61a* MO-injected, *trmt61a* MO and *fli1a*:*nrf1*^WT^-tdTomato or *fli1a*:*nrf1*^MUT^-tdTomato co-injected embryos (three biological replicates). (**H**) The protein level of Nrf1 and tdTomato (Left) and quantification (Right, three technical replicates) at 36 hpf in control MO-, *trmt61a* MO-injected, *trmt61a* MO and *fli1a*:*nrf1*^WT^-tdTomato or *fli1a*:*nrf1*^MUT^-tdTomato co-injected embryos (*n* = 30 embryos). (**I**) WISH (left) with quantification (right) showing the expression level of *cmyb* and *runx1* in control MO-, *trmt61a* MO-injected, *trmt61a* MO and *fli1a*:*nrf1*^WT^-tdTomato or *fli1a*:*nrf1*^MUT^-tdTomato co-injected embryos at 36 hpf. Arrowheads denote *runx1* or *cmyb* positive signals. The numbers indicating the number of embryos with representative phenotype/total number of embryos in each group (three biological replicates). Scale bars: 100 μm. Error bars represent mean ± SD. Two-tailed unpaired Student’s *t* test (**E**, **G**, **I**). Source data are available online for this figure.

To evaluate how Trmt61a-mediated tRNA-m^1^A58 modification regulates translation, we employed tRNA-m^1^A-seq and ribosome profiling sequencing (Ribo-seq) (Appendix Fig. S8A). tRNA-m^1^A-seq analysis suggested a broad reduction of m^1^A58 levels across most tRNAs in *trmt61a* -deficient embryos (Fig. 5A), without significant changes in tRNA abundance or fragmentation patterns (Appendix Fig. S8B–D). Ribo-seq analysis revealed a global decrease in translation efficiency (TE) upon *trmt61a* deficiency (Fig. 5B). Codon dependence analysis revealed that genes with reduced TE preferentially utilized codons decoded by m^1^A58-hypomodified tRNAs following *trmt61a* deficiency, contrasting with genes with increased TE (Fig. 5C). Functional enrichment analysis of the TE-reduced genes identified multiple overrepresented functional categories, including aerobic respiration, protein stabilization and additional signaling pathways (Appendix Fig. S8E). Collectively, these findings together demonstrate that diminished m^1^A58 modifications in tRNAs impair TE of codon-biased transcripts, including genes involved in mitochondrial function, protein quality control, and other signaling processes.

To further determine whether Nrf1 translation is regulated by *trmt61a*-mediated tRNA-m^1^A58, we performed polysome profiling on the AGM region. Results revealed that while *nrf1* mRNA abundance was modestly increased, its association with polysomes was significantly reduced (Fig. 5D,E). These results suggest that Nrf1 is translationally suppressed in the absence of functional Trmt61a. To test whether impaired Nrf1 translation contributes to *trmt61a*-deficient phenotypes, we engineered a codon-optimized *nrf1* cDNA variant with synonymous codon replacements to favor translation in the absence of functional Trmt61a and expressed it under the endothelial-specific *fli1a* promoter (Fig. 5F). Following microinjection of *fli1a*-*nrf1*^WT^-*tdTomato* and *fli1a*-*nrf1*^MUT^-*tdTomato* into *trmt61a* morphants, we observed comparable mRNA expression of *nrf1* and *tdTomato* between two samples (Fig. 5G). Critically, while *fli1a*-*nrf1*^WT^ expression in *trmt61a* morphants produced no significant changes in Nrf1 or tdTomato protein levels, the codon-optimized *fli1a*-*nrf1*^MUT^ construct was efficiently expressed (Fig. 5H). As anticipated, this codon-optimized *nrf1* construct restored Nrf1 protein abundance and significantly rescued defective HSPC generation in *trmt61a*-deficient embryos (Fig. 5H,I), whereas the WT *nrf1* construct yielded only a modest increase in protein expression without functional rescue (Fig. 5H,I). These results suggest Trmt61a regulates HSPC production primarily by controlling Nrf1 translation in ECs.

Taken together, these findings demonstrate that endothelial Trmt61a is essential for preserving Nrf1 translation required for proper HSPC development.

## Discussion

**Figure 6:**
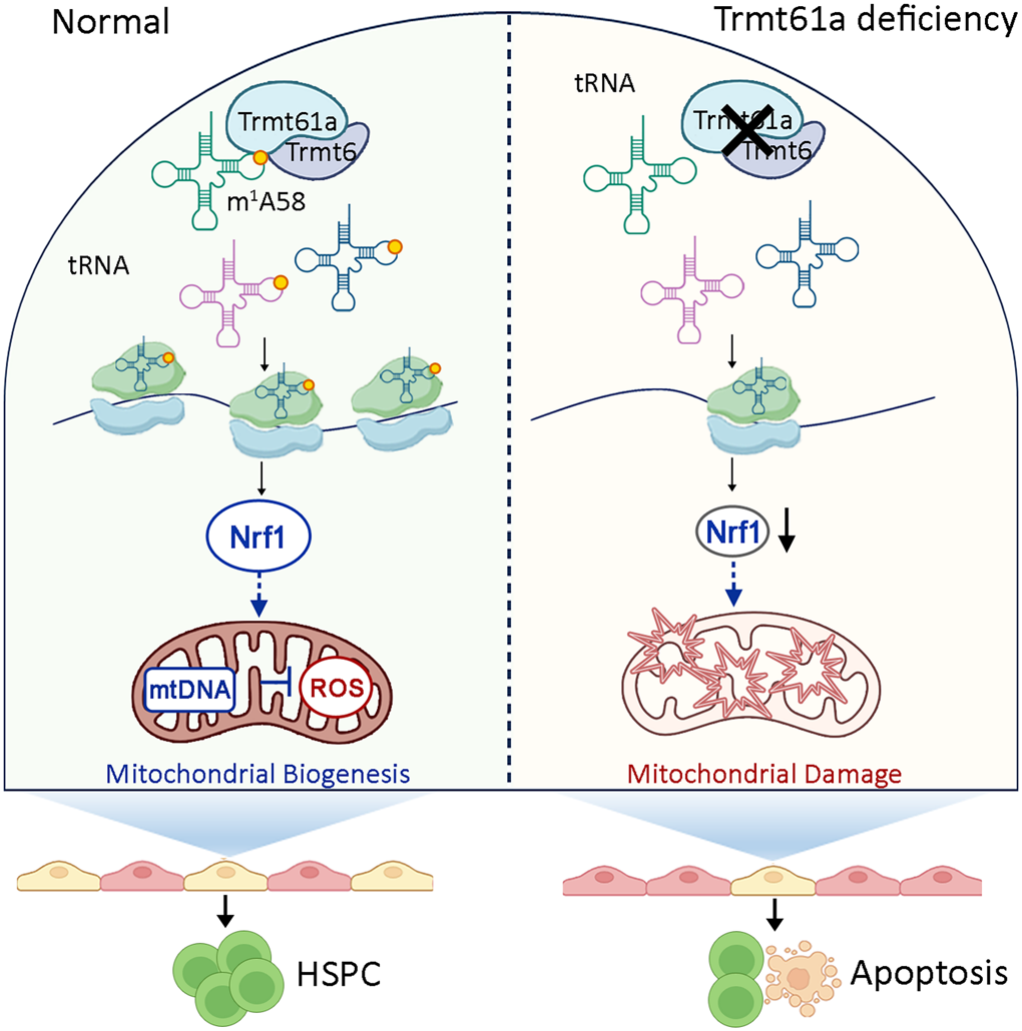
Schematic illustrating the underlying mechanism of Trmt61a-mediated tRNA m^1^A58 modification in HSPC generation. Trmt61a/ Trmt6 mediated m^1^A58 modification participates in HSPC generation by promoting Nrf1 protein translation, thereby governing mitochondrial biogenesis and facilitating HSPC generation during hematopoiesis. Deletion of *trmt61a* inhibits Nrf1 synthesis, thereby leading to mitochondria defects and apoptosis, which subsequently impairs HSPC production.

In this study, we identify that tRNA m^1^A58 modification as a pivotal translational regulator safeguards HSPC generation. Specifically, Trmt61a/Trmt6-mediated m^1^A58 enhances translation of Nrf1, which serves as the master regulator of mitochondrial biogenesis. This mechanism protects HECs/HSPCs by preserving mitochondrial integrity to avoid apoptosis during EHT (Fig. 6). These findings uncover a previously unrecognized function of tRNA m^1^A58 modification in modulating protein synthesis indispensable for embryonic hematopoiesis.

Translational control enables rapid cellular adaptation to developmental cues by bypassing de novo transcription while sustaining bulk protein synthesis (Giguere et al, 2024; Miao et al, 2025). tRNA modifications serve as critical epitranscriptomic regulators of this process (Zhang and Lu, 2025). Among them, tRNA m^1^A58 modification represents one of the most abundant and evolutionarily conserved tRNA modifications. Previous studies have implicated the TRMT61A/TRMT6 complex in adult HSC through both m^1^A-dependent translational regulation (Zuo et al, 2024) and additional non-canonical functions (He et al, 2024). However, its role during embryonic hematopoiesis has remained unclear. Here, we identify tRNA m^1^A58 as a translational regulator specifically required for embryonic HSPC emergence. Additionally, we demonstrated that *trmt61a* deficiency did not alter global tRNA abundance, individual tRNA species or tRNA-derived fragment profiles in zebrafish embryos. Unaltered the initiator tRNA^Met^ levels further suggest that translation initiation is largely preserved. Together, these findings support a model in which tRNA m^1^A58 likely fine-tunes the translation elongation of specific transcripts (e.g., Nrf1) during zebrafish hematopoiesis.

Among the affected targets, Nrf1 emerges as a critical downstream effector. While *nrf1* transcript levels remain largely preserved, its polysome association and protein abundance are markedly reduced upon *trmt61a* deficiency-induced m^1^A58 loss, indicating that this Trmt61a-Nrf1 regulation axis occurs at the translational level. As Nrf1 is a transcription factor governing mitochondrial gene expression and redox balance (Chow et al, 2019; Hu et al, 2022; Scarpulla, 2012), diminished Nrf1 protein is expected to propagate into downstream transcriptional changes, including activation of stress-responsive programs such as the *p53*-dependent apoptotic pathway.

In vertebrates, EHT is accompanied by a metabolic shift toward oxidative phosphorylation (Azzoni et al, 2021; Pv et al, 2024) and increased mitochondrial biogenesis (Prakash and Inamdar, 2025). Our findings show that *nrf1* expression progressively increases during EHT, paralleling increased mitochondrial content and demand, whereas impaired Nrf1 translation disrupts mitochondrial biogenesis and reduces mitochondrial content. Because mitochondria act as a central hub for intrinsic apoptosis (Desagher and Martinou, 2000; Nguyen et al, 2023), mitochondrial dysfunction likely sensitizes HECs to stress-induced cell death. Elevated *p53* signaling and apoptosis in *trmt61a*-deficient embryos thus represent downstream consequences of translationally induced mitochondrial imbalance.

In conclusion, our findings define a translational regulation during embryonic hematopoiesis in which tRNA m^1^A58-dependent control of selective protein synthesis safeguards mitochondrial integrity, restrains *p53*-mediated apoptosis, and ensures proper HSPC emergence. These insights offer novel avenues for in vitro HSPC induction and regenerative therapies.

## Methods

**Table:** Reagents and tools table

Reagent/resource	Reference or source	Identifier or catalog number
**Experimental models**		
Tg(*kdrl*:mCherry) *(D. rerio)*	Bertrand et al, 2010	
Tg(*cmyb*:EGFP) *(D. rerio)*	North et al, 2007	
Tg(*fli1a*:EGFP) *(D. rerio)*	Lawson and Weinstein, 2002	
Tg(*runx1*:en-GFP) *(D. rerio)*	Zhang et al, 2015	
Tg(*zpc*:zcas9) *(D. rerio)*	Zhang et al, 2021	
*trmt61a*^+/-4bp^ *(D. rerio)*	This study	
*Ki*(*trmt61a*^D181A^-GFP) *(D. rerio)*	This study	
Tg(*fli1a*: *trmt61a*^WT^-EGFP)*(D. rerio)*	This study	
Tg(*fli1a*: *trmt61a*^D181A^-EGFP) *(D. rerio)*	This study	
**Antibodies**		
Polyclonal β-Actin Antibody	Cell Signaling Technology	4967S
anti-Trmt61a antibody	Invitrogen	PA5-88013
Polyclonal Anti-Trmt6 Antibody	Invitrogen	PA5-65616
Rabbit Nuclear respiratory factor 1 Polyclonal antibody	Proteintech	12482-1-AP
TdTomato Monoclonal antibody	Cell Signaling Technology	20163
Monoclonal Anti-1-methyladenosine Antibody	MBL	D345-3
alkaline phosphatase-conjugated anti-Dig antibody	Roche	11093274910
POD conjugated anti-Dig antibody	Roche	11633716001
**Oligonucleotides and other sequence-based reagents**		
**Morpholinos and antisense oligonucleotides**		
trmt61a MO	5’-CATGAGGTACAGCTTTAGTGACAAT-3’ (this study)	
Control MO	5’-CCTCTTACCTCAGTTACAATTTATA-3’	
trmt6 MO	5’-CCATTTGTTGGATTGAGACCGTCCG-3’ (this study)	
P53 MO	5’-GCGCCATTGCTTTGCAAGA-3’ (Plaster et al, 2006)	
nrf1 ASO	5’-AUAAGTGAGACTGTTCCAUC-3’ (this study)	
control ASO	5′-GCGUATTATAGCCGATTAAC-3′(this study)	
Probe synthesis primers	This study	Table EV1
Genotyping primers	This study	Table EV1
sgRNAs	This study	Table EV1
qPCR primers	This study	Table EV1
**Chemicals, enzymes and other reagents**		
O-Propargyl-Puromycin	MCE	HY-15680
HiFi DNA Assembly Master Mix	NEBuilder	E2621S
TrueCut™ Cas9 Protein v2	Invitrogen	A36496
I-SceI	NEB	R0694
BM purple	Roche	11442074001
TRIzol Reagent	Invitrogen	15596018CN
MitoTracker	Invitrogen	M22426
optimal cutting temperature	SAKURA	4583
SuperReal PreMix Plus	TIANGEN	FP 205
M-MLV Reverse Transcriptase	Promega	M1701
AlkB	Beyotime	R0639S
T4 RNA ligase 1	NEB	M0204L
TRIzol™ LS	Invitrogen	10296010CN
TSA Plus Fluorescein Solution	PerkinElmer	NEL741E001KT
digoxigenin labeling NTPs mixtures	Roche	11277073910
Induro RTase	NEB	M0681S
**Software**		
Flow Jo v10	BD Life Sciences	
QuantStudio3 Real-Time PCR System	Thermo Scientific	
ImarisViewer	Oxford Instruments	
ImageJ	National Institutes of Health	
GraphPad Prism 8.0	GraphPad Software	
**Other**		
mMESSAGE mMACHINE™ SP6 kit	Invitrogen	AM1340
In Situ Cell Death Detection Kit (TMR red)	Roche	12156792910
Click-iT™ Cell Reaction Buffer cocktail	Invitrogen	C10269
MEGAclear™ Kit	Invitrogen	AM1908
RevertAid RT Kit	Thermo Scientific	K1691
Andor Dragonfly 505 confocal microscope	Oxford Instruments	
KAPA Hyper Prep Kit	Roche	KK8504
AMPure XP beads	Beckman	A63882
Zymo RNA kit	ZYMO RESEARCH	R1016
MyOne Silane Beads	Invitrogen	37002D
small RNA library preparation kit	VAZYME	NR801
RNA-Seq kit	YEASEN	12309ES24

### Zebrafish strains

Adult zebrafish including Tübingen strain, Tg(*kdrl*:mCherry), Tg(*cmyb*:EGFP, Tg(*fli1a*:EGFP), Tg(*runx1*:en-GFP), Tg(*zpc*:zcas9) (provided by Ming Shao), *trmt61a*^+/-4bp^, *Ki*(*trmt61a*^D181A^-EGFP), Tg(*fli1a*: *trmt61a*^WT^-EGFP), Tg(*fli1a*: *trmt61a*^D181A^-EGFP) were maintained in system water under standard conditions at 28.5 °C. Zebrafish embryos and larvae were obtained through natural spawning following previously described staging (Kimmel et al, 1995) and collection procedures (Li et al, 2022). Animal experiments were performed in compliance with ethical guidelines approved by the Ethics Committee of the Institute of Hematology, Chinese Academy of Medical Sciences.

### Morpholino, antisense oligonucleotide, vector construction, mRNA synthesis, and microinjection

MOs in this study were purchased from Gene Tools and were injected into 1-cell stage embryos (4 ng for *trmt61a* MO, 4 ng for *trmt6* MO, 4 ng for control MO and *p53* MO, 2 ng for low-dose of *trmt61a* MO per embryo). The *nrf1* antisense oligonucleotides (ASO) and control ASO were synthesized by GenePharma and 100 pg of ASO was injected into 1-cell stage embryo.

For mRNAs synthesis and injection, the full length CDS of *trmt61a* (ENSDART00000003517.9, escaping from *trmt61a* MO blocking) or *trmt6* mRNA (ENSDART00000062092.7, escaping from *trmt6* MO blocking) was cloned into a pCS2 plasmid and the mRNAs were synthesized using mMESSAGE mMACHINE™ SP6 kit (AM1340, Invitrogen) and 100 pg per embryo was injected into embryos at 1-cell stage.

For rescue experiment, the full length CDS of either *trmt61a* or mutated *trmt61a*^D181A^ sequences was cloned into the pDestTol2pA2 vector with *fli1a* promoter and EGFP using HiFi DNA Assembly Master Mix (E2621S, NEBuilder). Constructs (30-40 pg per embryo) were co-injected with *tol2* mRNA (30-40 pg per embryo) into embryos at 1-cell stage.

### Generation of *trmt61a*^-4bp^ Mutant and maternal *trmt61a* Mutant using CRISPR/Cas9

The *trmt61a* mutant was generated using CRISPR/Cas9 technology as previously described (Chang et al, 2013). The *trmt61a* sgRNA targeting exon 2 (5’-TAGAGGATCTGCGTTCGGTG-3’) was designed in https://www.benchling.com/crispr. The Cas9 protein (A36496, Invitrogen) and *trmt61a* sgRNA were co-injected into 1-cell stage embryos for genome editing. The identification of *trmt61a* mutant with 4 base pairs deletion was carried out by PCR and Sanger sequencing, with primers listed in Table EV1. Zygotic homozygous *trmt61a* mutants (Z*trmt61a*) were generated by cross-mating *trmt61a*^+/-4bp^ adult zebrafish.

For the generation of maternal *trmt61a* mutant, four high-efficiency sgRNAs targeting exon 2 of *trmt61a* (sgRNA 1: 5’-TCAGACTCAAACTCGCTACG-3’; sgRNA 2: 5’-GCTCAGACTCAAACTCGCTA-3’; sgRNA 3: 5’-TGCACCCTACACCAGAGCTG-3’; sgRNA 4: 5’-AGGTTGATAGTCCACAGCTC-3’) were designed in https://www.benchling.com/crispr. Using HiFi DNA Assembly Master Mix, four sgRNA expression modules driven by *U6* promoter were connected to generate pGGDestISceIEG-4sgRNA construct tagged with an EGFP reporter. The construct was co-injected with I-SceI (R0694, NEB) into 1-cell stage embryos of Tg(*zpc*: zcas9) background as previously described (Zhang et al, 2021). After identifying maternal EGFP expression and genotyping, the maternal mutant-producing female fish was subsequently outcrossed with male *trmt61a*^+/-4bp^ to produce M*trmt61a*; *trmt61a*^-4bp^ embryos. Then, EGFP-positive embryos were individually extracted genome to analyze their genotypes. The primers used for validating maternal *trmt61a* mutant were listed in Table EV1.

### Generation of *Ki*(*trmt61a*^D181A^-EGFP) using CRISPR/Cas9

To generate *Ki*(*trmt61a*^D181A^-EGFP) zebrafish line, sgRNA targeting intron 2 (5’-GAGTCATTTAAGGTTGTGGG-3’) was designed in https://www.benchling.com/crispr. The donor construct was designed based on established method (Li et al, 2015) and generated using HiFi DNA Assembly Master Mix. It comprises three core components: a left arm, a P2A-EGFP coding sequence, and a right arm. To preserve the full coding sequence of *trmt61a*, the left arm begins from the upstream of the sgRNA target site in intron 2, spans the whole exon 3 which harbors a specific point mutation (c.542 A > C; p.D181A), and terminates at the last base just before the stop codon of *trmt61a* in exon 4. The right arm contains the stop codon and 3′ untranslated region of *trmt61a*. Furthermore, to facilitate the identification of successfully edited embryos, the donor plasmid incorporates a cardiac-specific EGFP reporter, driven by the *myl7* promoter.

The donor construct was co-injected with sgRNA and Cas9 protein into 1-cell stage embryos. Larvae exhibiting cardiac EGFP expression were selected and raised to adulthood. Founders with germline transmission were identified through fluorescence pattern and junction PCR with sequencing verification, yielding stable *Ki*(*trmt61a*^D181A^-EGFP) lines. Primers for junction PCR are listed in Table EV1.

### Generation of *nrf1* crispant using CRISPR/Cas9 ribonucleoprotein complexes

The sgRNA targeting exon 2 of *nrf1* was designed. For microinjection, ribonucleoprotein (RNP) complexes were prepared by incubating the *nrf1* sgRNA, Cas9 protein, and KCl buffer (with concentrations determined using CrispantCal (Burger et al, 2016)) at 37 °C for 5 min (min). In total, 1 nL of the complex solution was injected into 1-cell stage embryo. Mutations were validated by PCR and Sanger sequencing using primers listed in Table EV1.

### Whole mount in situ hybridization (WISH), fluorescence in situ hybridization (FISH) and terminal deoxynucleotidyl transferase dUTP nick end labeling (TUNEL) staining

WISH was conducted according to standard protocols (Zhang et al, 2017). Briefly, DNA templates for antisense probes were cloned into pGEM-T vector. RNA probes were synthesized by in vitro transcription using T7 or SP6 RNA polymerase with digoxigenin (Dig) labeling NTPs mixtures (11277073910, Roche). The fixed and dehydrated zebrafish embryos were hybridized with RNA probes at 65 °C. After hybridization, the embryos were incubated with alkaline phosphatase-conjugated anti-Dig antibody (11093274910, Roche) and stained with BM purple (11442074001, Roche).

For FISH, embryos at 36 hpf were incubated with POD-conjugated anti-Dig antibody (11633716001, Roche) after hybridization with Dig-labeled *kdrl* probe. The TSA Plus Fluorescein Solution (NEL741E001KT, PerkinElmer) was used as substrate. After FISH, TUNEL staining was performed using the In Situ Cell Death Detection Kit (12156792910, TMR red, Roche) according to the manufacturer’s protocol.

### OP-Puro incorporation assay

O-Propargyl-Puromycin (5 µM, HY-15680, MCE) was injected into the yolk sac of Tg(*kdrl*:mCherry) transgenic zebrafish embryos at 35 hpf. After one hour, *kdrl*^+^ cells were isolated using fluorescence-activated cell sorting (FACS). The sorted cells were centrifuged and resuspended in 4% PFA at room temperature. After centrifuged, the pellet was washed and subsequently resuspended with the Click-iT™ Cell Reaction Buffer cocktail (C10269, Invitrogen). Following incubated at room temperature, the stained cell suspension was detected by flow cytometry.

### Quantification of tRNA m^1^A level by UHPLC-MS/MS or LC-MS/MS

Total RNA was extracted from AGM regions of zebrafish embryos using TRIzol Reagent (15596018CN, Invitrogen). Small RNA fractions (<200 nt, enriched for mature tRNA species) or other RNA fractions (≥200 nt) was purified using the MEGAclear™ Kit (AM1908, Invitrogen), following the manufacturer’s protocol as previously described (Liu et al, 2022; Miao et al, 2025). For M*trmt61a*; *trmt61a*^-4bp^ and *trmt61a*^D181A/D181A^, the products were subjected to ultra-high performance liquid chromatography coupled to mass spectrometry (UHPLC-MS/MS) after complete hydrolysis, following previously established method (Yang et al, 2017). Three independent biological replicates were statistically evaluated.

### Mitochondria detection

To assess mitochondria content, the AGM regions from Tg(*fli1a*: EGFP) embryos at 36 hpf were dissociated into single-cell suspensions, and stained with MitoTracker (M22426, Invitrogen) at 28.5 °C for 20 min under light-protected conditions. All samples were analyzed using a BD FACS Canto II (BD Biosciences). The proportion of MitoTracker positive cells and mean fluorescence intensity (MFI) of MitoSOX™ or CellROX™ within the gated *fli1a*^+^ endothelial cells were quantified using Flow Jo v10 software (BD Life Sciences).

### Western blotting

Western blotting was performed according to established protocols (Zhang et al, 2017). The trunk regions of zebrafish embryos were homogenized in lysis buffer, and immunoblotting was performed using the following antibodies: anti-β-Actin antibody (4967S, Cell Signaling Technology, 1:1000), anti-Trmt61a antibody (PA5-88013, Invitrogen, 1:750), anti-Trmt6 antibody (PA5-65616, Invitrogen, 1:750), anti-Flag antibody (F7425, Sigma-Aldrich, 1:1000), anti-Nrf1 antibody (12482-1-AP, Proteintech, 1:1000).

### Immunofluorescence

For whole-mount immunofluorescence, zebrafish embryos at the 1k cell stage were fixed in 4% PFA, permeabilized using 0.3% Triton X-100, and blocked with 1% BSA. Subsequently, the embryos were incubated with anti-Trmt61a antibody (PA5-88013, Invitrogen, 1:50) or anti-m^1^A antibody (D345-3, MBL, 1:50).

Immunofluorescence on transverse sections was performed as previously described (Li et al, 2023). Briefly, zebrafish embryos at 36 hpf were cryoprotected into 30% sucrose, washed with PBS containing 0.1% Tween-20 (PBST), embedded in optimal cutting temperature (OCT, 4583, SAKURA) compound, and sectioned using LEICA CM1900 Cryostats. Sections were mounted onto the slides and incubated with anti-Trmt61a antibody (PA5-88013, Invitrogen, 1:50) or anti-m^1^A antibody (D345-3, MBL,1:50).

### Quantitative real-time PCR (qPCR)

Total RNA was isolated from AGM regions (at 36 hpf), CHT regions (2 dpf or 5 dpf) from zebrafish embryos (*n* = 30), or sorted cell populations at 36 hpf from AGM regions using TRIzol reagent. For tissue samples, first-strand cDNA synthesis was conducted with M-MLV Reverse Transcriptase (M1701, Promega). For sorted cell populations, RevertAid RT Kit (K1691, Thermo Scientific) was employed. Subsequently, qPCR reactions were prepared with SuperReal PreMix Plus (FP 205, TIANGEN) and analyzed on a QuantStudio3 Real-Time PCR System (Thermo Scientific). All experiments were conducted with three independent biological replicates. Primers used for qPCR are listed in Table EV1.

### Confocal microscopy

For in vivo imaging, embryos were immobilized in 1% low-melting-point agarose. For embryos after FISH, and TUNEL, the fish embryos were mounted on microscope slides. Imaging was performed using Andor Dragonfly 505 confocal microscope (Oxford Instruments), and subsequent image analysis was conducted with ImarisViewer (Oxford Instruments) and ImageJ (National Institutes of Health).

### Data-independent acquisition (DIA)-based quantitative proteomics

Endothelial cells (*kdrl*^+^) were isolated from the AGM region of wild-type or *trmt61a* MO-injected embryos at 36 hpf by FACS. Approximately 1 × 10^5^ enriched cells were lysed and low-abundance proteins were selectively enriched with magnetic beads prior to trypsin digestion. Subsequently, peptide separation was performed on a Vanquish Neo UHPLC system (Thermo Scientific), followed by data-independent acquisition (DIA) mass spectrometry analysis using an Orbitrap Astral mass spectrometer (Thermo Scientific). Raw DIA mass spectrometry data were processed with DIA-NN software (v1.8.1) for database searching and protein quantification against the *Danio rerio* UniProtKB reference proteome (taxonomy ID: 7955). Proteins with a fold change >1.5 (up-/downregulated) and *P* value < 0.05 were considered significantly differentially expressed. The complete list of differentially expressed proteins is provided in Dataset EV1.

### RNA-seq and analysis

The *kdrl*^+^ endothelial cells were sorted into cell lysis buffer containing RNase inhibitor (2313 A, Clontech), Triton X-100 solution, dNTP mix, and oligo-dT primer. Reverse transcription and PCR preamplification were performed using KAPA HiFi HotStart Ready MIX (KAPA Biosystems). Following purification with AMPure XP beads (A63882, Beckman), cDNA fragments were captured on C1 beads for library construction using KAPA Hyper Prep Kit (KK8504, Roche). Libraries were ligated with the NEB U-shape adaptor and sequenced on the Illumina Novaseq 6000 platform.

For RNA-seq data analysis, the high-quality reads were aligned to the zebrafish reference genome (version GRCz11) using Hisat2 (version 2.2.1) with default parameters. Reads with a quality score ≥20 and unique mapping were retained for each sample using Samtools (version 1.9). HTSeq (version 0.13.5) was used to quantify the number of aligned reads. Differential gene expression analysis was conducted using DESeq2, with a significance threshold of *P* value < 0.05 and an absolute fold change ≥1.5. Differentially expressed genes in *trmt61a* morphants are listed in Dataset EV2. GO enrichment analysis was conducted using metascape (version 3.5), and visualized in ggplot2. GSEA (version 4.0.3) employed gene sets from the Molecular Signatures Database (MSigDB version 7.5).

### tRNA-seq and analysis

Small RNAs (<200 nt, predominantly mature tRNAs) were extracted from total RNA using the Zymo RNA kit (R1016, ZYMO RESEARCH). 1 µg of small RNA was deacylated by incubation in 0.1 M Tris-HCl (pH 9.0) at 37 °C for 30 min. Half of the deacylated small RNA was then subjected to in vitro demethylation with AlkB (R0639S, Beyotime). The demethylation reaction (20 µL) contained 0.3 µL AlkB enzyme, 2 µL buffer I, and 2 µL buffer II, and was incubated at 37 °C for 2 h. After extraction and ethanol precipitation, both demethylated and untreated RNA samples were then processed for library preparation.

For library construction, RNA was first dephosphorylated using T4 PNK (M0201S, NEB) at 37 °C for 1 h. The 3’ RNA adapter (5’-rAPP-AGATCGGAAGAGCGTCGTG-3’-SpC3) was ligated to the RNA using T4 RNA ligase 1 (M0204L, NEB). After removing the excess adapters, the RNA was purified using MyOne Silane Beads (37002D, Invitrogen). Then, the purified RNA (10 µL) was mixed with 10 pmol RT primer (ACACGACGCTCTTCCGATCT), denatured at 80 °C for 2 min, and immediately chilled on ice. Reverse transcription was performed at 55 °C for 2 h in a reaction buffer containing Induro RTase (M0681S, NEB), 1× RT buffer, 3 mM MgCl₂, 1 mM dNTPs, and 1 U/µL RNase inhibitor. Subsequently, the resulting cDNA was purified using MyOne Silane Beads. The 5’ adapter (5’-Phos-NNNNNNNNNAGATCGGAAGAGCACACGTCTG-3’-SpC3) was ligated to the cDNA 3’ end using T4 RNA ligase1, followed by purification with MyOne Silane Beads. Libraries were amplified by PCR using indexed primers, and purified on an 8% TBE gel. Sequencing was performed on the Novaseq 6000 platform (PE 150).

For tRNA-seq analysis, raw sequencing reads were quality-controlled and preprocessed using fastp (v0.24.0) with the following parameters: -U --umi_loc=read2 --umi_len=10 --umi_prefix=UMI --merge. Clean reads were then reverse-complemented and mapped to the Danio rerio mature tRNA annotations from GtRNAdb using bwa-mem with default parameters. Duplicate reads were removed using UMI-tools with default settings. Subsequently, the BAM files were converted to mpileup format using samtools mpileup, and mutations were identified using awk code, excluding insertions and deletions. m^1^A sites were defined as A bases located at positions 55–61, with mutation rates from at least 30% reduced to zero following AlkB treatment. Finally, all tRNA isoforms corresponding to the same codon were merged for downstream analysis.

### Ribosome profiling and analysis

Ribo-seq libraries were prepared as previously described with minor modification (Ferguson et al, 2023; Reid et al, 2015). For library construction, 250 µL of lysis buffer was added to the sample. After incubation on ice and centrifugation, the supernatant was collected and Bis-Tris buffer (pH 6.0) was added to acidify the lysis solution. Then, nuclease P1 was added to the lysate, followed by incubation at 37 °C for 100 min. RNA was then extracted by adding two volumes of TRIzol™ LS (10296010CN, Thermo Fisher Scientific), and small RNA fragments were separated. Libraries were prepared using the Vazyme small RNA library preparation kit (NR801, VAZYME) for experimental samples and the RNA-Seq kit (12309ES24, YEASEN) for input samples, following the manufacturer’s protocol.

For RNA-seq analysis, raw sequencing reads were quality-controlled and preprocessed using fastp (v0.24.0) with default parameters. The clean reads were then aligned to the reference genome (GRCz11) using STAR (v2.7.11b) with the default parameters. Gene expression quantification was performed using featureCounts (v2.0.3) with the parameters: -p --countReadPairs -s 2 -t exon. For Ribo-seq analysis, raw sequencing reads were quality-controlled, adapter-trimmed, and merged into single-end sequences using fastp (v0.24.0) with the parameters --length_required 20 --merge. The clean reads were first mapped to tRNA and rRNA sequence downloaded from GtRNAdb and NCBI, respectively. Unmapped reads were then aligned to the reference genome (GRCz11) using STAR (v2.7.11b) with the parameters: --outFilterMultimapNmax 1 --outSAMattributes NH HI AS nM --outSJfilterReads Unique. Ribosome-protected fragments (RPF) were quantified using featureCounts (v2.0.3), and differential translation efficiency analysis was conducted using xtail (v1.1.5) with default parameters. Genes with a *P* value < 0.05 and an absolute fold change ≥1.5 were considered to exhibit significant changes in translation efficiency.

### Polysome profiling and qPCR

Polysome profiling was performed using a modified protocol (Leesch et al, 2023). Briefly, the AGM regions from 200 wild-type or *trmt61a* MO-injected embryos at 36 hpf were incubated in medium containing cycloheximide, followed by homogenization in lysis buffer. The resulting lysates were incubated and centrifuged, after which supernatants were layered onto 10-50% sucrose gradients supplemented with 100 µg/mL cycloheximide and 40 U/mL RNase inhibitor. Following centrifugation, fractions were collected and RNA was extracted using TRIzol reagent. Subsequently, reverse transcription and qPCR analyses were performed to assess translation efficiency of target genes.

### Codon switch assay

For codon switch assay, the coding sequences of wild-type *nrf1* (*nrf1*^WT^) (ENSDART00000138183.2) or modified *nrf1*(*nrf1*^MUT^) were cloned into the pDestTol2pA2 plasmid containing the endothelial-specific *fli1a* promoter and tdTomato. Constructs (40 pg per embryo) were then co-injected with *tol2* mRNA (35 pg per embryo) into embryos at 1-cell stage.

### Statistical analysis

Quantification of WISH results was conducted using ImageJ software, based on established protocols (Dobrzycki et al, 2020). Statistical analysis was executed using GraphPad Prism 8.0. Quantitative data were presented as the mean ± standard deviation (SD) from multiple biological replicates. Statistical significance was assessed using two-tailed unpaired Student’s *t* test.

## Supplementary information

Appendix

Table EV1

Peer Review File

Dataset EV1

Dataset EV2

Source data Fig. 1

Source data Fig. 2

Source data Fig. 3

Source data Fig. 4

Source data Fig. 5

Appendix Figure1-2 Source Data

Appendix Figure 3-4 Source Data

Appendix Figure 5-7 Source Data

## Data Availability

The tRNA-seq, Ribosome profiling, Proteomics, and RNA-seq sequencing data supporting the conclusions of this article have been deposited in the Genome Sequence Archive database (GSA: CRA028758). All scripts for tRNA-m^1^A-seq and Ribo-seq data analysis and visualization are available at: https://github.com/jiangliufengzi/tRNA-m1A-seq-and-Ribo-seq-data-analysis. The source data of this paper are collected in the following database record: biostudies:S-SCDT-10_1038-S44319-026-00805-5.
