# Supplementary material for: tRNA m1A modification ensures HSPC production via modulating Nrf1 translation in zebrafish
Source: EMBO Rep. 2026 May 27;27(13):3826–41. doi: 10.1038/s44319-026-00805-5 (PMC13354807; doi:10.1038/s44319-026-00805-5)
Supplement: Supplementary file 1 — Appendix [file 44319_2026_805_MOESM1_ESM.pdf]

Appendix

tRNA m^{1}A modification ensures HSPC production via modulating Nrf1 translation in zebrafish

Zhenkun Dong[1,8], Panfeng Li[2,8], Mengyao Liu[1], Sifeng Wang[3], Weiyi Lai[4], Yining Liu[5], Hailin Wang[4], Ang Li[2], Lu Wang[1,6,7,*]

[*]Correspondence: wanglu1@ihcams.ac.cn (L.W.)

[8] These authors contributed equally to this work.

List of Appendix Figures:

Appendix Fig. S1………………..page 2
Appendix Fig. S2………………..page 4
Appendix Fig. S3………………..page 6
Appendix Fig. S4………………..page 8
Appendix Fig. S5………………..page 10
Appendix Fig. S6………………..page 12
Appendix Fig. S7………………..page 14
Appendix Fig. S8………………..page 16

Appendix Fig. S1

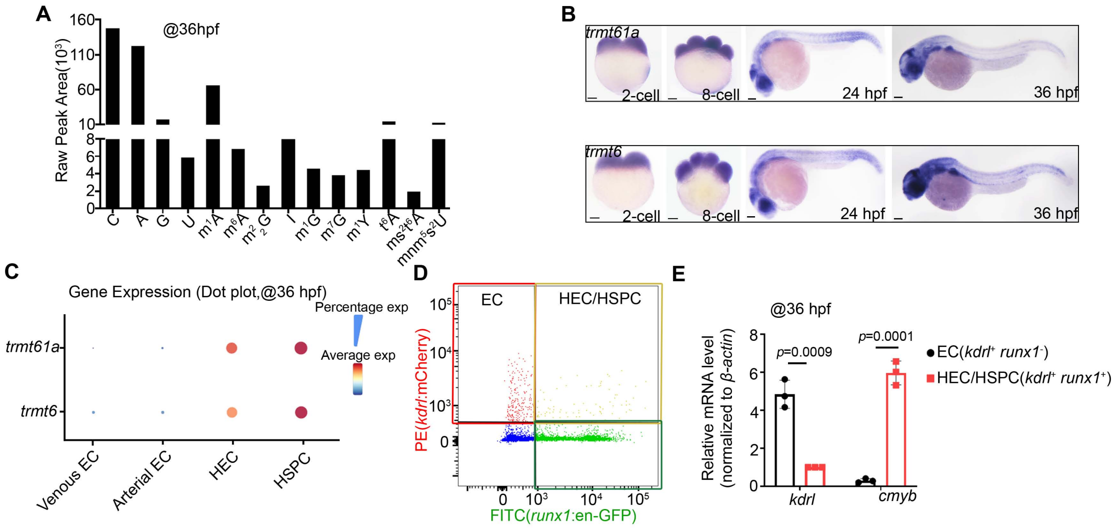

Appendix Fig. S1. The expression of *trmt61a* and *trmt6* is at a relatively high level in HECs and HSPCs.

(A) Quantification of different tRNA modifications in trunk region of WT embryos at 36 hpf by UHPLC-MS/MS (n=30 embryos).

(B) WISH showing the expression of *trmt61a* and *trmt6* from 2-cell stage to 36 hpf. Scale bars: 100 μm. (three biological replicates, one representative image is shown.).

(C) Analysis of published single cell transcriptomics (scRNA-seq) from Tg(*kdrl*:mCherry;*cmyb*:EGFP) zebrafish at 36 hpf (Xia et al., 2023; data ref: Xia et al, 2023) showing the high expression level of *trmt61a* and *trmt6* in HEC and HSPC.

(D) The strategy of flow cytometric sorting of EC (*kdrl*$^{+}$*runx1*$^{-}$), HEC/HSPC (*kdrl*$^{+}$*runx1*$^{+}$), and HSPC (*kdrl*$^{-}$*runx1*$^{+}$) from the AGM region of Tg(*kdrl*:mCherry;*runx1*:en-GFP) embryos at 36 hpf. One representative sample of three biological replicates is shown.

(E) qPCR analysis showing the relative mRNA expression of *kdrl* and *cmyb* in *kdrl*$^{+}$*runx1*$^{-}$ EC, *kdrl*$^{+}$*runx1*$^{+}$ HEC/HSPC at 36 hpf. n=3 biological replicates. Error bars represent mean ± SD. Two-tailed unpaired Student's *t*-test.

Appendix Fig. S2

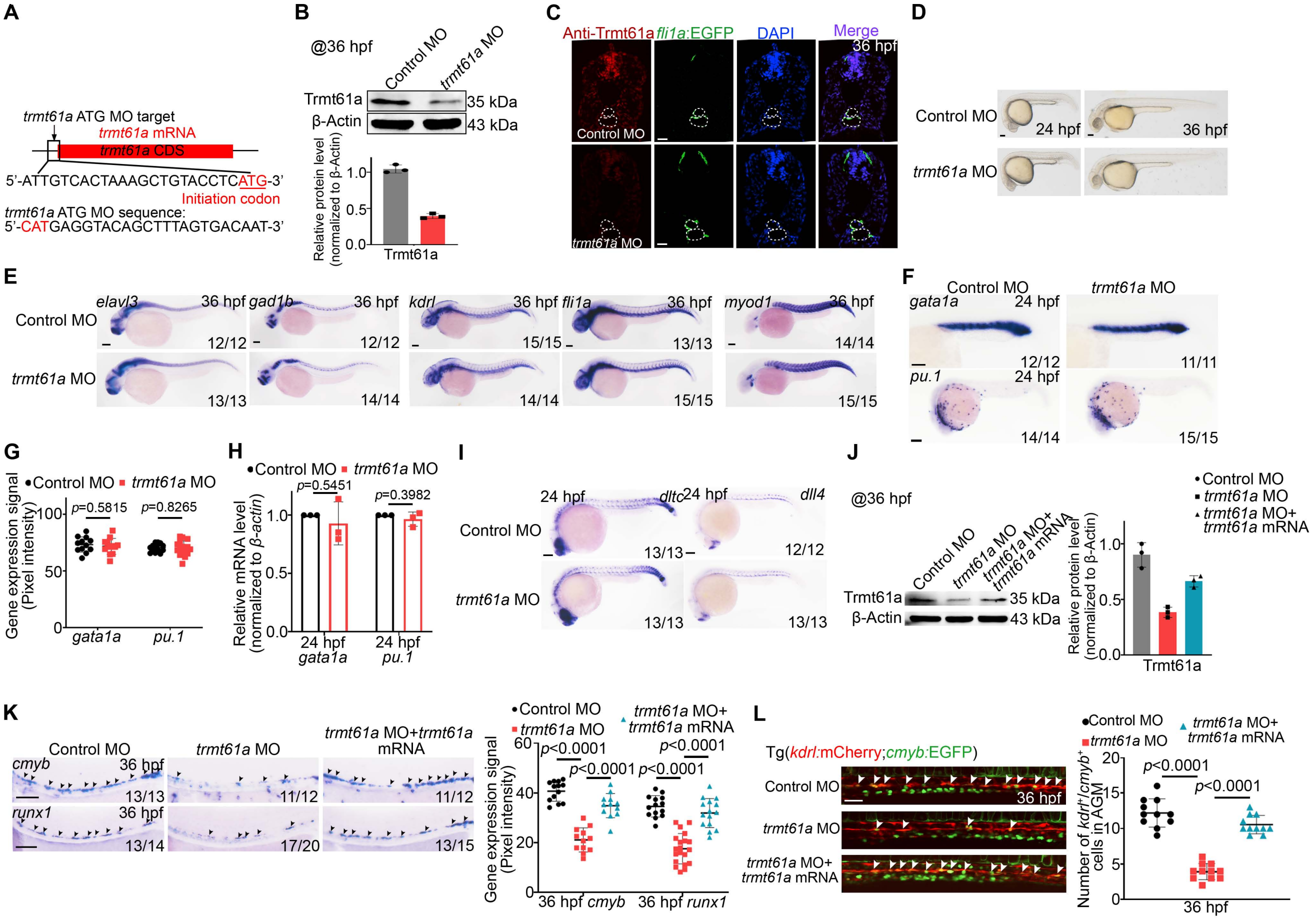

Appendix Fig. S2. *trmt61a* is required for HSPC development.

(A) Schematic diagram of *trmt61a* ATG morpholino blocking translation.

(B) The protein level of Trmt61a (upper) and quantification (lower, three technical replicates) in control MO- and *trmt61a* MO-injected embryos at 36 hpf (n=30 embryos). Error bars represent mean ± SD.

(C) Immunofluorescence on transverse sections showing decreased Trmt61a levels in the AGM region of *trmt61a* morphants at 36 hpf. The white dashed line outlines the dorsal aorta and vein (three biological replicates). One representative image of three biological replicates is shown.

(D) Morphology of control morphants and *trmt61a* morphants at 24 and 36 hpf. One representative image of three biological replicates is shown.

(E) WISH showing the expression of *elval3*, *gad1b*, *kdrl*, *fli1a*, and *myod1* in control MO- and *trmt61a* MO-injected embryos at 36 hpf (three biological replicates).

(F-G) WISH results **(F)** and quantification **(G)** showing the expression of *gata1a* and *pu.1* in control MO- and *trmt61a* MO-injected embryos at 24 hpf (three biological replicates).

(H) qPCR analysis showing the relative mRNA expression of *gata1a* and *pu.1* in control MO- and *trmt61a* MO-injected embryos at 24hpf (three biological replicates).

(I) WISH showing the expression of *dltc* and *dll4* in control MO- and *trmt61a* MO-injected embryos at 24 hpf (three biological replicates).

(J) The protein level of Trmt61a (left) and quantification (right, three technical replicates) in control MO- and *trmt61a* MO-injected embryos and *trmt61a* mRNA injected *trmt61a* morphants at 36 hpf (n=30 embryos). Error bars represent mean ± SD.

(K) WISH results (left) and quantification (right) showing the expression of *cmyb* and *runx1* in control MO-, *trmt61a* MO-, and *trmt61a* MO+*trmt61a* mRNA-injected embryos at 36 hpf (three biological replicates).

(L) Confocal imaging (left) and statistical data (right) showing HECs and emerging HSPCs (white arrowheads) in the AGM region at 36 hpf in control MO-, *trmt61a* MO-, and *trmt61a* MO+*trmt61a* mRNA-injected embryos under Tg(*kdrl*:mCherry;*cmyb*:EGFP) background (three biological replicates).

Error bars represent mean ± SD. Two-tailed unpaired Student's *t*-test (G, H, K, L). The arrowheads indicate the expression of the corresponding markers (K). The numbers indicating the number of embryos with representative phenotype/total number of embryos in each group (E, F, I, K). Scale bars: 100 μm (D, E, F, I, K); 50 μm(L); 20 μm (C).

Appendix Fig. S3

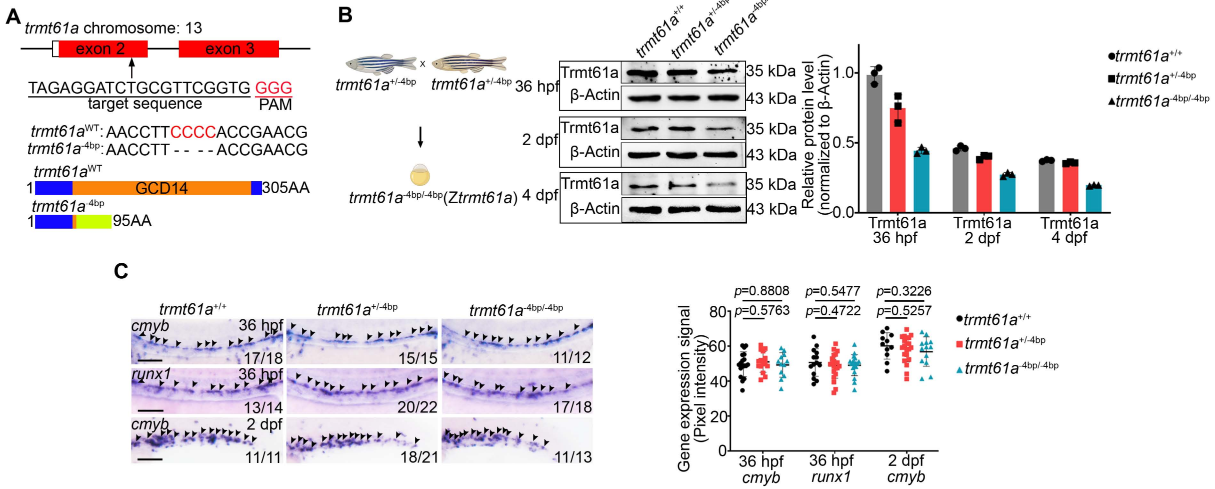

Appendix Fig. S3. Zygotic *trmt61a* mutants display unaltered HSPC generation.

(A) Generation of *trmt61a* mutants via CRISPR/Cas9. The *trmt61a* gRNA targets exon 2 leads to frameshift and premature termination of translation.

(B) Generation of *trmt61a* zygotic mutant (Z*trmt61a*, left), and the protein level of Trmt61a (middle) with quantification (right, three technical replicates) at 36 hpf, 2 dpf and 4 dpf in *trmt61a*$^{+/+}$, *trmt61a*$^{+/-4bp}$ and *trmt61a* $^{-4bp/-4bp}$ embryos (n=30 embryos). Error bars represent mean ± SD.

(C) WISH results (left) and quantification (right) showing the expression of *cmyb* and *runx1* at 36 hpf and 2 dpf in *trmt61a*$^{+/+}$, *trmt61a*$^{+/-4bp}$ and *trmt61a* $^{-4bp/-4bp}$ embryos. Arrowheads denote *runx1* or *cmyb* positive signals. The numbers indicating the number of embryos with representative phenotype/total number of embryos in each group. Scale bars: 100 μm. Error bars represent mean ± SD. Two-tailed unpaired Student's *t*-test.

Appendix Fig. S4

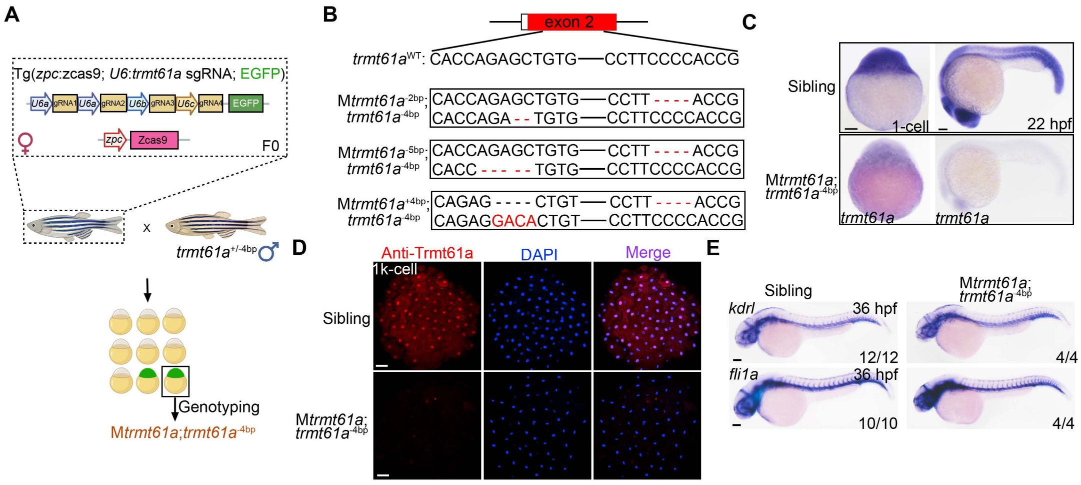

Appendix Fig. S4. Generation of M*trmt61a*;*trmt61a*$^{-4bp}$ mutant.

(A) Pipeline to generate M*trmt61a*;*trmt61a*$^{-4bp}$ embryos.

(B) Different frameshift mutations of M*trmt61a*;*trmt61a*$^{-4bp}$ embryos.

(C) WISH results showing the absent *trmt61a* expression in M*trmt61a*;*trmt61a*$^{-4bp}$ embryos at 1-cell stage and 22 hpf, compared with siblings (three biological replicates). Scale bars: 100 μm

(D) Immunofluorescence showing the maternal Trmt61a protein in siblings and M*trmt61a*;*trmt61a*$^{-4bp}$ embryos at 1k-cell stage (three biological replicates). Scale bars: 20 μm. One representative image of three biological replicates is shown.

(E) WISH showing the expression of *kdrl* and *fli1a* in siblings and M*trmt61a*;*trmt61a*$^{-4bp}$ embryos. The numbers indicating the number of embryos with representative phenotype/total number of embryos in each group (three biological replicates). Scale bars: 100 μm.

Appendix Fig. S5

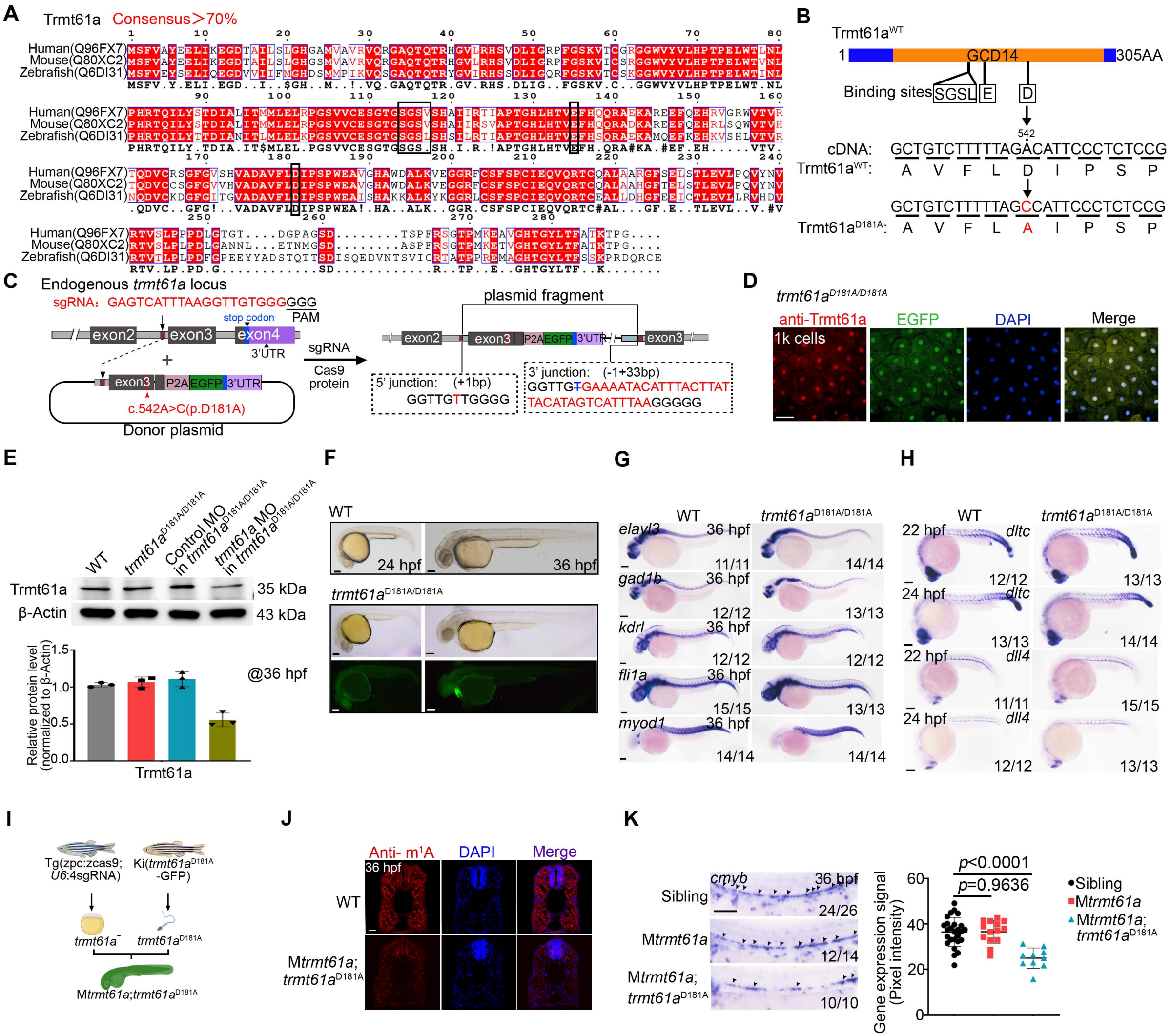

Appendix Fig. S5. Trmt61a regulates HSPC generation via m^{1}A58 modification

(A) Protein sequence alignment using Clustal Omega displaying over 70% sequence of Trmt61a conservation across human, mouse, and zebrafish, with nearly completely conserved binding sites (Black squares).

(B) Schematic representation of conserved binding site of Trmt61a^{WT} and Trmt61a^{D181A}. The bases and corresponding amino acid changes are highlighted in red.

(C) Procedure of the intron targeting-mediated strategy for generating *trmt61a*^{D181A} knockin zebrafish line by CRISPR/Cas9 system and 5' and 3' junction sequences of F1 progenies (right). The donor cassette was integrated into the *trmt61a* locus after co-injection of the donor with the sgRNA and Cas9 protein. The zebrafish *trmt61a* has 4 exons. The arrows in left panel indicate the sgRNA target sites.

(D) Immunostaining of Trmt61a and EGFP in *trmt61a*$^{D181A/D181A}$ knockin embryos. One representative image of three biological replicates is shown.

(E) Western blot analysis (upper) and quantification (lower, three technical replicates) showed the Trmt61a protein level in wild-type embryos, *trmt61a*$^{D181A/D181A}$ embryos, and control MO-, or *trmt61a* MO-injected *trmt61a*$^{D181A/D181A}$ embryos (n=30 embryos). Error bars represent mean ± SD.

(F) Morphology of WT and *trmt61a*$^{D181A/D181A}$ embryos (three biological replicates).

(G) WISH showing the expression of *elval3*, *gad1b*, *kdrl*, *fli1a*, and *myod1* in WT and *trmt61a*$^{D181A/D181A}$ embryos at 36 hpf (three biological replicates).

(H) WISH showing the expression of *dltc* and *dll4* in WT and *trmt61a*$^{D181A/D181A}$ embryos at 22 hpf and 24 hpf (three biological replicates).

(I) Schematic diagram of the M*trmt61a; trmt61a*^{D181A} zebrafish line generation.

(J) Immunofluorescence on transverse sections showing the decreased m^{1}A level in the AGM region in siblings and *trmt61a*$^{D181A/D181A}$ embryos at 36 hpf. The white dashed line outlines the dorsal aorta and vein (three biological replicates).

(K) WISH (left) and quantification (right) at 36 hpf showing the expression of *cmyb* in siblings, M*trmt61a*, and M*trmt61a*;*trmt61a*^{D181A} embryos. Arrowheads denote *cmyb* positive signals (three biological replicates). Error bars represent mean ± SD. Two-tailed unpaired Student's *t*-test.

The numbers indicating the number of embryos with representative phenotype/total number of embryos in each group (G, H, K). Scale bars: 100 μm (F, G, H, K); 20 μm (D, J).

Appendix Fig. S6

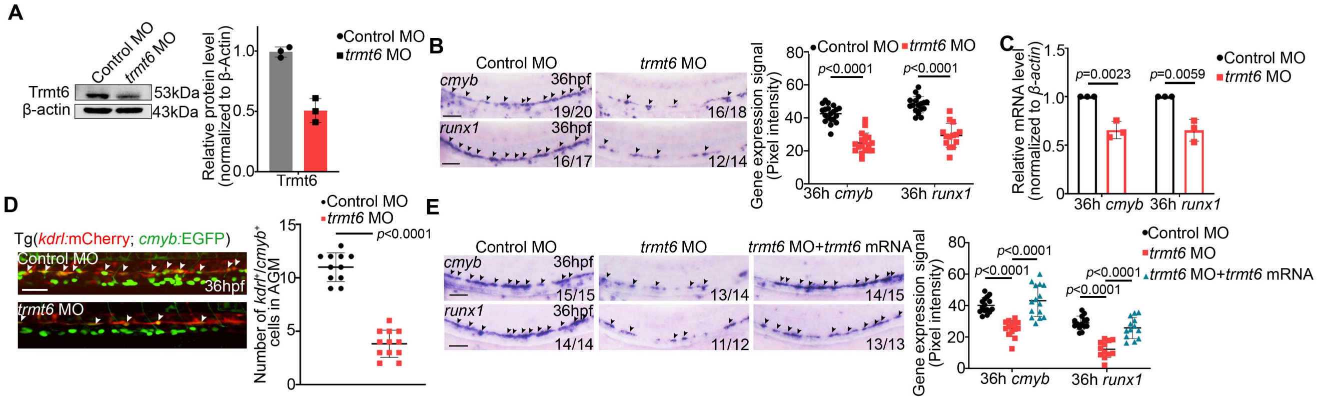

Appendix Fig. S6. *trmt6* deficiency leads to impaired HSPC generation.

(A) The protein level of Trmt6 (left) and quantification (right, three technical replicates) in control MO- and *trmt6a* MO-injected embryos (n=30 embryos). Error bars represent mean ± SD.

(B-C) WISH **(B, left)** with quantification **(B, right)**, and qPCR **(C)** showing the expression of *cmyb* and *runx1* in control MO- and *trmt6a* MO-injected embryos at 36 hpf (three biological replicates).

(D) Confocal imaging (left) and statistical data (right) of Tg(*kdrl*:mCherry;*cmyb*:EGFP) showing the number of HECs and emerging HSPCs (white arrowheads) in control MO- and *trmt6a* MO-injected embryos (three biological replicates).

(E) WISH (left) and quantification (right) showing the expression of *cmyb* and *runx1* at 36 hpf in the AGM region of control MO-, *trmt6* MO- and *trmt6* MO+mRNA-injected embryos (three biological replicates).

Error bars represent mean ± SD. Two-tailed unpaired Student's *t*-test (B, C, D, E). Arrowheads denote *cmyb* and *runx1* positive signals (B, E). The numbers indicating the number of embryos with representative phenotype/total number of embryos in each group (B, E). Scale bars: 100 μm (B, E); 50 μm (D).

Appendix Fig. S7

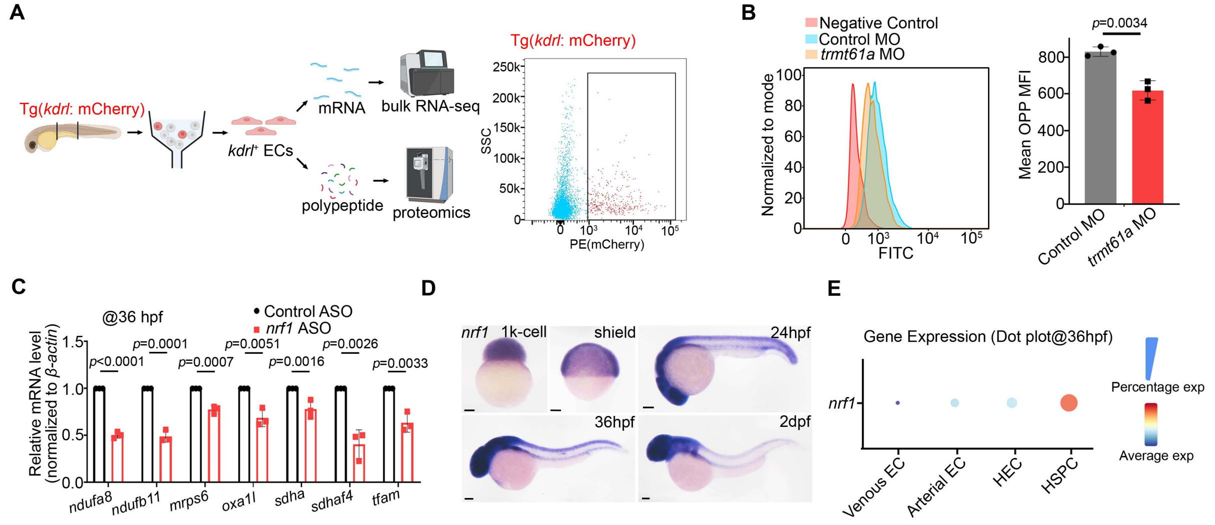

Appendix Fig. S7 *trmt61a* deficiency impairs global translation.

(A) Schematic diagram of RNA sequencing and proteomic analysis of *kdrl*$^+$ ECs sorted from the AGM region in uninjected embryos or *trmt61a* morphants.

(B) O-Propargyl-puromycin (OP-puro) in *kdrl*$^+$ ECs from the AGM region in control MO- and *trmt6a* MO-injected embryos. Error bars represent mean ± SD. Two-tailed unpaired Student's *t*-test. One representative sample of three biological replicates is shown.

(C) qPCR analysis showing the relative expression of mitochondrial related genes in *kdrl*$^+$ ECs from control ASO- and *nrf1* ASO-injected embryos at 36 hpf (three biological replicates). Error bars represent mean ± SD. Two-tailed unpaired Student's t-test.

(D) WISH showing the expression of *nrf1* from 1k-cell stage to 2 dpf. Scale bars: 100 μm. n≥3 biological replicates. One representative sample of three biological replicates is shown.

(E) Analysis of published single cell transcriptomics (scRNA-seq) from Tg(*kdrl*:mCherry; *runx1*:en-GFP) zebrafish at 36 hpf (Xia et al., 2023; data ref: Xia et al, 2023) showing the high expression level of *nrf1* in HEC and HSPC.

Appendix Fig. S8

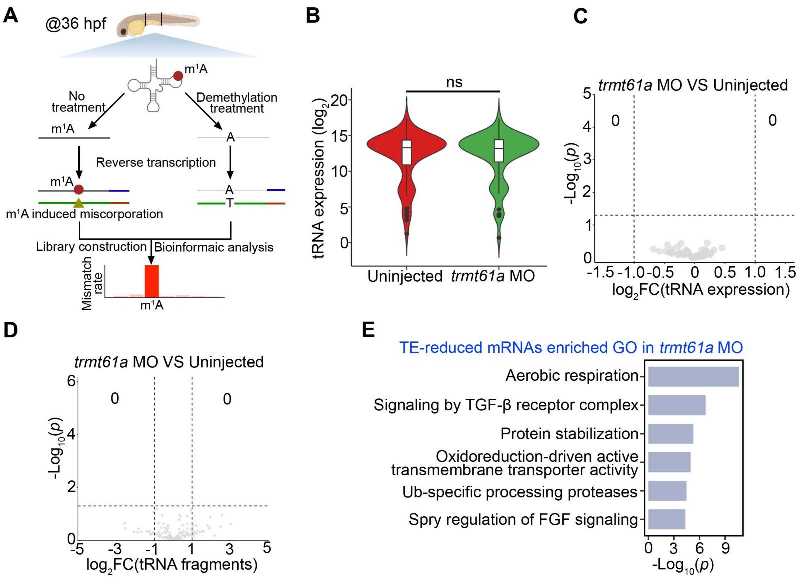

Appendix Fig. S8. Loss of Trmt61a has no effect on tRNA abundance or stability.

(A) Scheme of tRNA seq and tRNA m^1A-seq.

(B) The global tRNA expression in uninjected embryos and *trmt61a* morphants. ns=0.2403.

(C) Differential expression analysis of tRNAs in uninjected embryos and *trmt61a* morphants.

(D) Differential expression analysis of tRNA fragments in uninjected embryos and *trmt61a* morphants.

(E) Representative gene ontology biological process categories enriched in TE-reduced genes in *trmt61a* morphants.

References

Xia, J., Liu, M., Zhu, C., Liu, S., Ai, L., Ma, D., Zhu, P., Wang, L., and Liu, F. (2023). Activation of lineage competence in hemogenic endothelium precedes the formation of hematopoietic stem cell heterogeneity. Cell Res *33*, 448-463. GSE186427 [DATASET]
