# Supplementary figures and images for: tRNA m1A modification ensures HSPC production via modulating Nrf1 translation in zebrafish (part 1 of 3)
Source: EMBO Rep. 2026 May 27;27(13):3826–41. doi: 10.1038/s44319-026-00805-5 (PMC13354807; doi:10.1038/s44319-026-00805-5)

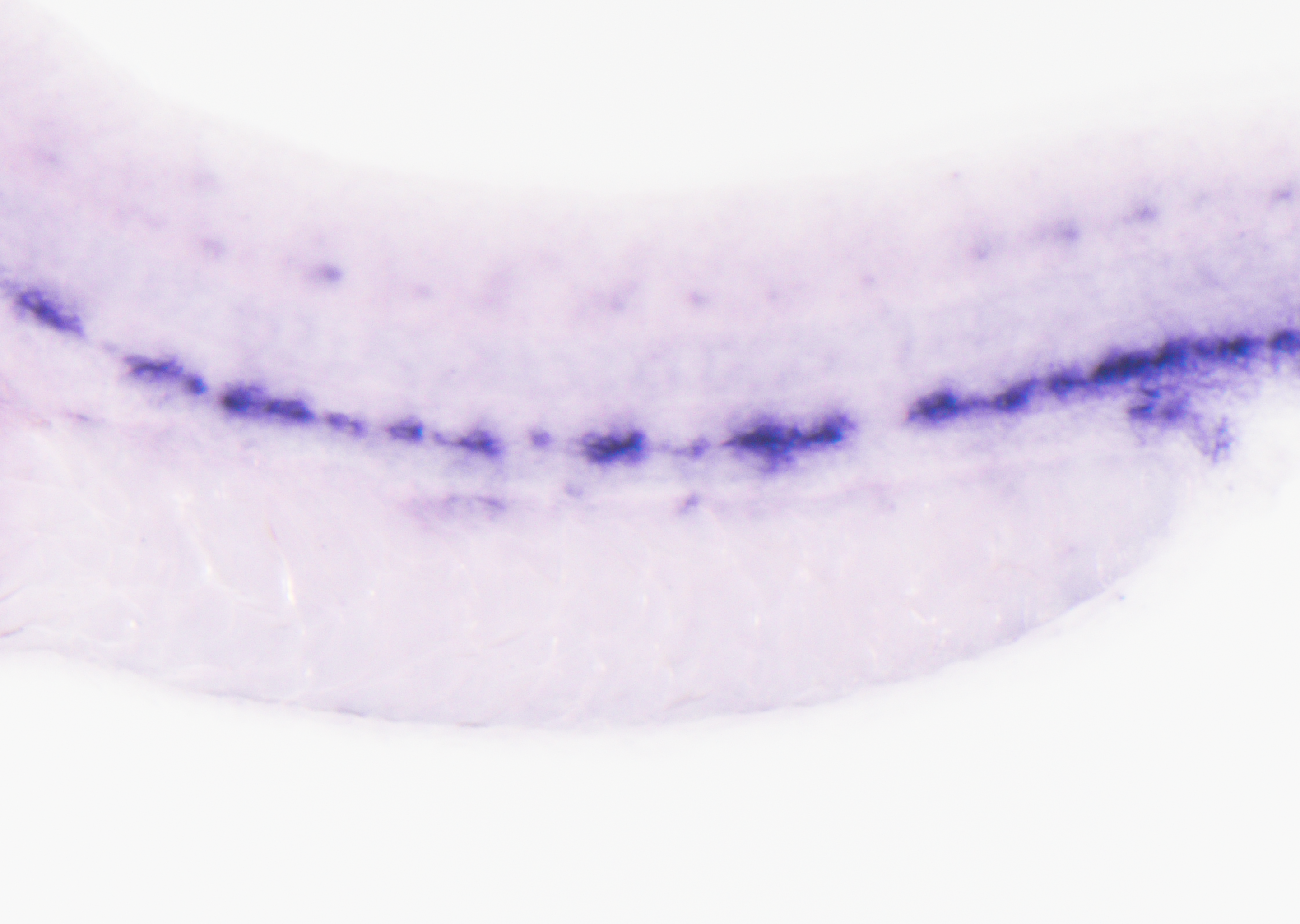

Supplement: Supplementary file 6 — Source data Fig. 1 [file 44319_2026_805_MOESM6_ESM.zip › Source Data Fig.1/Fig.1/B/1. runx1 36hpf controlMO.tif]

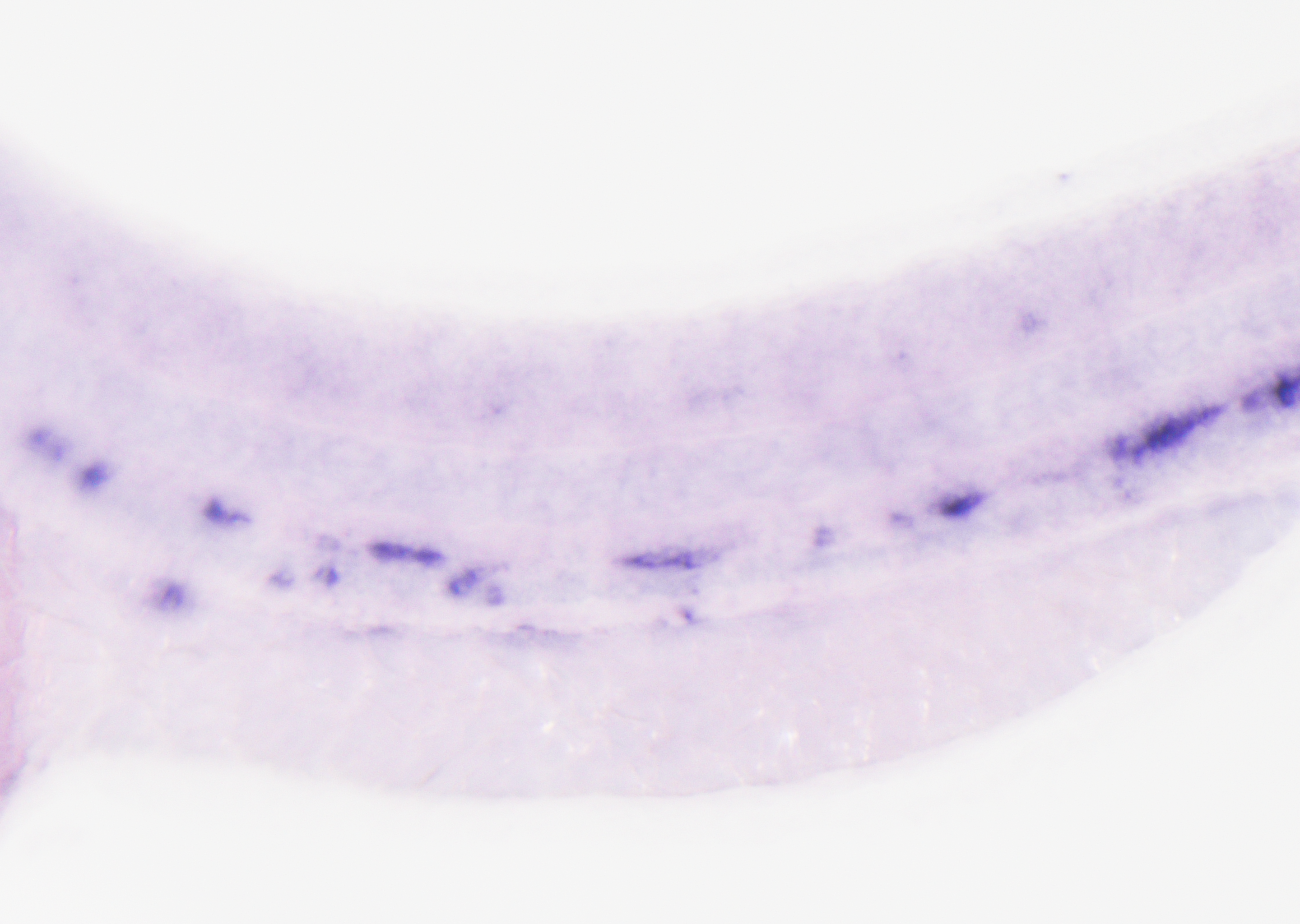

Supplement: Supplementary file 6 — Source data Fig. 1 [file 44319_2026_805_MOESM6_ESM.zip › Source Data Fig.1/Fig.1/B/2. runx1 36hpf trmt61aMO.tif]

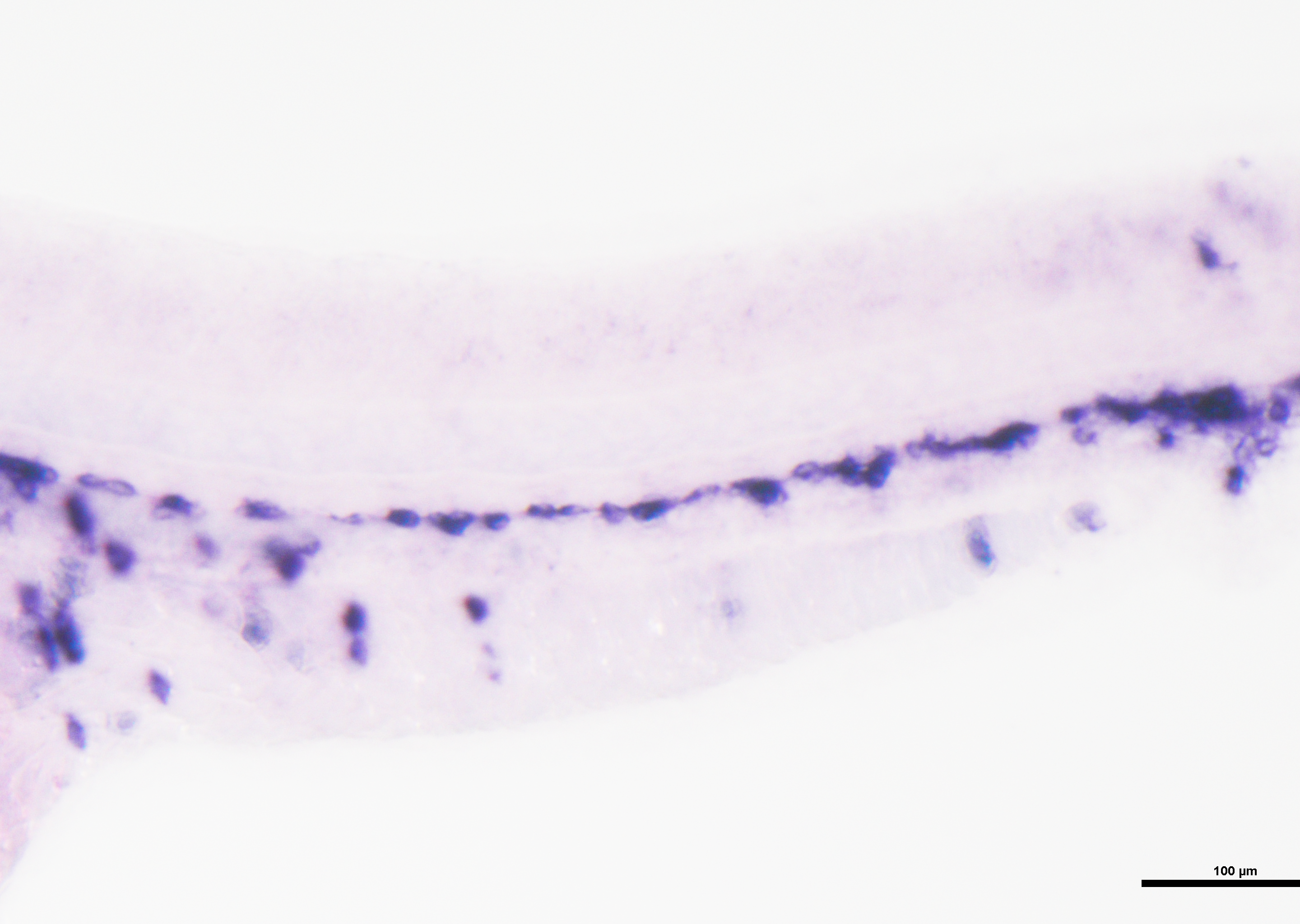

Supplement: Supplementary file 6 — Source data Fig. 1 [file 44319_2026_805_MOESM6_ESM.zip › Source Data Fig.1/Fig.1/B/3. cmyb 36hpf controlMO.tif]

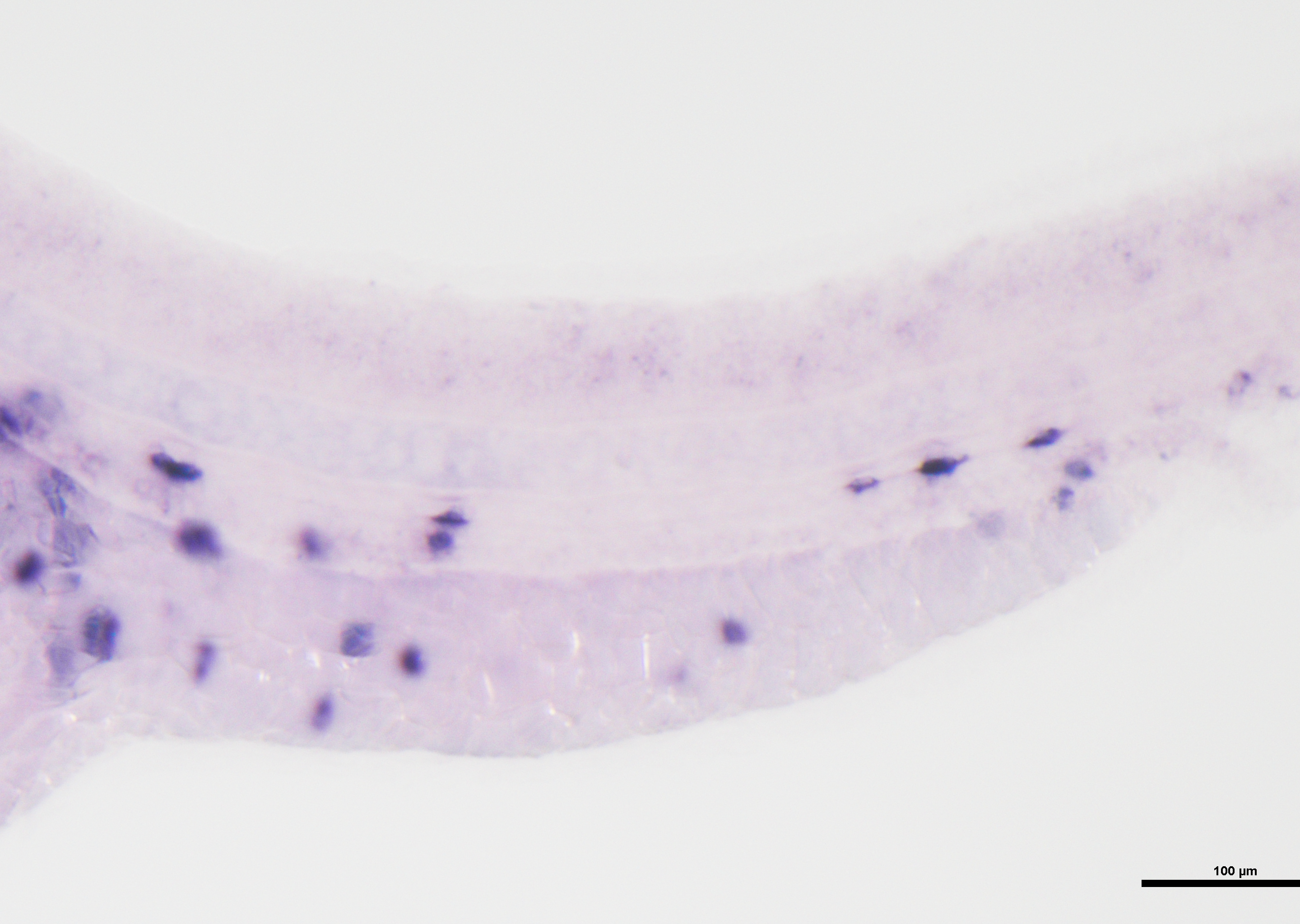

Supplement: Supplementary file 6 — Source data Fig. 1 [file 44319_2026_805_MOESM6_ESM.zip › Source Data Fig.1/Fig.1/B/4. cmyb 36hpf trmt61aMO.tif]

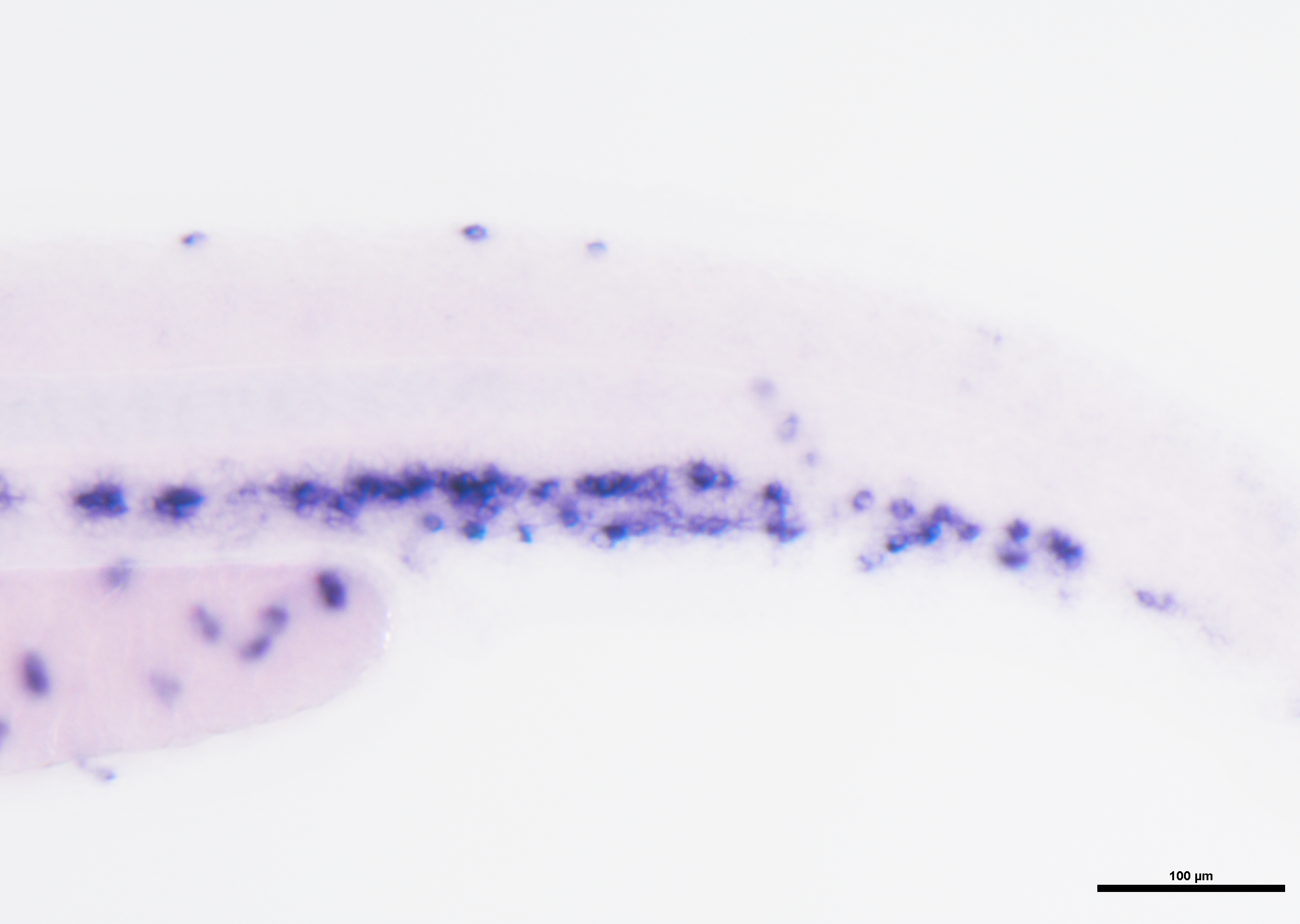

Supplement: Supplementary file 6 — Source data Fig. 1 [file 44319_2026_805_MOESM6_ESM.zip › Source Data Fig.1/Fig.1/B/5. cmyb 2dpf controlMO.tif]

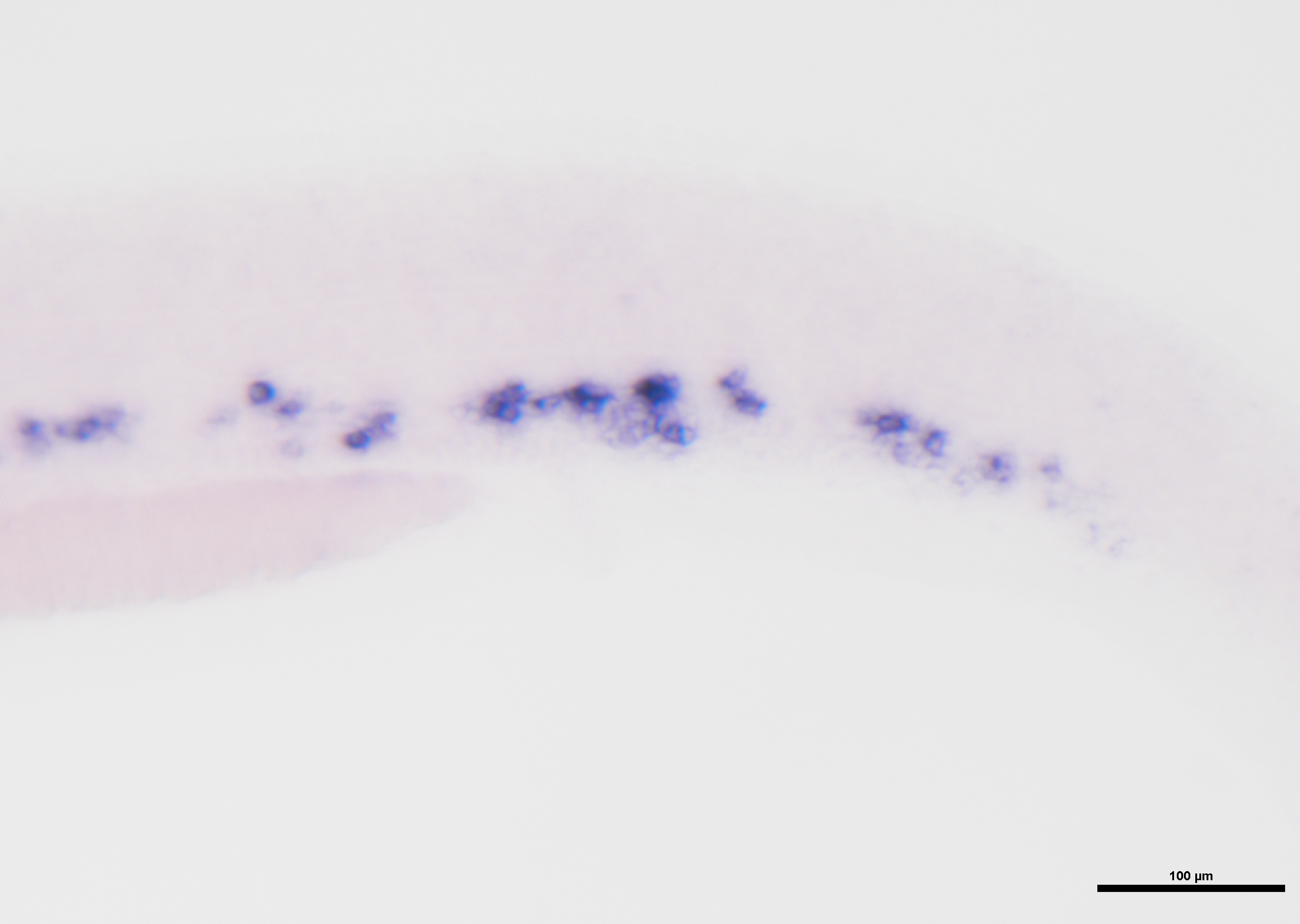

Supplement: Supplementary file 6 — Source data Fig. 1 [file 44319_2026_805_MOESM6_ESM.zip › Source Data Fig.1/Fig.1/B/6. cmyb 2dpf trmt61aMO.tif]

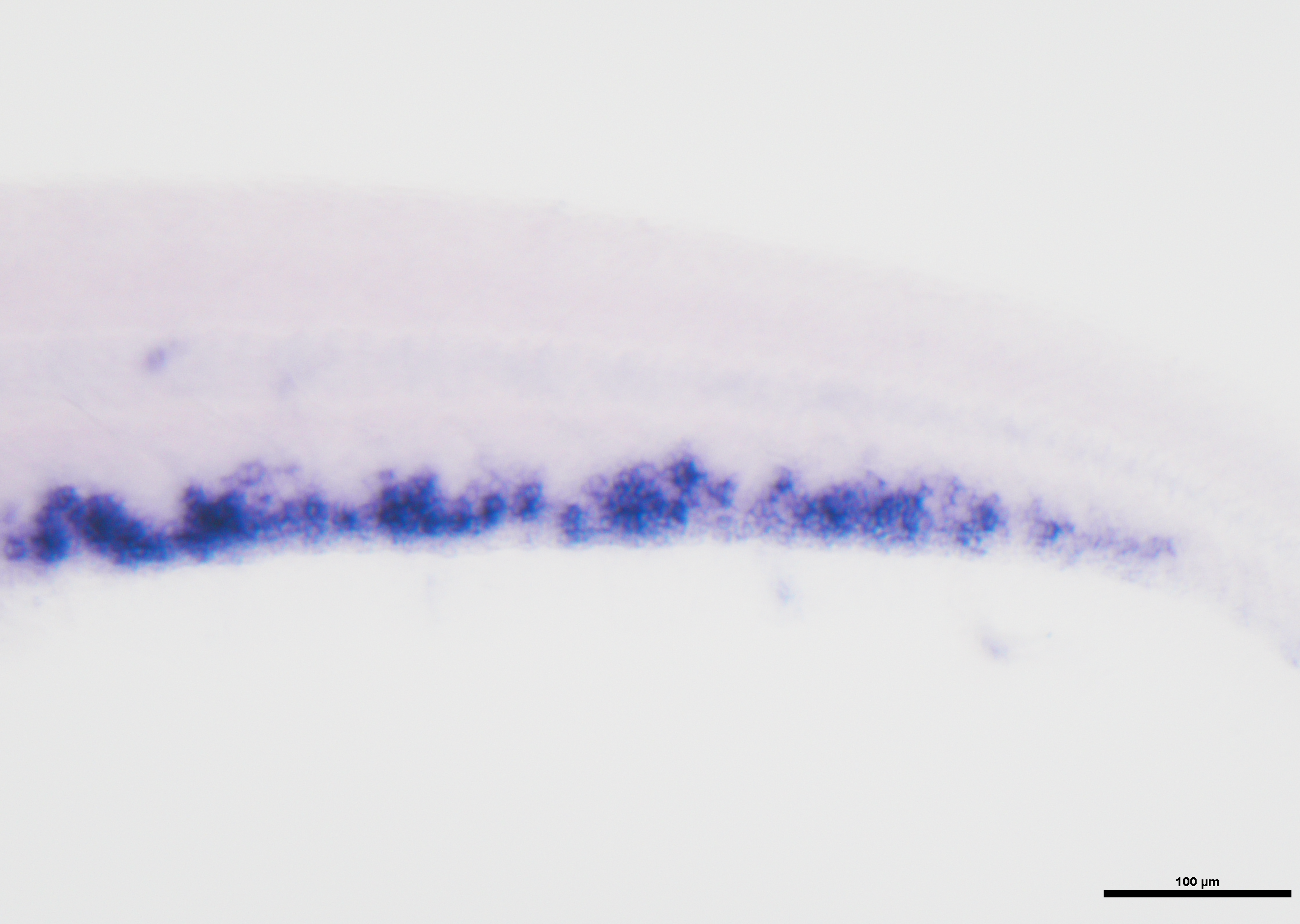

Supplement: Supplementary file 6 — Source data Fig. 1 [file 44319_2026_805_MOESM6_ESM.zip › Source Data Fig.1/Fig.1/B/7. cmyb 5dpf controlMO.tif]

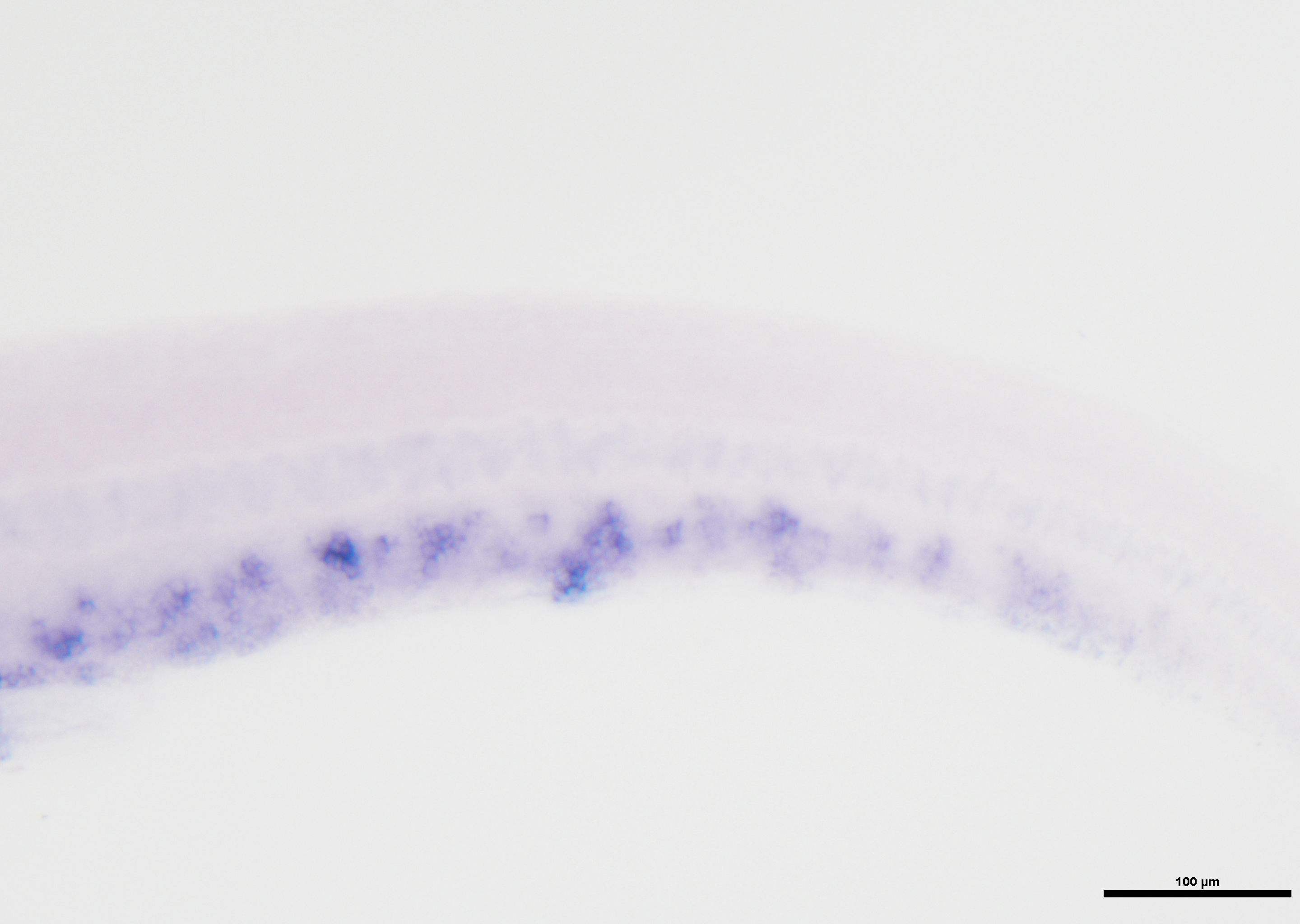

Supplement: Supplementary file 6 — Source data Fig. 1 [file 44319_2026_805_MOESM6_ESM.zip › Source Data Fig.1/Fig.1/B/8. cmyb 5dpf trmt61aMO.tif]

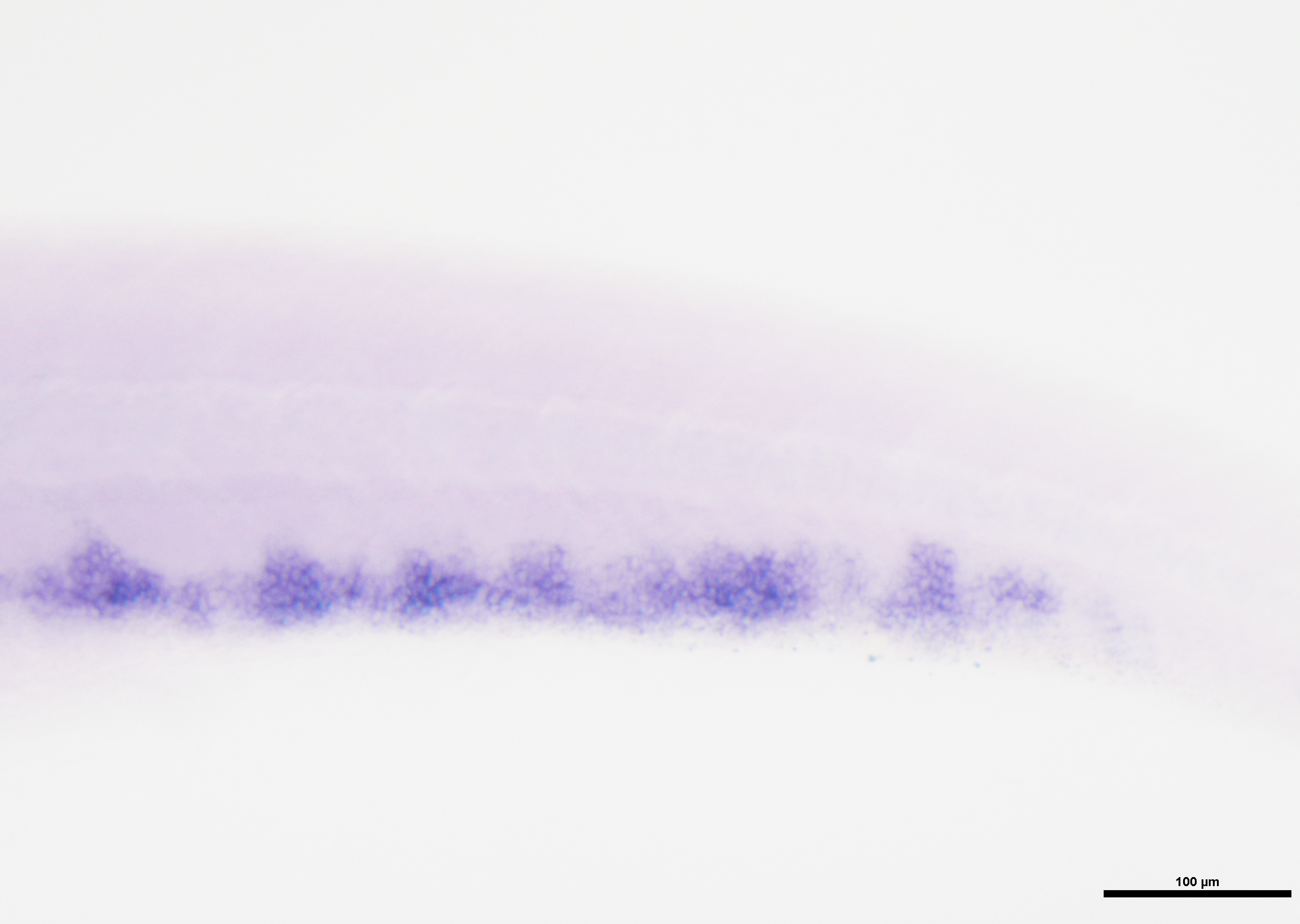

Supplement: Supplementary file 6 — Source data Fig. 1 [file 44319_2026_805_MOESM6_ESM.zip › Source Data Fig.1/Fig.1/D/1. gata1a 5dpf controlMO.tif]

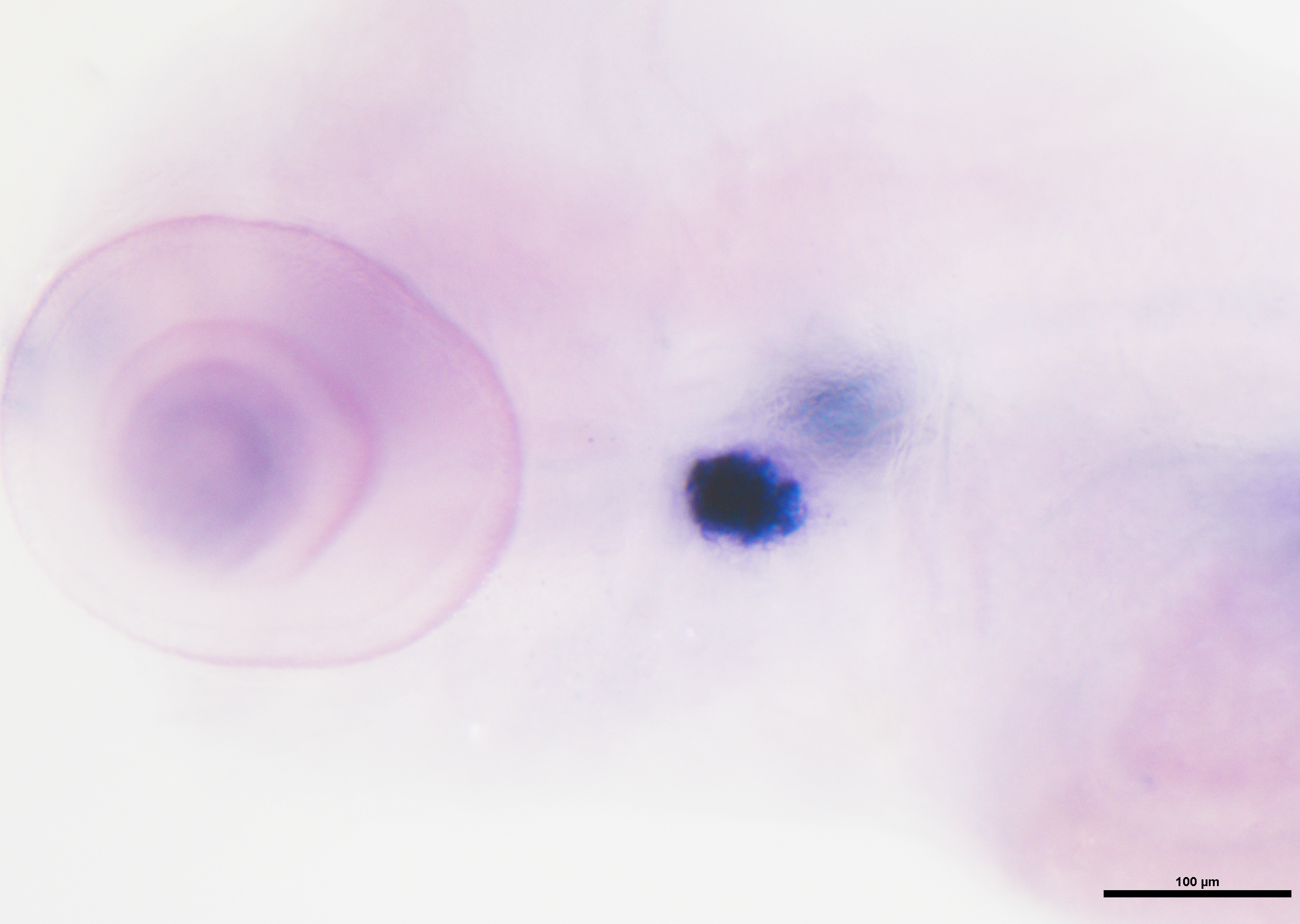

Supplement: Supplementary file 6 — Source data Fig. 1 [file 44319_2026_805_MOESM6_ESM.zip › Source Data Fig.1/Fig.1/D/10. rag1 5dpf controlMO.tif]

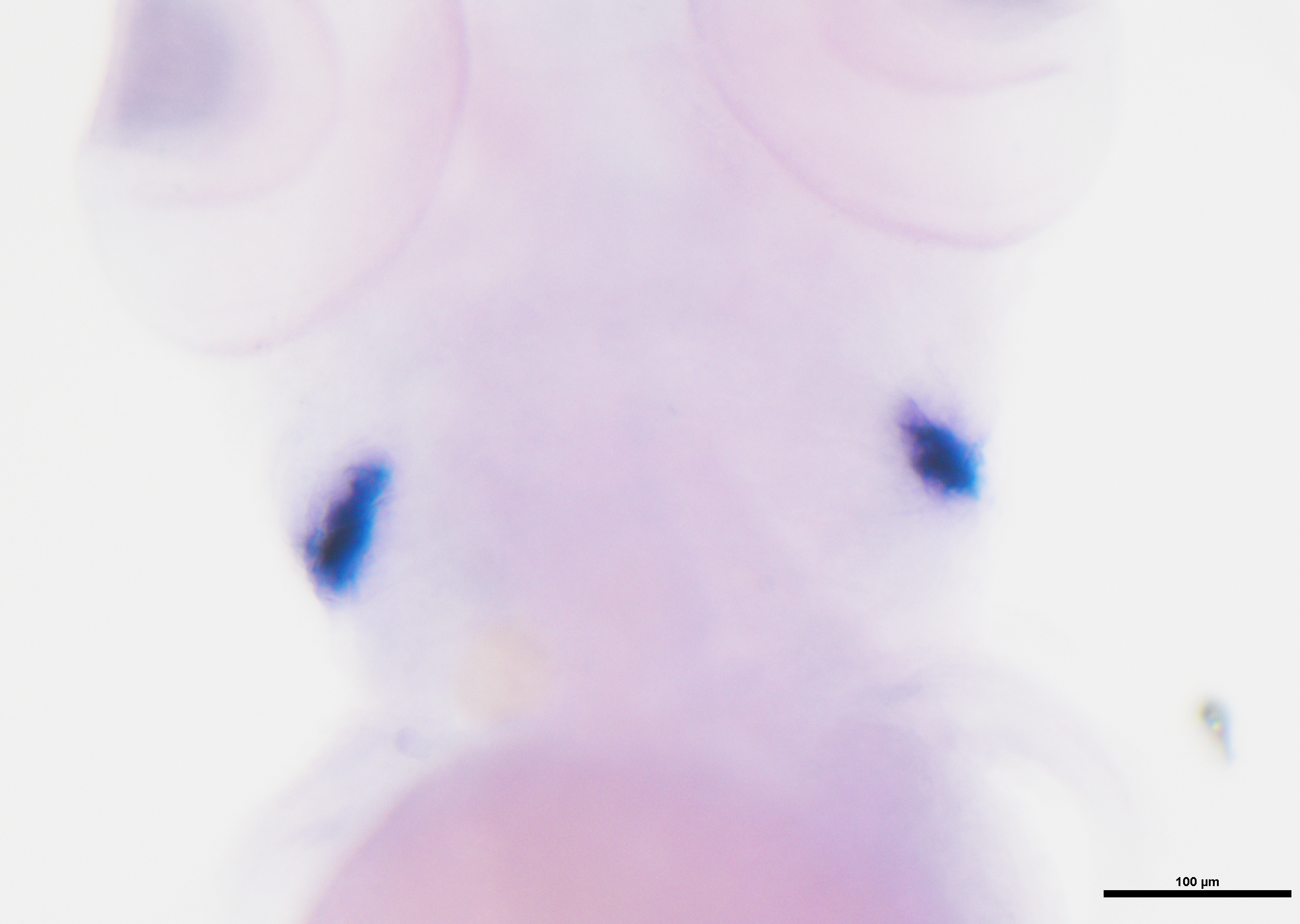

Supplement: Supplementary file 6 — Source data Fig. 1 [file 44319_2026_805_MOESM6_ESM.zip › Source Data Fig.1/Fig.1/D/11. rag1 5dpf trmt61aMO.tif]

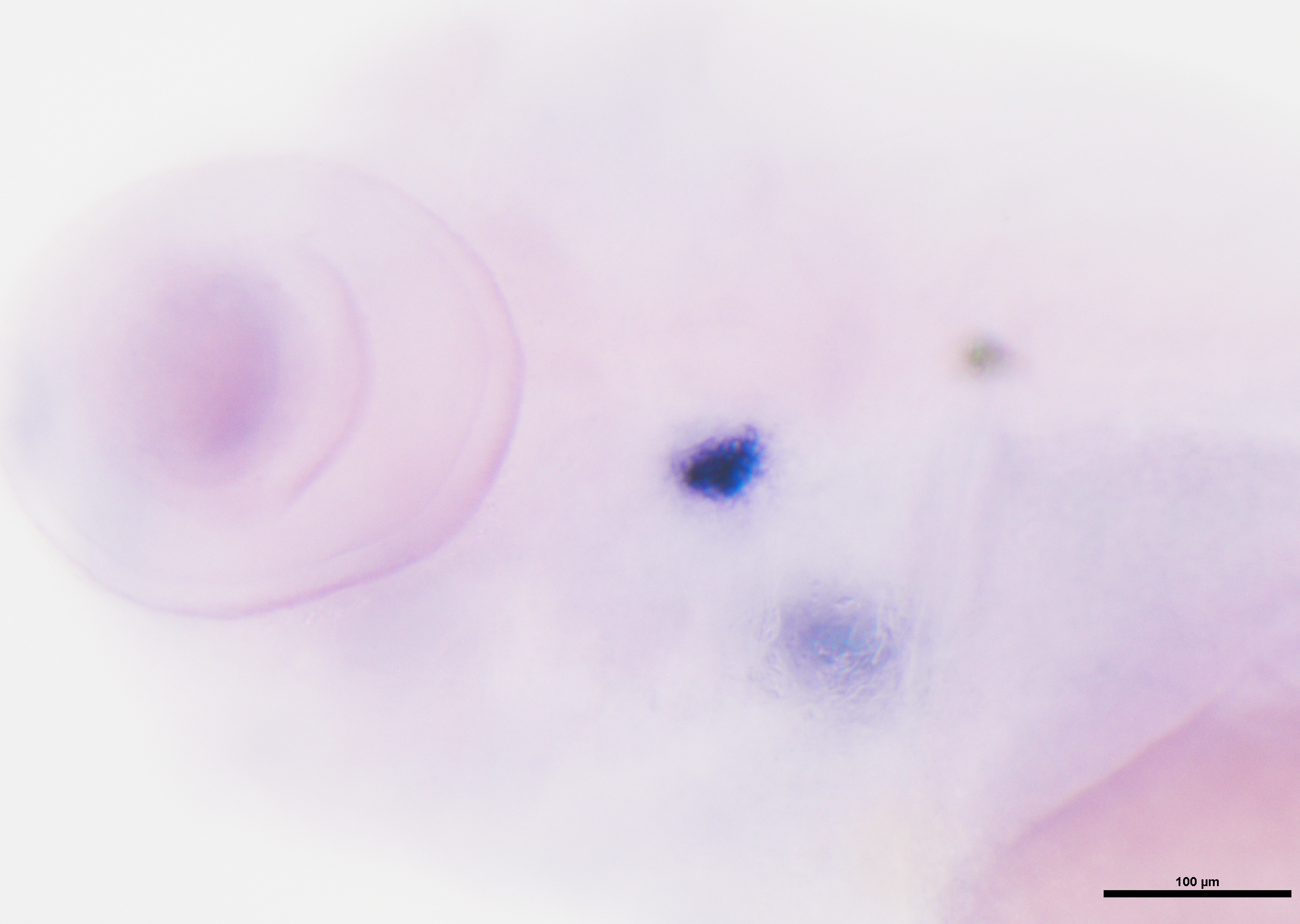

Supplement: Supplementary file 6 — Source data Fig. 1 [file 44319_2026_805_MOESM6_ESM.zip › Source Data Fig.1/Fig.1/D/12. rag1 5dpf trmt61aMO.tif]

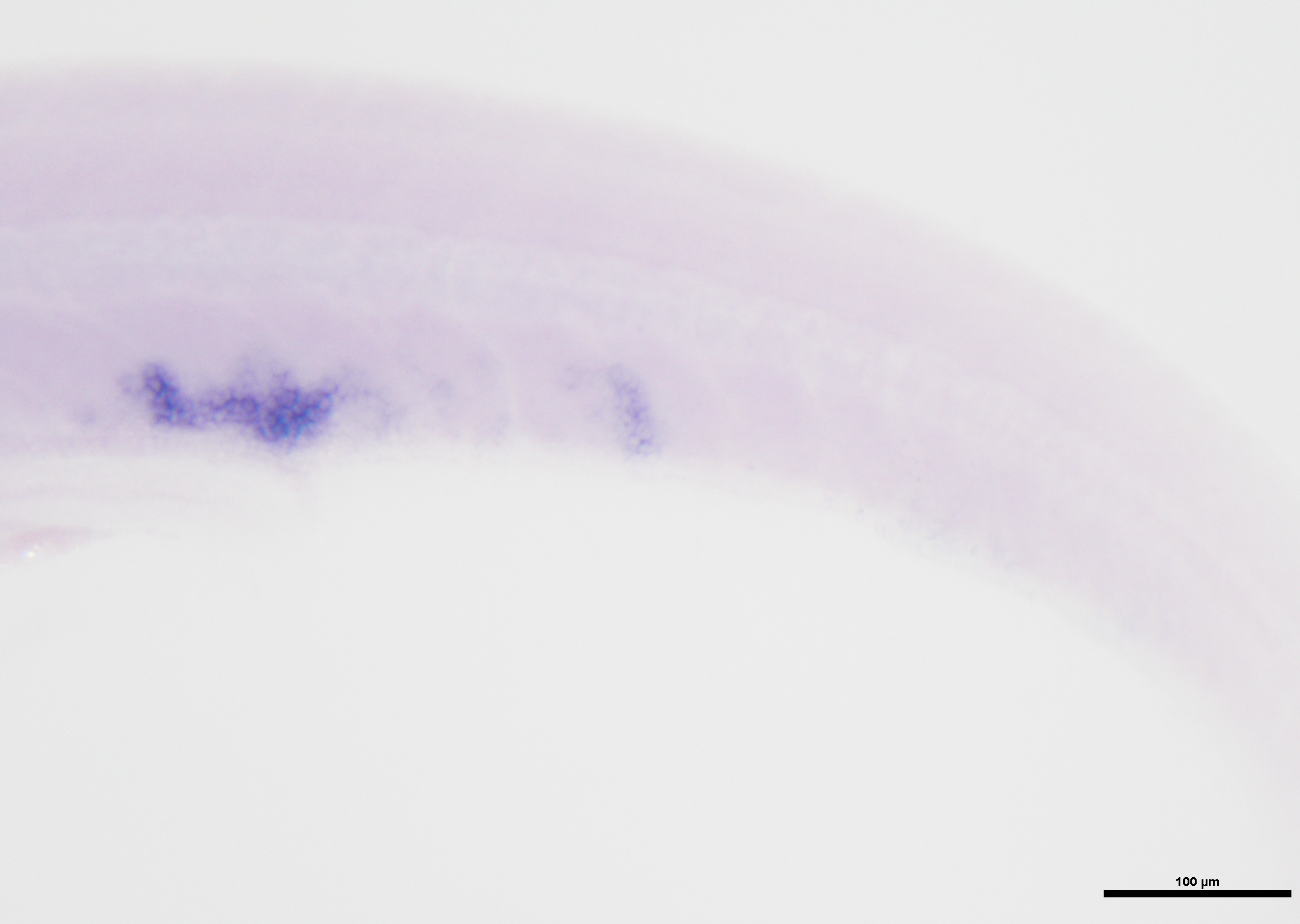

Supplement: Supplementary file 6 — Source data Fig. 1 [file 44319_2026_805_MOESM6_ESM.zip › Source Data Fig.1/Fig.1/D/2. gata1a 5dpf trmt61aMO.tif]

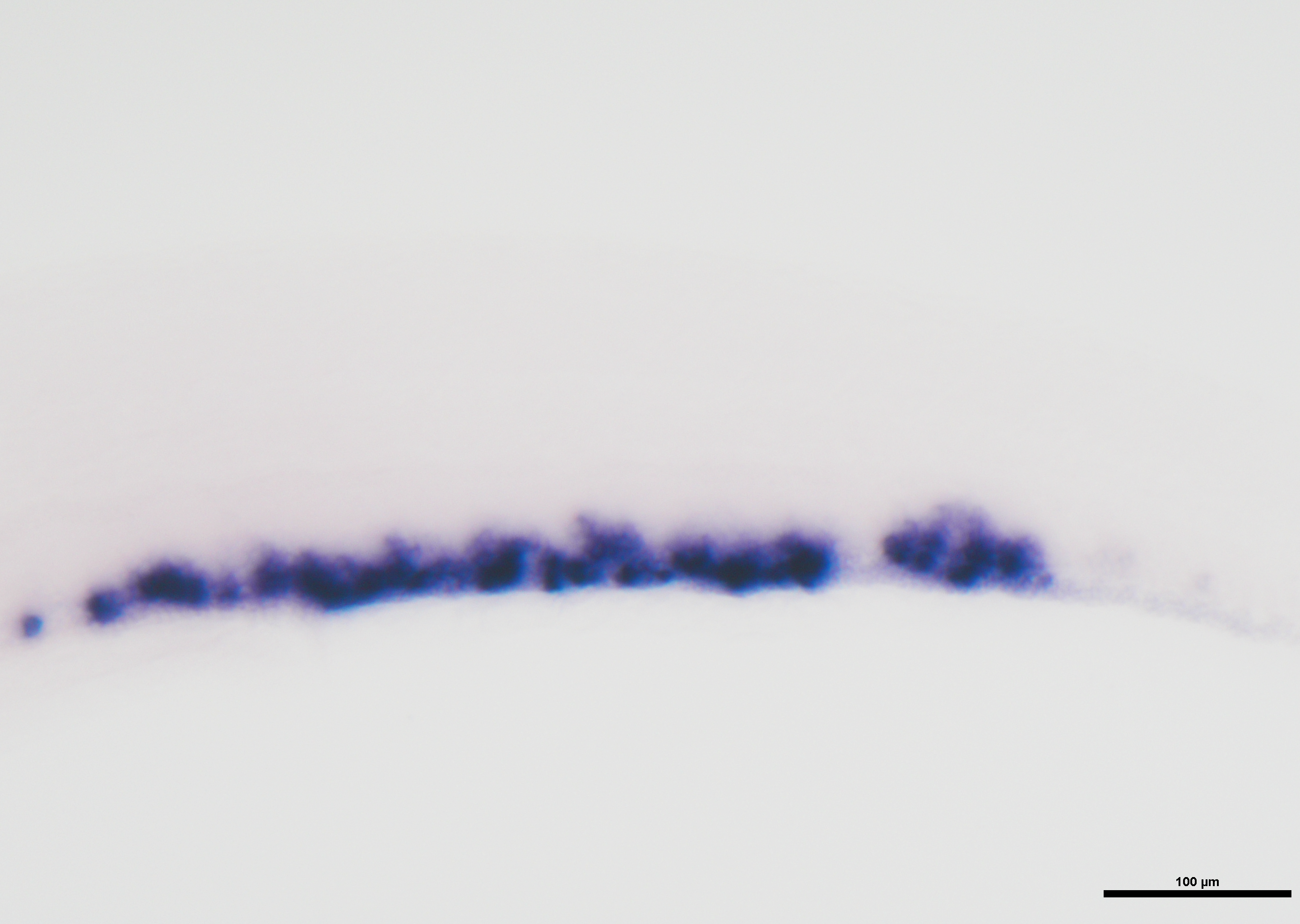

Supplement: Supplementary file 6 — Source data Fig. 1 [file 44319_2026_805_MOESM6_ESM.zip › Source Data Fig.1/Fig.1/D/3. hbae1.1 5dpf controlMO.tif]

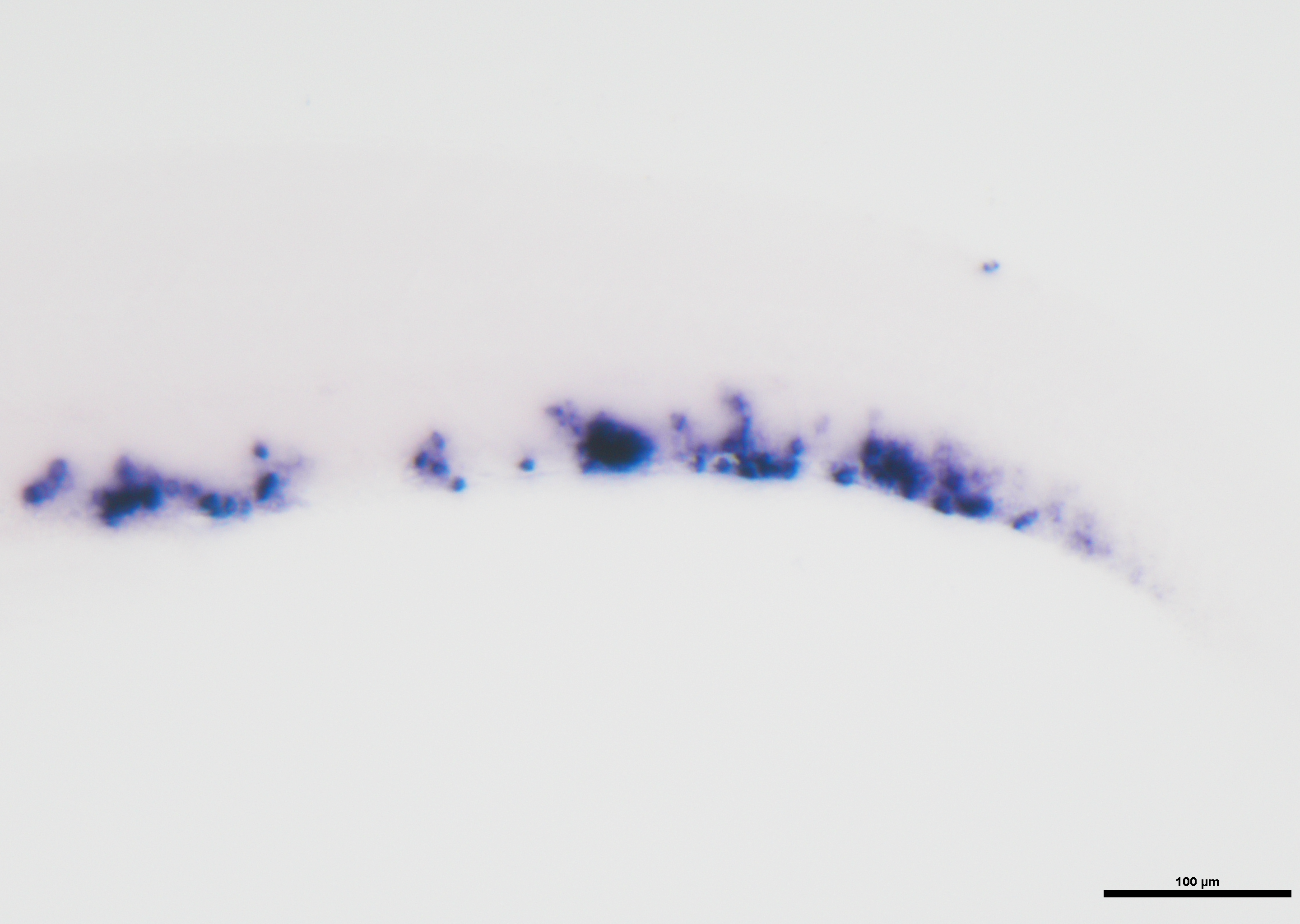

Supplement: Supplementary file 6 — Source data Fig. 1 [file 44319_2026_805_MOESM6_ESM.zip › Source Data Fig.1/Fig.1/D/4. hbae1.1 5dpf trmt61aMO.tif]

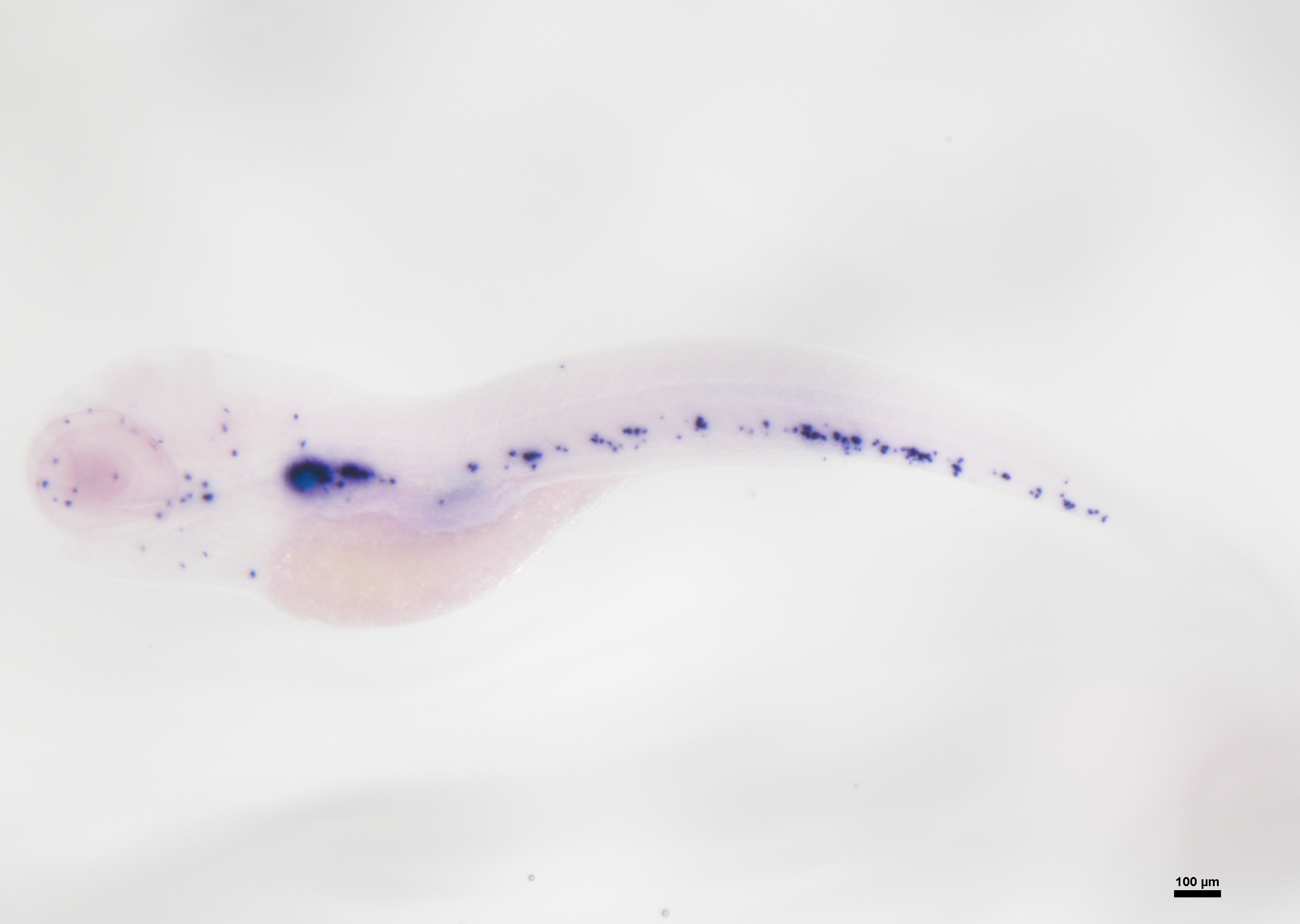

Supplement: Supplementary file 6 — Source data Fig. 1 [file 44319_2026_805_MOESM6_ESM.zip › Source Data Fig.1/Fig.1/D/5. lyz 5dpf controlMO.tif]

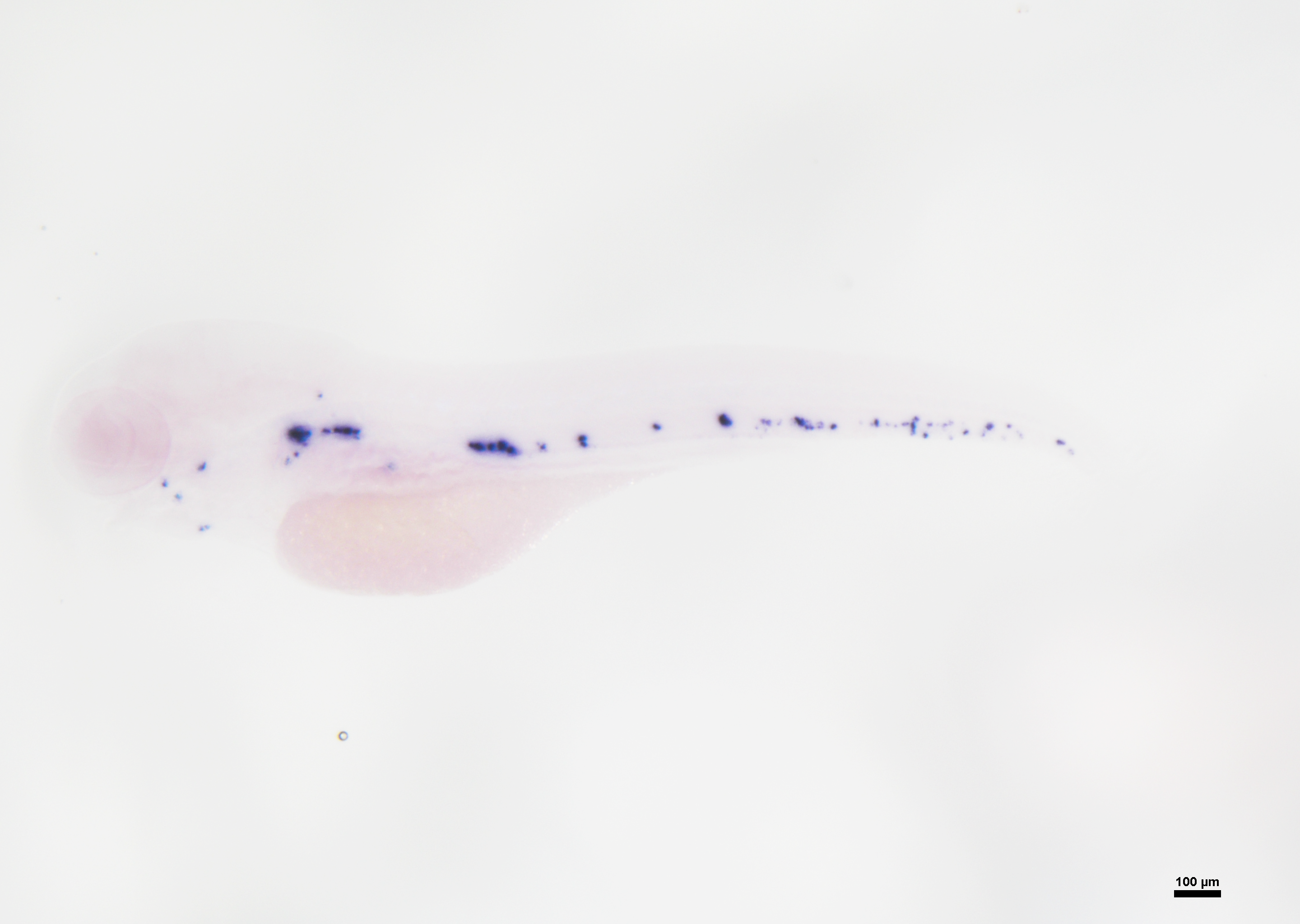

Supplement: Supplementary file 6 — Source data Fig. 1 [file 44319_2026_805_MOESM6_ESM.zip › Source Data Fig.1/Fig.1/D/6. lyz 5dpf trmt61aMO.tif]

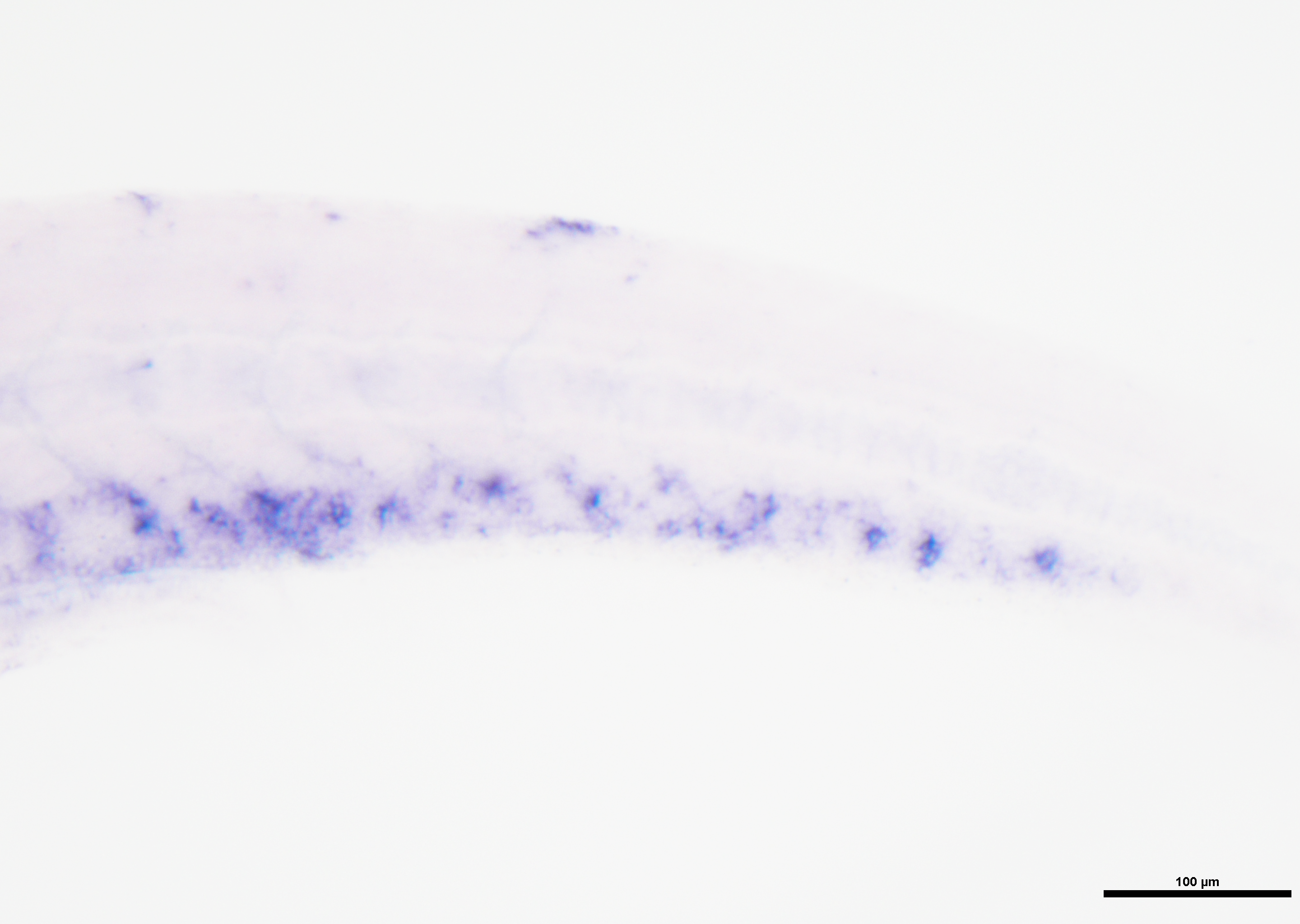

Supplement: Supplementary file 6 — Source data Fig. 1 [file 44319_2026_805_MOESM6_ESM.zip › Source Data Fig.1/Fig.1/D/7. pu.1 5dpf ControlMO.tif]

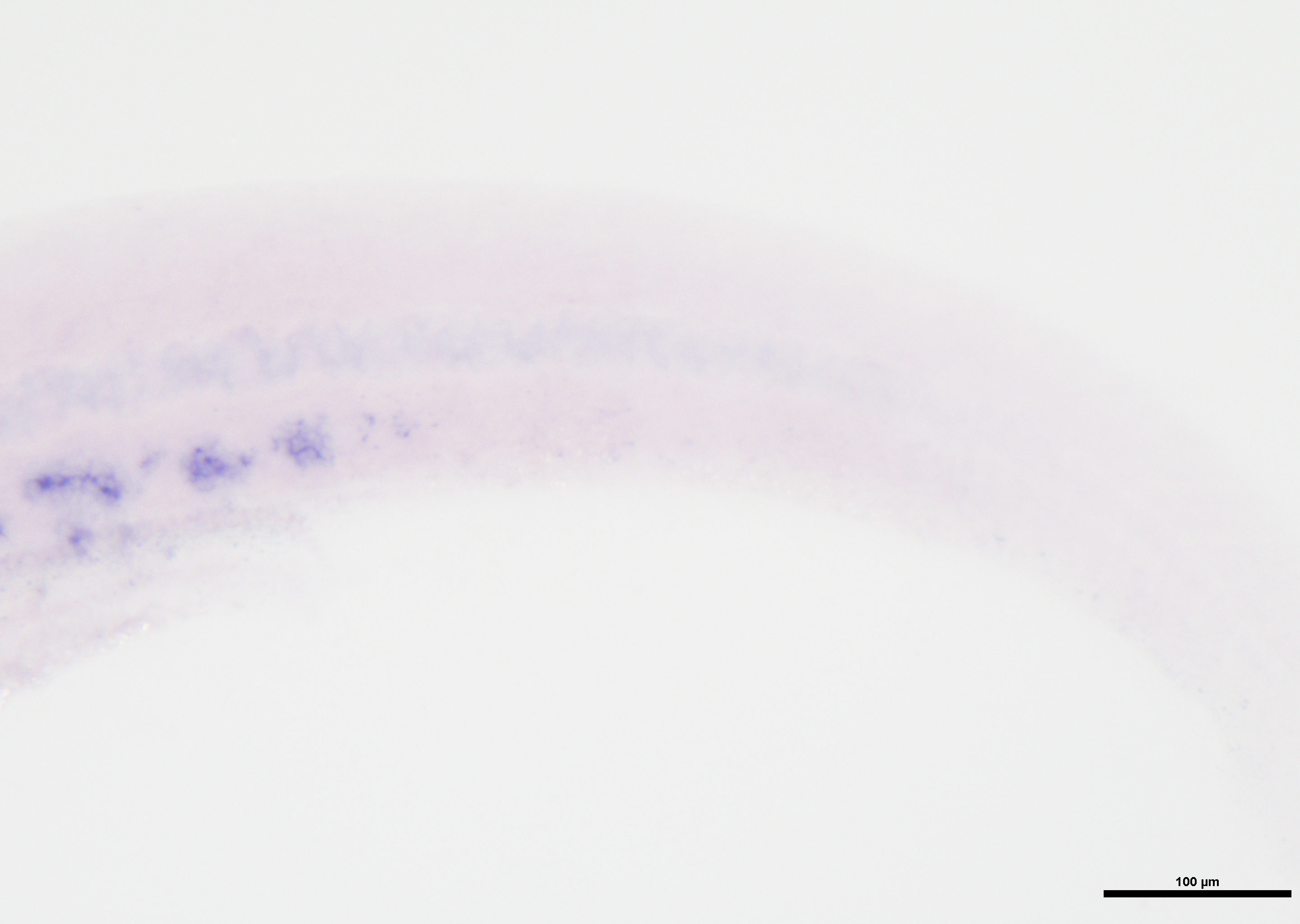

Supplement: Supplementary file 6 — Source data Fig. 1 [file 44319_2026_805_MOESM6_ESM.zip › Source Data Fig.1/Fig.1/D/8. pu.1 5dpf trmt61aMO-2.tif]

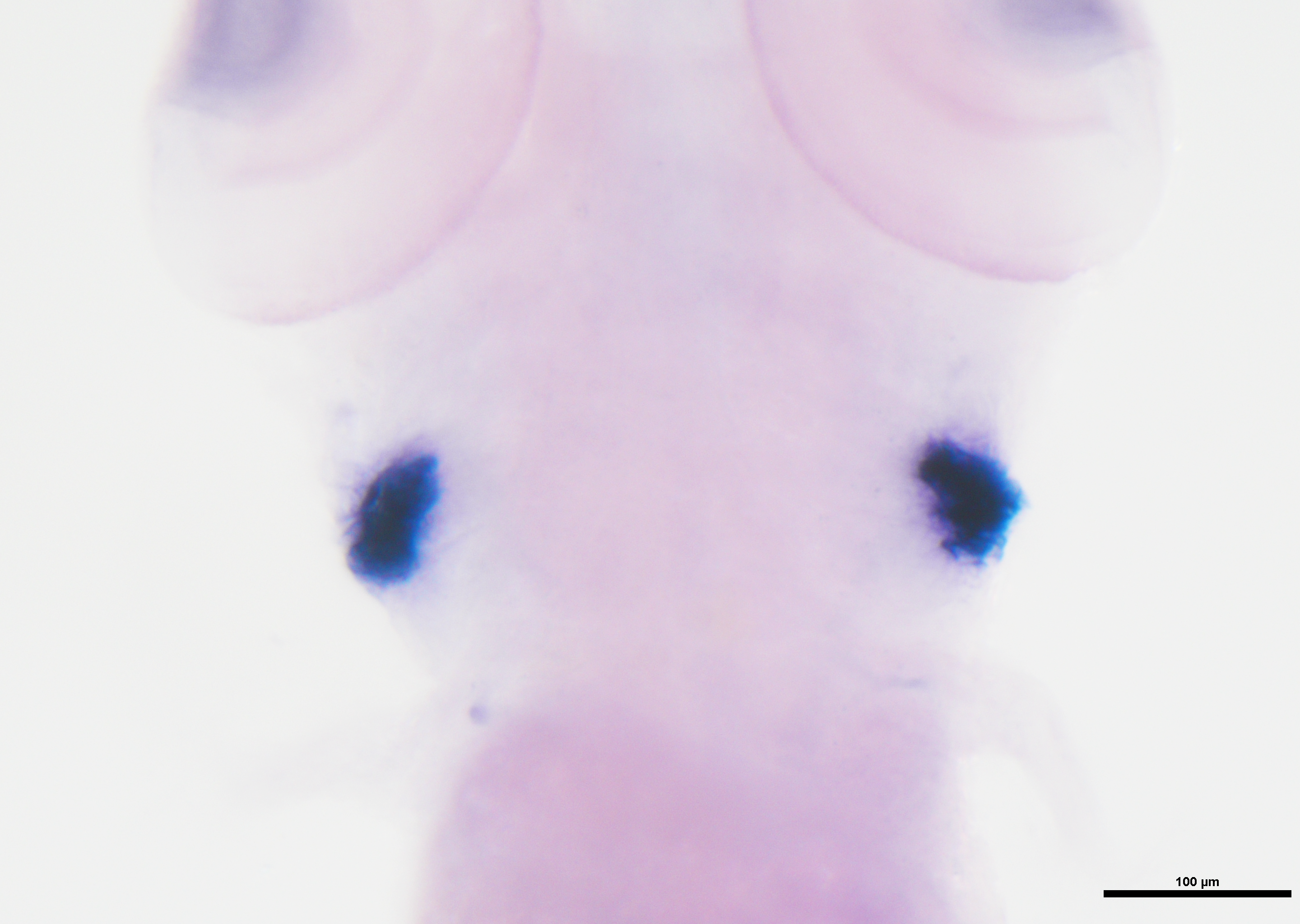

Supplement: Supplementary file 6 — Source data Fig. 1 [file 44319_2026_805_MOESM6_ESM.zip › Source Data Fig.1/Fig.1/D/9. rag1 5dpf controlMO.tif]

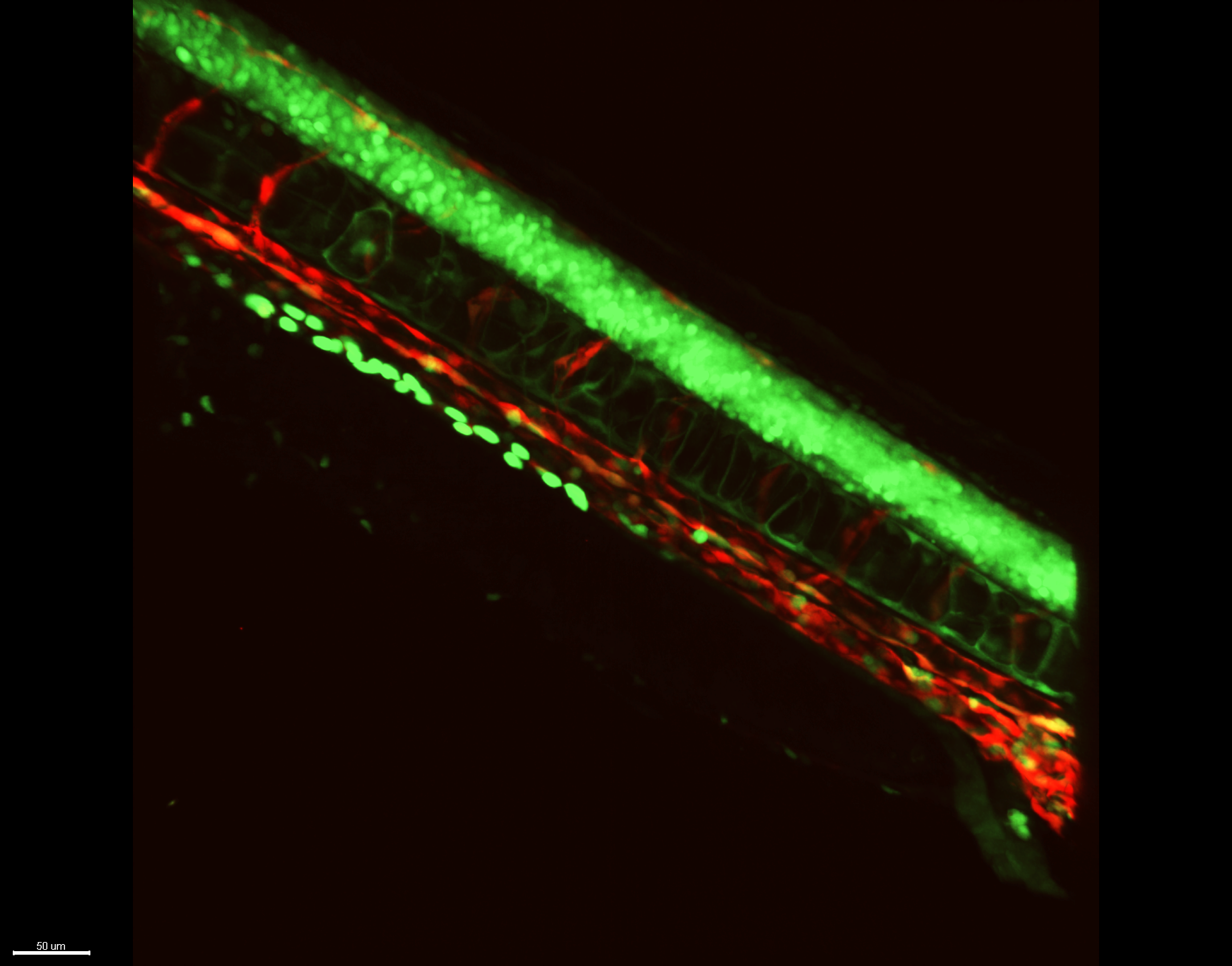

Supplement: Supplementary file 6 — Source data Fig. 1 [file 44319_2026_805_MOESM6_ESM.zip › Source Data Fig.1/Fig.1/F/1. 32hpf controlMO.tif]

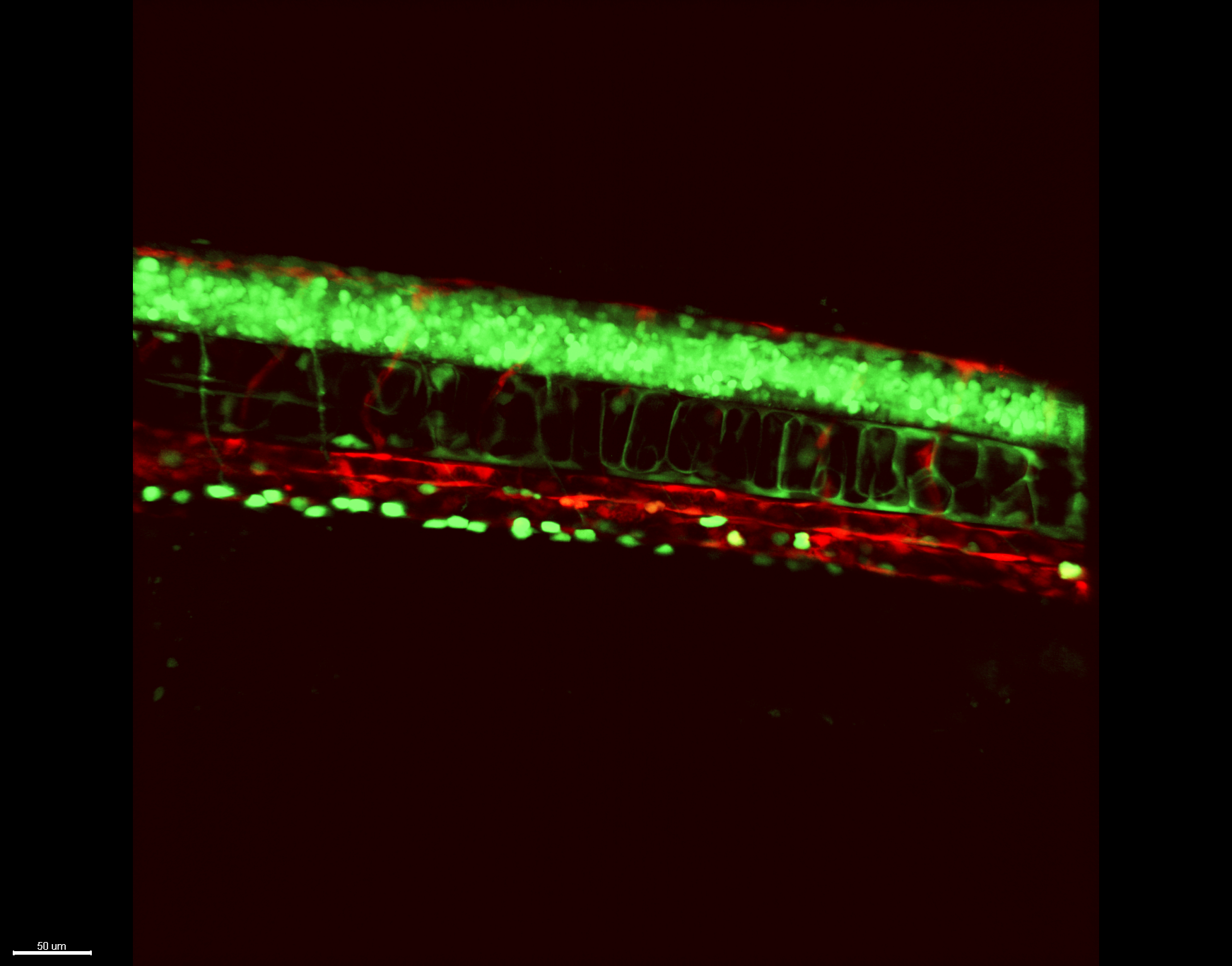

Supplement: Supplementary file 6 — Source data Fig. 1 [file 44319_2026_805_MOESM6_ESM.zip › Source Data Fig.1/Fig.1/F/2. 32hpf trmt61aMO.tif]

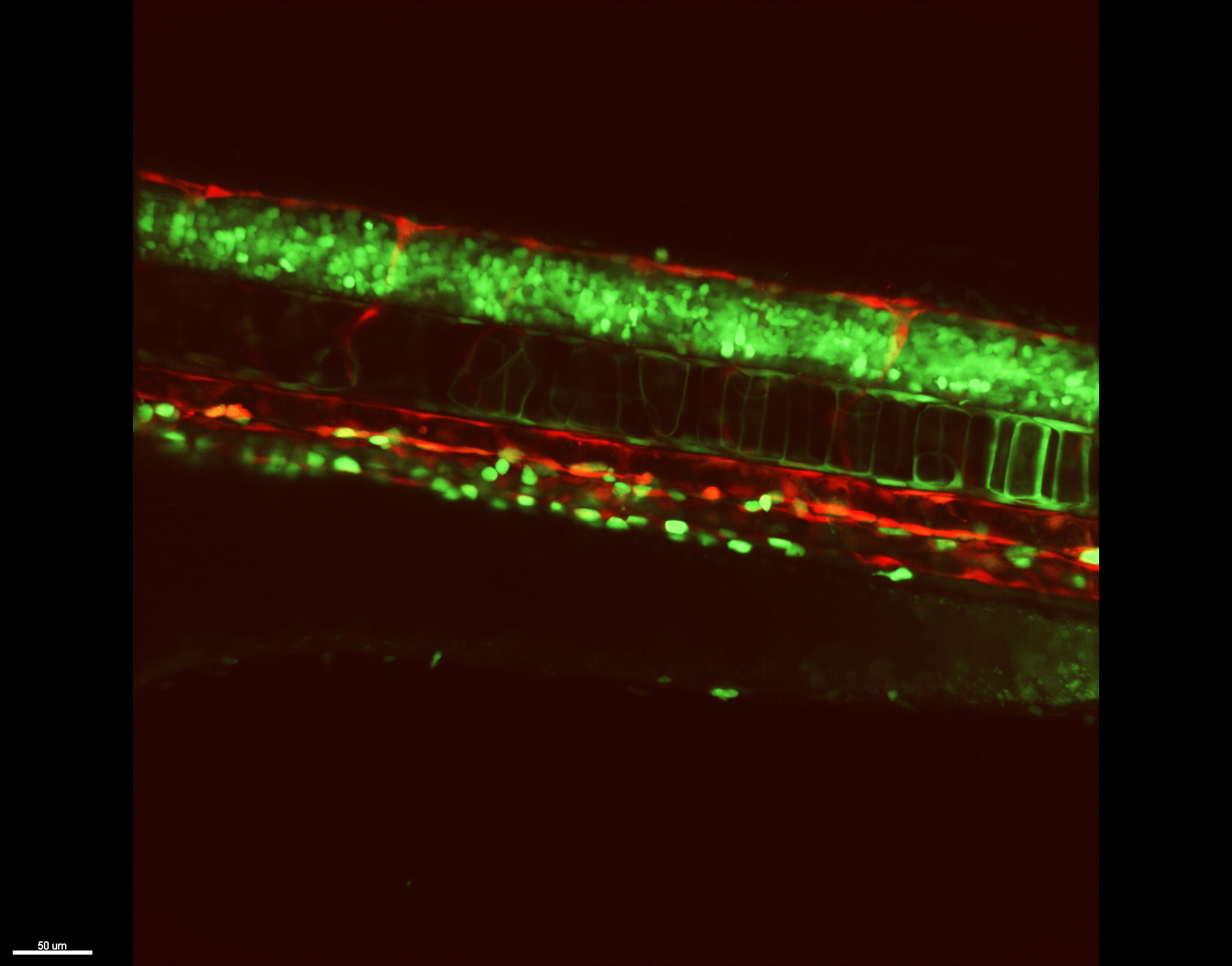

Supplement: Supplementary file 6 — Source data Fig. 1 [file 44319_2026_805_MOESM6_ESM.zip › Source Data Fig.1/Fig.1/F/3. 36hpf controlMO.tif]

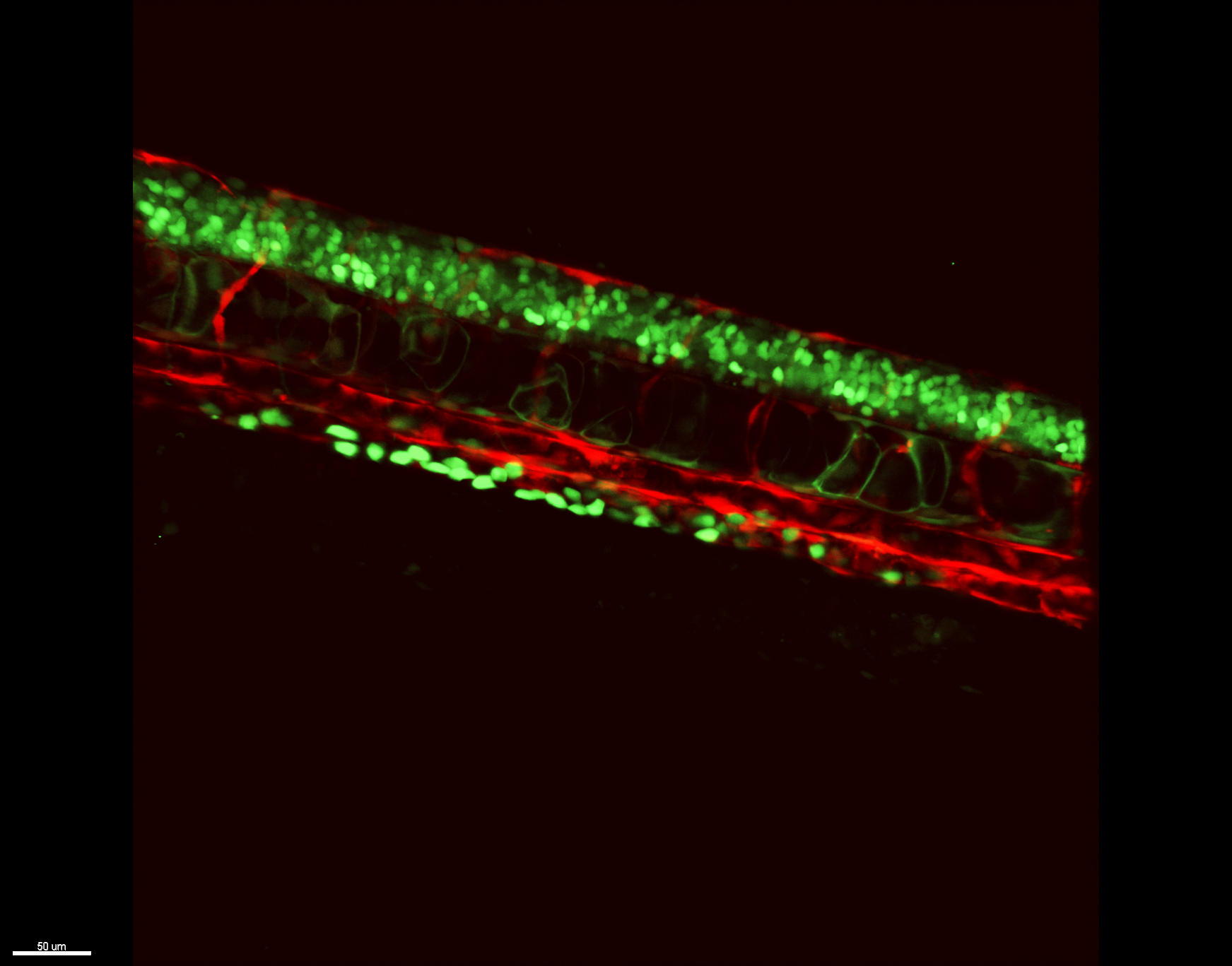

Supplement: Supplementary file 6 — Source data Fig. 1 [file 44319_2026_805_MOESM6_ESM.zip › Source Data Fig.1/Fig.1/F/4. 36hpf trmt61aMO.tif]

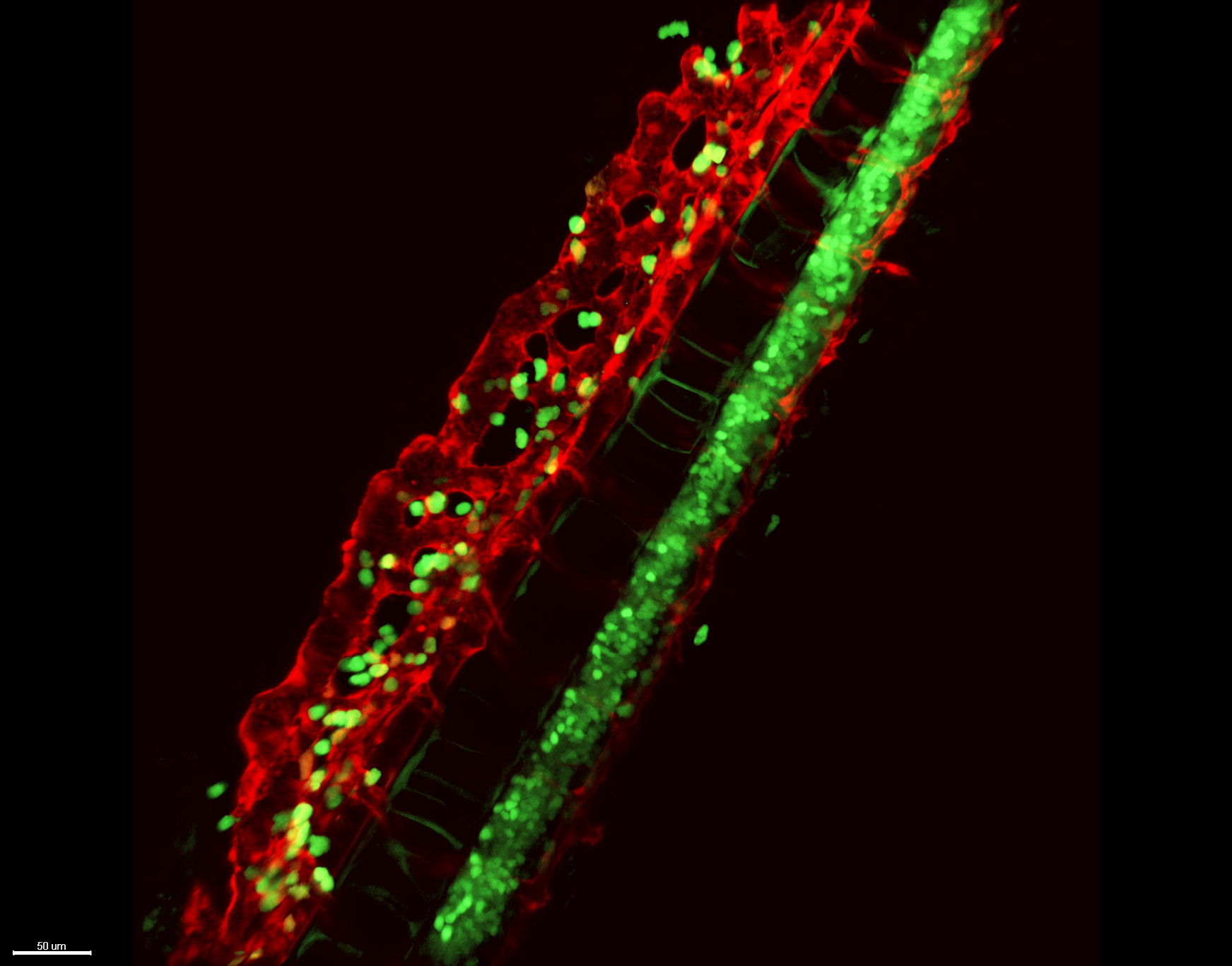

Supplement: Supplementary file 6 — Source data Fig. 1 [file 44319_2026_805_MOESM6_ESM.zip › Source Data Fig.1/Fig.1/F/5. 2dpf Control MO.tif]

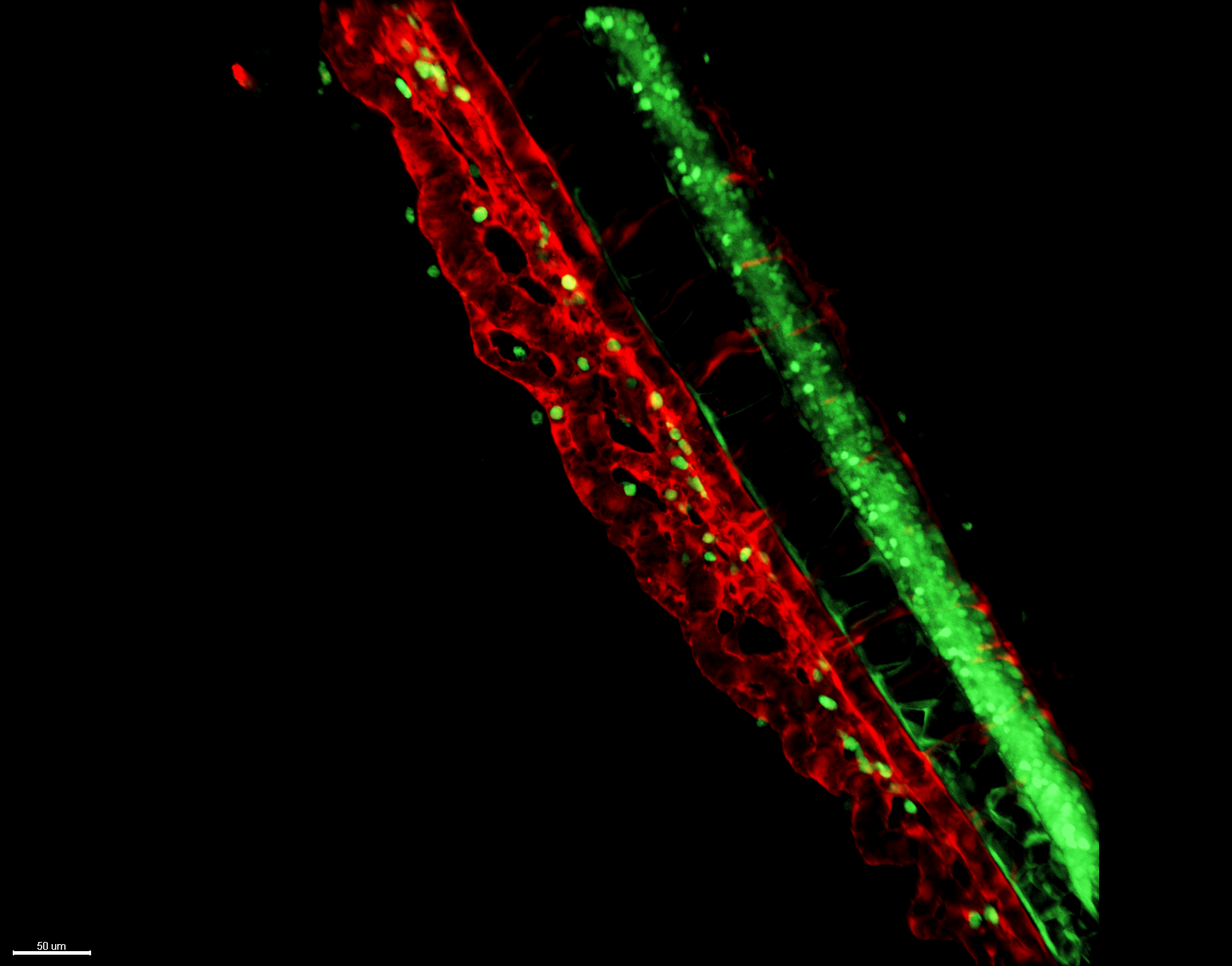

Supplement: Supplementary file 6 — Source data Fig. 1 [file 44319_2026_805_MOESM6_ESM.zip › Source Data Fig.1/Fig.1/F/6. 2dpf trmt61aMO.tif]

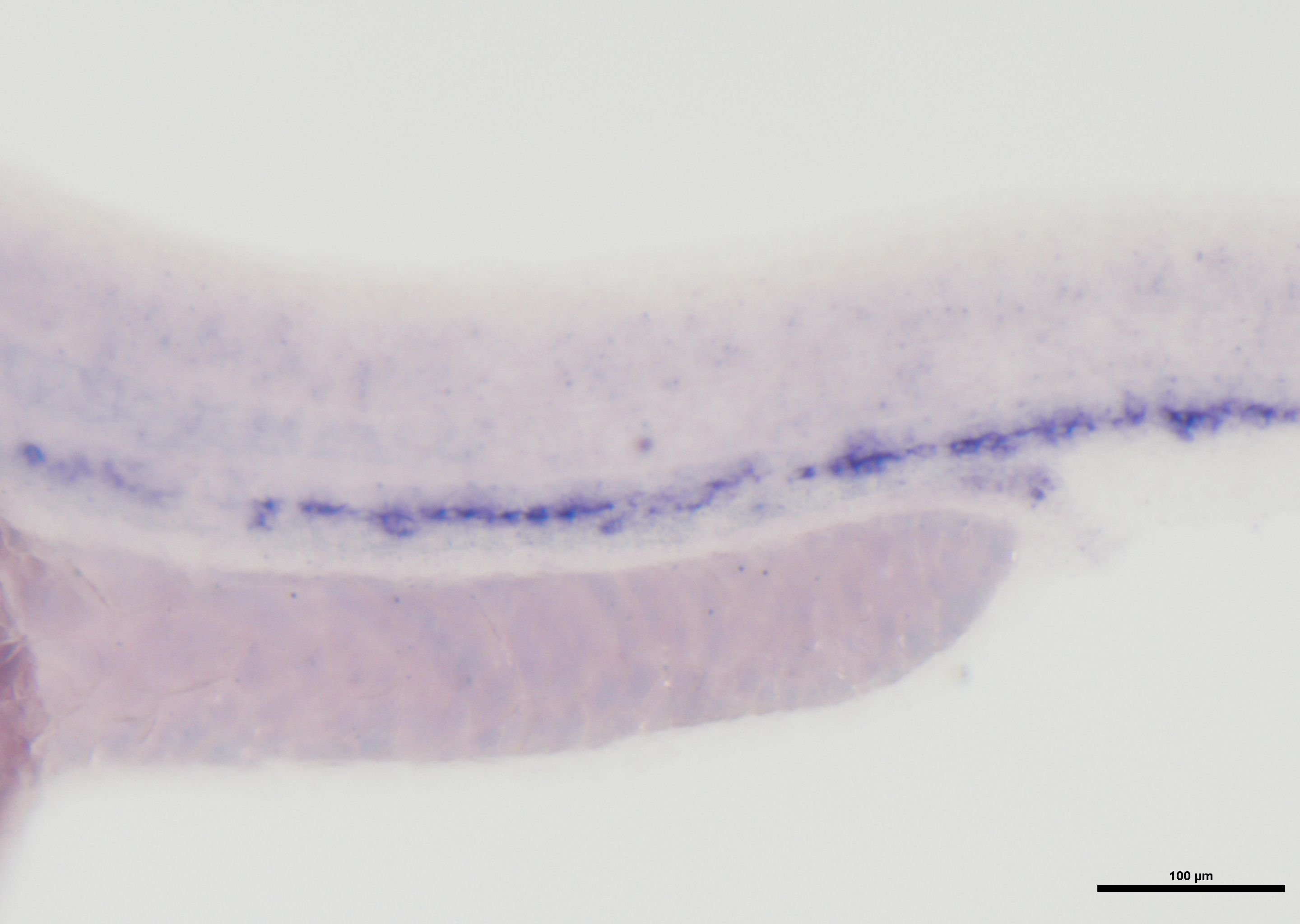

Supplement: Supplementary file 6 — Source data Fig. 1 [file 44319_2026_805_MOESM6_ESM.zip › Source Data Fig.1/Fig.1/H/1. gata2b 32hpf controlMO.tif]

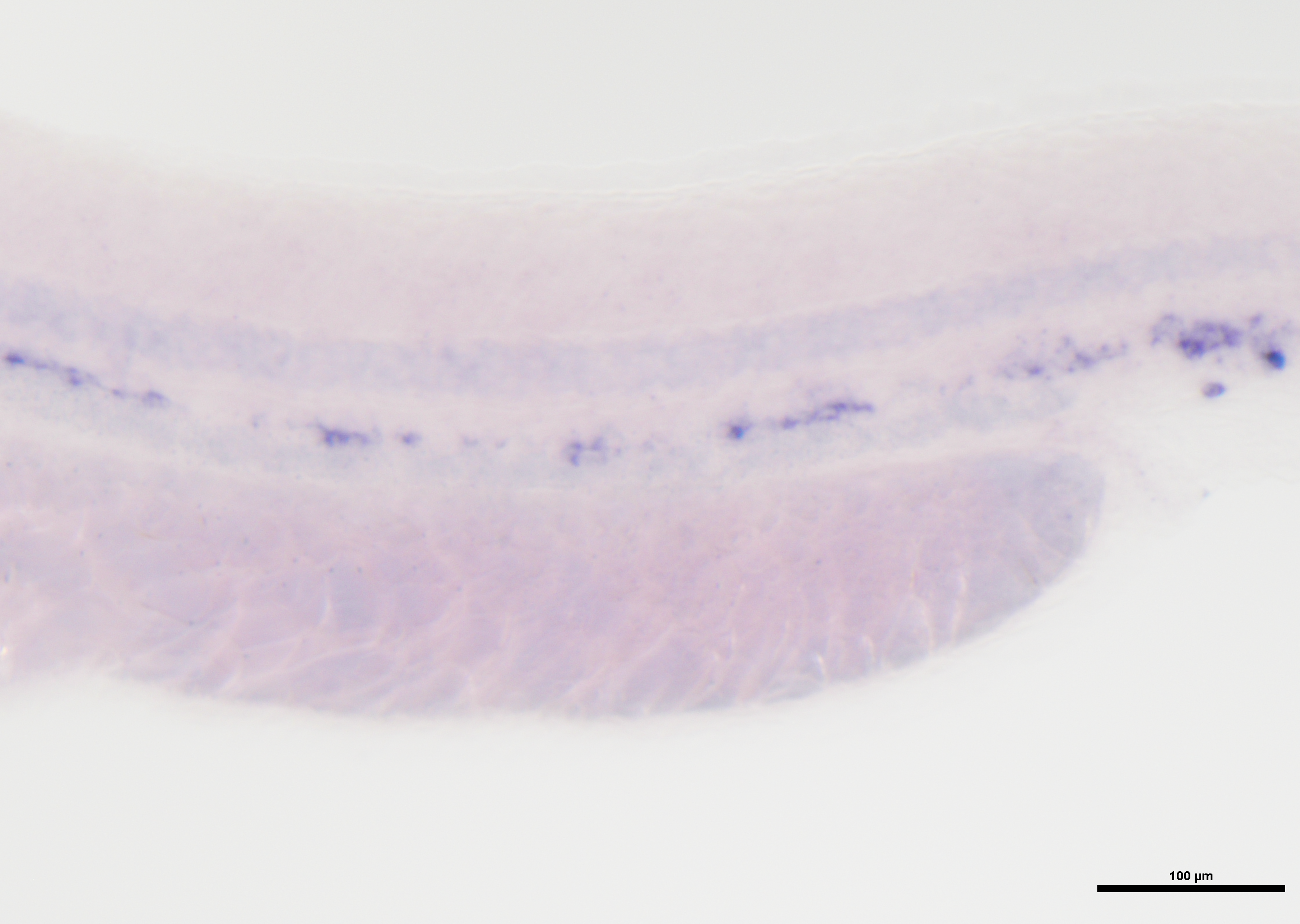

Supplement: Supplementary file 6 — Source data Fig. 1 [file 44319_2026_805_MOESM6_ESM.zip › Source Data Fig.1/Fig.1/H/2. gata2b 32hpf trmt61aMO.tif]

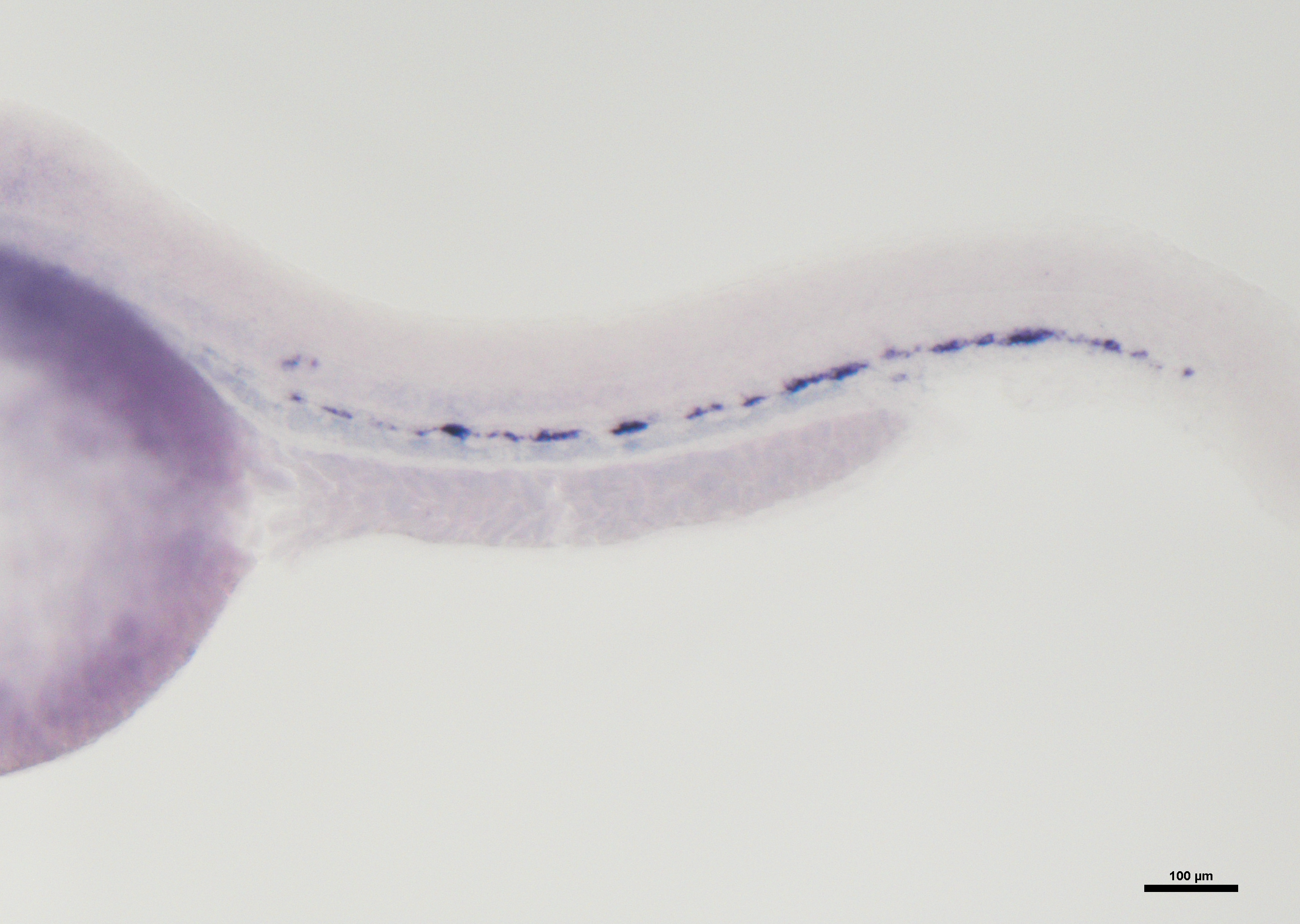

Supplement: Supplementary file 6 — Source data Fig. 1 [file 44319_2026_805_MOESM6_ESM.zip › Source Data Fig.1/Fig.1/H/3. gfi1aa 32hpf controlMO.tif]

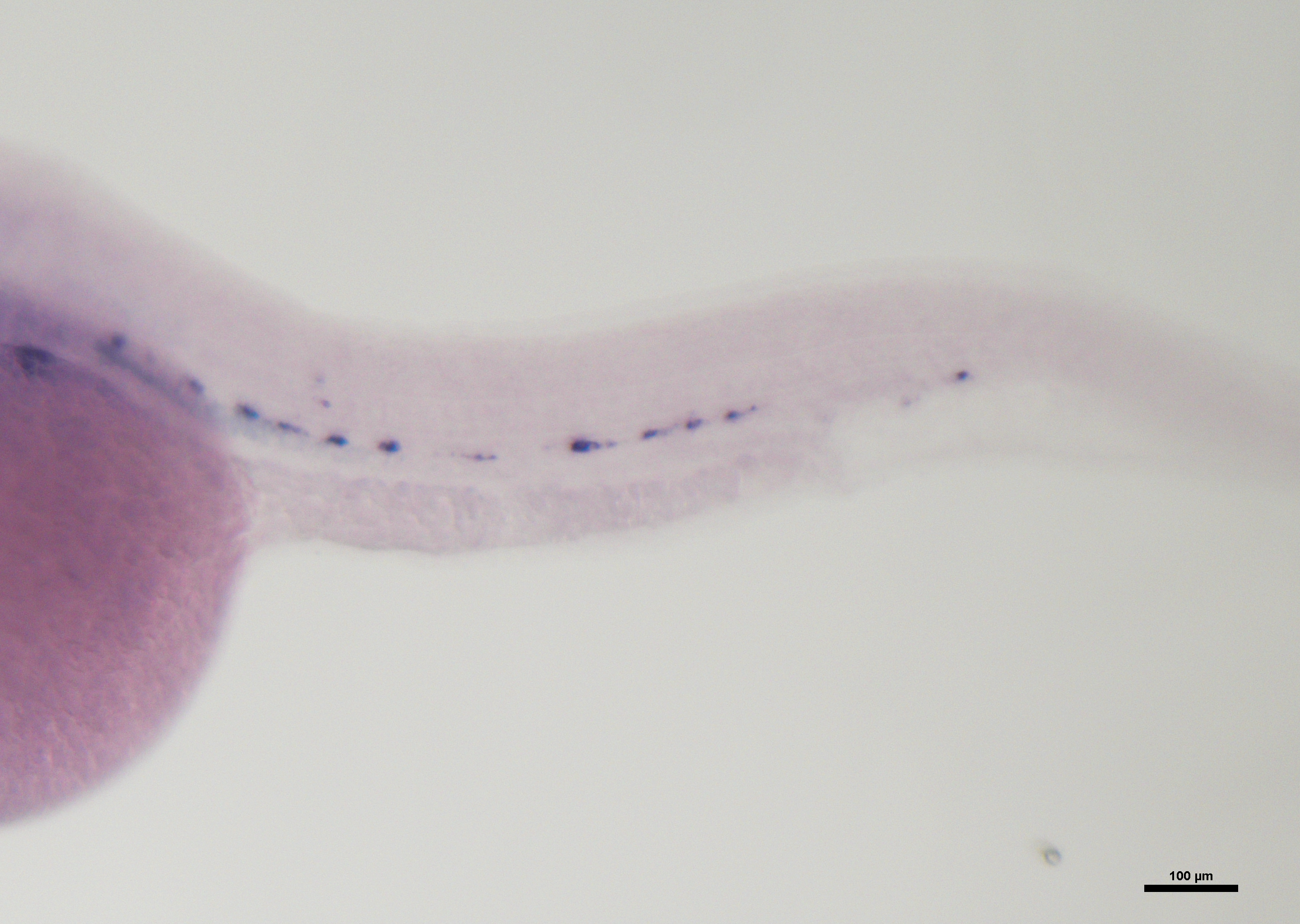

Supplement: Supplementary file 6 — Source data Fig. 1 [file 44319_2026_805_MOESM6_ESM.zip › Source Data Fig.1/Fig.1/H/4. gfi1aa 32hpf trmt61aMO.tif]

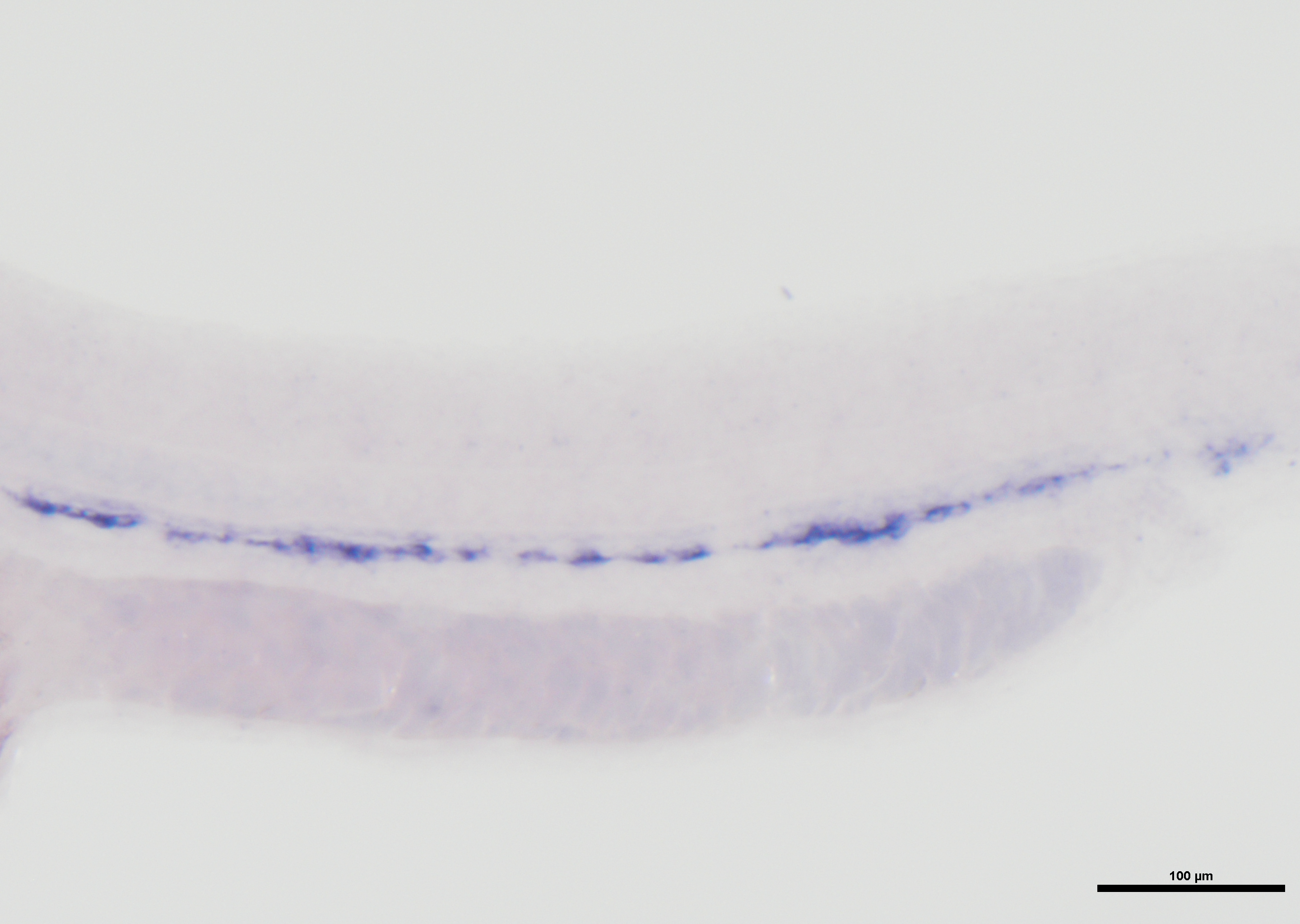

Supplement: Supplementary file 6 — Source data Fig. 1 [file 44319_2026_805_MOESM6_ESM.zip › Source Data Fig.1/Fig.1/H/5. gata2b 36hpf controlMO.tif]

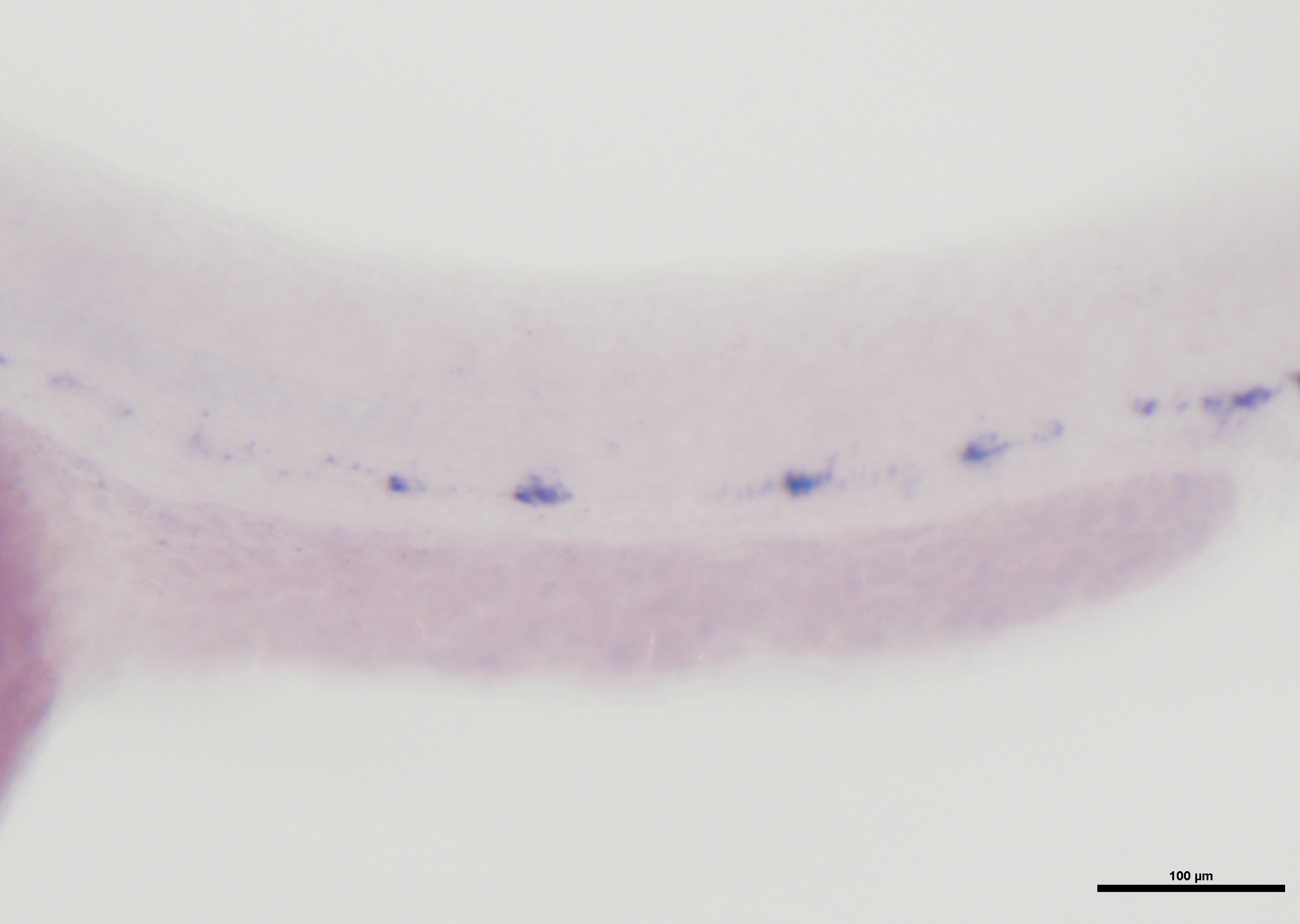

Supplement: Supplementary file 6 — Source data Fig. 1 [file 44319_2026_805_MOESM6_ESM.zip › Source Data Fig.1/Fig.1/H/6. gata2b 36hpf trmt61aMO.tif]

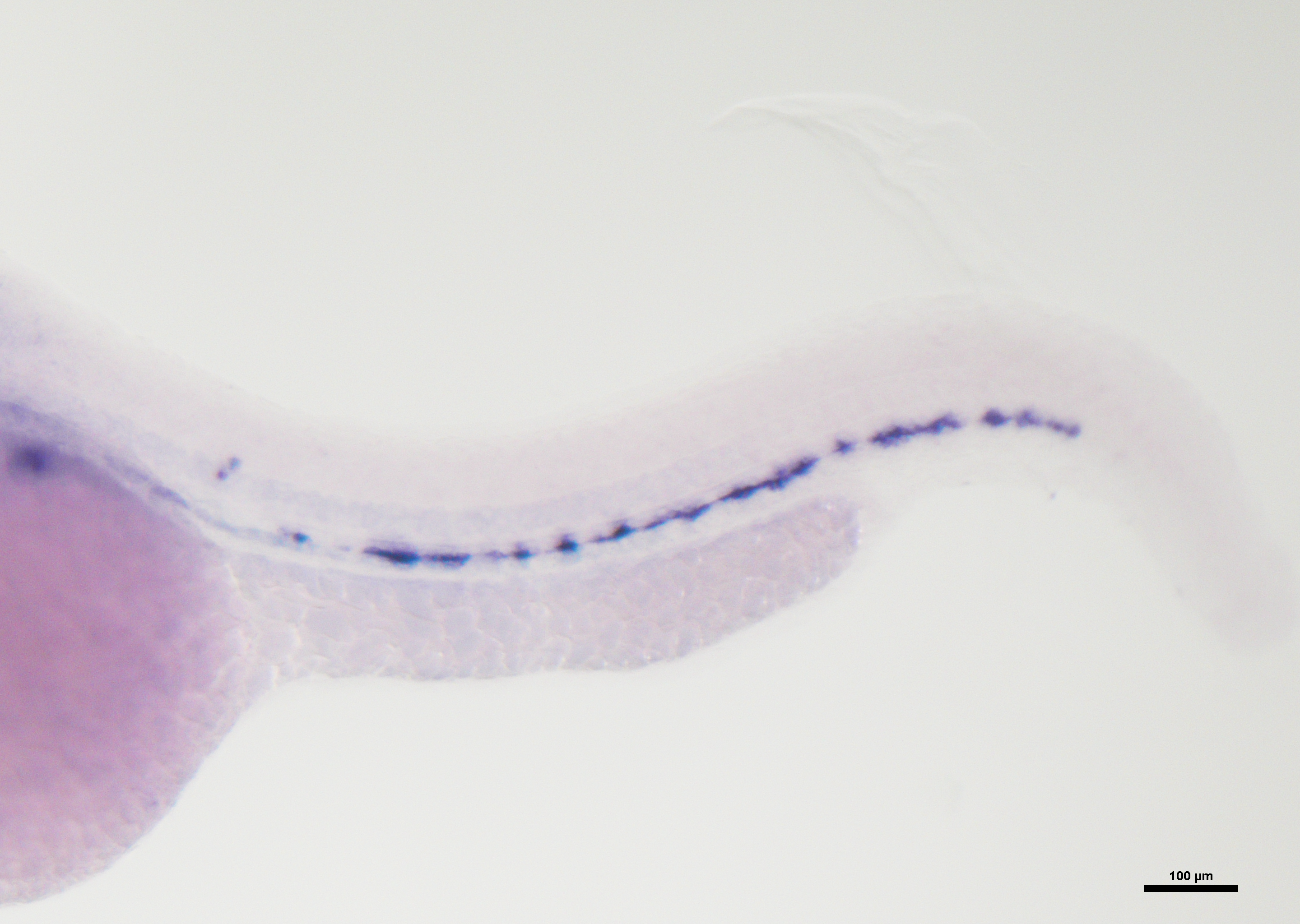

Supplement: Supplementary file 6 — Source data Fig. 1 [file 44319_2026_805_MOESM6_ESM.zip › Source Data Fig.1/Fig.1/H/7. gfi1aa 36hpf controlMO.tif]

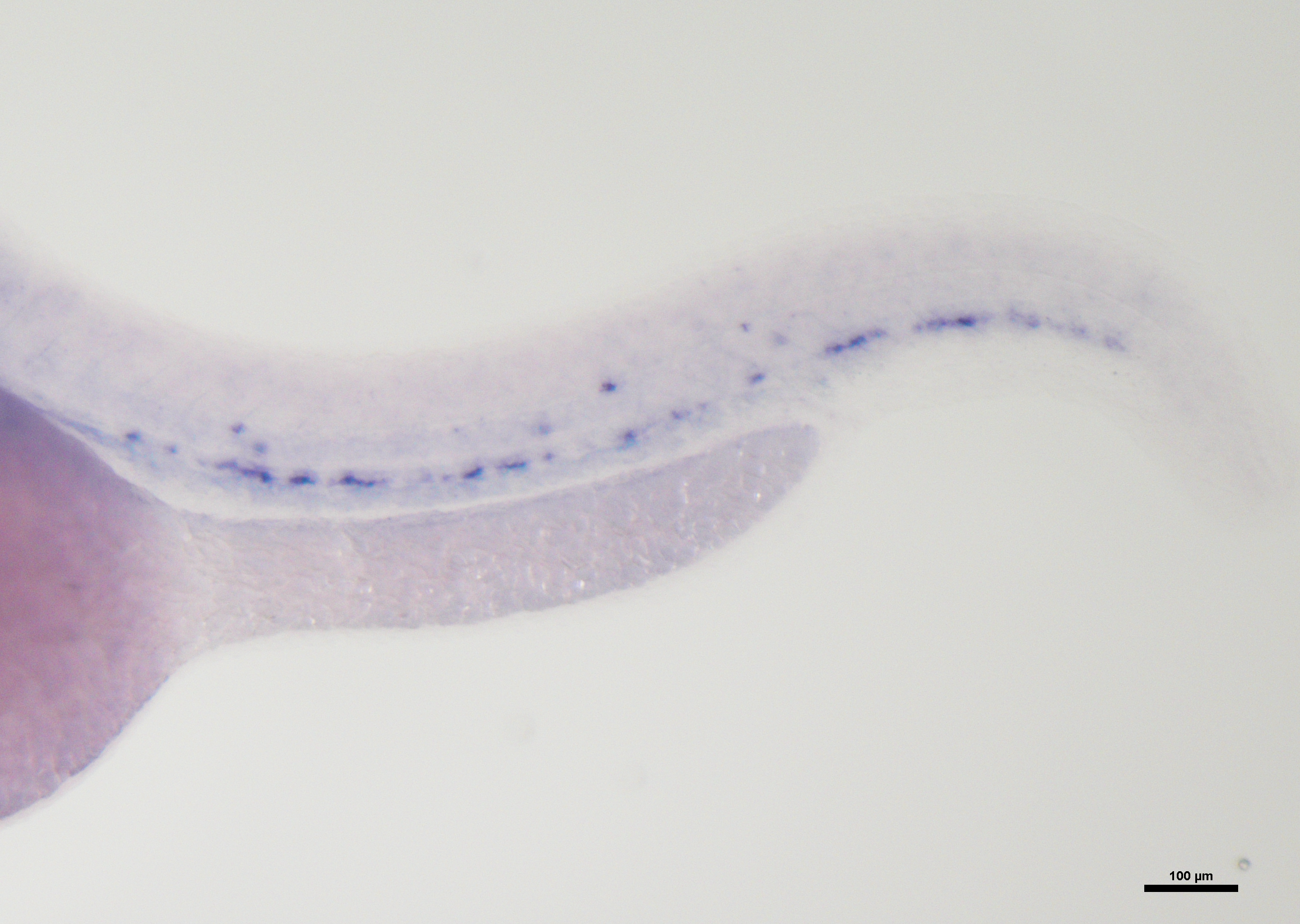

Supplement: Supplementary file 6 — Source data Fig. 1 [file 44319_2026_805_MOESM6_ESM.zip › Source Data Fig.1/Fig.1/H/8. gfi1aa 36hpf trmt61aMO.tif]

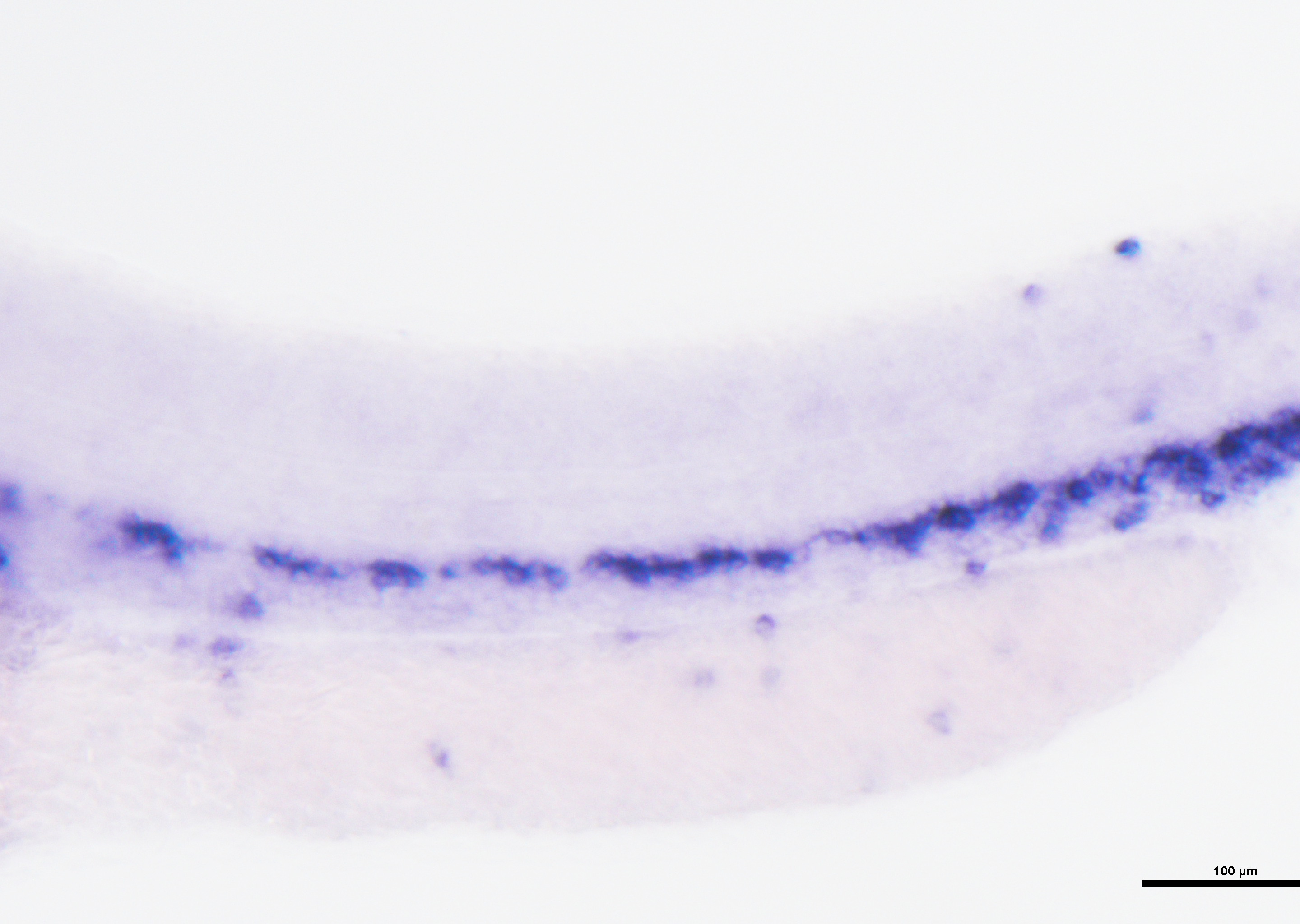

Supplement: Supplementary file 6 — Source data Fig. 1 [file 44319_2026_805_MOESM6_ESM.zip › Source Data Fig.1/Fig.1/K/1. cmyb 36hpf sibing.tif]

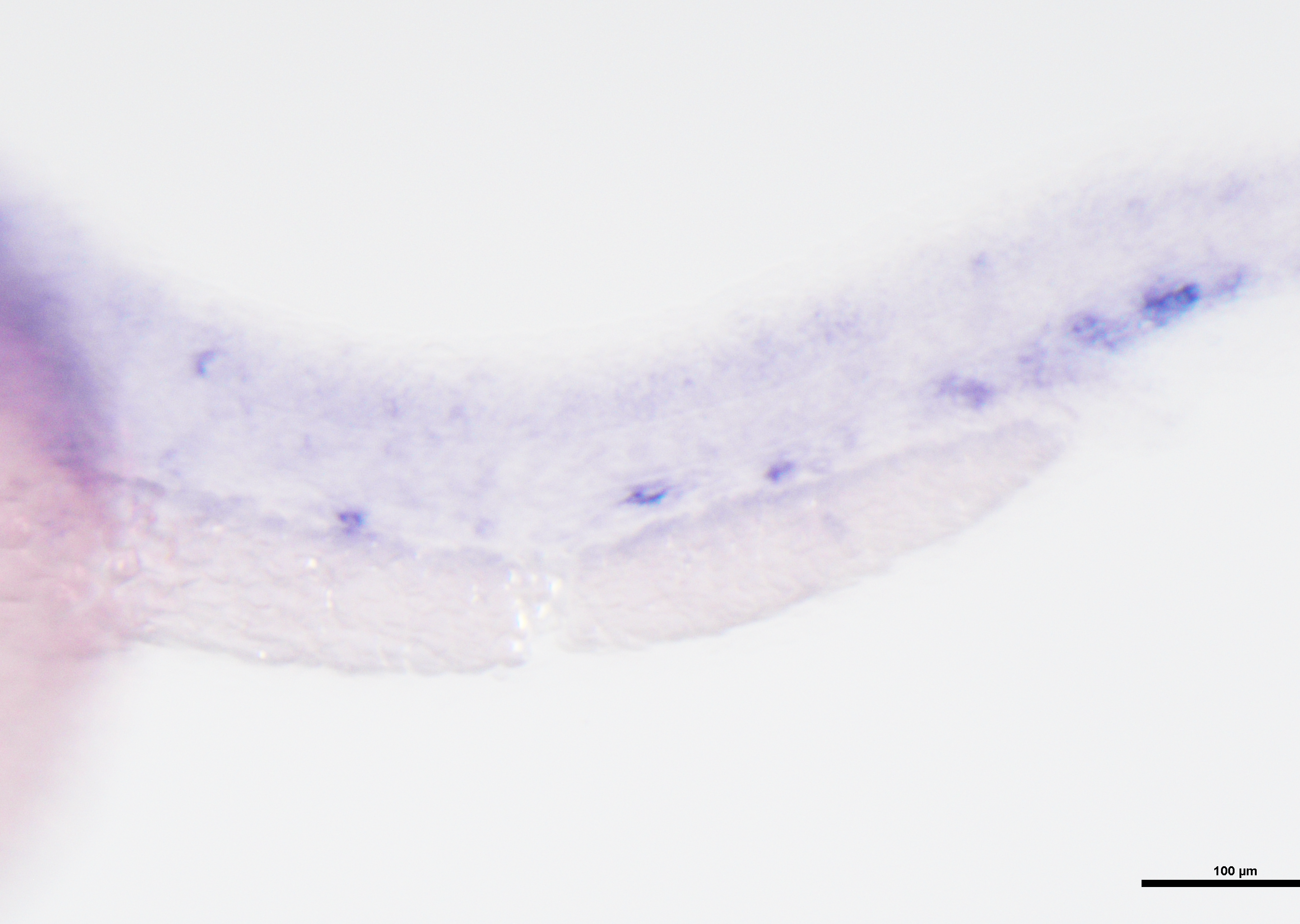

Supplement: Supplementary file 6 — Source data Fig. 1 [file 44319_2026_805_MOESM6_ESM.zip › Source Data Fig.1/Fig.1/K/2. cmyb 36hpf Mtrmt61a;trmt61a-4bp.tif]

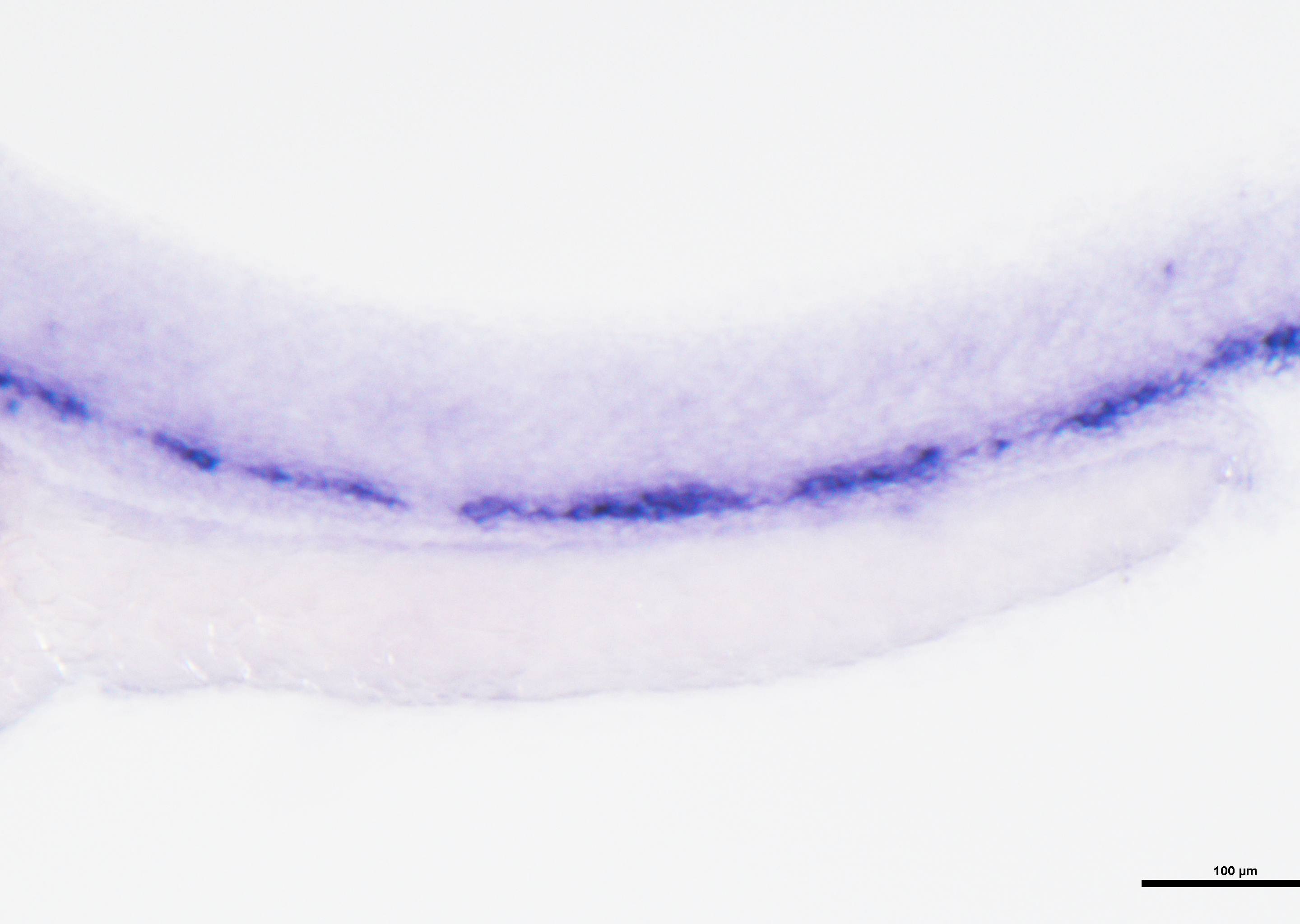

Supplement: Supplementary file 6 — Source data Fig. 1 [file 44319_2026_805_MOESM6_ESM.zip › Source Data Fig.1/Fig.1/K/3. runx1 36hpf sibing.tif]

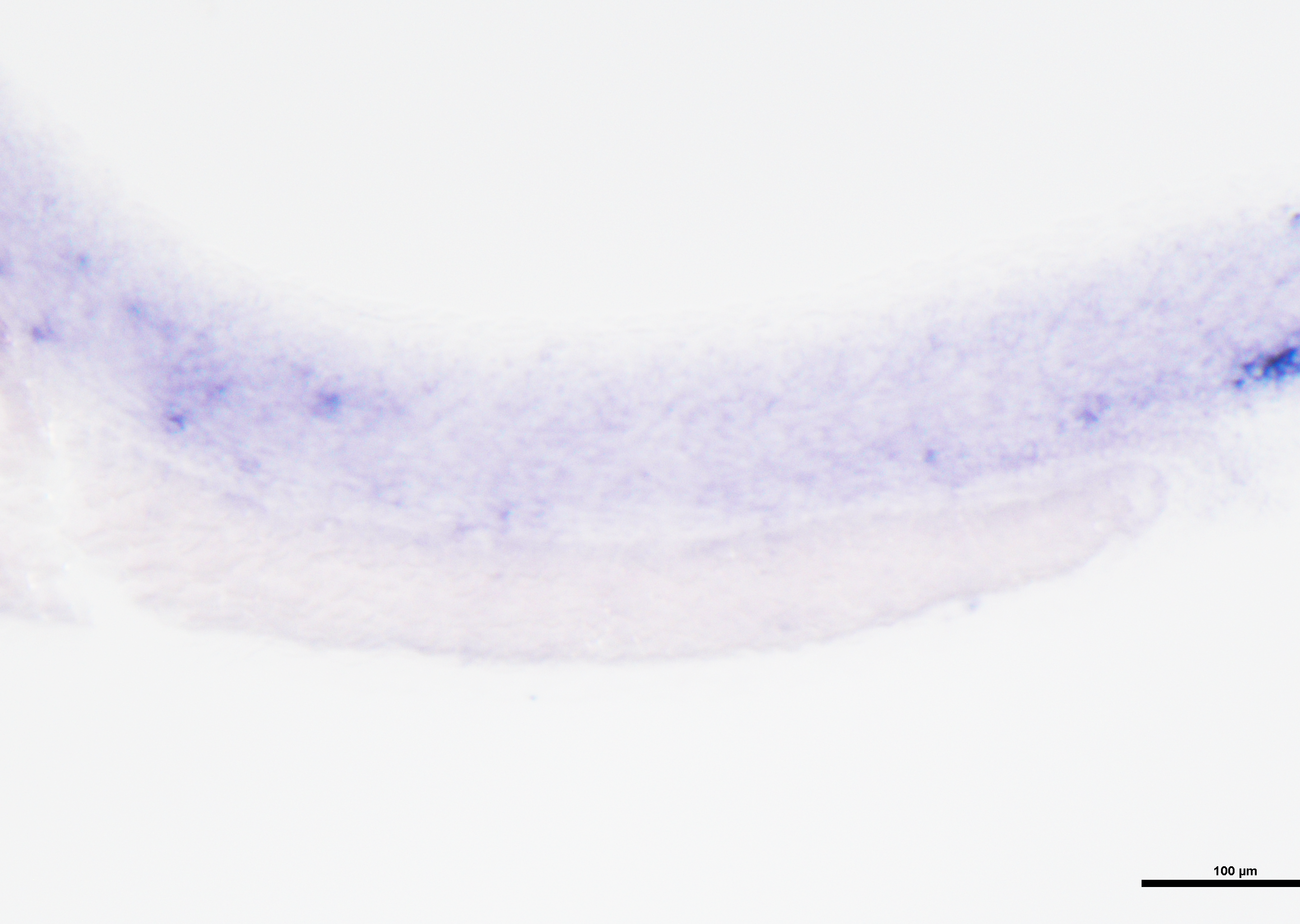

Supplement: Supplementary file 6 — Source data Fig. 1 [file 44319_2026_805_MOESM6_ESM.zip › Source Data Fig.1/Fig.1/K/4. runx1 36hpf Mtrmt61a;trmt61a-4bp.tif]

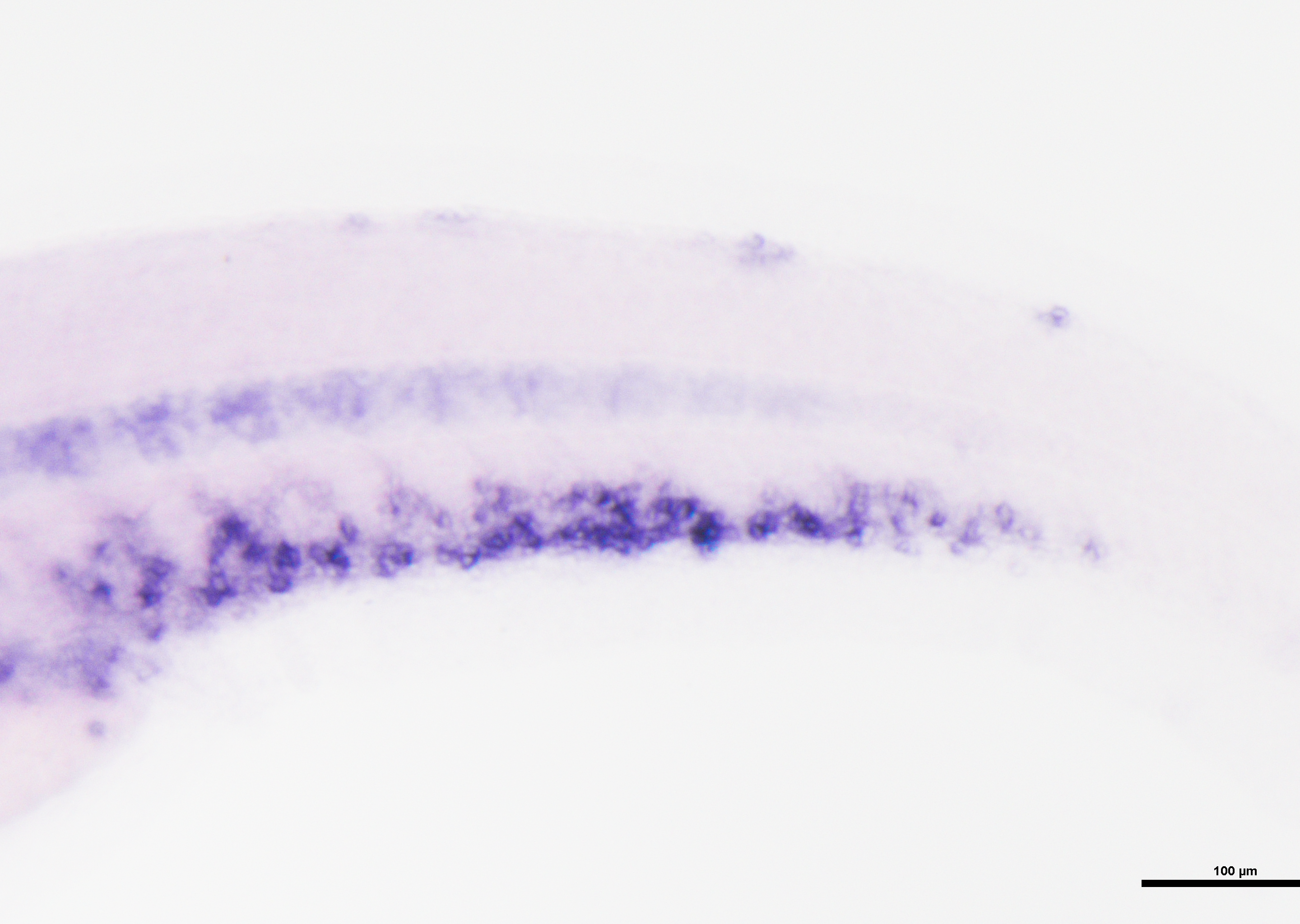

Supplement: Supplementary file 6 — Source data Fig. 1 [file 44319_2026_805_MOESM6_ESM.zip › Source Data Fig.1/Fig.1/K/5. cmyb 2dpf sibing.tif]

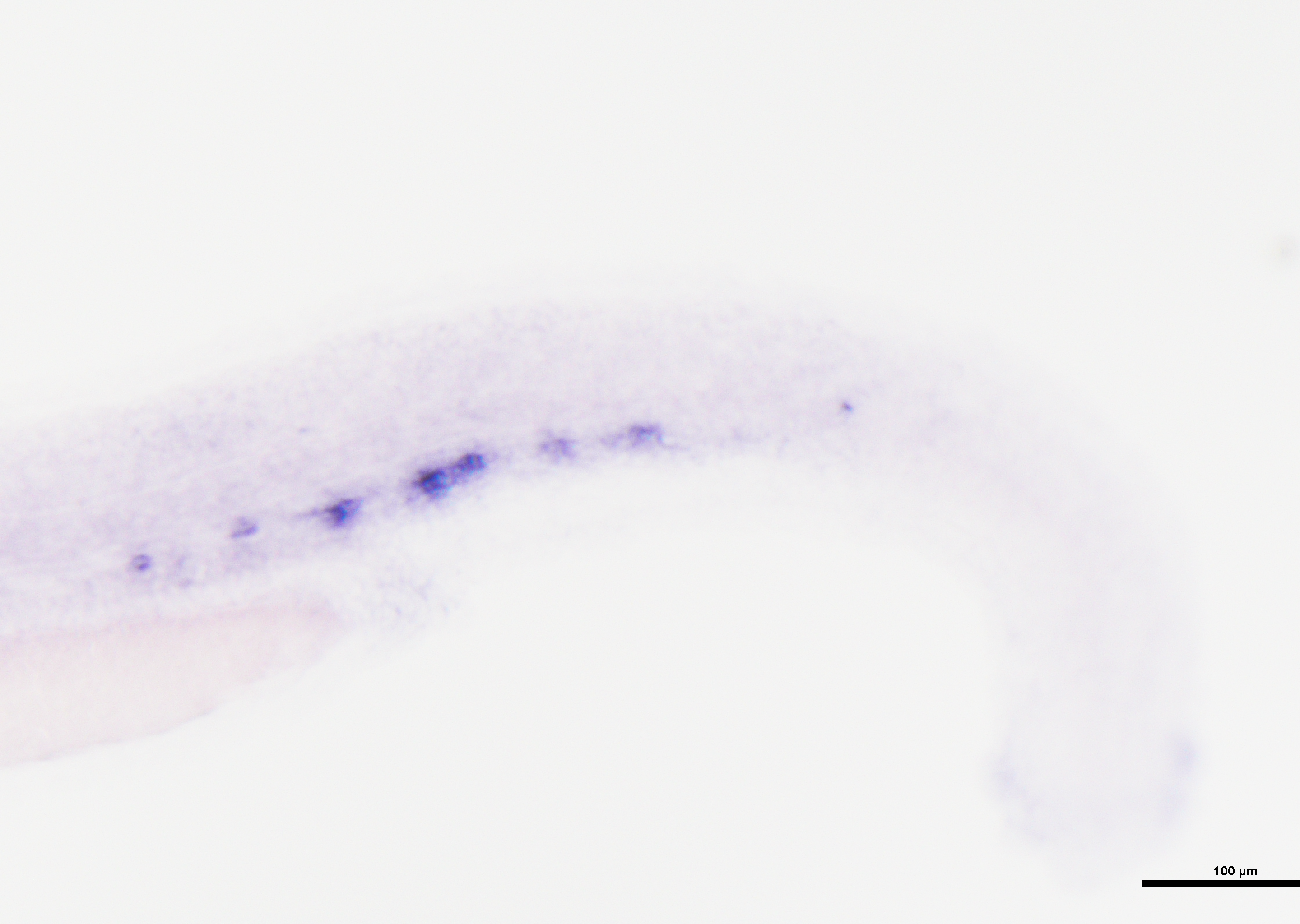

Supplement: Supplementary file 6 — Source data Fig. 1 [file 44319_2026_805_MOESM6_ESM.zip › Source Data Fig.1/Fig.1/K/6. cmyb 2dpf Mtrmt61a;trmt61a-4bp.tif]

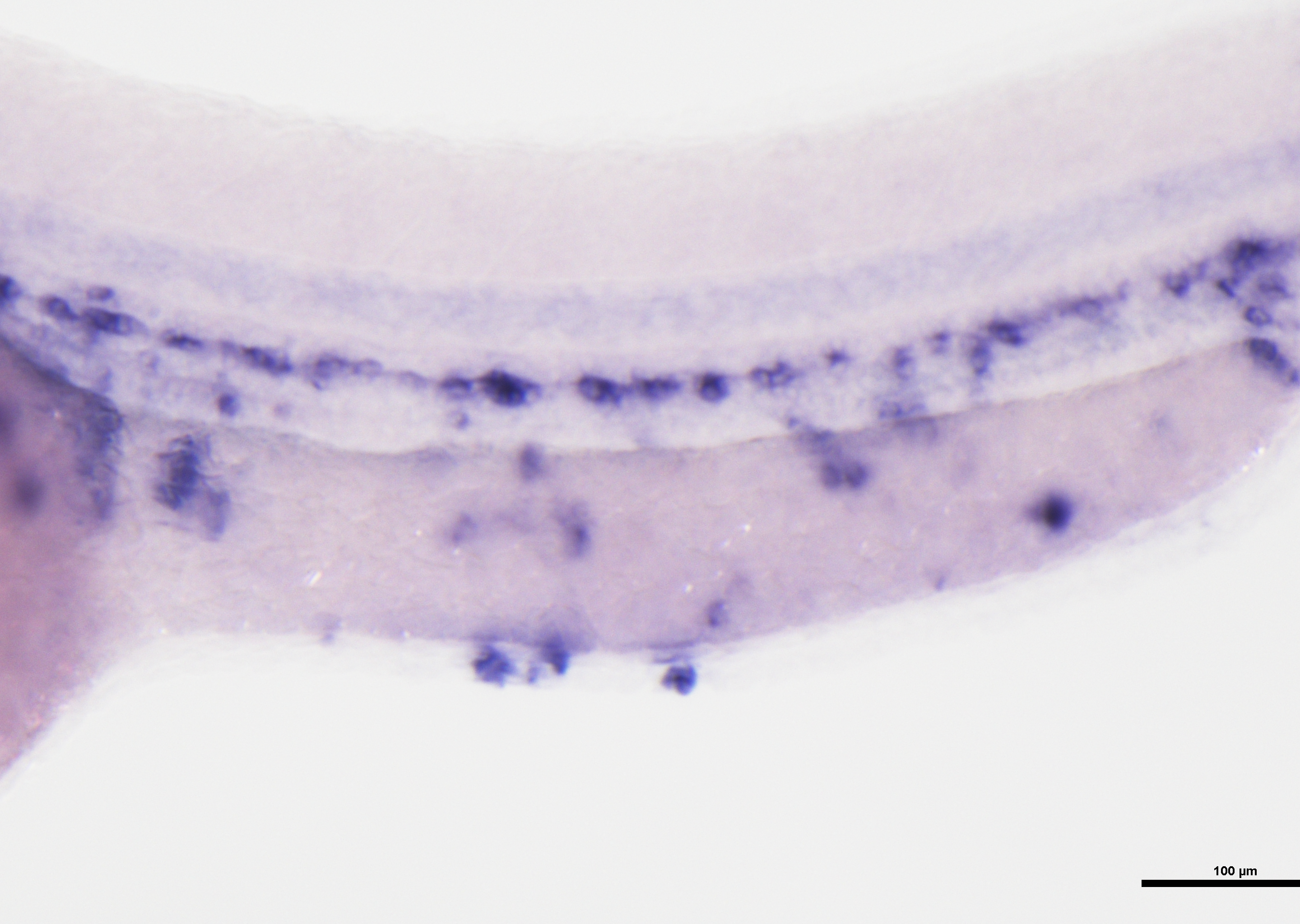

Supplement: Supplementary file 6 — Source data Fig. 1 [file 44319_2026_805_MOESM6_ESM.zip › Source Data Fig.1/Fig.1/L/1. cmyb 36hpf sibling.tif]

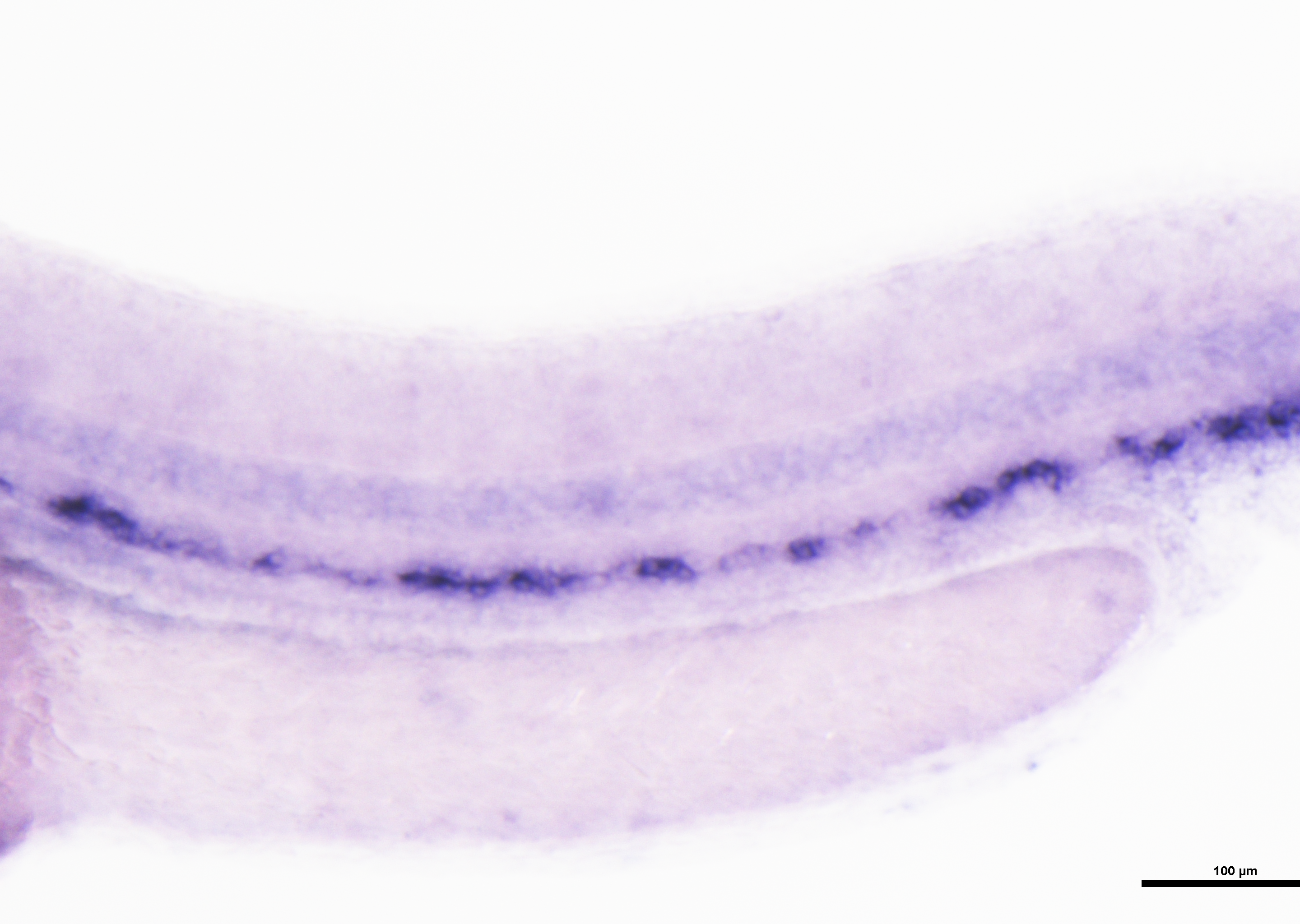

Supplement: Supplementary file 6 — Source data Fig. 1 [file 44319_2026_805_MOESM6_ESM.zip › Source Data Fig.1/Fig.1/L/2. runx1 36hpf sibling.tif]

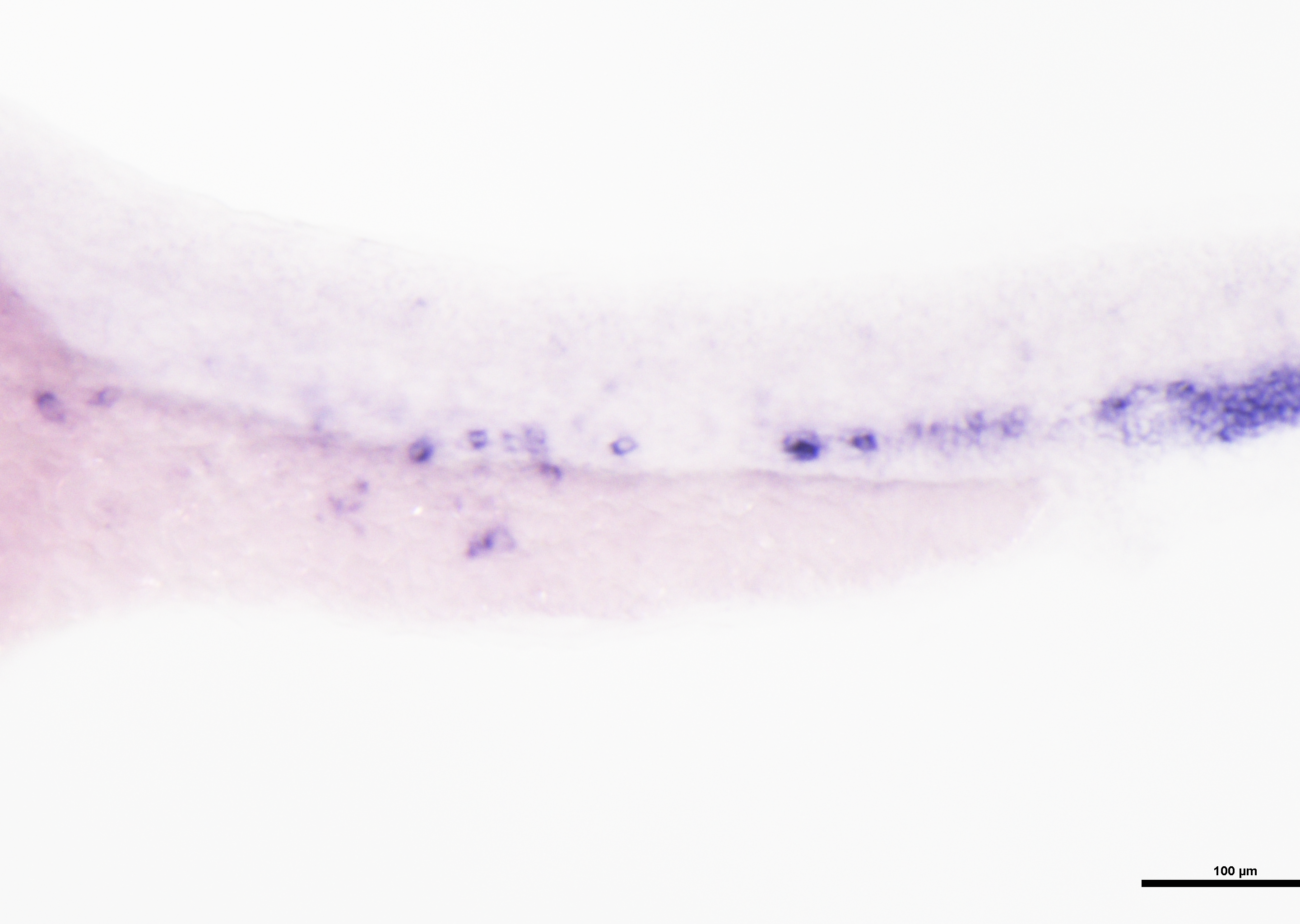

Supplement: Supplementary file 6 — Source data Fig. 1 [file 44319_2026_805_MOESM6_ESM.zip › Source Data Fig.1/Fig.1/L/3. cmyb 36hpf Mtrmt61a;trmt61a-4bp.tif]

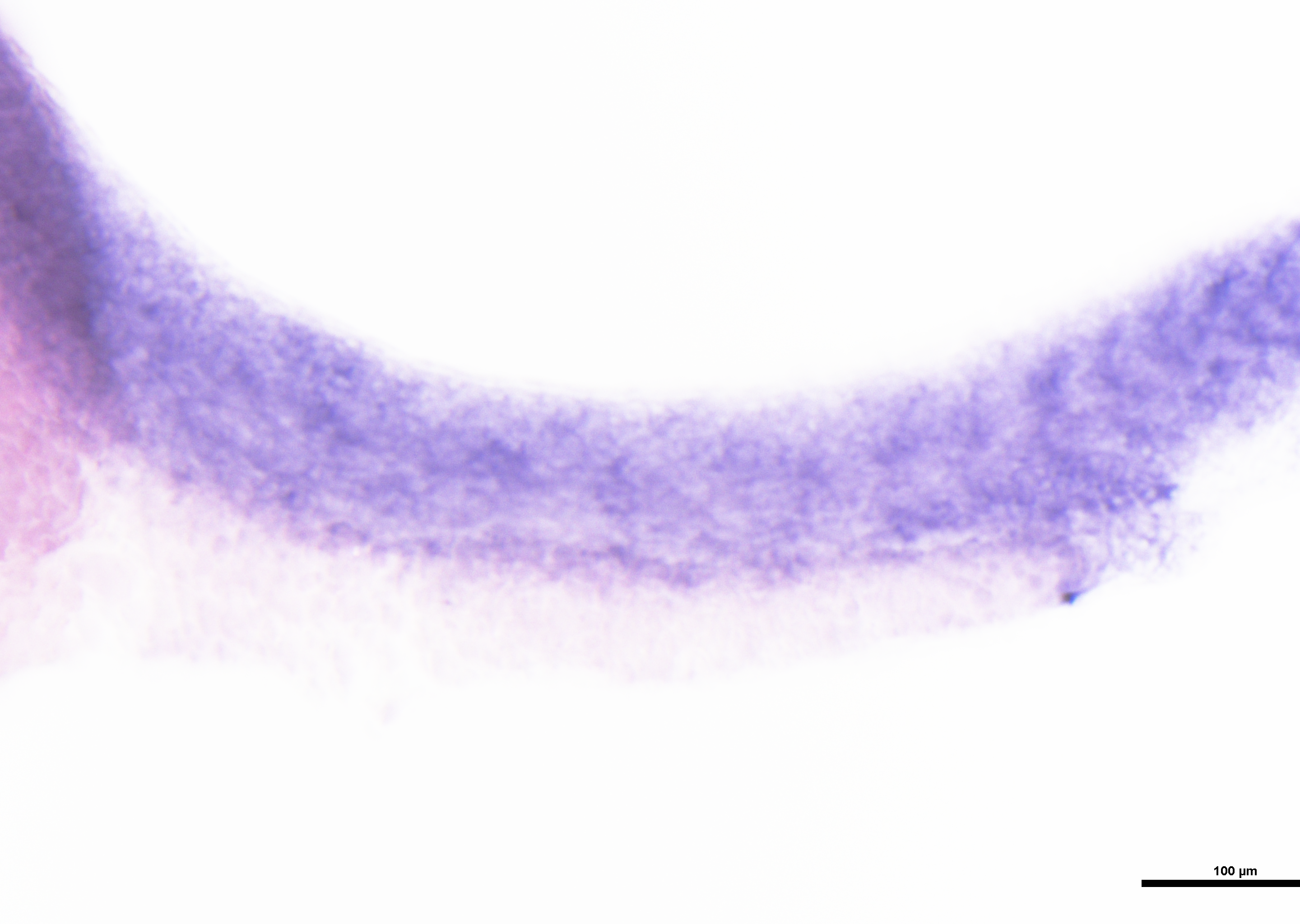

Supplement: Supplementary file 6 — Source data Fig. 1 [file 44319_2026_805_MOESM6_ESM.zip › Source Data Fig.1/Fig.1/L/4. runx1 36hpf Mtrmt61a;trmt61a-4bp.tif]

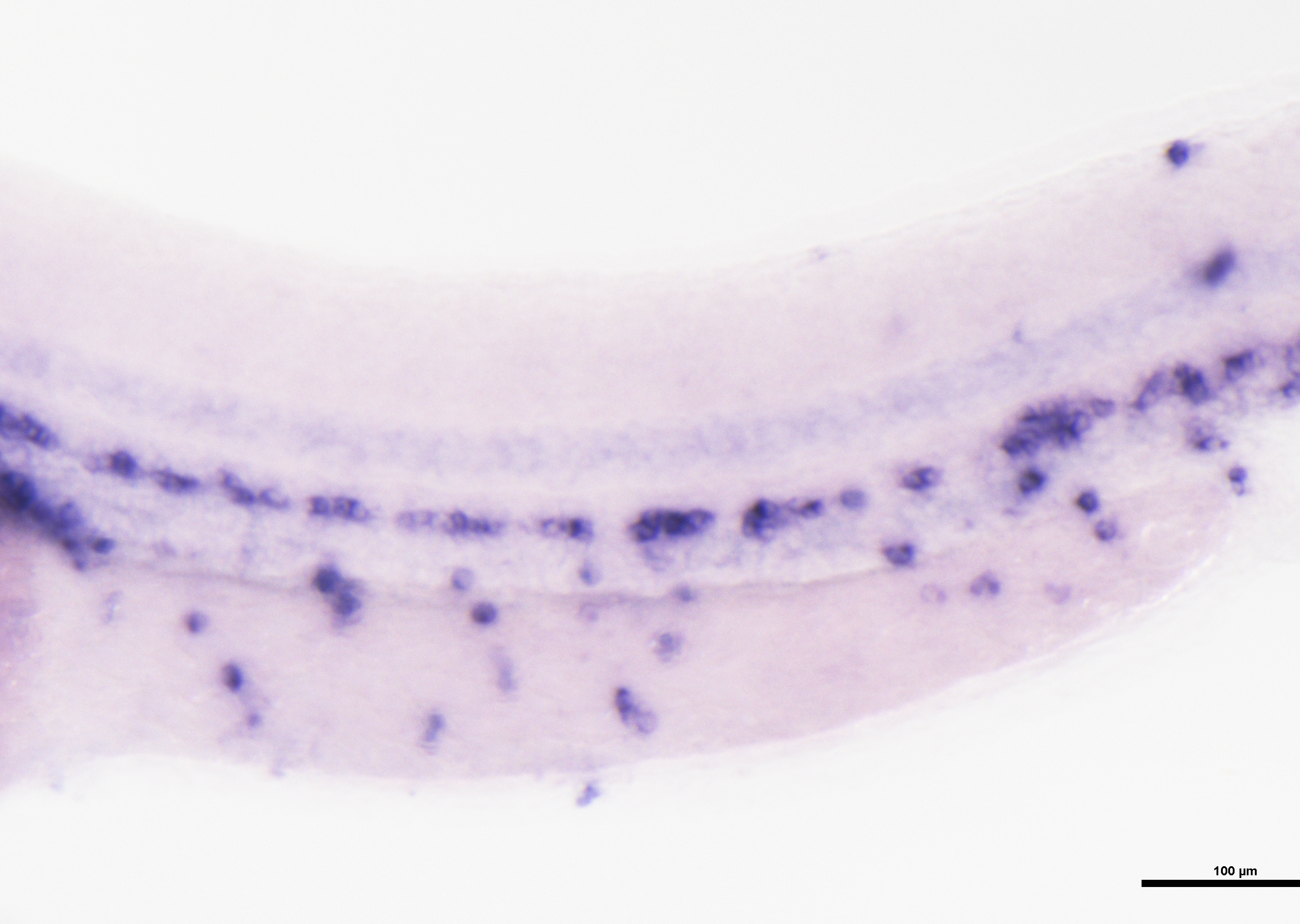

Supplement: Supplementary file 6 — Source data Fig. 1 [file 44319_2026_805_MOESM6_ESM.zip › Source Data Fig.1/Fig.1/L/5. cmyb 36hpf Mtrmt61a;trmt61a-4bp+trmt61amRNA.tif]

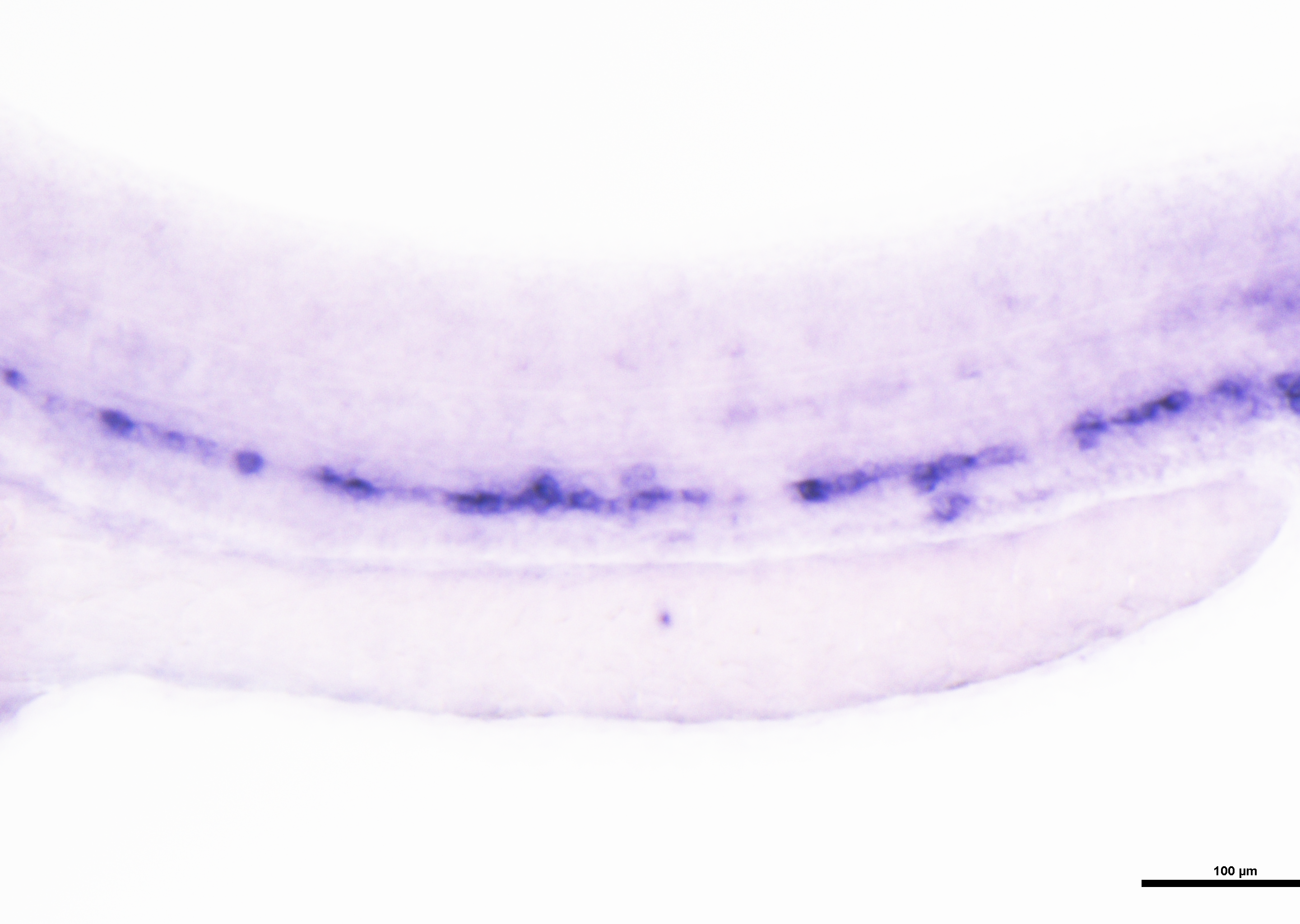

Supplement: Supplementary file 6 — Source data Fig. 1 [file 44319_2026_805_MOESM6_ESM.zip › Source Data Fig.1/Fig.1/L/6. runx1 36hpf Mtrmt61a;trmt61a-4bp+trmt61amRNA.tif]

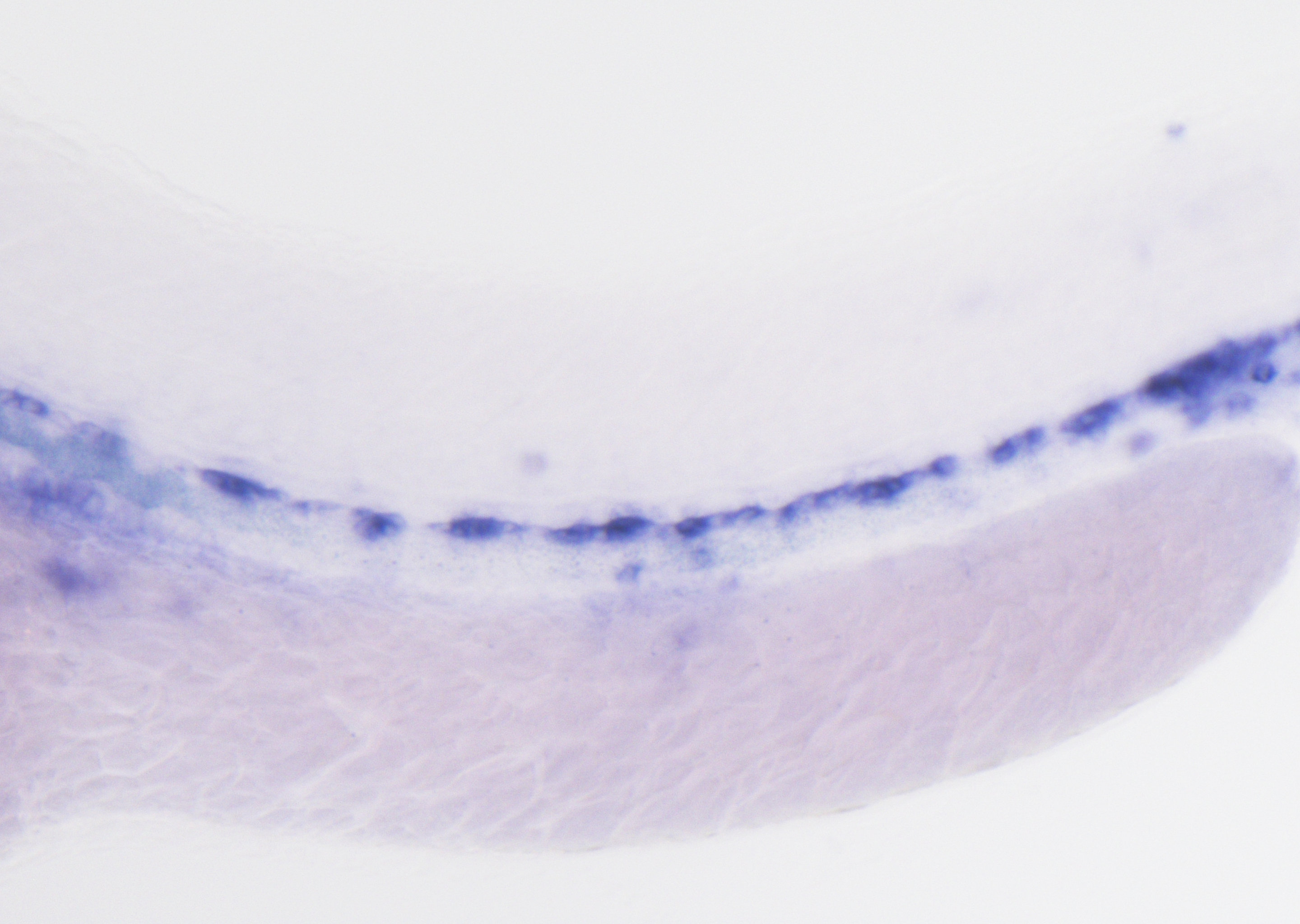

Supplement: Supplementary file 7 — Source data Fig. 2 [file 44319_2026_805_MOESM7_ESM.zip › Source Data Fig.2/Fig.2/B/1. cmyb 36hpf WT.tif]

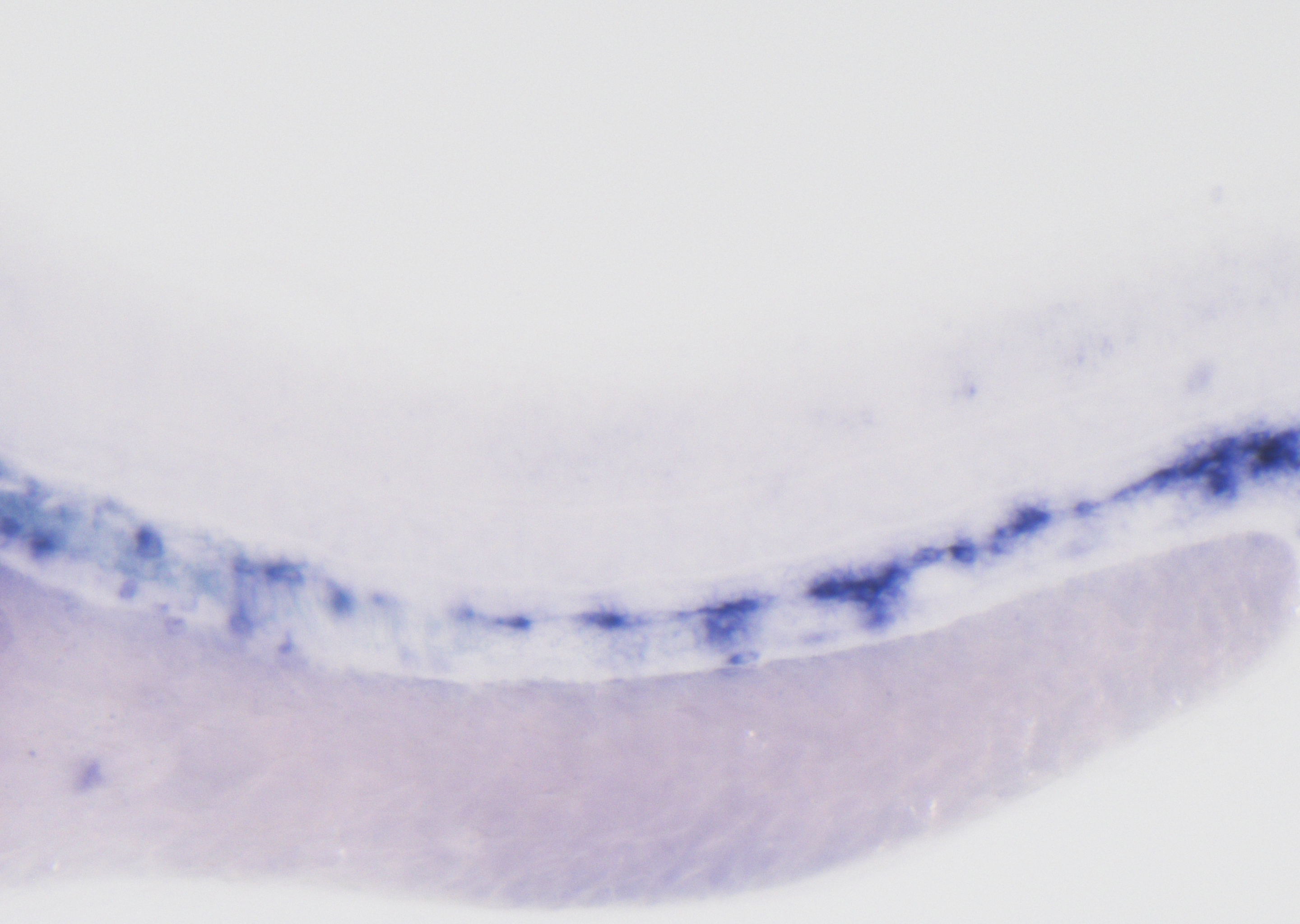

Supplement: Supplementary file 7 — Source data Fig. 2 [file 44319_2026_805_MOESM7_ESM.zip › Source Data Fig.2/Fig.2/B/2. cmyb 36hpf trmt61aD181AD181A.tif]

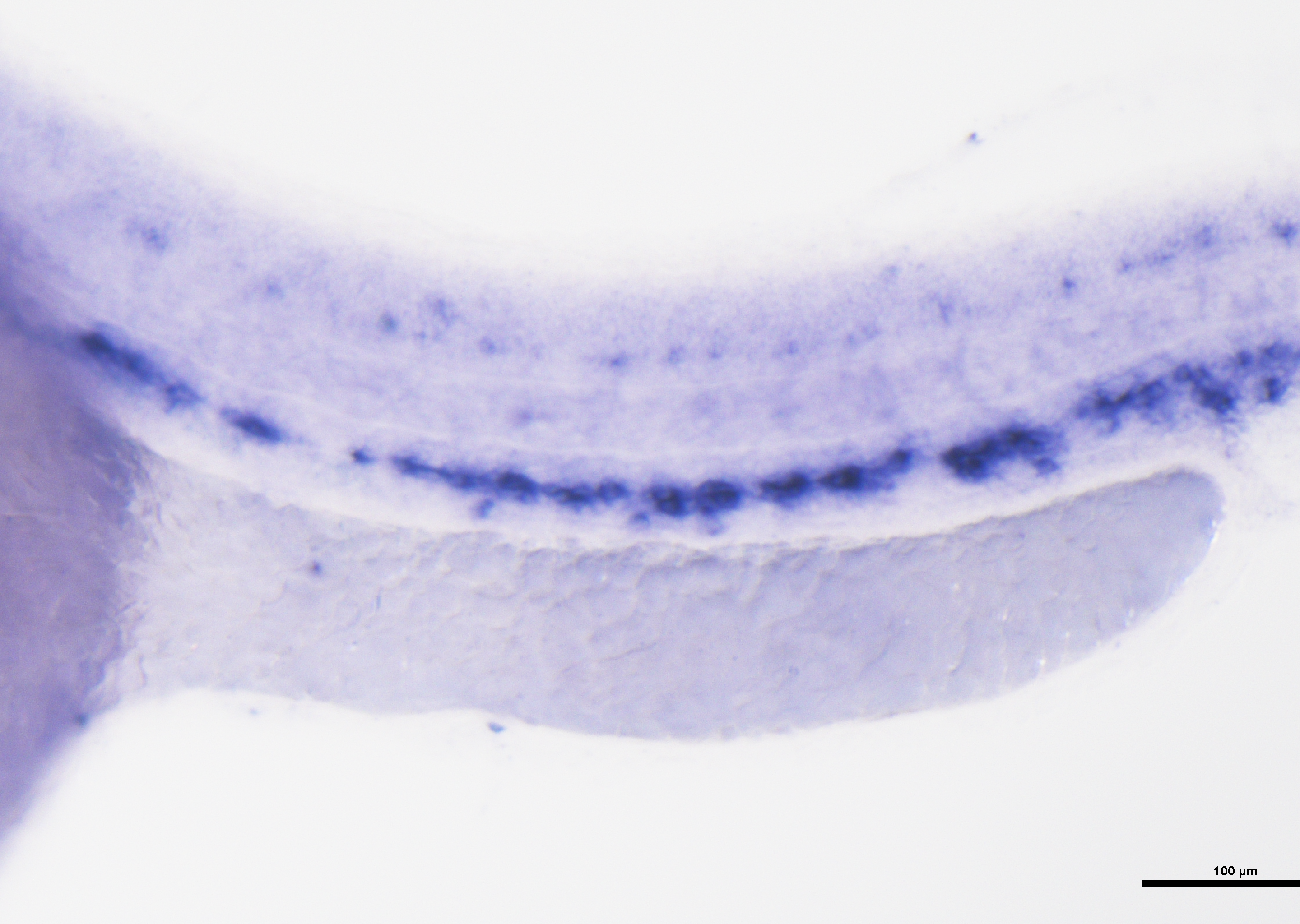

Supplement: Supplementary file 7 — Source data Fig. 2 [file 44319_2026_805_MOESM7_ESM.zip › Source Data Fig.2/Fig.2/B/3. runx1 36hpf WT.tif]

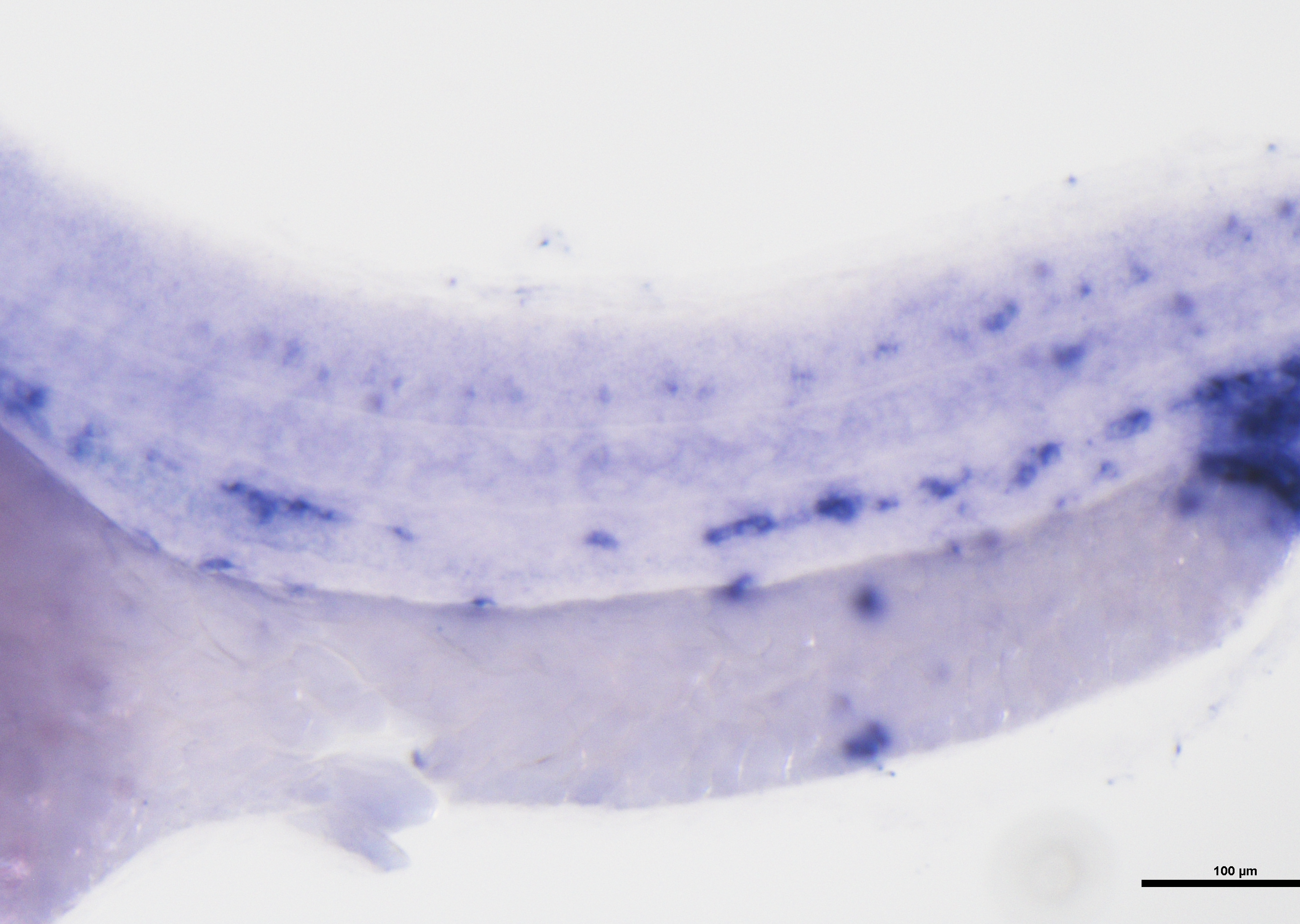

Supplement: Supplementary file 7 — Source data Fig. 2 [file 44319_2026_805_MOESM7_ESM.zip › Source Data Fig.2/Fig.2/B/4. runx1 36hpf trmt61aD181AD181A.tif]

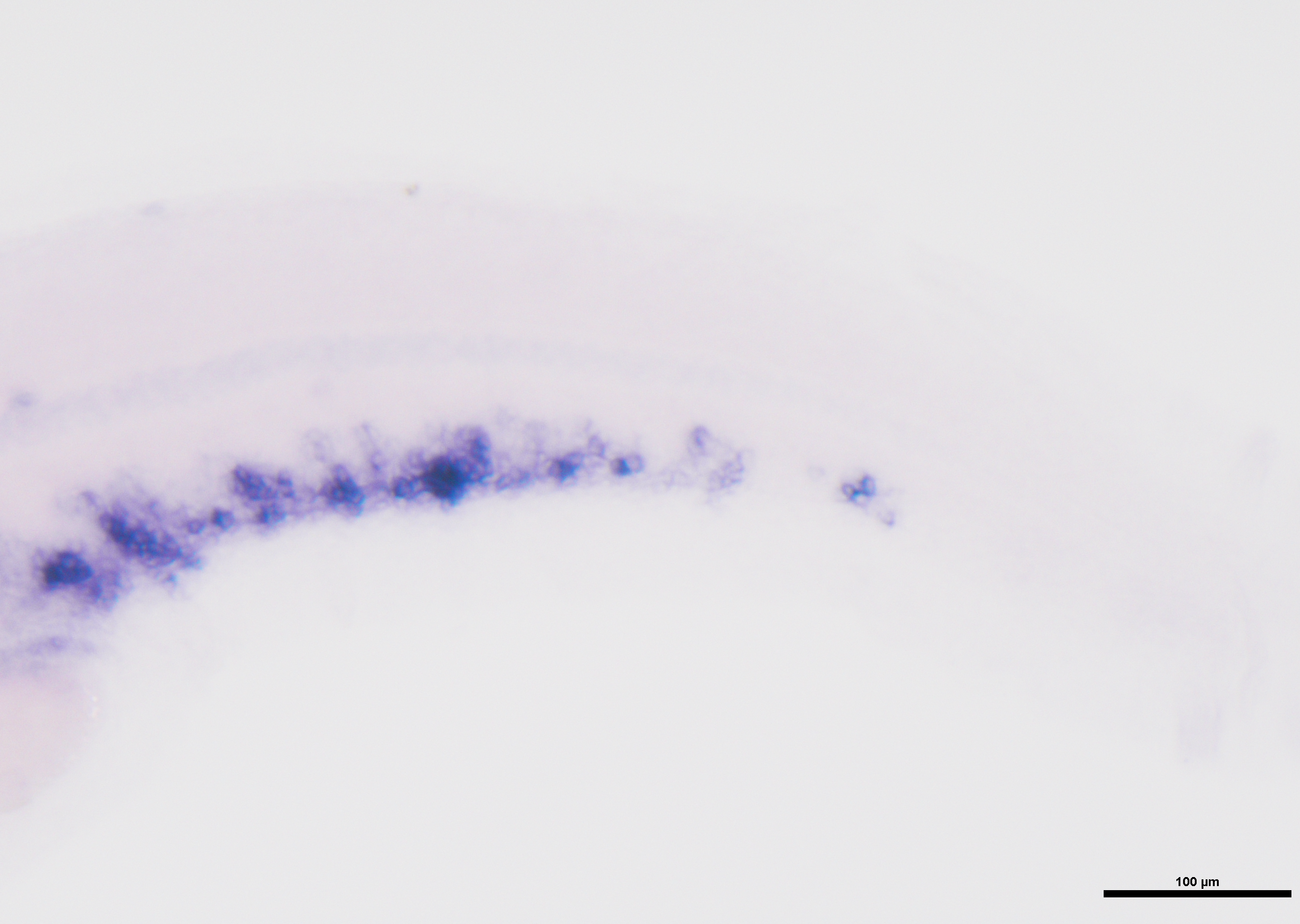

Supplement: Supplementary file 7 — Source data Fig. 2 [file 44319_2026_805_MOESM7_ESM.zip › Source Data Fig.2/Fig.2/B/5. cmyb 2dpf WT.tif]

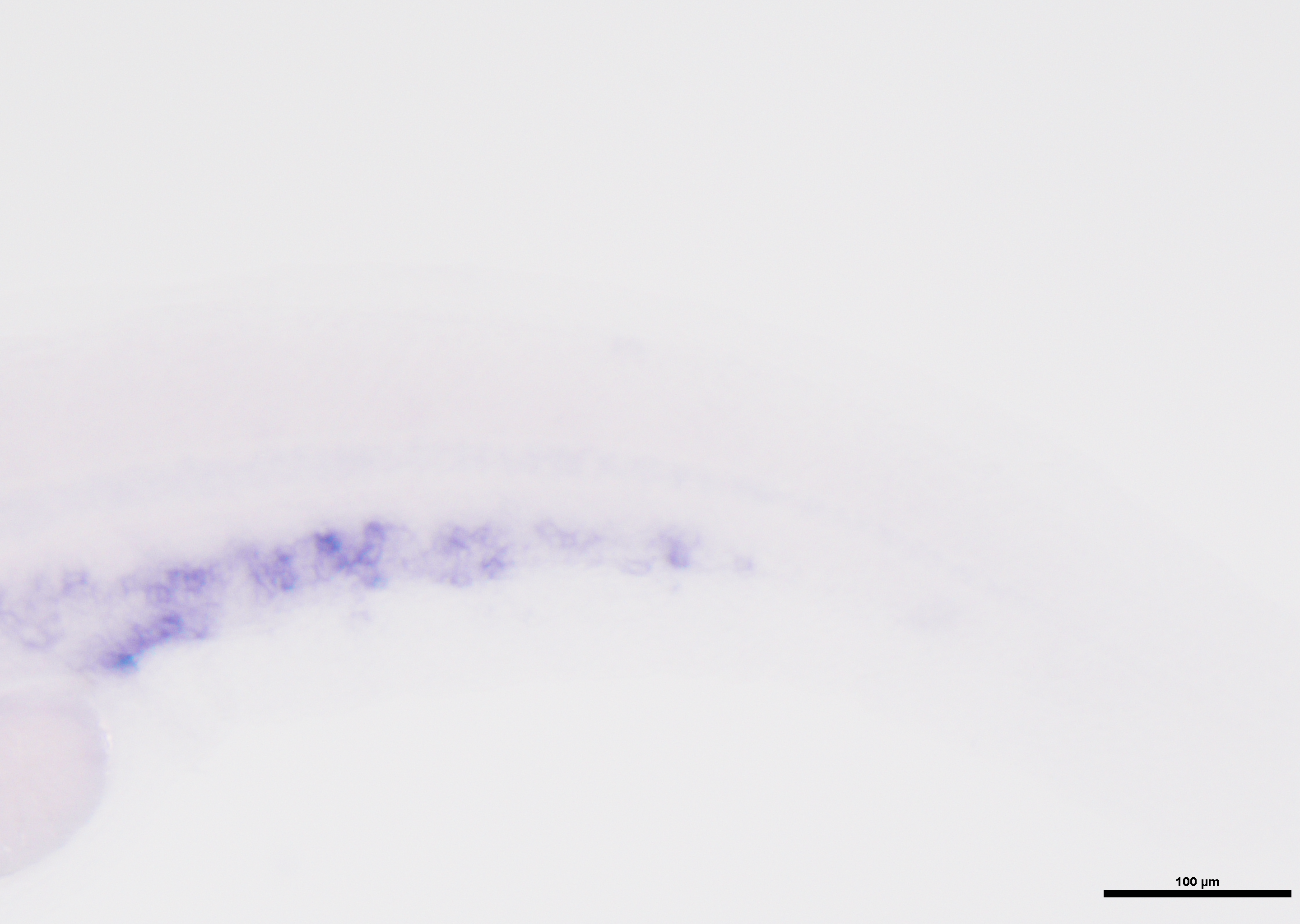

Supplement: Supplementary file 7 — Source data Fig. 2 [file 44319_2026_805_MOESM7_ESM.zip › Source Data Fig.2/Fig.2/B/6. cmyb 2dpf trmt61aD181AD181A.tif]

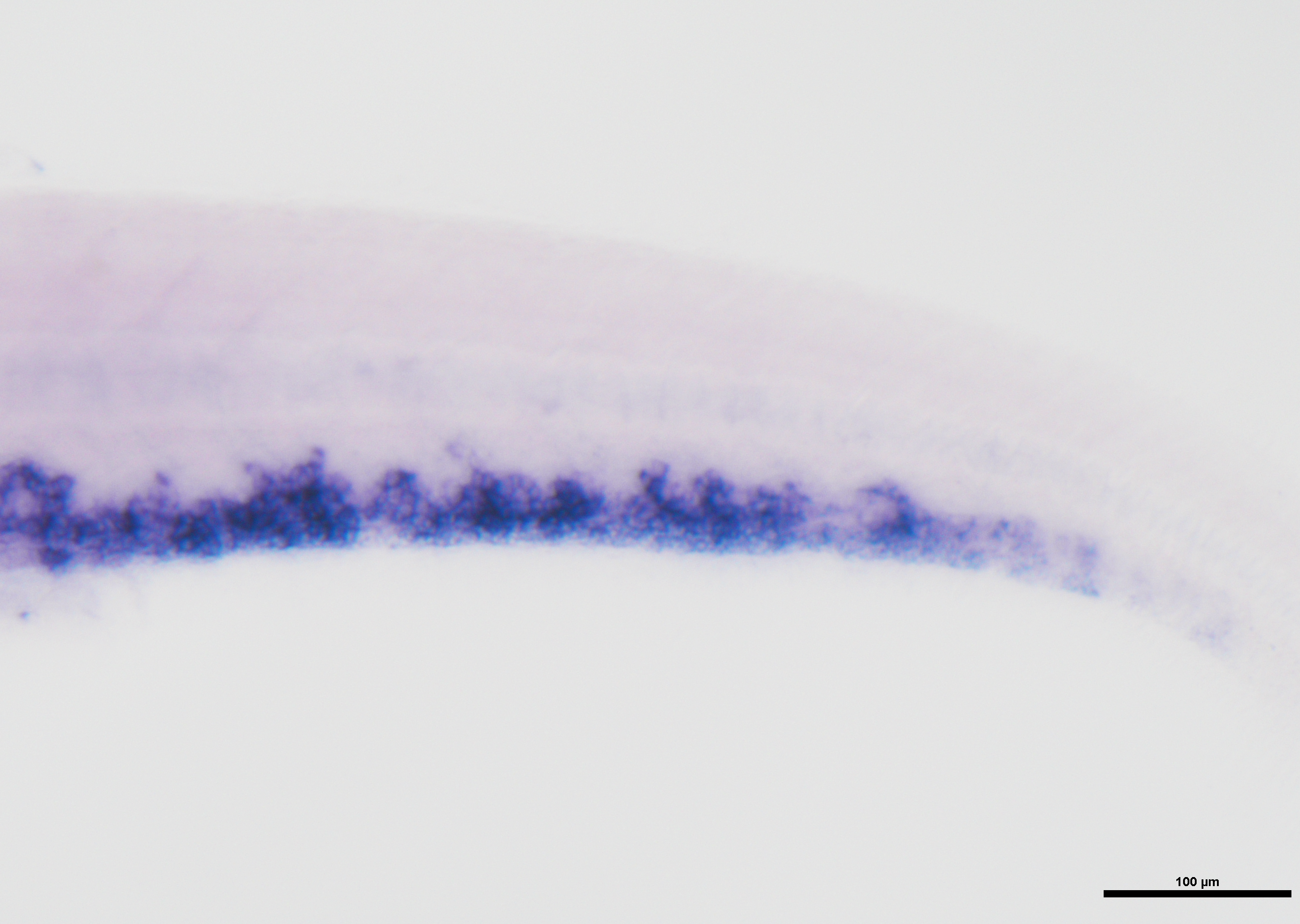

Supplement: Supplementary file 7 — Source data Fig. 2 [file 44319_2026_805_MOESM7_ESM.zip › Source Data Fig.2/Fig.2/B/7. cmyb 5dpf WT.tif]

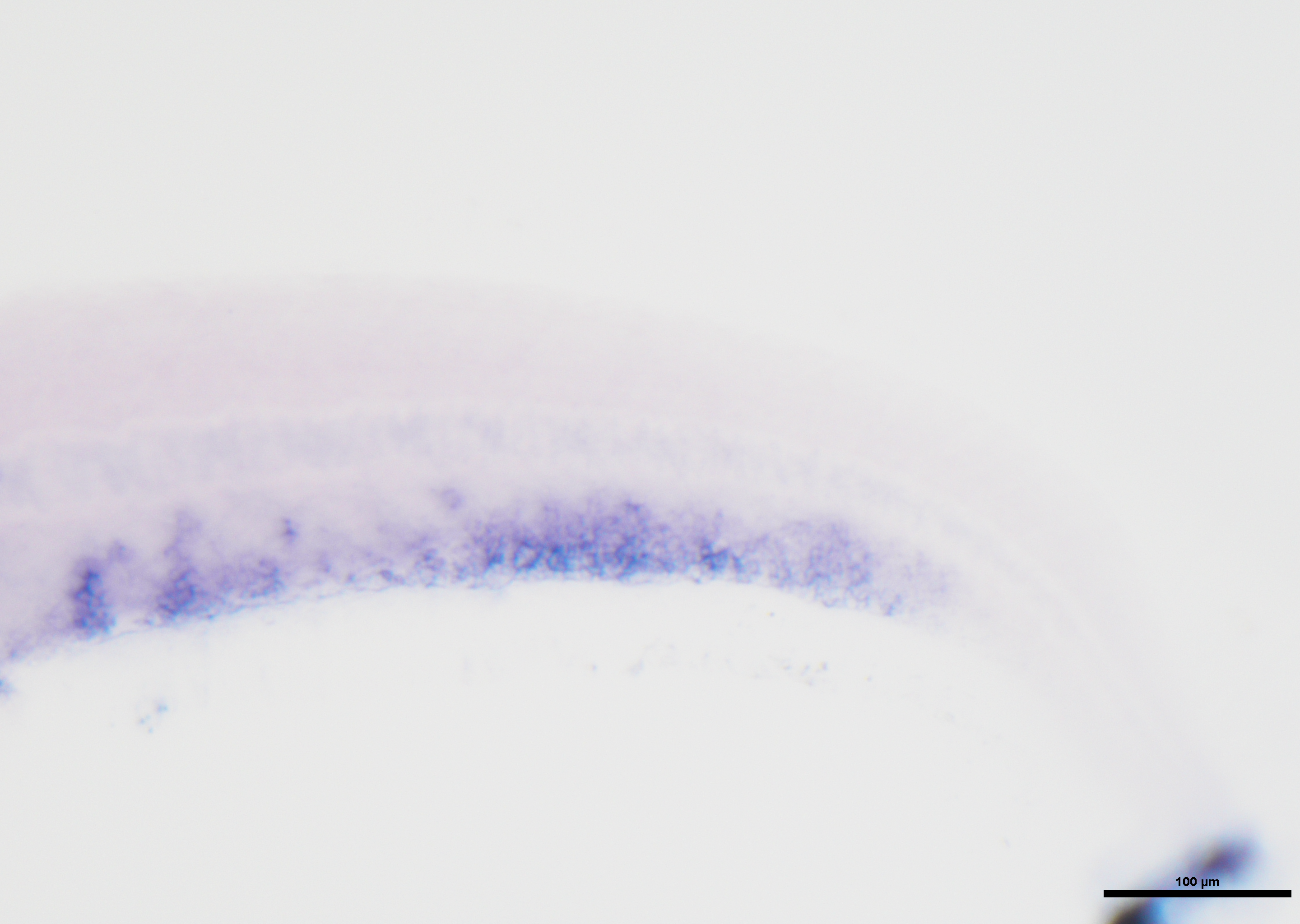

Supplement: Supplementary file 7 — Source data Fig. 2 [file 44319_2026_805_MOESM7_ESM.zip › Source Data Fig.2/Fig.2/B/8. cmyb 5dpf trmt61aD181AD181A.tif]

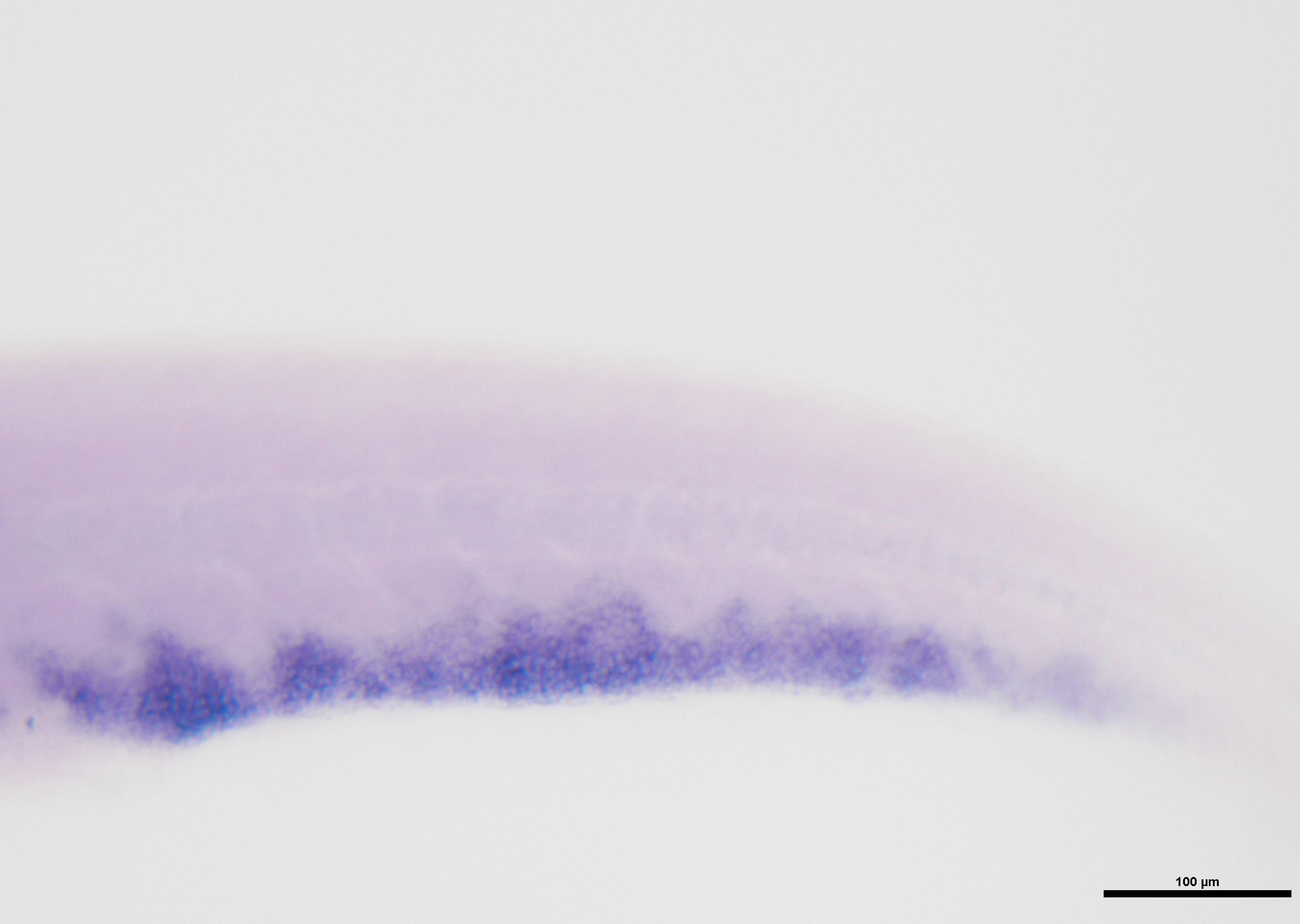

Supplement: Supplementary file 7 — Source data Fig. 2 [file 44319_2026_805_MOESM7_ESM.zip › Source Data Fig.2/Fig.2/D/1. gata1a 5dpf WT.tif]

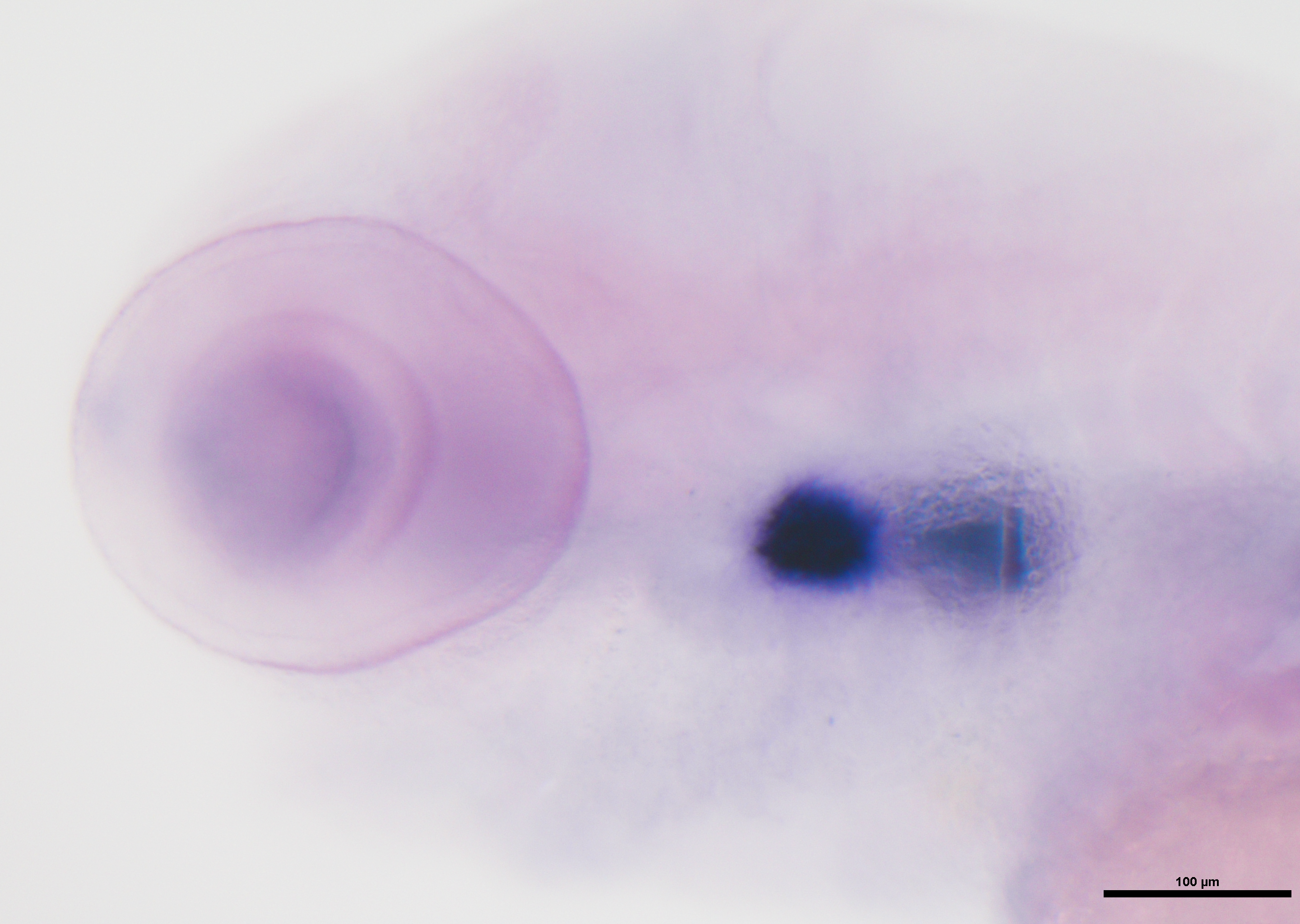

Supplement: Supplementary file 7 — Source data Fig. 2 [file 44319_2026_805_MOESM7_ESM.zip › Source Data Fig.2/Fig.2/D/10. rag1 5dpf WT.tif]

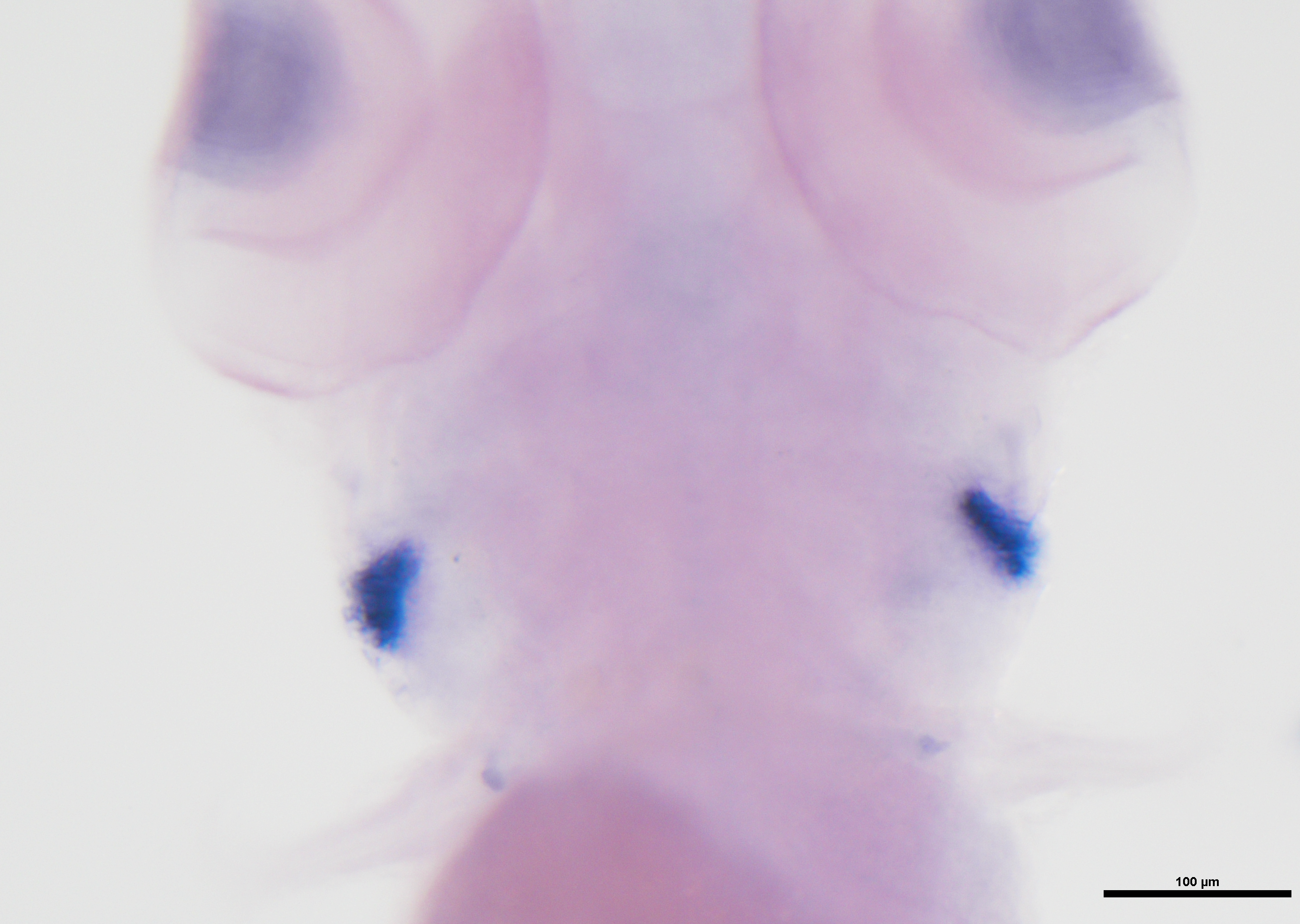

Supplement: Supplementary file 7 — Source data Fig. 2 [file 44319_2026_805_MOESM7_ESM.zip › Source Data Fig.2/Fig.2/D/11. rag1 5dpf trmt61aD181AD181A.tif]

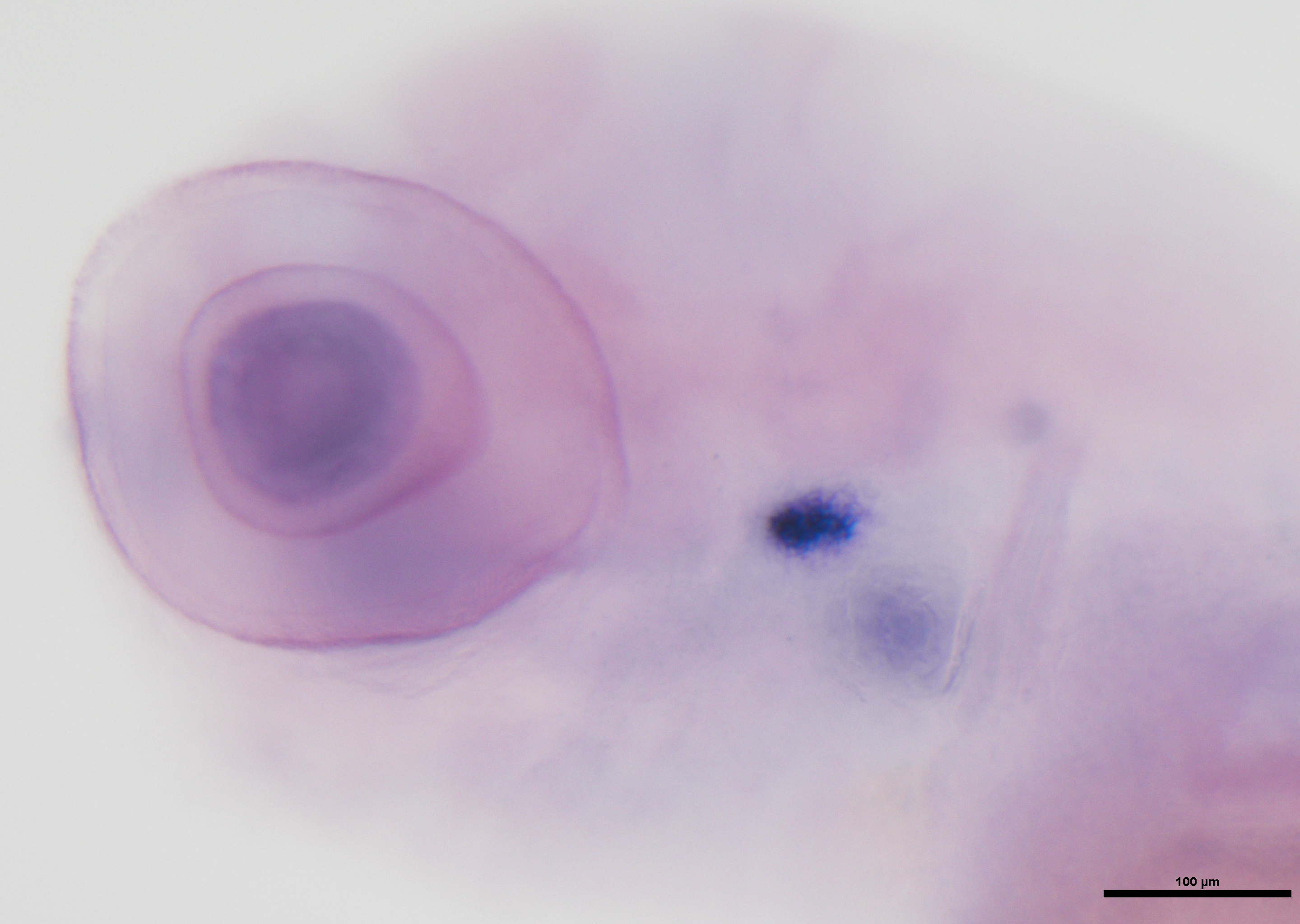

Supplement: Supplementary file 7 — Source data Fig. 2 [file 44319_2026_805_MOESM7_ESM.zip › Source Data Fig.2/Fig.2/D/12. rag1 5dpf trmt61aD181AD181A.tif]

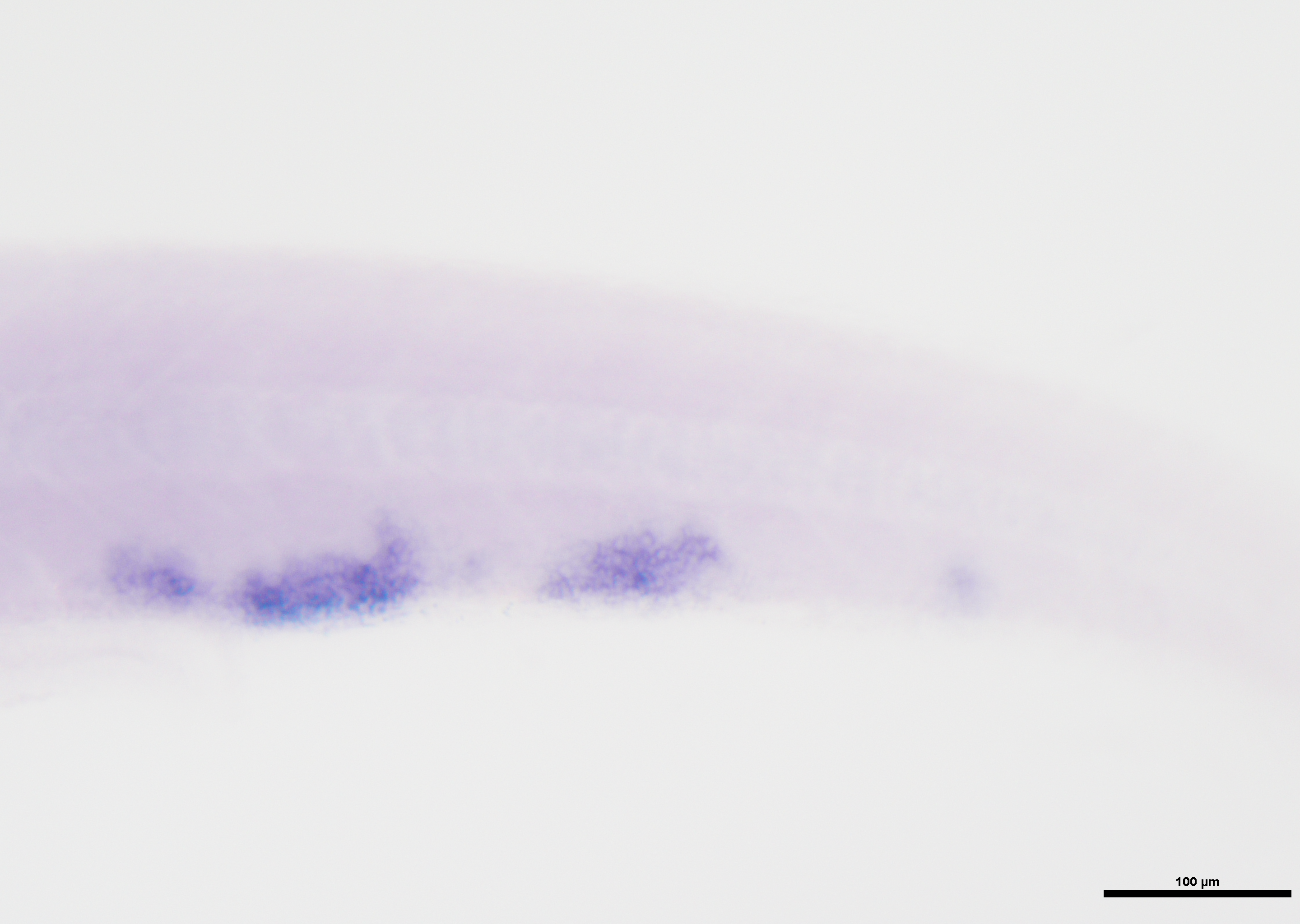

Supplement: Supplementary file 7 — Source data Fig. 2 [file 44319_2026_805_MOESM7_ESM.zip › Source Data Fig.2/Fig.2/D/2. gata1a 5dpf trmt61aD181AD181A.tif]

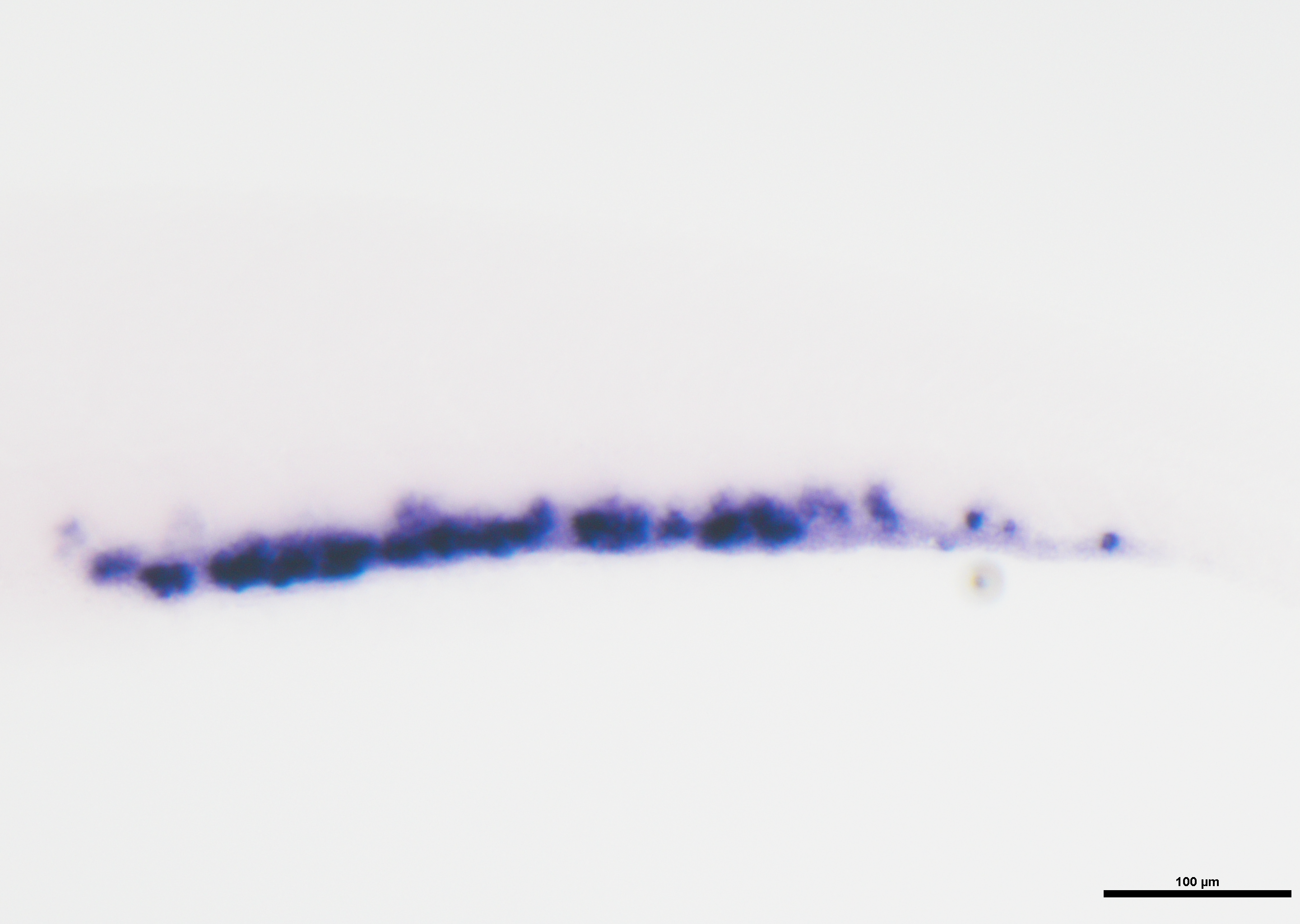

Supplement: Supplementary file 7 — Source data Fig. 2 [file 44319_2026_805_MOESM7_ESM.zip › Source Data Fig.2/Fig.2/D/3. hbae1.1 5dpf WT.tif]

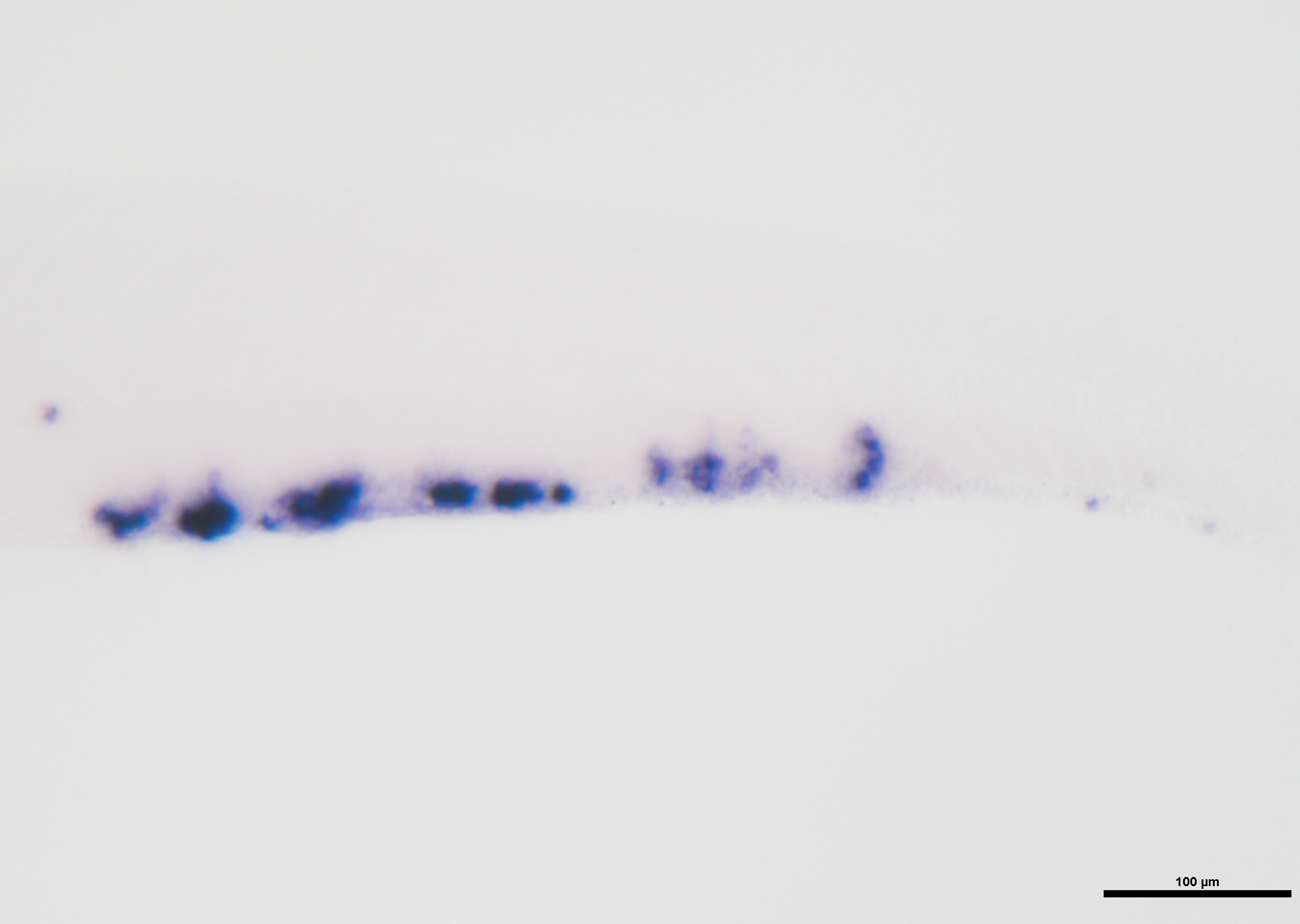

Supplement: Supplementary file 7 — Source data Fig. 2 [file 44319_2026_805_MOESM7_ESM.zip › Source Data Fig.2/Fig.2/D/4. hbae1.1 5dpf trmt61aD181AD181A.tif]

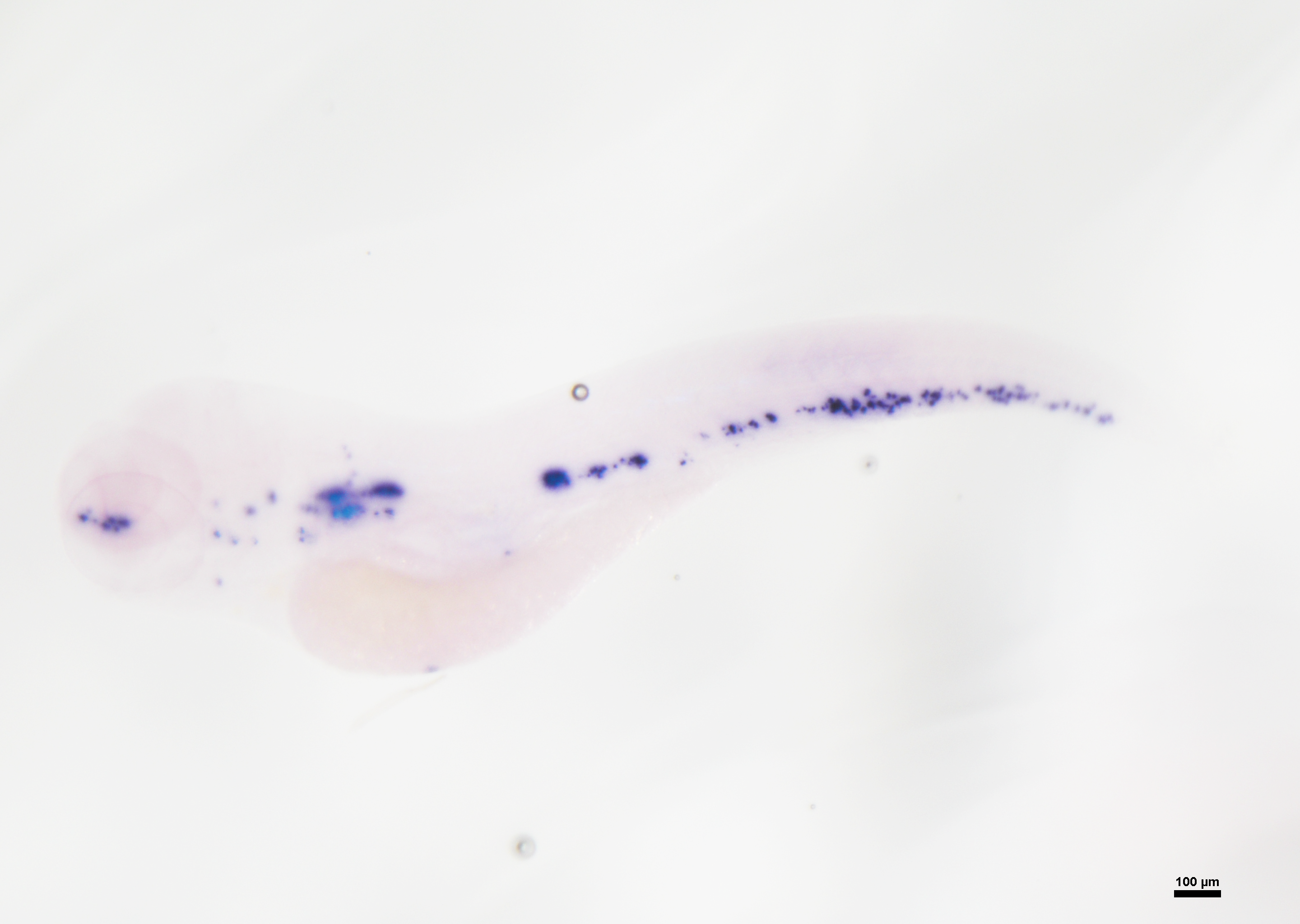

Supplement: Supplementary file 7 — Source data Fig. 2 [file 44319_2026_805_MOESM7_ESM.zip › Source Data Fig.2/Fig.2/D/5. lyz 5dpf WT.tif]

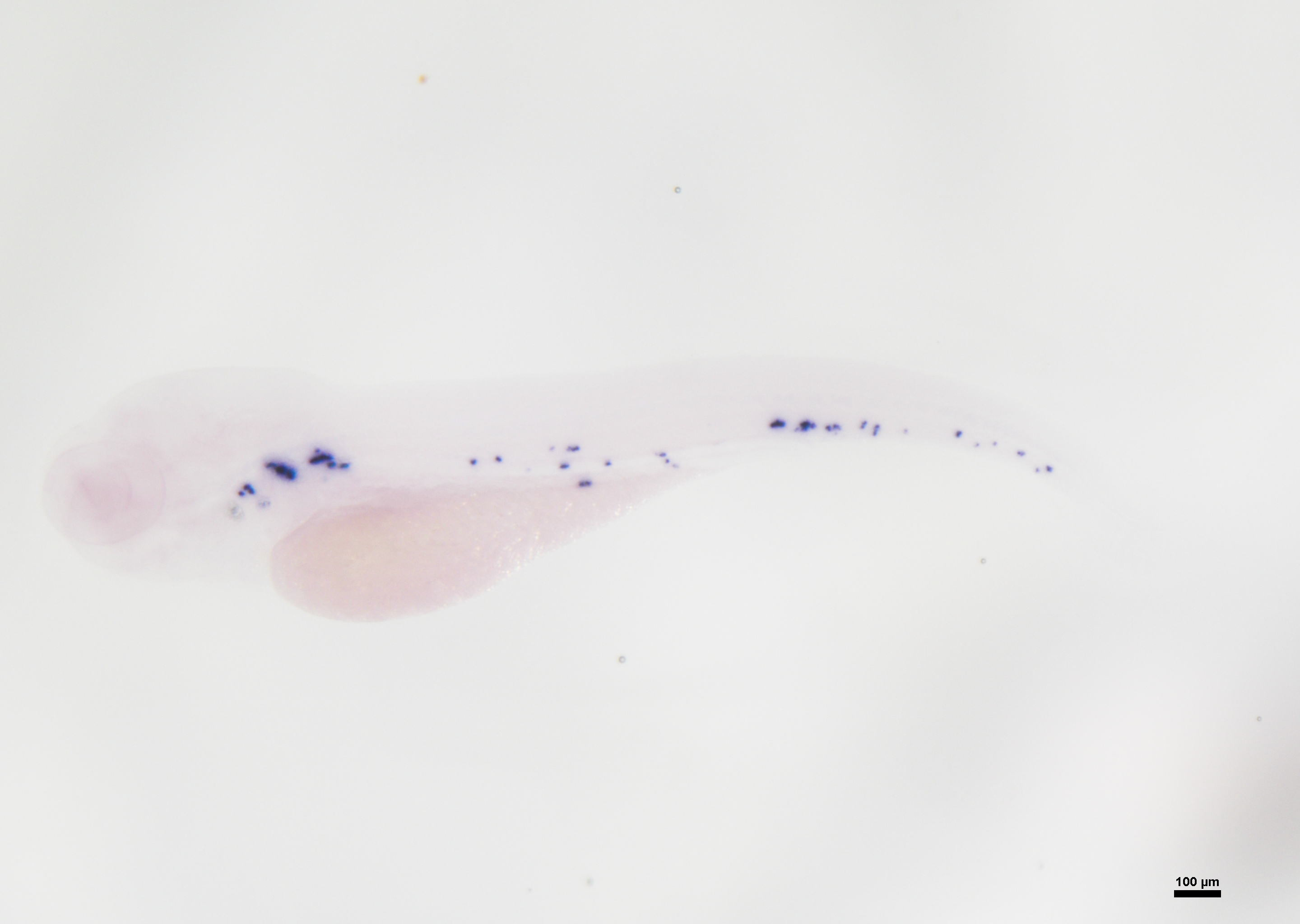

Supplement: Supplementary file 7 — Source data Fig. 2 [file 44319_2026_805_MOESM7_ESM.zip › Source Data Fig.2/Fig.2/D/6. lyz 5dpf trmt61aD181AD181A.tif]

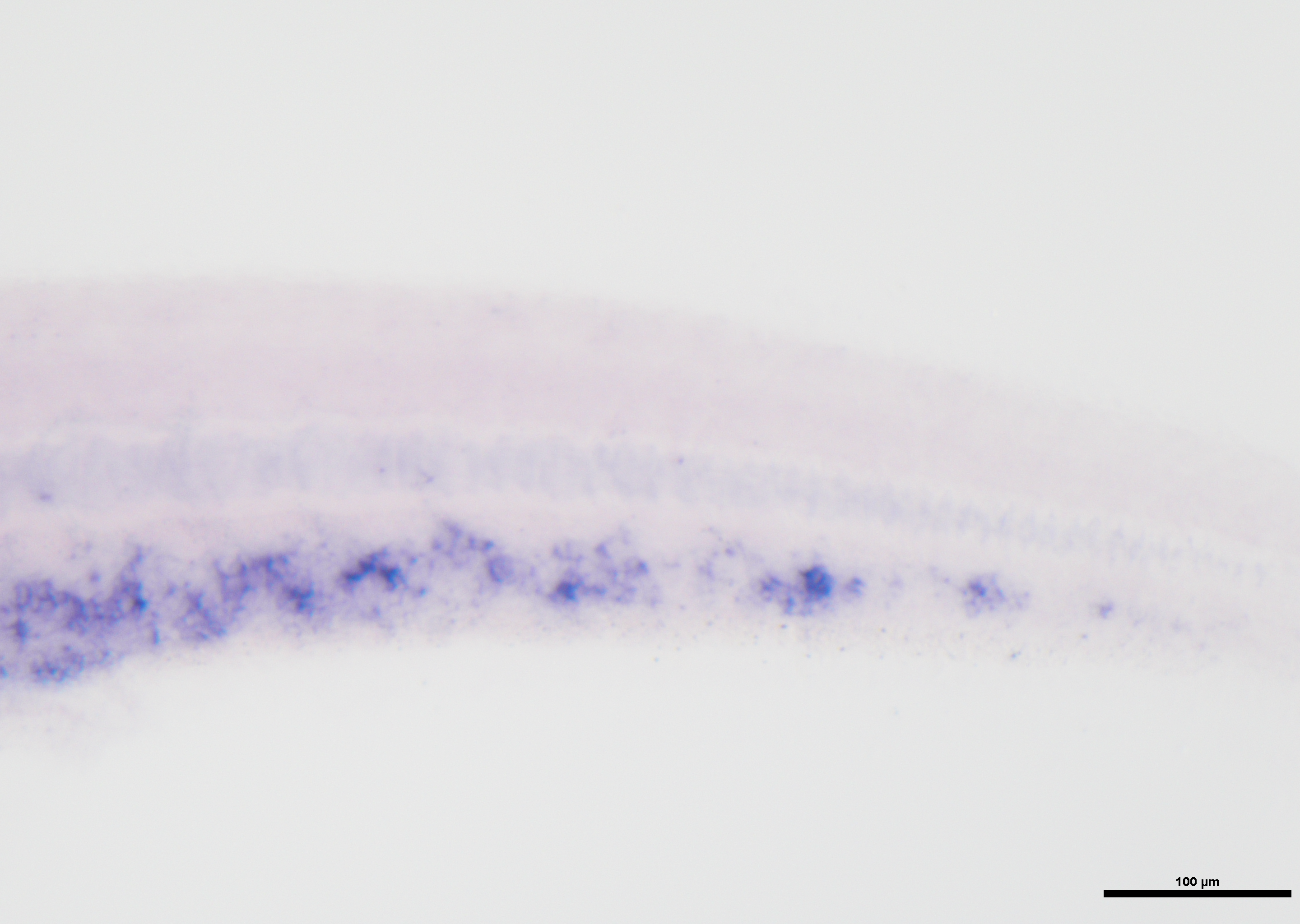

Supplement: Supplementary file 7 — Source data Fig. 2 [file 44319_2026_805_MOESM7_ESM.zip › Source Data Fig.2/Fig.2/D/7. pu.1 5dpf WT.tif]

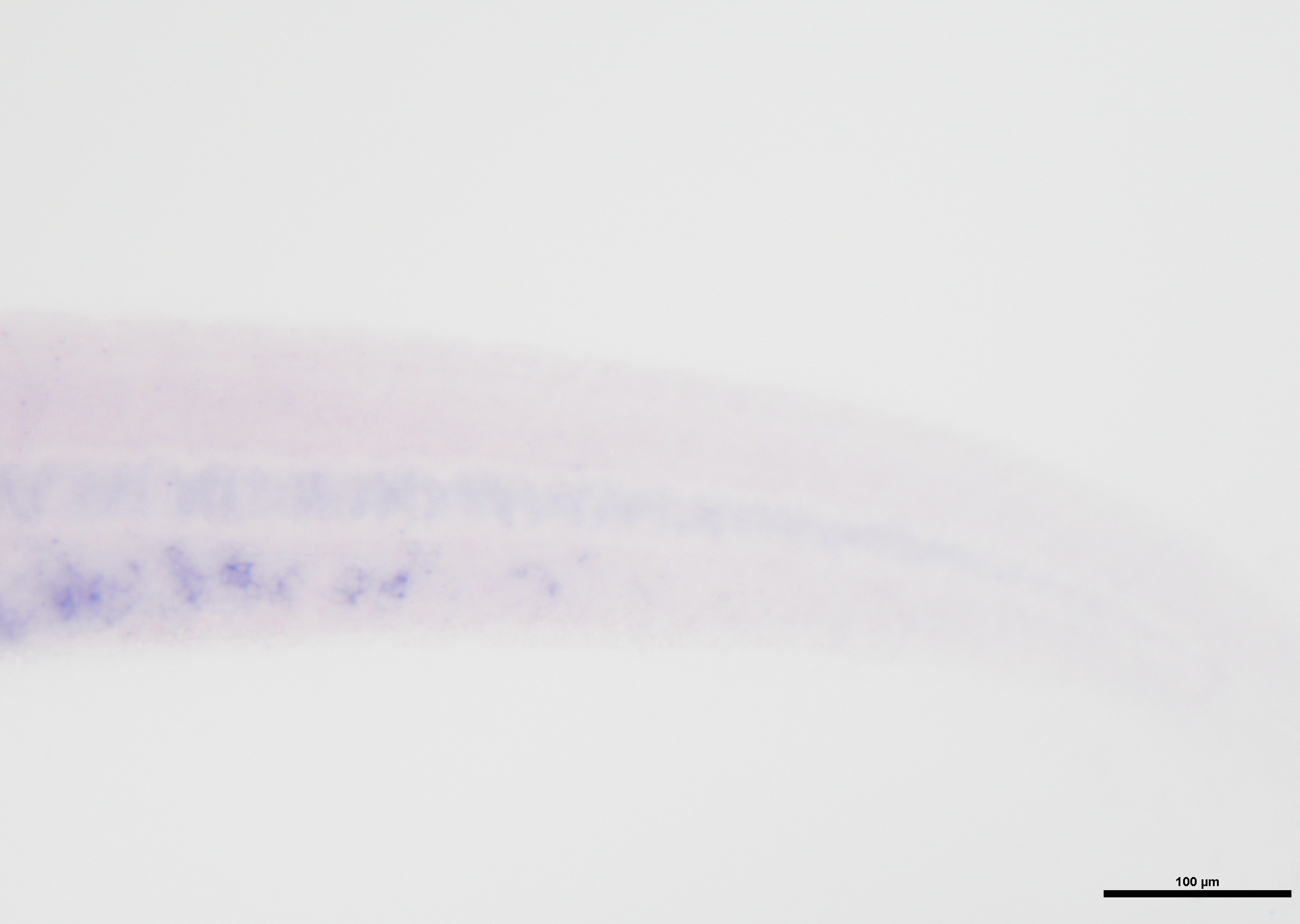

Supplement: Supplementary file 7 — Source data Fig. 2 [file 44319_2026_805_MOESM7_ESM.zip › Source Data Fig.2/Fig.2/D/8. pu.1 5dpf trmt61aD181AD181A.tif]

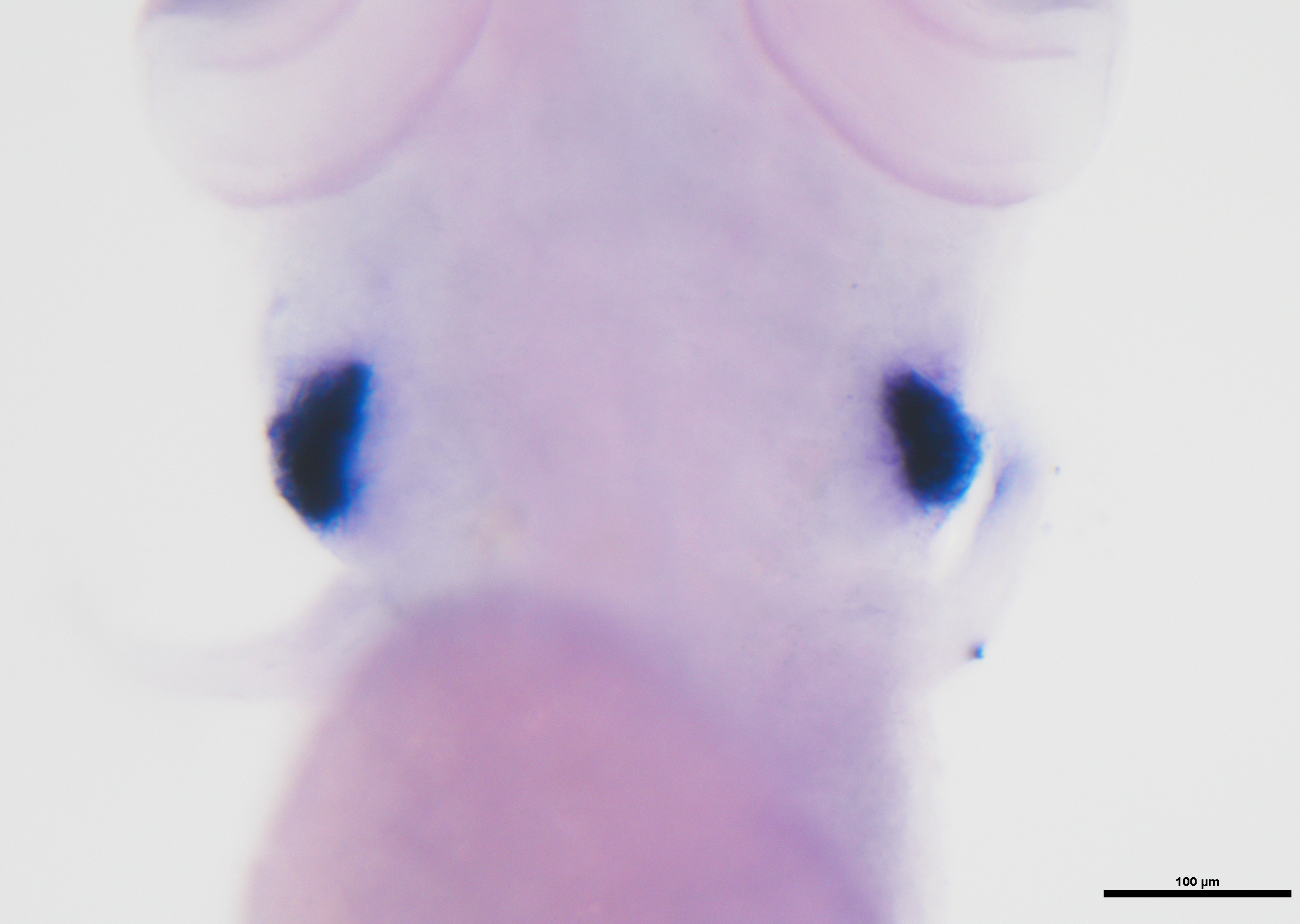

Supplement: Supplementary file 7 — Source data Fig. 2 [file 44319_2026_805_MOESM7_ESM.zip › Source Data Fig.2/Fig.2/D/9. rag1 5dpf WT.tif]

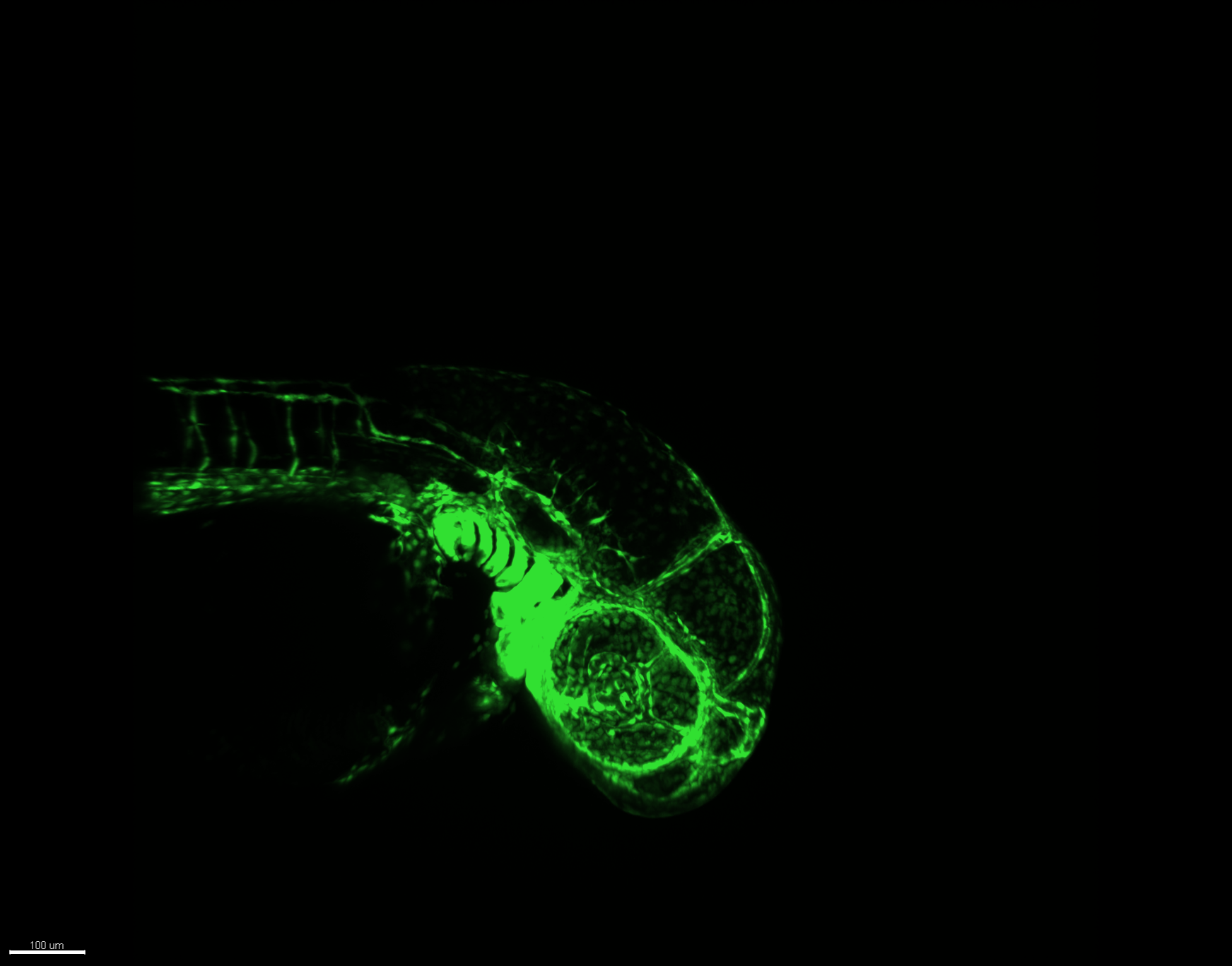

Supplement: Supplementary file 7 — Source data Fig. 2 [file 44319_2026_805_MOESM7_ESM.zip › Source Data Fig.2/Fig.2/F/1.1 36hpf fli1a trmt61a WT.tif]

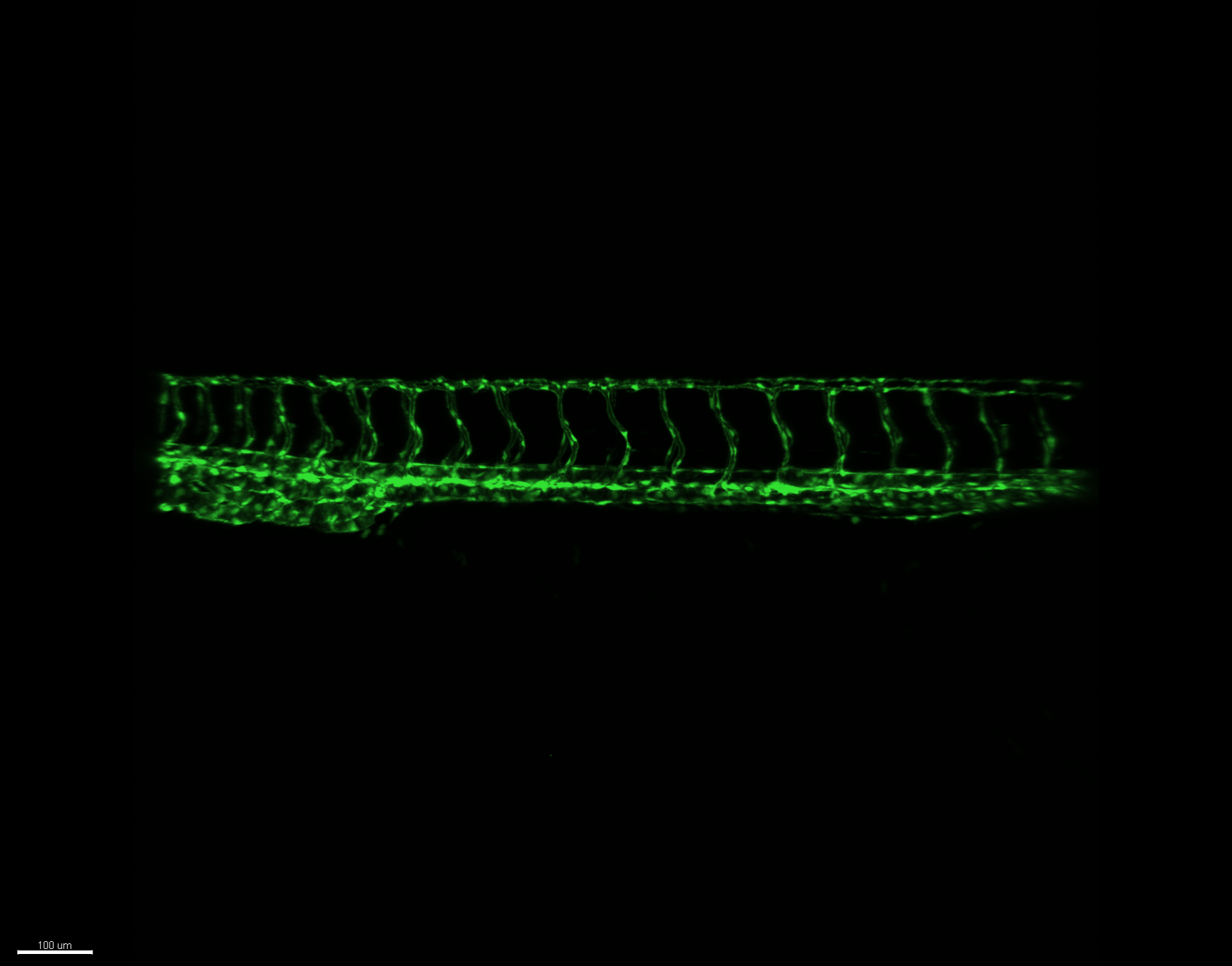

Supplement: Supplementary file 7 — Source data Fig. 2 [file 44319_2026_805_MOESM7_ESM.zip › Source Data Fig.2/Fig.2/F/1.2 36hpf fli1a trmt61a WT.tif]

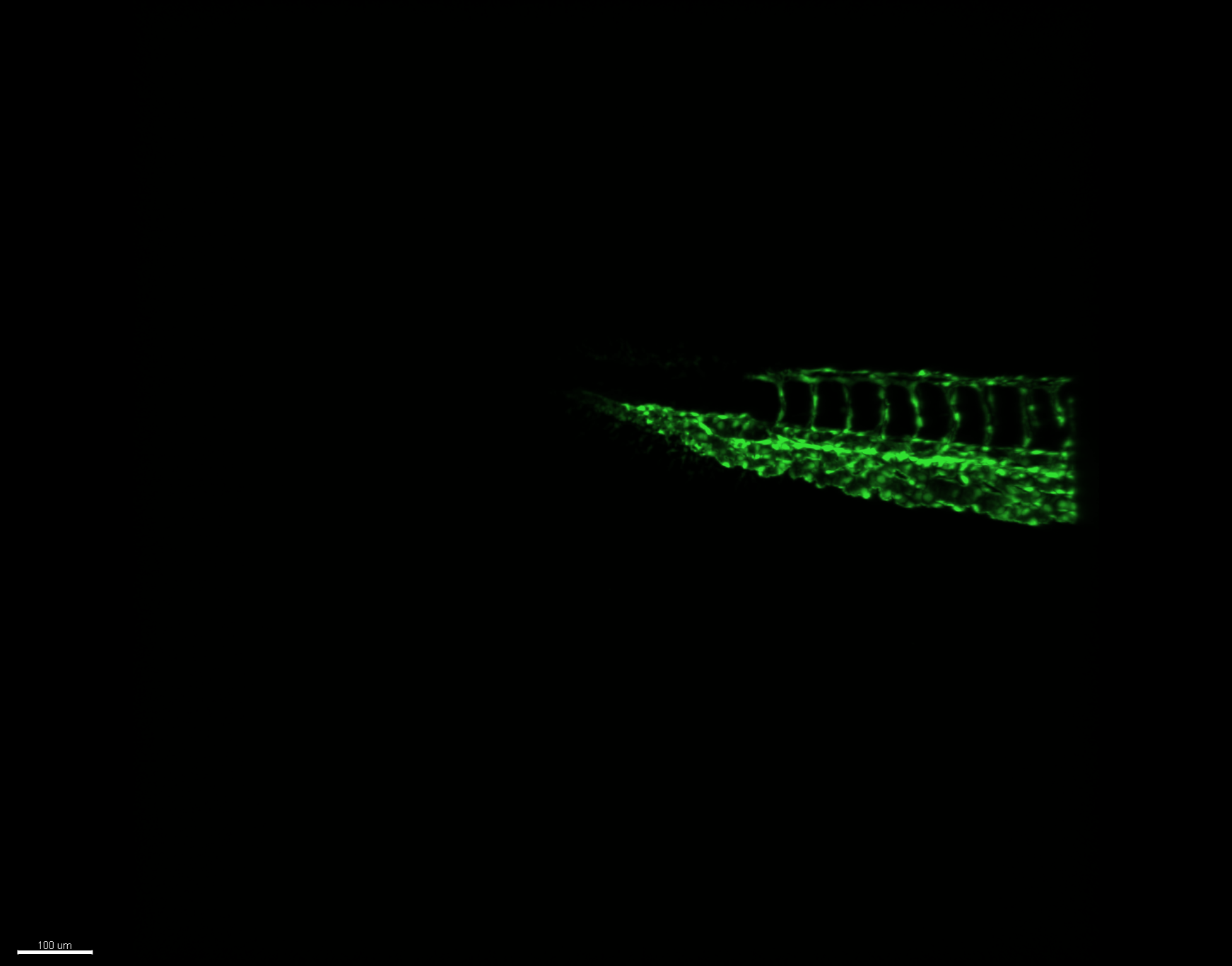

Supplement: Supplementary file 7 — Source data Fig. 2 [file 44319_2026_805_MOESM7_ESM.zip › Source Data Fig.2/Fig.2/F/1.3 36hpf fli1a trmt61a WT.tif]

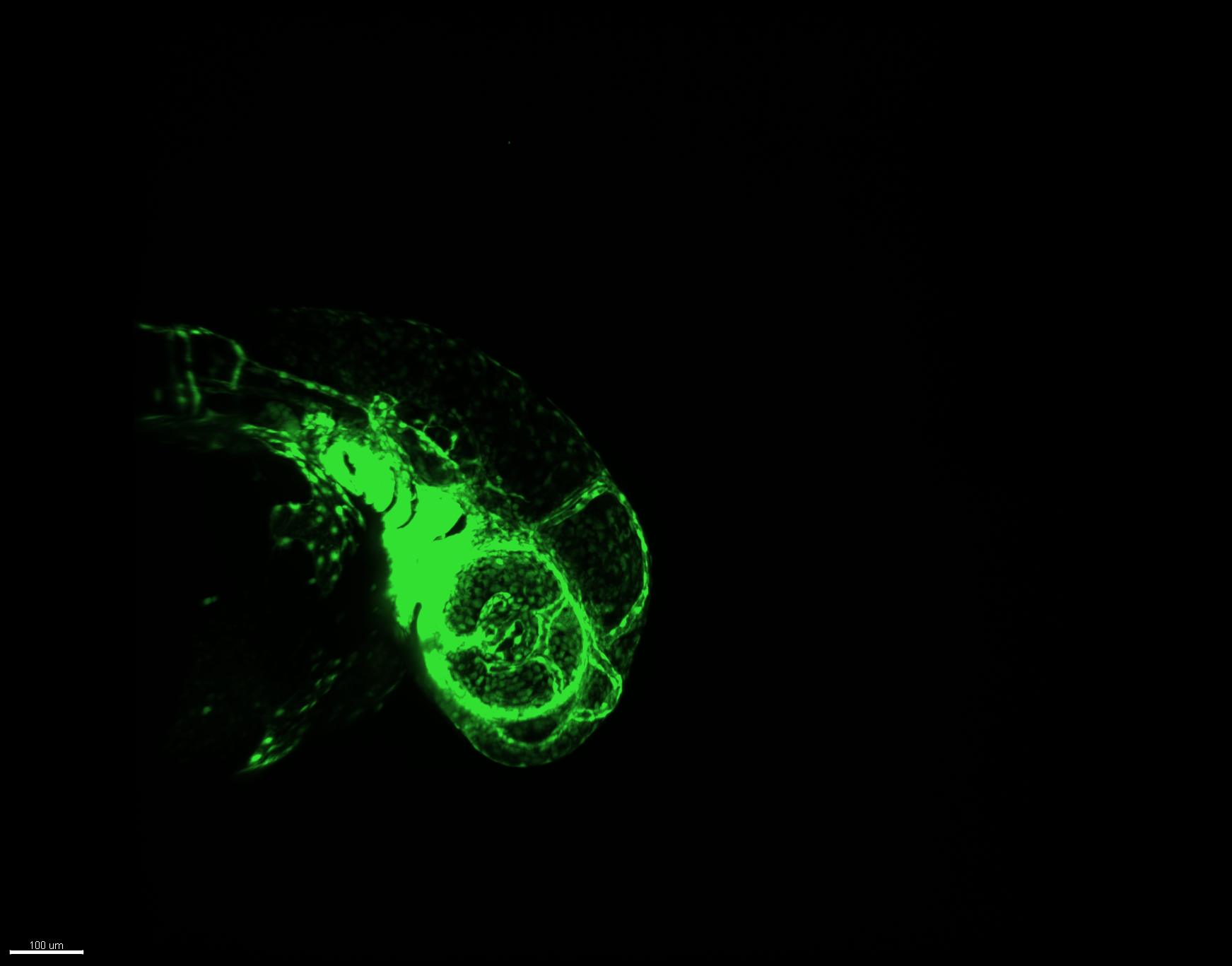

Supplement: Supplementary file 7 — Source data Fig. 2 [file 44319_2026_805_MOESM7_ESM.zip › Source Data Fig.2/Fig.2/F/2.1 36hpf fli1a trmt61a D181A.tif]

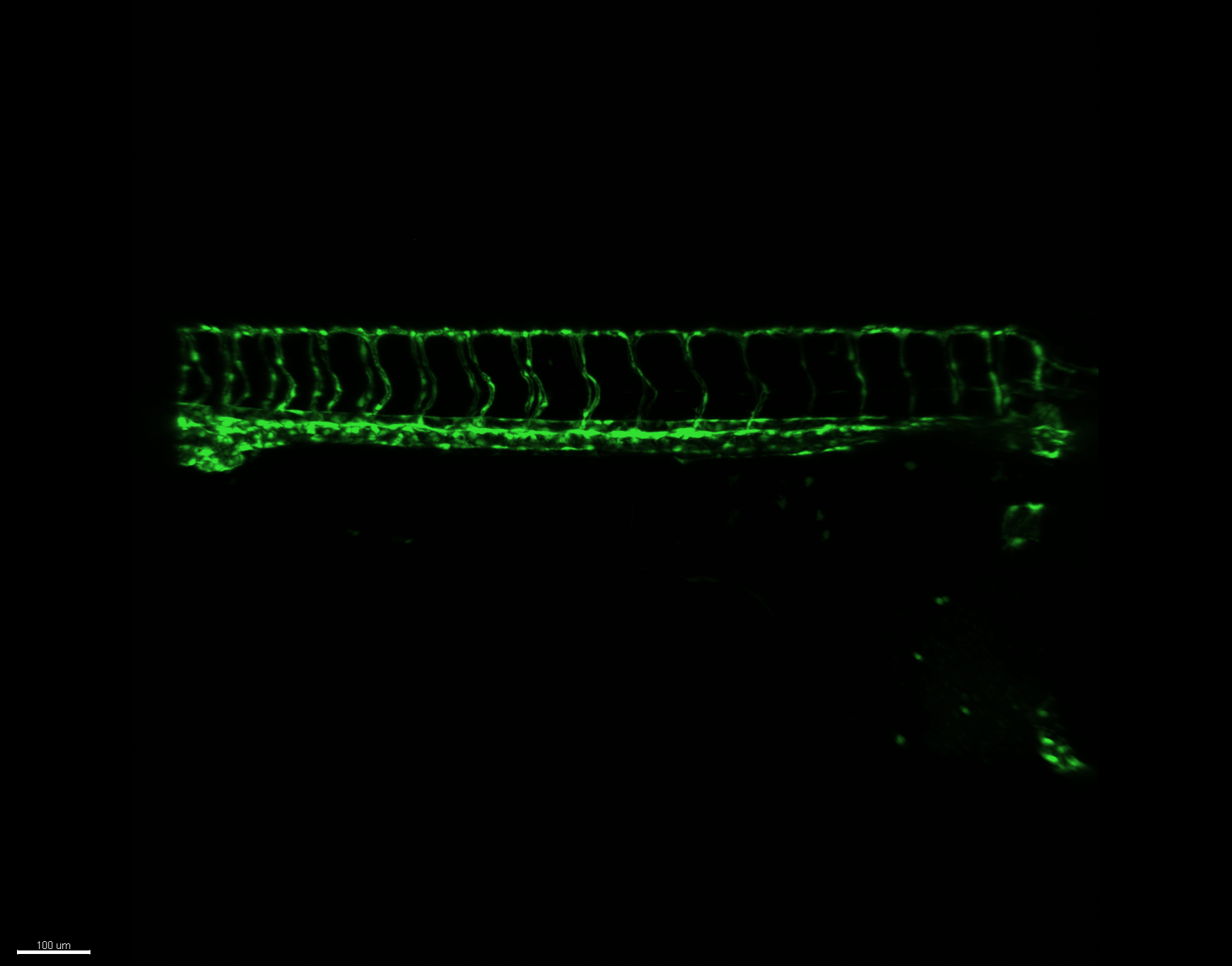

Supplement: Supplementary file 7 — Source data Fig. 2 [file 44319_2026_805_MOESM7_ESM.zip › Source Data Fig.2/Fig.2/F/2.2 36hpf fli1a trmt61a D181A.tif]

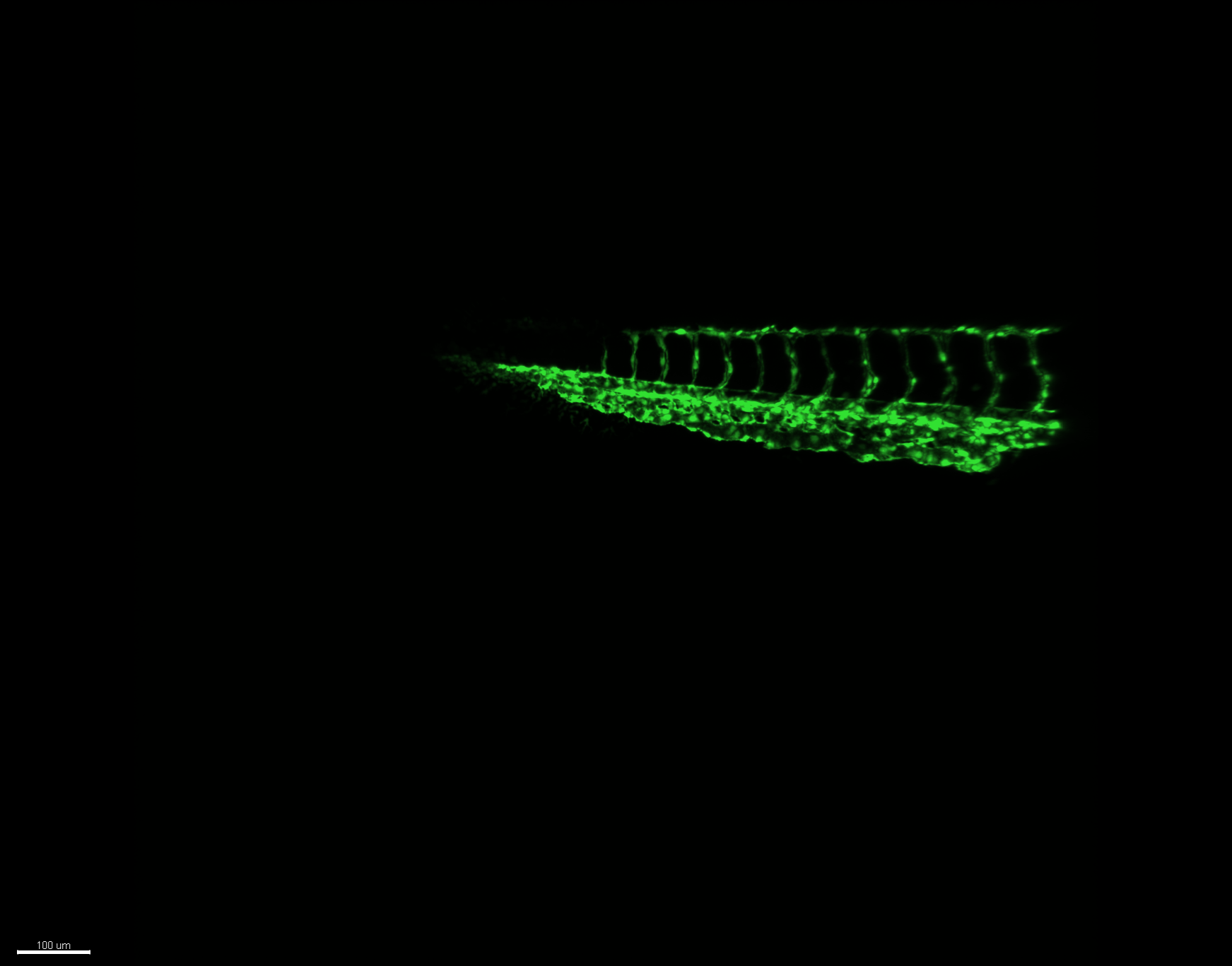

Supplement: Supplementary file 7 — Source data Fig. 2 [file 44319_2026_805_MOESM7_ESM.zip › Source Data Fig.2/Fig.2/F/2.3 36hpf fli1a trmt61a D181A.tif]

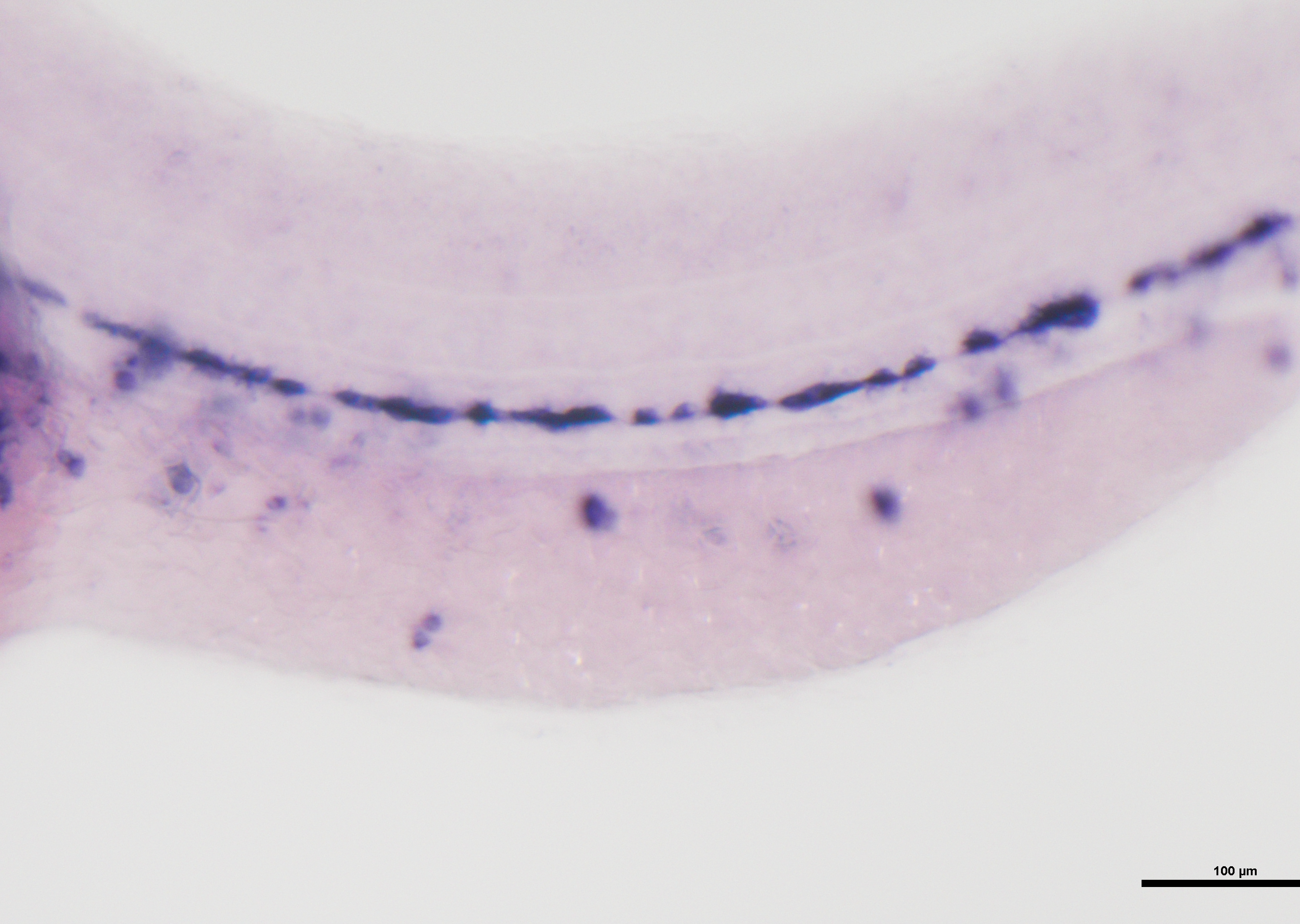

Supplement: Supplementary file 7 — Source data Fig. 2 [file 44319_2026_805_MOESM7_ESM.zip › Source Data Fig.2/Fig.2/G/1. cmyb 36hpf controlMO.tif]

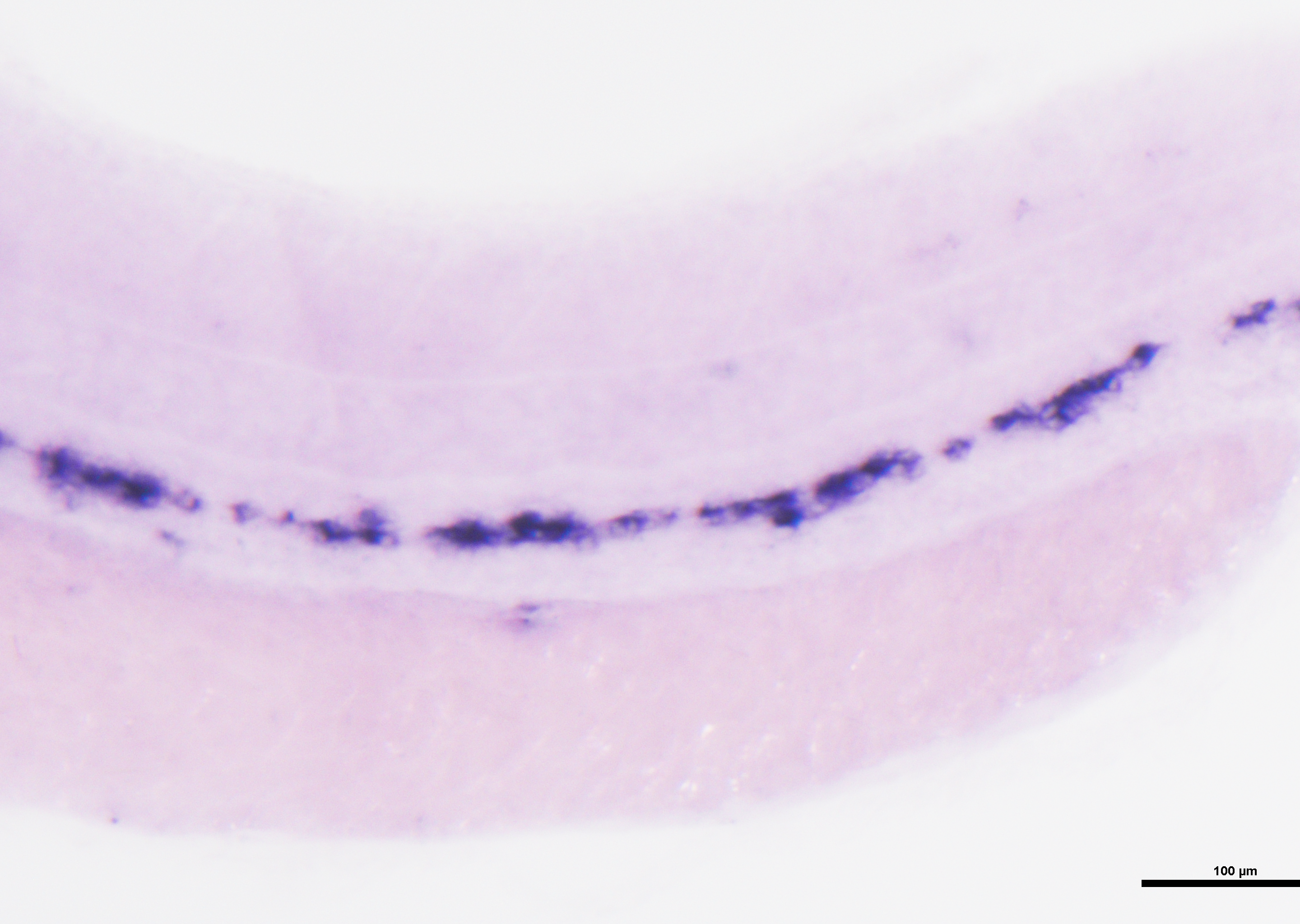

Supplement: Supplementary file 7 — Source data Fig. 2 [file 44319_2026_805_MOESM7_ESM.zip › Source Data Fig.2/Fig.2/G/2. runx1 36hpf controlMO.tif]

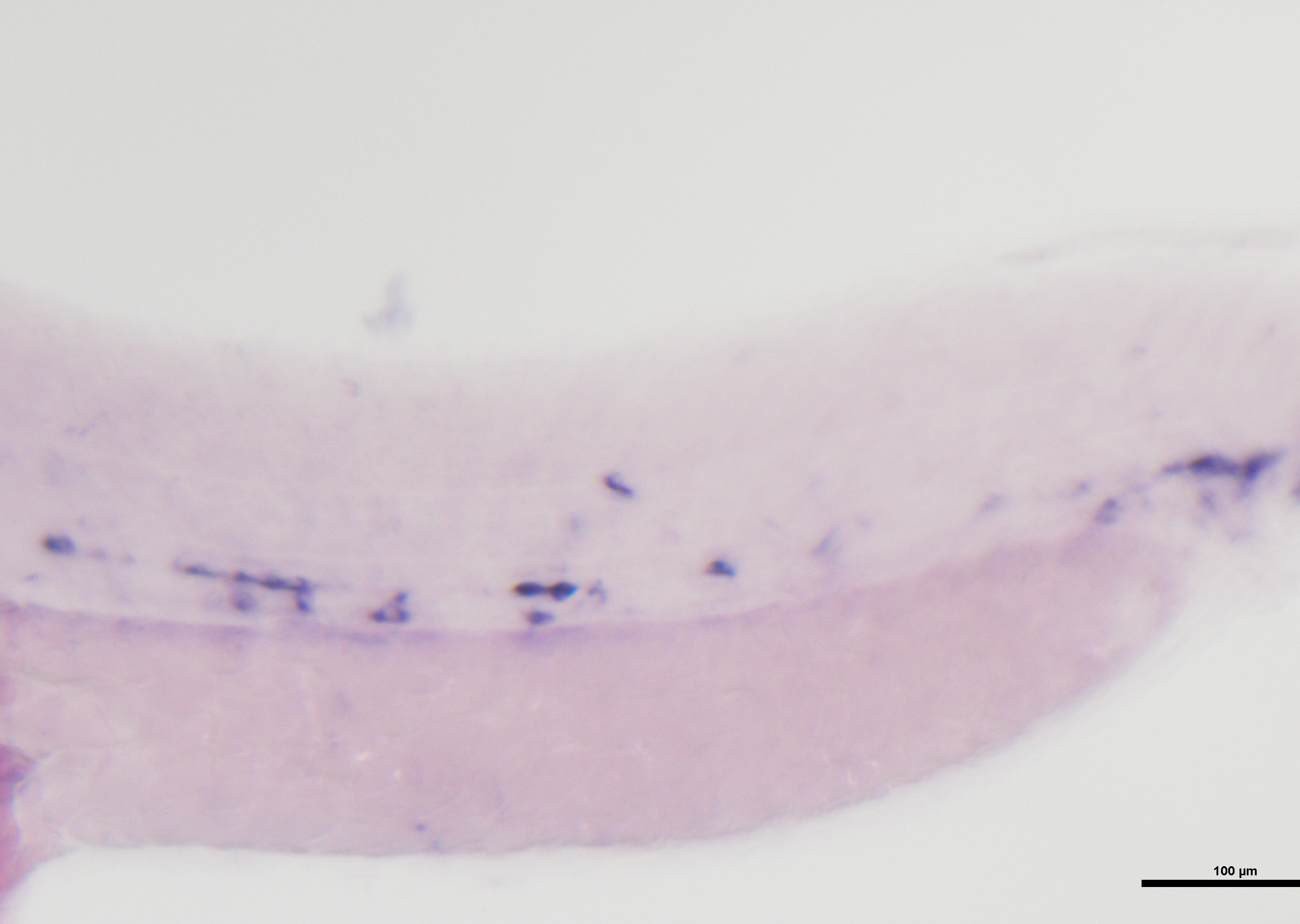

Supplement: Supplementary file 7 — Source data Fig. 2 [file 44319_2026_805_MOESM7_ESM.zip › Source Data Fig.2/Fig.2/G/3. cmyb 36hpf trmt61aMO.tif]

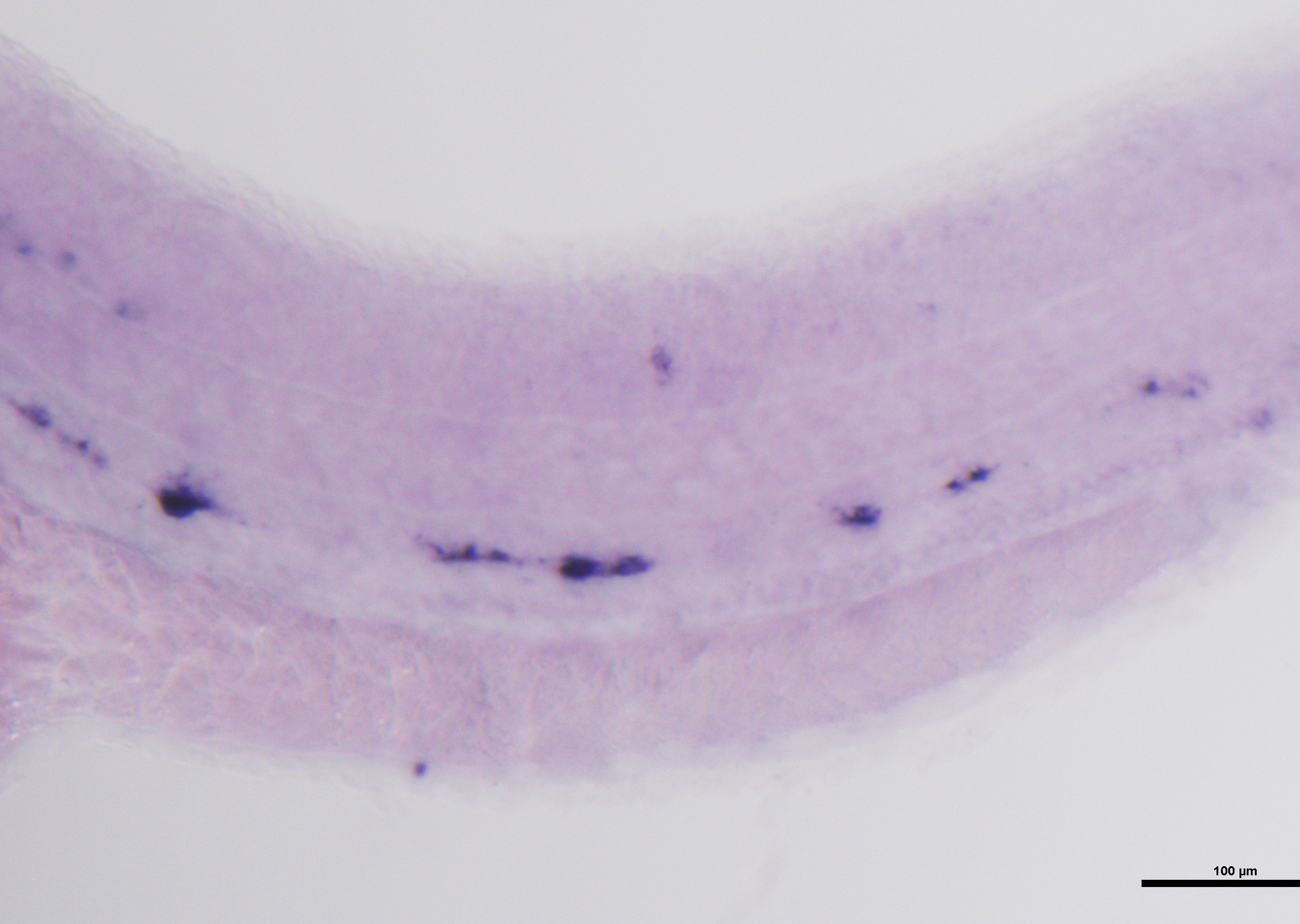

Supplement: Supplementary file 7 — Source data Fig. 2 [file 44319_2026_805_MOESM7_ESM.zip › Source Data Fig.2/Fig.2/G/4. runx1 36hpf trmt61aMO.tif]

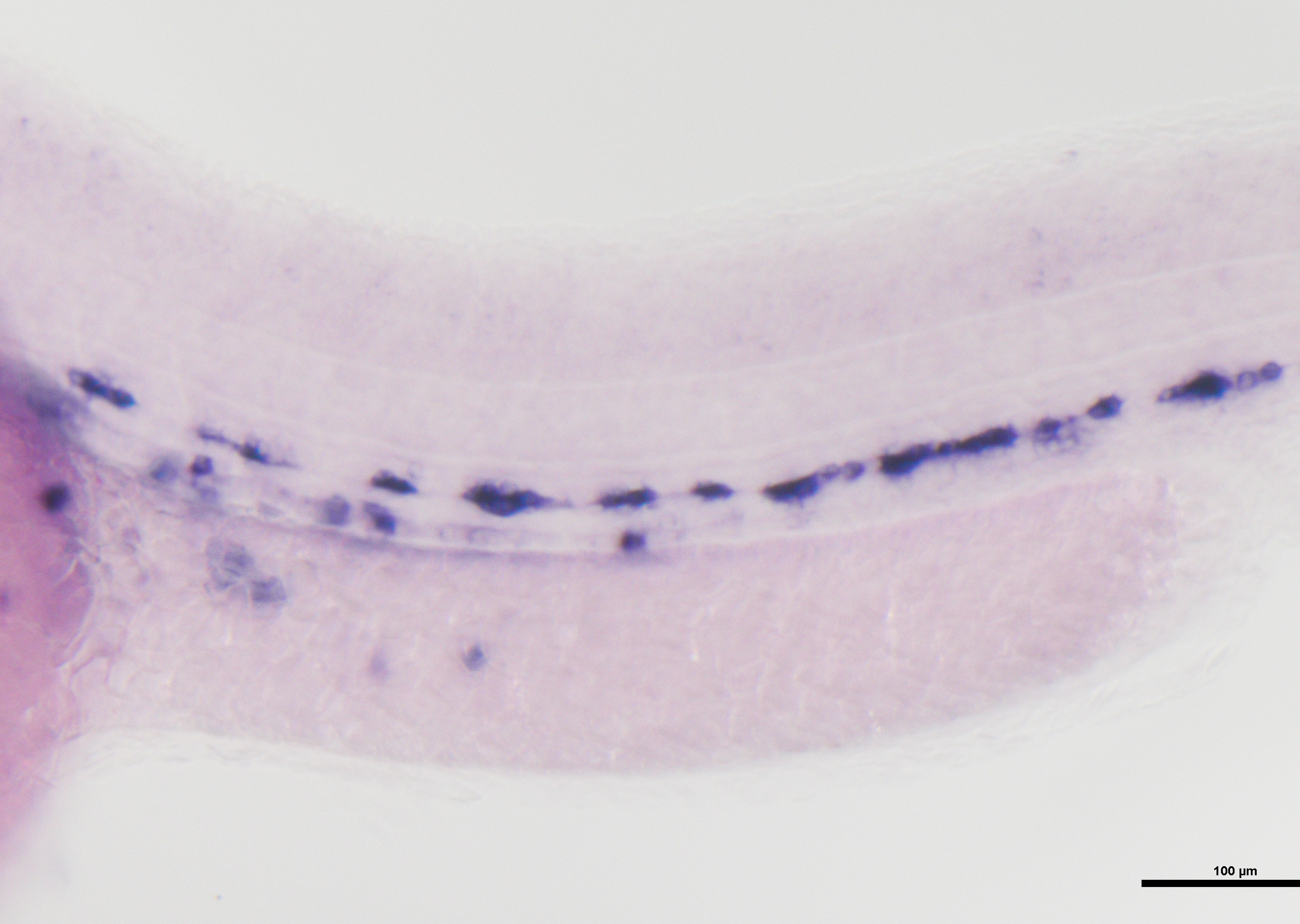

Supplement: Supplementary file 7 — Source data Fig. 2 [file 44319_2026_805_MOESM7_ESM.zip › Source Data Fig.2/Fig.2/G/5. cmyb 36hpf trmt61aMO+fli1atrmt61aWT.tif]

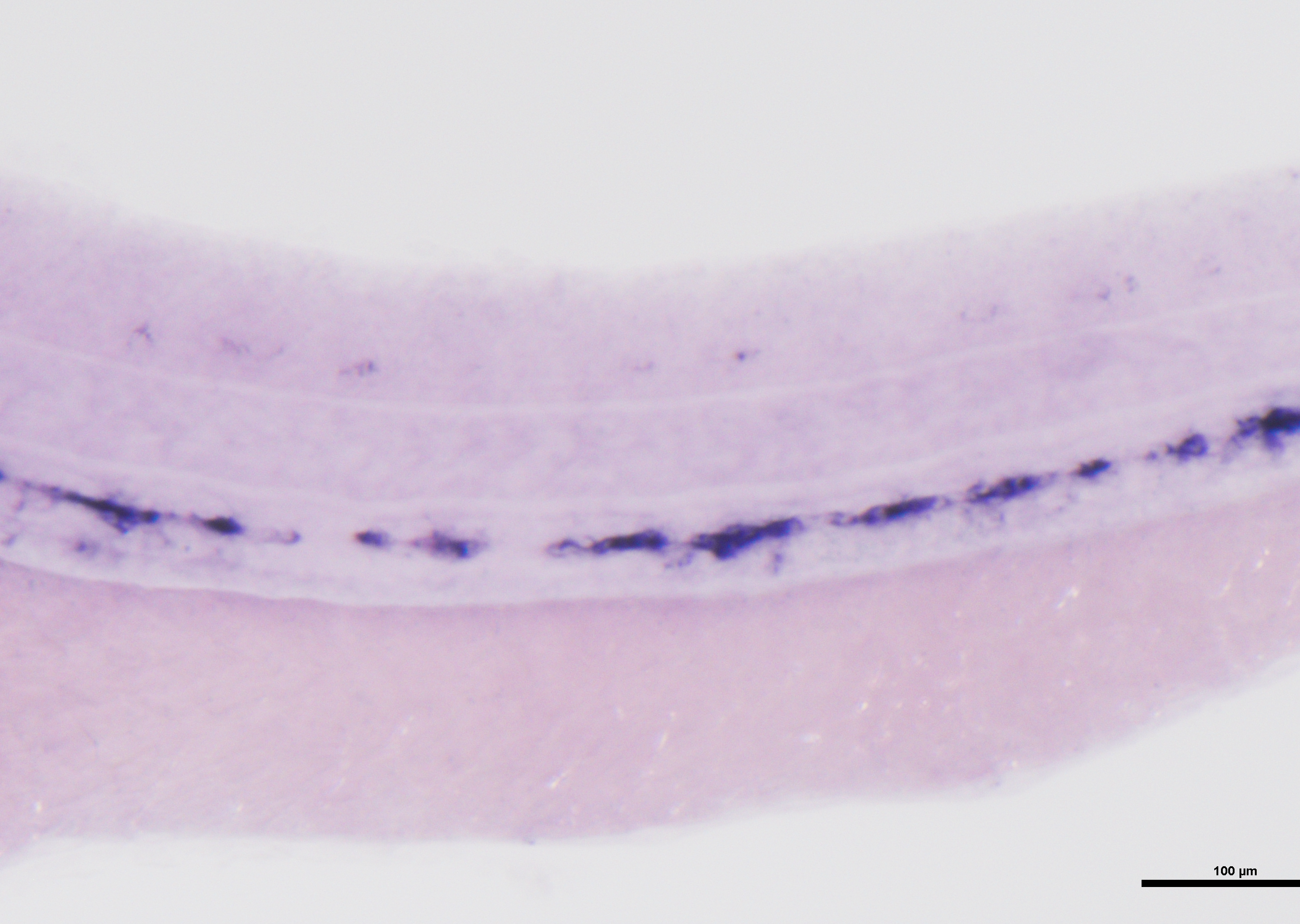

Supplement: Supplementary file 7 — Source data Fig. 2 [file 44319_2026_805_MOESM7_ESM.zip › Source Data Fig.2/Fig.2/G/6. runx1 36hpf trmt61aMO+fli1atrmt61aWT.tif]

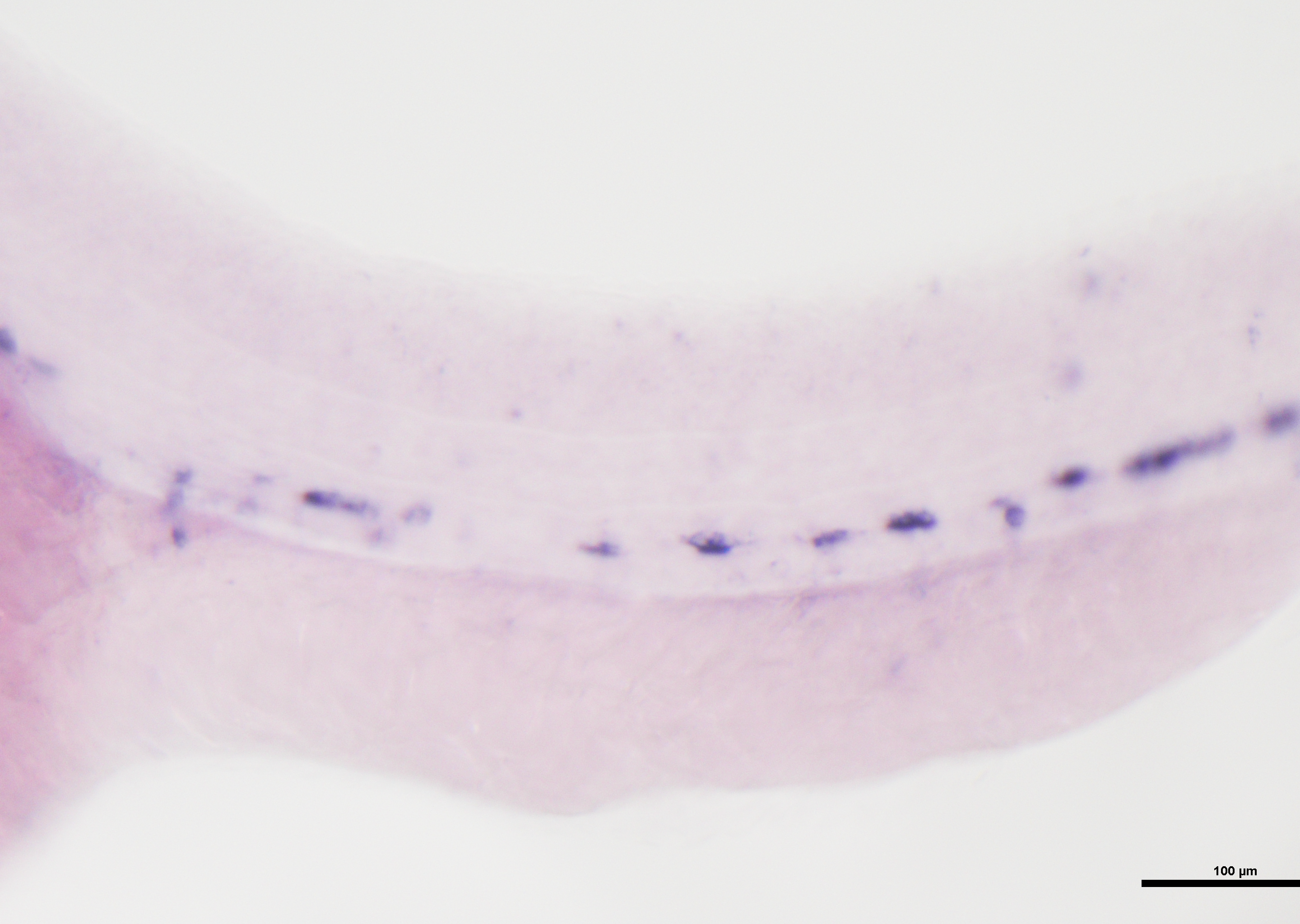

Supplement: Supplementary file 7 — Source data Fig. 2 [file 44319_2026_805_MOESM7_ESM.zip › Source Data Fig.2/Fig.2/G/7. cmyb 36hpf trmt61aMO+fli1atrmt61aD181A.tif]

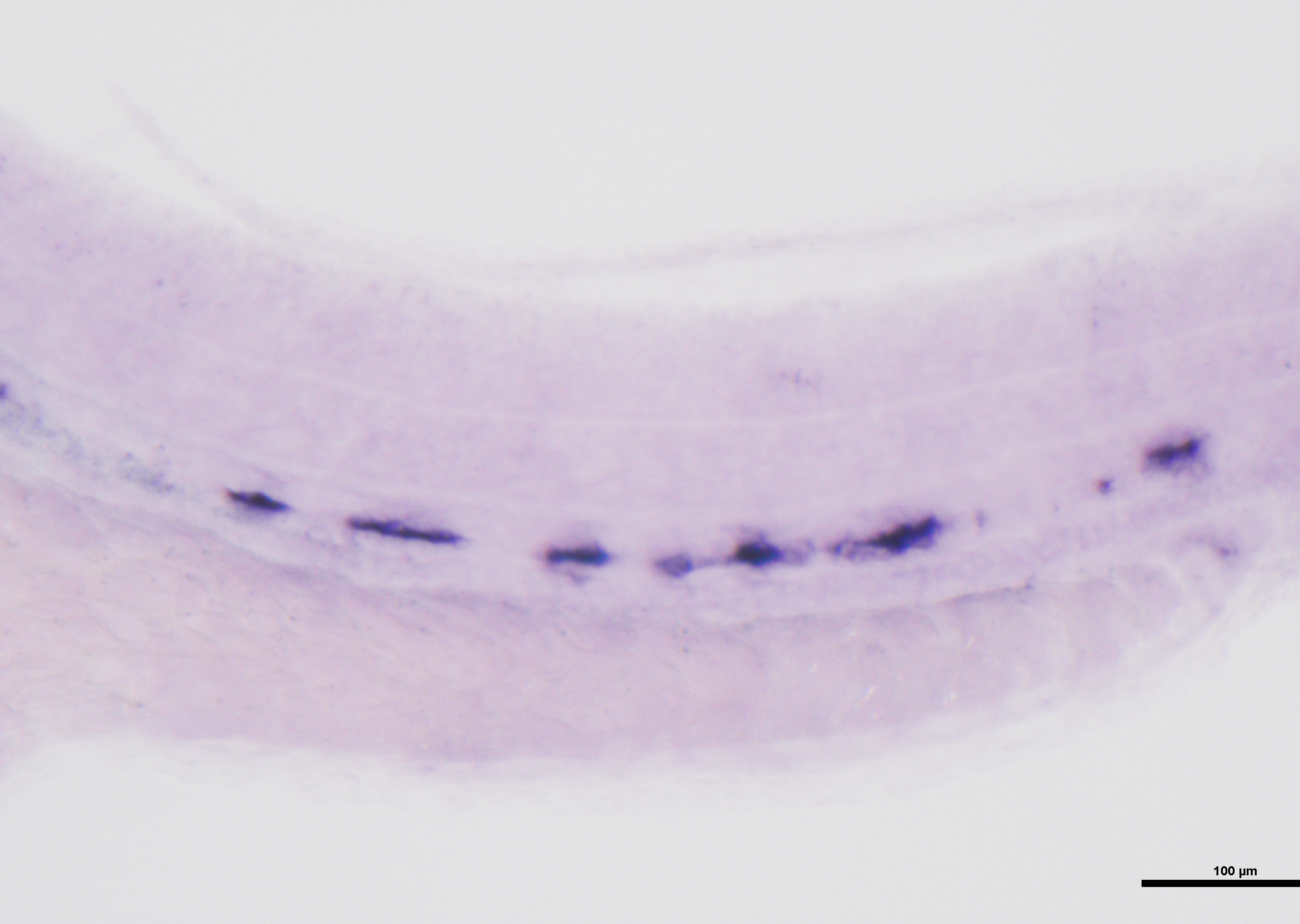

Supplement: Supplementary file 7 — Source data Fig. 2 [file 44319_2026_805_MOESM7_ESM.zip › Source Data Fig.2/Fig.2/G/8. runx1 36hpf trmt61aMO+fli1atrmt61aD181A.tif]

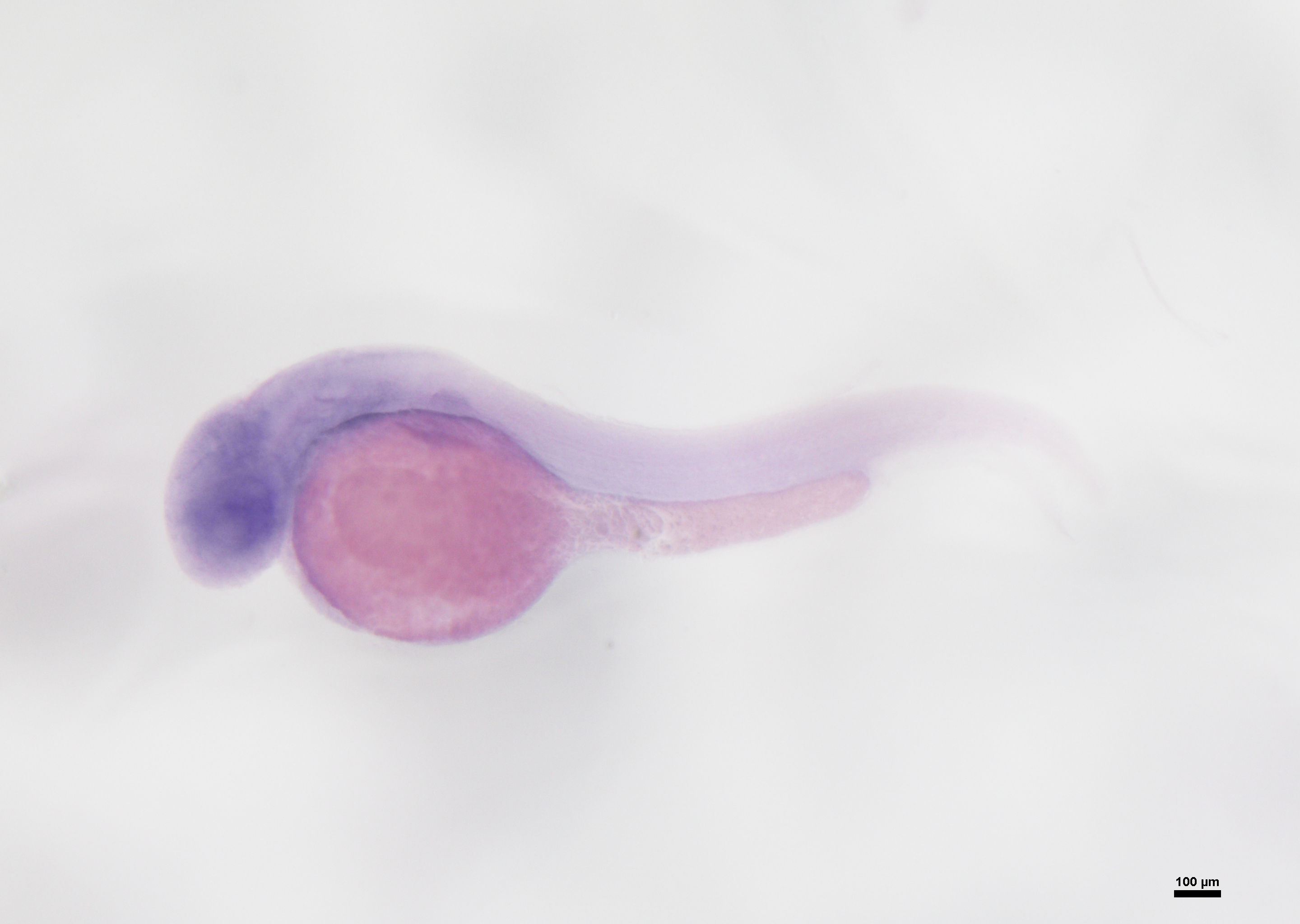

Supplement: Supplementary file 8 — Source data Fig. 3 [file 44319_2026_805_MOESM8_ESM.zip › Source Data Fig.3/Fig.3/D/1. p53 36hpf sibling.tif]

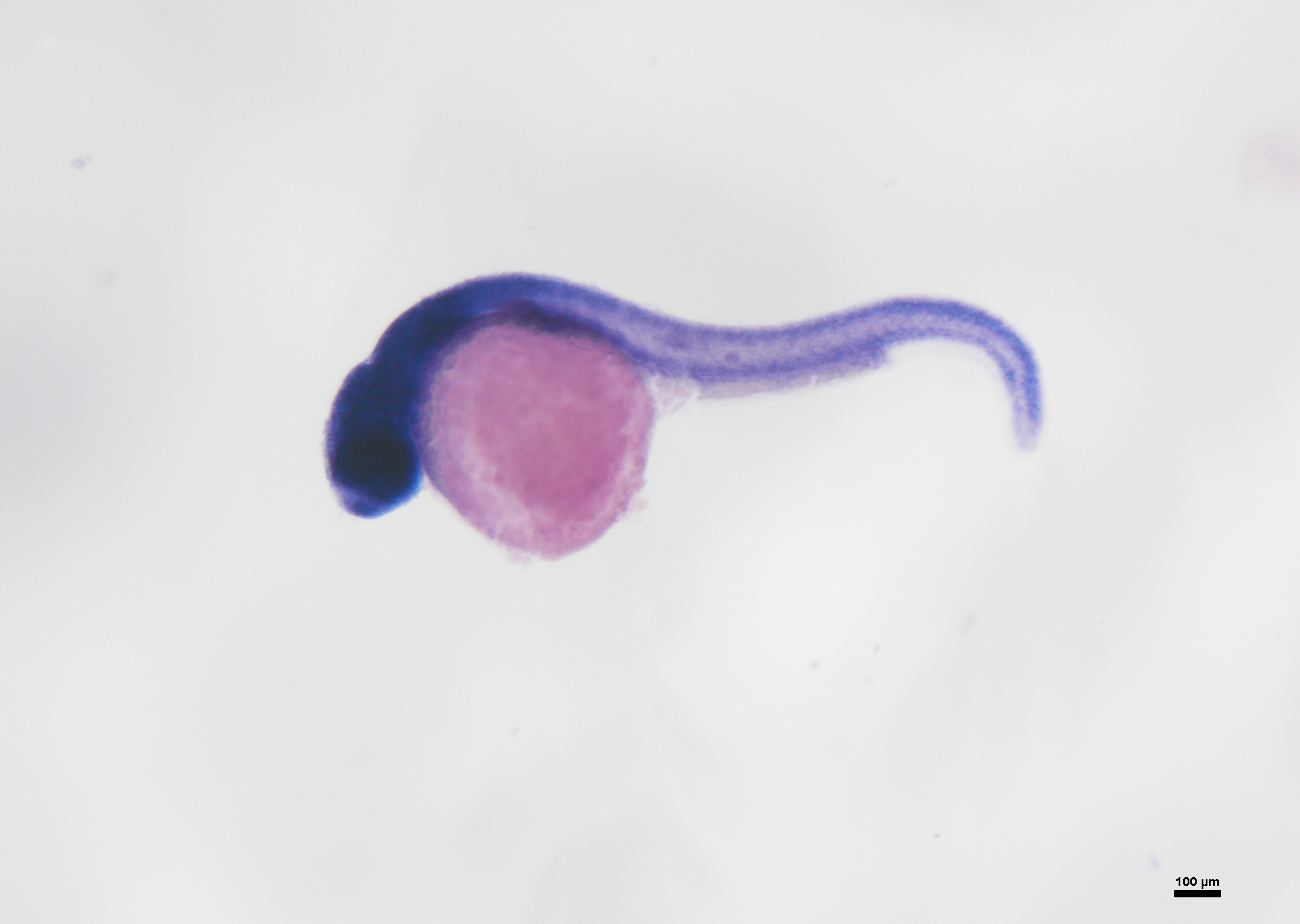

Supplement: Supplementary file 8 — Source data Fig. 3 [file 44319_2026_805_MOESM8_ESM.zip › Source Data Fig.3/Fig.3/D/2. p53 36hpf Mtrmt61a;trmt61a-4bp.tif]

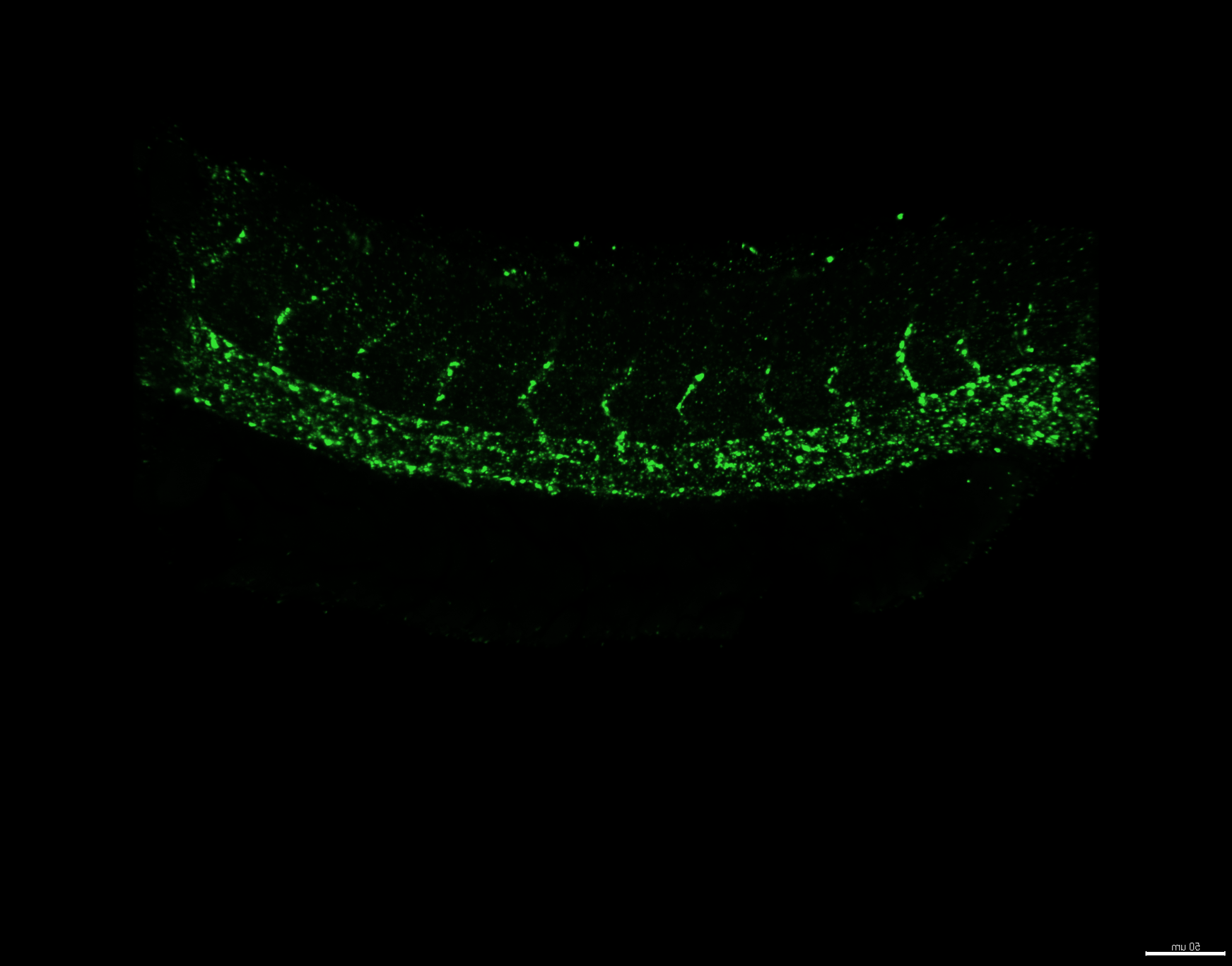

Supplement: Supplementary file 8 — Source data Fig. 3 [file 44319_2026_805_MOESM8_ESM.zip › Source Data Fig.3/Fig.3/F/1. kdrl 36hpf sibling.tif]

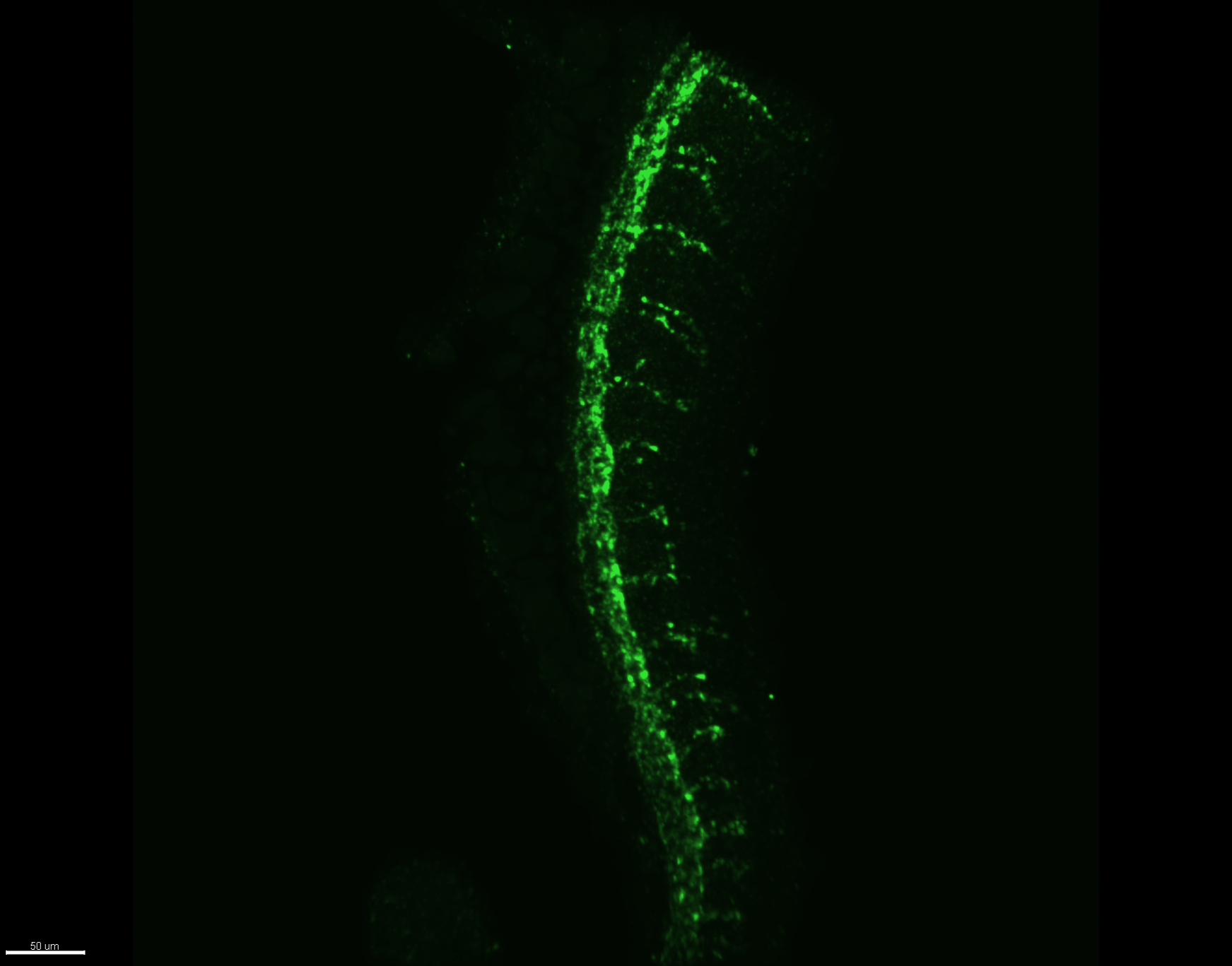

Supplement: Supplementary file 8 — Source data Fig. 3 [file 44319_2026_805_MOESM8_ESM.zip › Source Data Fig.3/Fig.3/F/2. kdrl 36hpf Mtrmt61a;trmt61a-4bp.tif]

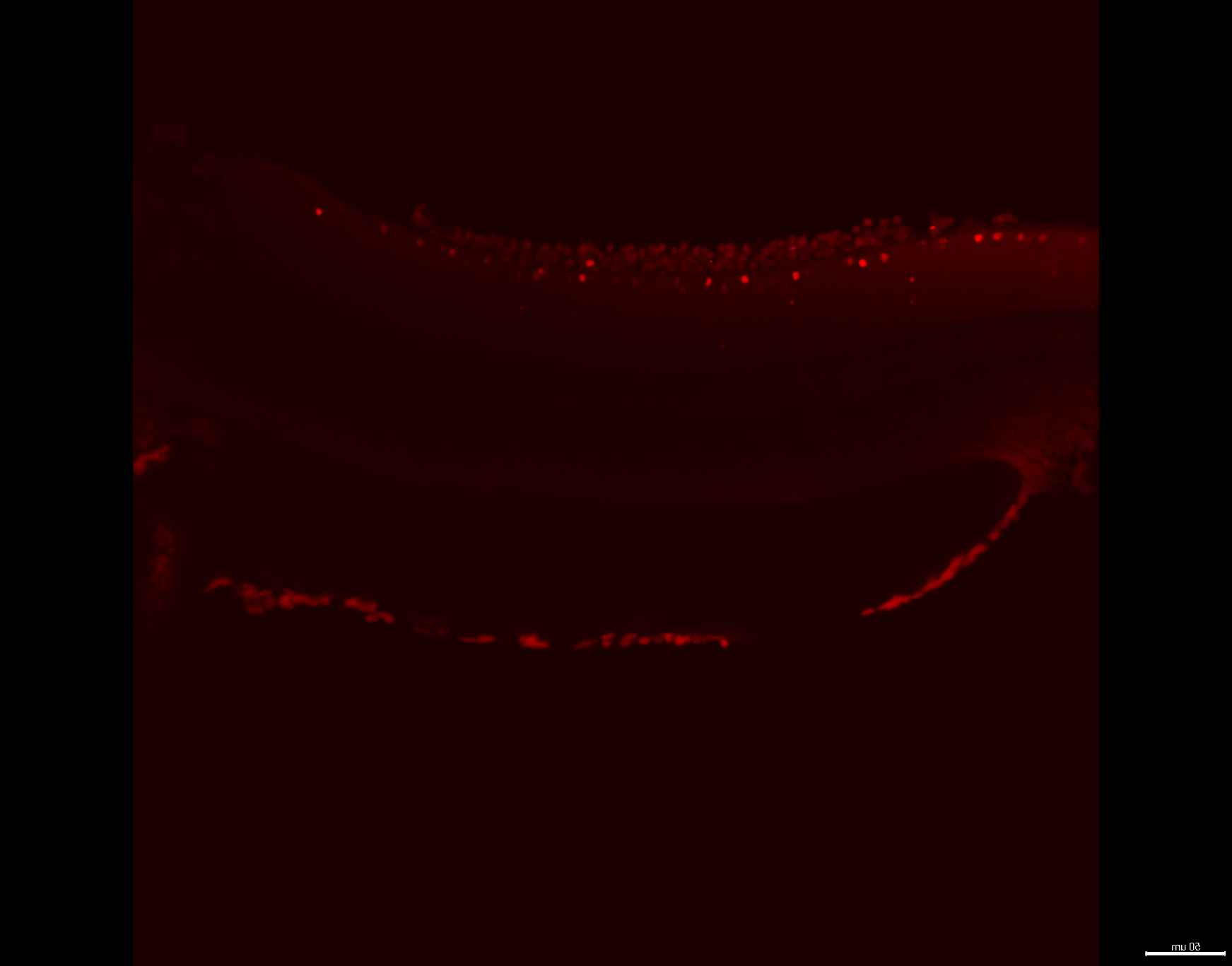

Supplement: Supplementary file 8 — Source data Fig. 3 [file 44319_2026_805_MOESM8_ESM.zip › Source Data Fig.3/Fig.3/F/3. tunel 36hpf sibling.tif]

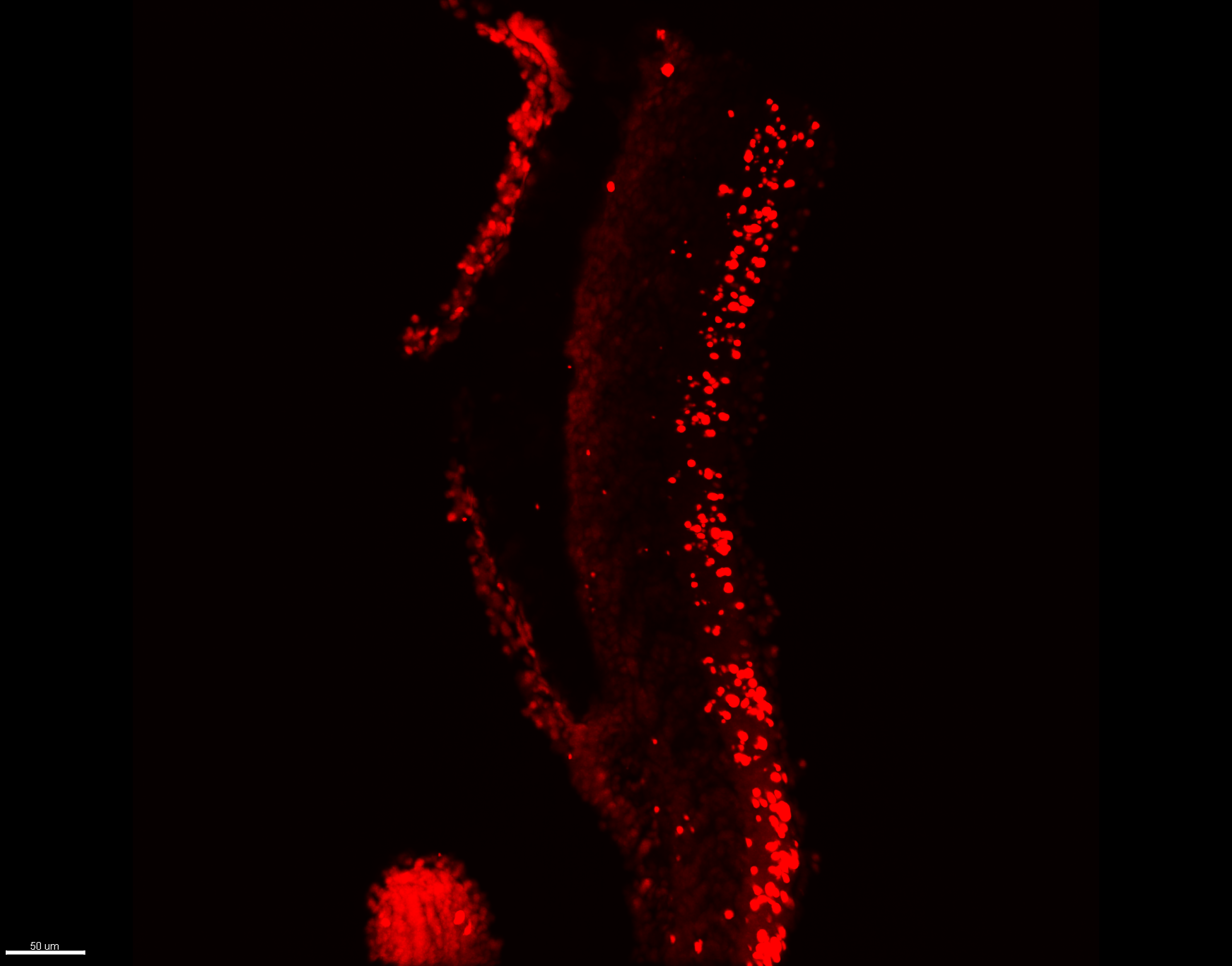

Supplement: Supplementary file 8 — Source data Fig. 3 [file 44319_2026_805_MOESM8_ESM.zip › Source Data Fig.3/Fig.3/F/4. tunel 36hpf Mtrmt61a;trmt61a-4bp.tif]

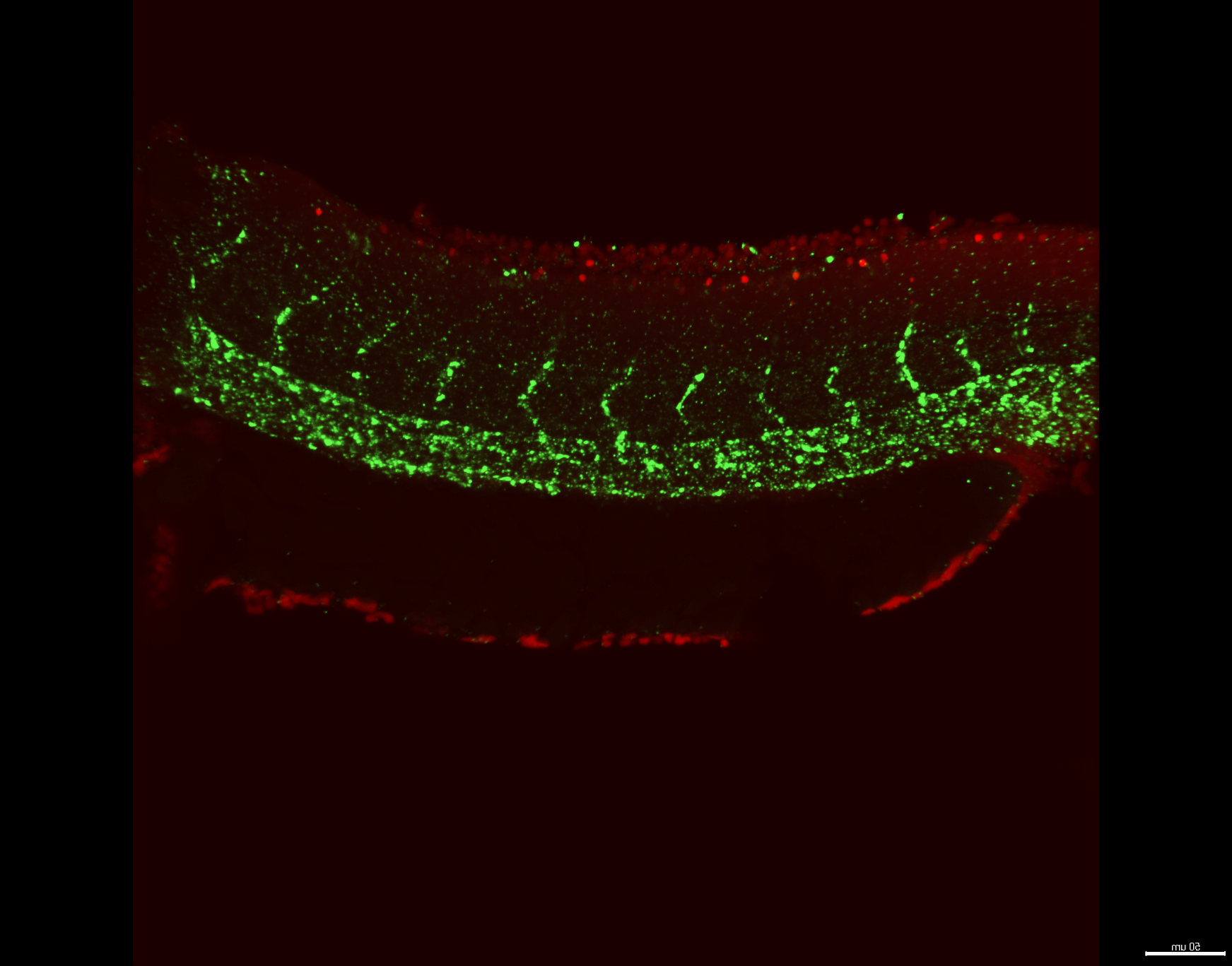

Supplement: Supplementary file 8 — Source data Fig. 3 [file 44319_2026_805_MOESM8_ESM.zip › Source Data Fig.3/Fig.3/F/5. merge 36hpf sibling.tif]

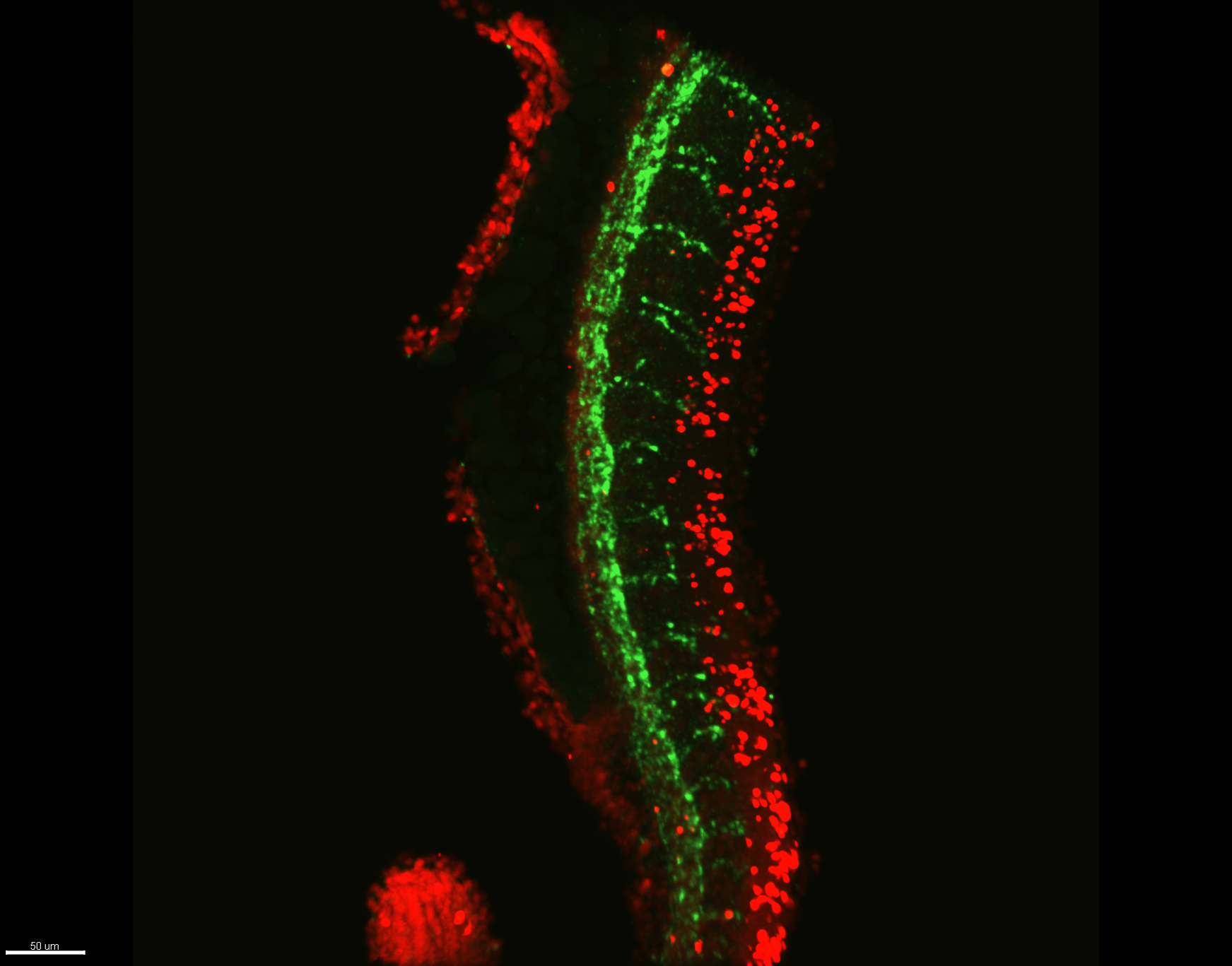

Supplement: Supplementary file 8 — Source data Fig. 3 [file 44319_2026_805_MOESM8_ESM.zip › Source Data Fig.3/Fig.3/F/6. merge 36hpf Mtrmt61a;trmt61a-4bp.tif]

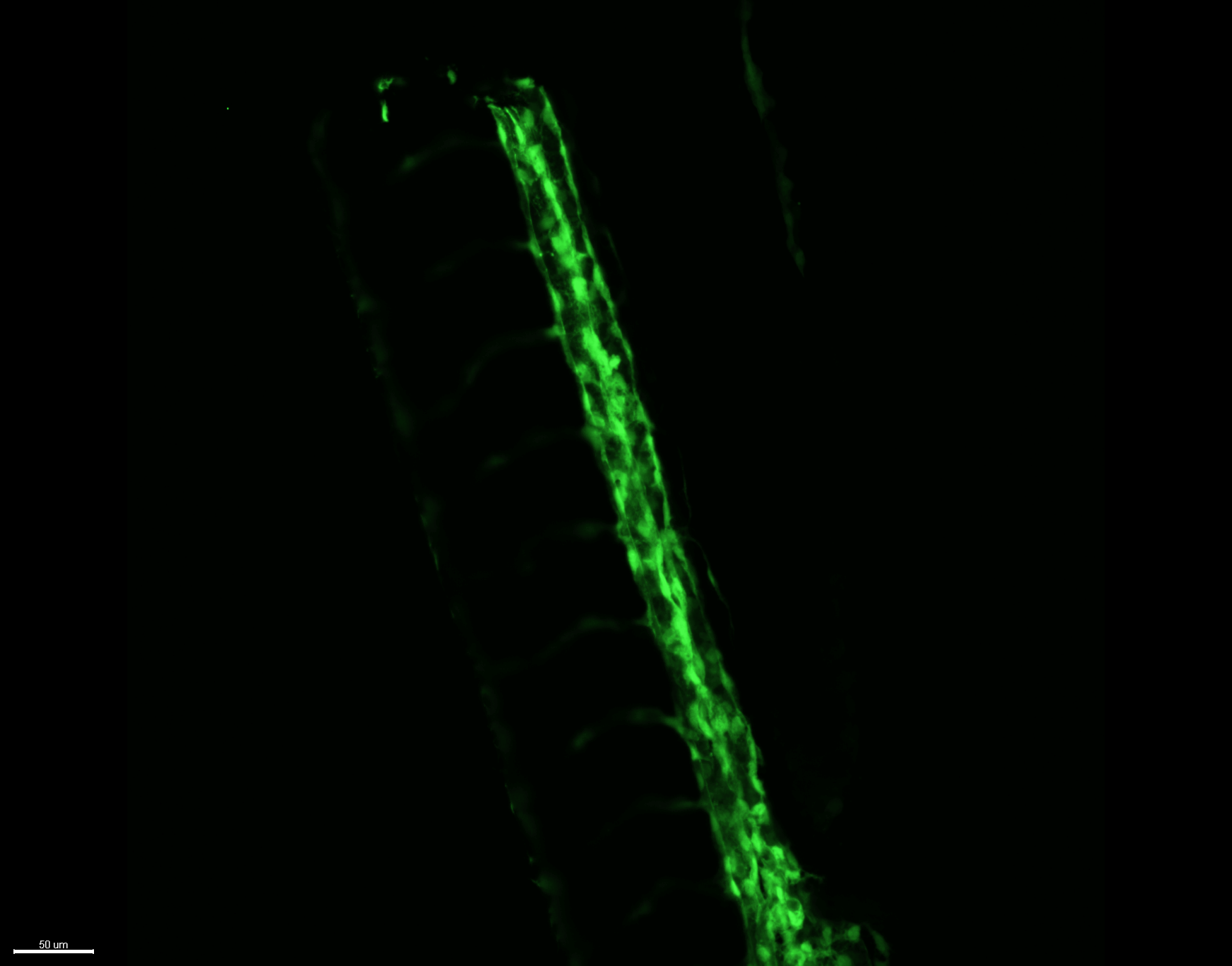

Supplement: Supplementary file 8 — Source data Fig. 3 [file 44319_2026_805_MOESM8_ESM.zip › Source Data Fig.3/Fig.3/H/1. fli1aEGFP 36hpf controlMO.tif]

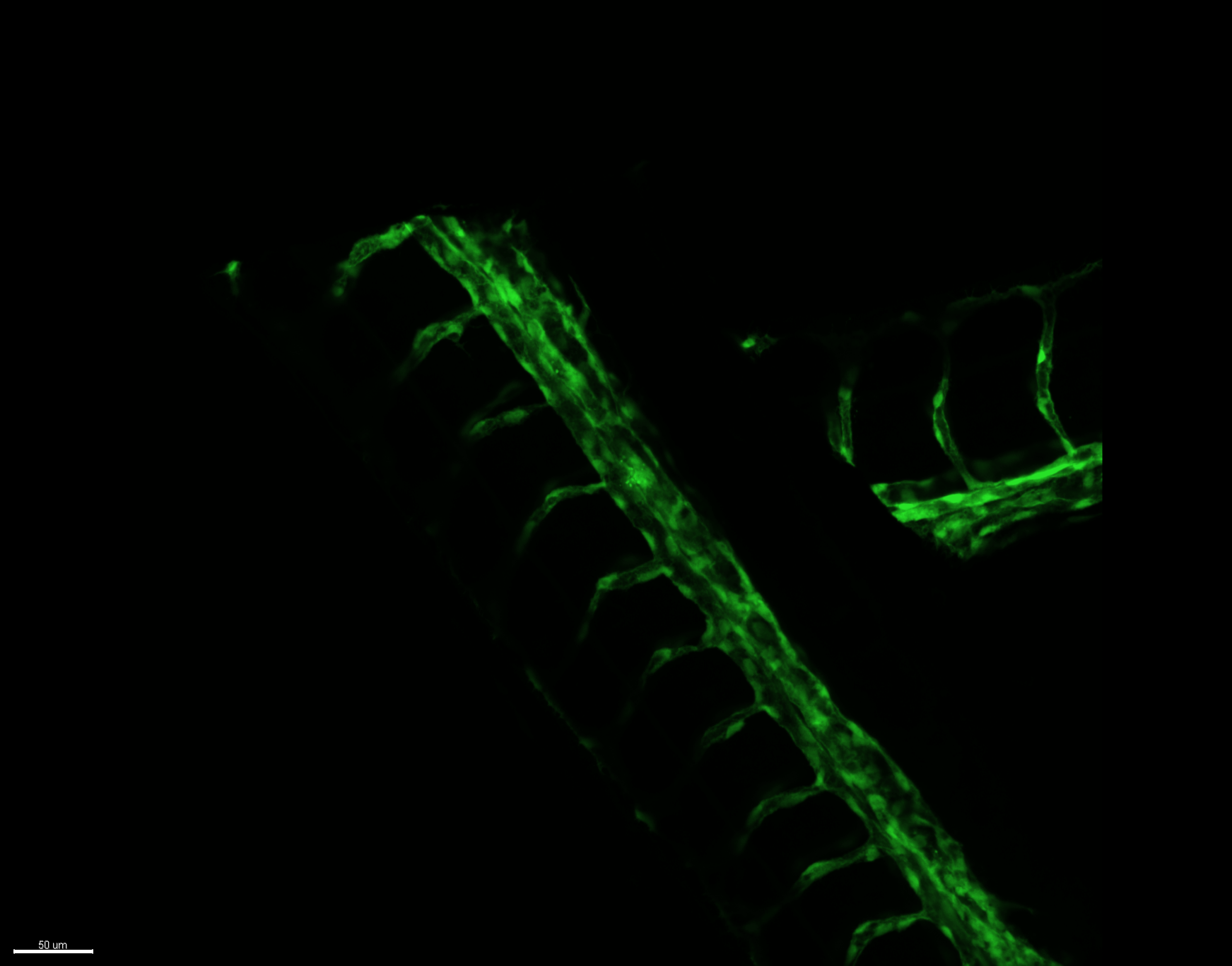

Supplement: Supplementary file 8 — Source data Fig. 3 [file 44319_2026_805_MOESM8_ESM.zip › Source Data Fig.3/Fig.3/H/10. fli1aEGFP 36hpf trmt61aMO+p53MO.tif]

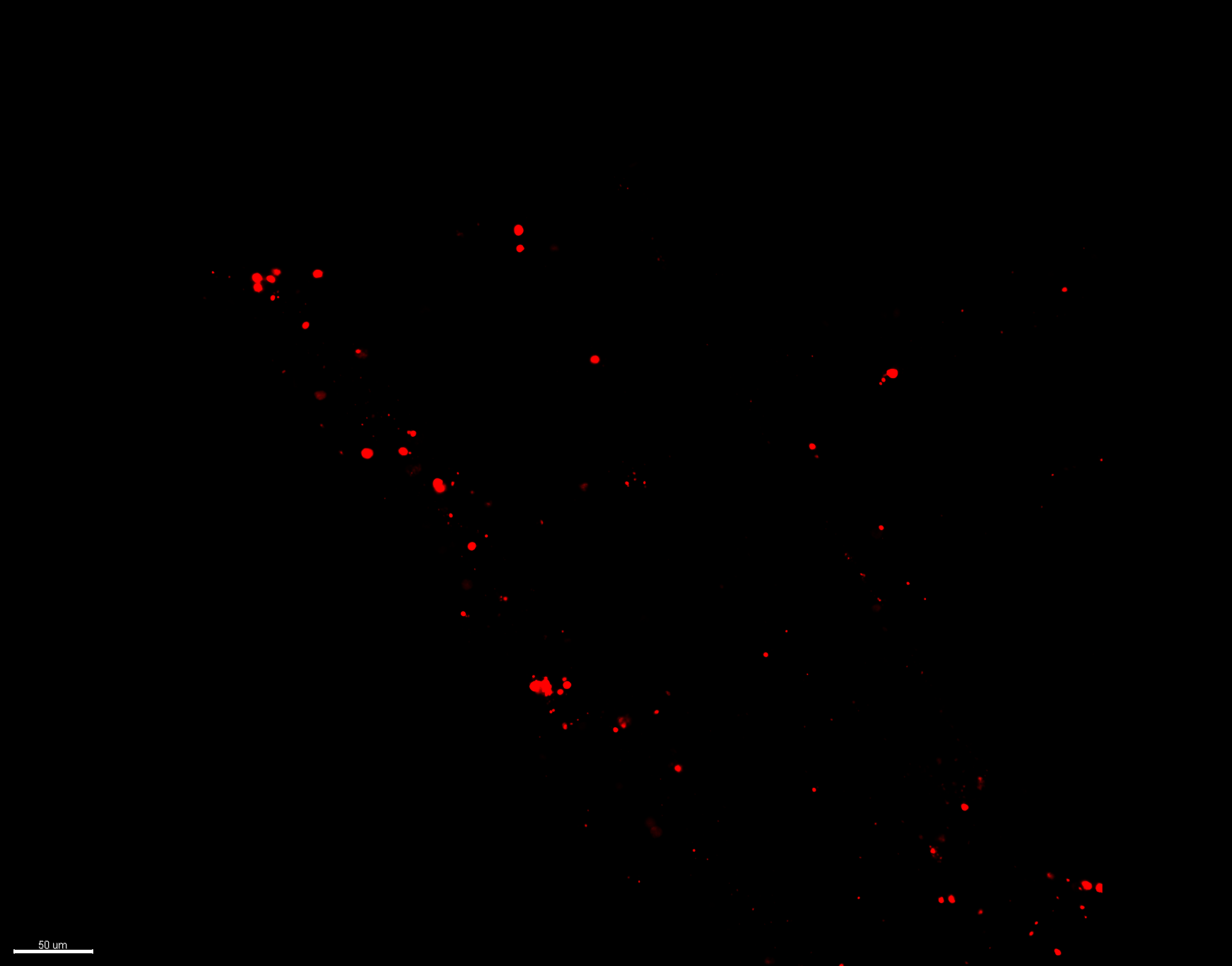

Supplement: Supplementary file 8 — Source data Fig. 3 [file 44319_2026_805_MOESM8_ESM.zip › Source Data Fig.3/Fig.3/H/11. tunel 36hpf trmt61aMO+p53MO.tif]

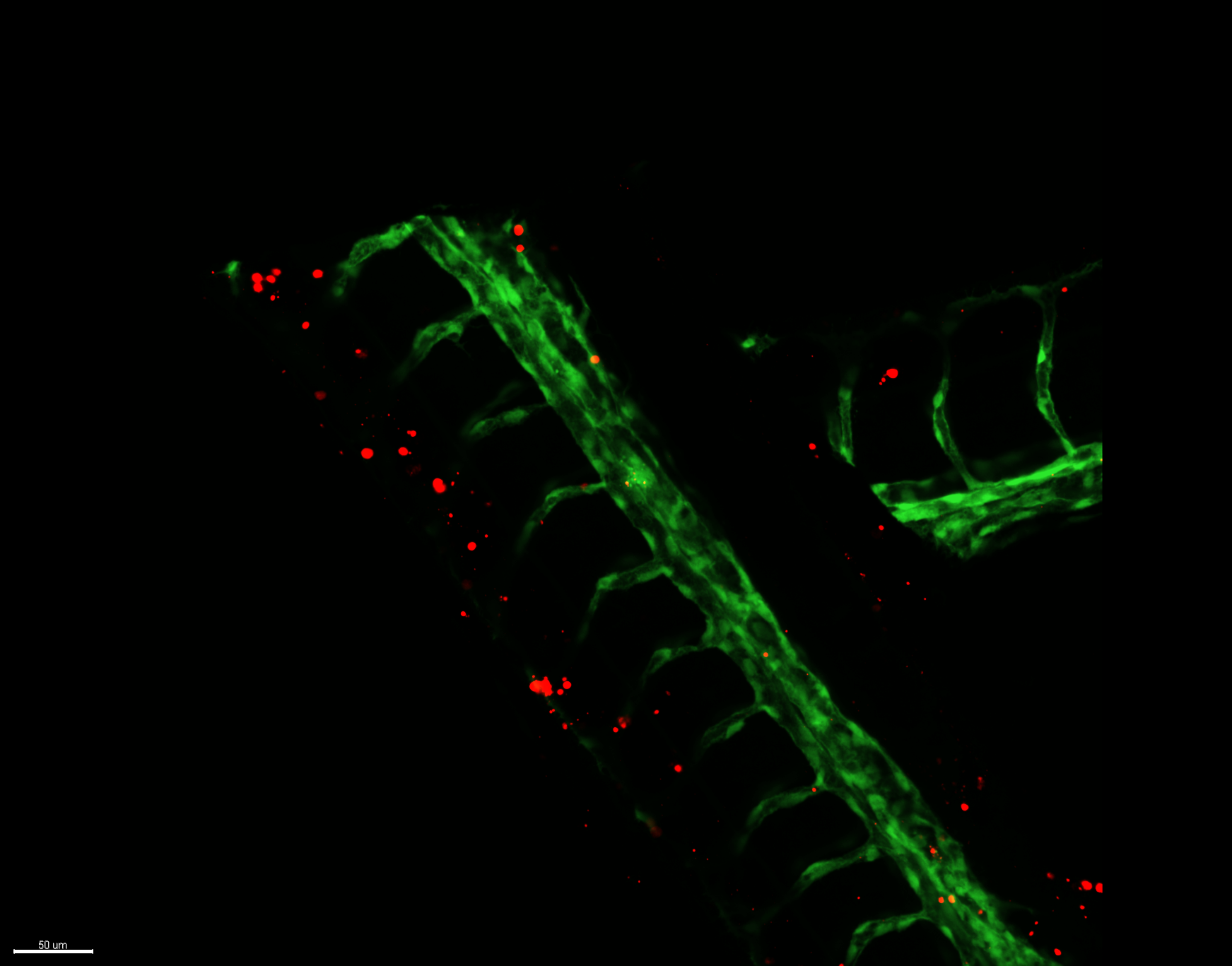

Supplement: Supplementary file 8 — Source data Fig. 3 [file 44319_2026_805_MOESM8_ESM.zip › Source Data Fig.3/Fig.3/H/12. merge 36hpf trmt61aMO+p53MO.tif]

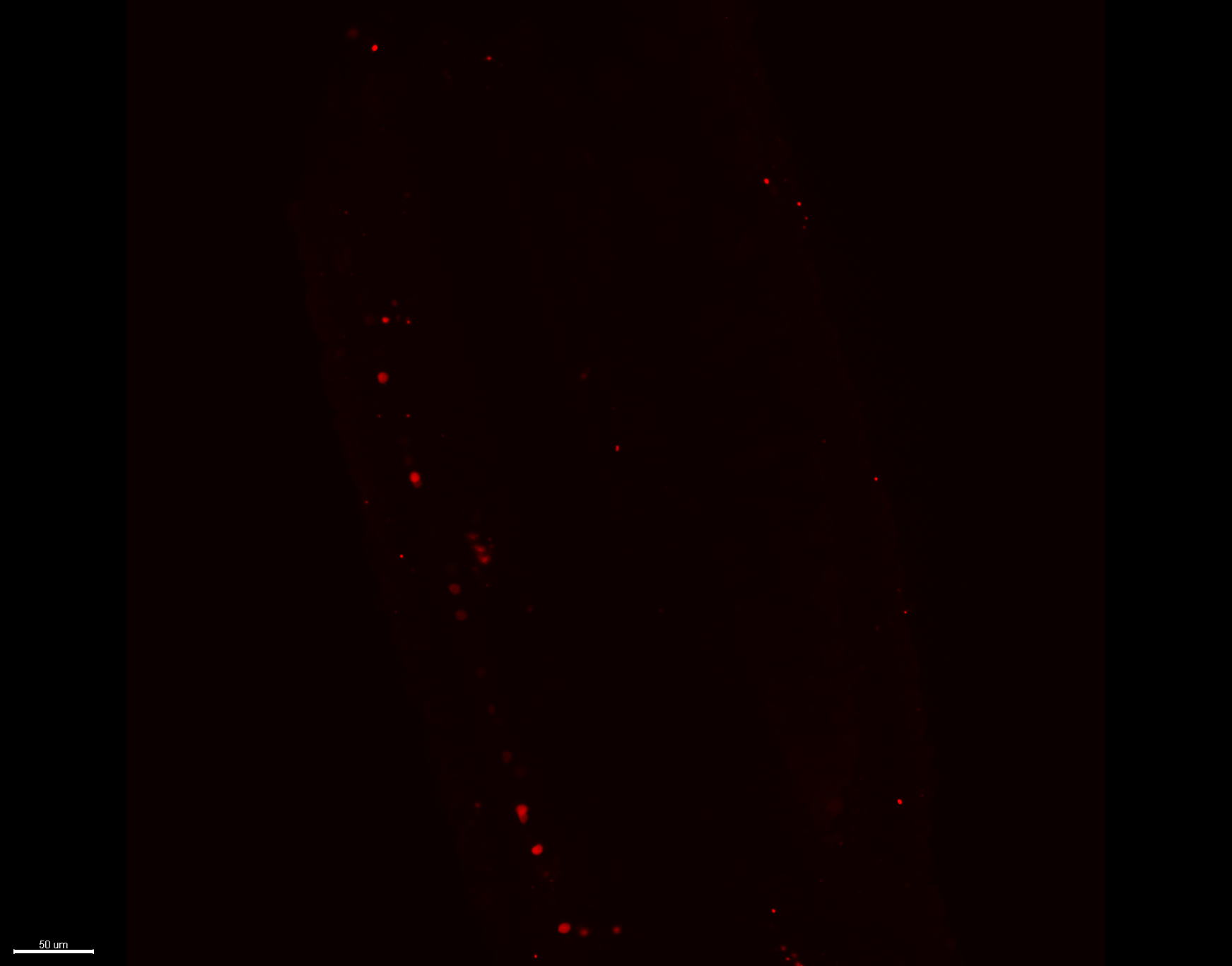

Supplement: Supplementary file 8 — Source data Fig. 3 [file 44319_2026_805_MOESM8_ESM.zip › Source Data Fig.3/Fig.3/H/2. tunel 36hpf controlMO.tif]

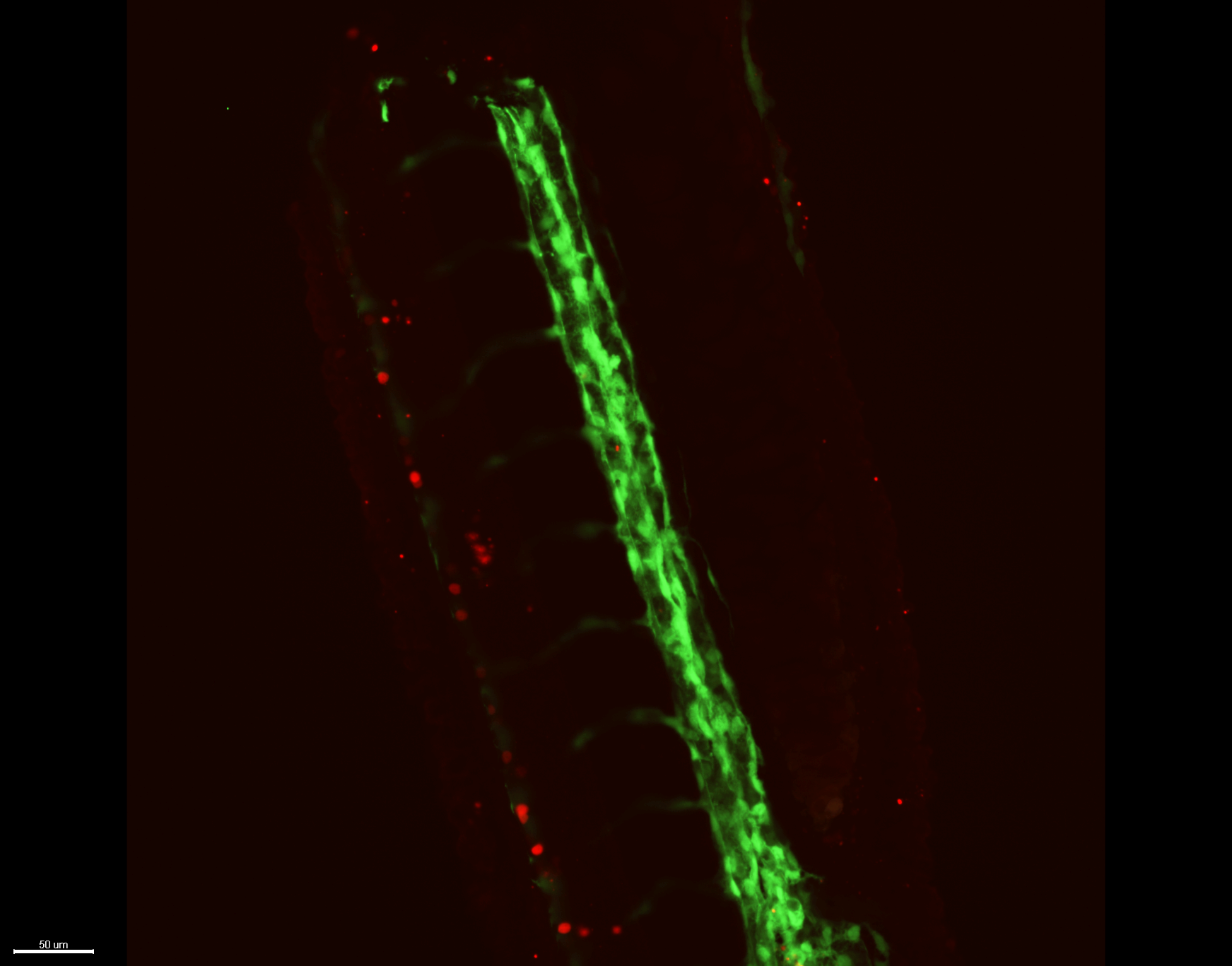

Supplement: Supplementary file 8 — Source data Fig. 3 [file 44319_2026_805_MOESM8_ESM.zip › Source Data Fig.3/Fig.3/H/3. merge 36hpf controlMO.tif]

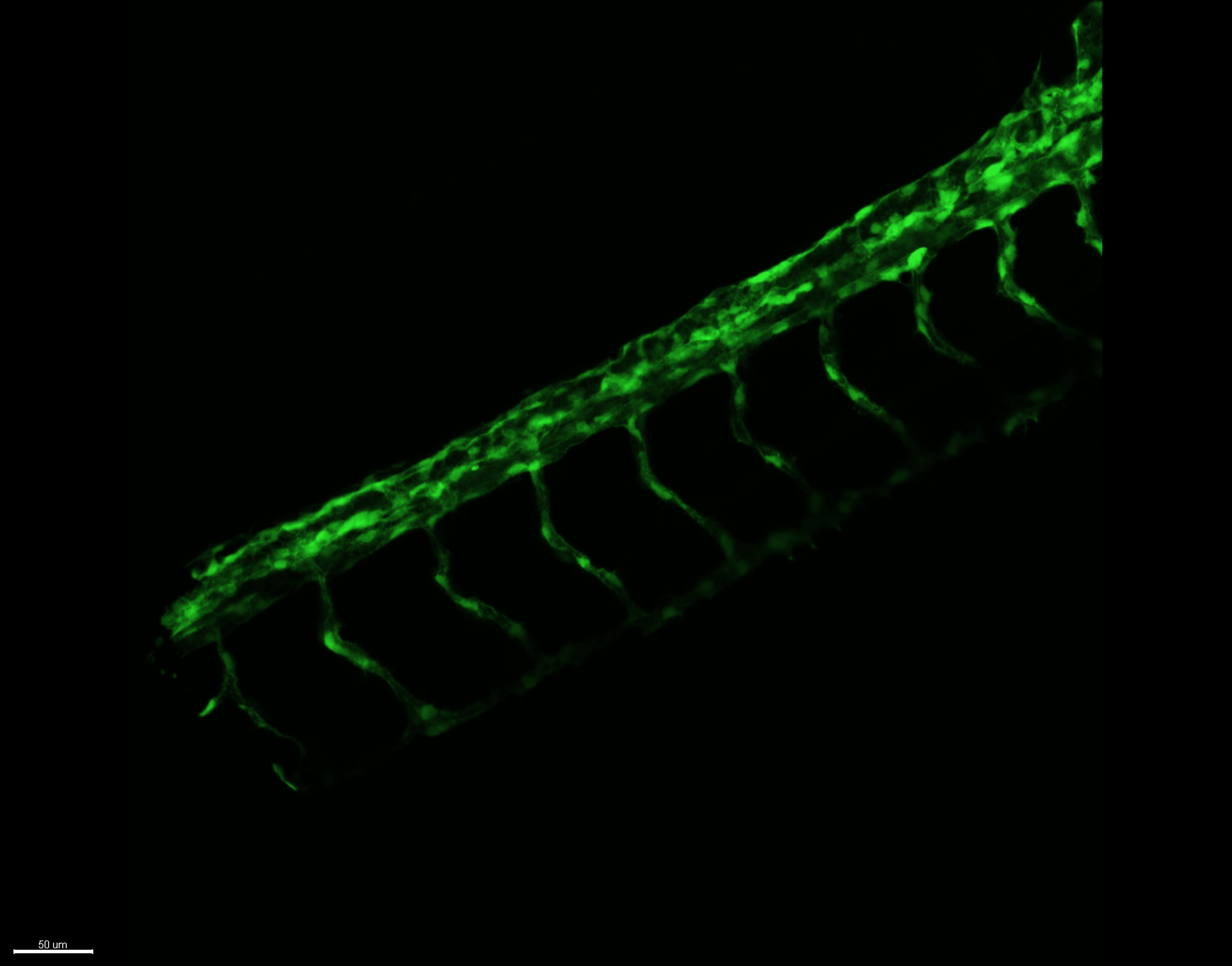

Supplement: Supplementary file 8 — Source data Fig. 3 [file 44319_2026_805_MOESM8_ESM.zip › Source Data Fig.3/Fig.3/H/4. fli1aEGFP 36hpf controlMO+p53MO.tif]

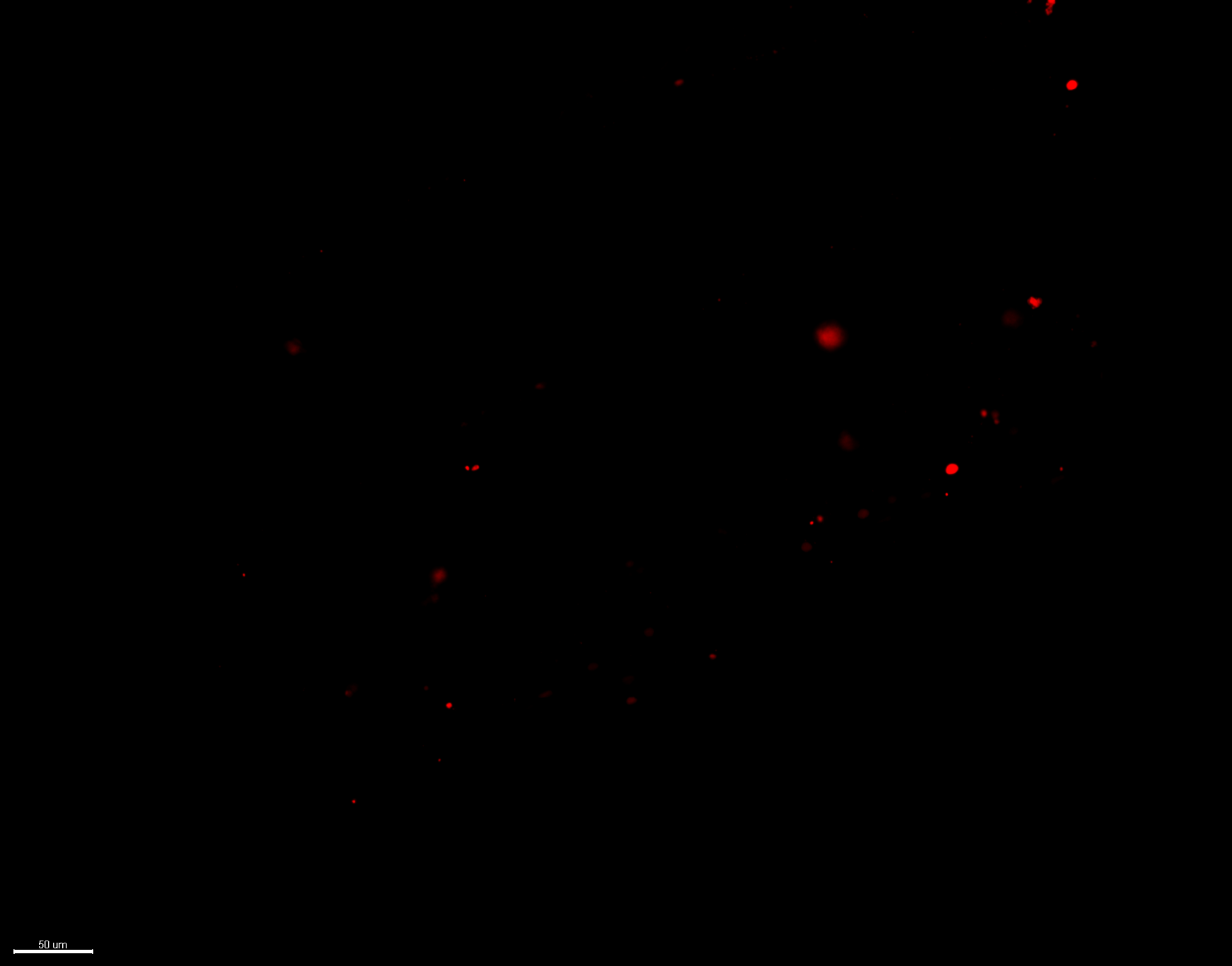

Supplement: Supplementary file 8 — Source data Fig. 3 [file 44319_2026_805_MOESM8_ESM.zip › Source Data Fig.3/Fig.3/H/5. tunel 36hpf controlMO+p53MO.tif]

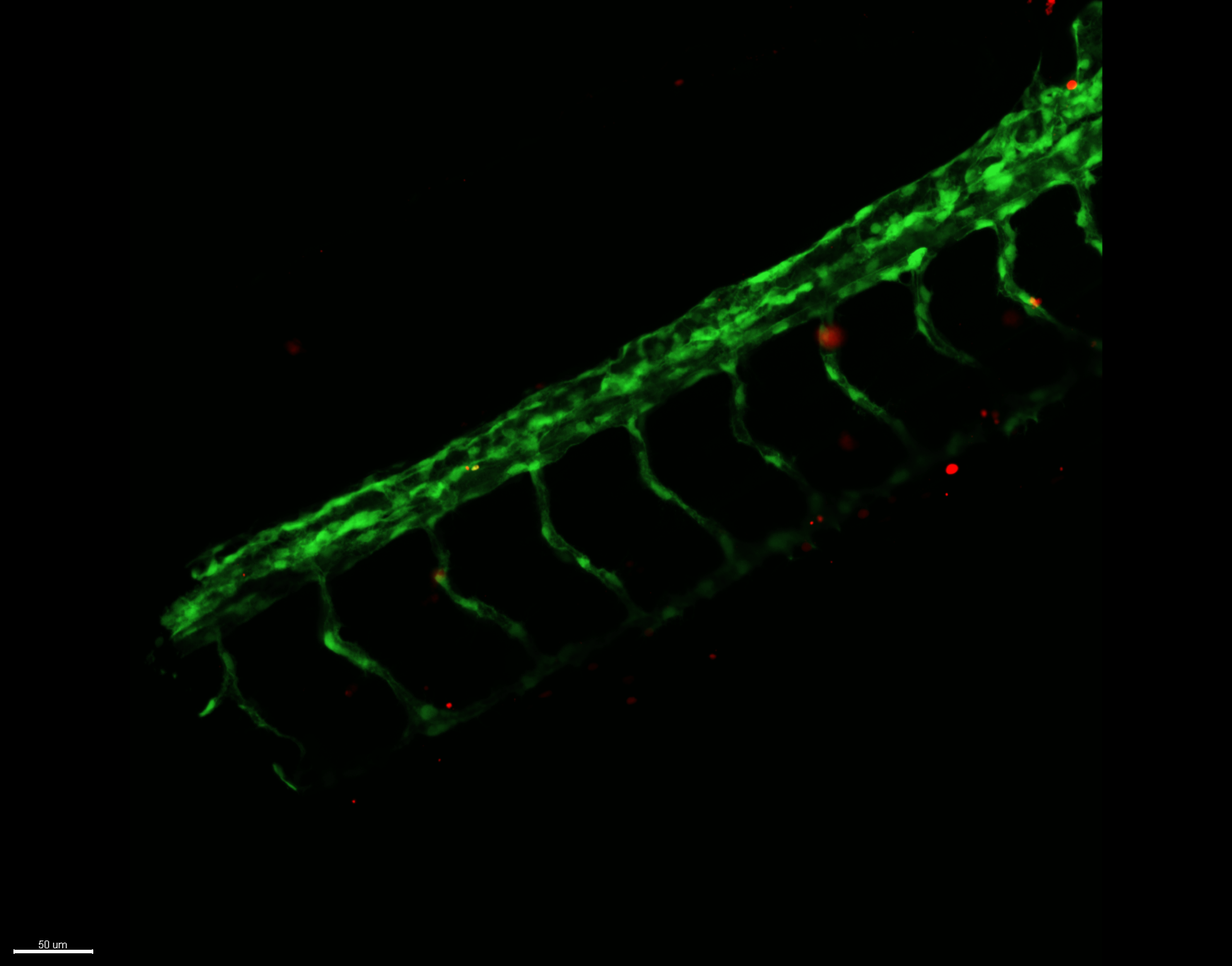

Supplement: Supplementary file 8 — Source data Fig. 3 [file 44319_2026_805_MOESM8_ESM.zip › Source Data Fig.3/Fig.3/H/6. merge 36hpf controlMO+p53MO.tif]

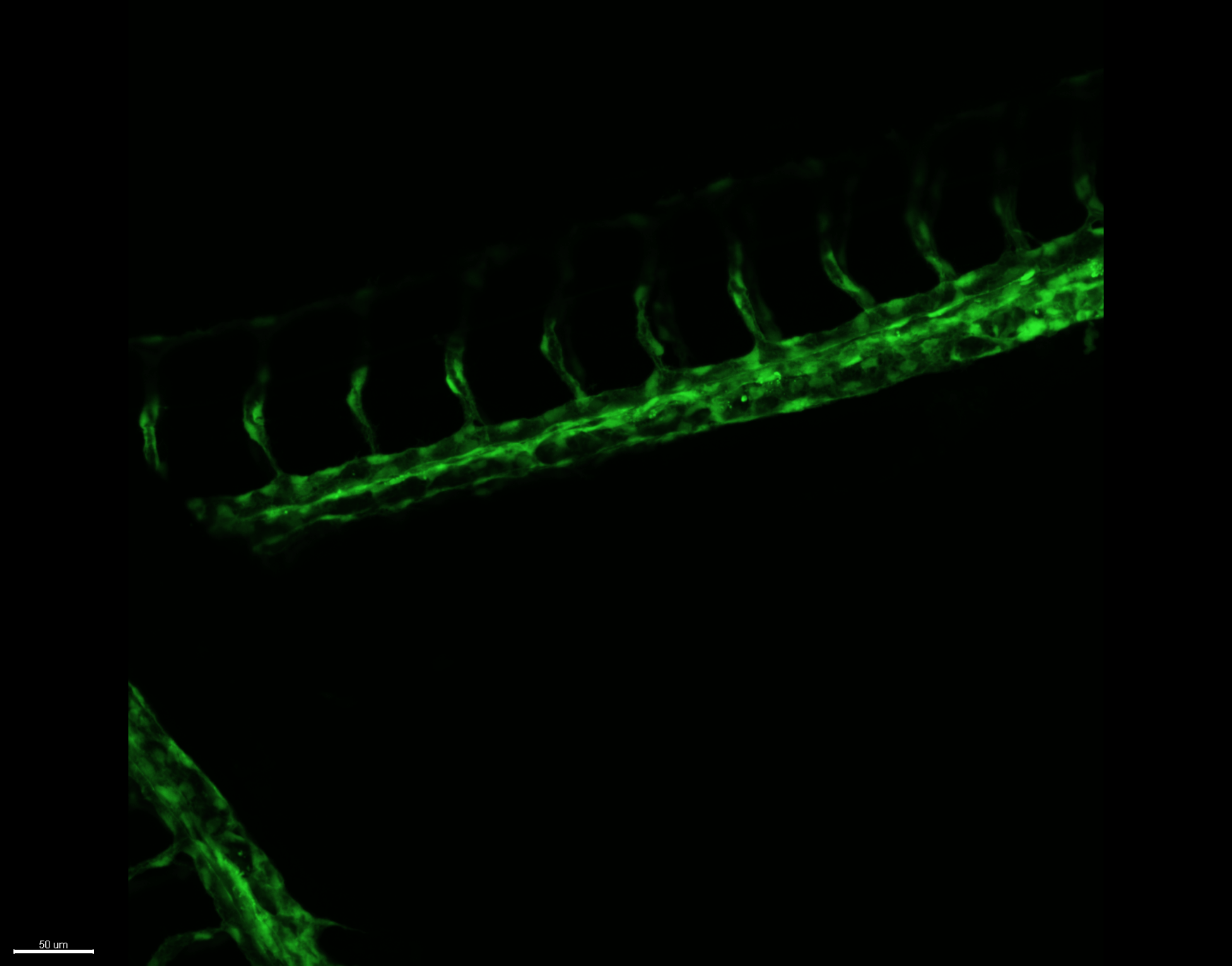

Supplement: Supplementary file 8 — Source data Fig. 3 [file 44319_2026_805_MOESM8_ESM.zip › Source Data Fig.3/Fig.3/H/7. fli1aEGFP 36hpf trmt61aMO.tif]

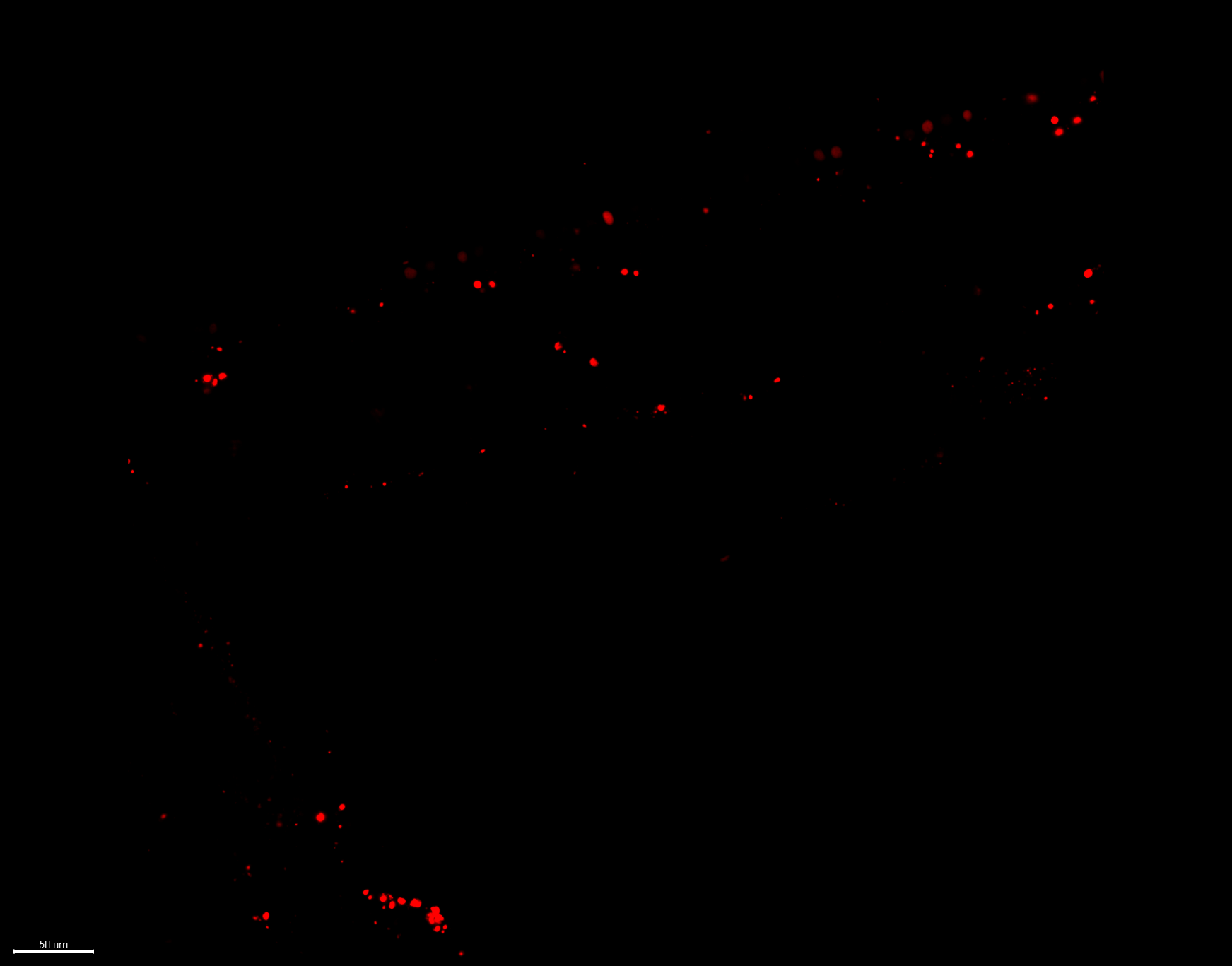

Supplement: Supplementary file 8 — Source data Fig. 3 [file 44319_2026_805_MOESM8_ESM.zip › Source Data Fig.3/Fig.3/H/8. tunel 36hpf trmt61aMO.tif]

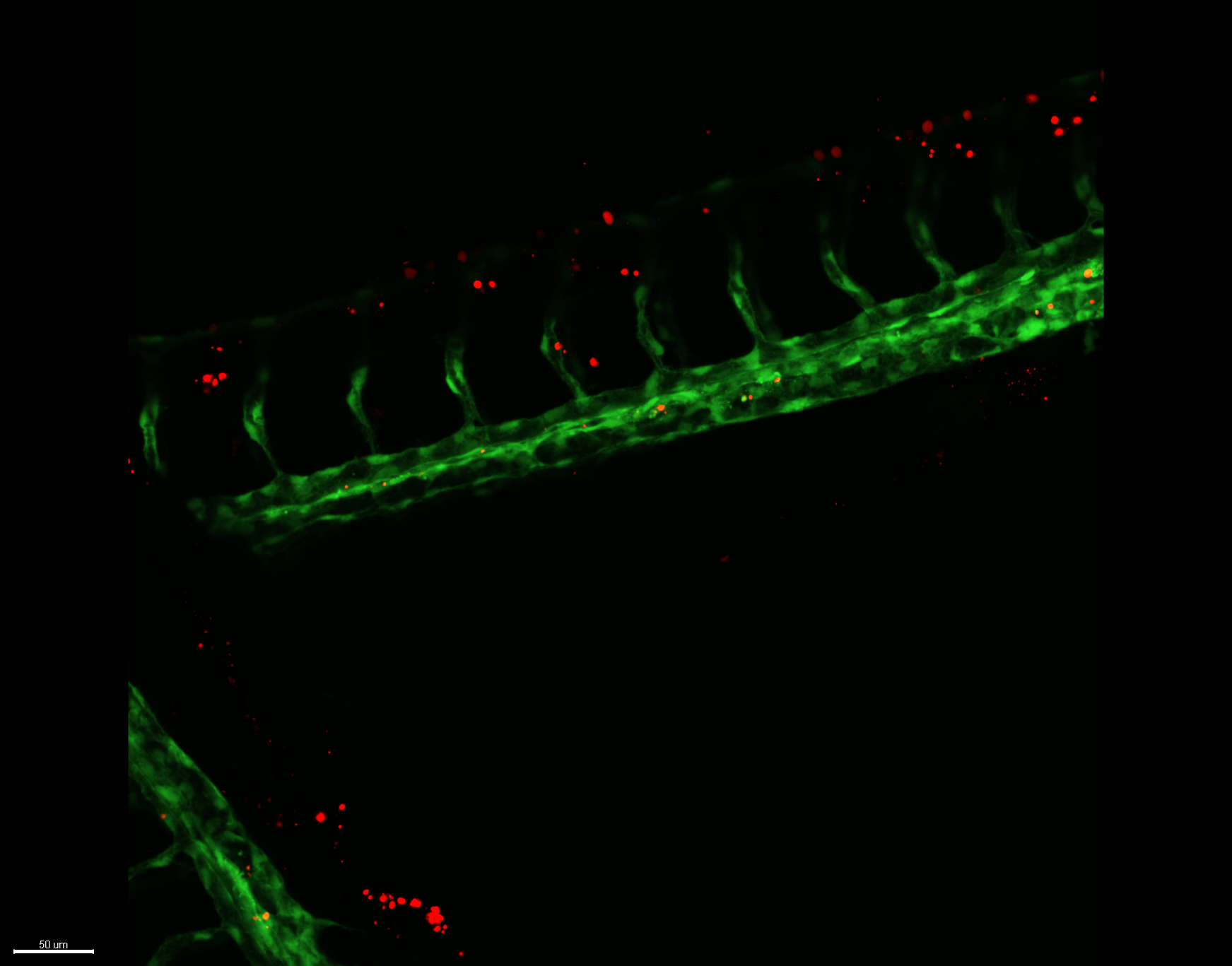

Supplement: Supplementary file 8 — Source data Fig. 3 [file 44319_2026_805_MOESM8_ESM.zip › Source Data Fig.3/Fig.3/H/9. merge 36hpf trmt61aMO.tif]
