# Supplementary figures and images for: tRNA m1A modification ensures HSPC production via modulating Nrf1 translation in zebrafish (part 2 of 3)
Source: EMBO Rep. 2026 May 27;27(13):3826–41. doi: 10.1038/s44319-026-00805-5 (PMC13354807; doi:10.1038/s44319-026-00805-5)

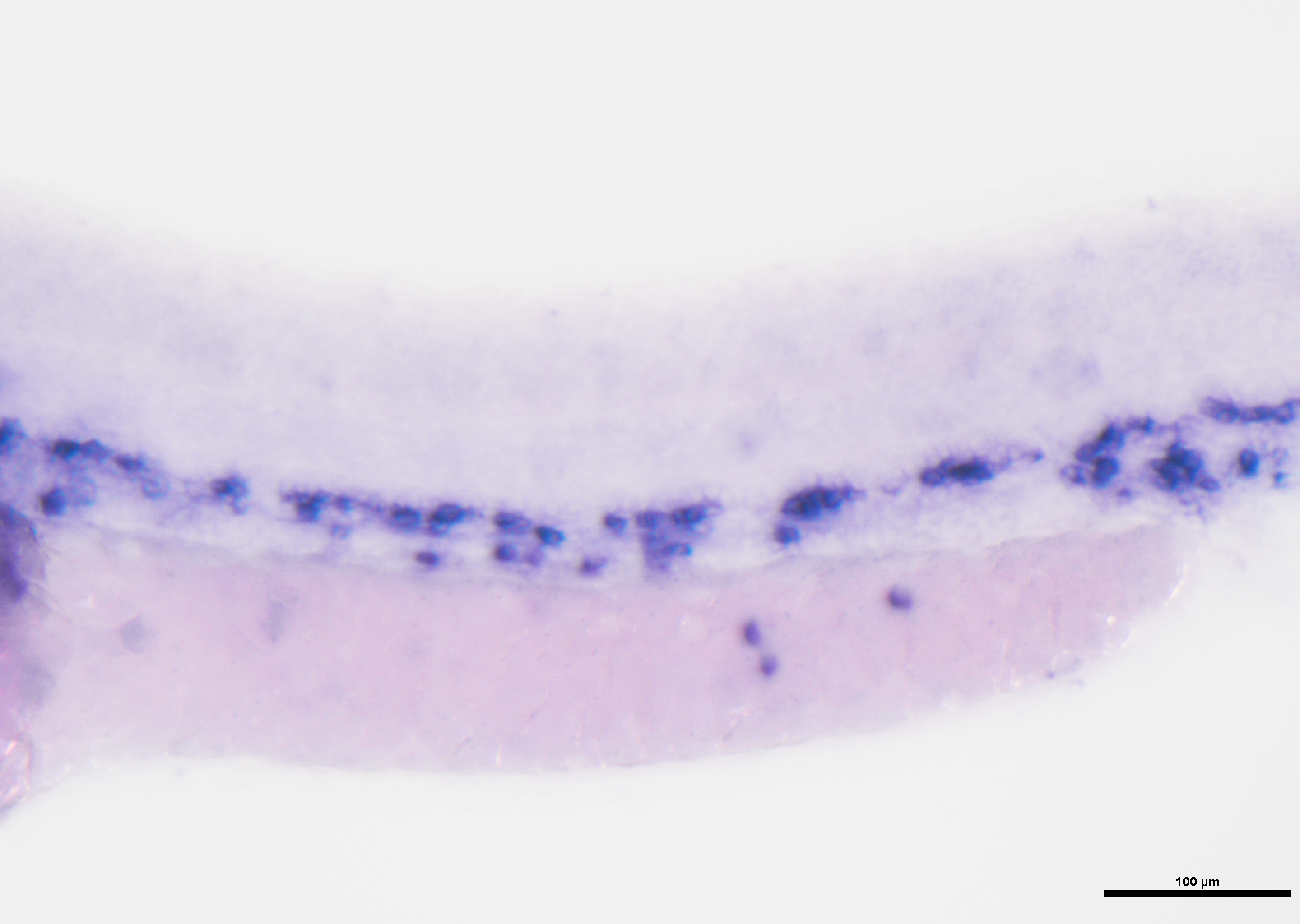

Supplement: Supplementary file 8 — Source data Fig. 3 [file 44319_2026_805_MOESM8_ESM.zip › Source Data Fig.3/Fig.3/J/1. cmyb 36hpf controlMO.tif]

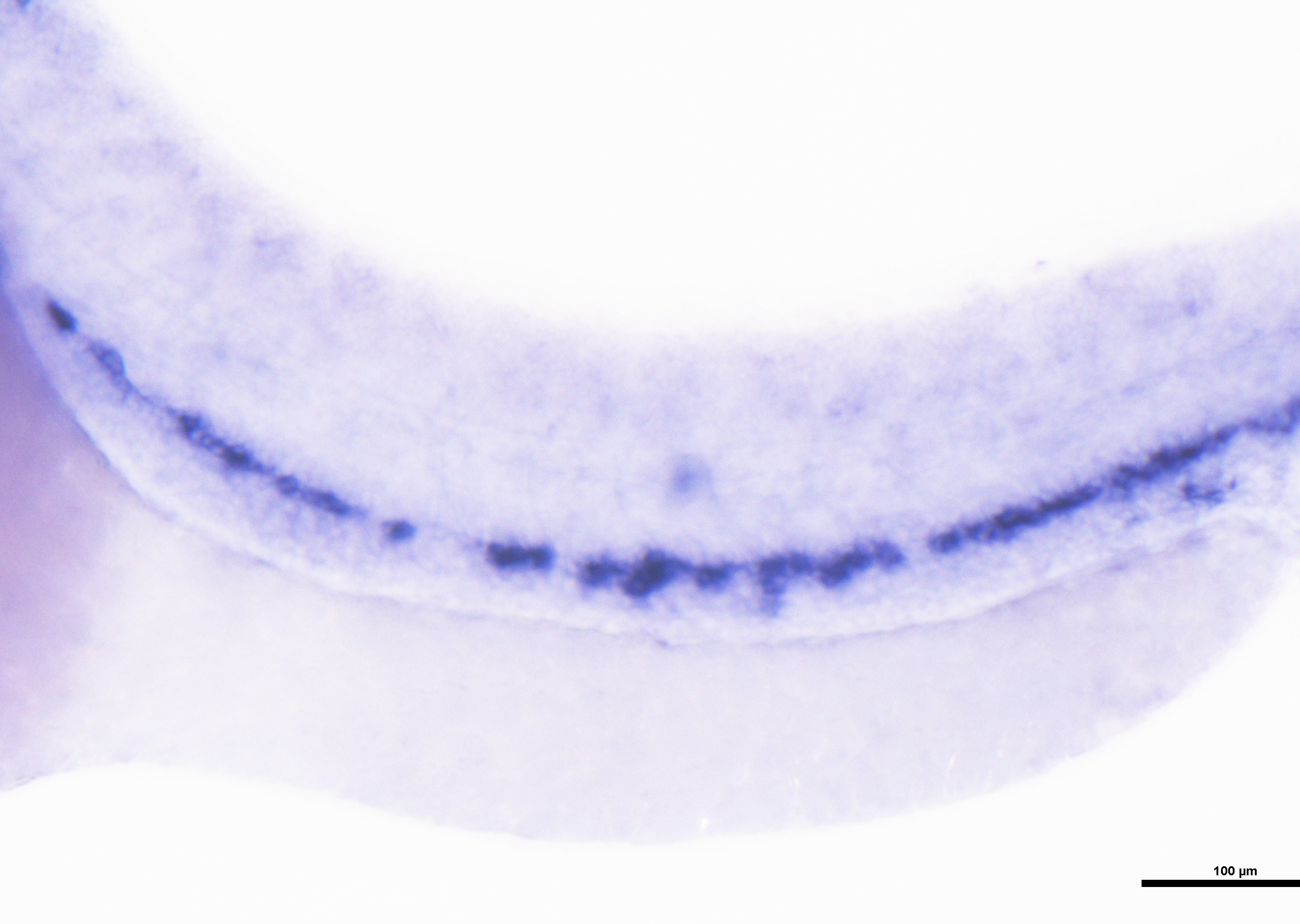

Supplement: Supplementary file 8 — Source data Fig. 3 [file 44319_2026_805_MOESM8_ESM.zip › Source Data Fig.3/Fig.3/J/2. runx1 36hpf controlMO.tif]

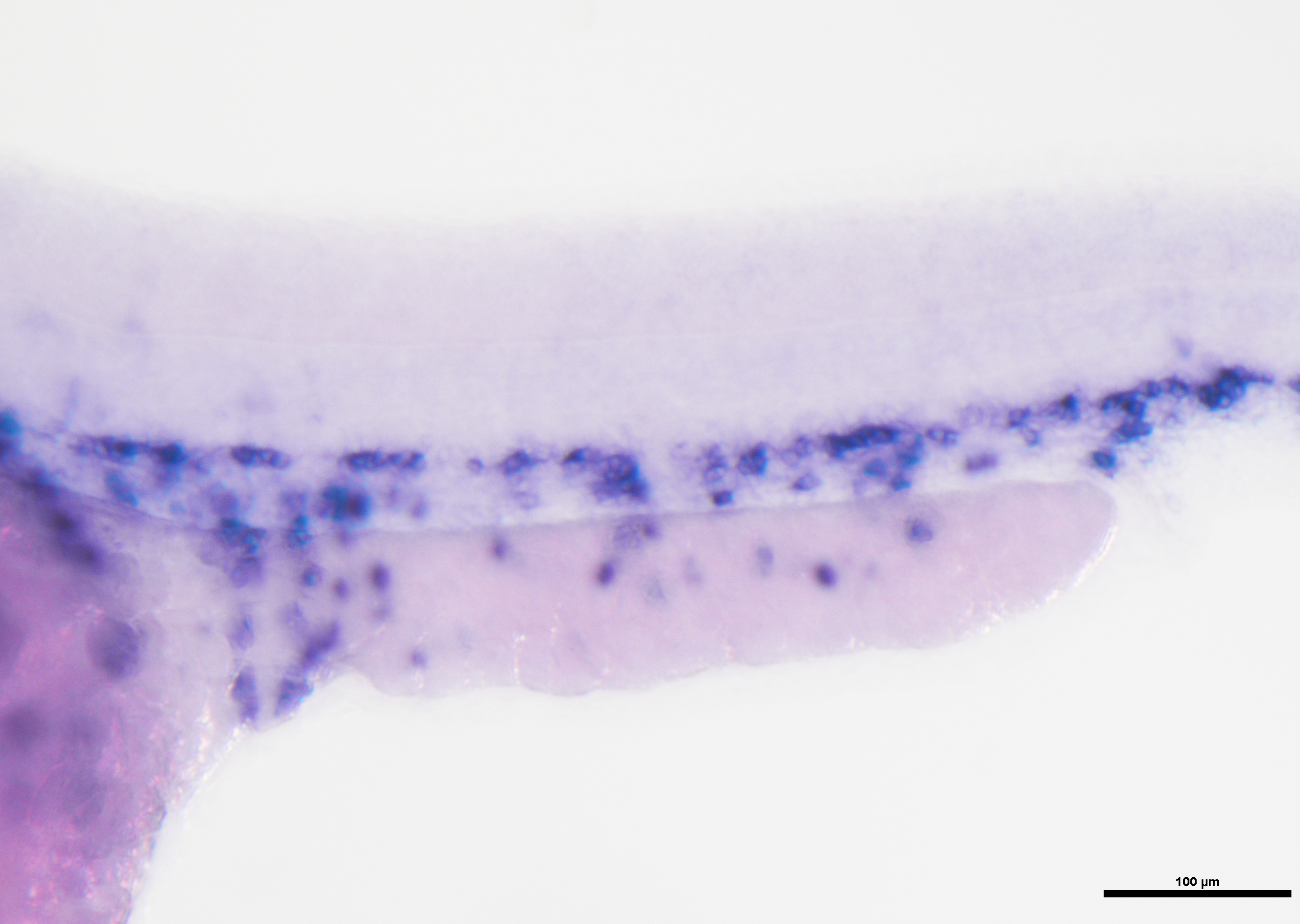

Supplement: Supplementary file 8 — Source data Fig. 3 [file 44319_2026_805_MOESM8_ESM.zip › Source Data Fig.3/Fig.3/J/3. cmyb 36hpf controlMO+p53MO.tif]

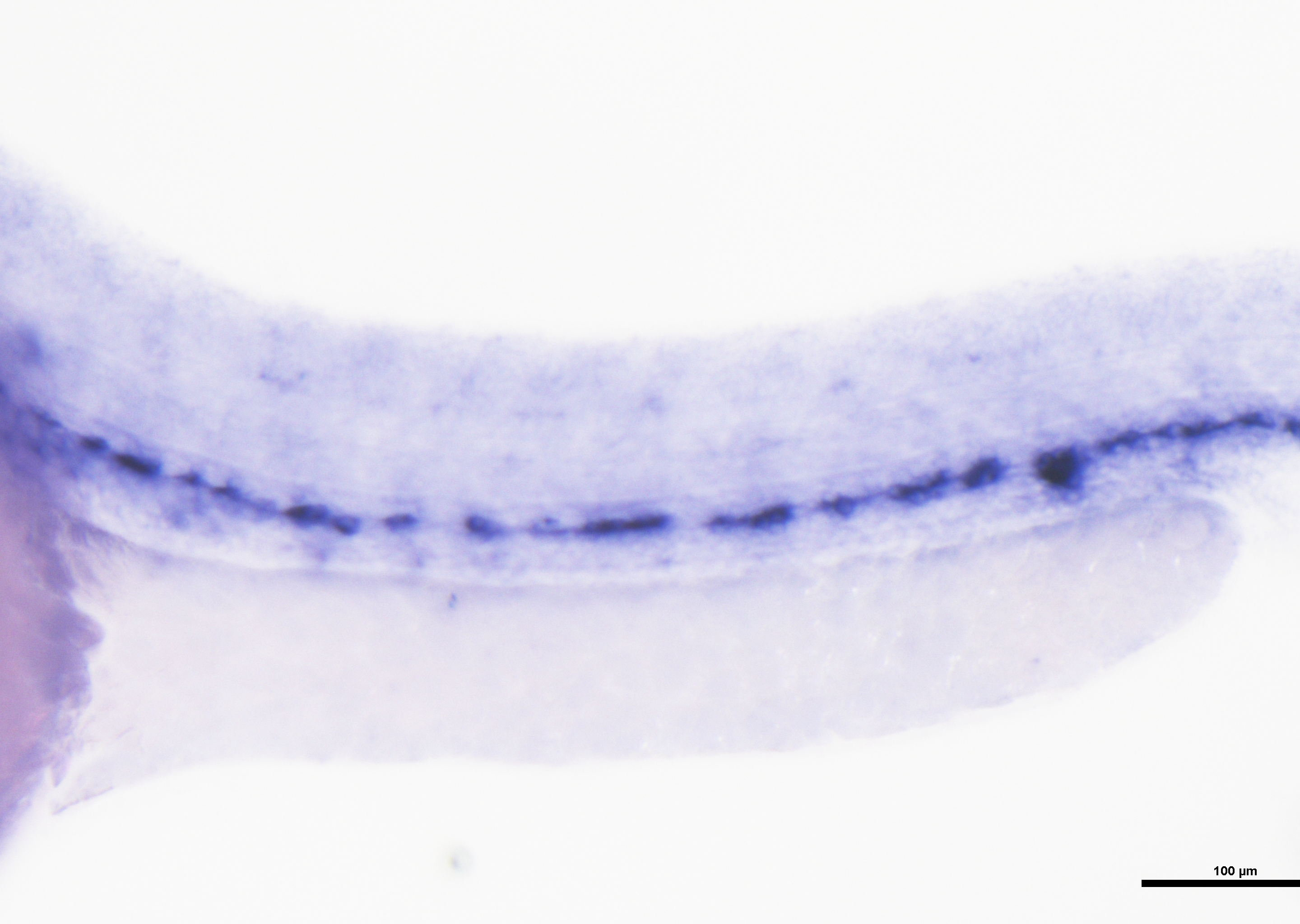

Supplement: Supplementary file 8 — Source data Fig. 3 [file 44319_2026_805_MOESM8_ESM.zip › Source Data Fig.3/Fig.3/J/4. runx1 36hpf controlMO+p53MO.tif]

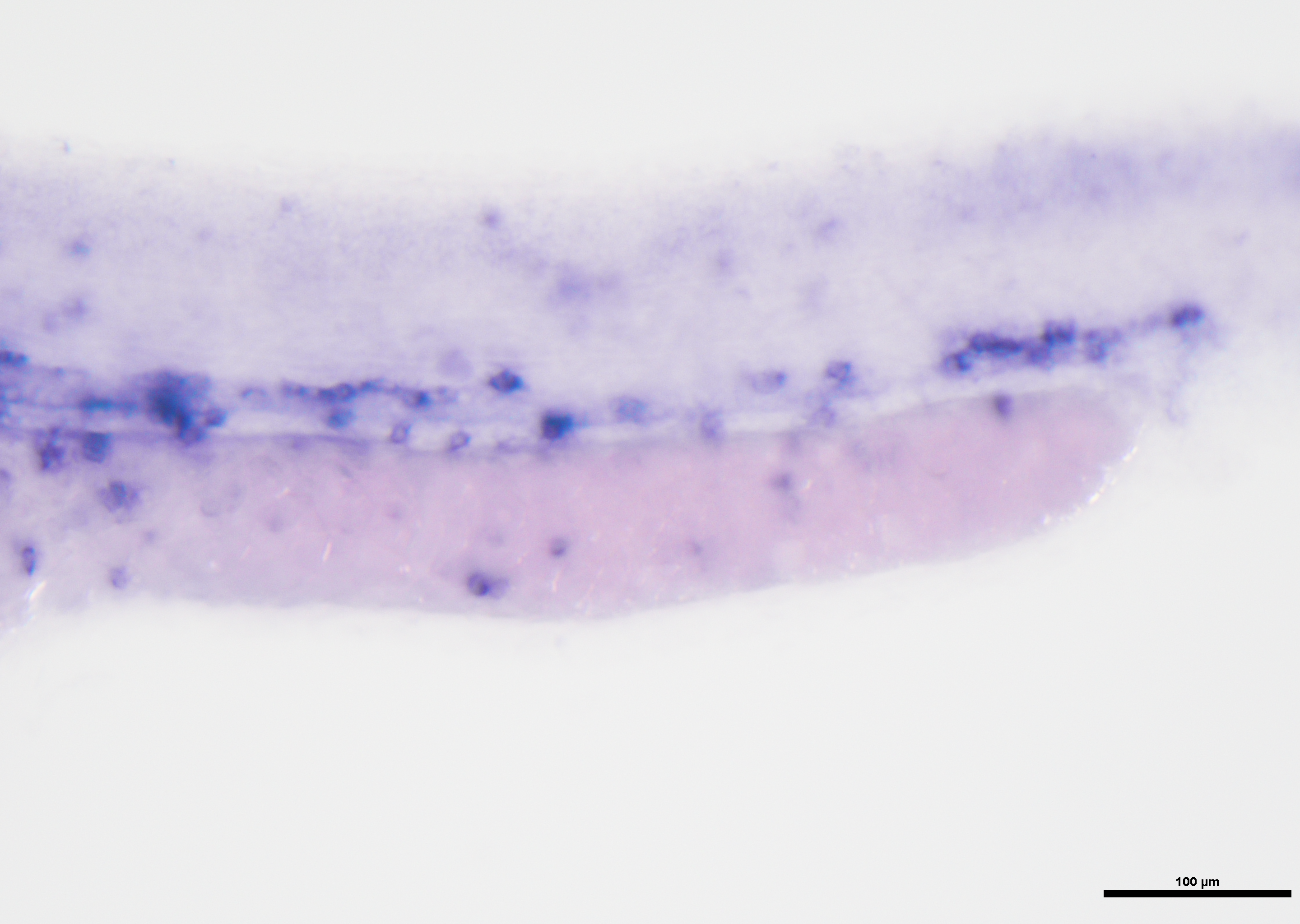

Supplement: Supplementary file 8 — Source data Fig. 3 [file 44319_2026_805_MOESM8_ESM.zip › Source Data Fig.3/Fig.3/J/5. cmyb 36hpf trmt61aMO.tif]

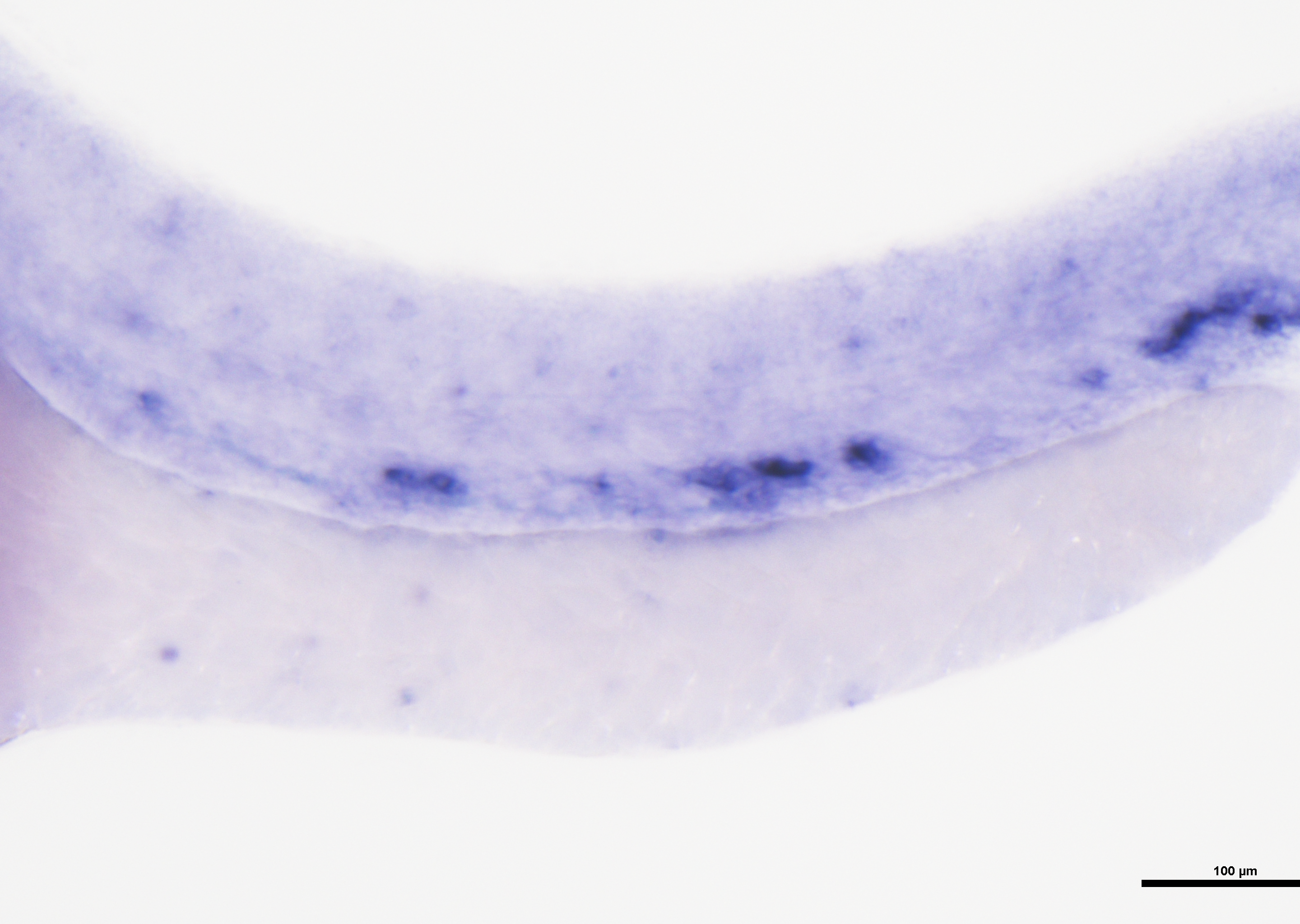

Supplement: Supplementary file 8 — Source data Fig. 3 [file 44319_2026_805_MOESM8_ESM.zip › Source Data Fig.3/Fig.3/J/6. runx1 36hpf trmt61aMO.tif]

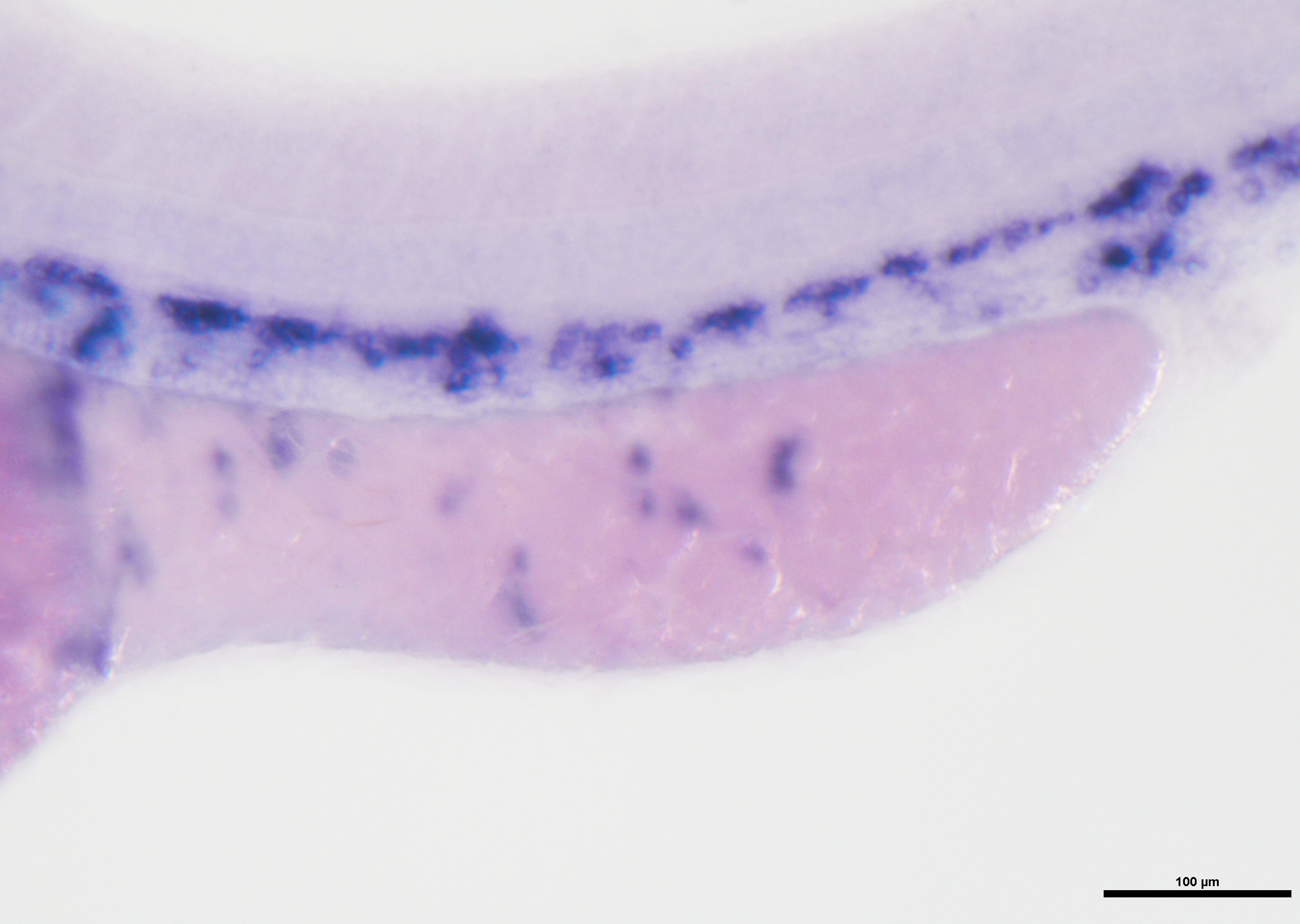

Supplement: Supplementary file 8 — Source data Fig. 3 [file 44319_2026_805_MOESM8_ESM.zip › Source Data Fig.3/Fig.3/J/7. cmyb 36hpf trmt61aMO+p53MO.tif]

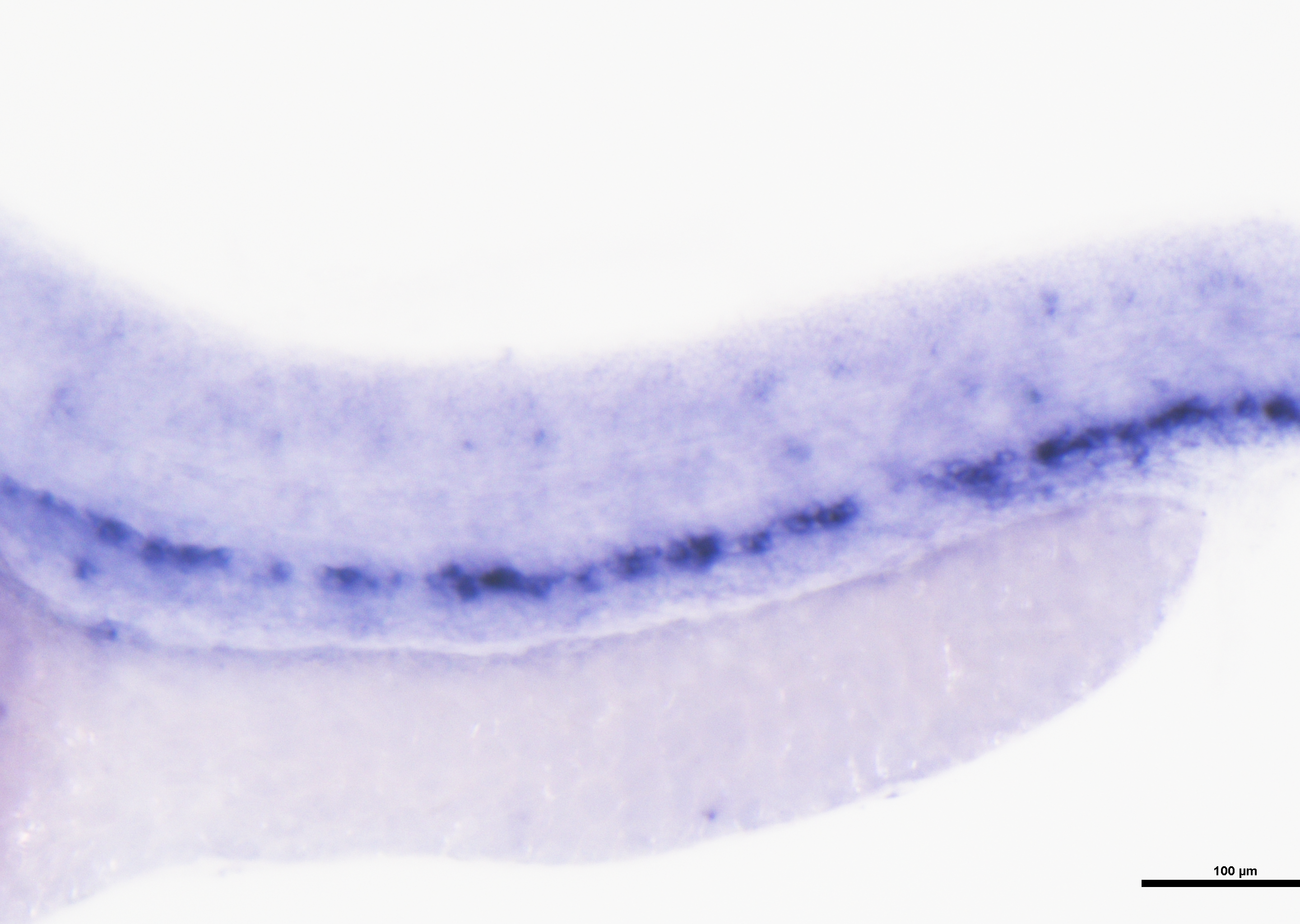

Supplement: Supplementary file 8 — Source data Fig. 3 [file 44319_2026_805_MOESM8_ESM.zip › Source Data Fig.3/Fig.3/J/8. runx1 36hpf trmt61aMO+p53MO.tif]

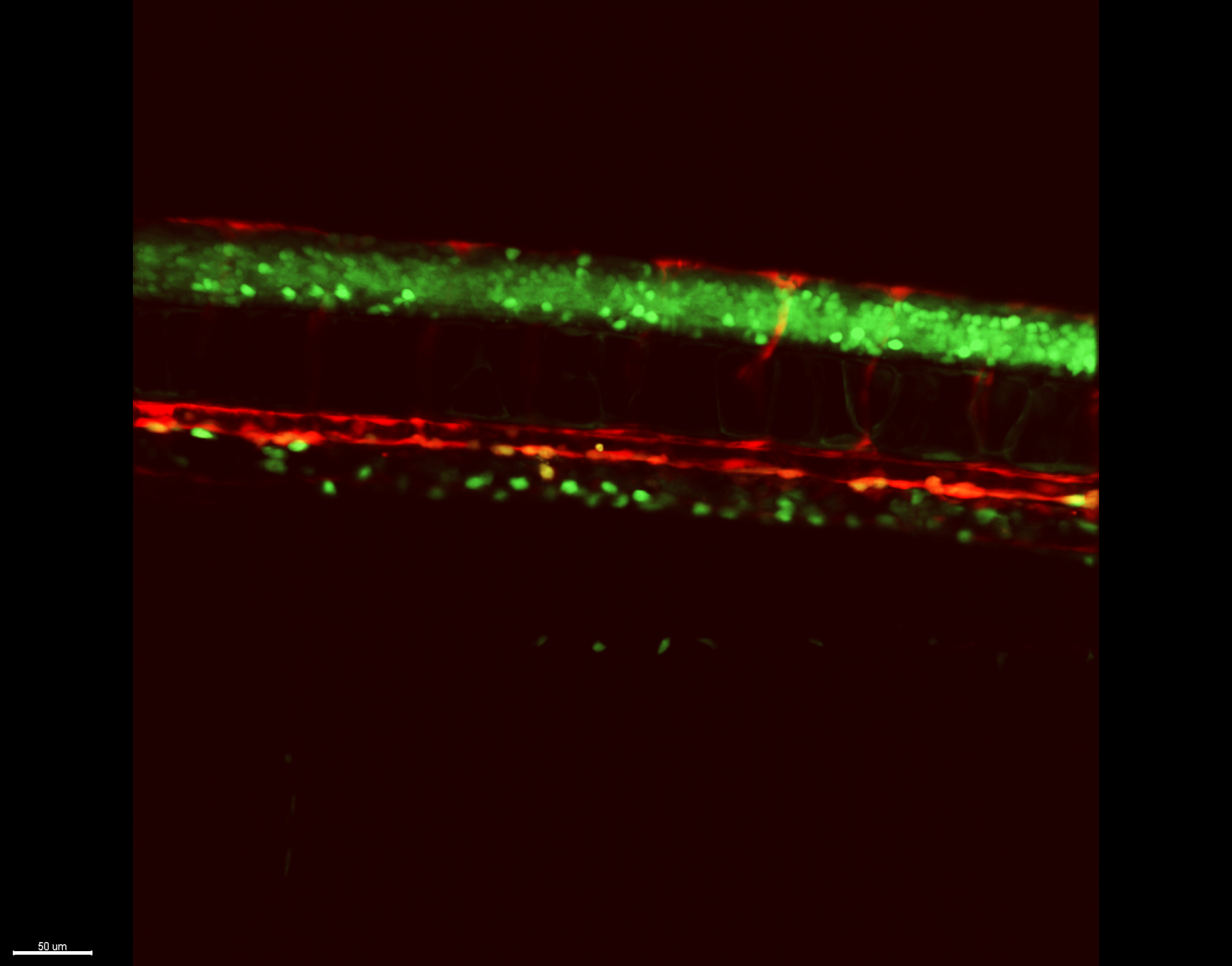

Supplement: Supplementary file 8 — Source data Fig. 3 [file 44319_2026_805_MOESM8_ESM.zip › Source Data Fig.3/Fig.3/K/1. 36hpf controlMO.tif]

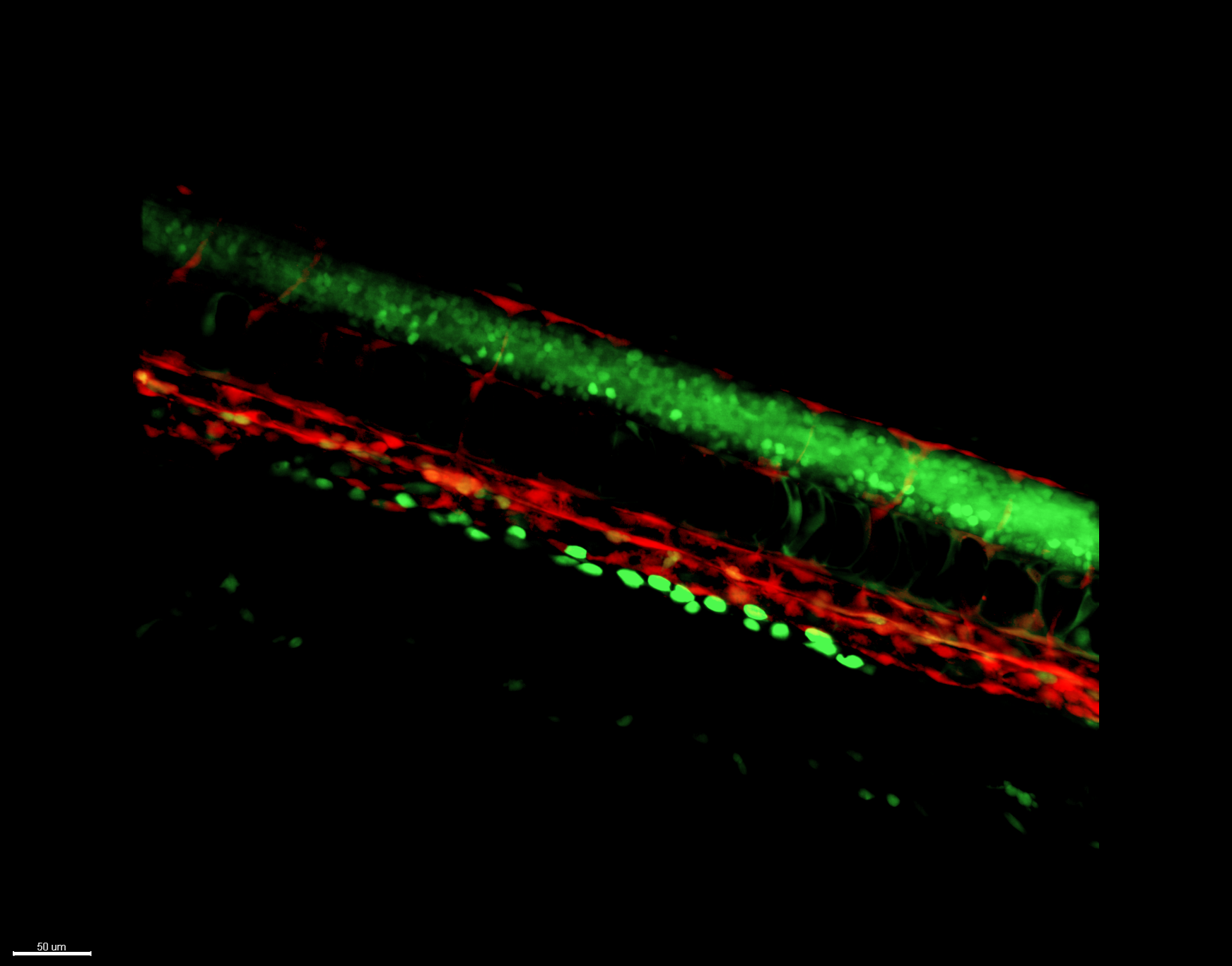

Supplement: Supplementary file 8 — Source data Fig. 3 [file 44319_2026_805_MOESM8_ESM.zip › Source Data Fig.3/Fig.3/K/2. 36hpf controlMO+p53MO.tif]

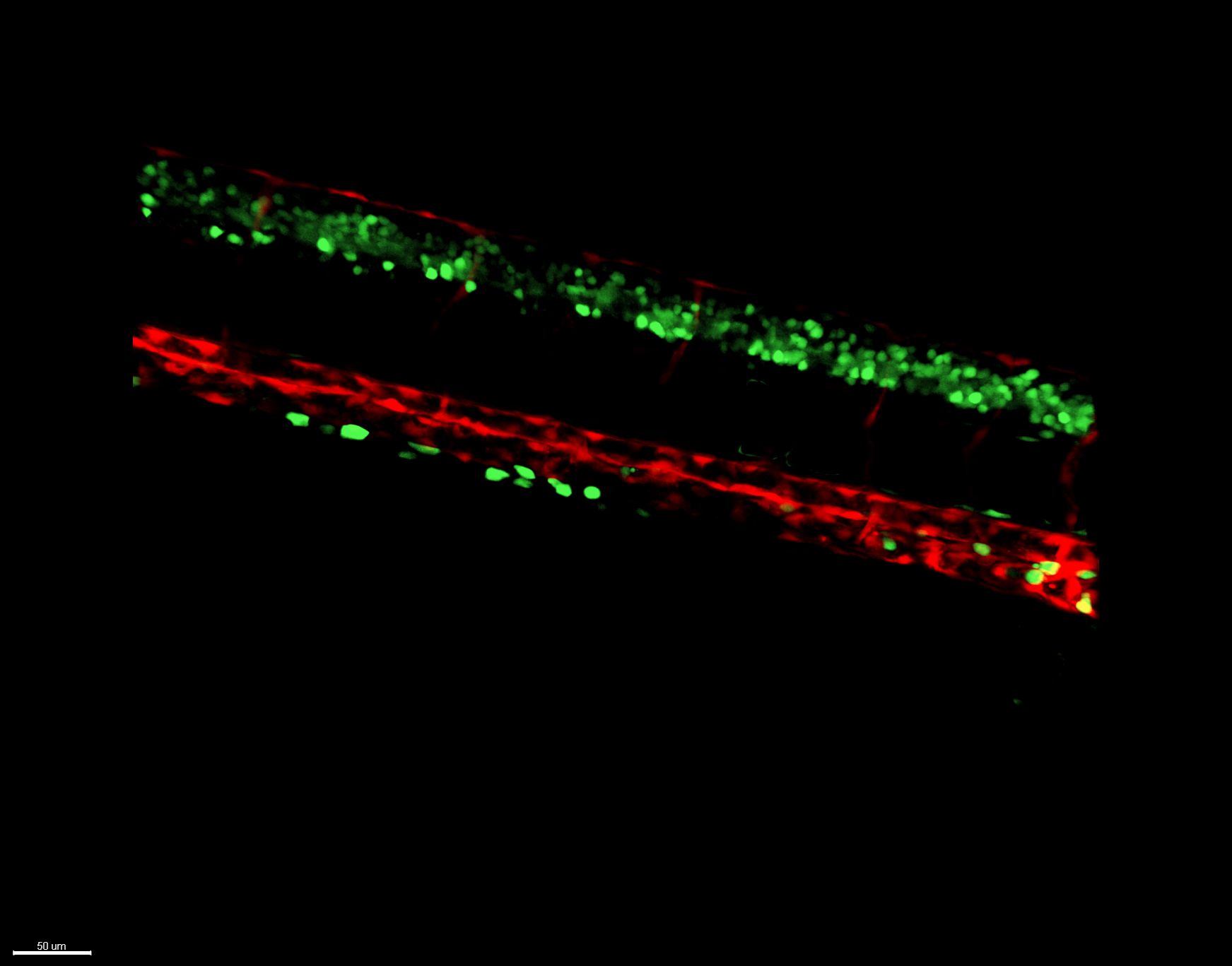

Supplement: Supplementary file 8 — Source data Fig. 3 [file 44319_2026_805_MOESM8_ESM.zip › Source Data Fig.3/Fig.3/K/3. 36hpf trmt61aMO.tif]

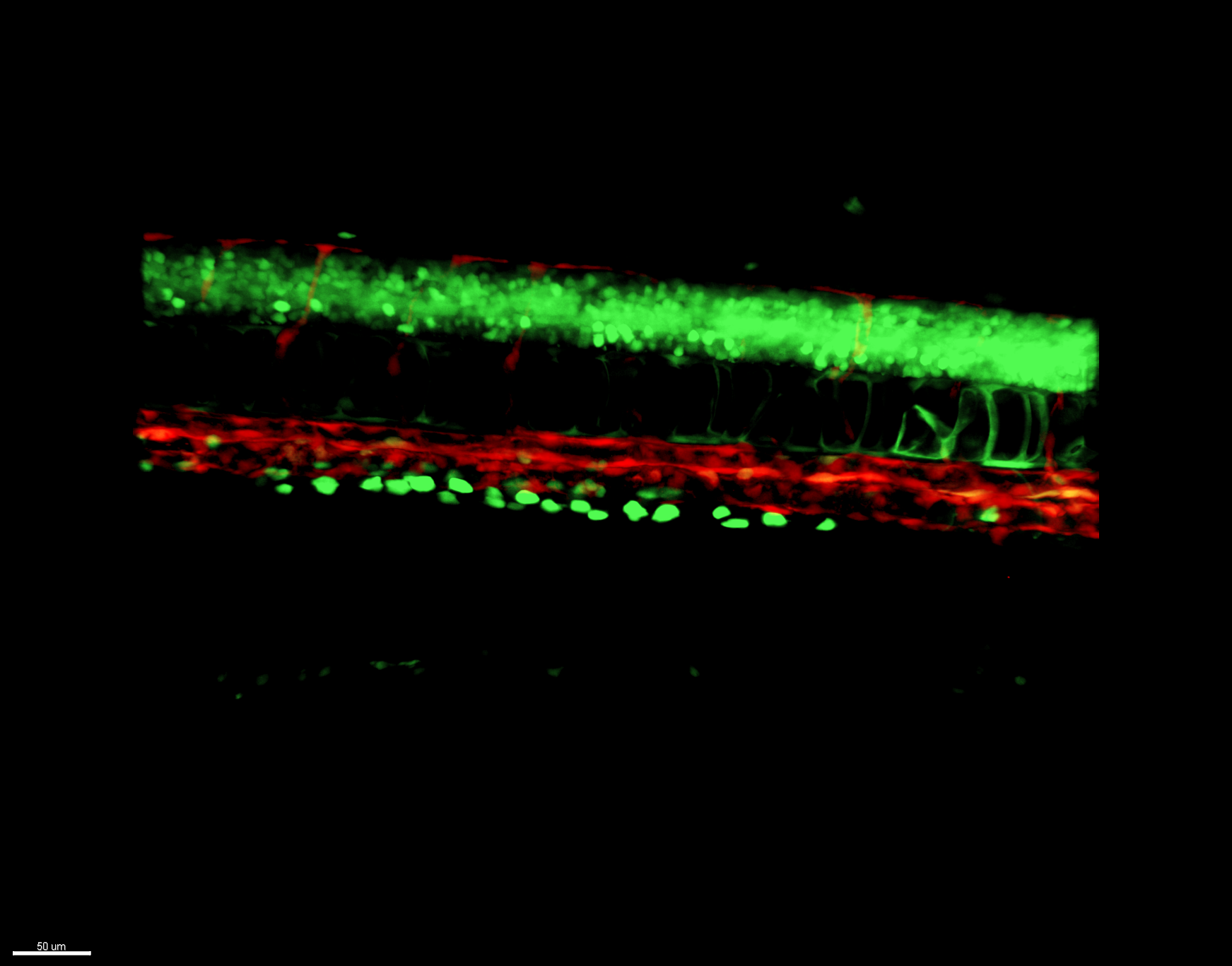

Supplement: Supplementary file 8 — Source data Fig. 3 [file 44319_2026_805_MOESM8_ESM.zip › Source Data Fig.3/Fig.3/K/4. 36hpf trmt61aMO+P53mo.tif]

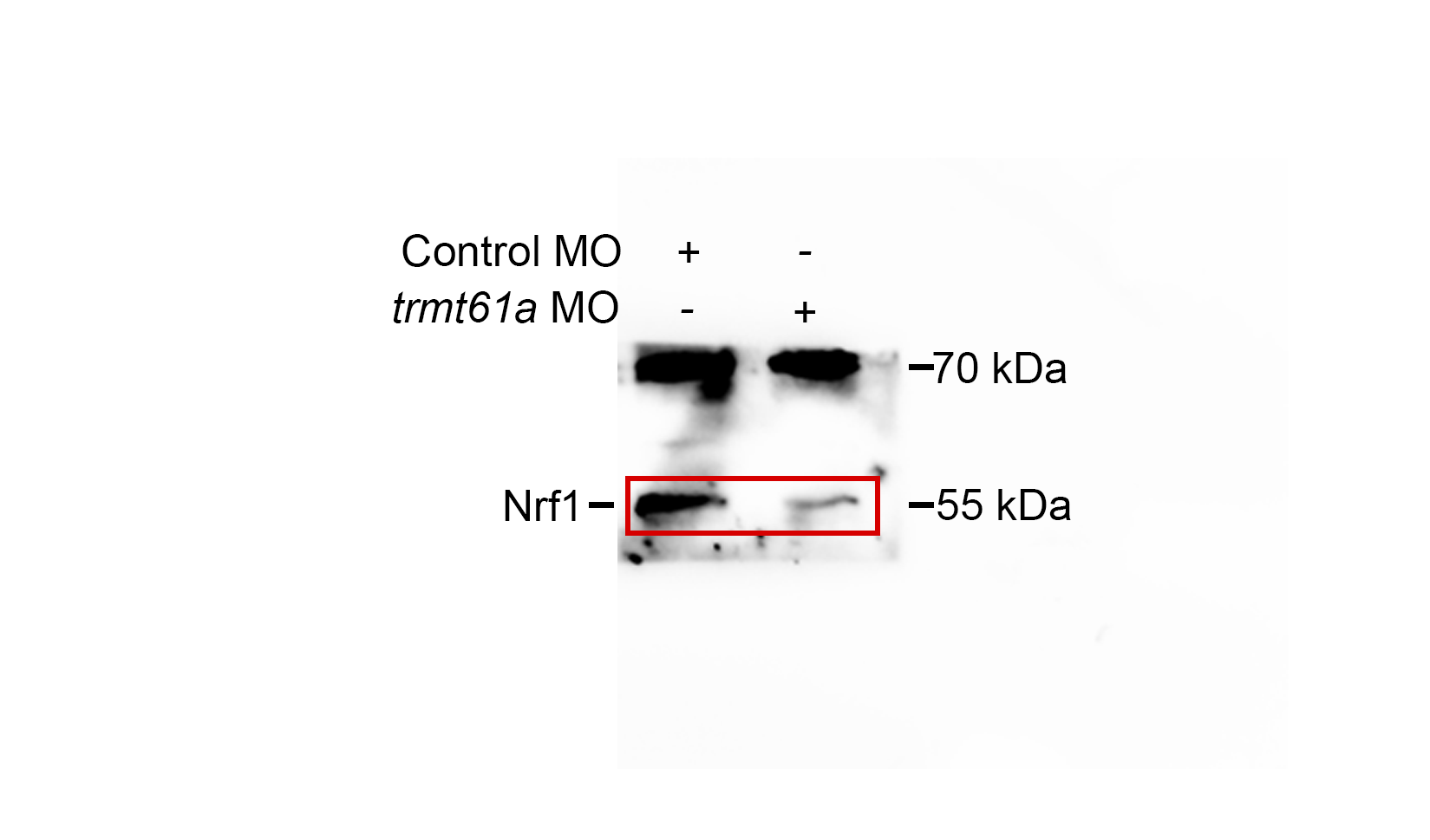

Supplement: Supplementary file 9 — Source data Fig. 4 [file 44319_2026_805_MOESM9_ESM.zip › Source Data Fig.4/Fig.4/G/4G_Nrf1 WB .tif]

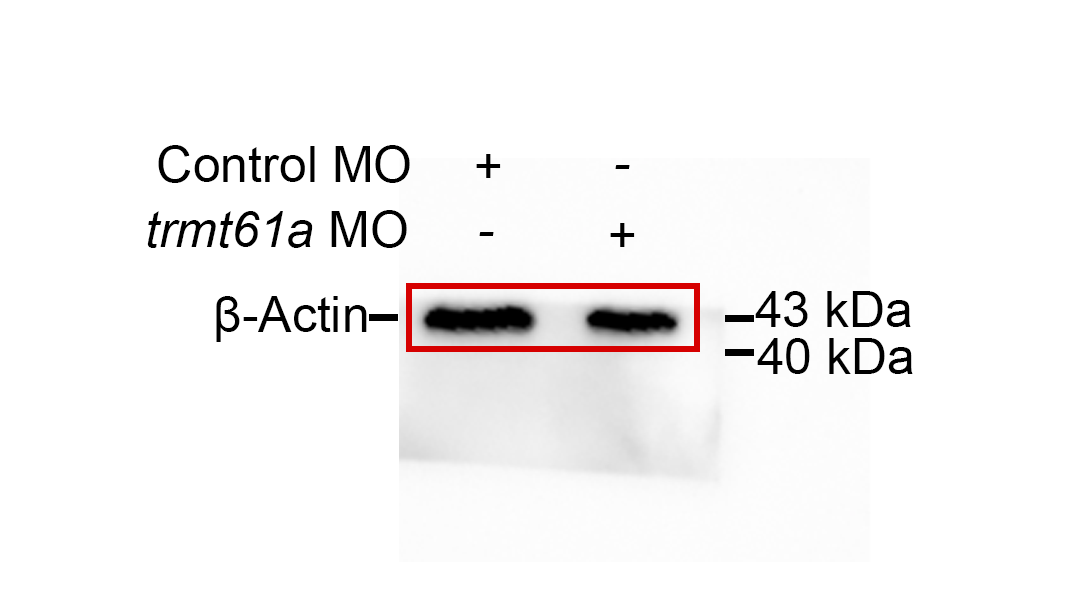

Supplement: Supplementary file 9 — Source data Fig. 4 [file 44319_2026_805_MOESM9_ESM.zip › Source Data Fig.4/Fig.4/G/4G_β-Actin WB.tif]

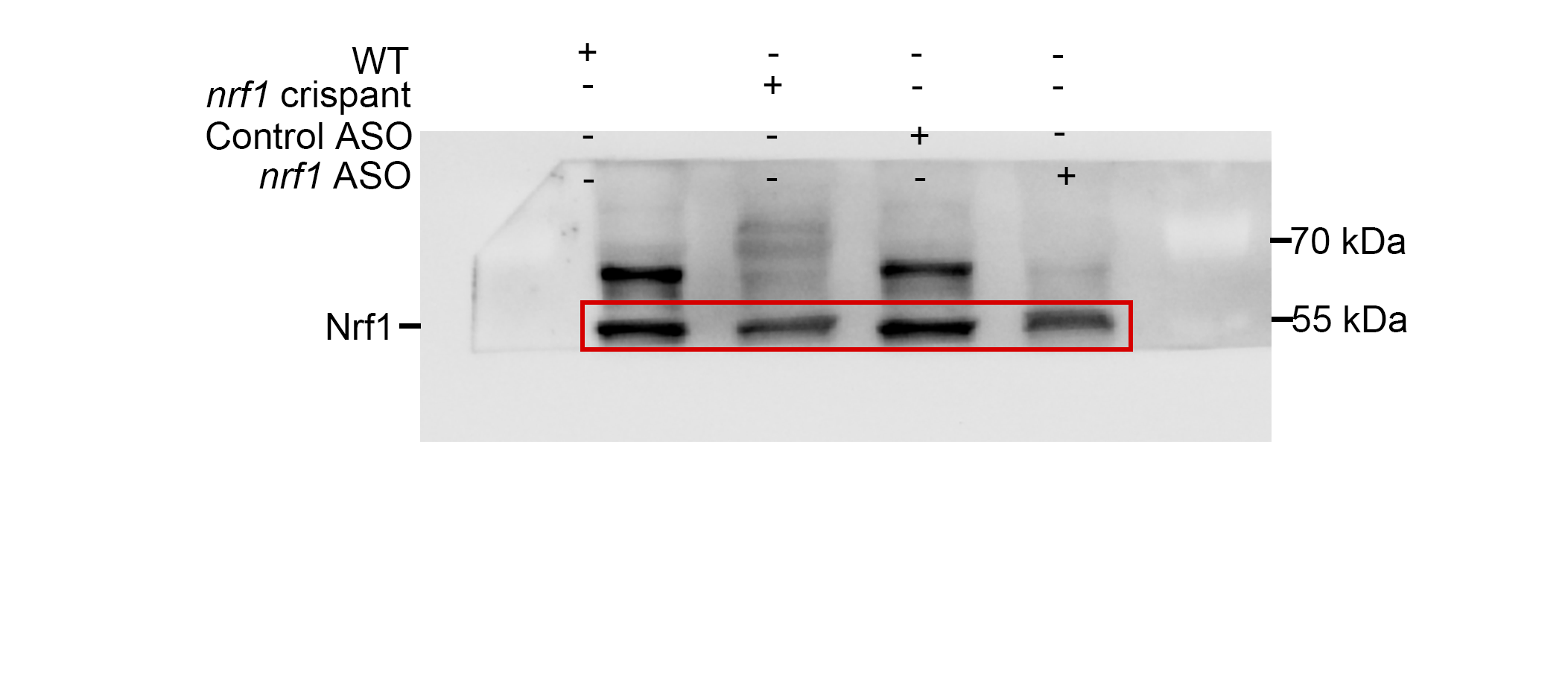

Supplement: Supplementary file 9 — Source data Fig. 4 [file 44319_2026_805_MOESM9_ESM.zip › Source Data Fig.4/Fig.4/H/4H_Nrf1 WB.tif]

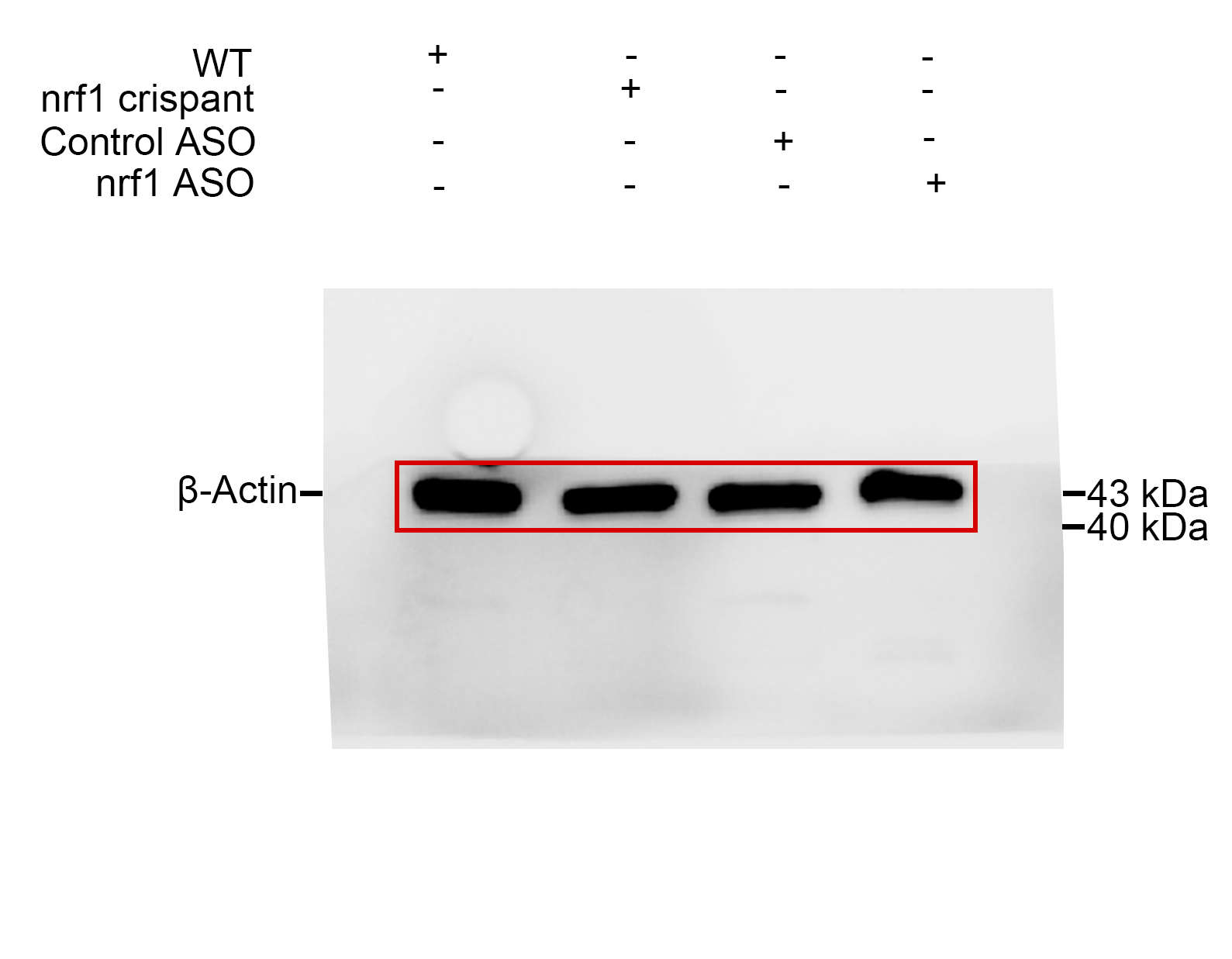

Supplement: Supplementary file 9 — Source data Fig. 4 [file 44319_2026_805_MOESM9_ESM.zip › Source Data Fig.4/Fig.4/H/4H_β-Actin WB.tif]

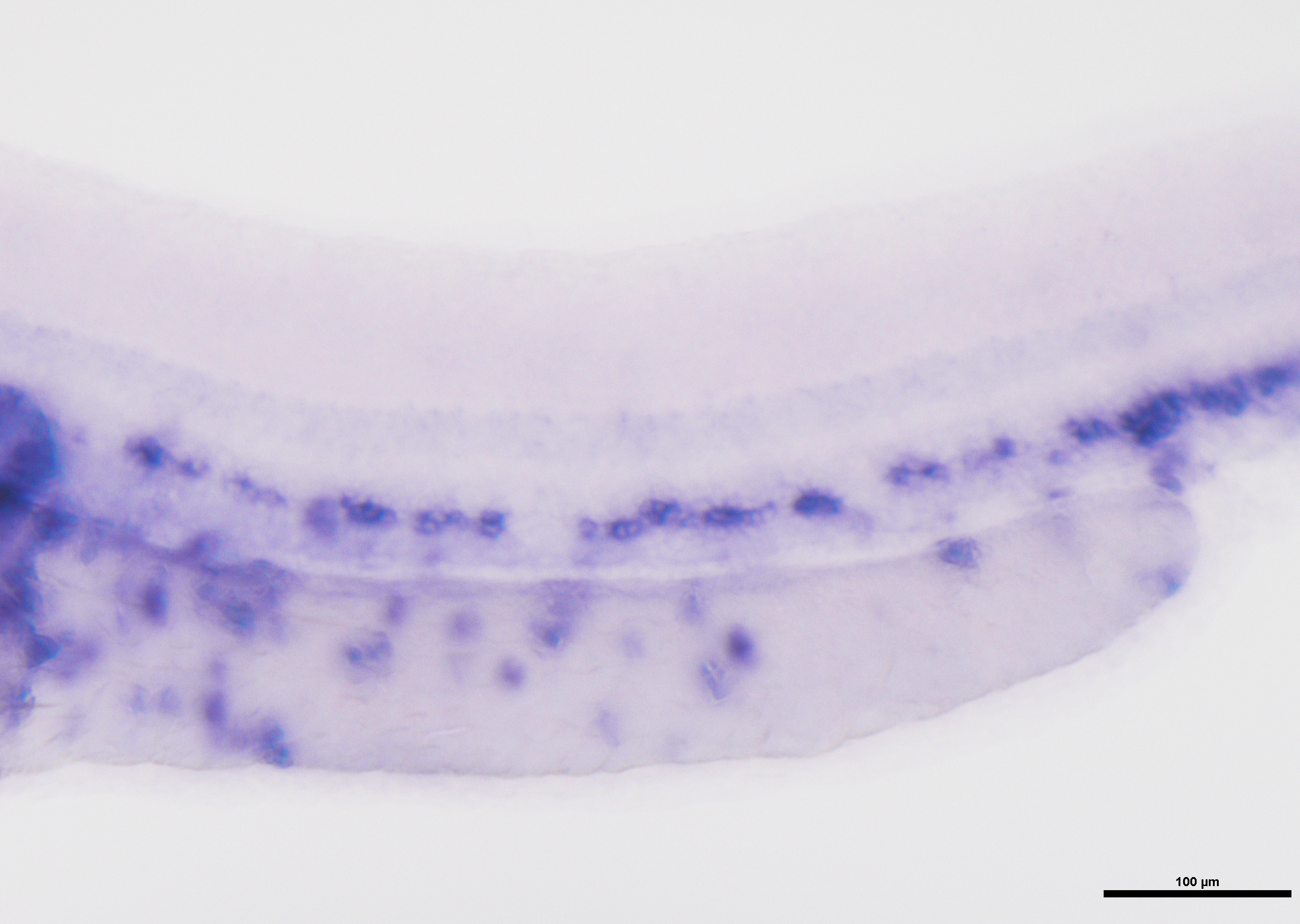

Supplement: Supplementary file 9 — Source data Fig. 4 [file 44319_2026_805_MOESM9_ESM.zip › Source Data Fig.4/Fig.4/K/1. cmyb 36hpf WT.tif]

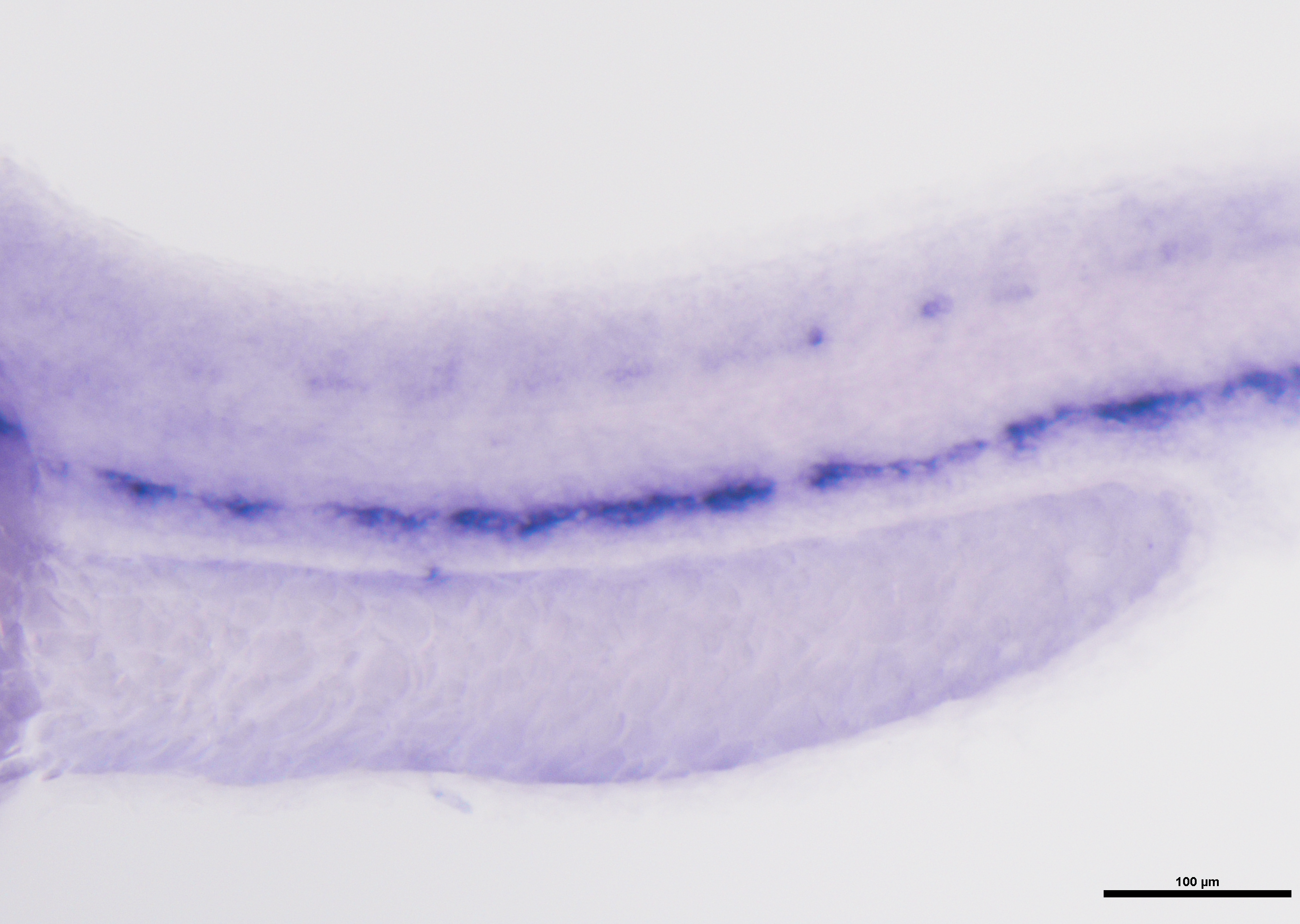

Supplement: Supplementary file 9 — Source data Fig. 4 [file 44319_2026_805_MOESM9_ESM.zip › Source Data Fig.4/Fig.4/K/2. runx1 36hpf WT.tif]

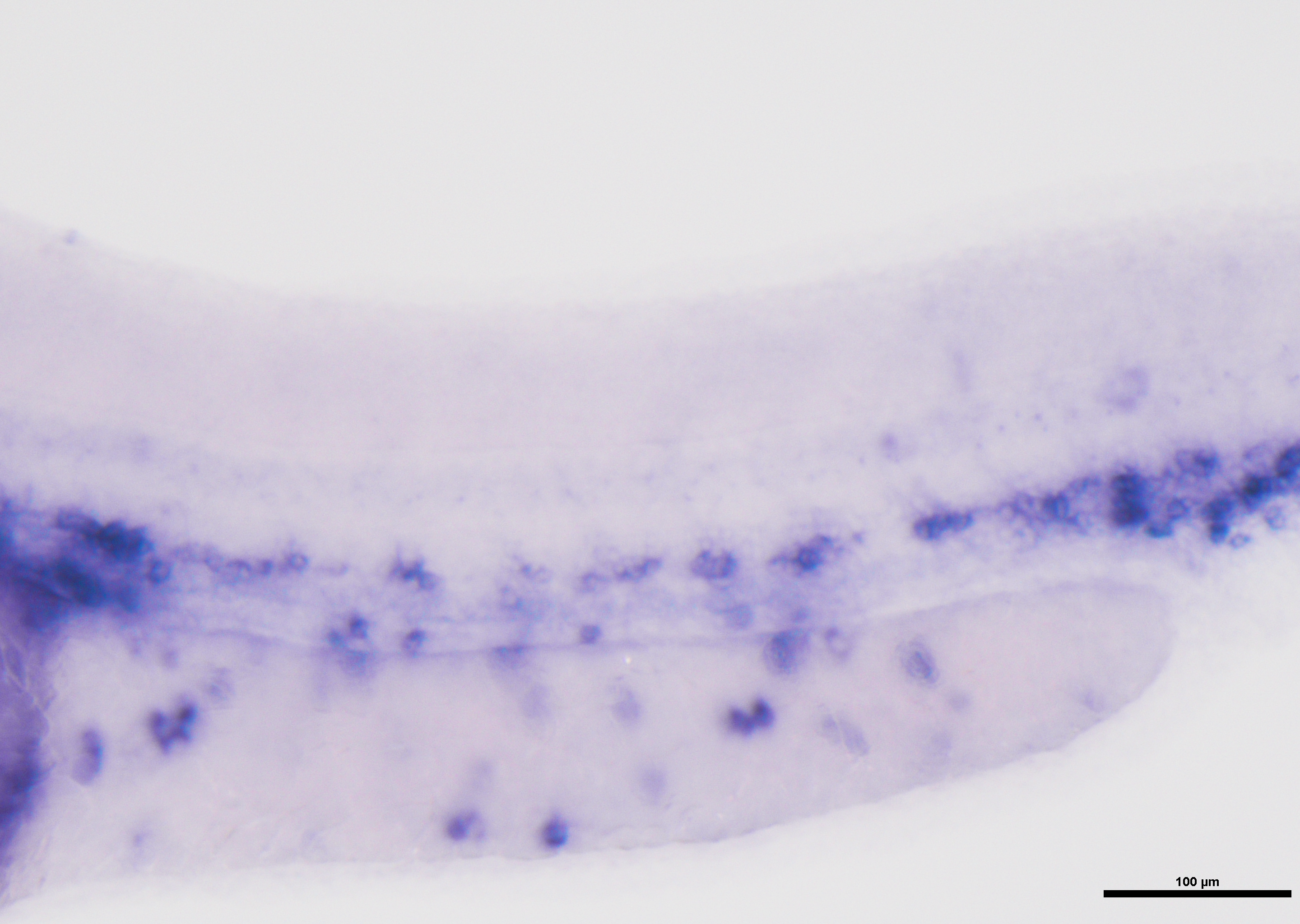

Supplement: Supplementary file 9 — Source data Fig. 4 [file 44319_2026_805_MOESM9_ESM.zip › Source Data Fig.4/Fig.4/K/3. cmyb 36hpf nrf1crispant.tif]

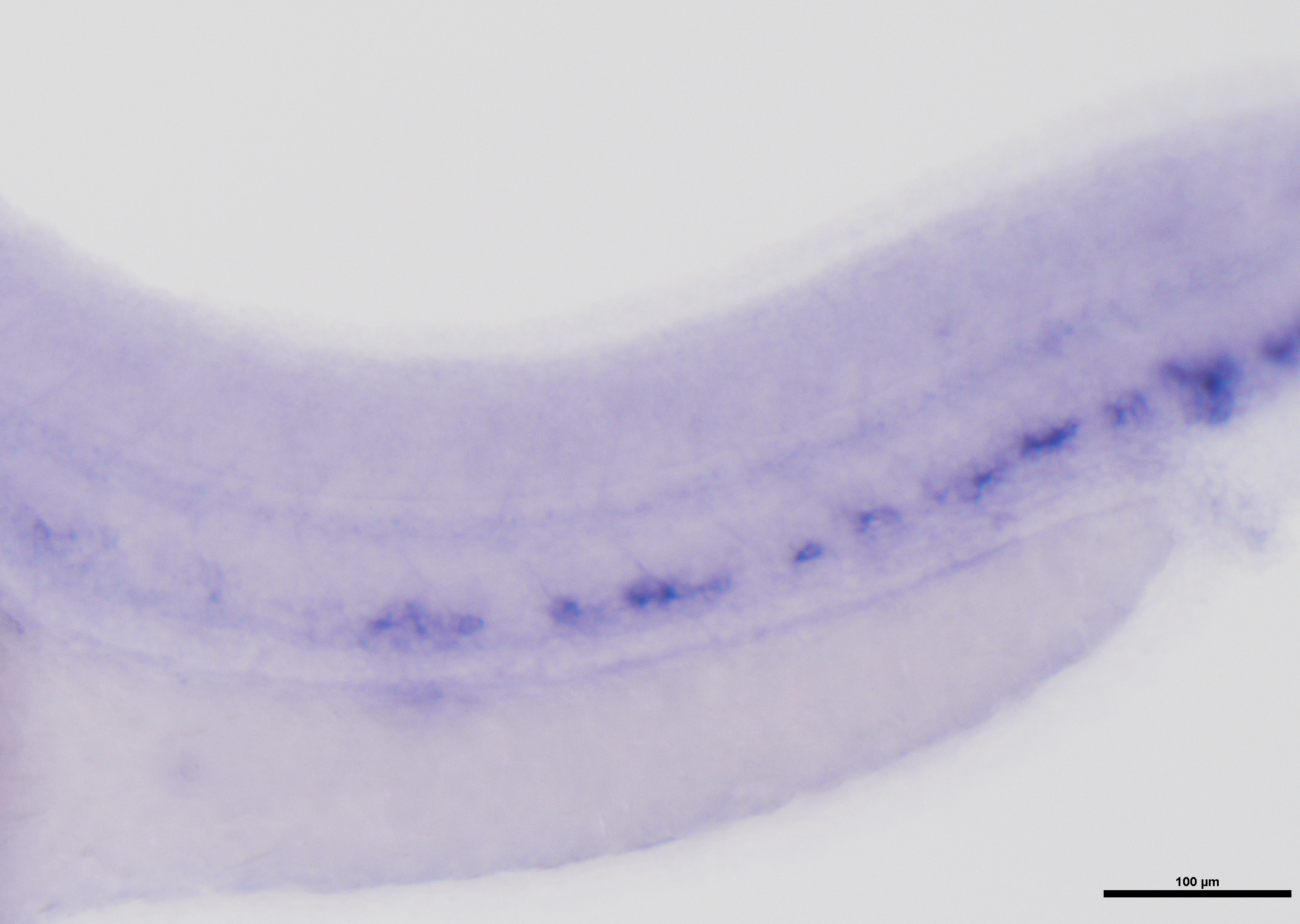

Supplement: Supplementary file 9 — Source data Fig. 4 [file 44319_2026_805_MOESM9_ESM.zip › Source Data Fig.4/Fig.4/K/4. runx1 36hpf nrf1crispant.tif]

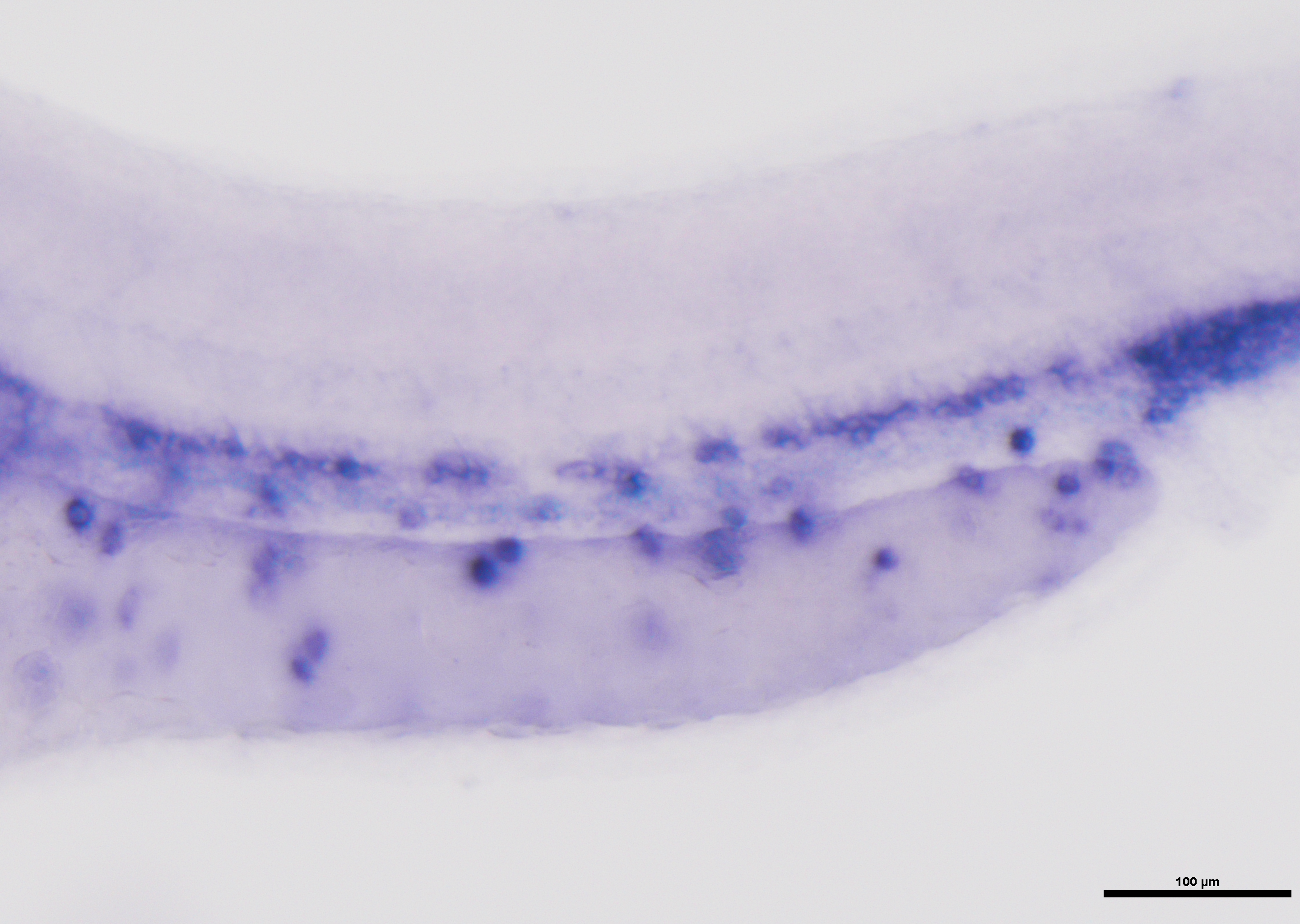

Supplement: Supplementary file 9 — Source data Fig. 4 [file 44319_2026_805_MOESM9_ESM.zip › Source Data Fig.4/Fig.4/K/5. cmyb 36hpf controlASO.tif]

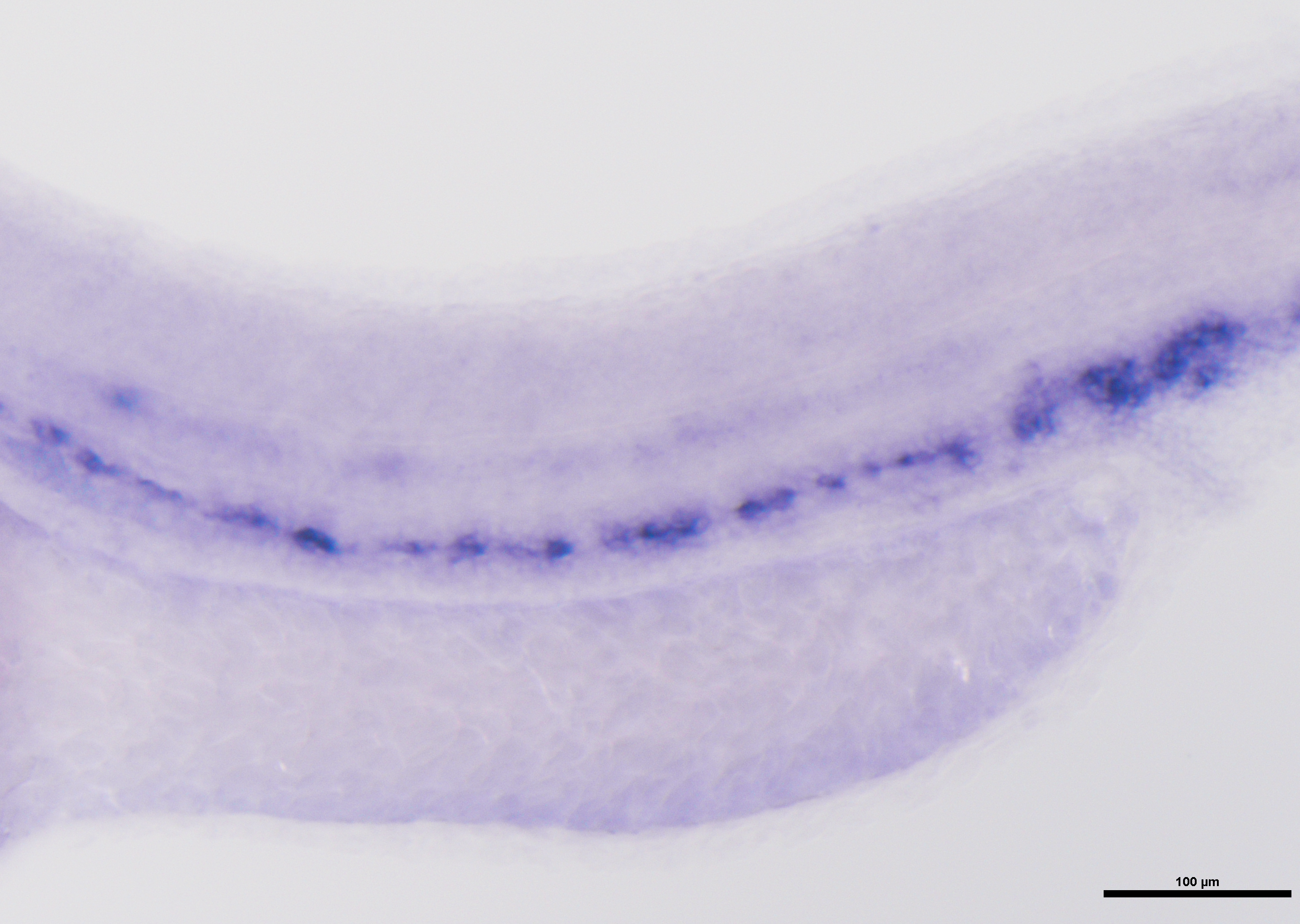

Supplement: Supplementary file 9 — Source data Fig. 4 [file 44319_2026_805_MOESM9_ESM.zip › Source Data Fig.4/Fig.4/K/6. runx1 36hpf controlASO.tif]

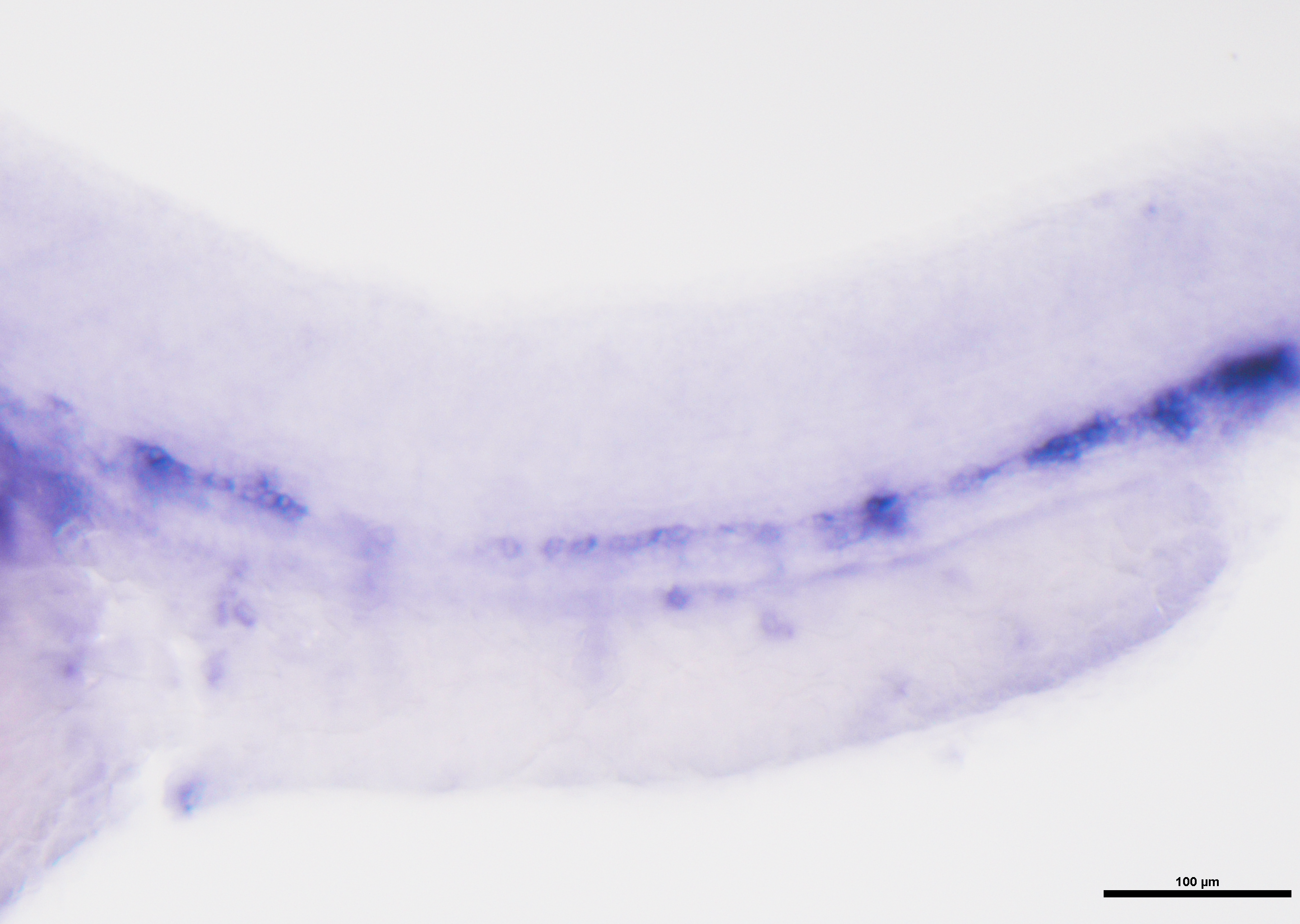

Supplement: Supplementary file 9 — Source data Fig. 4 [file 44319_2026_805_MOESM9_ESM.zip › Source Data Fig.4/Fig.4/K/7. cmyb 36hpf nrf1ASO.tif]

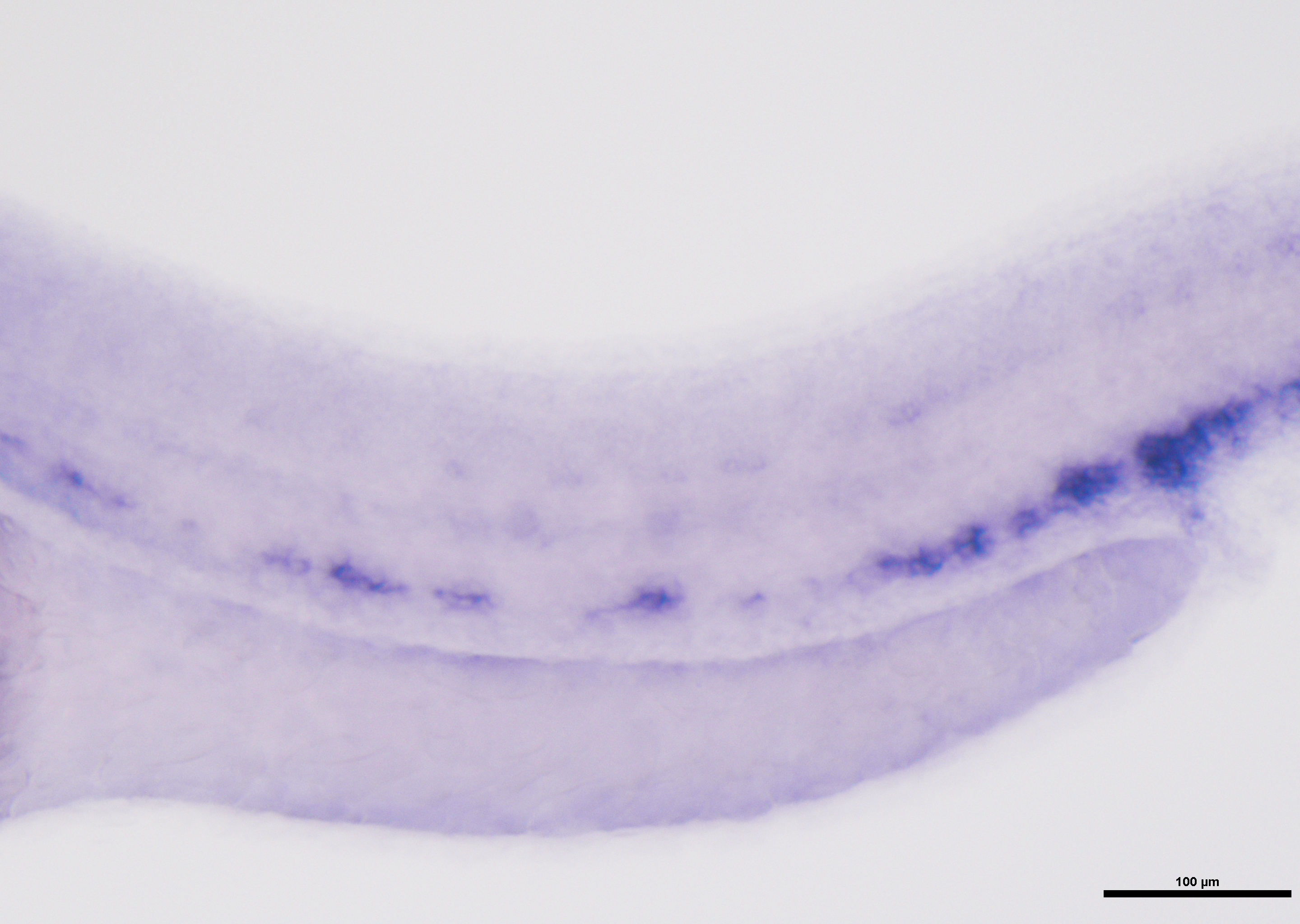

Supplement: Supplementary file 9 — Source data Fig. 4 [file 44319_2026_805_MOESM9_ESM.zip › Source Data Fig.4/Fig.4/K/8. runx1 36hpf nrf1ASO.tif]

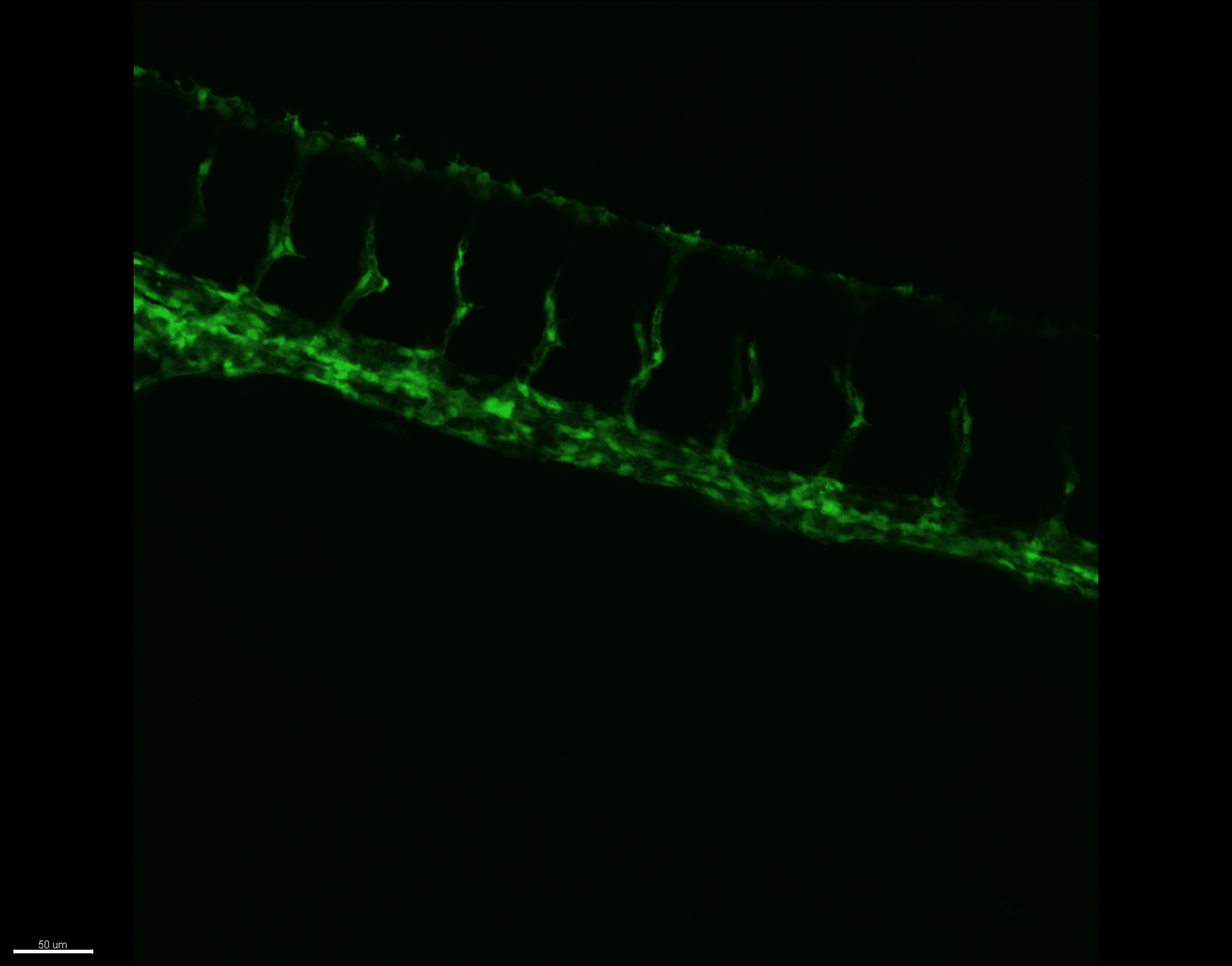

Supplement: Supplementary file 9 — Source data Fig. 4 [file 44319_2026_805_MOESM9_ESM.zip › Source Data Fig.4/Fig.4/M/1. fli1aEGFP 36hpf controlASO.tif]

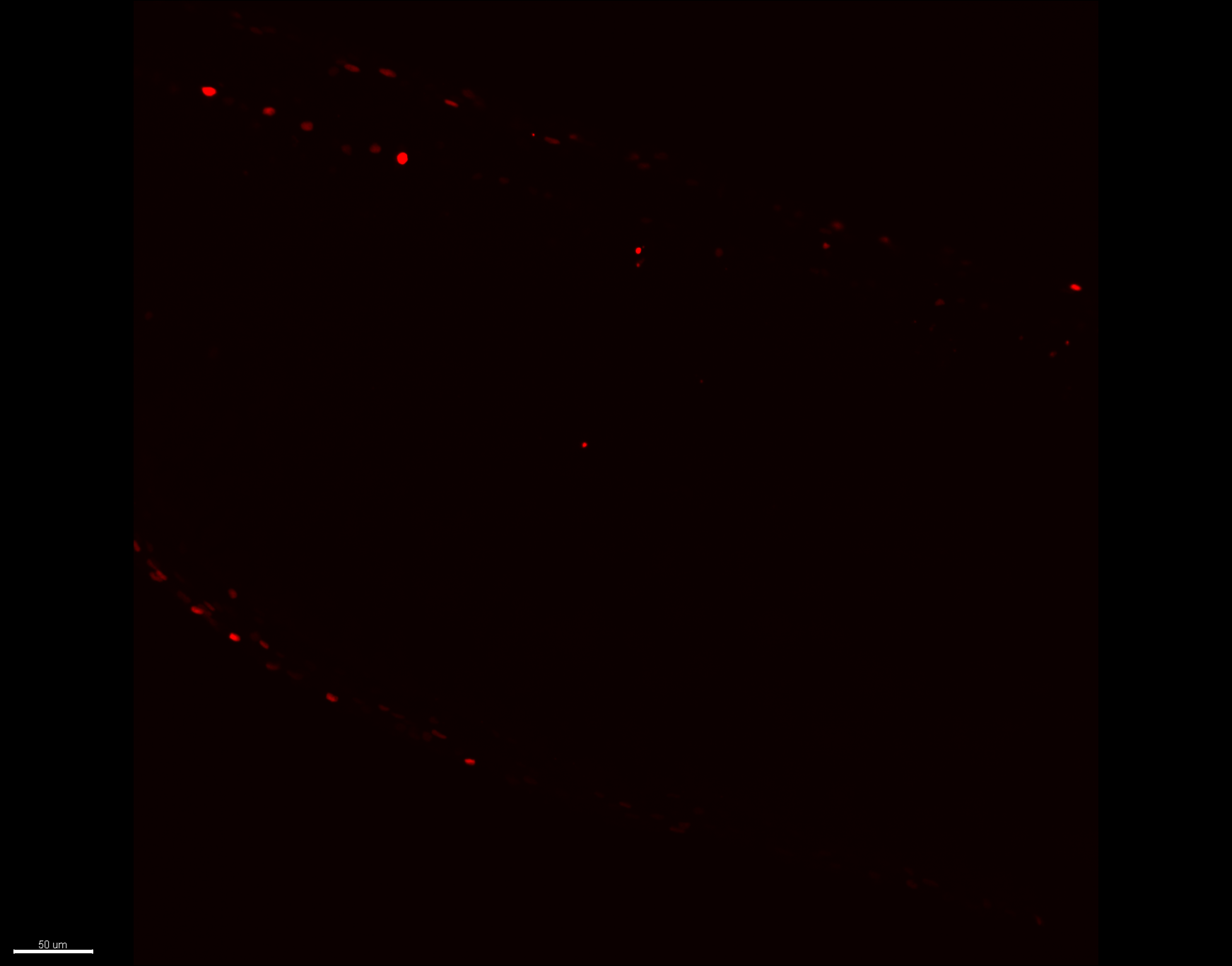

Supplement: Supplementary file 9 — Source data Fig. 4 [file 44319_2026_805_MOESM9_ESM.zip › Source Data Fig.4/Fig.4/M/2. tunel 36hpf controlASO.tif]

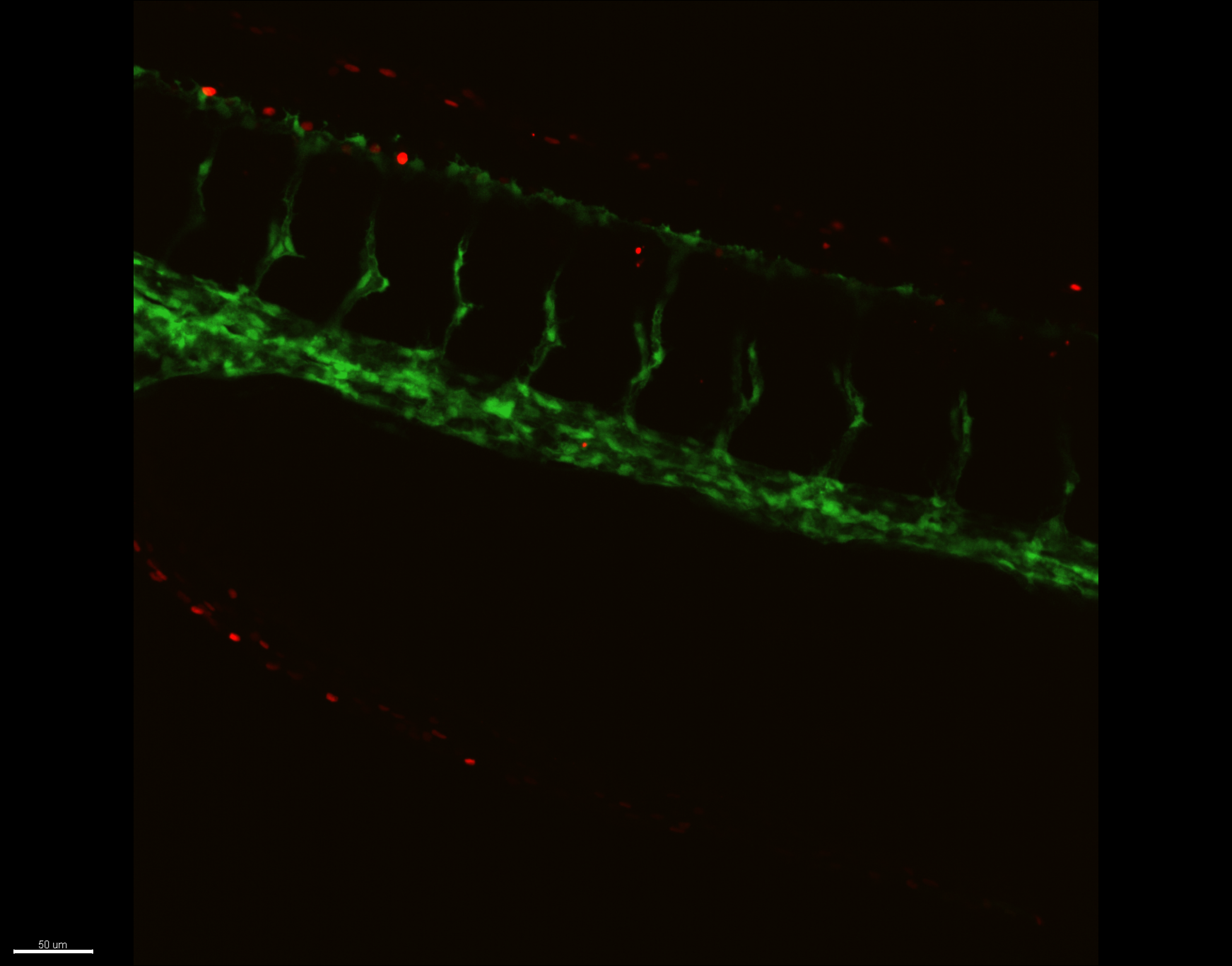

Supplement: Supplementary file 9 — Source data Fig. 4 [file 44319_2026_805_MOESM9_ESM.zip › Source Data Fig.4/Fig.4/M/3. merge 36hpf controlASO.tif]

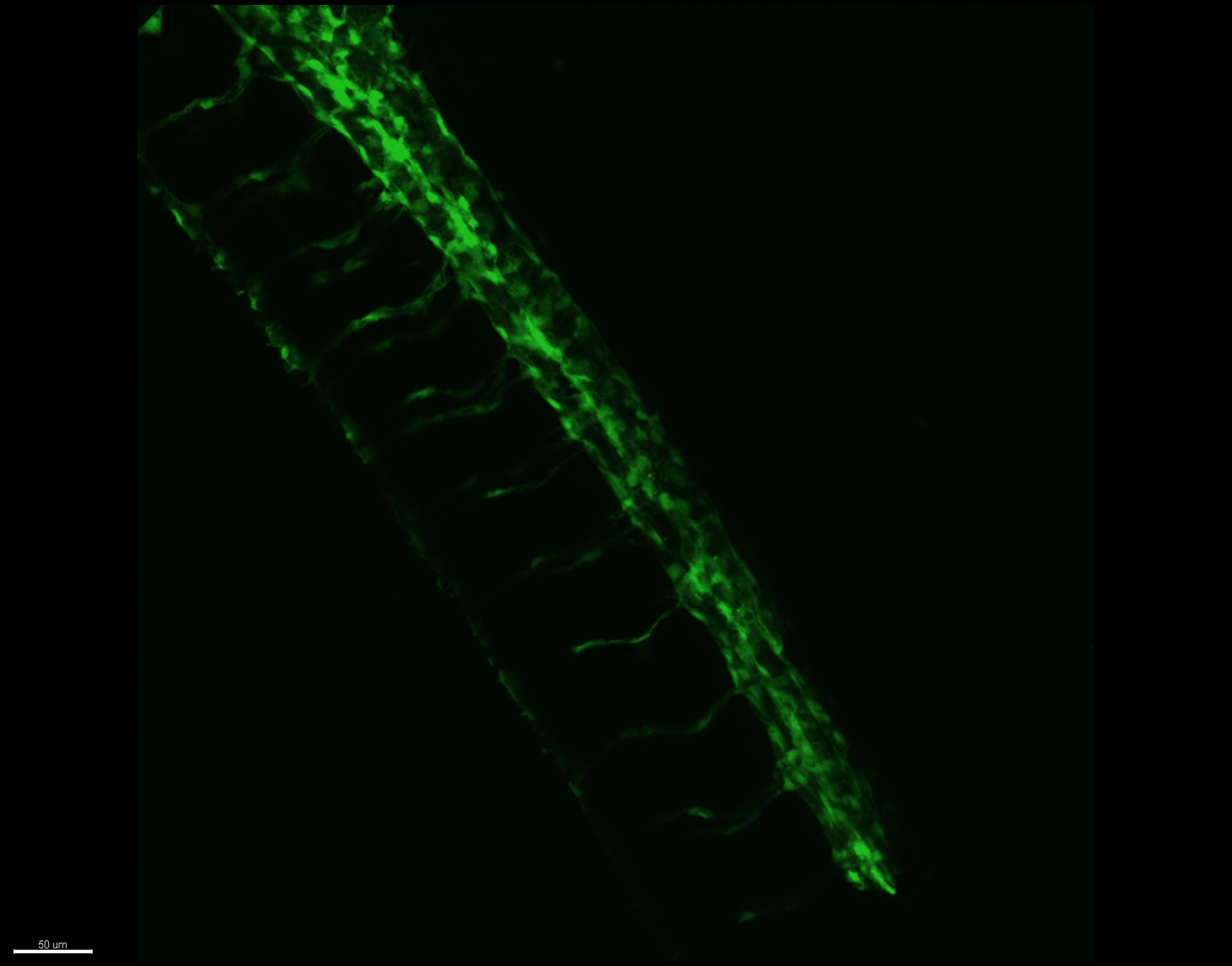

Supplement: Supplementary file 9 — Source data Fig. 4 [file 44319_2026_805_MOESM9_ESM.zip › Source Data Fig.4/Fig.4/M/4. fli1aEGFP 36hpf nrf1ASO.tif]

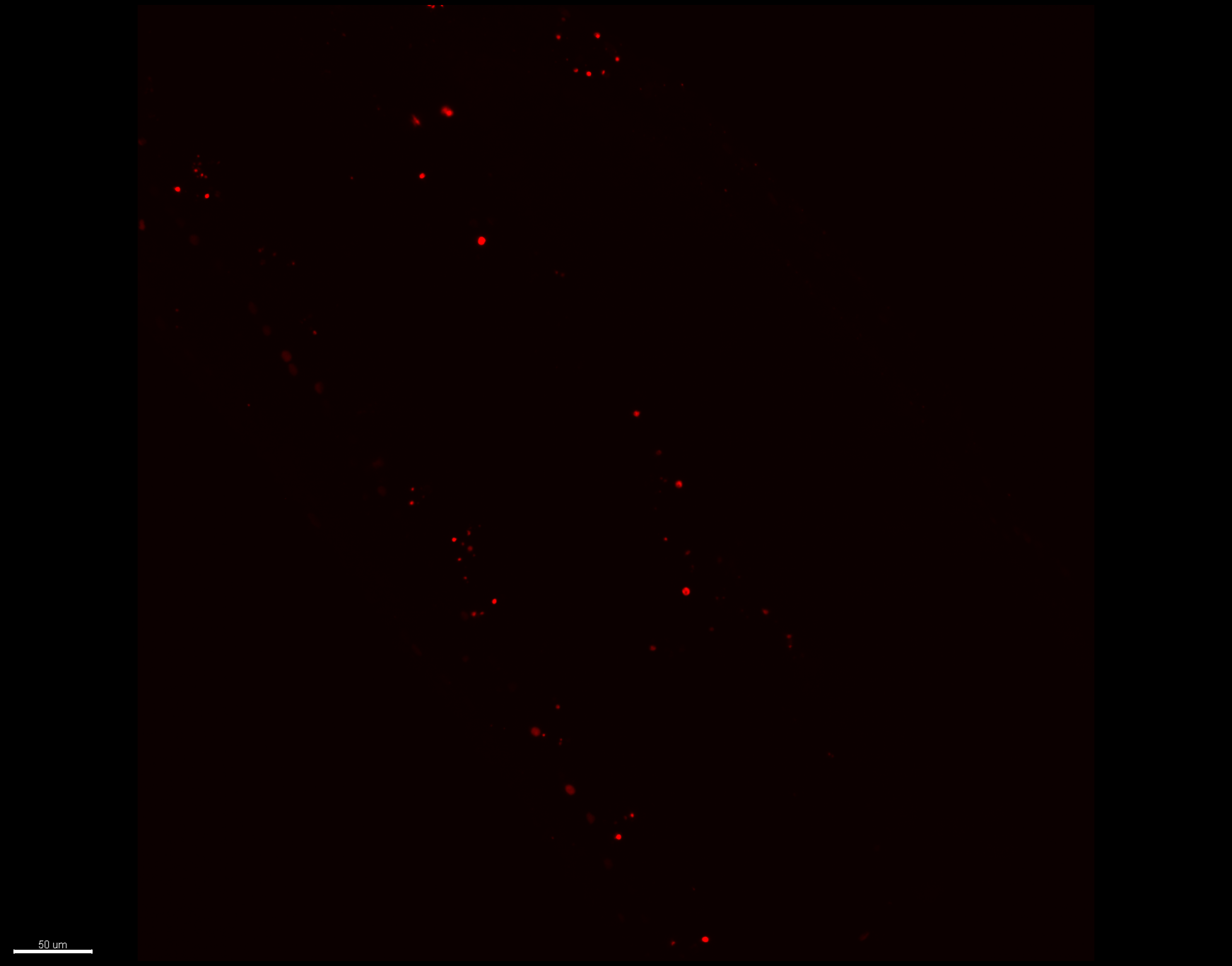

Supplement: Supplementary file 9 — Source data Fig. 4 [file 44319_2026_805_MOESM9_ESM.zip › Source Data Fig.4/Fig.4/M/5. tunel 36hpf nrf1ASO.tif]

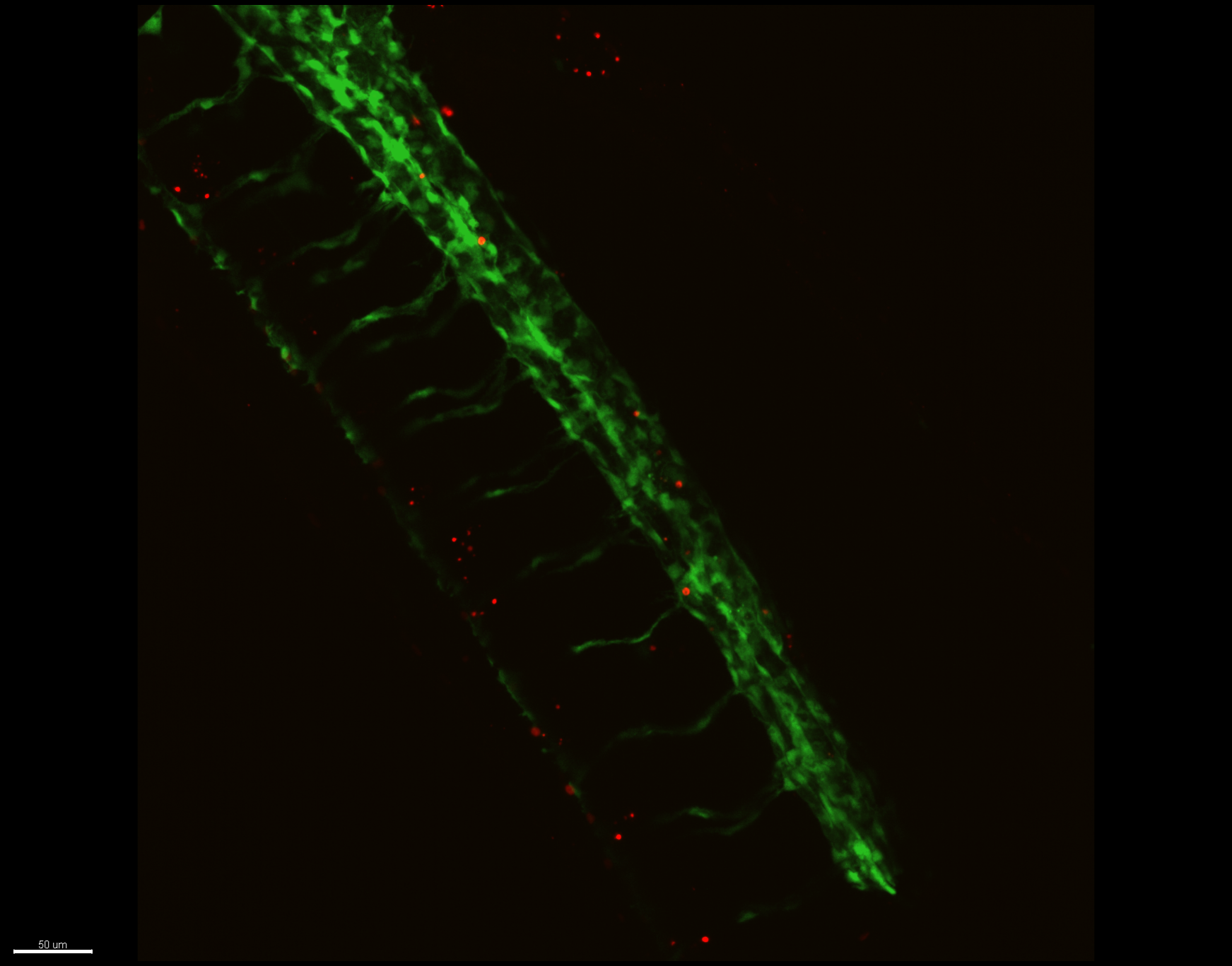

Supplement: Supplementary file 9 — Source data Fig. 4 [file 44319_2026_805_MOESM9_ESM.zip › Source Data Fig.4/Fig.4/M/6. merge 36hpf nrf1ASO.tif]

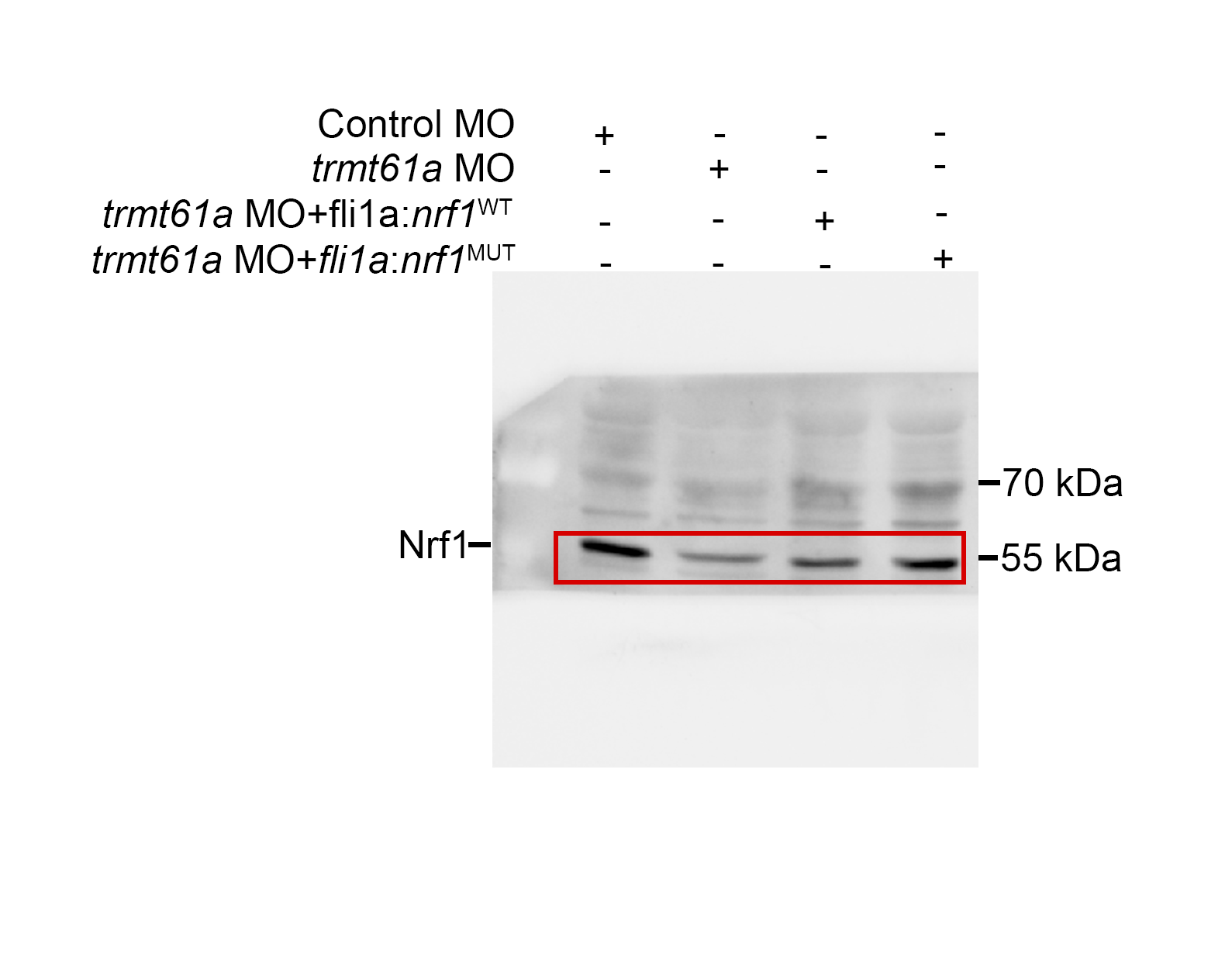

Supplement: Supplementary file 10 — Source data Fig. 5 [file 44319_2026_805_MOESM10_ESM.zip › Source Data Fig.5/Fig.5/H/5H_Nrf1 WB.tif]

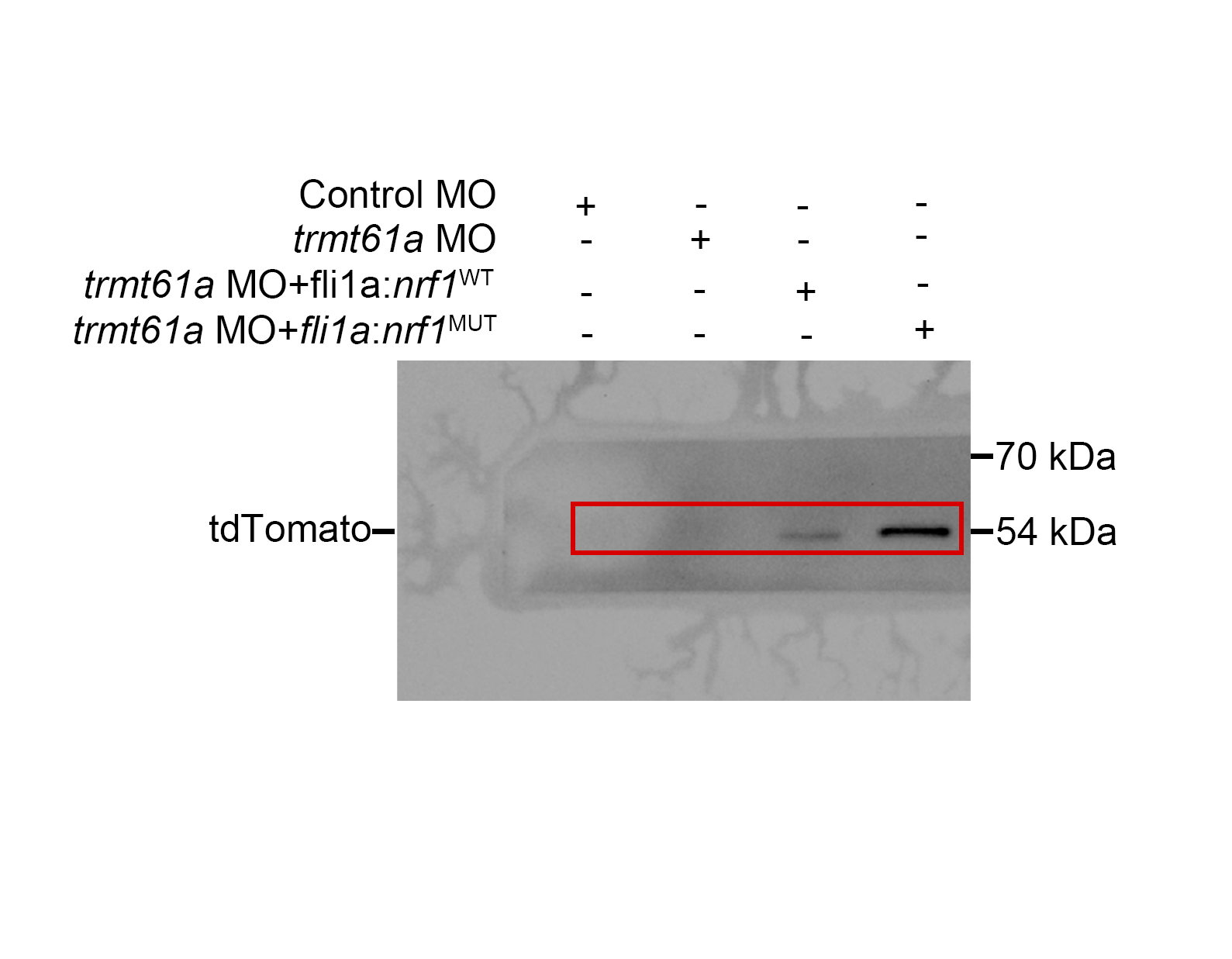

Supplement: Supplementary file 10 — Source data Fig. 5 [file 44319_2026_805_MOESM10_ESM.zip › Source Data Fig.5/Fig.5/H/5H_tdTomato WB.tif]

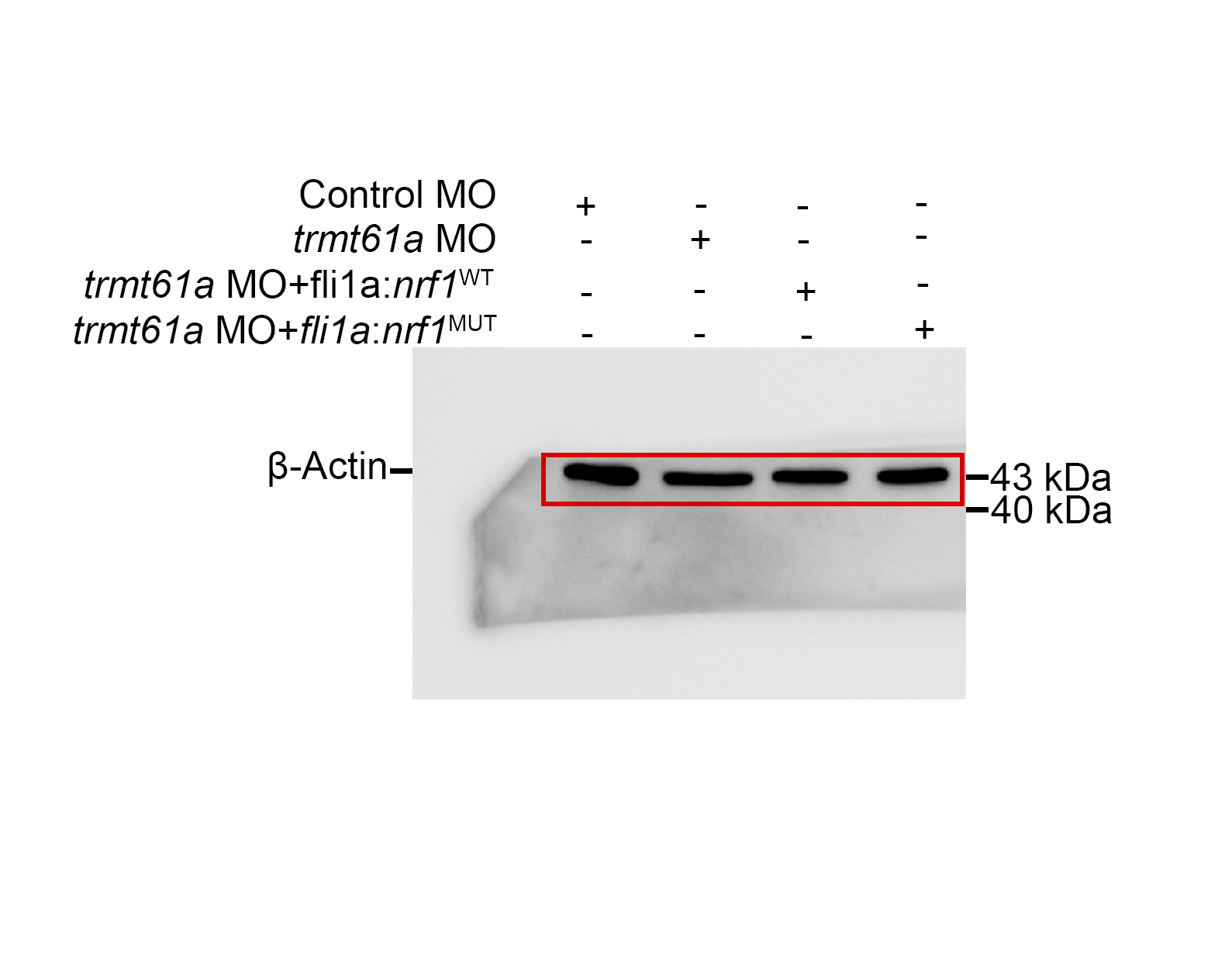

Supplement: Supplementary file 10 — Source data Fig. 5 [file 44319_2026_805_MOESM10_ESM.zip › Source Data Fig.5/Fig.5/H/5H_β-Actin WB.tif]

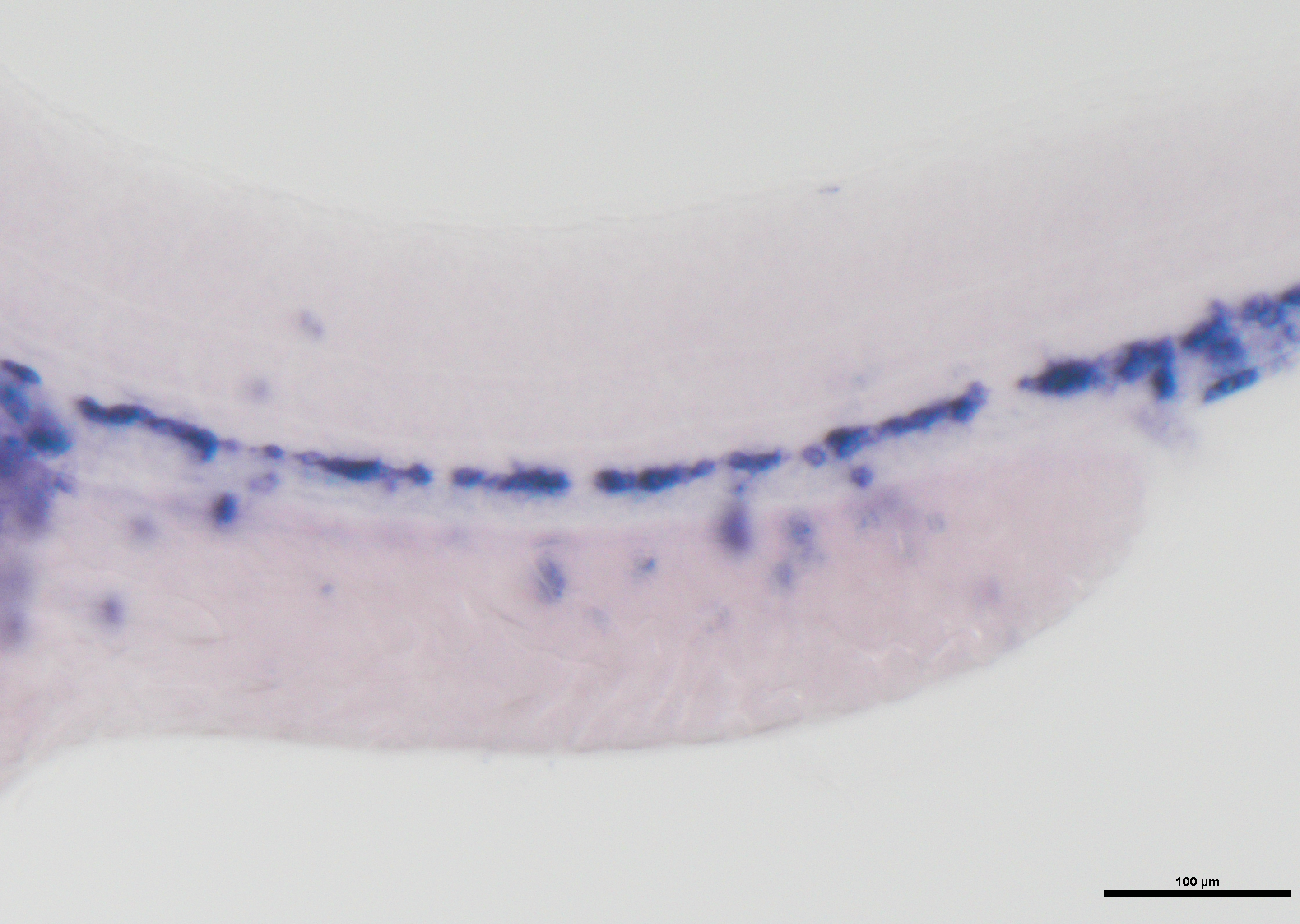

Supplement: Supplementary file 10 — Source data Fig. 5 [file 44319_2026_805_MOESM10_ESM.zip › Source Data Fig.5/Fig.5/I/1. cmyb 36hpf controlMO.tif]

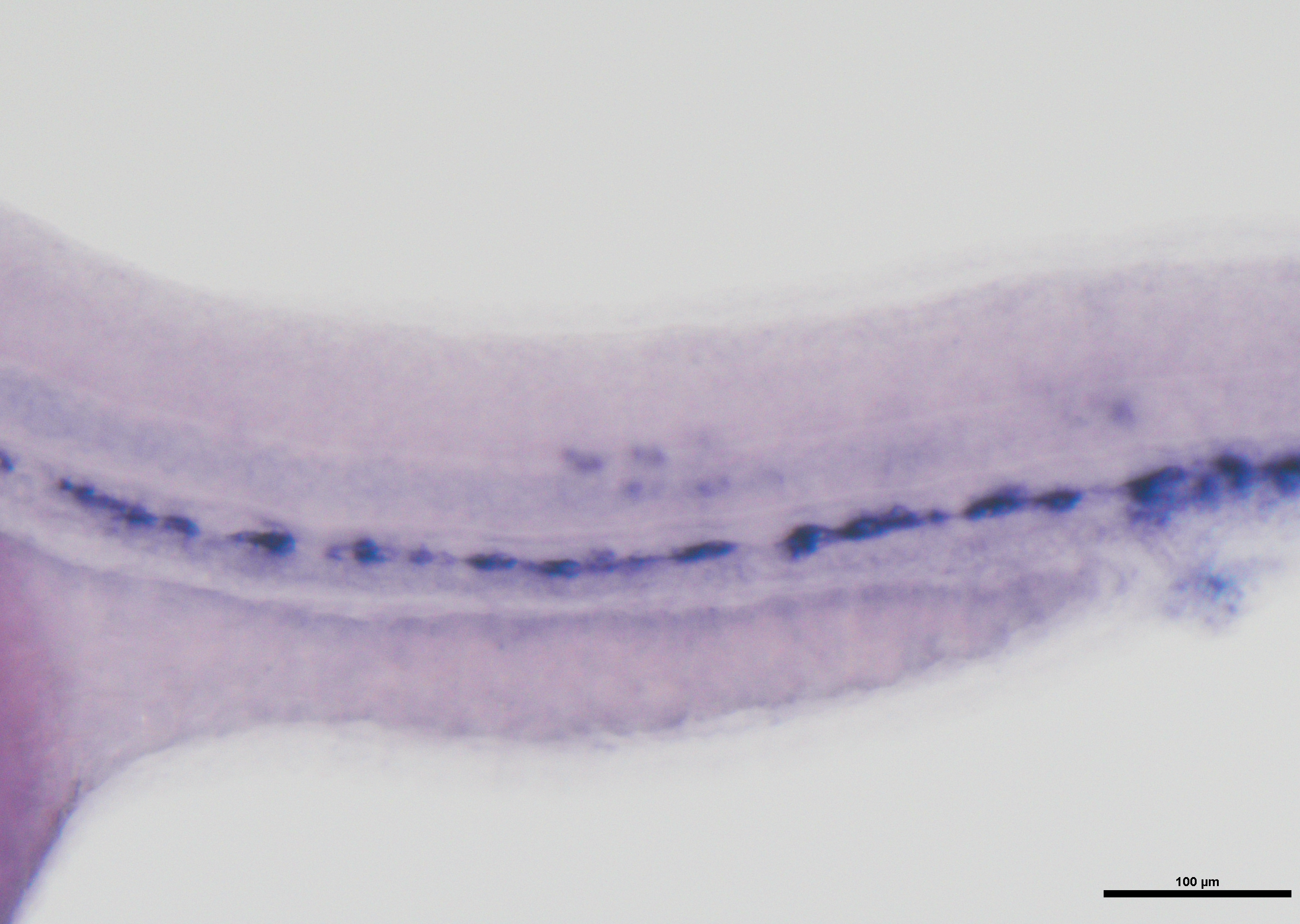

Supplement: Supplementary file 10 — Source data Fig. 5 [file 44319_2026_805_MOESM10_ESM.zip › Source Data Fig.5/Fig.5/I/2. runx1 36hpf controlMO.tif]

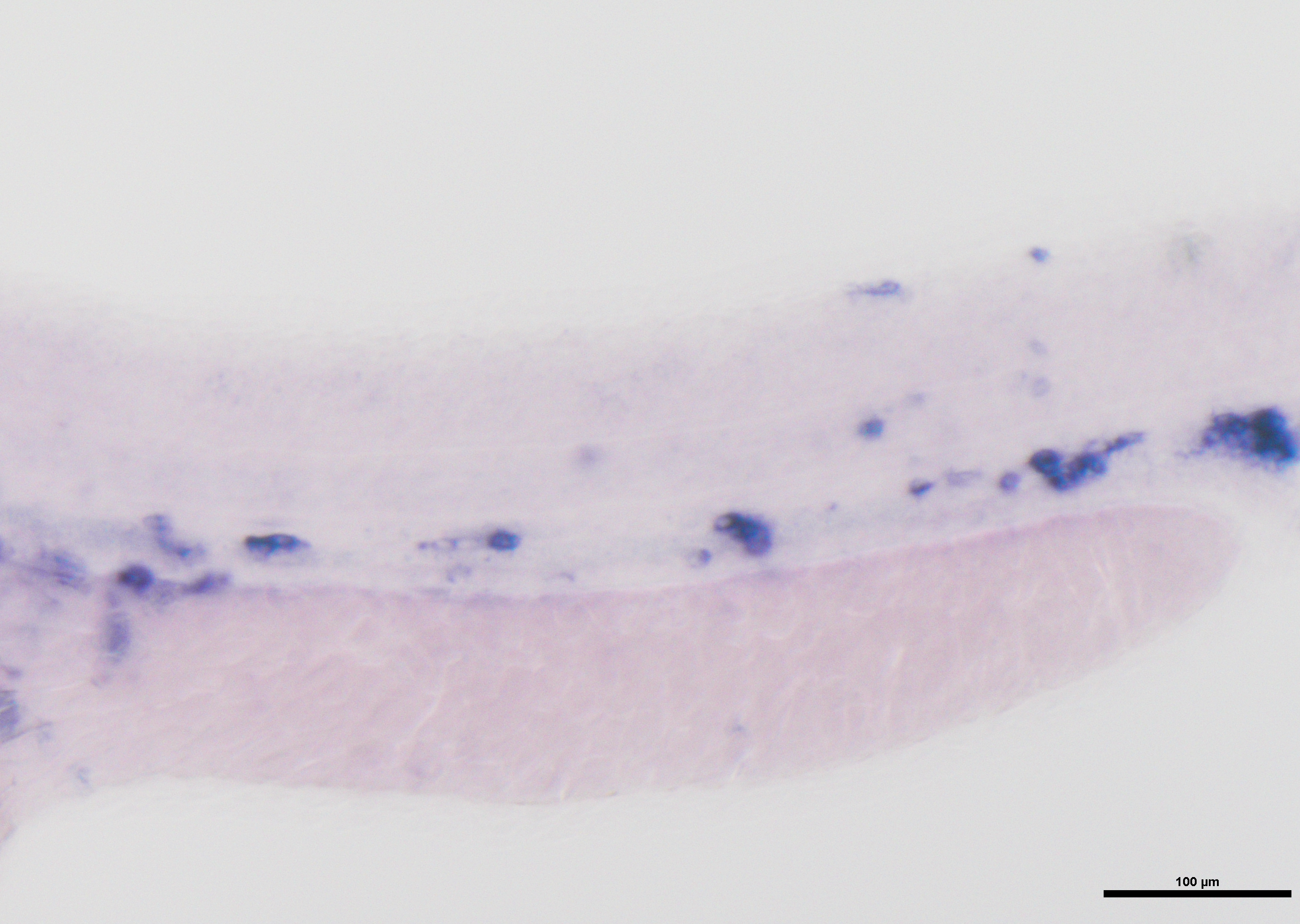

Supplement: Supplementary file 10 — Source data Fig. 5 [file 44319_2026_805_MOESM10_ESM.zip › Source Data Fig.5/Fig.5/I/3. cmyb 36hpf trmt61aMO.tif]

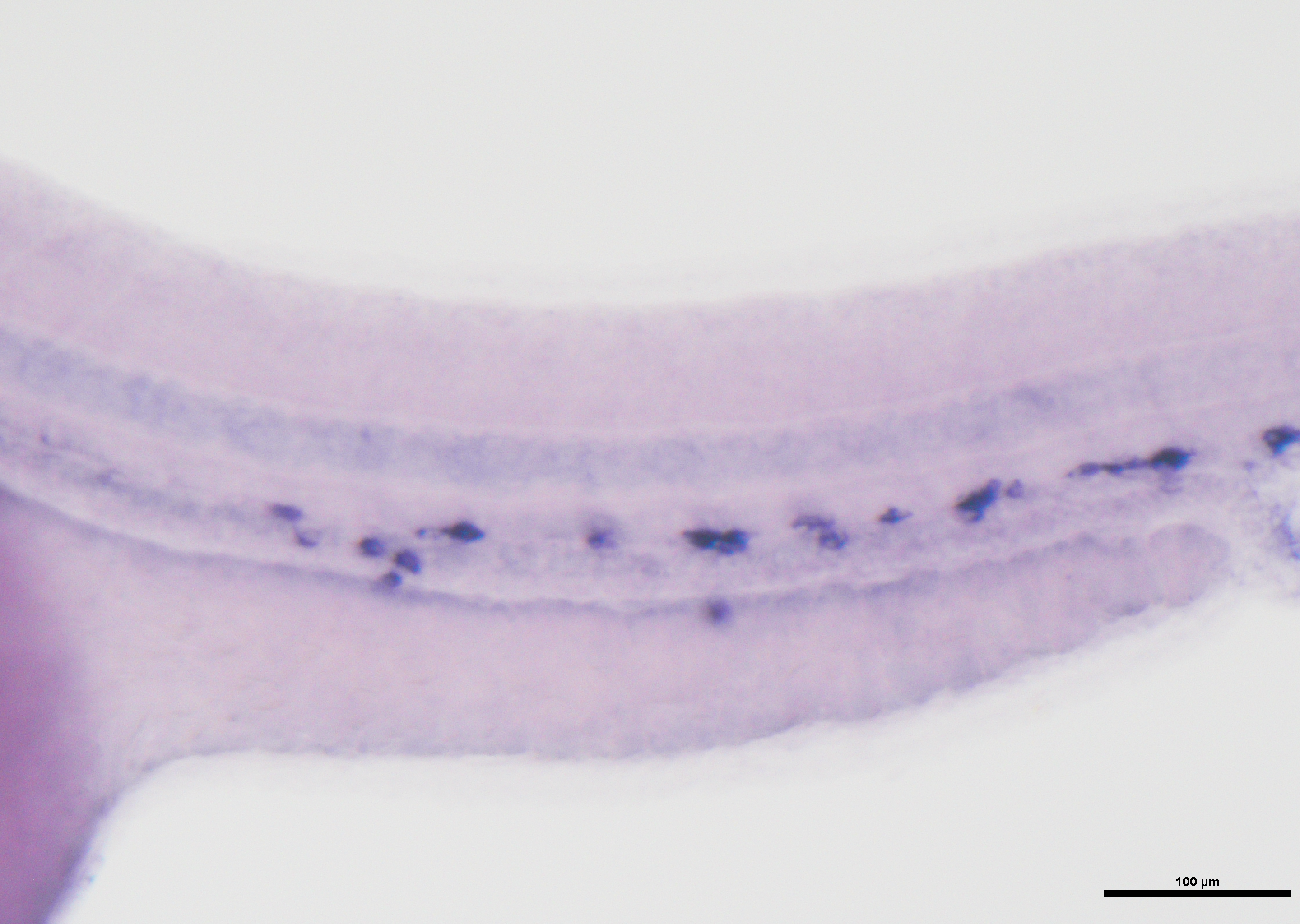

Supplement: Supplementary file 10 — Source data Fig. 5 [file 44319_2026_805_MOESM10_ESM.zip › Source Data Fig.5/Fig.5/I/4. runx1 36hpf trmt61aMO.tif]

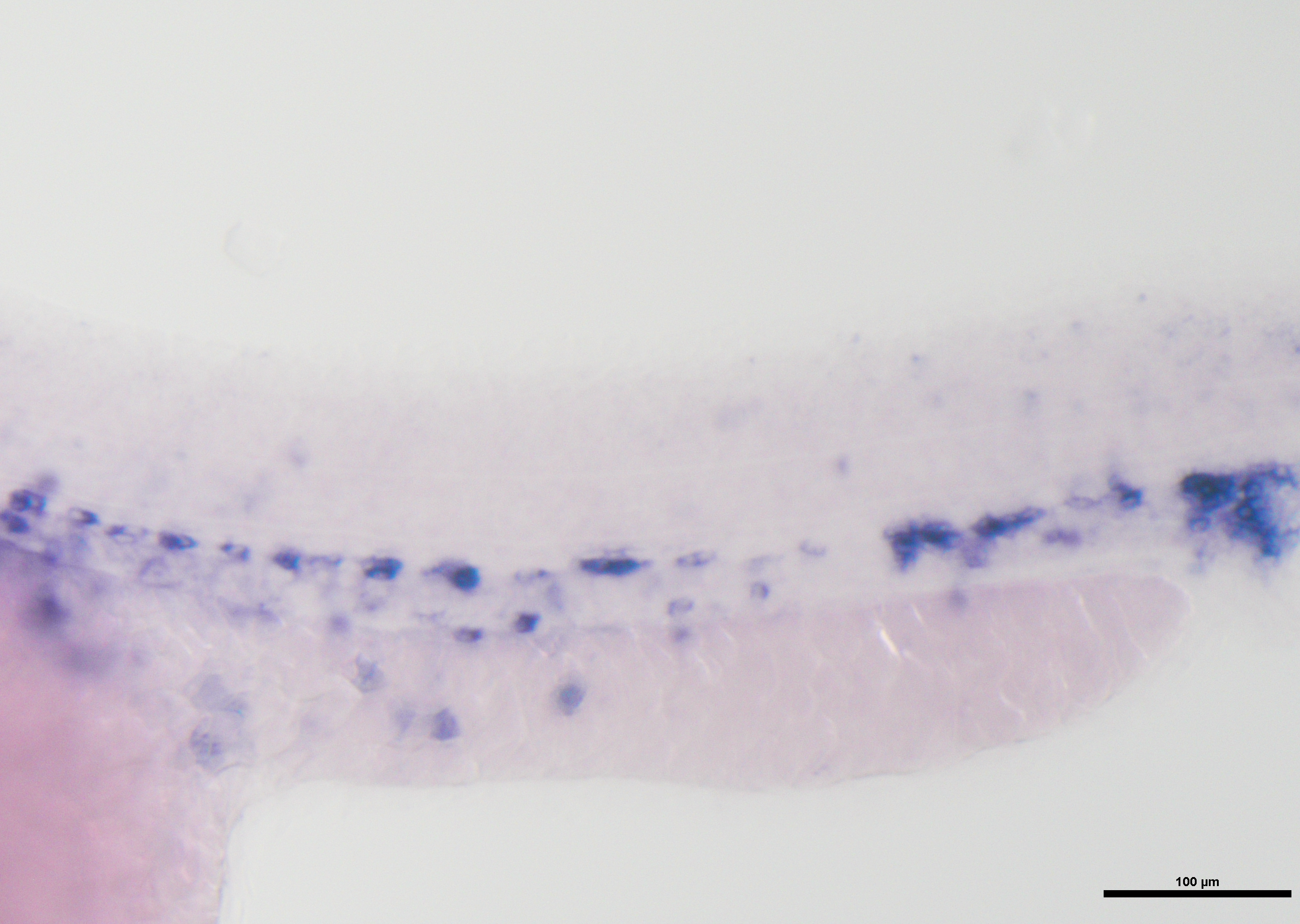

Supplement: Supplementary file 10 — Source data Fig. 5 [file 44319_2026_805_MOESM10_ESM.zip › Source Data Fig.5/Fig.5/I/5. cmyb 36hpf trmt61aMO+fli1anrf1WT.tif]

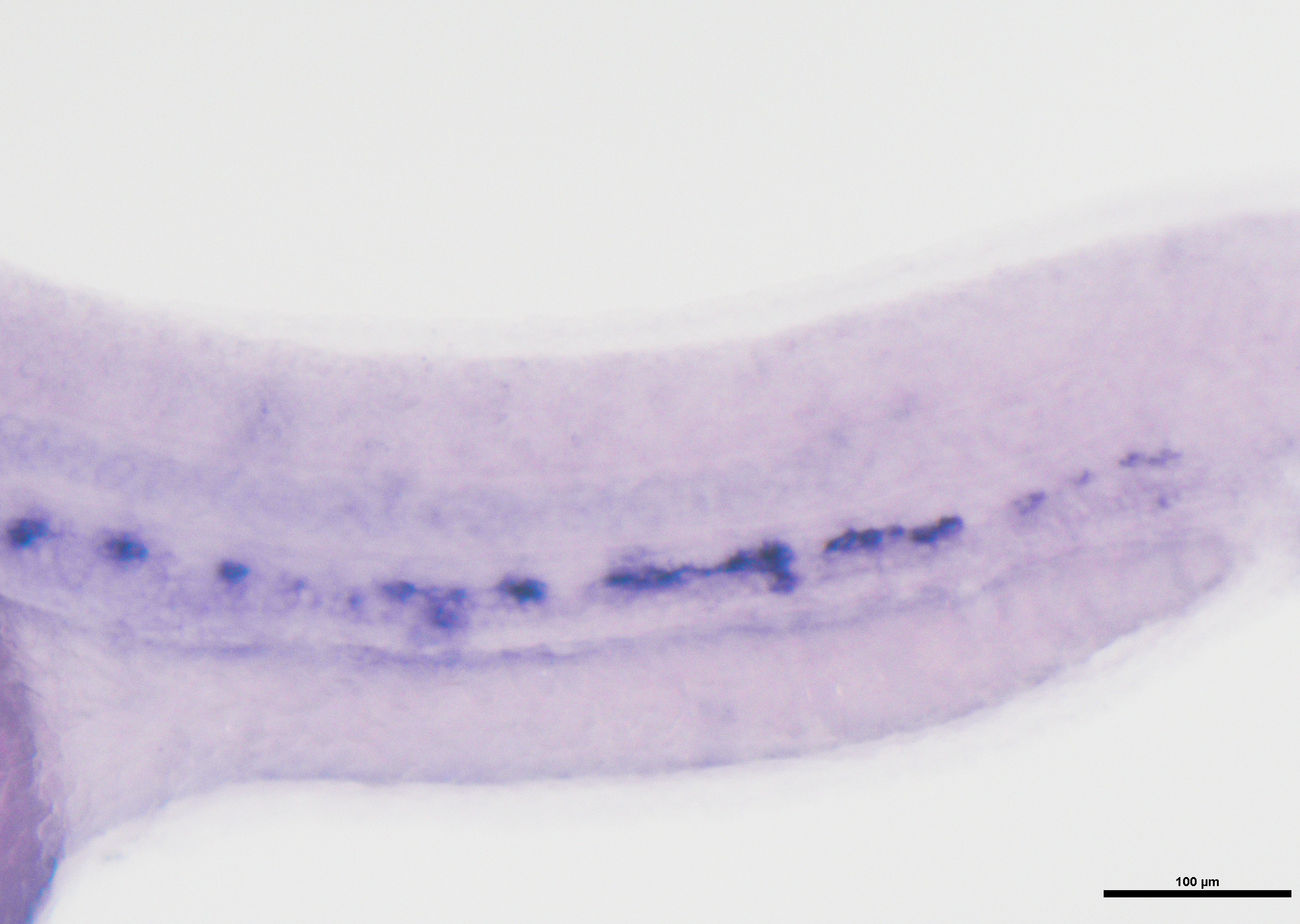

Supplement: Supplementary file 10 — Source data Fig. 5 [file 44319_2026_805_MOESM10_ESM.zip › Source Data Fig.5/Fig.5/I/6. runx1 36hpf trmt61aMO+fli1anrf1WT.tif]

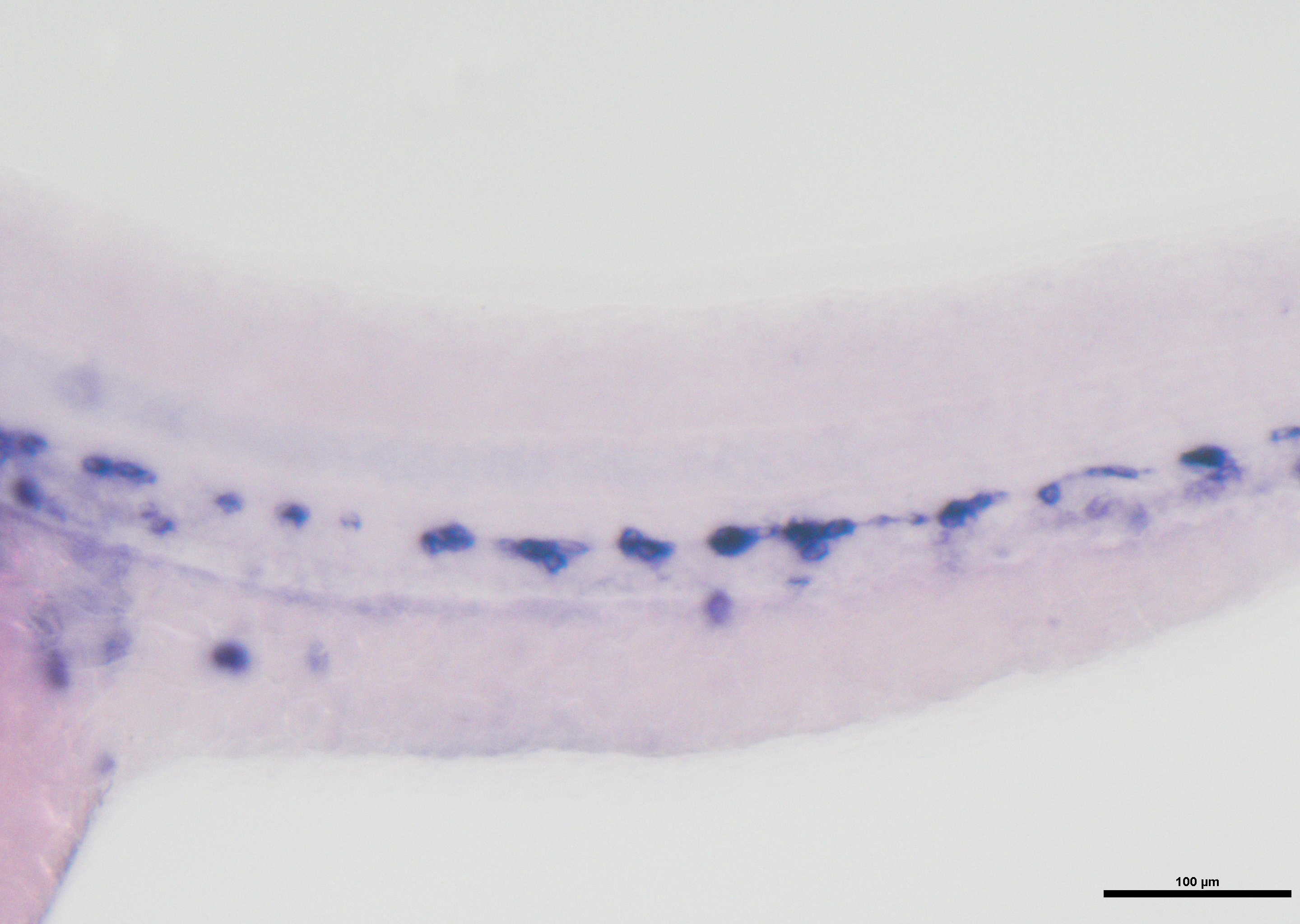

Supplement: Supplementary file 10 — Source data Fig. 5 [file 44319_2026_805_MOESM10_ESM.zip › Source Data Fig.5/Fig.5/I/7. cmyb 36hpf trmt61aMO+fli1anrf1MUT.tif]

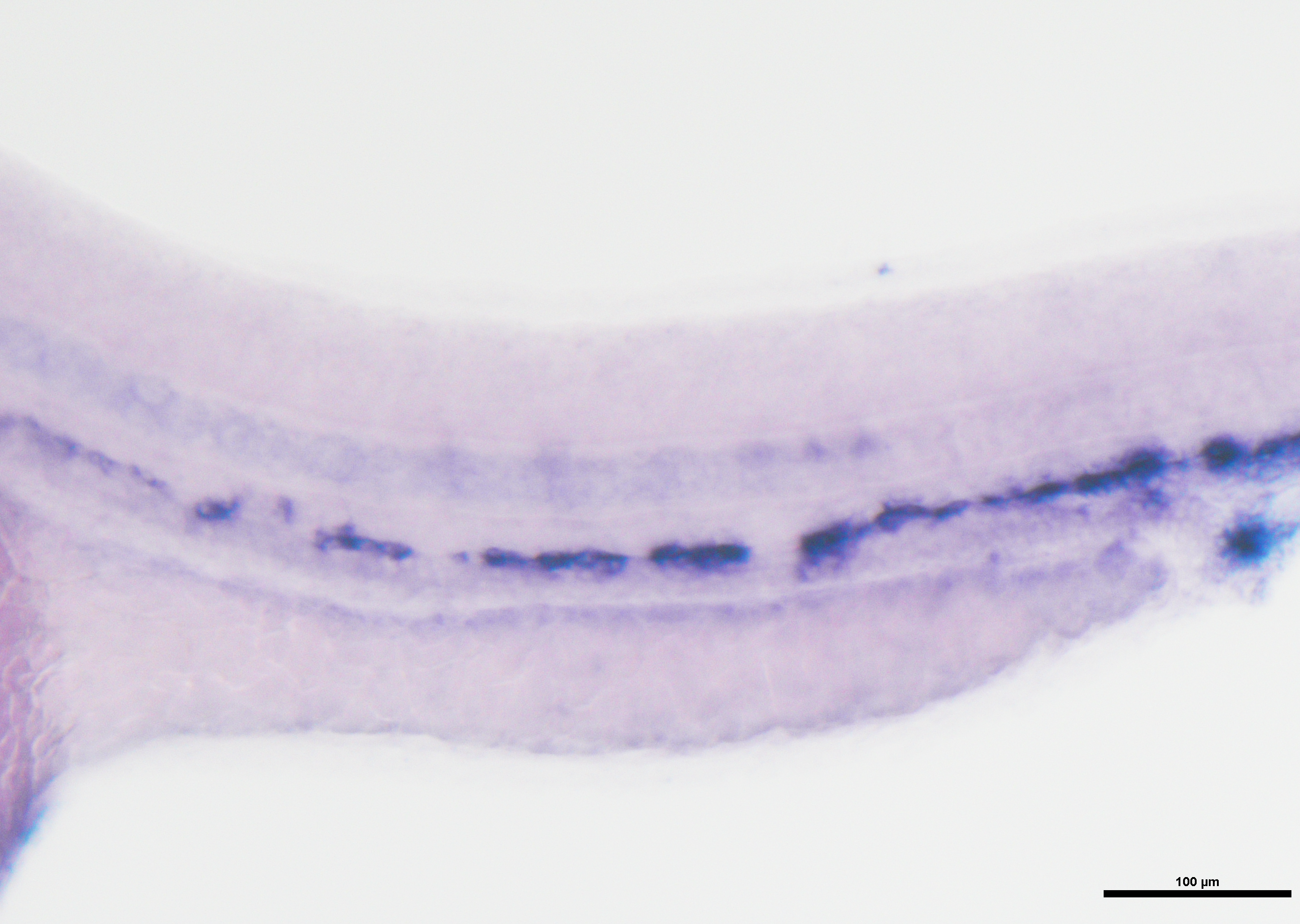

Supplement: Supplementary file 10 — Source data Fig. 5 [file 44319_2026_805_MOESM10_ESM.zip › Source Data Fig.5/Fig.5/I/8. runx1 36hpf trmt61aMO+fli1anrf1MUT.tif]

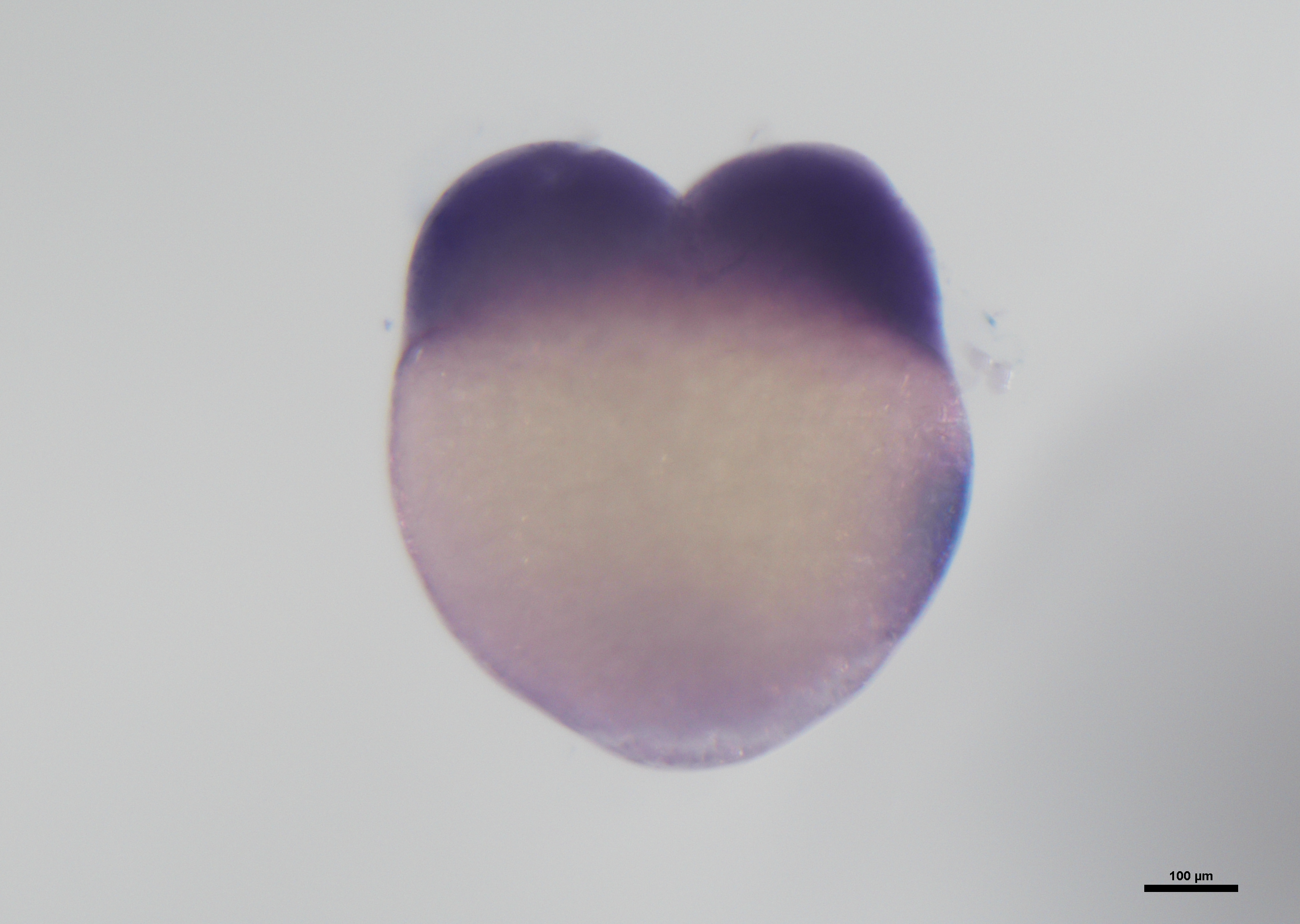

Supplement: Supplementary file 11 — Appendix Figure1-2 Source Data [file 44319_2026_805_MOESM11_ESM.zip › Appendix Source Data 1/Appendix Fig.1/B/1. trmt61a 2-cell.tif]

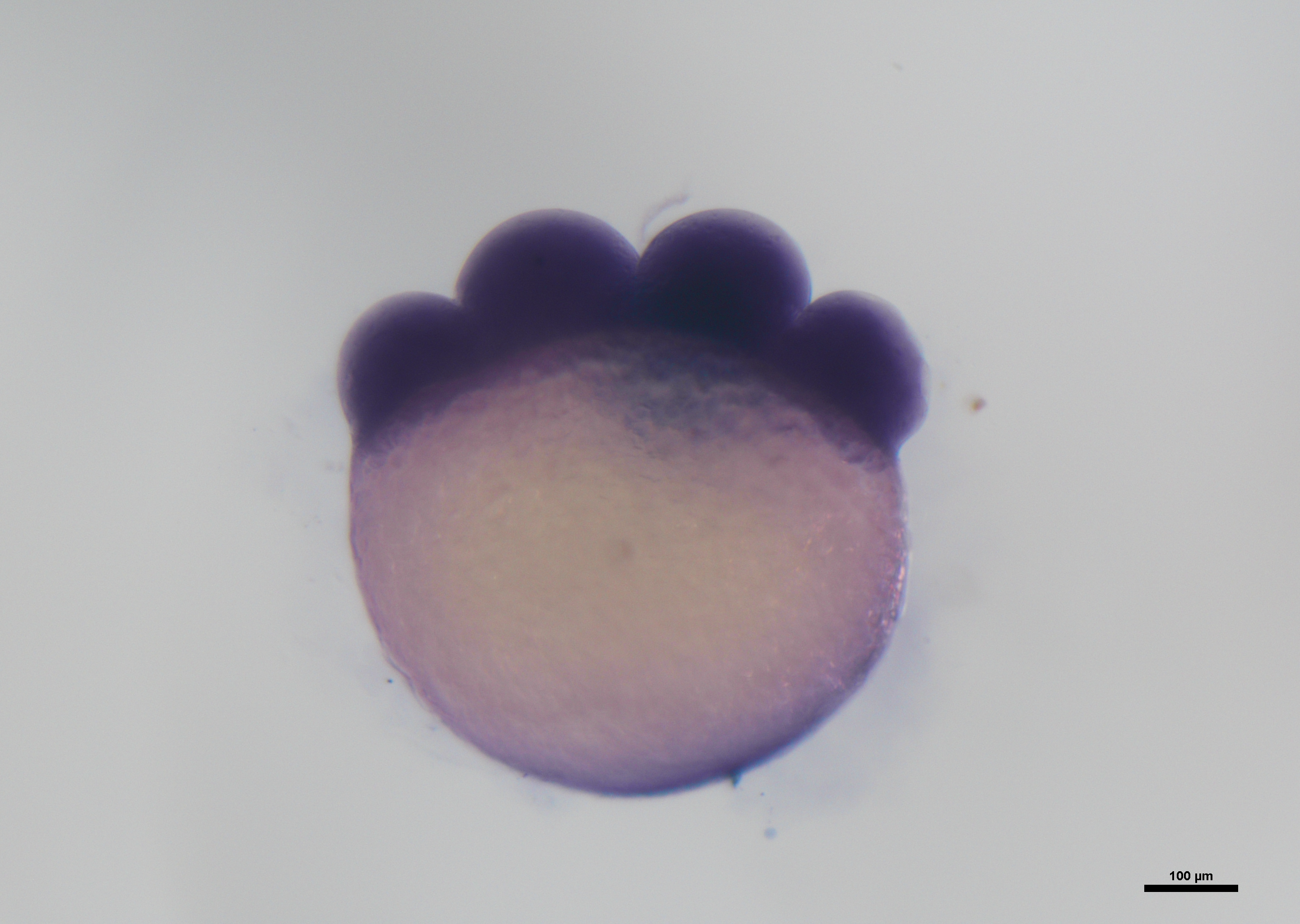

Supplement: Supplementary file 11 — Appendix Figure1-2 Source Data [file 44319_2026_805_MOESM11_ESM.zip › Appendix Source Data 1/Appendix Fig.1/B/2. trmt61a 8-cell.tif]

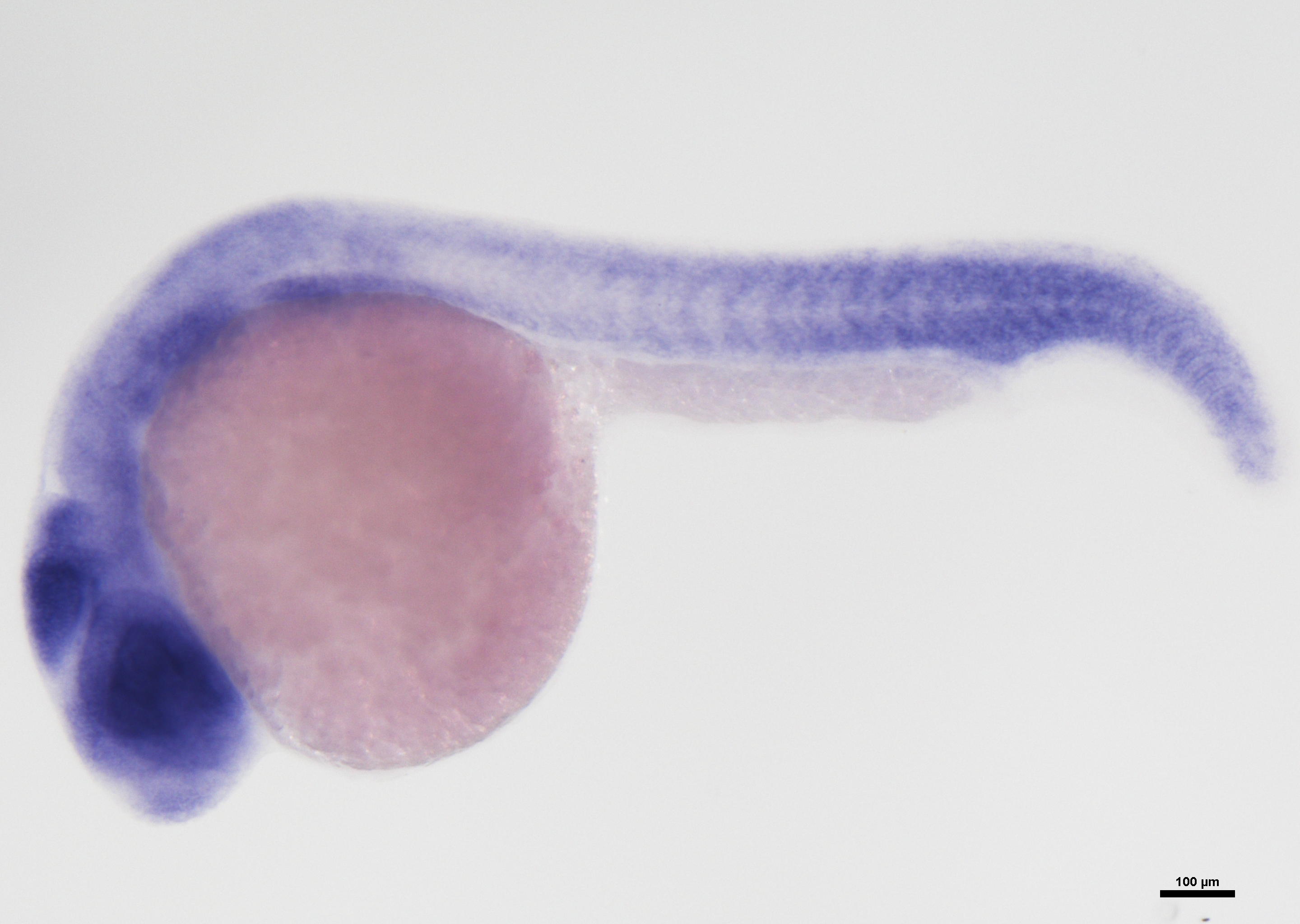

Supplement: Supplementary file 11 — Appendix Figure1-2 Source Data [file 44319_2026_805_MOESM11_ESM.zip › Appendix Source Data 1/Appendix Fig.1/B/3. trmt61a 24hpf.tif]

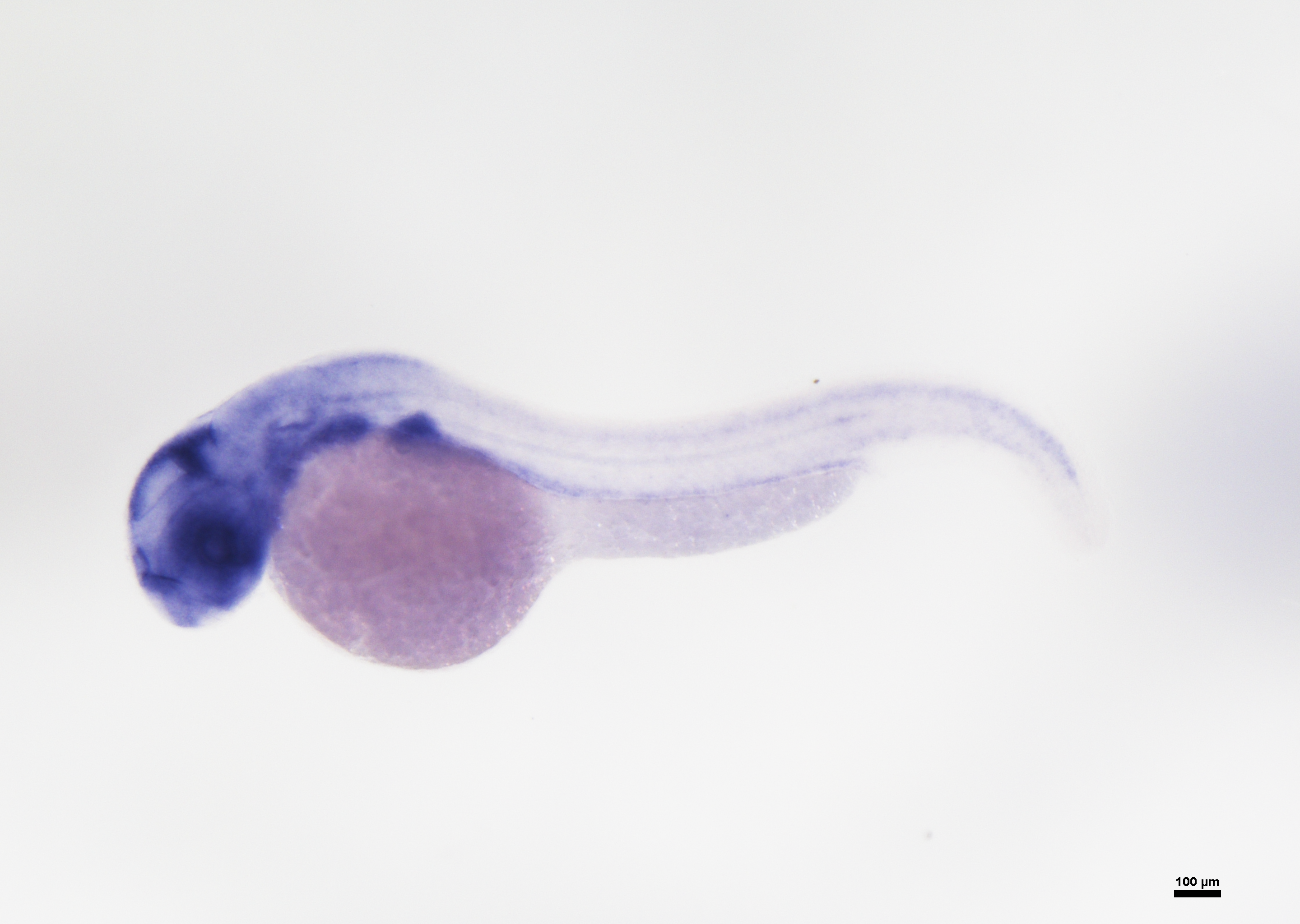

Supplement: Supplementary file 11 — Appendix Figure1-2 Source Data [file 44319_2026_805_MOESM11_ESM.zip › Appendix Source Data 1/Appendix Fig.1/B/4. trmt61a 36hpf.tif]

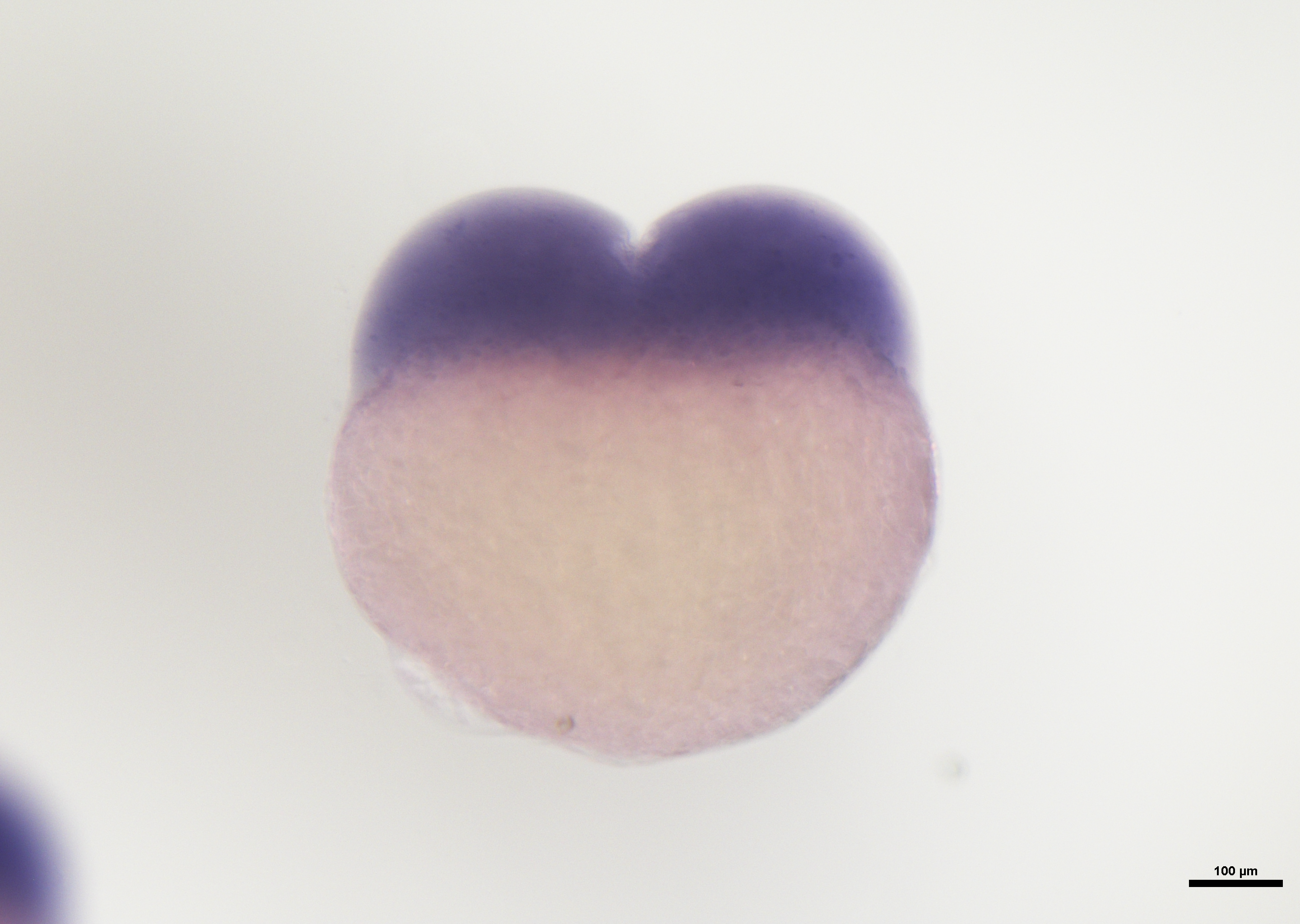

Supplement: Supplementary file 11 — Appendix Figure1-2 Source Data [file 44319_2026_805_MOESM11_ESM.zip › Appendix Source Data 1/Appendix Fig.1/B/5. trmt6 2-cell.tif]

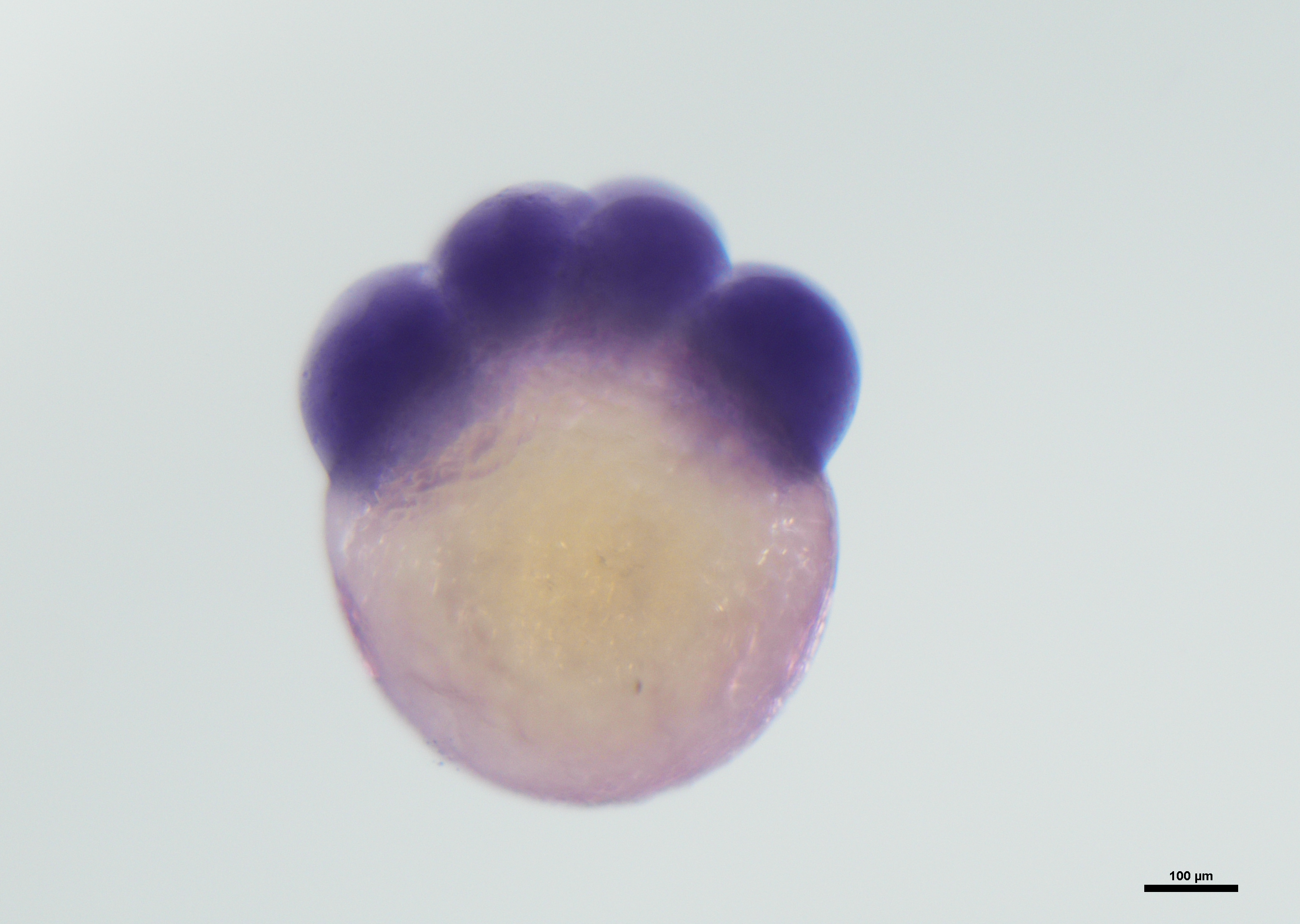

Supplement: Supplementary file 11 — Appendix Figure1-2 Source Data [file 44319_2026_805_MOESM11_ESM.zip › Appendix Source Data 1/Appendix Fig.1/B/6. trmt6 8-cell.tif]

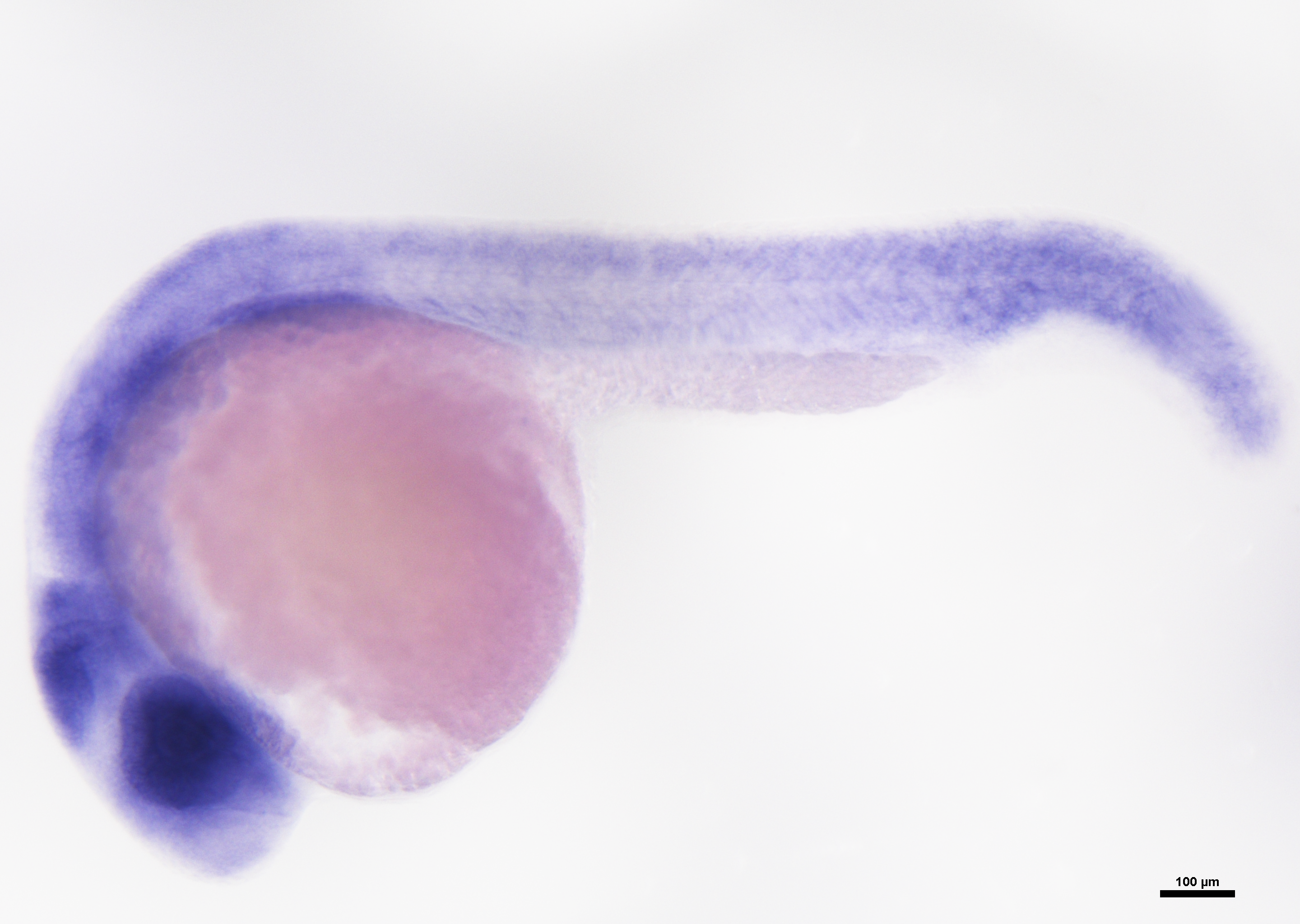

Supplement: Supplementary file 11 — Appendix Figure1-2 Source Data [file 44319_2026_805_MOESM11_ESM.zip › Appendix Source Data 1/Appendix Fig.1/B/7. trmt6 24hpf.tif]

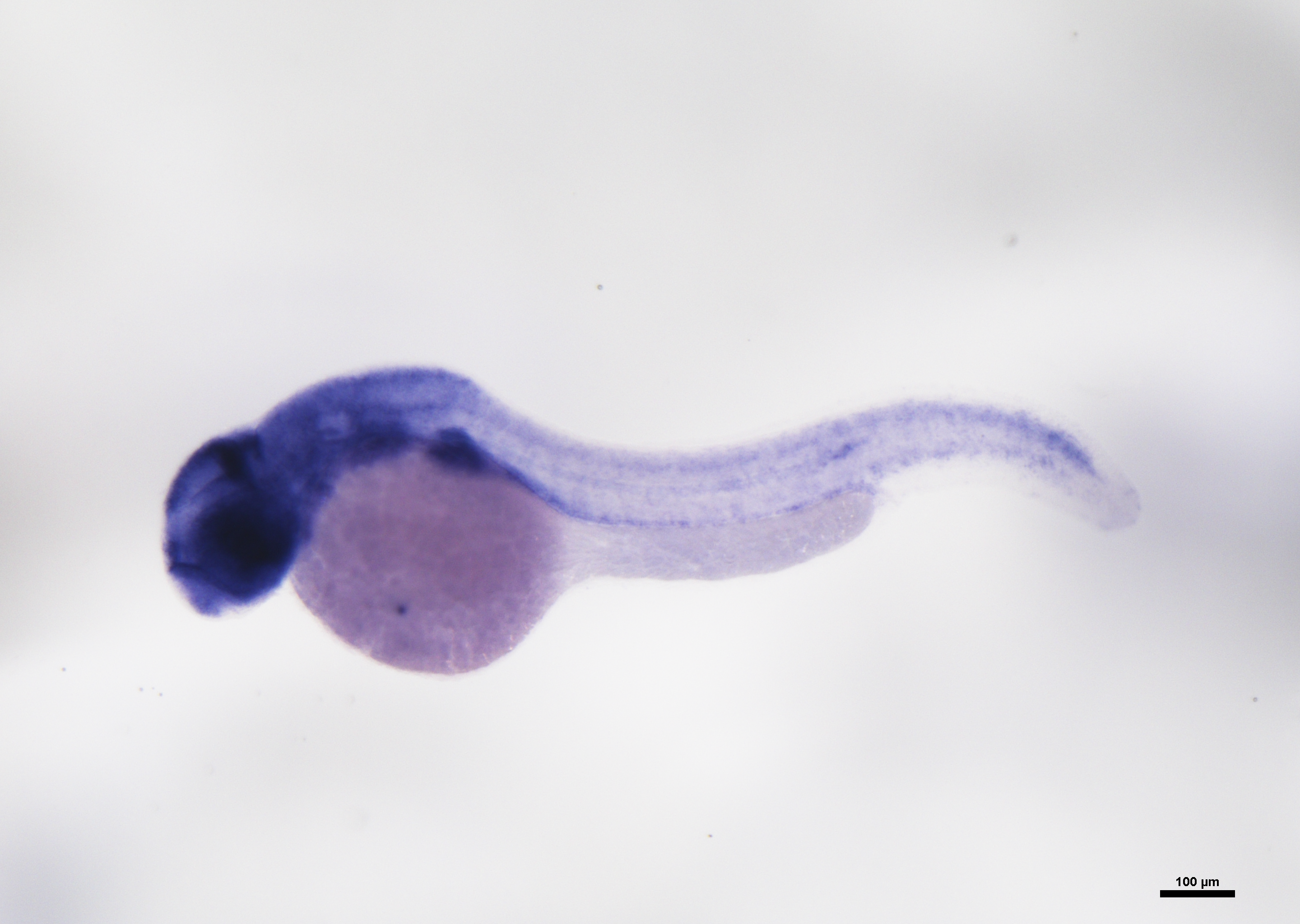

Supplement: Supplementary file 11 — Appendix Figure1-2 Source Data [file 44319_2026_805_MOESM11_ESM.zip › Appendix Source Data 1/Appendix Fig.1/B/8. trmt6 36hpf.tif]

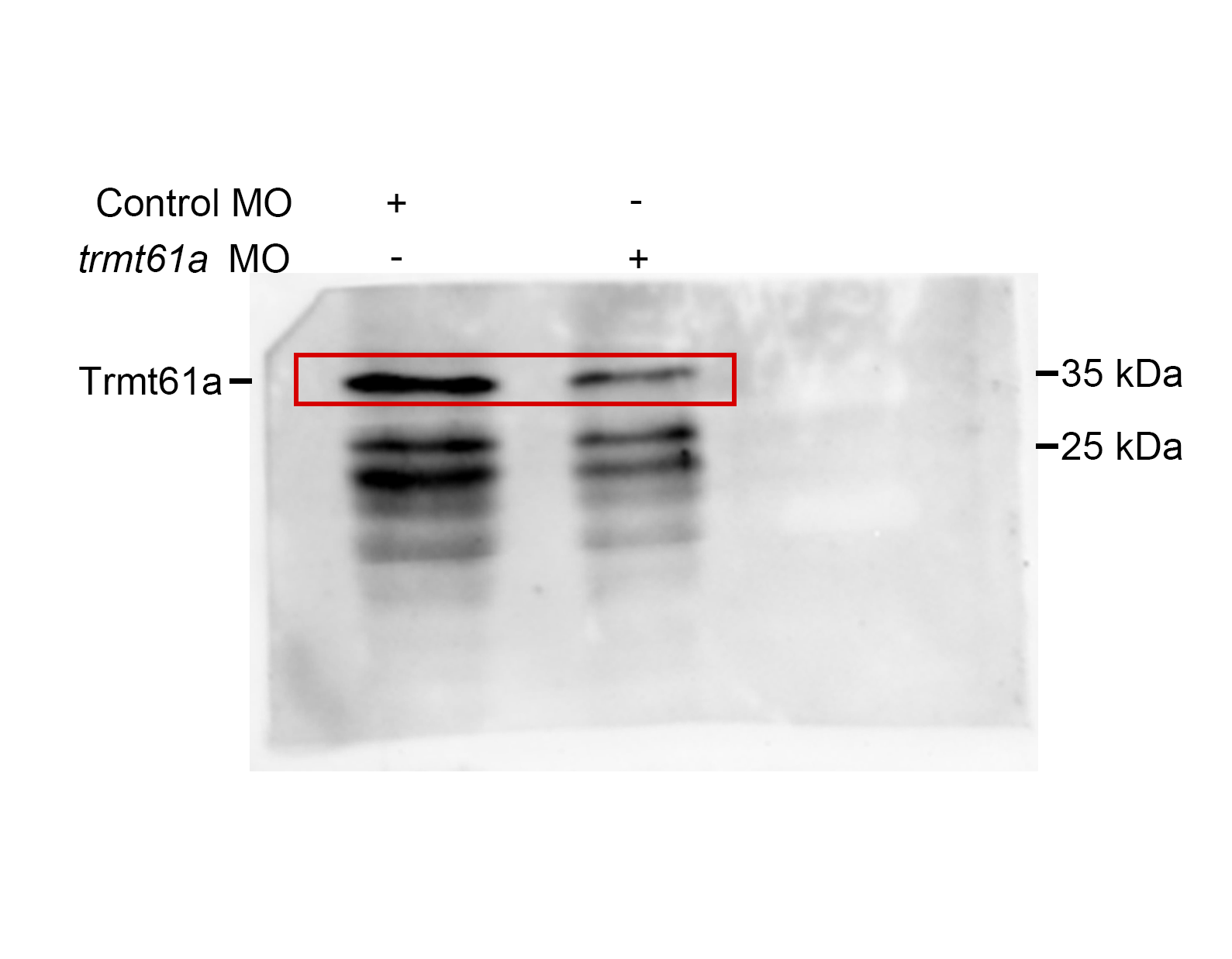

Supplement: Supplementary file 11 — Appendix Figure1-2 Source Data [file 44319_2026_805_MOESM11_ESM.zip › Appendix Source Data 1/Appendix Fig.2/B/2B_Trmt61a WB.tif]

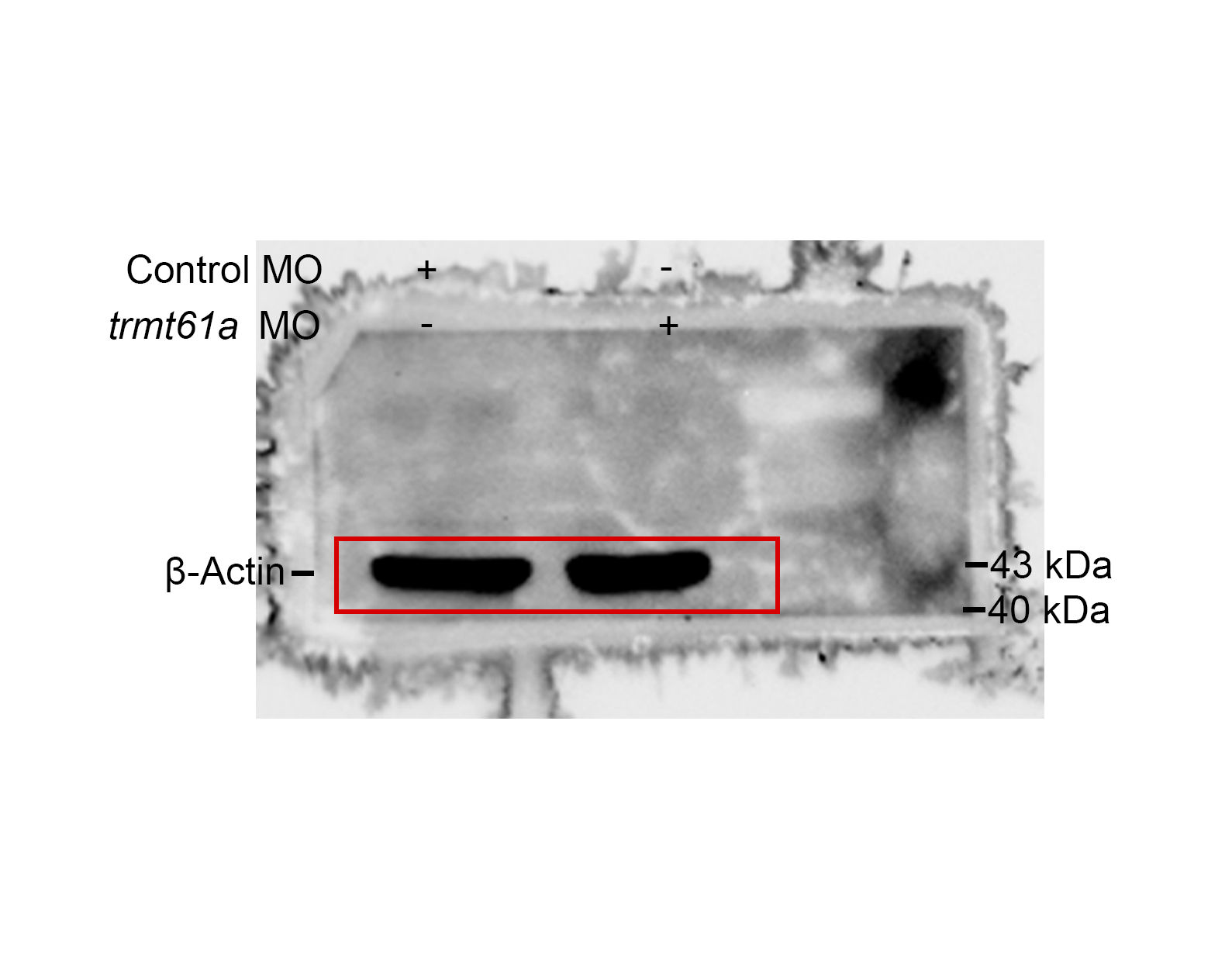

Supplement: Supplementary file 11 — Appendix Figure1-2 Source Data [file 44319_2026_805_MOESM11_ESM.zip › Appendix Source Data 1/Appendix Fig.2/B/2B_β-Actin WB.tif]

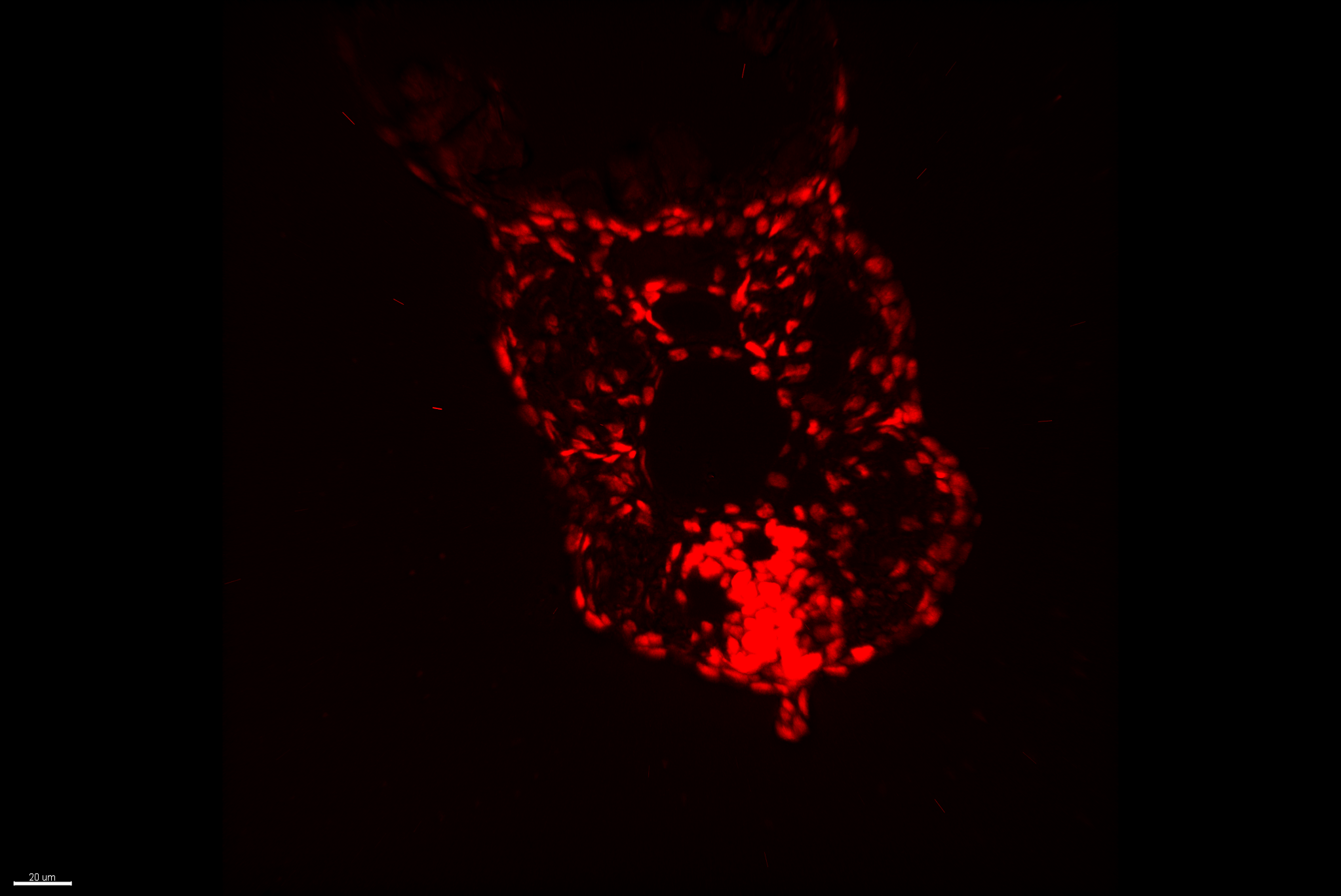

Supplement: Supplementary file 11 — Appendix Figure1-2 Source Data [file 44319_2026_805_MOESM11_ESM.zip › Appendix Source Data 1/Appendix Fig.2/C/1. anti-trmt61a 36hpf controlMO.tif]

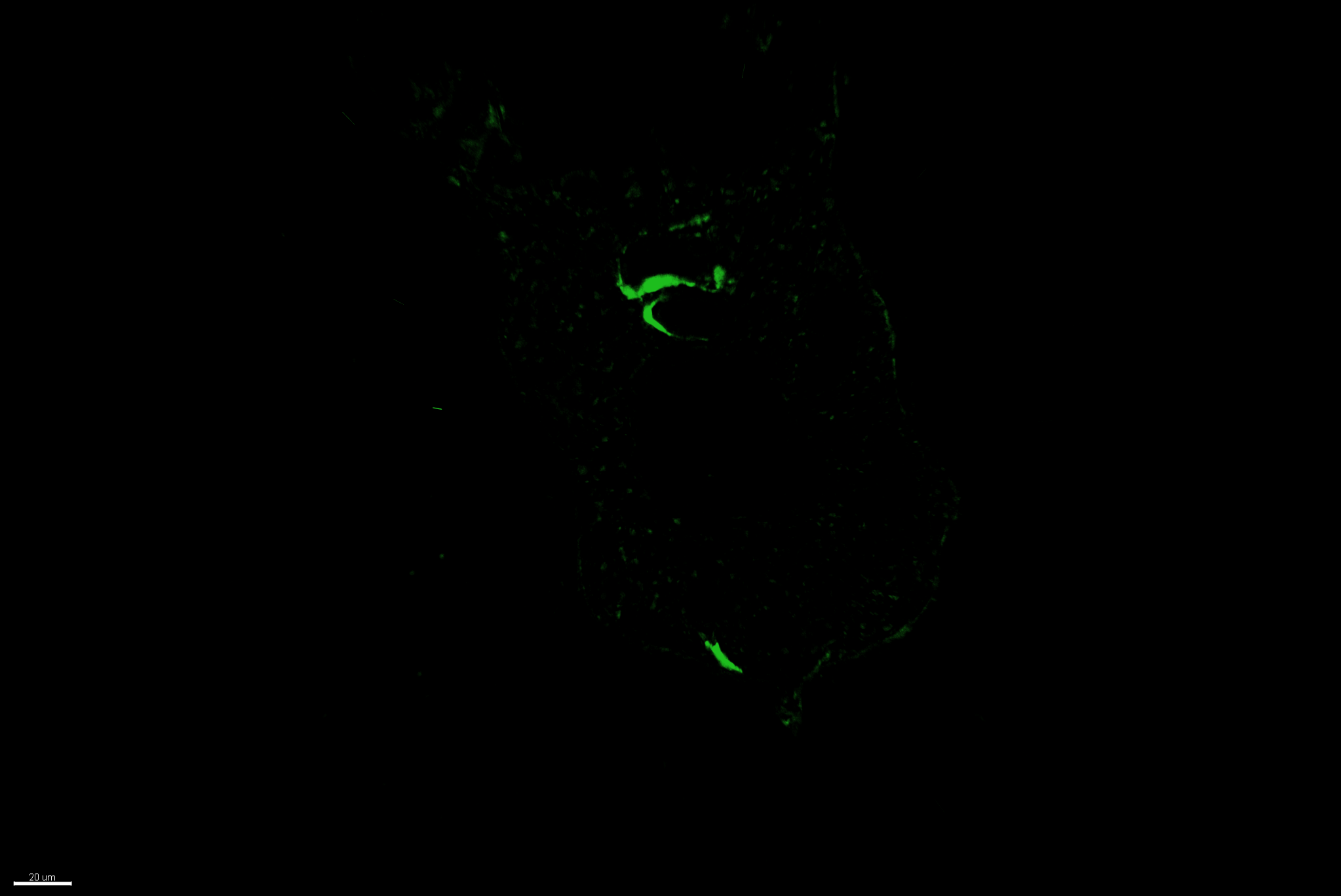

Supplement: Supplementary file 11 — Appendix Figure1-2 Source Data [file 44319_2026_805_MOESM11_ESM.zip › Appendix Source Data 1/Appendix Fig.2/C/2. fli1aEGFP 36hpf controlMO.tif]

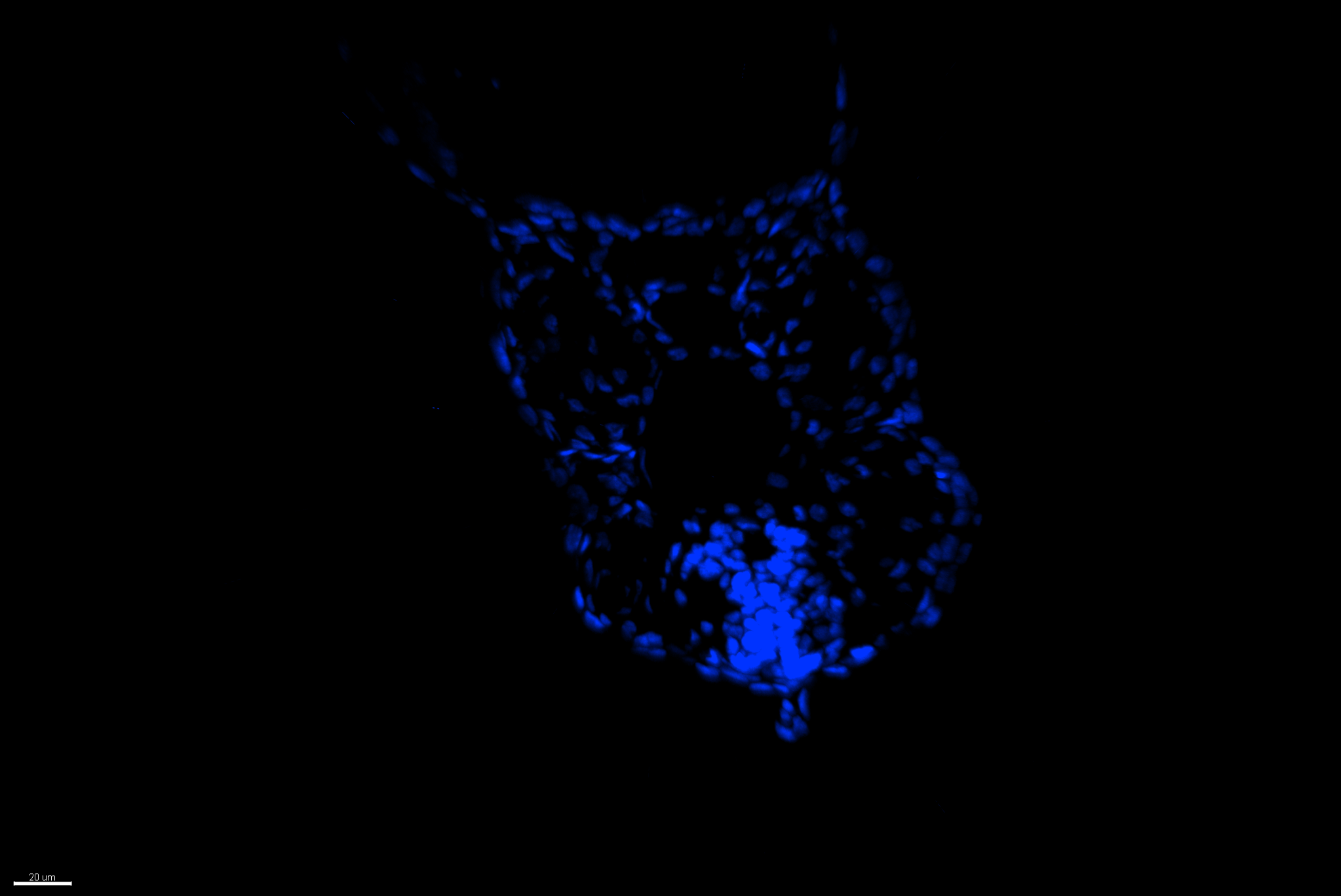

Supplement: Supplementary file 11 — Appendix Figure1-2 Source Data [file 44319_2026_805_MOESM11_ESM.zip › Appendix Source Data 1/Appendix Fig.2/C/3. DAPI 36hpf controlMO.tif]

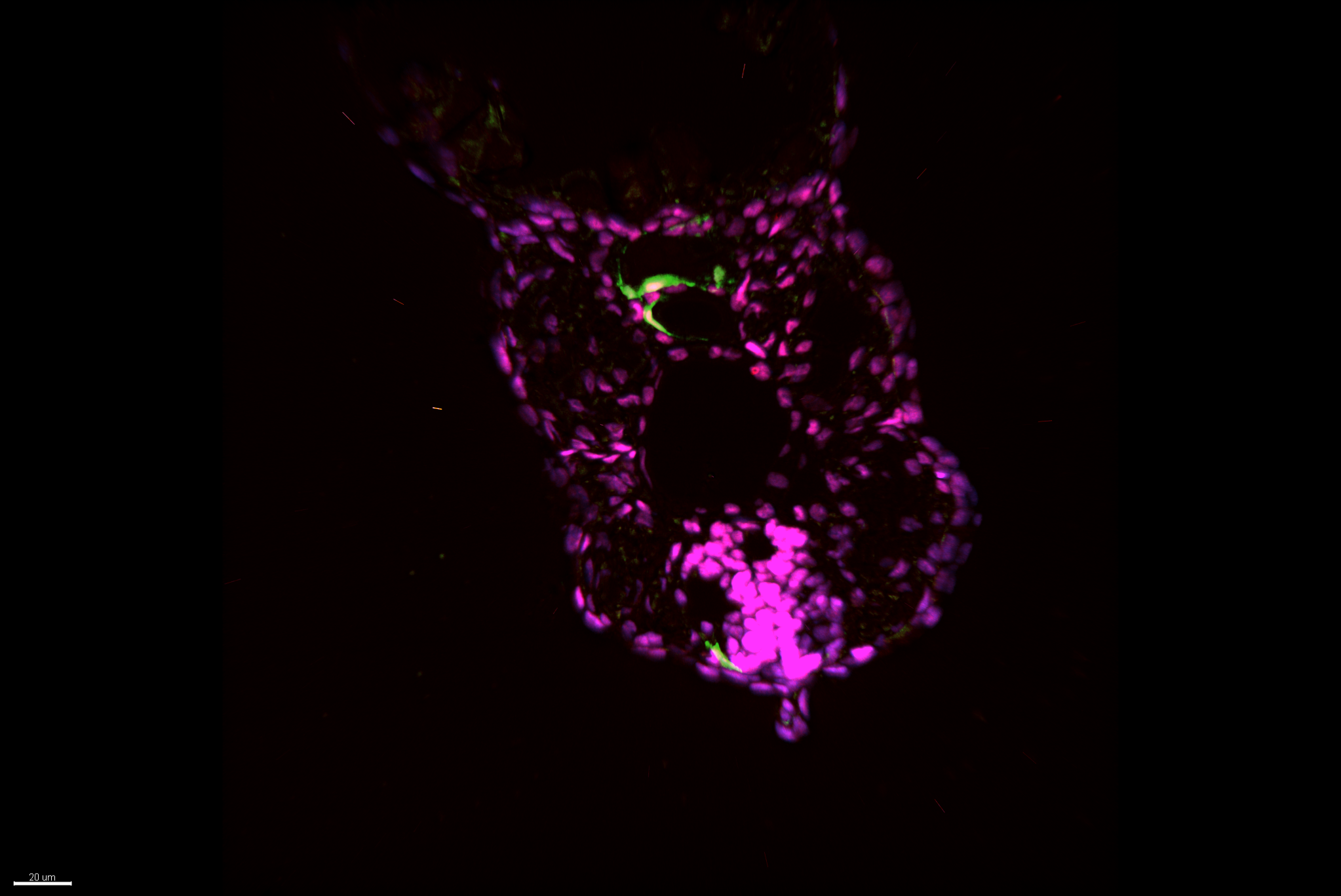

Supplement: Supplementary file 11 — Appendix Figure1-2 Source Data [file 44319_2026_805_MOESM11_ESM.zip › Appendix Source Data 1/Appendix Fig.2/C/4. merge 36hpf controlMO.tif]

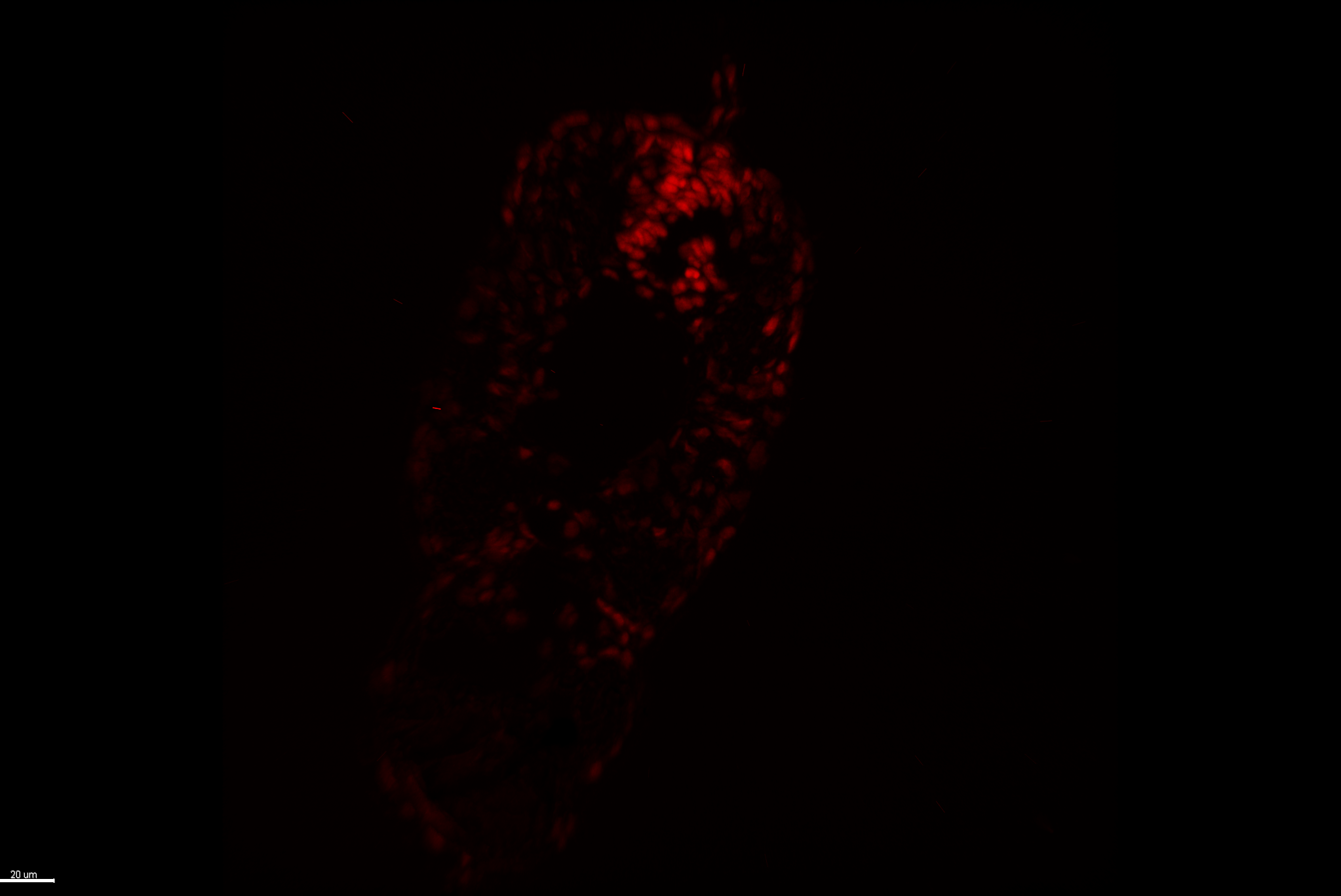

Supplement: Supplementary file 11 — Appendix Figure1-2 Source Data [file 44319_2026_805_MOESM11_ESM.zip › Appendix Source Data 1/Appendix Fig.2/C/5. anti-trmt61a 36hpf trmt61aMO.tif]

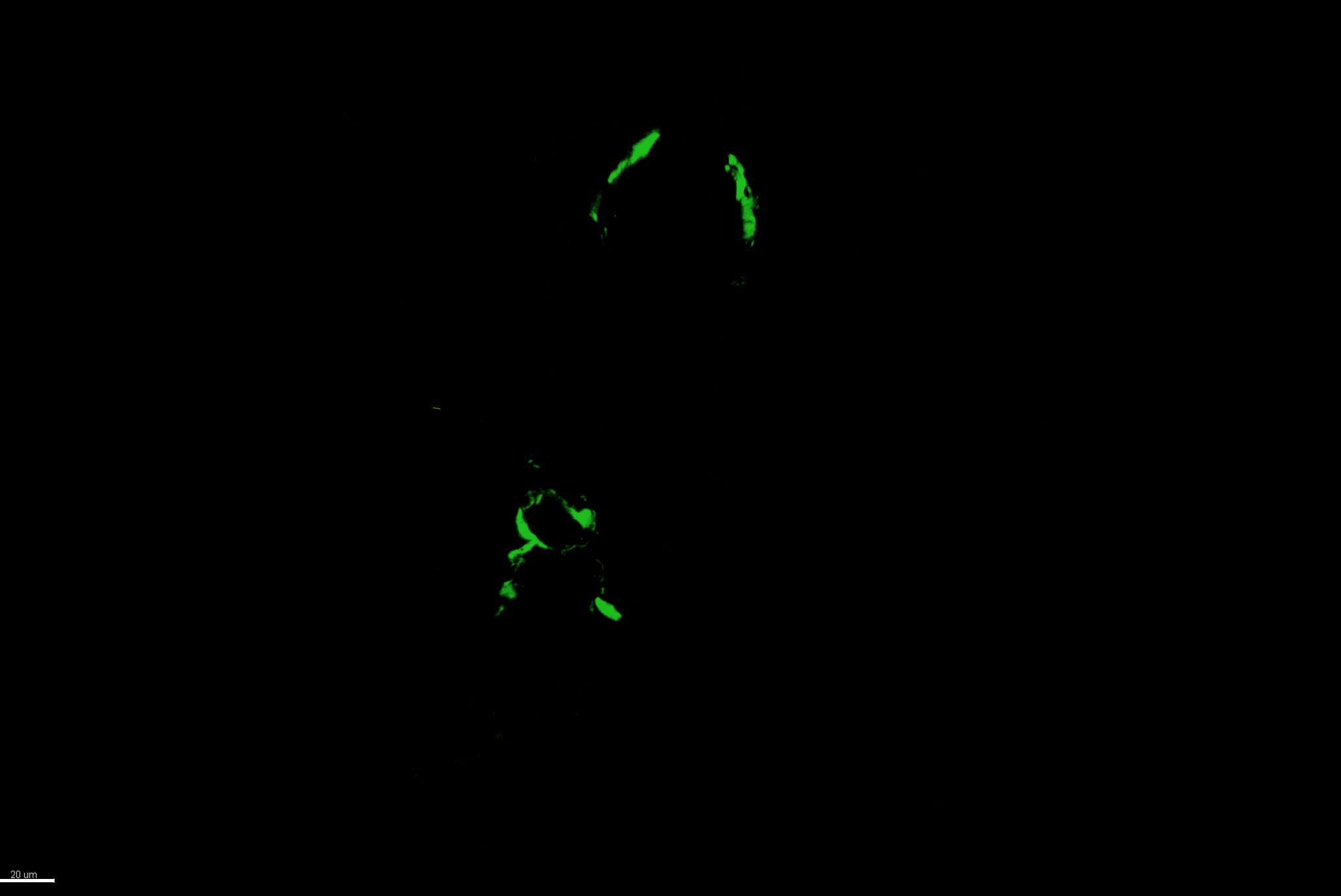

Supplement: Supplementary file 11 — Appendix Figure1-2 Source Data [file 44319_2026_805_MOESM11_ESM.zip › Appendix Source Data 1/Appendix Fig.2/C/6. fli1aEGFP 36hpf trmt61aMO.tif]

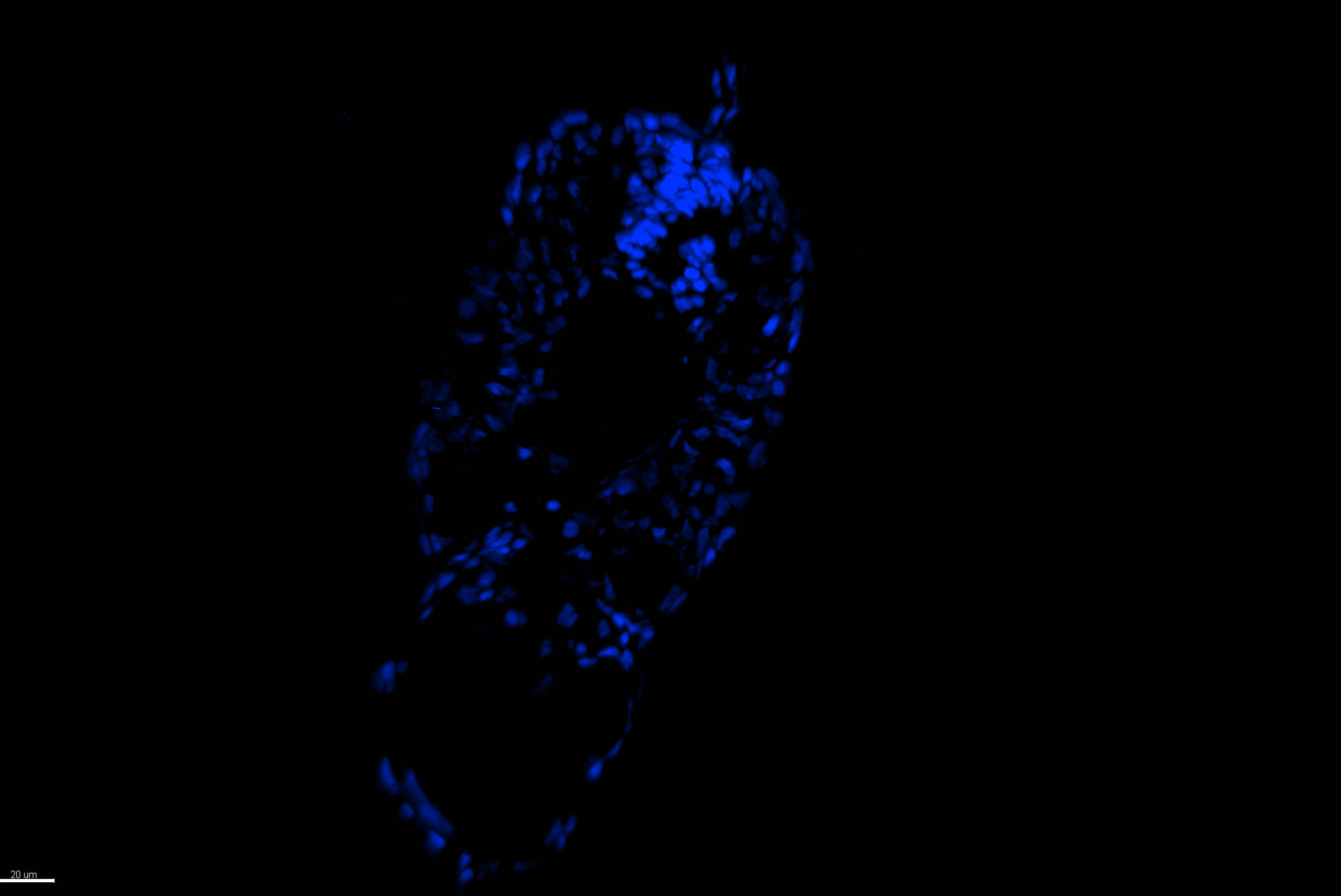

Supplement: Supplementary file 11 — Appendix Figure1-2 Source Data [file 44319_2026_805_MOESM11_ESM.zip › Appendix Source Data 1/Appendix Fig.2/C/7. DAPI 36hpf trmt61aMO.tif]

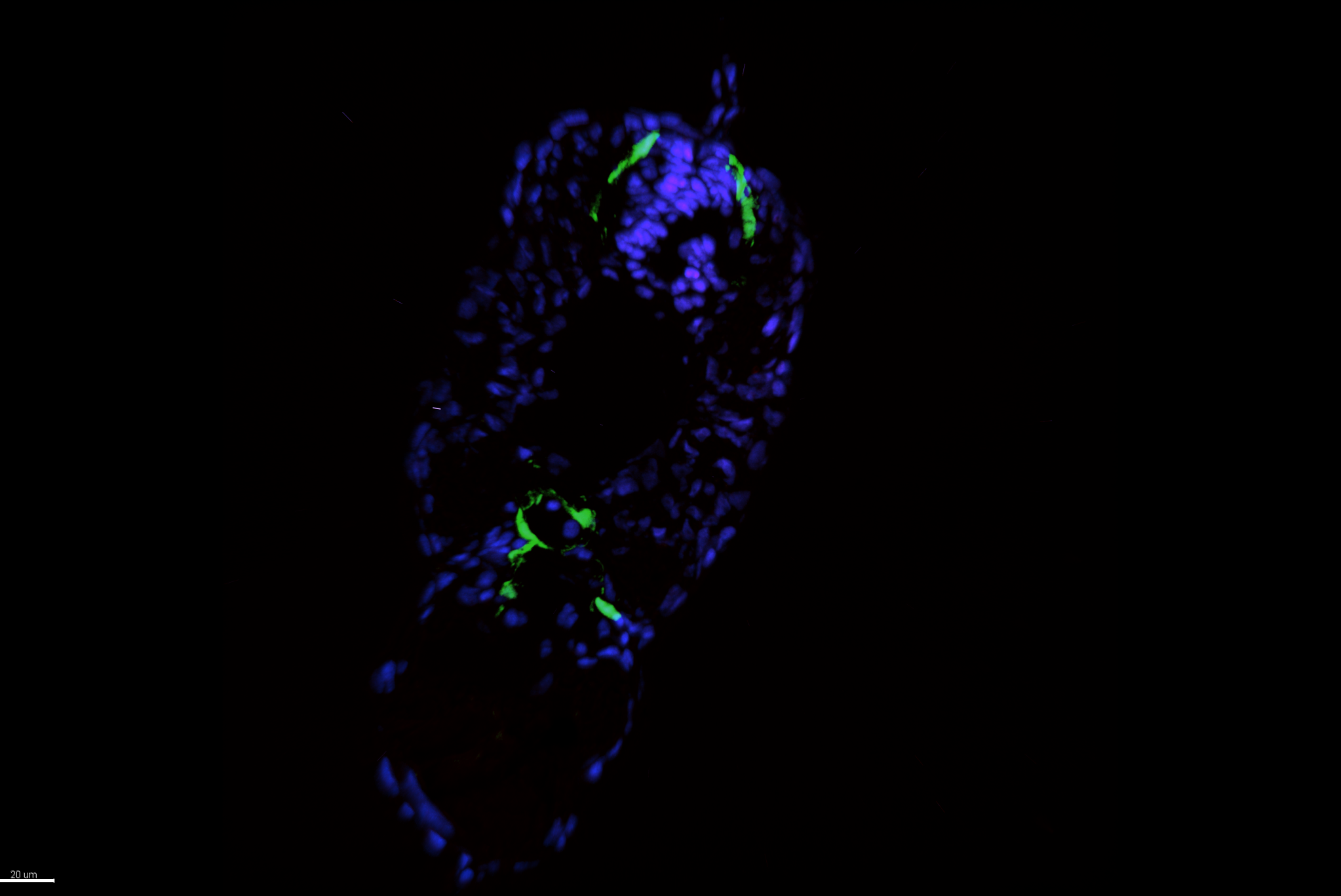

Supplement: Supplementary file 11 — Appendix Figure1-2 Source Data [file 44319_2026_805_MOESM11_ESM.zip › Appendix Source Data 1/Appendix Fig.2/C/8. merge 36hpf trmt61aMO.tif]

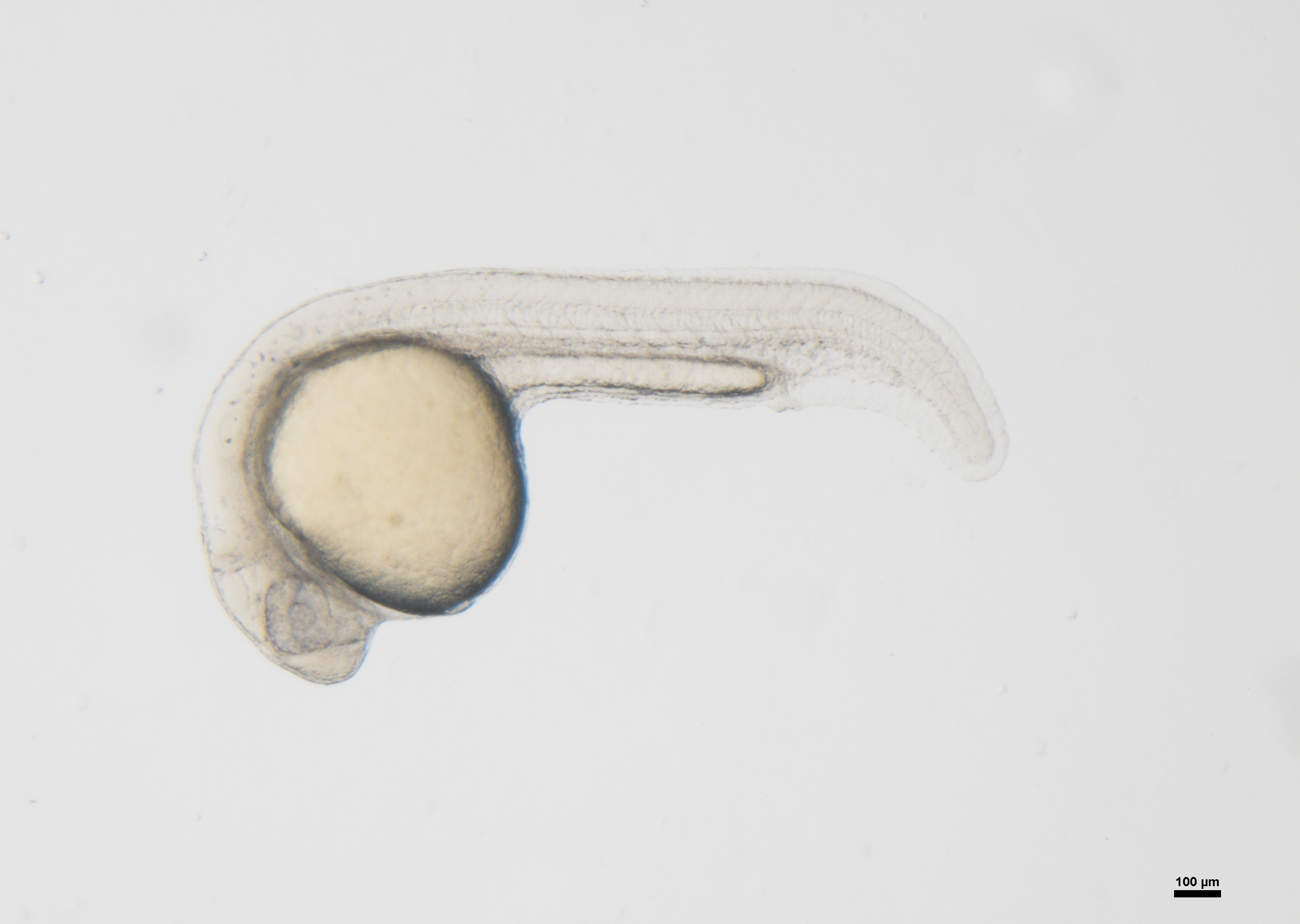

Supplement: Supplementary file 11 — Appendix Figure1-2 Source Data [file 44319_2026_805_MOESM11_ESM.zip › Appendix Source Data 1/Appendix Fig.2/D/1. 24hpf controlMO.tif]

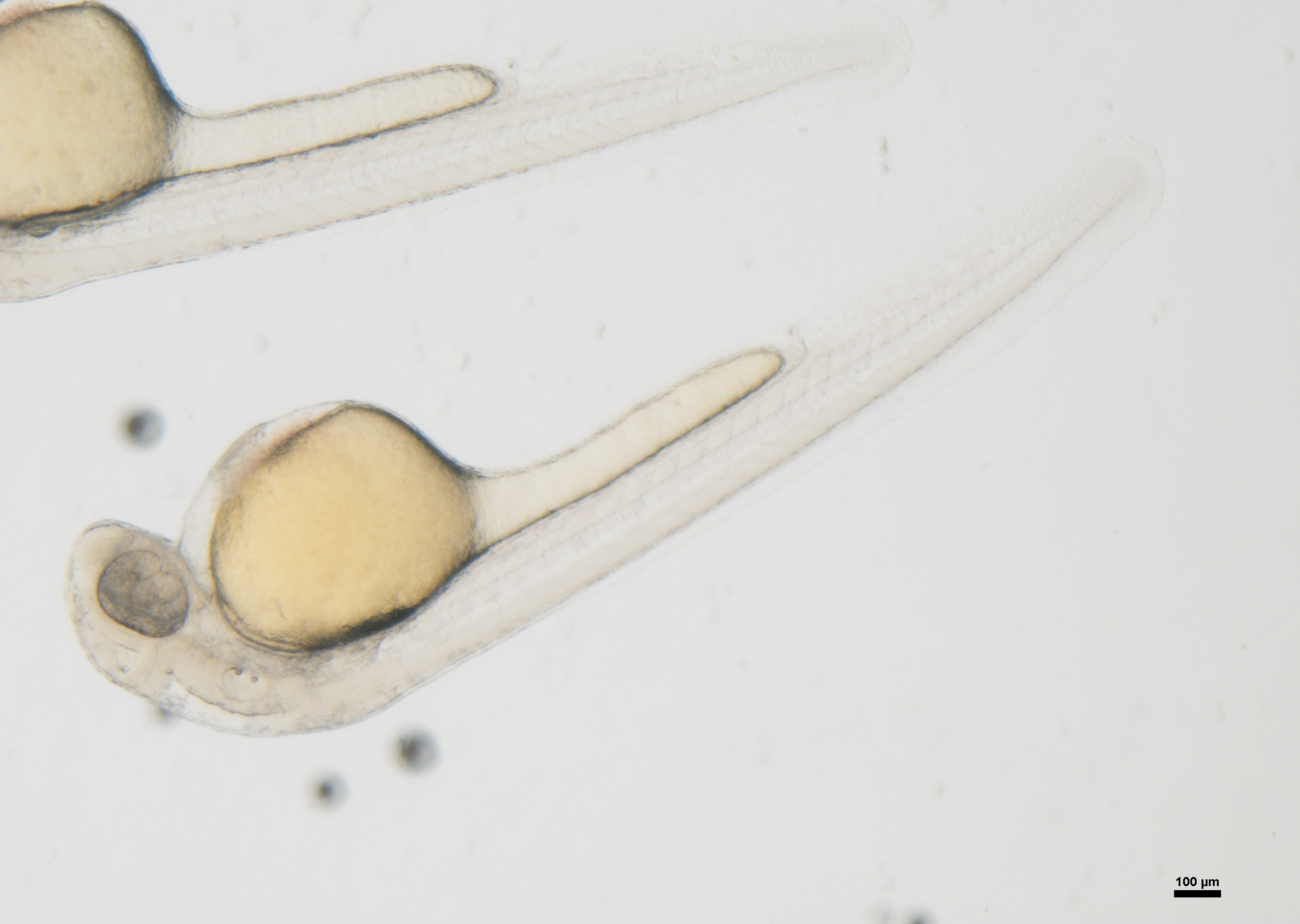

Supplement: Supplementary file 11 — Appendix Figure1-2 Source Data [file 44319_2026_805_MOESM11_ESM.zip › Appendix Source Data 1/Appendix Fig.2/D/2. 36hpf controlMO.tif]

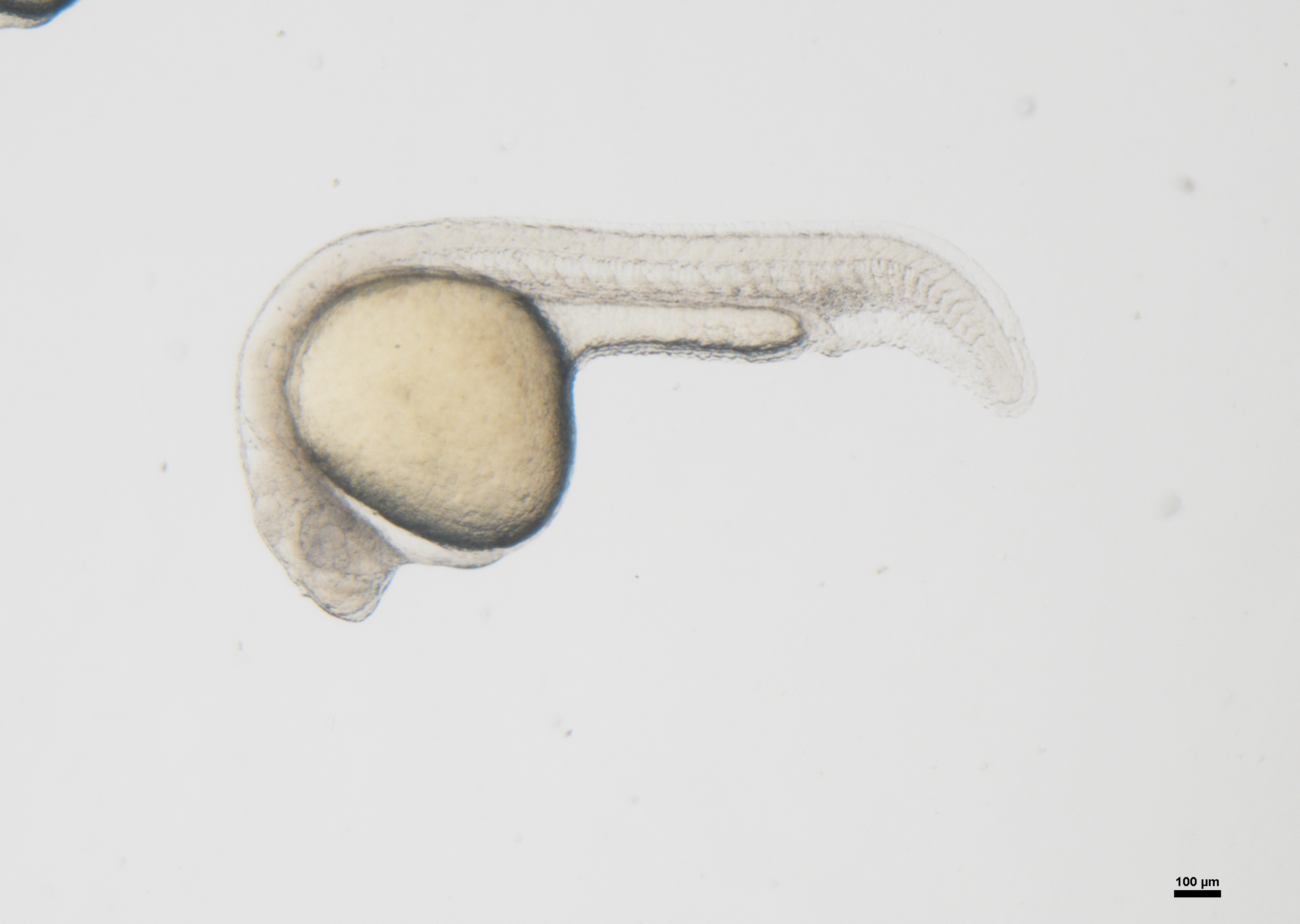

Supplement: Supplementary file 11 — Appendix Figure1-2 Source Data [file 44319_2026_805_MOESM11_ESM.zip › Appendix Source Data 1/Appendix Fig.2/D/3. 24hpf trmt61aMO.tif]

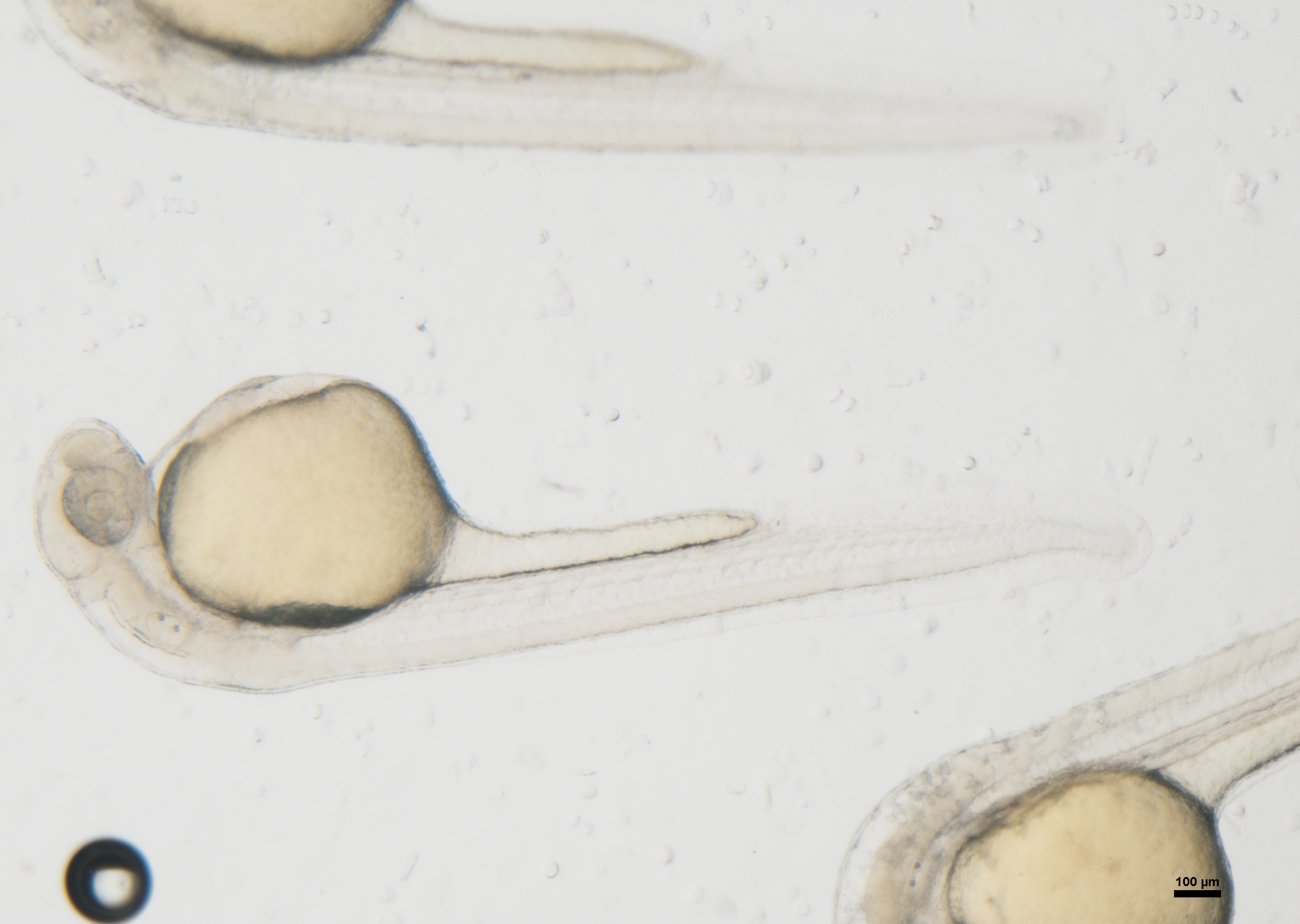

Supplement: Supplementary file 11 — Appendix Figure1-2 Source Data [file 44319_2026_805_MOESM11_ESM.zip › Appendix Source Data 1/Appendix Fig.2/D/4. 36hpf trmt61aMO.tif]

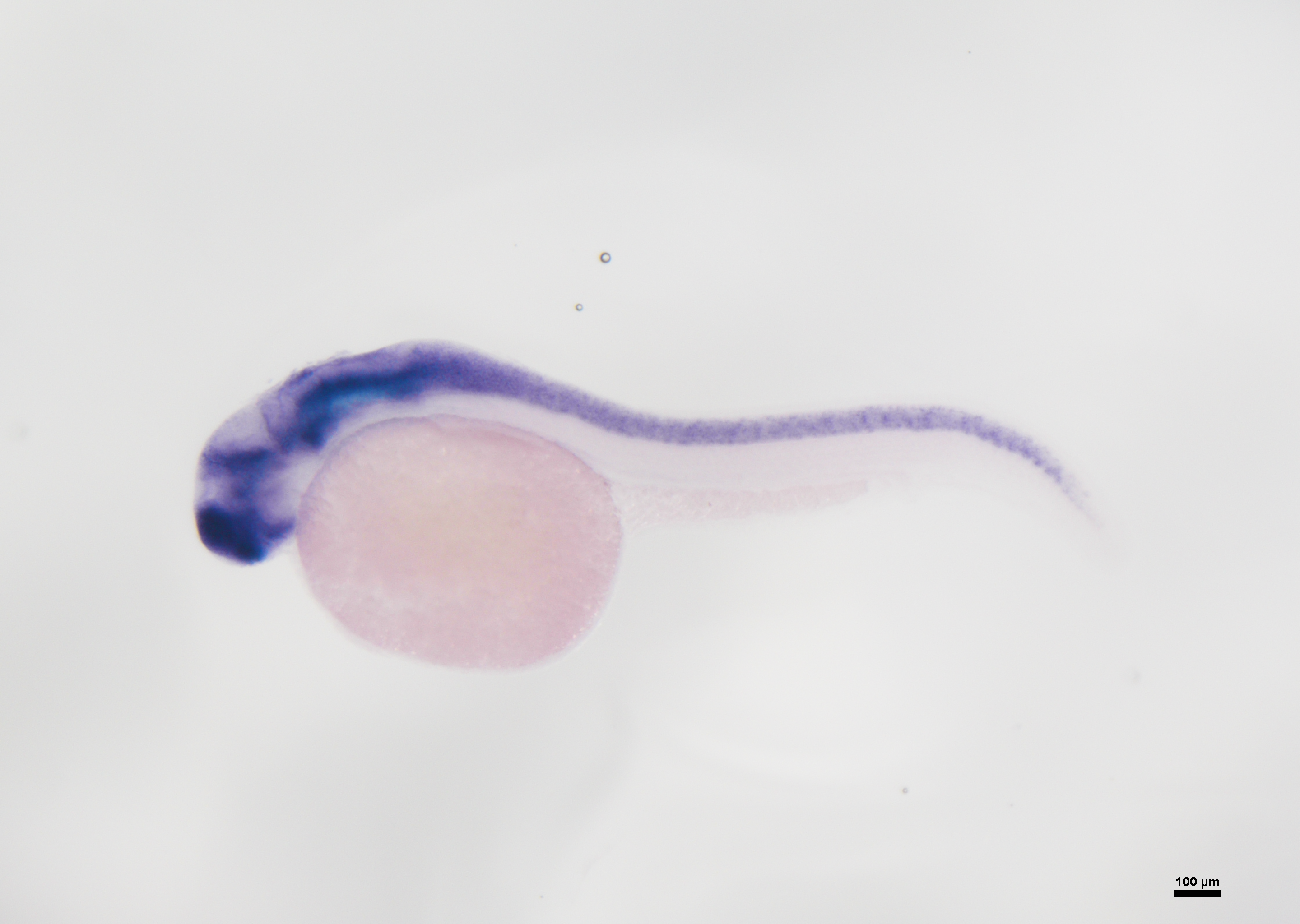

Supplement: Supplementary file 11 — Appendix Figure1-2 Source Data [file 44319_2026_805_MOESM11_ESM.zip › Appendix Source Data 1/Appendix Fig.2/E/1. elavl3 36hpf controlMO.tif]

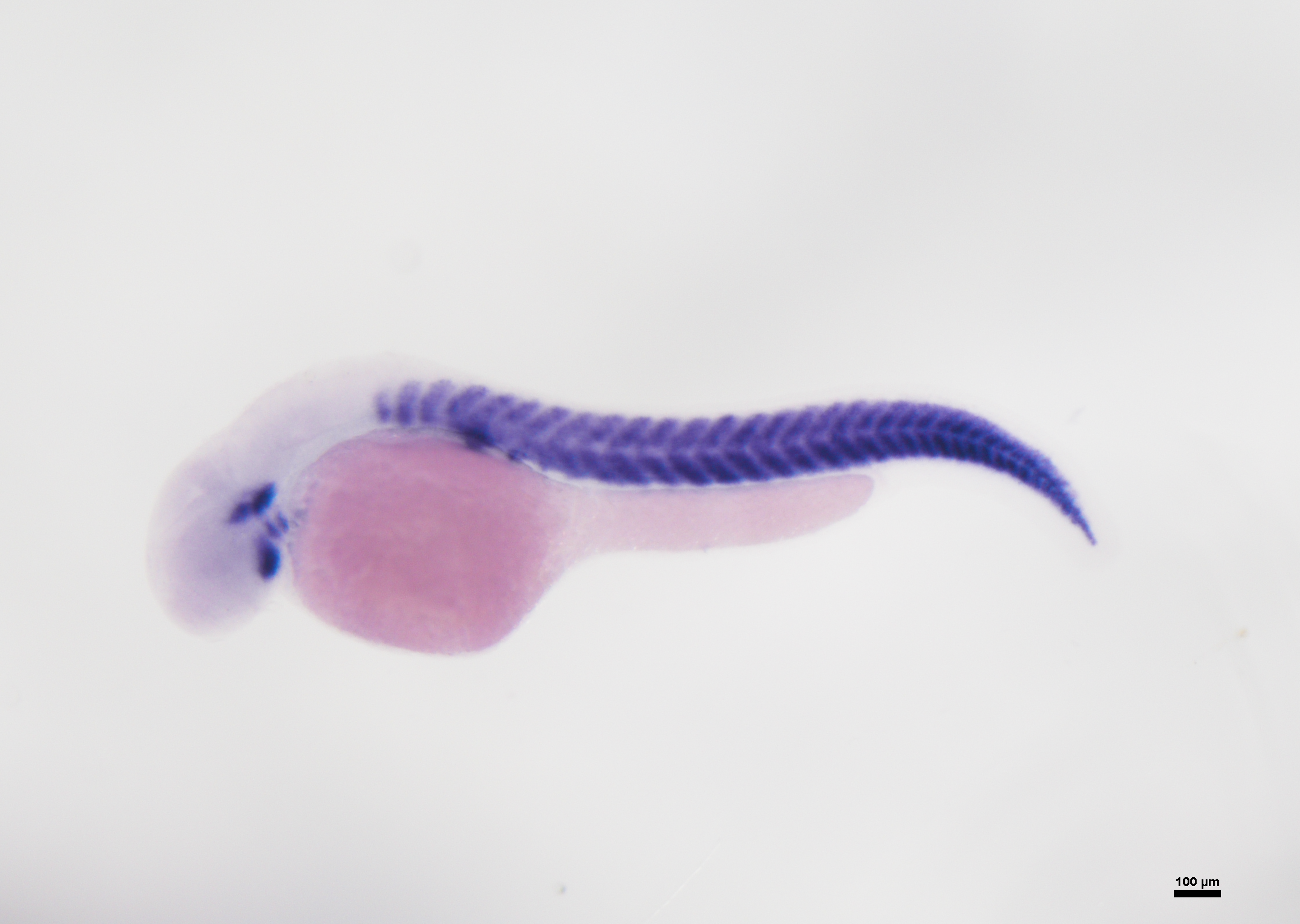

Supplement: Supplementary file 11 — Appendix Figure1-2 Source Data [file 44319_2026_805_MOESM11_ESM.zip › Appendix Source Data 1/Appendix Fig.2/E/10. moyd1 36hpf trmt61aMO.tif]

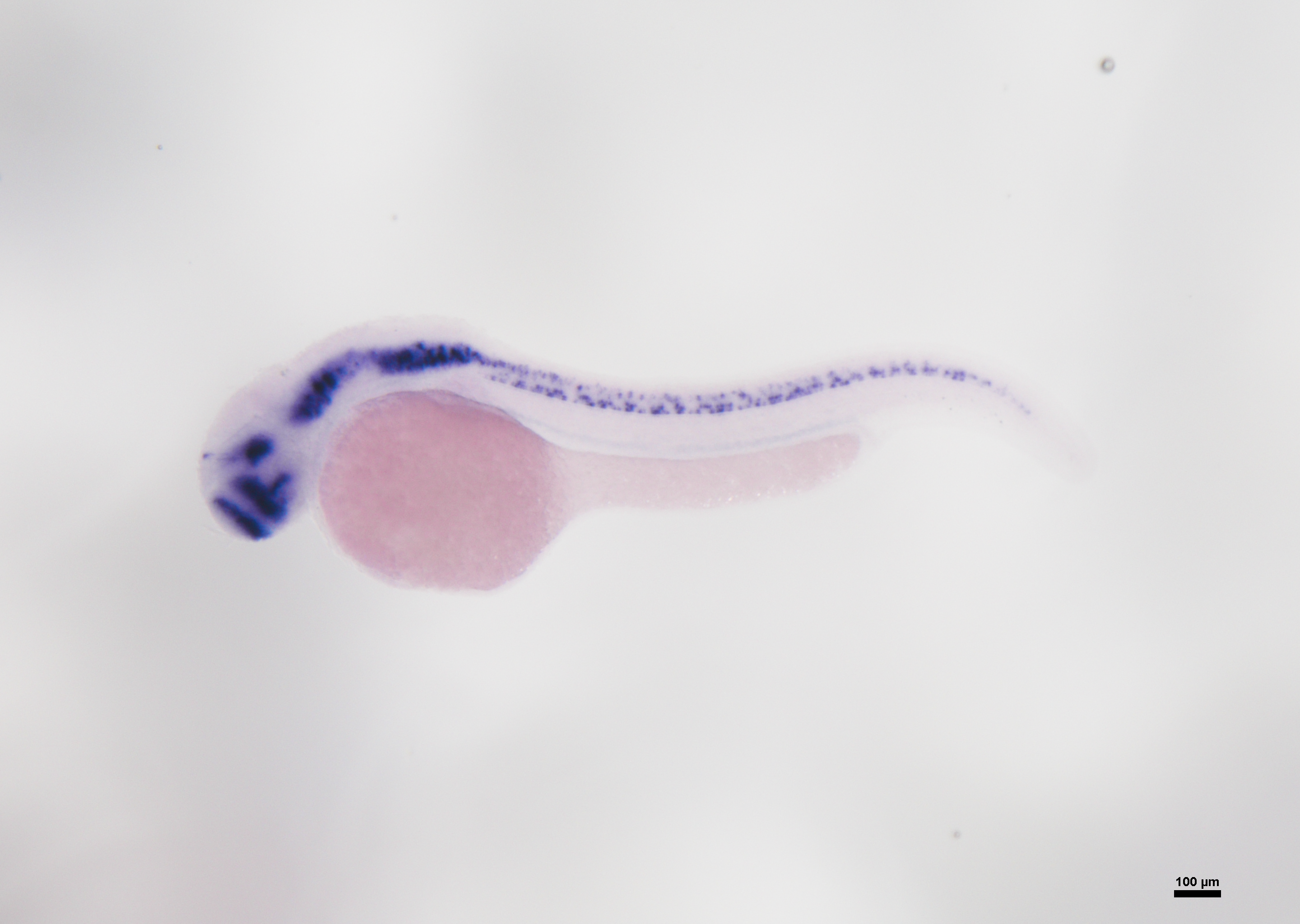

Supplement: Supplementary file 11 — Appendix Figure1-2 Source Data [file 44319_2026_805_MOESM11_ESM.zip › Appendix Source Data 1/Appendix Fig.2/E/2. gad1b 36hpf controlMO.tif]

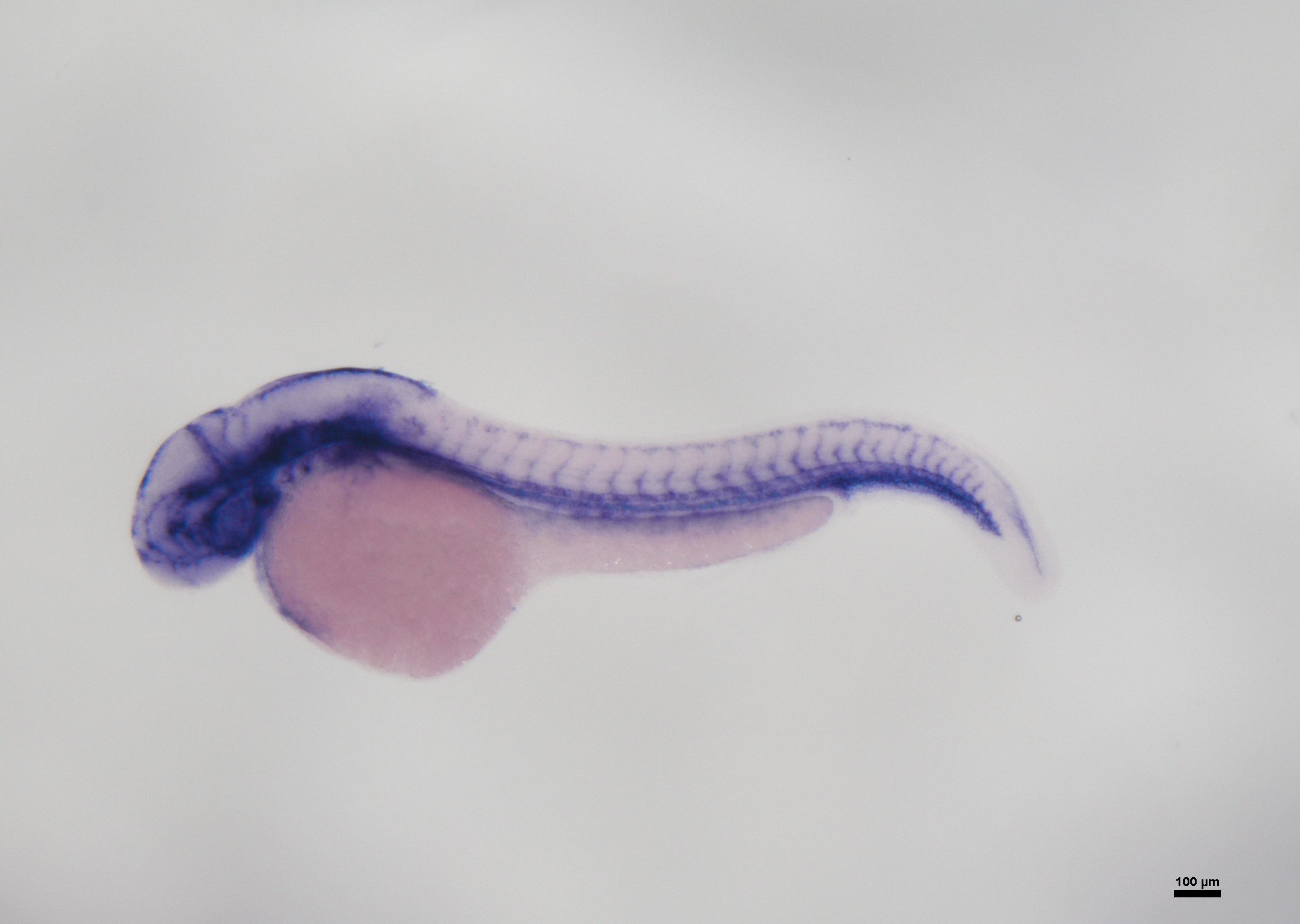

Supplement: Supplementary file 11 — Appendix Figure1-2 Source Data [file 44319_2026_805_MOESM11_ESM.zip › Appendix Source Data 1/Appendix Fig.2/E/3. kdrl 36hpf controlMO.tif]

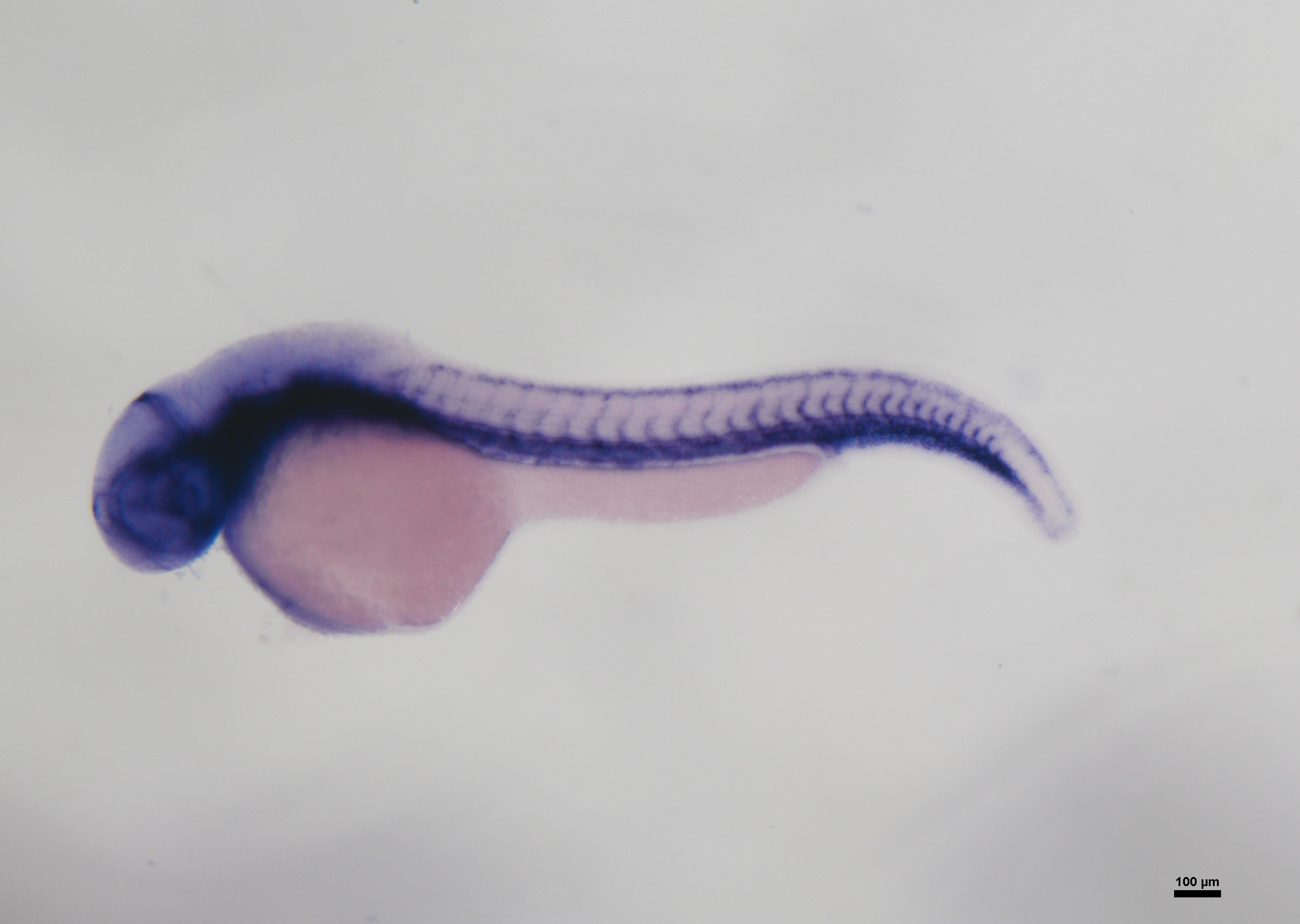

Supplement: Supplementary file 11 — Appendix Figure1-2 Source Data [file 44319_2026_805_MOESM11_ESM.zip › Appendix Source Data 1/Appendix Fig.2/E/4. fli1a 36hpf controlMO.tif]

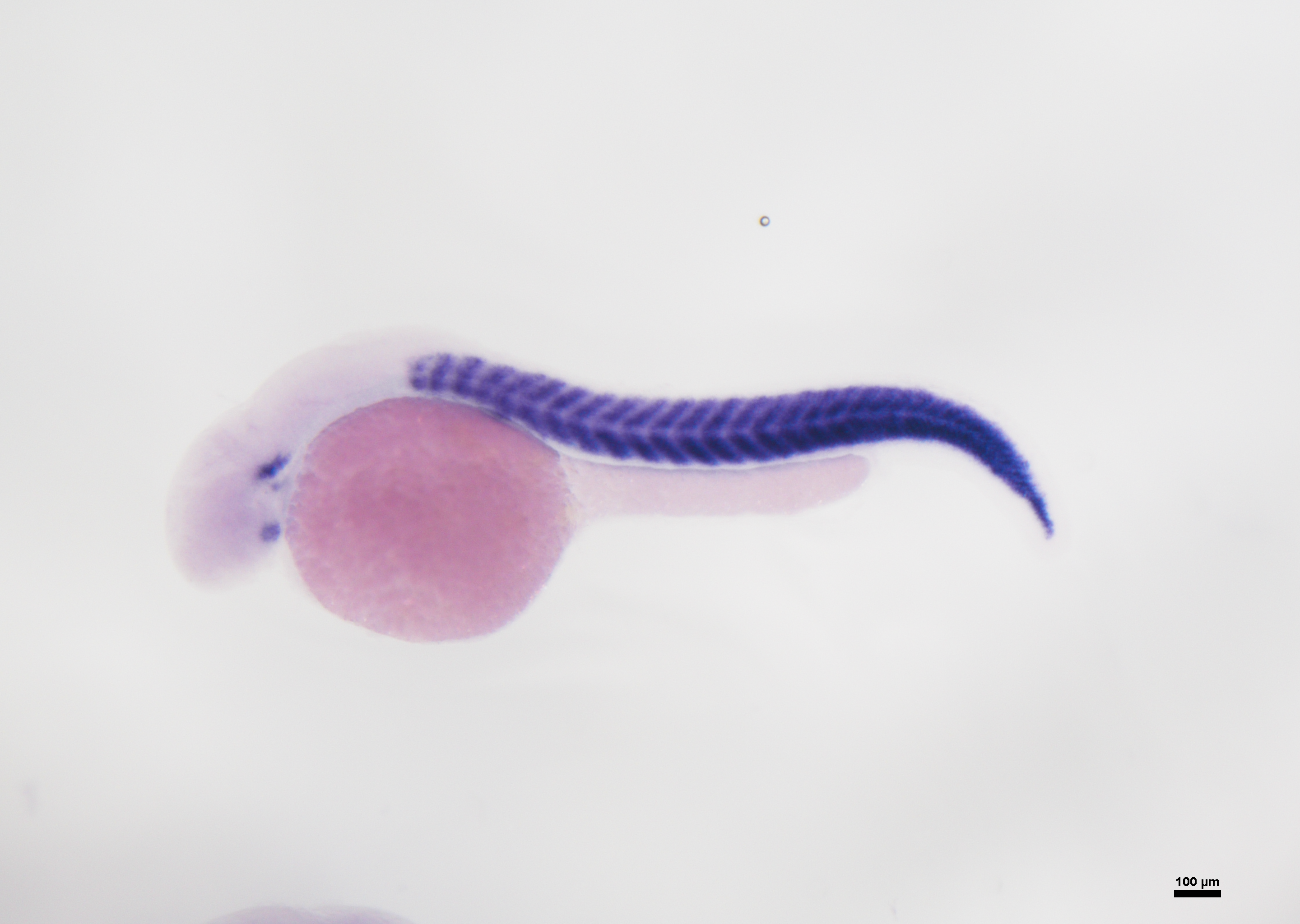

Supplement: Supplementary file 11 — Appendix Figure1-2 Source Data [file 44319_2026_805_MOESM11_ESM.zip › Appendix Source Data 1/Appendix Fig.2/E/5. myod1 36hpf controlMO.tif]

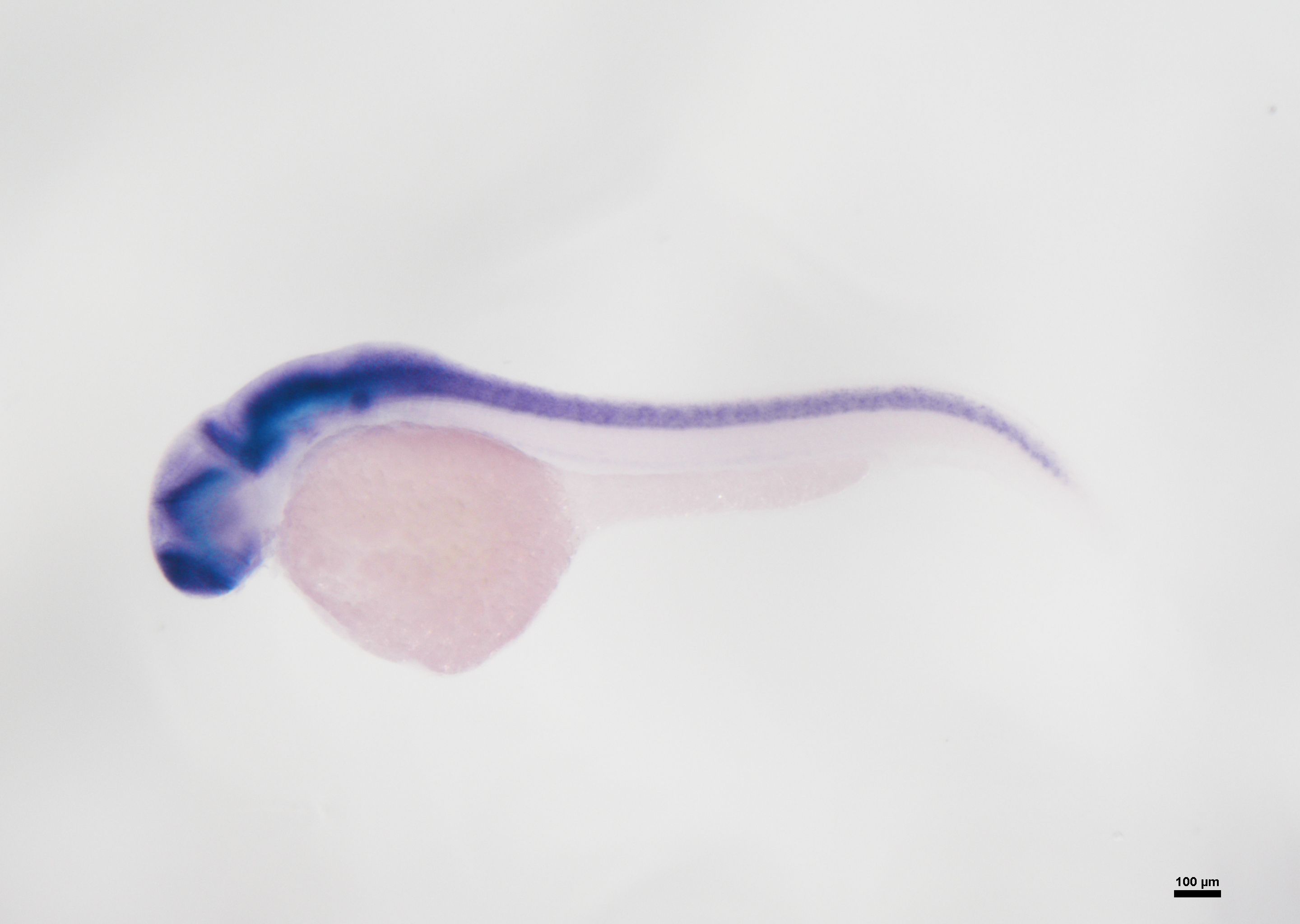

Supplement: Supplementary file 11 — Appendix Figure1-2 Source Data [file 44319_2026_805_MOESM11_ESM.zip › Appendix Source Data 1/Appendix Fig.2/E/6. elavl3 36hpf trmt61aMO.tif]

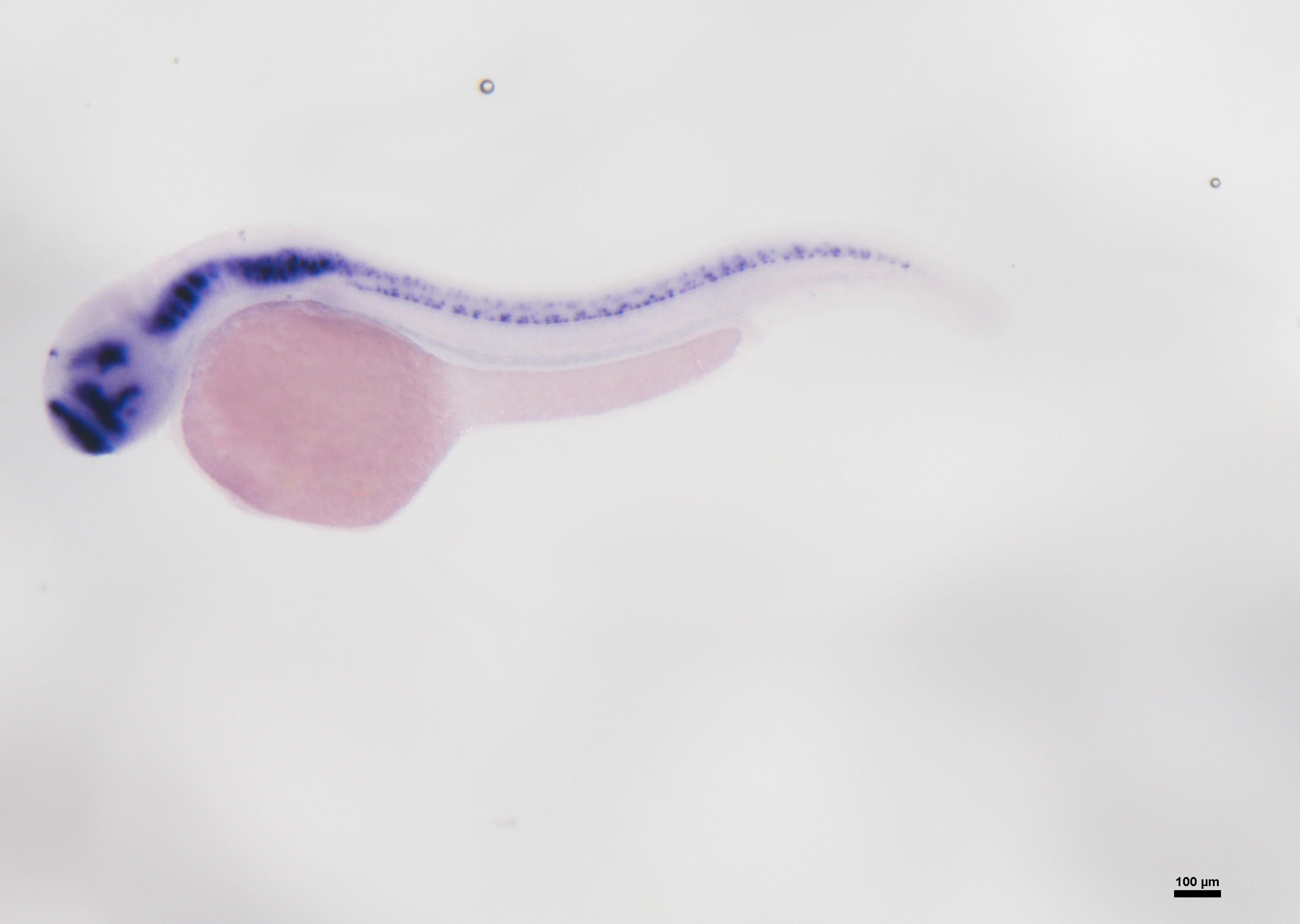

Supplement: Supplementary file 11 — Appendix Figure1-2 Source Data [file 44319_2026_805_MOESM11_ESM.zip › Appendix Source Data 1/Appendix Fig.2/E/7. gad1b 36hpf trmt61aMO.tif]

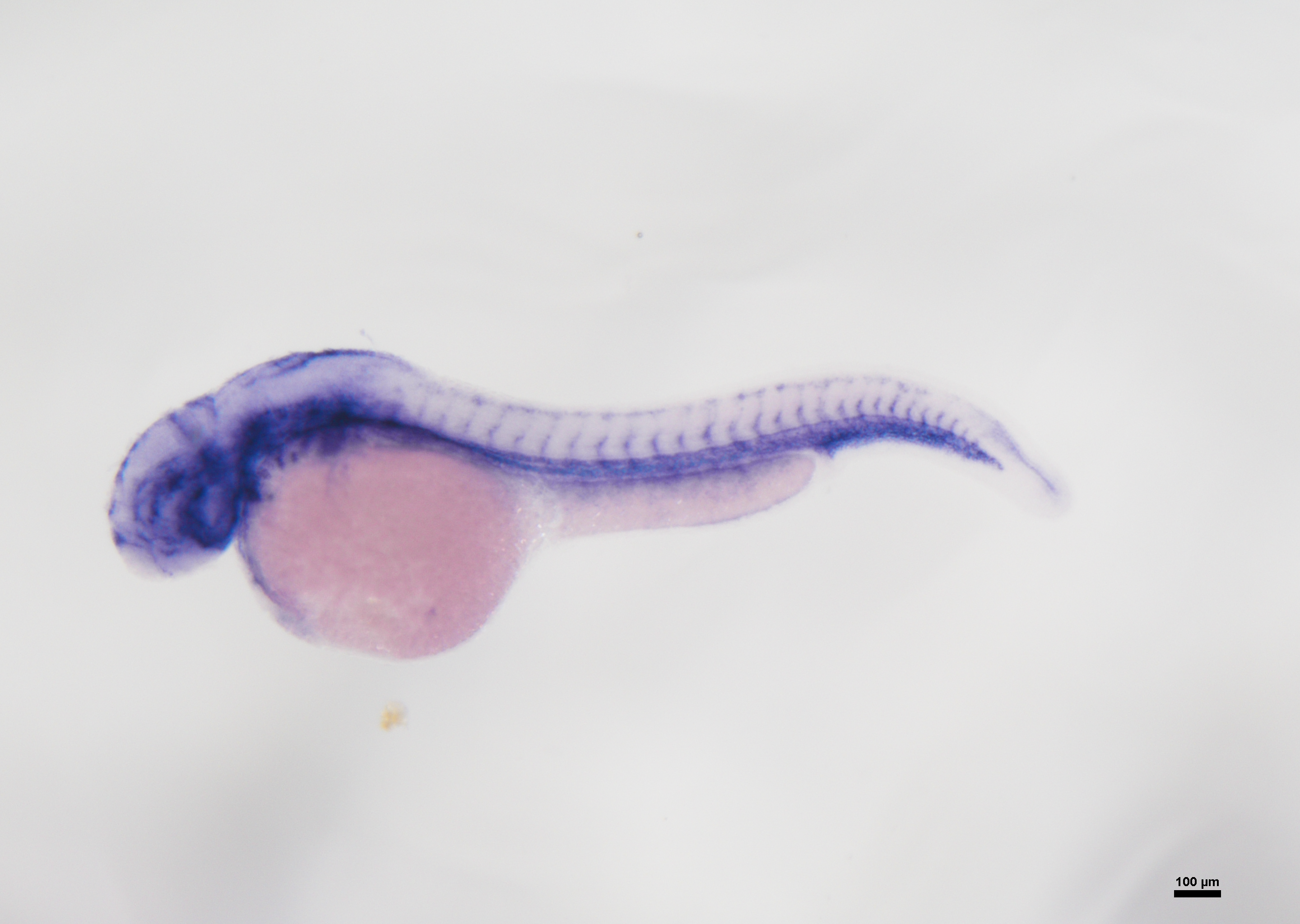

Supplement: Supplementary file 11 — Appendix Figure1-2 Source Data [file 44319_2026_805_MOESM11_ESM.zip › Appendix Source Data 1/Appendix Fig.2/E/8. kdrl 36hpf trmt61aMO.tif]

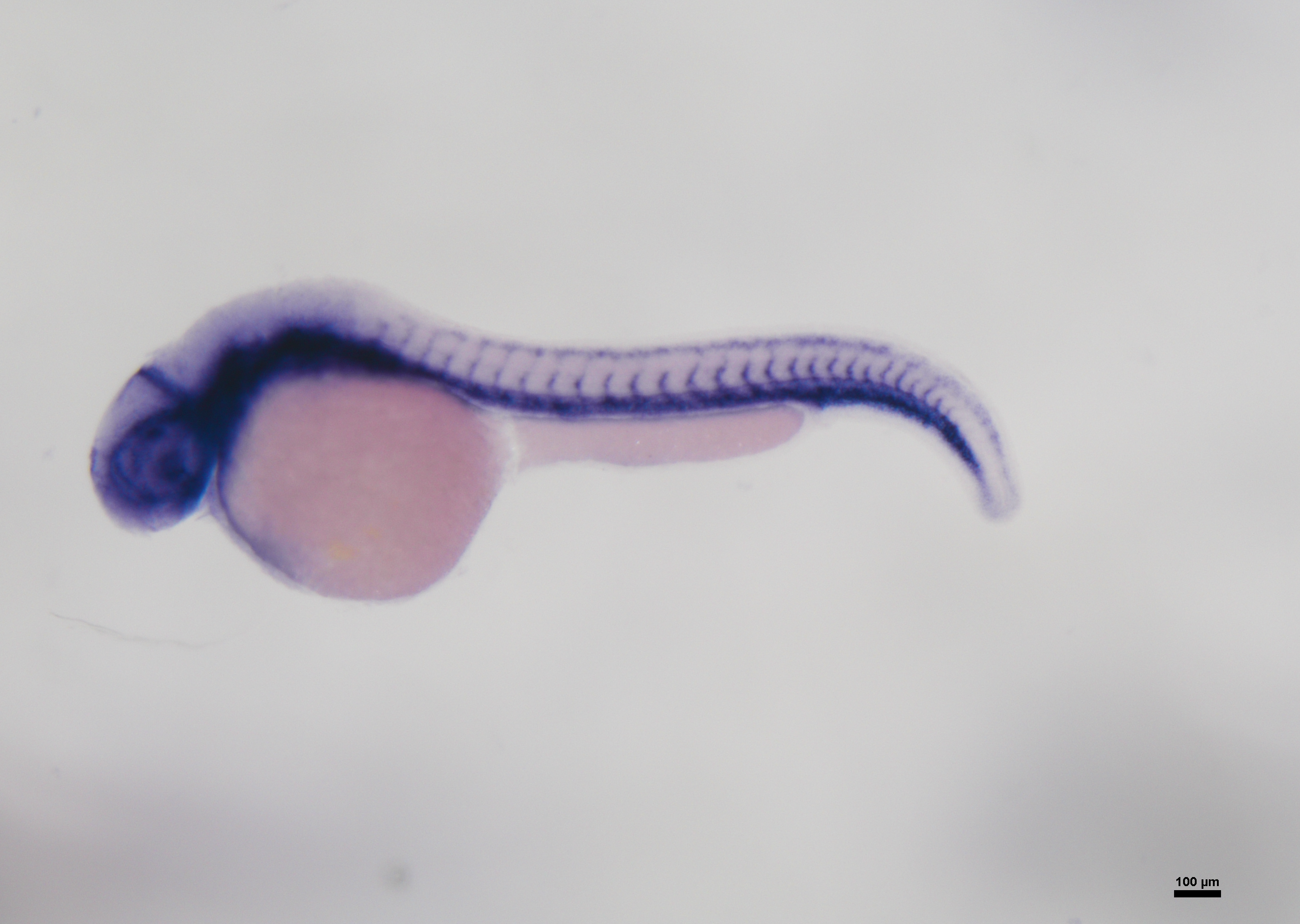

Supplement: Supplementary file 11 — Appendix Figure1-2 Source Data [file 44319_2026_805_MOESM11_ESM.zip › Appendix Source Data 1/Appendix Fig.2/E/9. fli1a 36hpf trmt61aMO.tif]

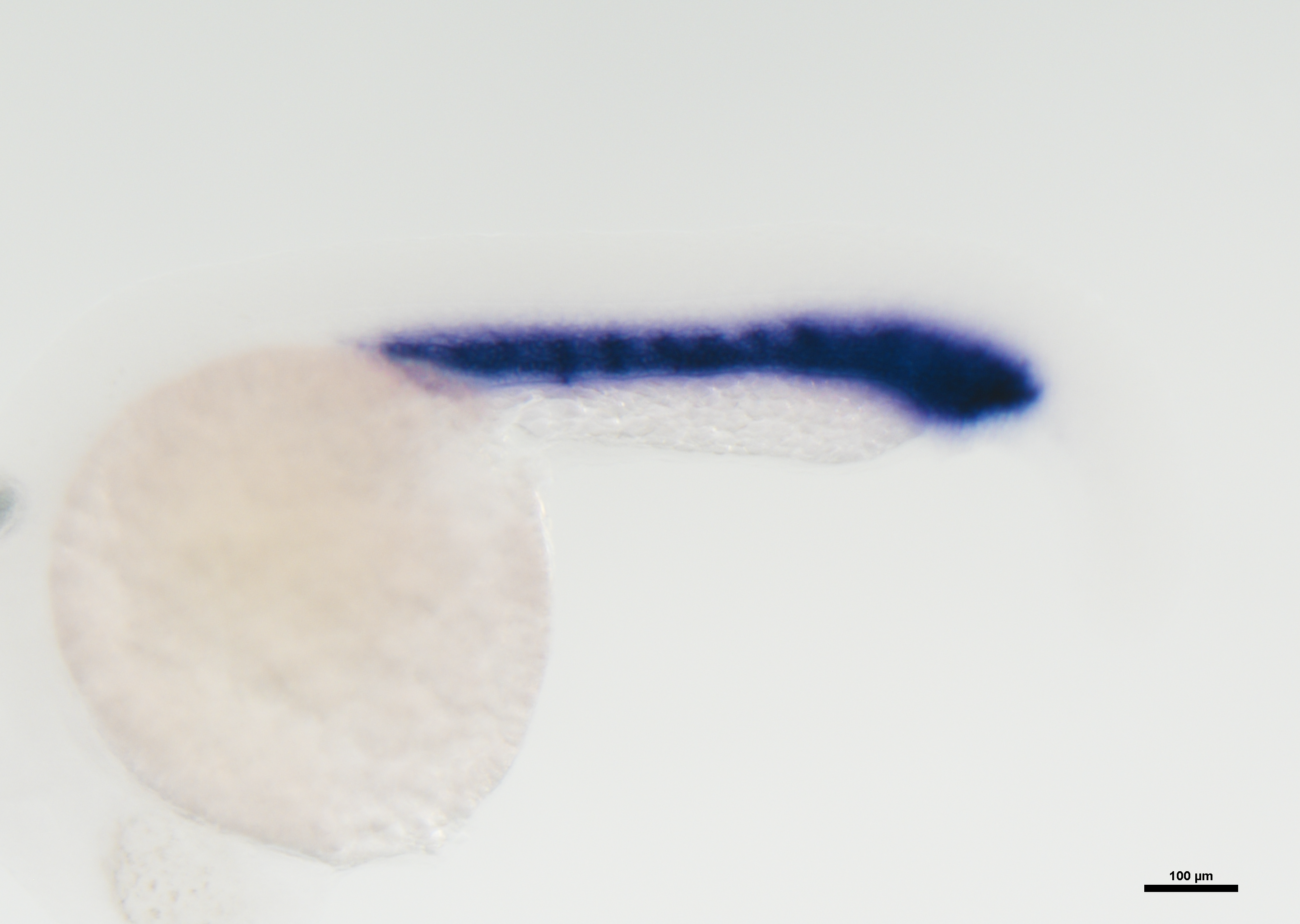

Supplement: Supplementary file 11 — Appendix Figure1-2 Source Data [file 44319_2026_805_MOESM11_ESM.zip › Appendix Source Data 1/Appendix Fig.2/F/1. gata1a 24hpf controlMO.tif]

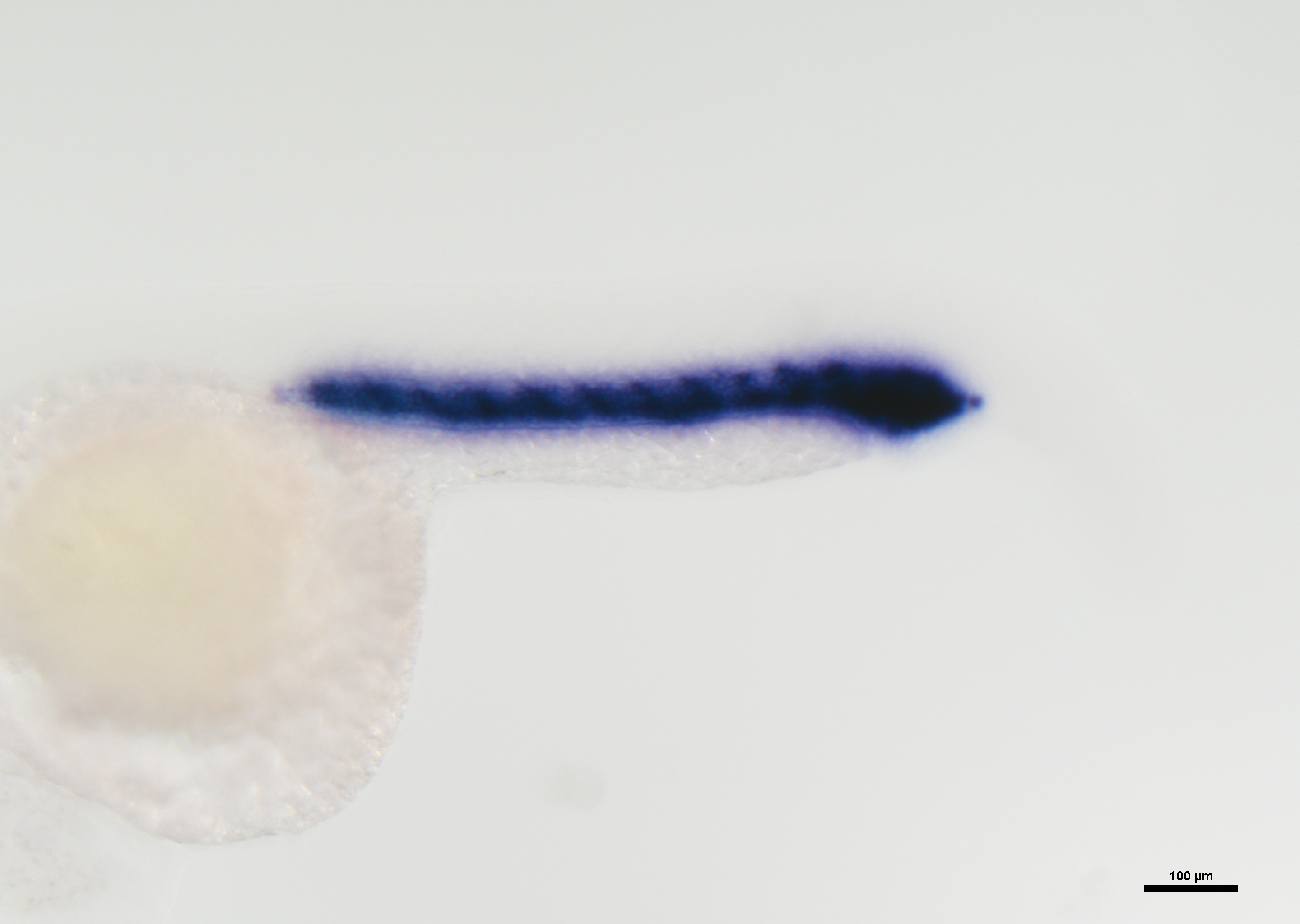

Supplement: Supplementary file 11 — Appendix Figure1-2 Source Data [file 44319_2026_805_MOESM11_ESM.zip › Appendix Source Data 1/Appendix Fig.2/F/2. gata1a 24hpf trmt61aMO.tif]

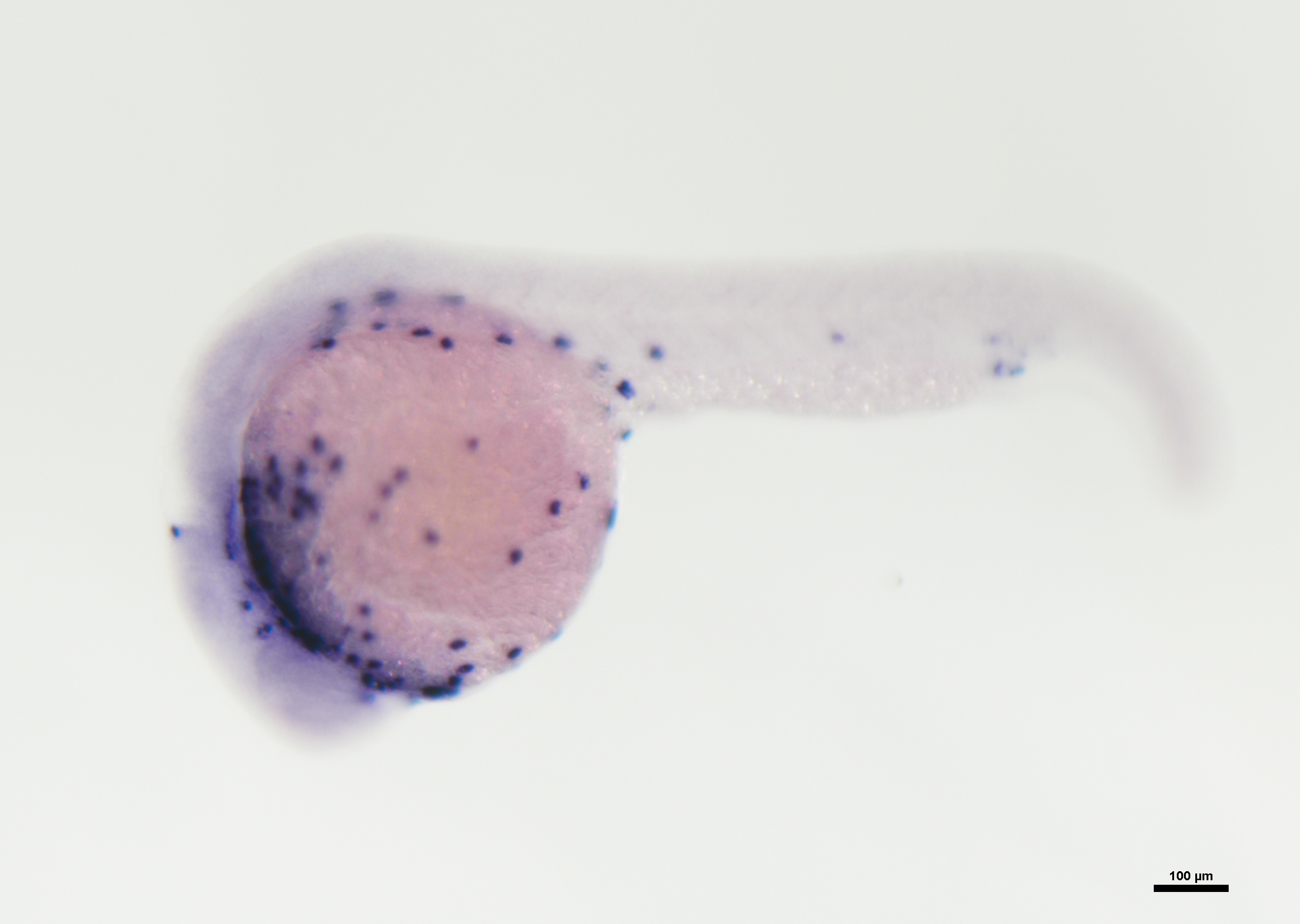

Supplement: Supplementary file 11 — Appendix Figure1-2 Source Data [file 44319_2026_805_MOESM11_ESM.zip › Appendix Source Data 1/Appendix Fig.2/F/3. pu.1 24hpf controlMO.tif]

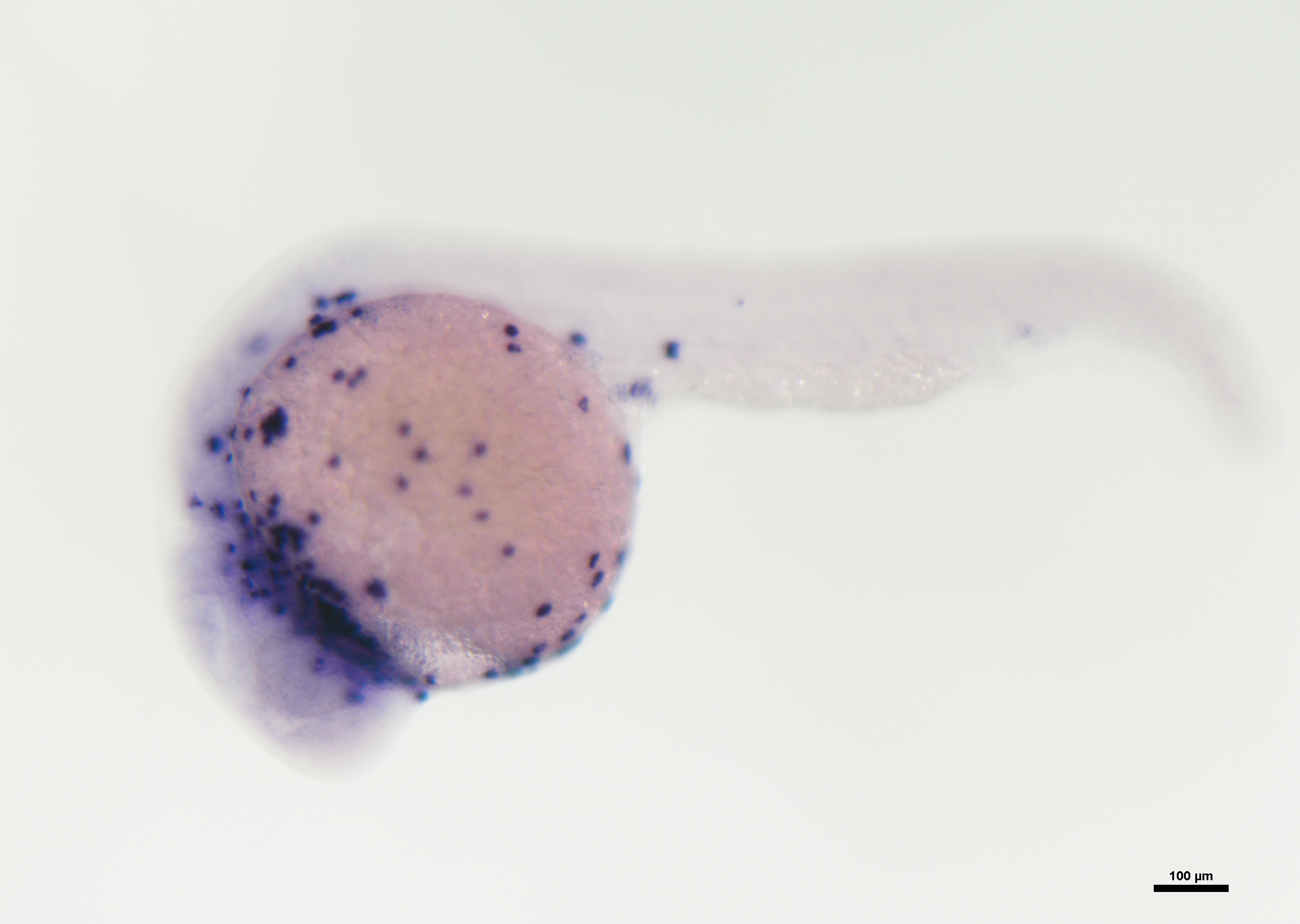

Supplement: Supplementary file 11 — Appendix Figure1-2 Source Data [file 44319_2026_805_MOESM11_ESM.zip › Appendix Source Data 1/Appendix Fig.2/F/4. pu.1 24hpf trmt61aMO.tif]

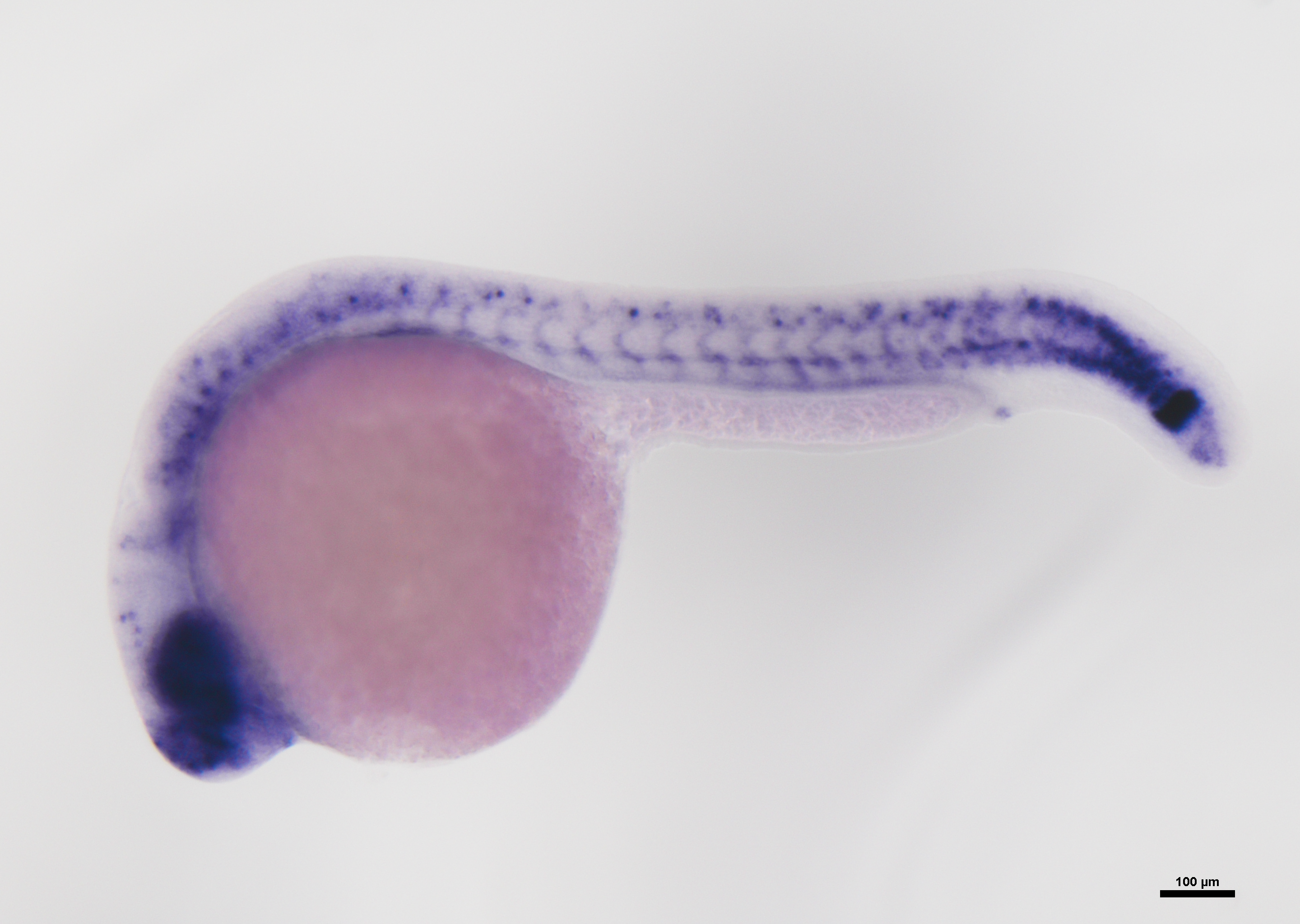

Supplement: Supplementary file 11 — Appendix Figure1-2 Source Data [file 44319_2026_805_MOESM11_ESM.zip › Appendix Source Data 1/Appendix Fig.2/I/1. dltc 24hpf controlMO.tif]

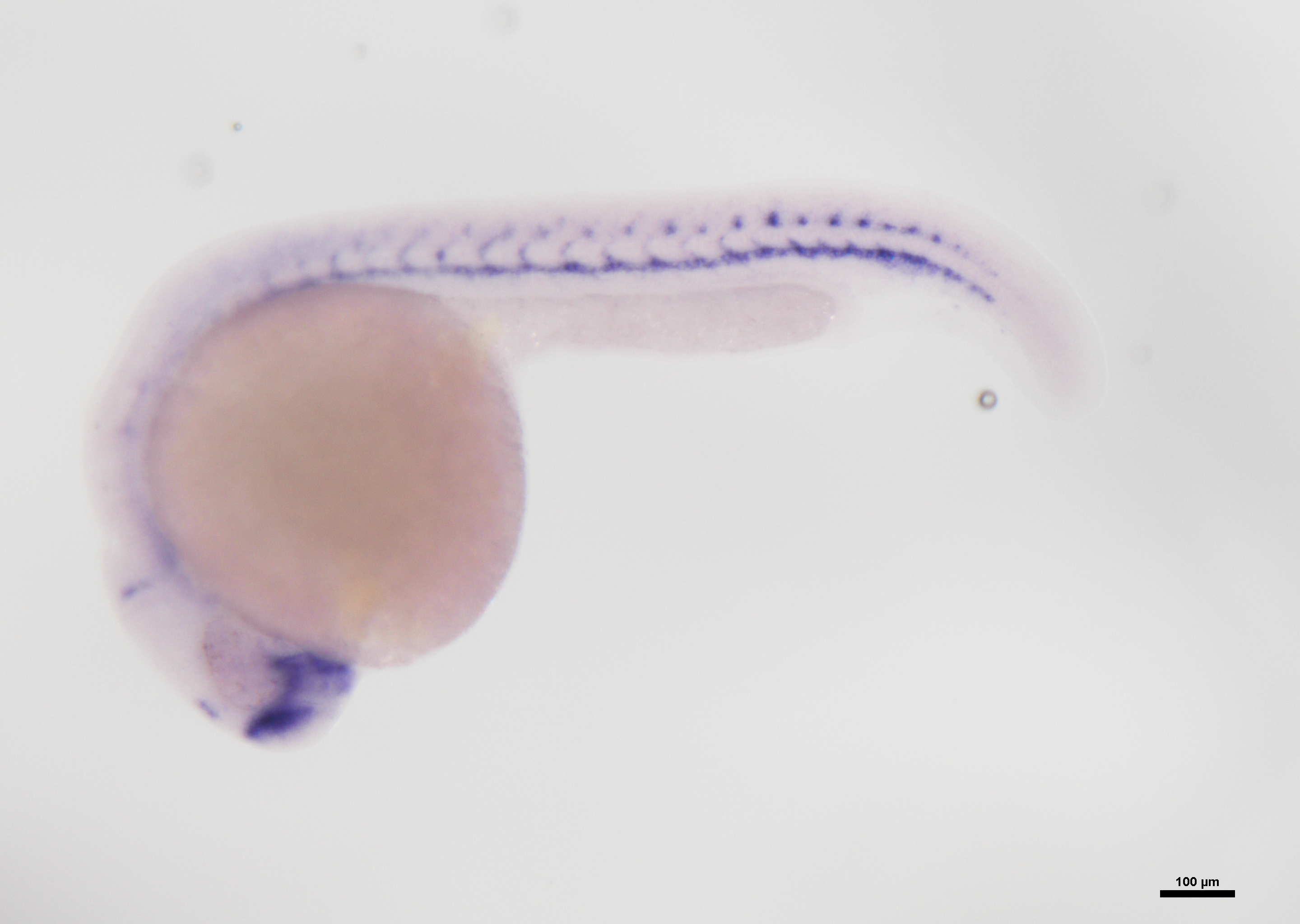

Supplement: Supplementary file 11 — Appendix Figure1-2 Source Data [file 44319_2026_805_MOESM11_ESM.zip › Appendix Source Data 1/Appendix Fig.2/I/2. dll4 24hpf controlMO.tif]

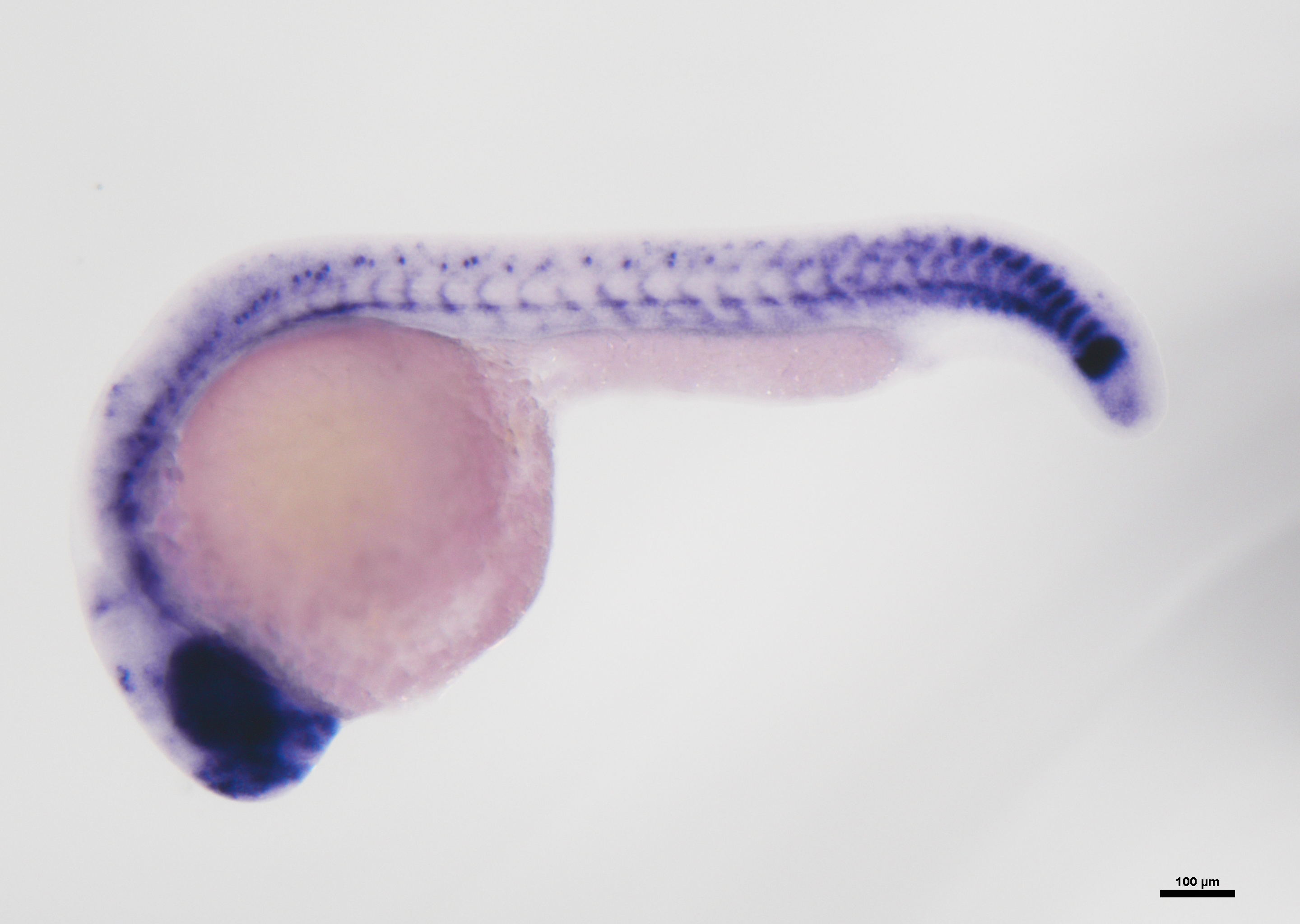

Supplement: Supplementary file 11 — Appendix Figure1-2 Source Data [file 44319_2026_805_MOESM11_ESM.zip › Appendix Source Data 1/Appendix Fig.2/I/3. dltc 24hpf trmt61aMO.tif]

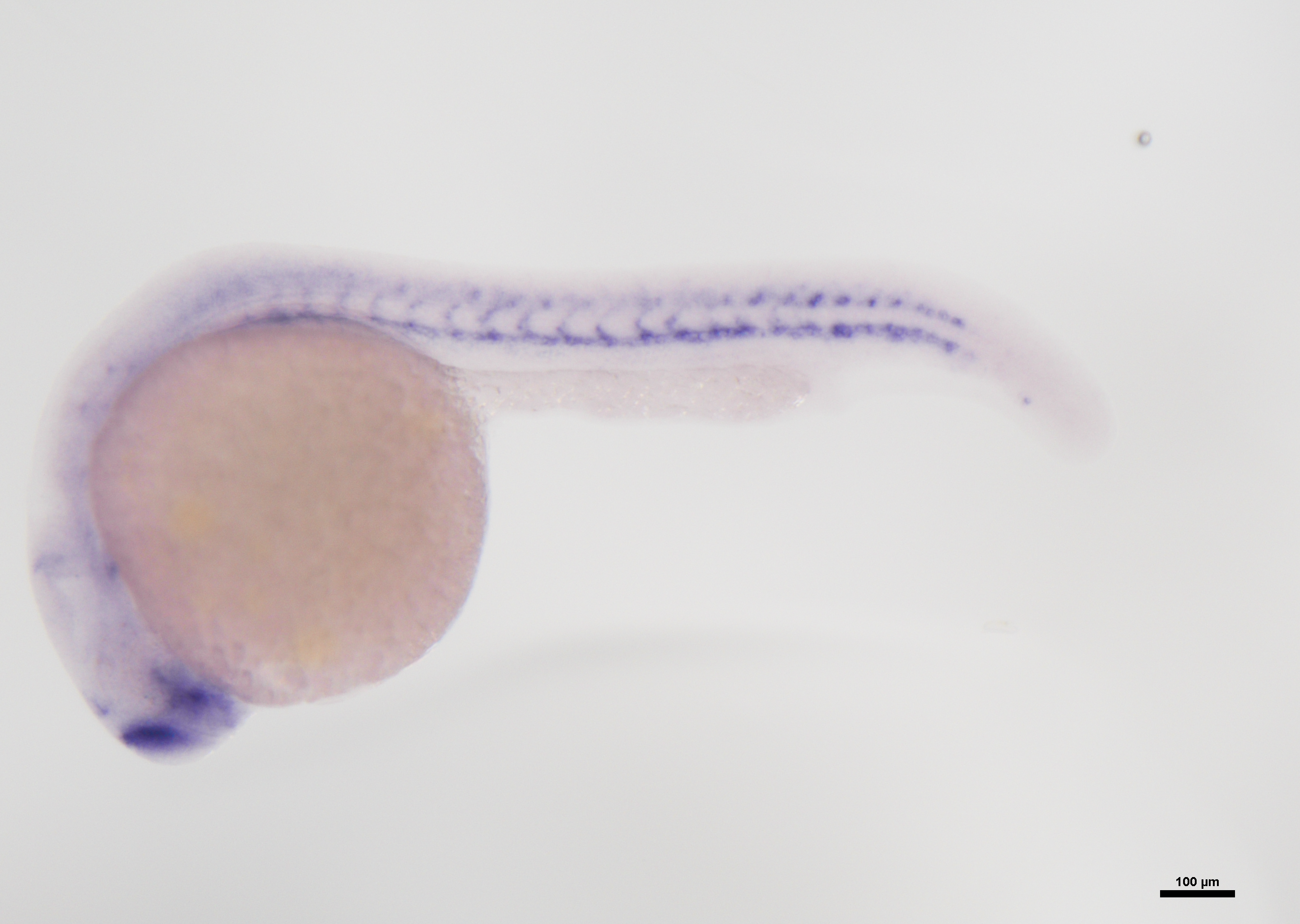

Supplement: Supplementary file 11 — Appendix Figure1-2 Source Data [file 44319_2026_805_MOESM11_ESM.zip › Appendix Source Data 1/Appendix Fig.2/I/4. dll4 24hpf trmt61aMO.tif]

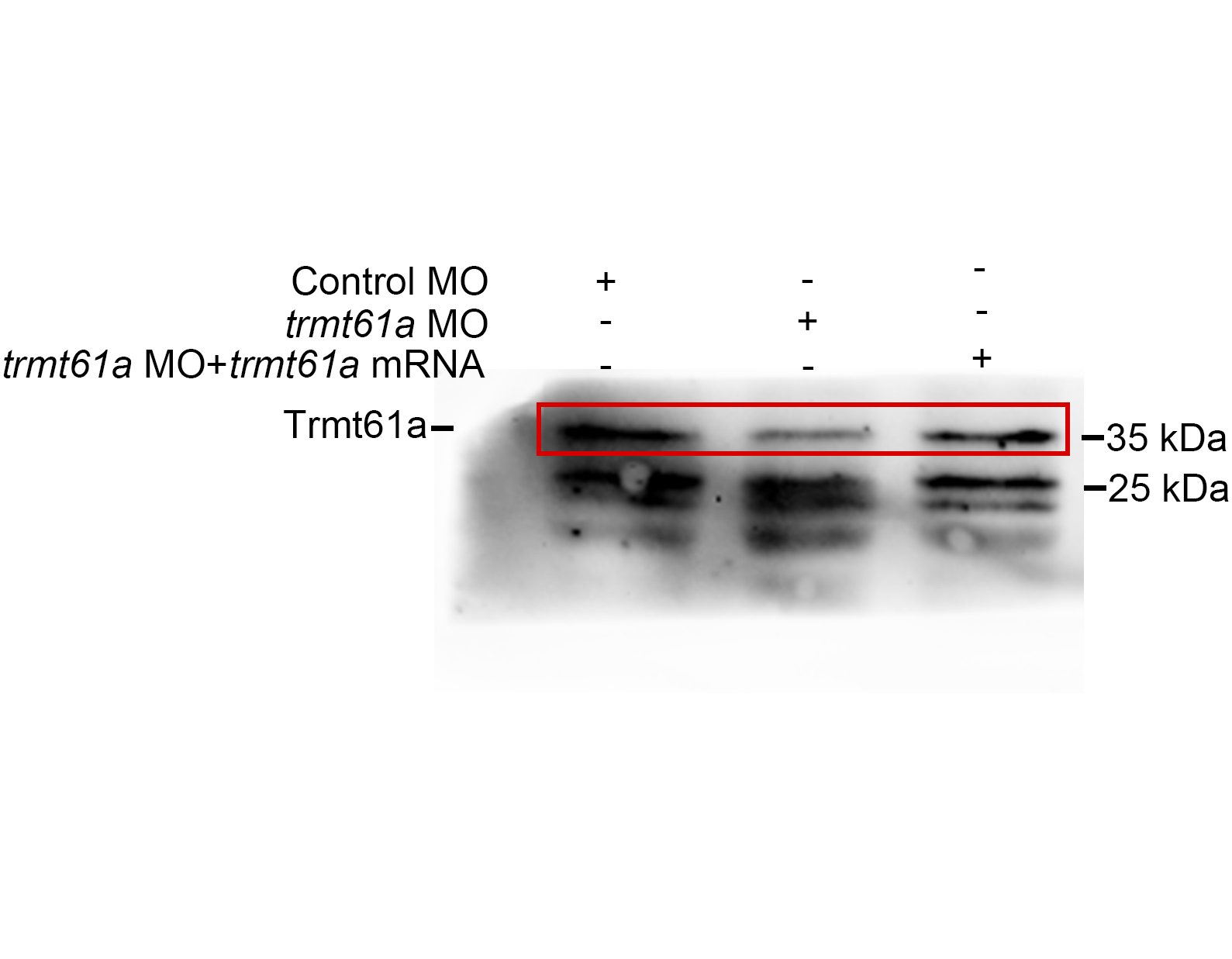

Supplement: Supplementary file 11 — Appendix Figure1-2 Source Data [file 44319_2026_805_MOESM11_ESM.zip › Appendix Source Data 1/Appendix Fig.2/J/2J_Trmt61a WB.tif]

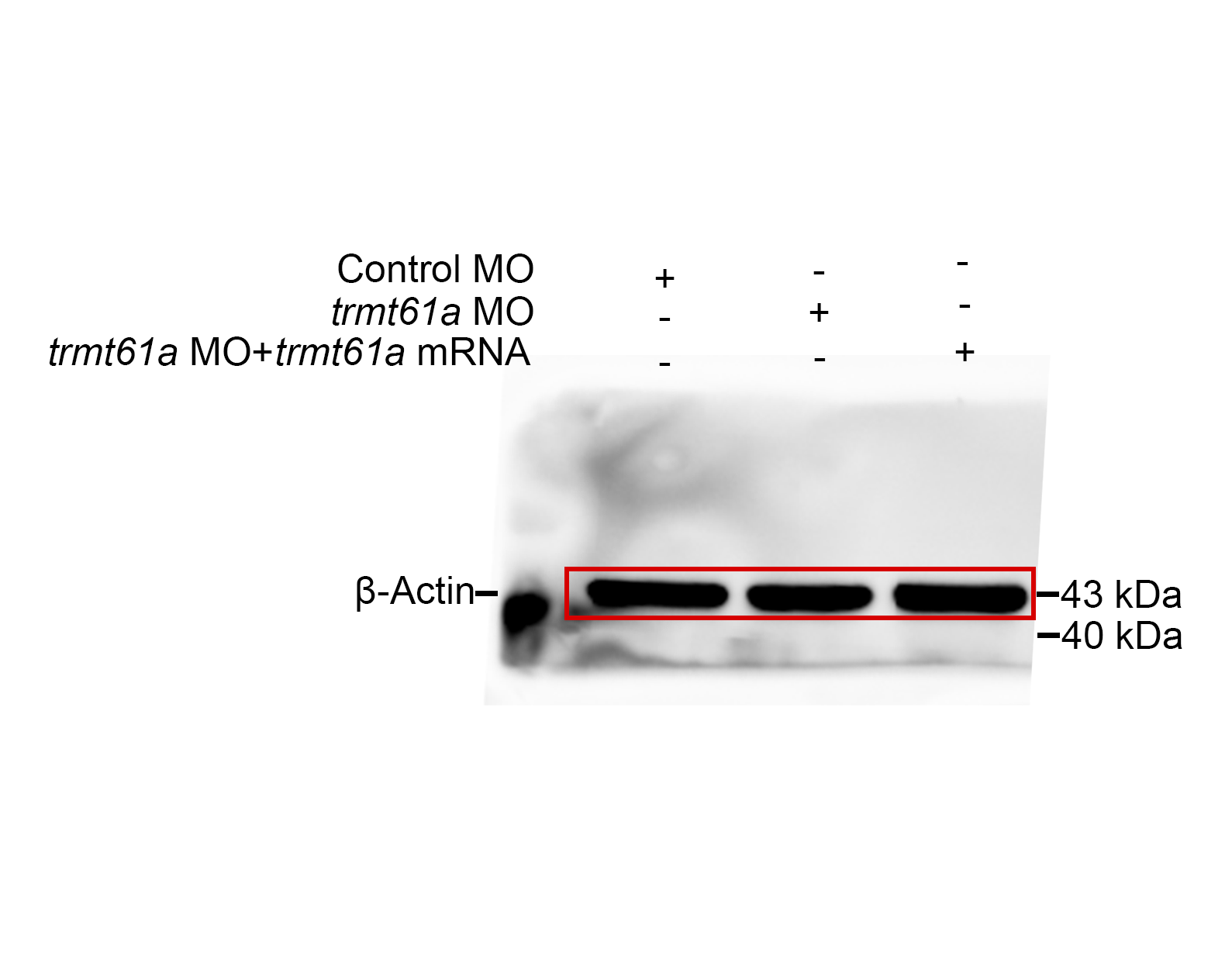

Supplement: Supplementary file 11 — Appendix Figure1-2 Source Data [file 44319_2026_805_MOESM11_ESM.zip › Appendix Source Data 1/Appendix Fig.2/J/2J_β-Actin WB.tif]

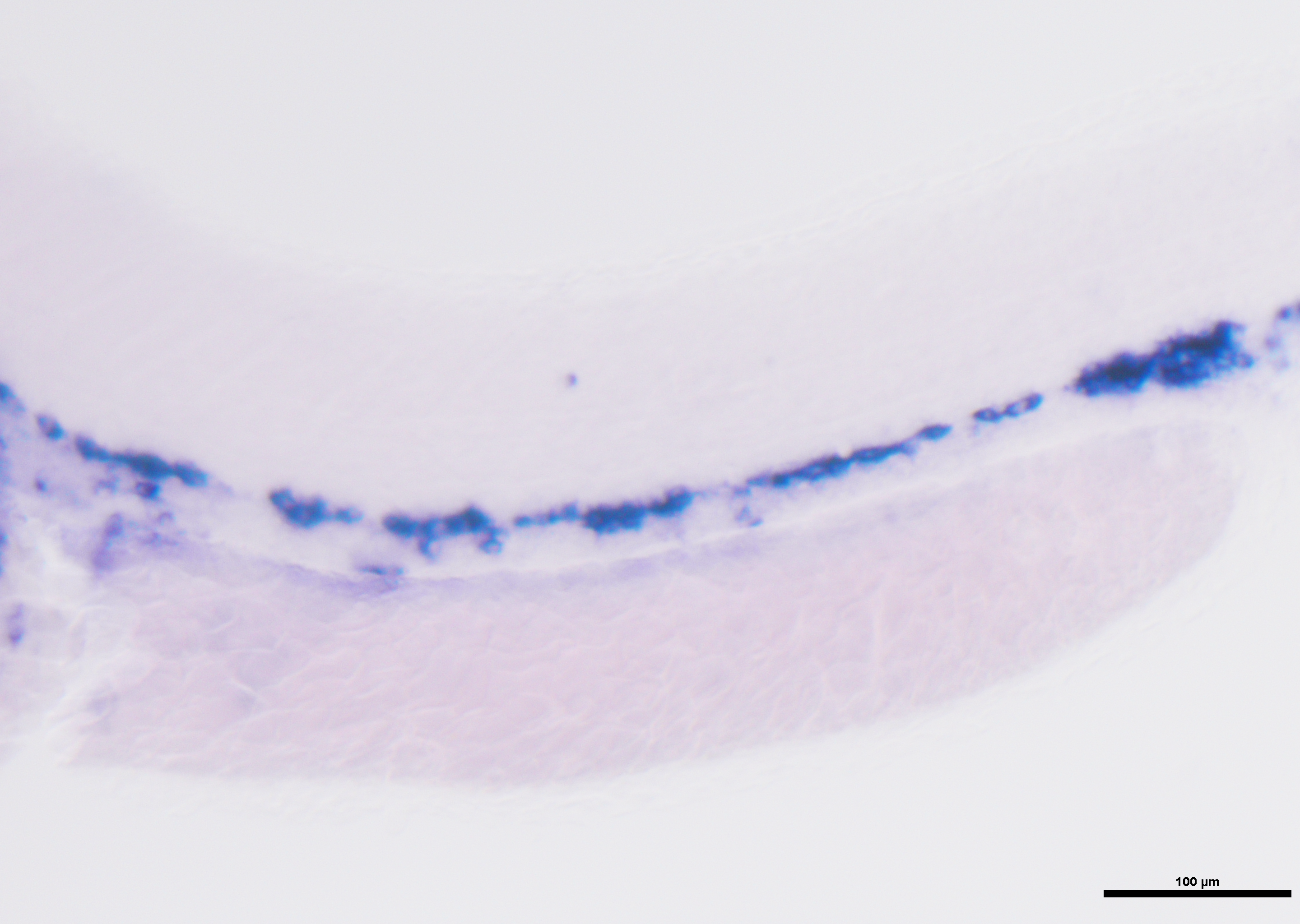

Supplement: Supplementary file 11 — Appendix Figure1-2 Source Data [file 44319_2026_805_MOESM11_ESM.zip › Appendix Source Data 1/Appendix Fig.2/K/1. cmyb 36hpf controlMO.tif]

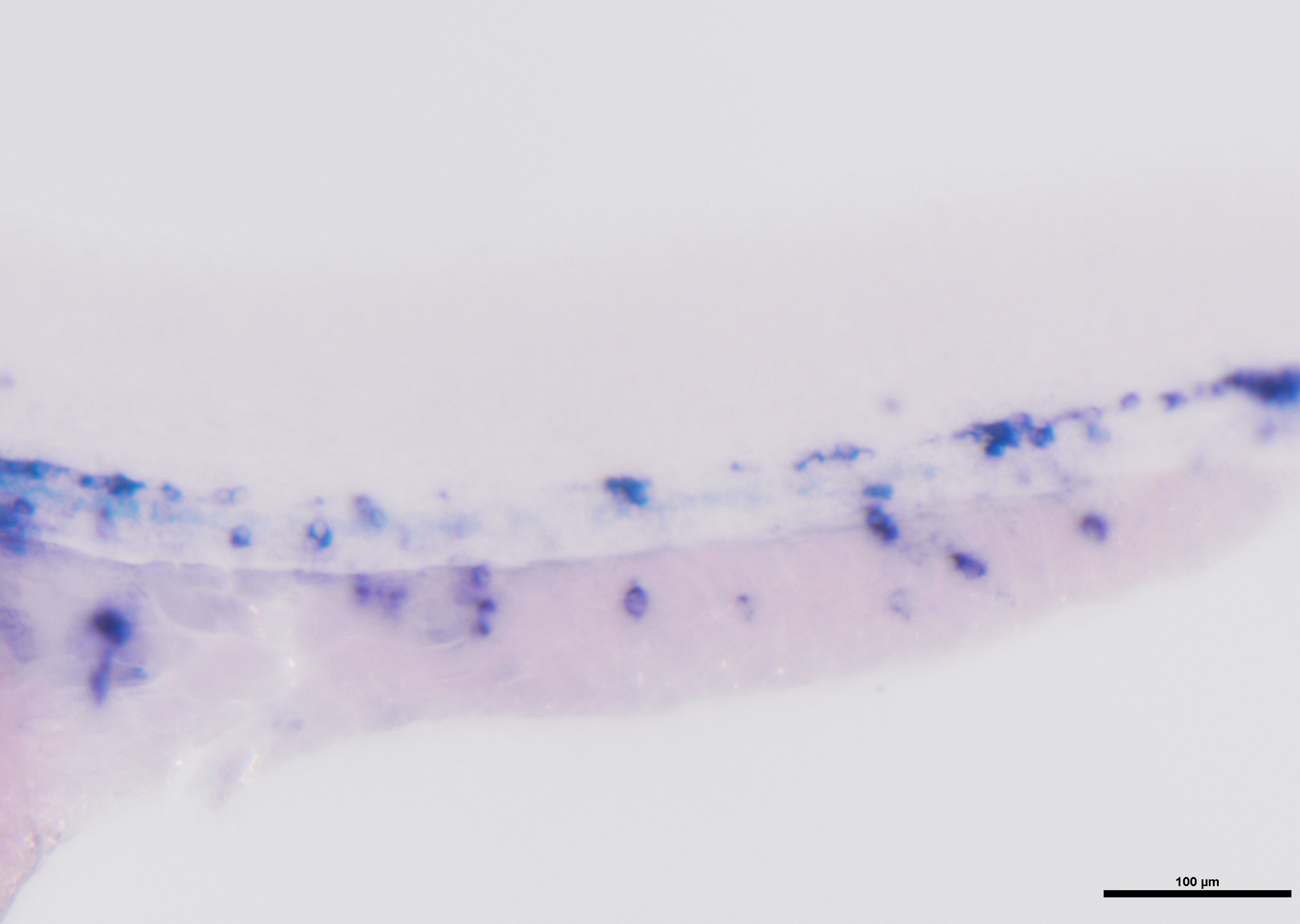

Supplement: Supplementary file 11 — Appendix Figure1-2 Source Data [file 44319_2026_805_MOESM11_ESM.zip › Appendix Source Data 1/Appendix Fig.2/K/2. cmyb 36hpf trmt61aMO.tif]

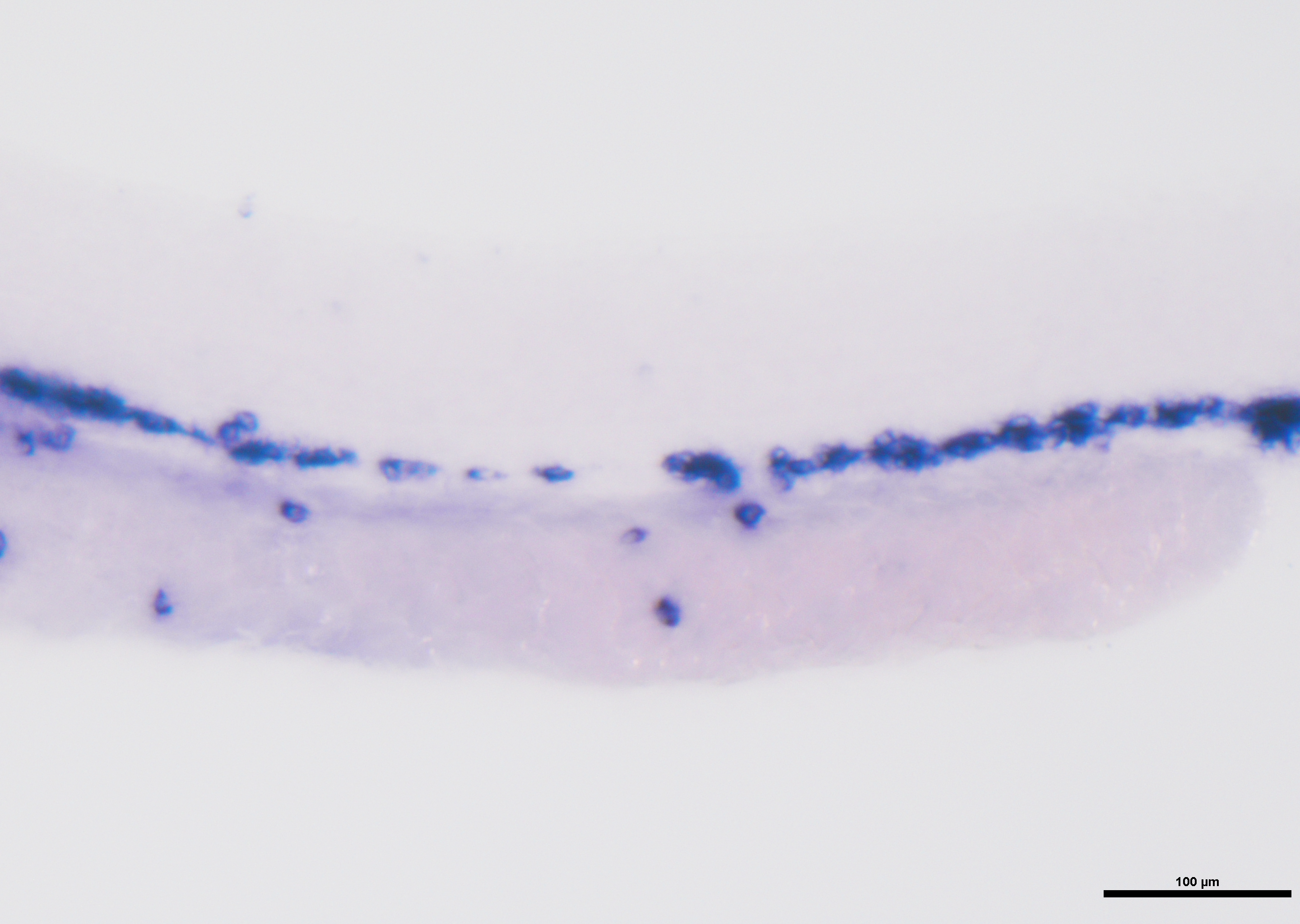

Supplement: Supplementary file 11 — Appendix Figure1-2 Source Data [file 44319_2026_805_MOESM11_ESM.zip › Appendix Source Data 1/Appendix Fig.2/K/3. cmyb 36hpf trmt61aMO+trmt61amRNA.tif]

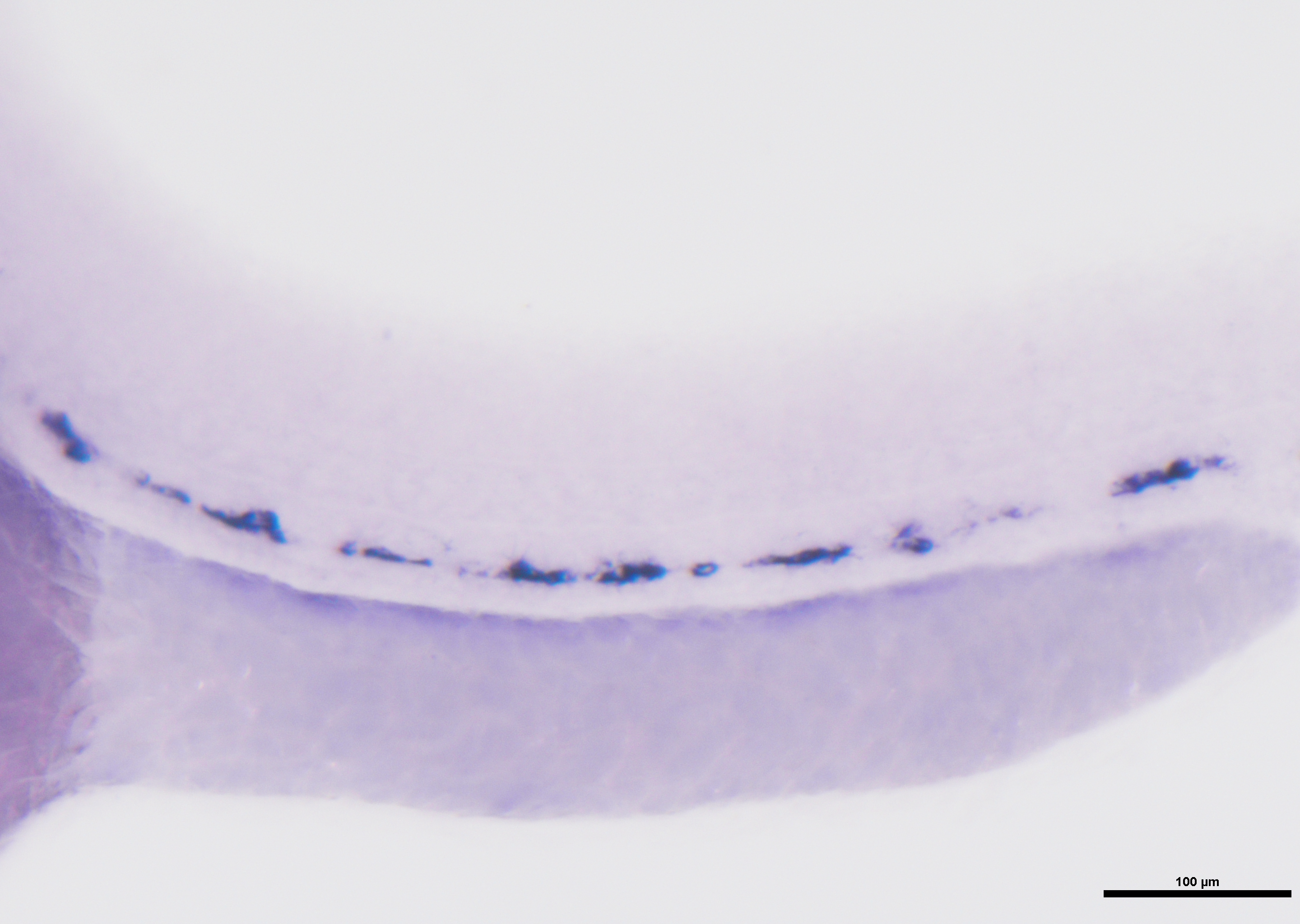

Supplement: Supplementary file 11 — Appendix Figure1-2 Source Data [file 44319_2026_805_MOESM11_ESM.zip › Appendix Source Data 1/Appendix Fig.2/K/4. runx1 36hpf controlMO.tif]

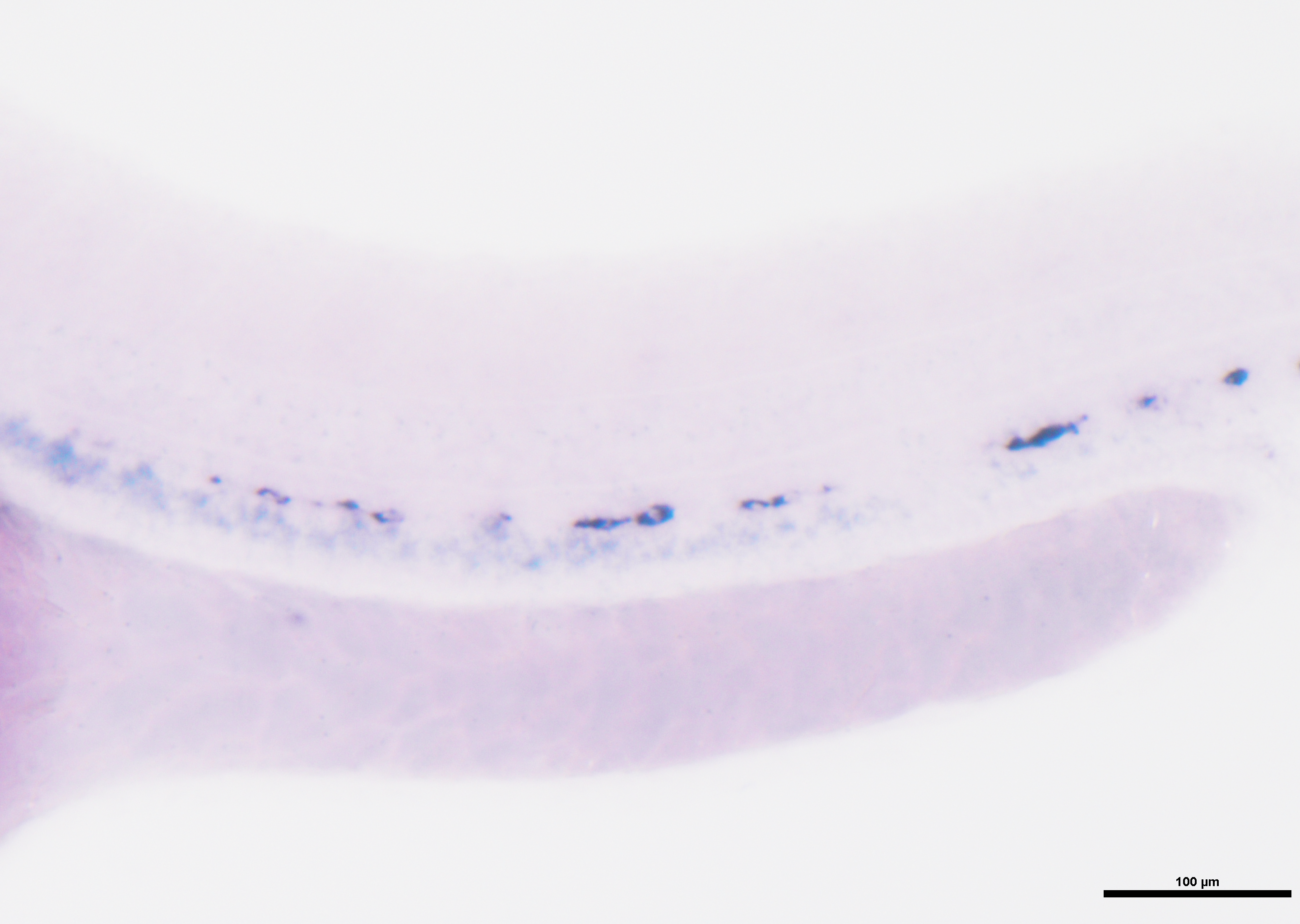

Supplement: Supplementary file 11 — Appendix Figure1-2 Source Data [file 44319_2026_805_MOESM11_ESM.zip › Appendix Source Data 1/Appendix Fig.2/K/5. runx1 36hpf trmt61aMO.tif]

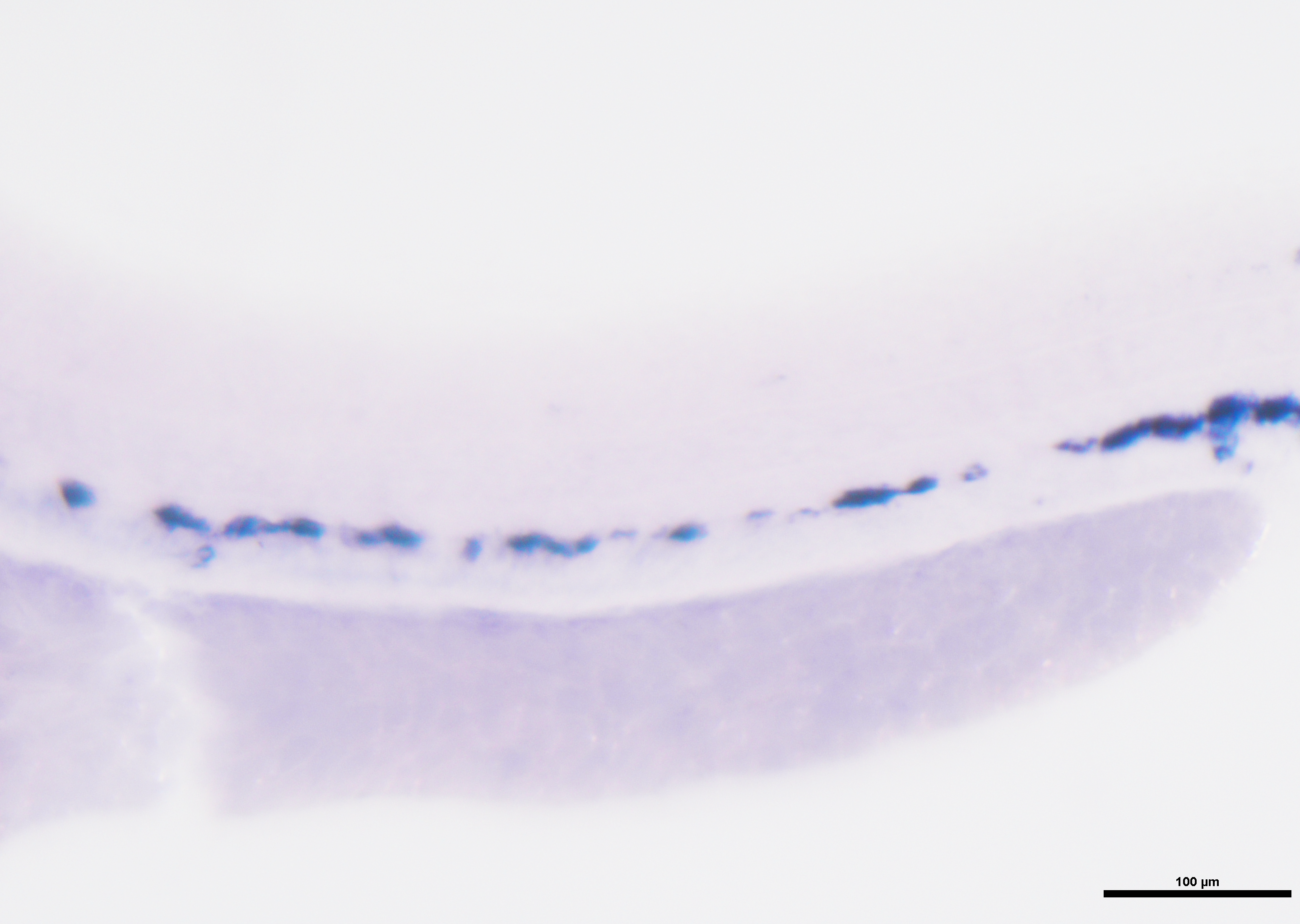

Supplement: Supplementary file 11 — Appendix Figure1-2 Source Data [file 44319_2026_805_MOESM11_ESM.zip › Appendix Source Data 1/Appendix Fig.2/K/6. runx1 36hpf trmt61aMO+trmt61amRNA.tif]

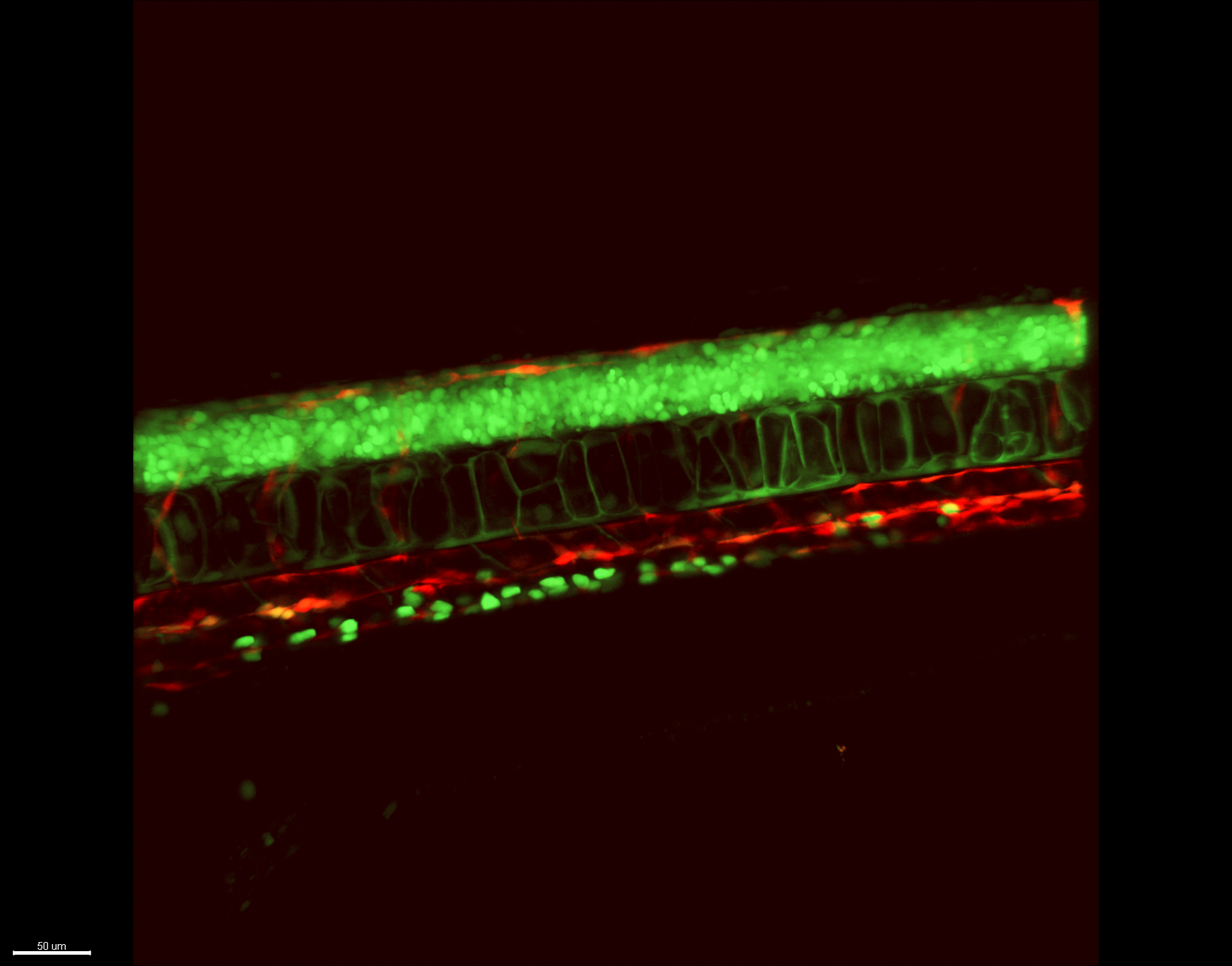

Supplement: Supplementary file 11 — Appendix Figure1-2 Source Data [file 44319_2026_805_MOESM11_ESM.zip › Appendix Source Data 1/Appendix Fig.2/L/1. 36hpf controlMO.tif]

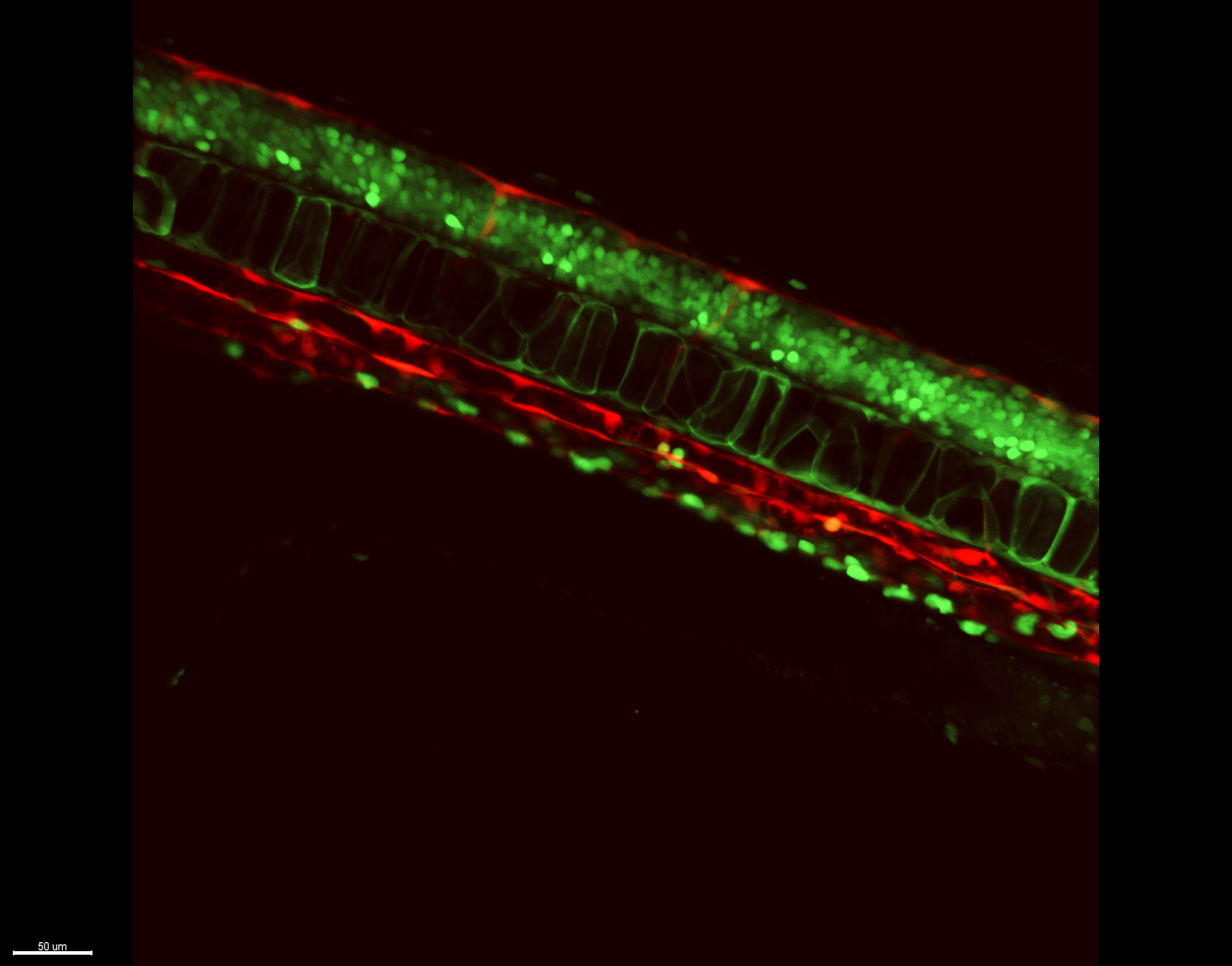

Supplement: Supplementary file 11 — Appendix Figure1-2 Source Data [file 44319_2026_805_MOESM11_ESM.zip › Appendix Source Data 1/Appendix Fig.2/L/2. 36hpf trmt61aMO.tif]

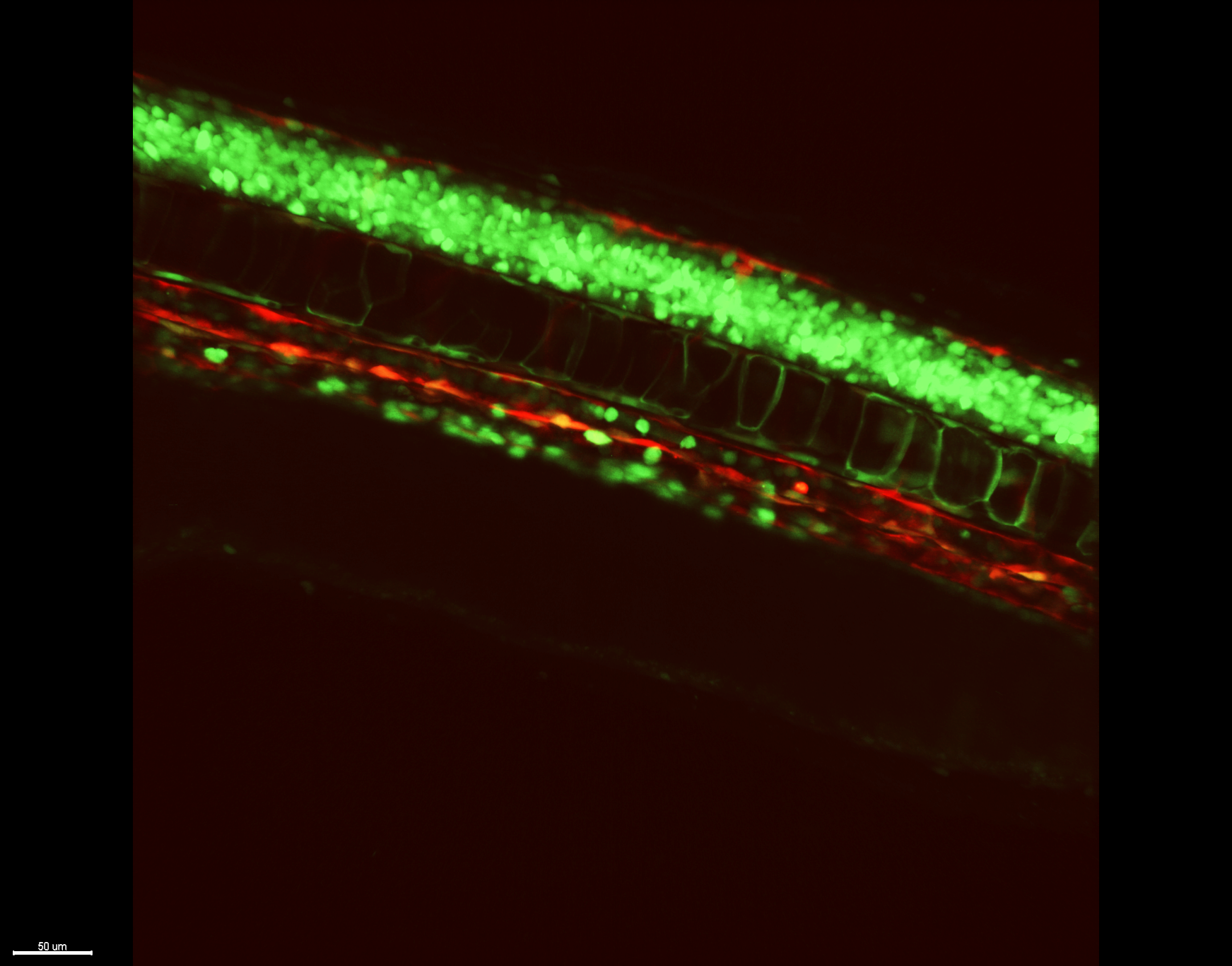

Supplement: Supplementary file 11 — Appendix Figure1-2 Source Data [file 44319_2026_805_MOESM11_ESM.zip › Appendix Source Data 1/Appendix Fig.2/L/3. 36hpf trmt61aMO+trmt61amRNA.tif]

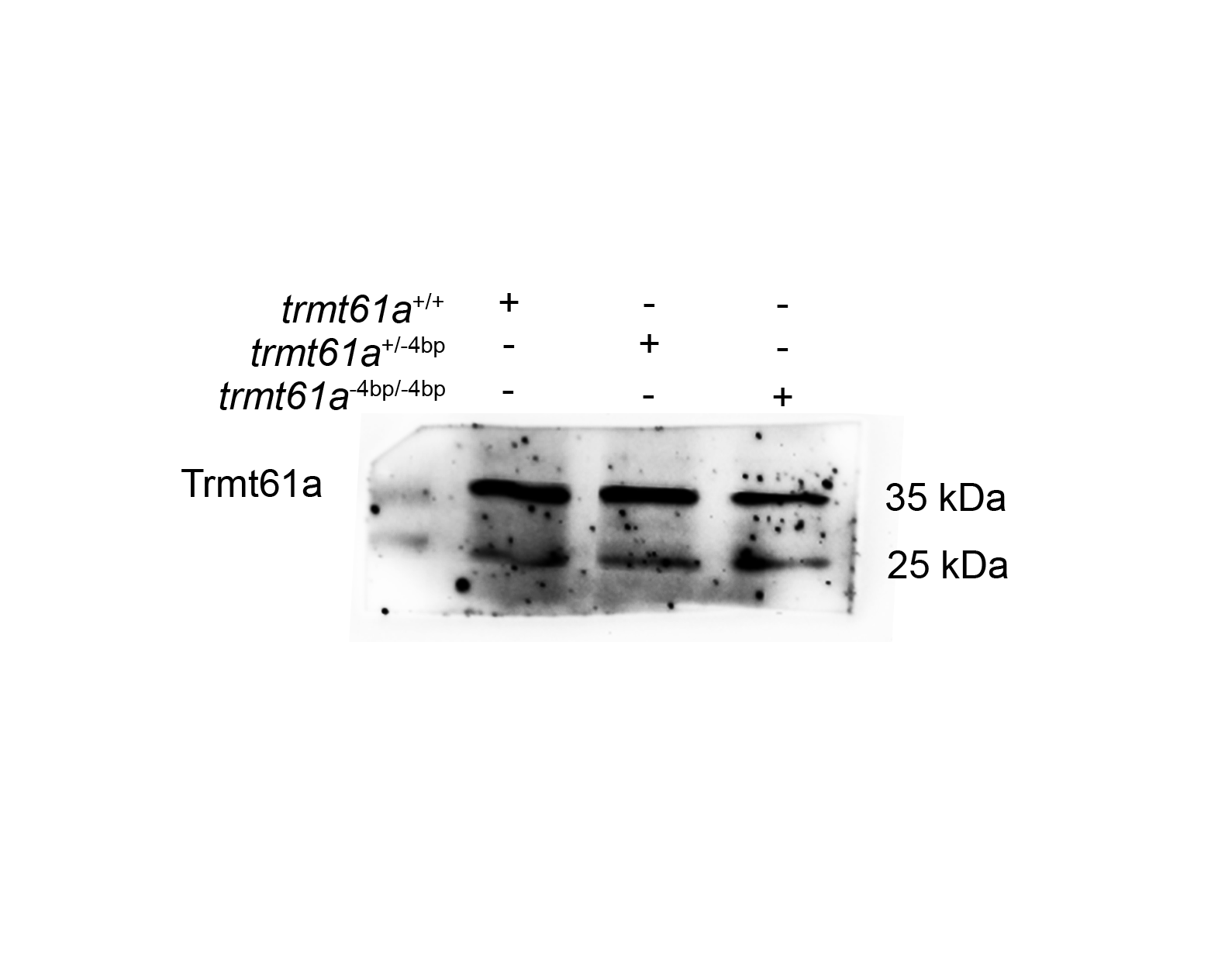

Supplement: Supplementary file 12 — Appendix Figure 3-4 Source Data [file 44319_2026_805_MOESM12_ESM.zip › Appendix Source Data 2/Appendix Fig.3/B/1.Trmt61a_36hpf WB.tif]

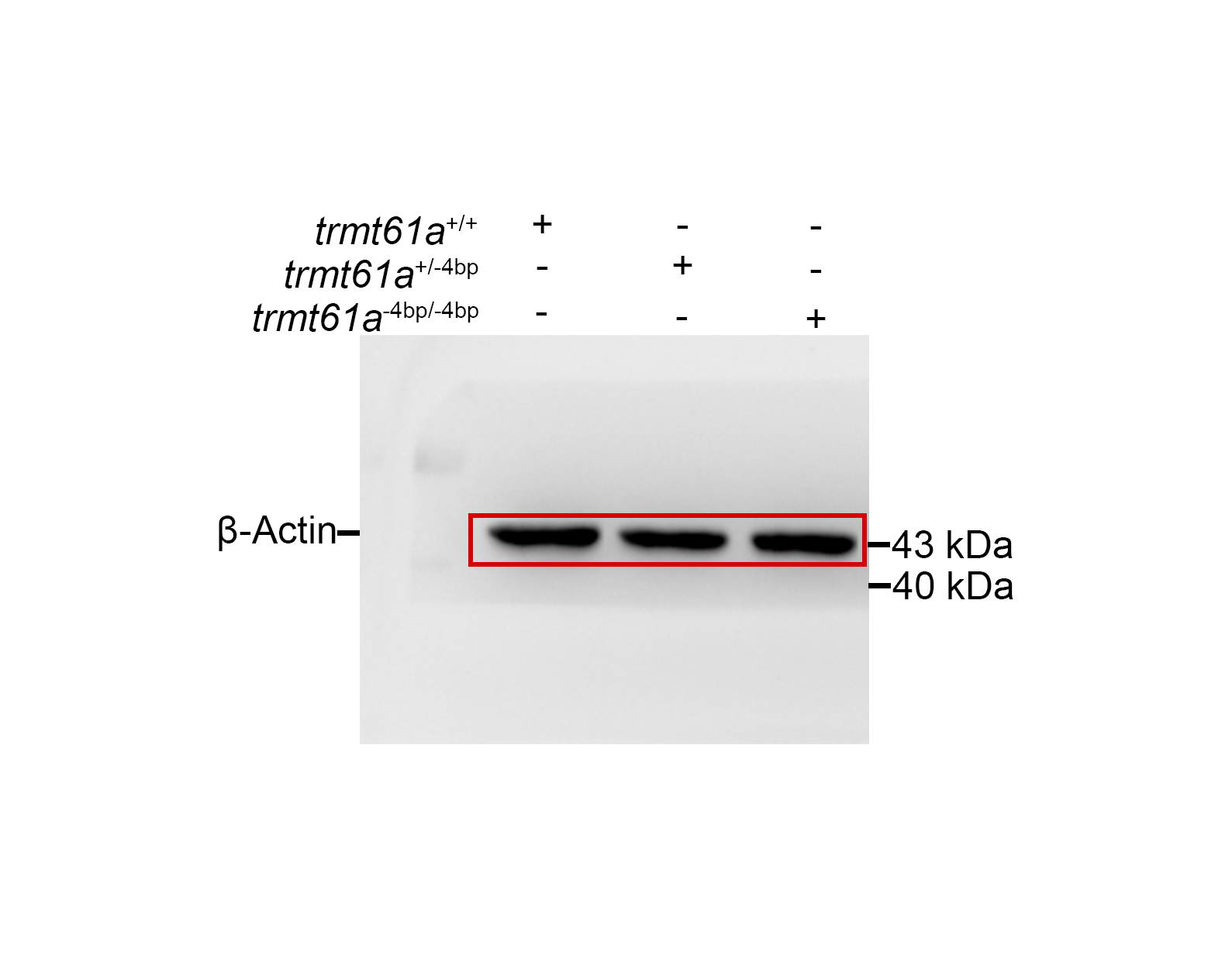

Supplement: Supplementary file 12 — Appendix Figure 3-4 Source Data [file 44319_2026_805_MOESM12_ESM.zip › Appendix Source Data 2/Appendix Fig.3/B/2. β-Actin_36hpf WB.tif]

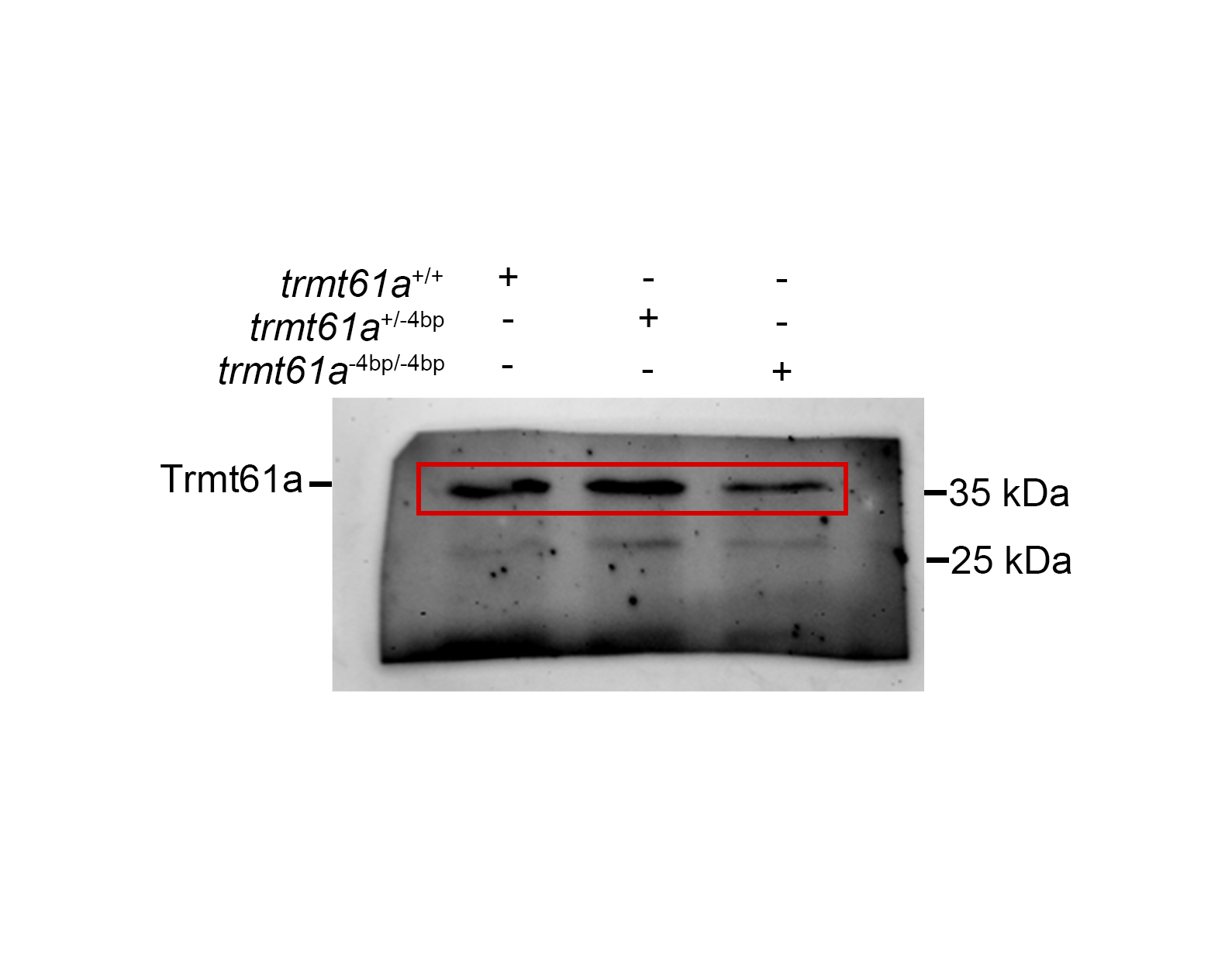

Supplement: Supplementary file 12 — Appendix Figure 3-4 Source Data [file 44319_2026_805_MOESM12_ESM.zip › Appendix Source Data 2/Appendix Fig.3/B/3.Trmt61a_2dpf WB.tif]

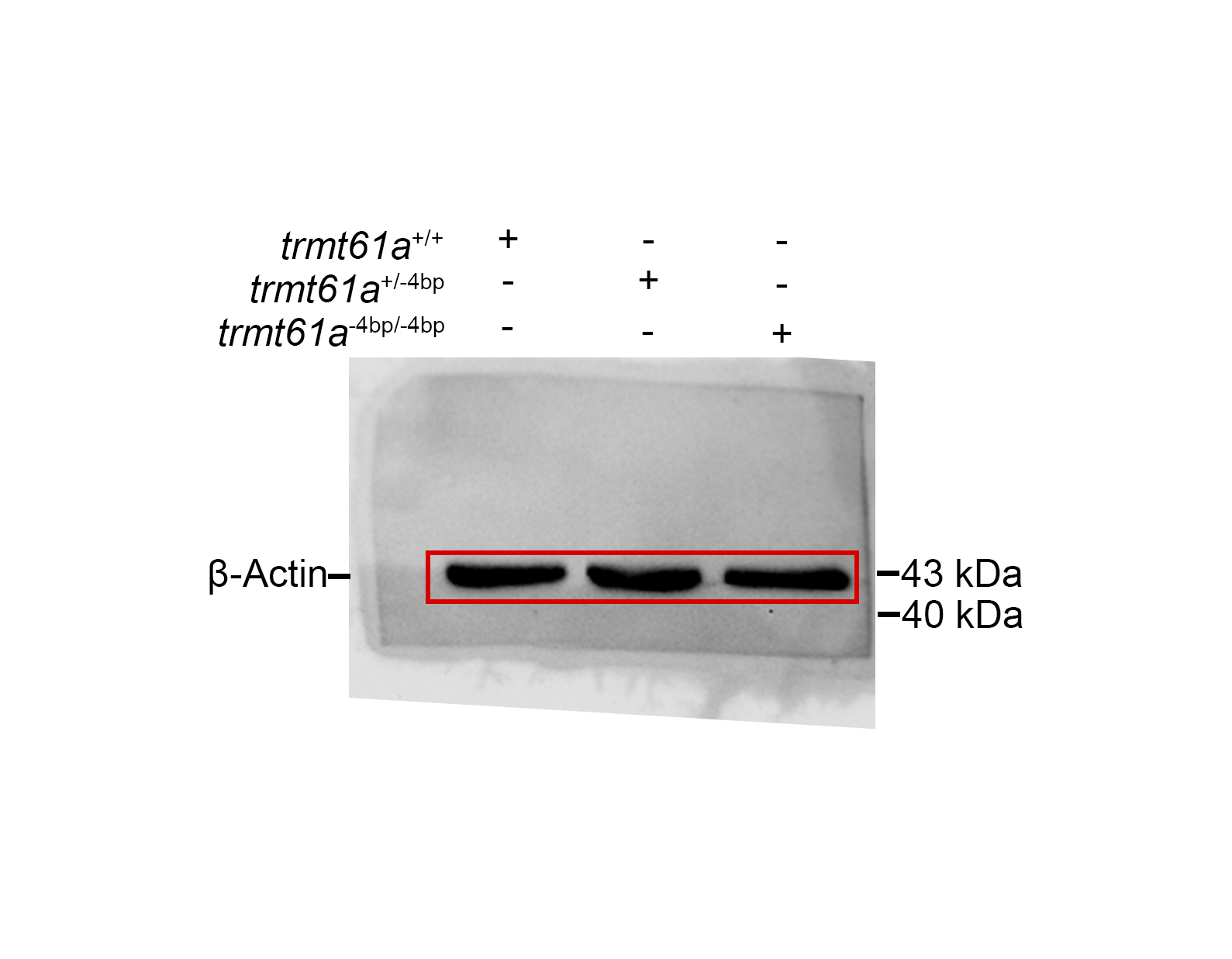

Supplement: Supplementary file 12 — Appendix Figure 3-4 Source Data [file 44319_2026_805_MOESM12_ESM.zip › Appendix Source Data 2/Appendix Fig.3/B/4. β-Actin_2dpf WB.tif]

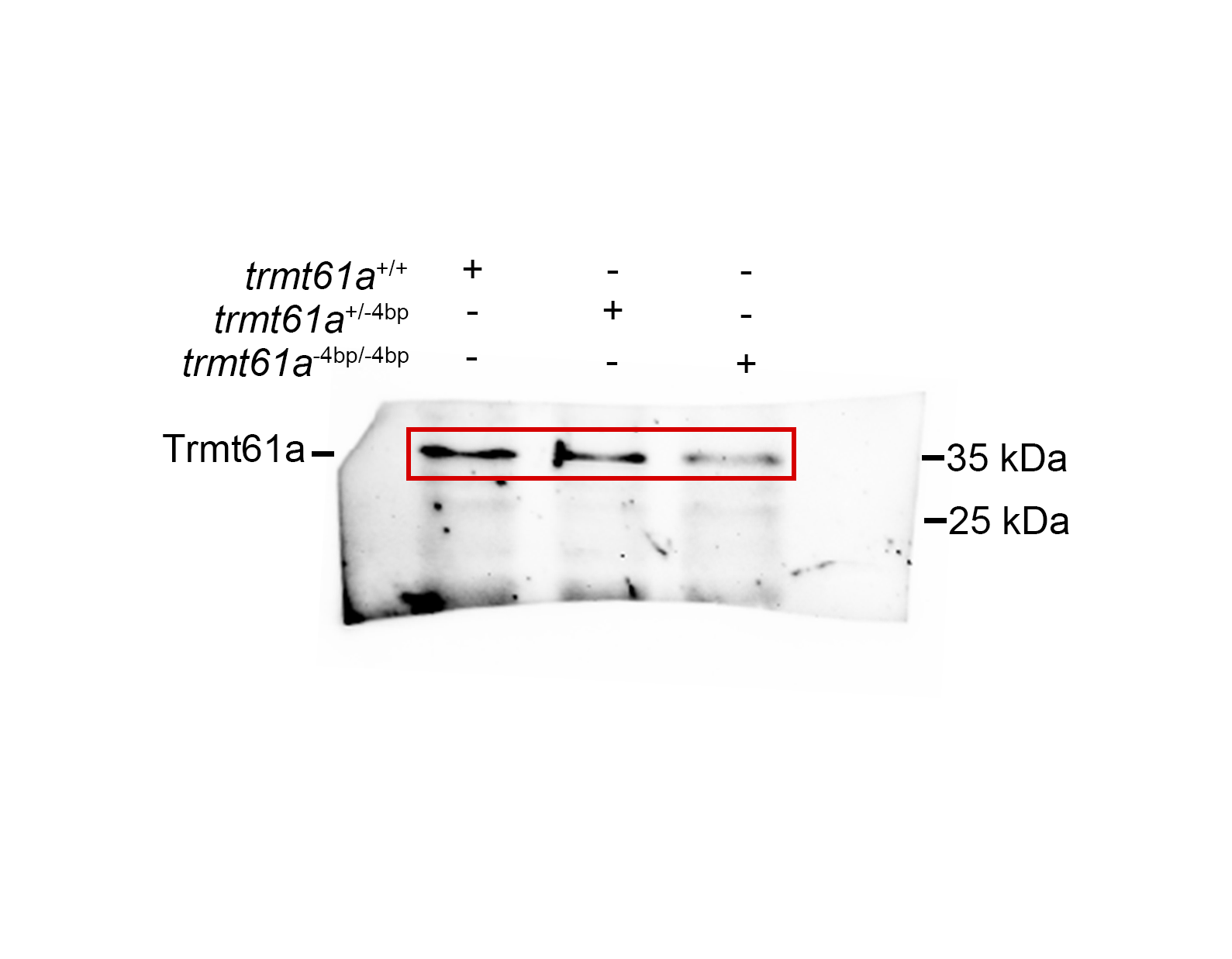

Supplement: Supplementary file 12 — Appendix Figure 3-4 Source Data [file 44319_2026_805_MOESM12_ESM.zip › Appendix Source Data 2/Appendix Fig.3/B/5. Trmt61a_4dpf WB.tif]

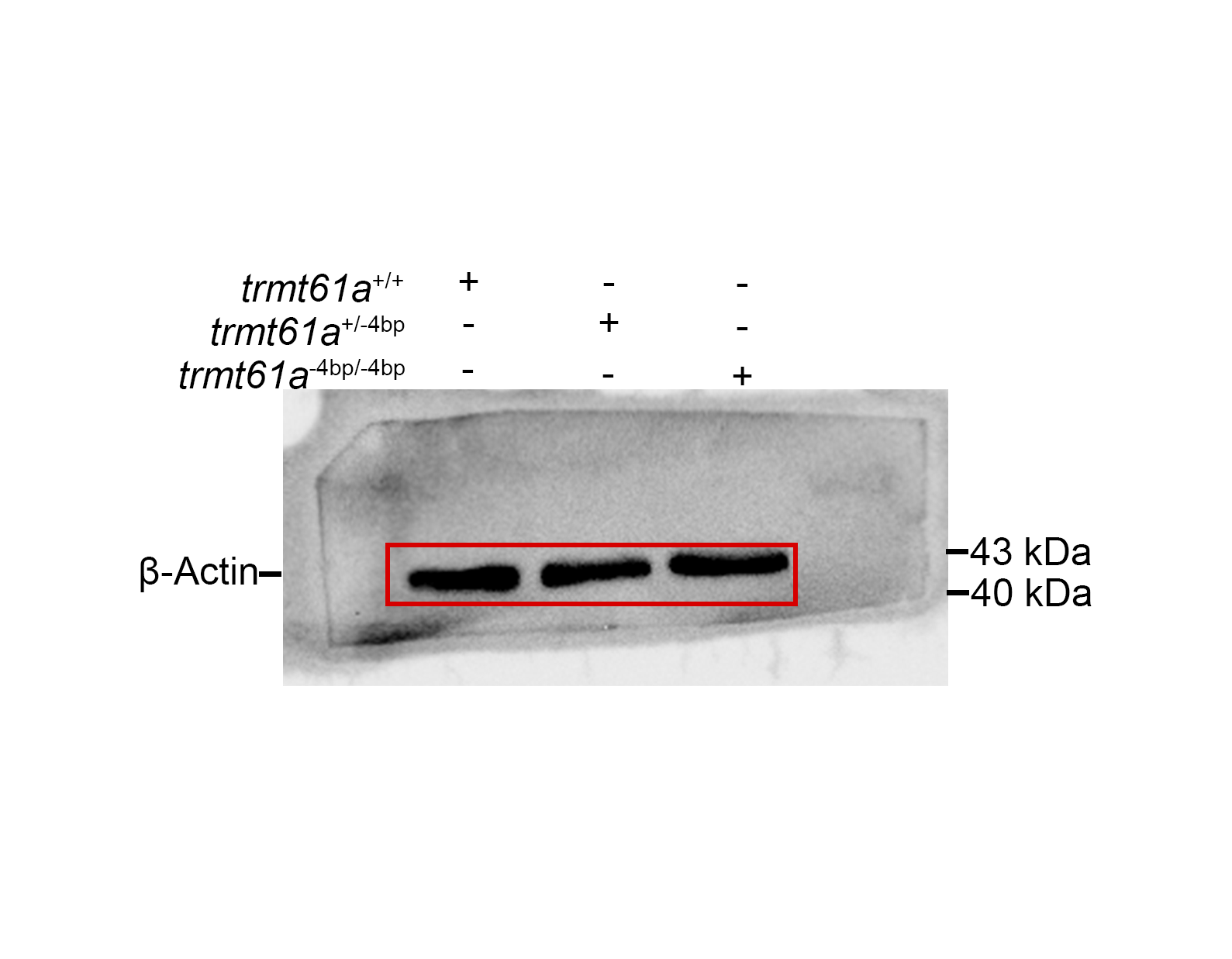

Supplement: Supplementary file 12 — Appendix Figure 3-4 Source Data [file 44319_2026_805_MOESM12_ESM.zip › Appendix Source Data 2/Appendix Fig.3/B/6. β-Actin_4dpf WB.tif]

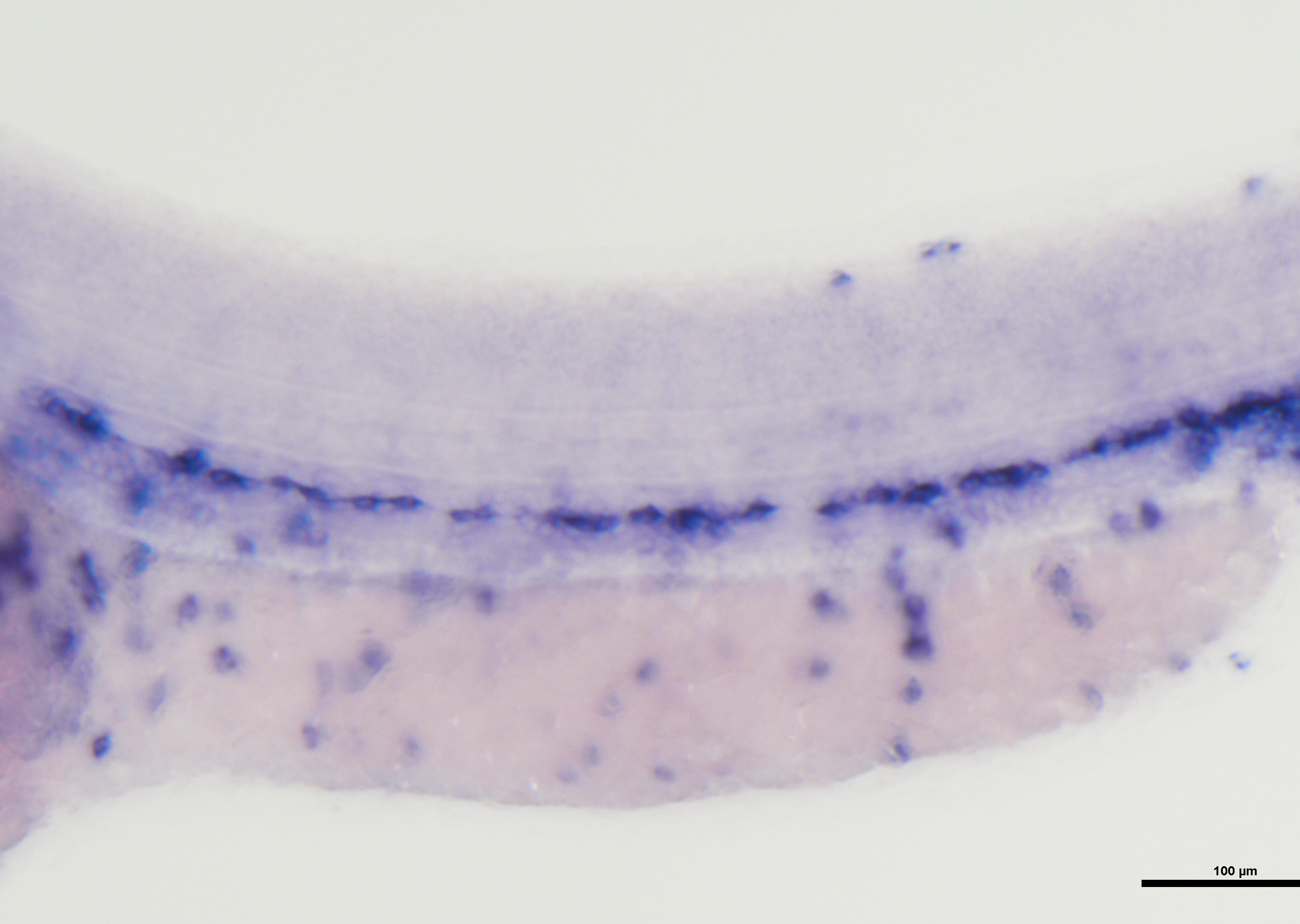

Supplement: Supplementary file 12 — Appendix Figure 3-4 Source Data [file 44319_2026_805_MOESM12_ESM.zip › Appendix Source Data 2/Appendix Fig.3/C/1. cmyb 36hpf trmt61a++.tif]

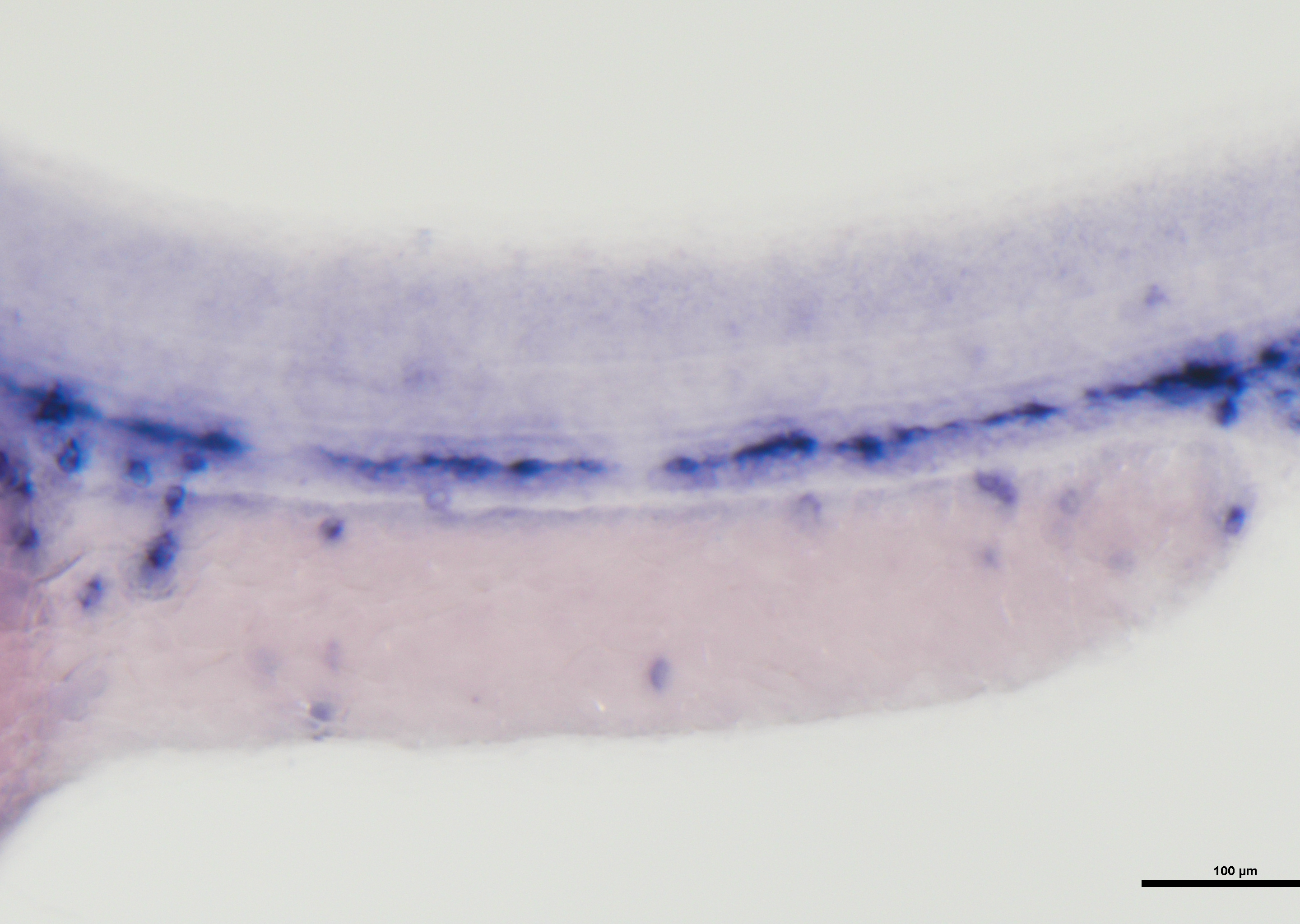

Supplement: Supplementary file 12 — Appendix Figure 3-4 Source Data [file 44319_2026_805_MOESM12_ESM.zip › Appendix Source Data 2/Appendix Fig.3/C/2. cmyb 36hpf trmt61a+-4bp.tif]
