# Supplementary figures and images for: tRNA m1A modification ensures HSPC production via modulating Nrf1 translation in zebrafish (part 3 of 3)
Source: EMBO Rep. 2026 May 27;27(13):3826–41. doi: 10.1038/s44319-026-00805-5 (PMC13354807; doi:10.1038/s44319-026-00805-5)

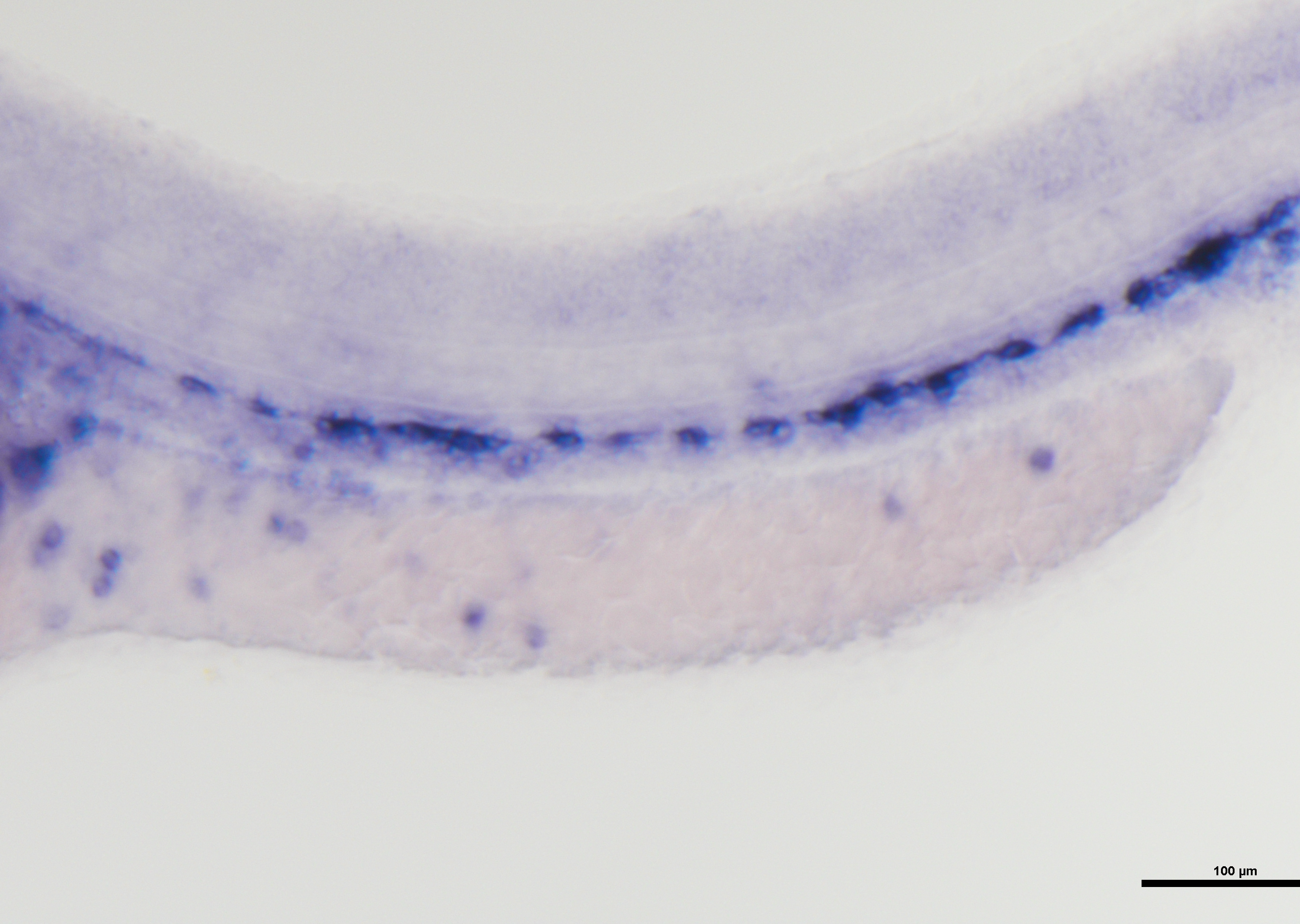

Supplement: Supplementary file 12 — Appendix Figure 3-4 Source Data [file 44319_2026_805_MOESM12_ESM.zip › Appendix Source Data 2/Appendix Fig.3/C/3. cmyb 36hpf trmt61a-4bp-4bp.tif]

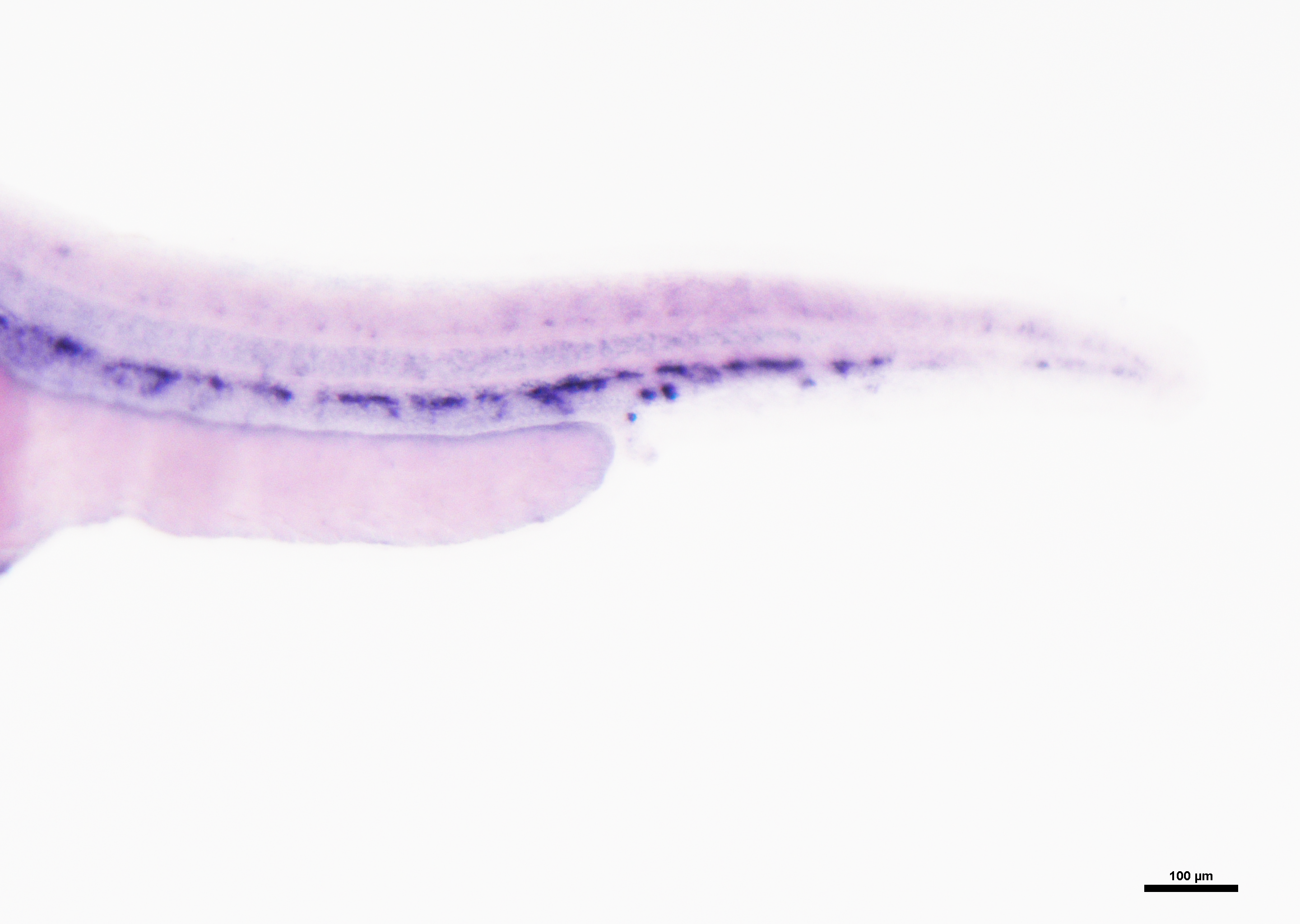

Supplement: Supplementary file 12 — Appendix Figure 3-4 Source Data [file 44319_2026_805_MOESM12_ESM.zip › Appendix Source Data 2/Appendix Fig.3/C/4. runx1 36hpf trmt61a++.tif]

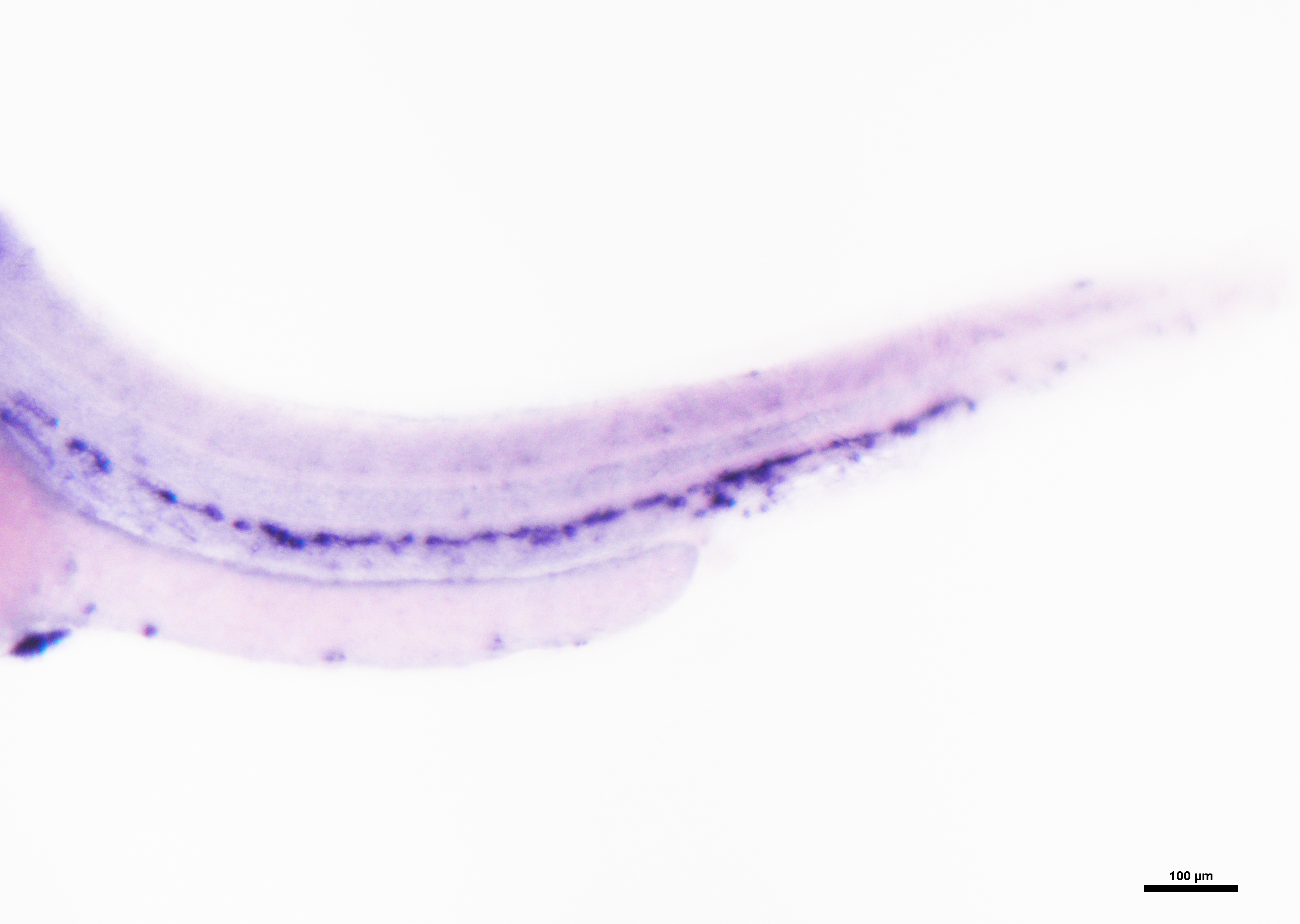

Supplement: Supplementary file 12 — Appendix Figure 3-4 Source Data [file 44319_2026_805_MOESM12_ESM.zip › Appendix Source Data 2/Appendix Fig.3/C/5. runx1 36hpf trmt61a+-4bp.tif]

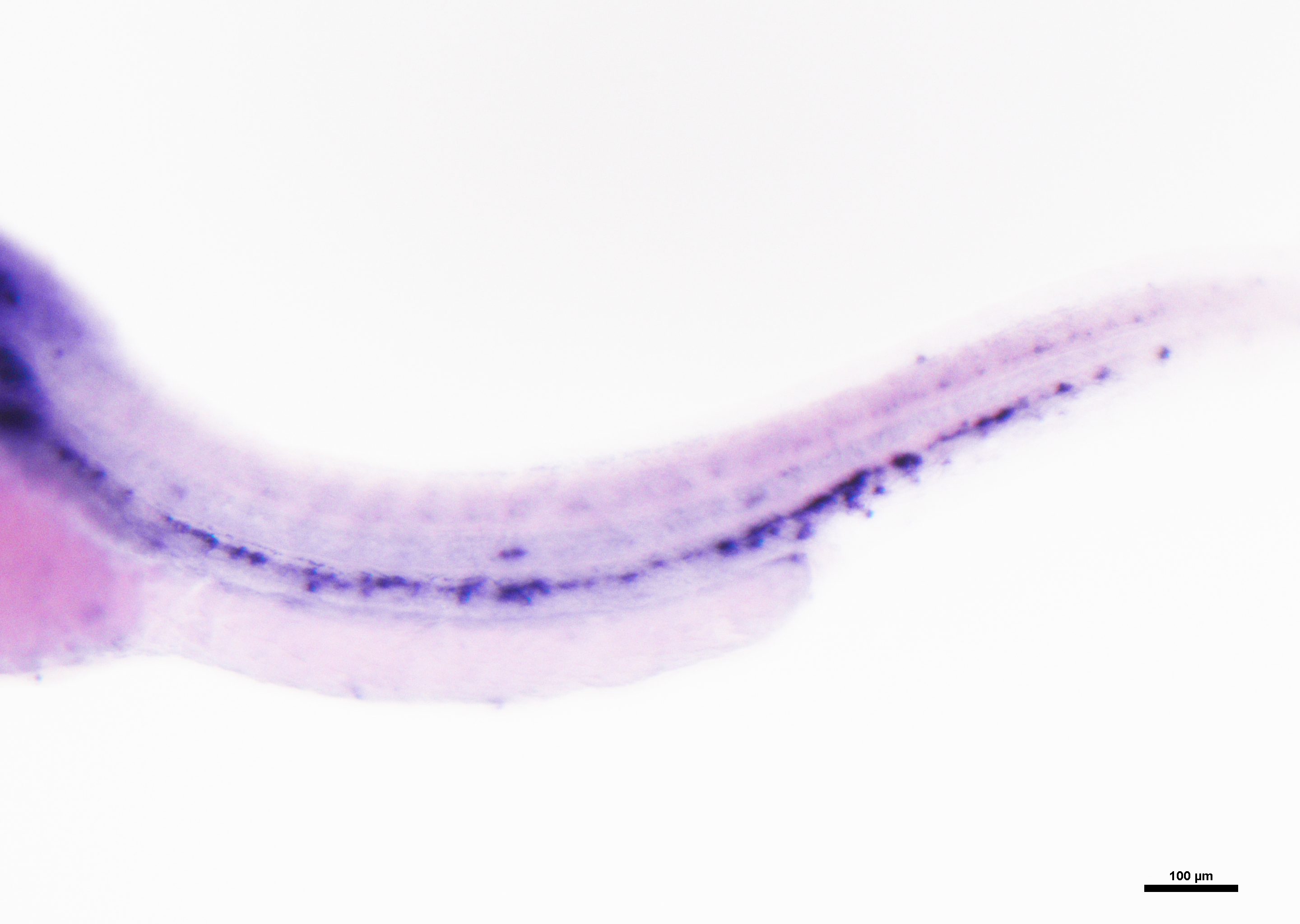

Supplement: Supplementary file 12 — Appendix Figure 3-4 Source Data [file 44319_2026_805_MOESM12_ESM.zip › Appendix Source Data 2/Appendix Fig.3/C/6. runx1 36hpf trmt61a-4bp-4bp.tif]

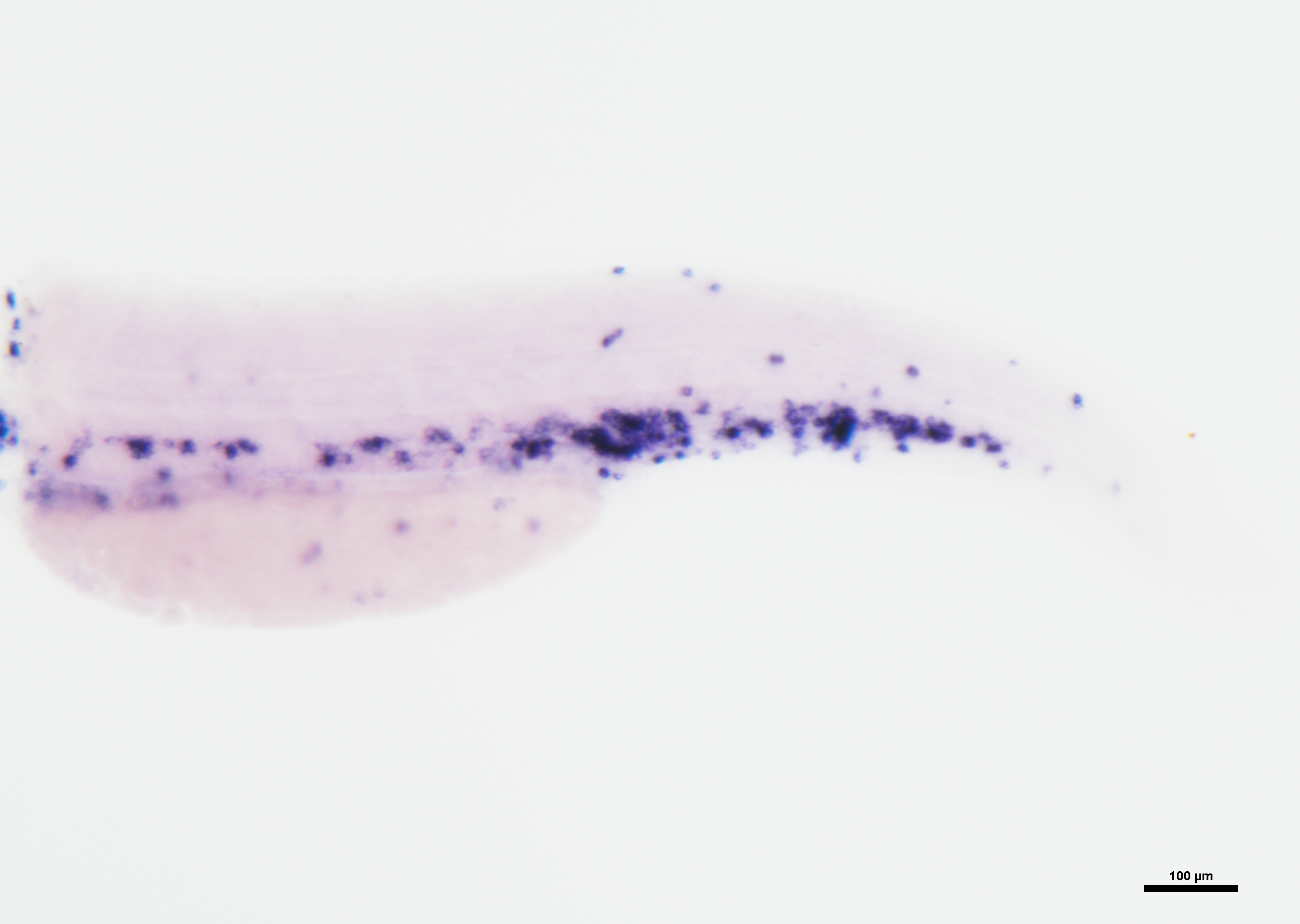

Supplement: Supplementary file 12 — Appendix Figure 3-4 Source Data [file 44319_2026_805_MOESM12_ESM.zip › Appendix Source Data 2/Appendix Fig.3/C/7. cmyb 2dpf trmt61a++.tif]

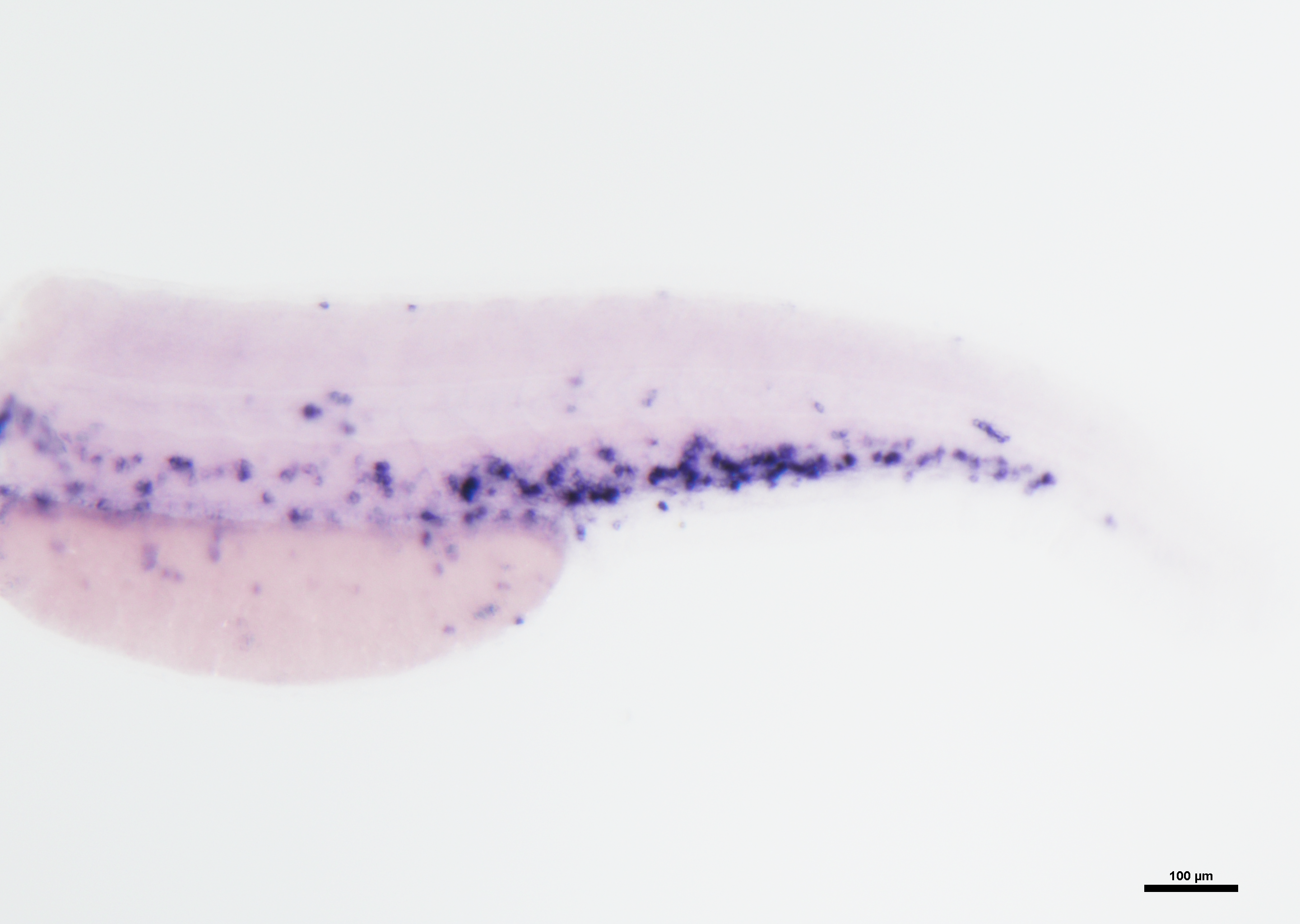

Supplement: Supplementary file 12 — Appendix Figure 3-4 Source Data [file 44319_2026_805_MOESM12_ESM.zip › Appendix Source Data 2/Appendix Fig.3/C/8. cmyb 2dpf trmt61a+-4bp.tif]

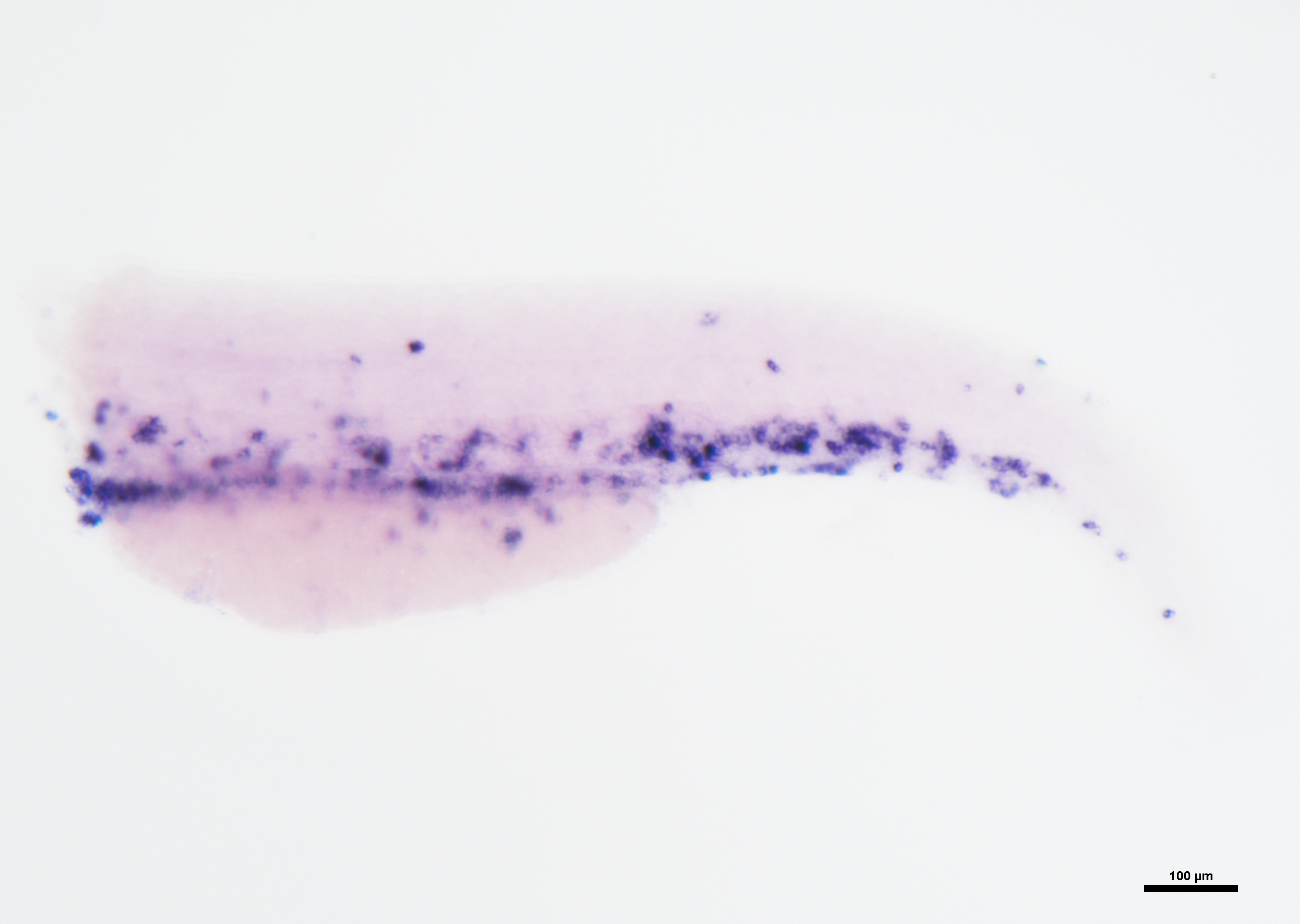

Supplement: Supplementary file 12 — Appendix Figure 3-4 Source Data [file 44319_2026_805_MOESM12_ESM.zip › Appendix Source Data 2/Appendix Fig.3/C/9. cmyb 2dpf trmt61a-4bp-4bp.tif]

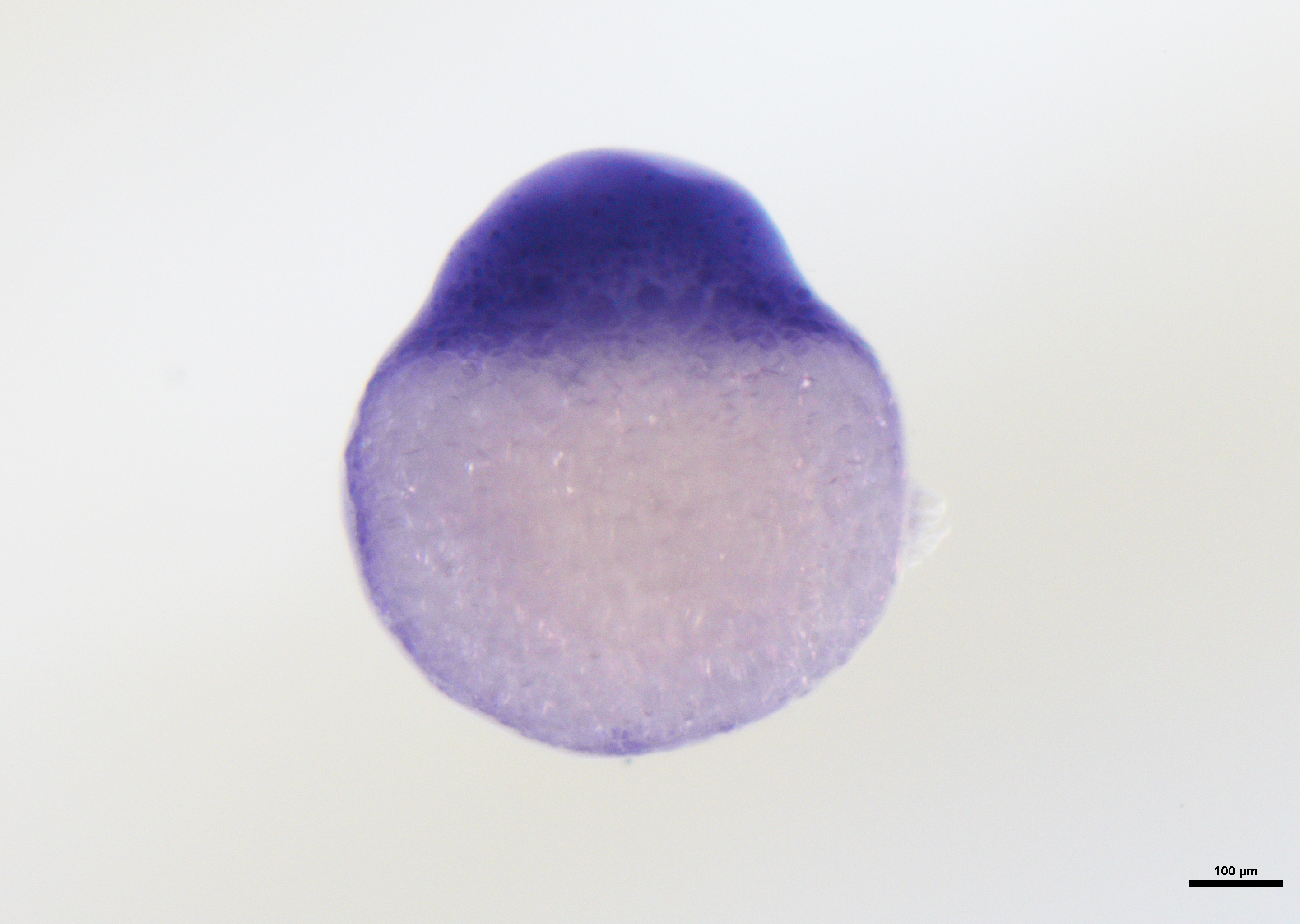

Supplement: Supplementary file 12 — Appendix Figure 3-4 Source Data [file 44319_2026_805_MOESM12_ESM.zip › Appendix Source Data 2/Appendix Fig.4/C/1. trmt61a 1-cell sibling.tif]

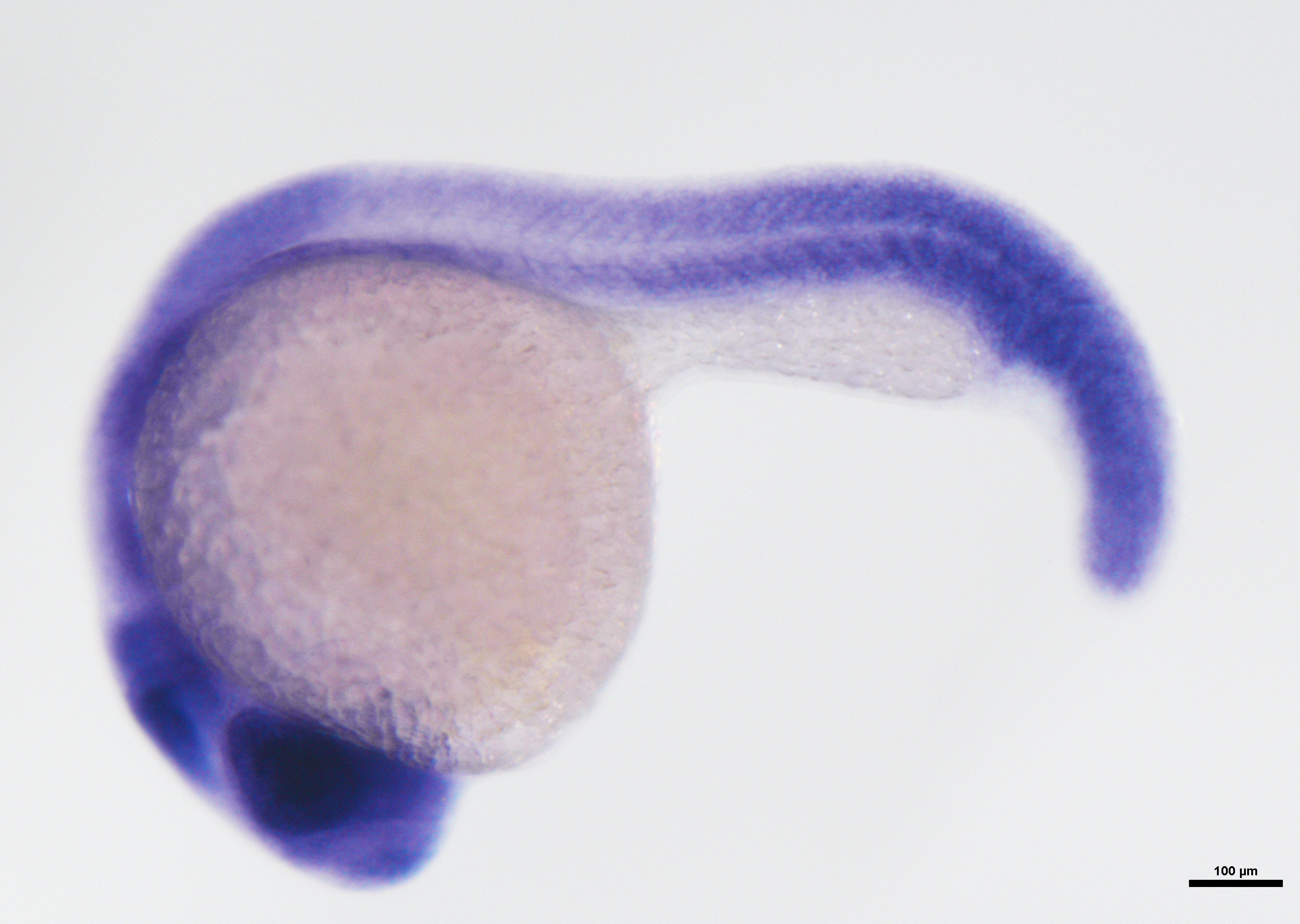

Supplement: Supplementary file 12 — Appendix Figure 3-4 Source Data [file 44319_2026_805_MOESM12_ESM.zip › Appendix Source Data 2/Appendix Fig.4/C/2. trmt61a 24hpf sibling.tif]

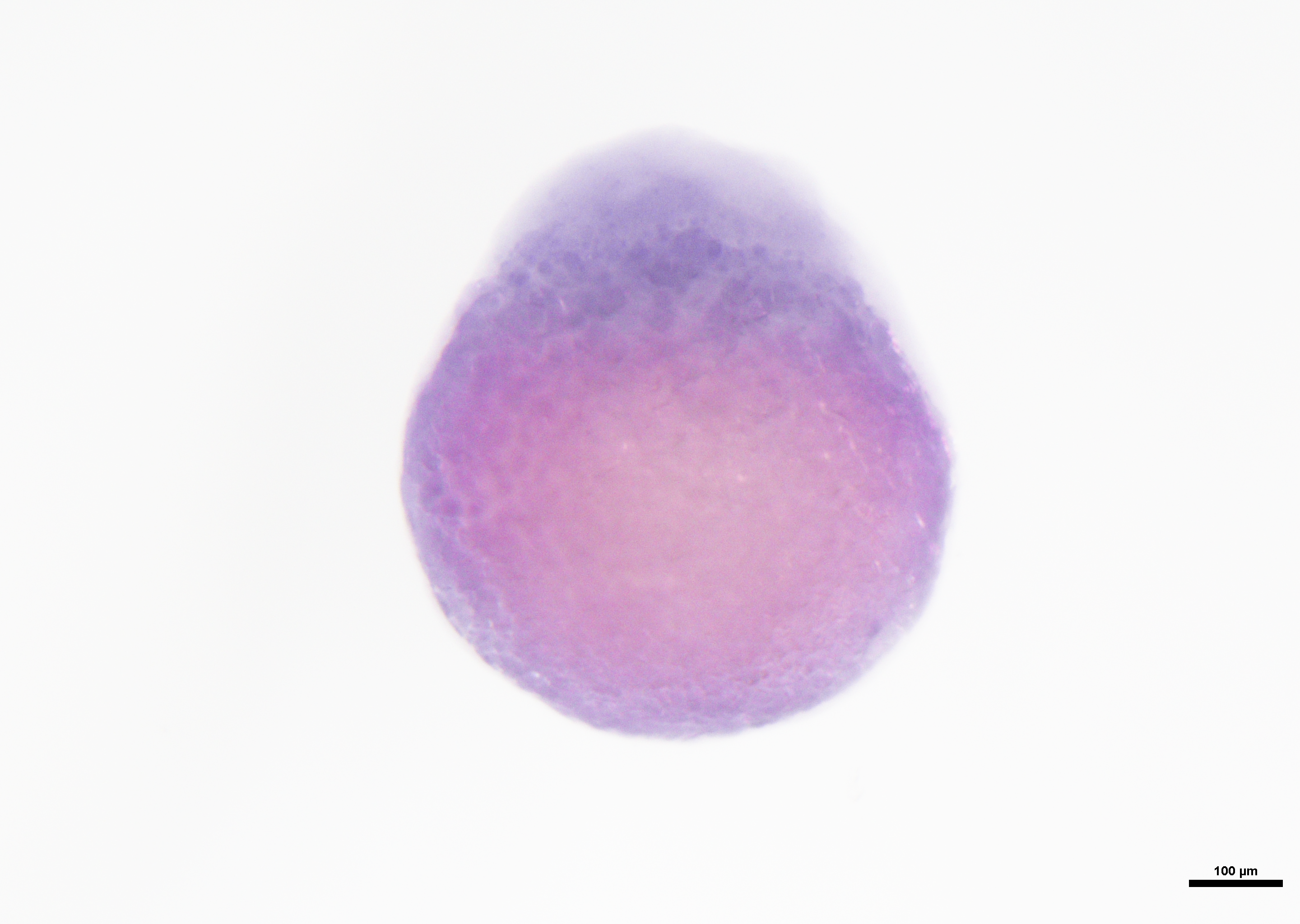

Supplement: Supplementary file 12 — Appendix Figure 3-4 Source Data [file 44319_2026_805_MOESM12_ESM.zip › Appendix Source Data 2/Appendix Fig.4/C/3. trmt61a 1-cell Mtrmt61a;trmt61-4bp.tif]

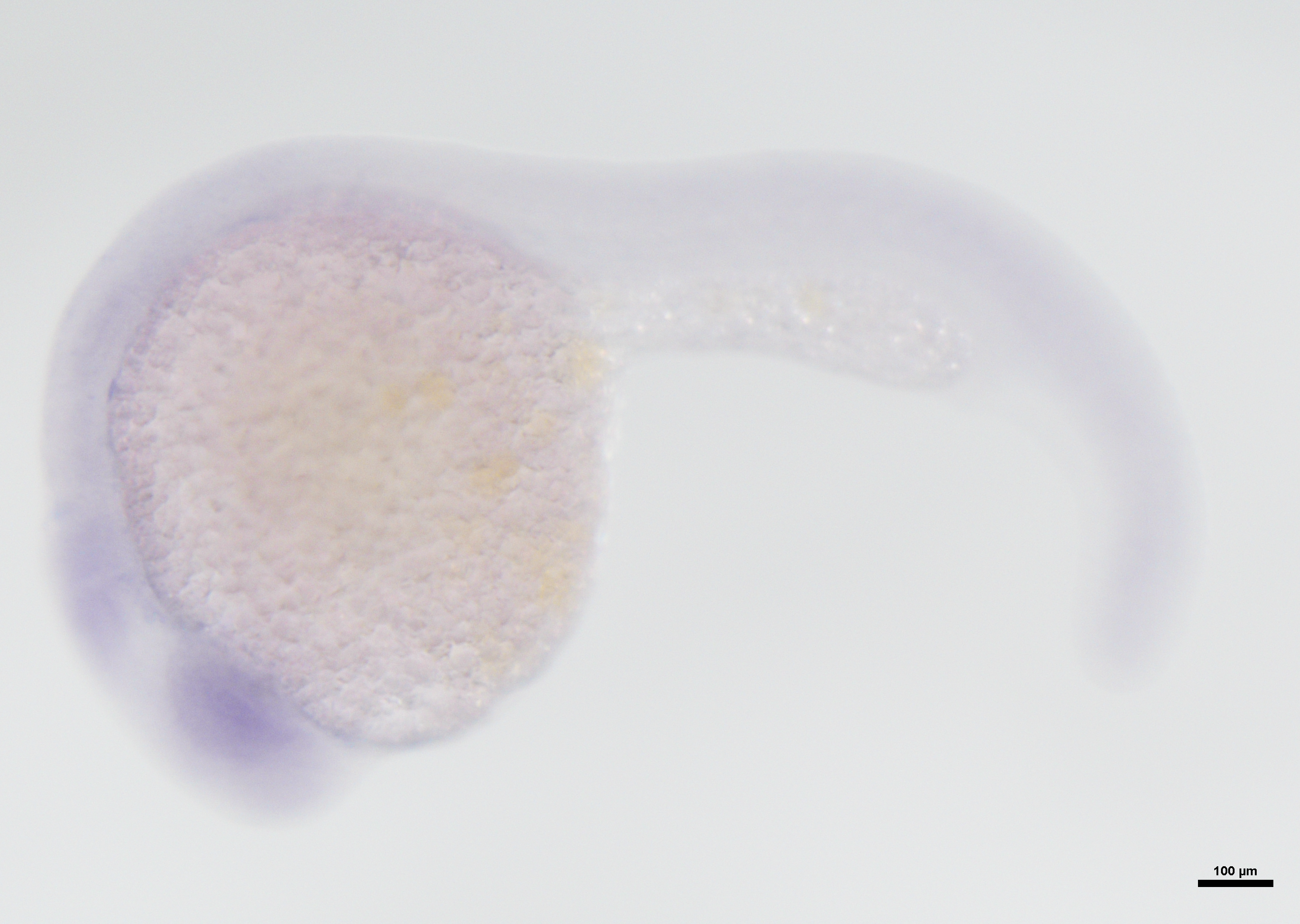

Supplement: Supplementary file 12 — Appendix Figure 3-4 Source Data [file 44319_2026_805_MOESM12_ESM.zip › Appendix Source Data 2/Appendix Fig.4/C/4. trmt61a 24hpf Mtrmt61a;trmt61-4bp.tif]

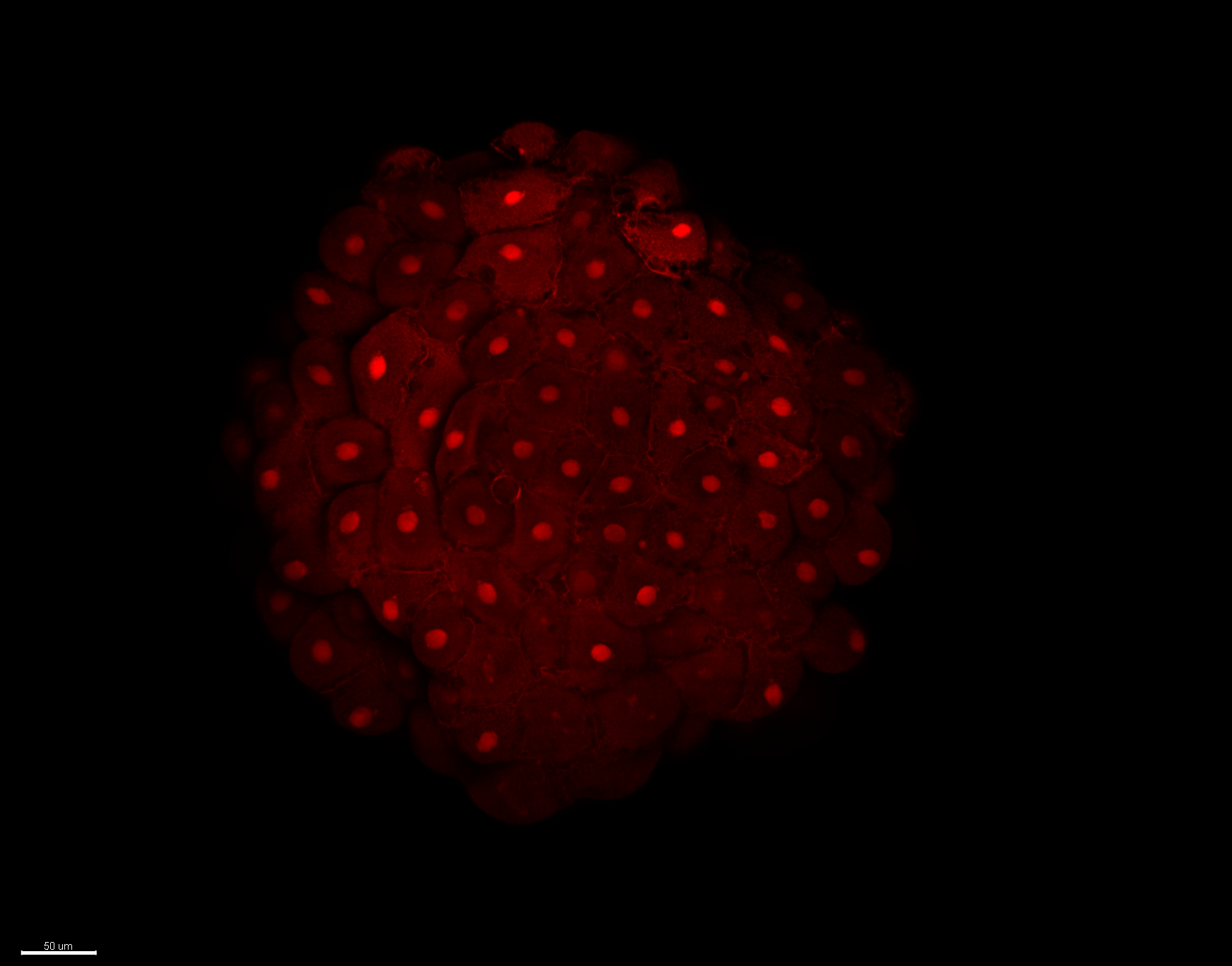

Supplement: Supplementary file 12 — Appendix Figure 3-4 Source Data [file 44319_2026_805_MOESM12_ESM.zip › Appendix Source Data 2/Appendix Fig.4/D/1. anti-trmt61a 1k-cell sibling.tif]

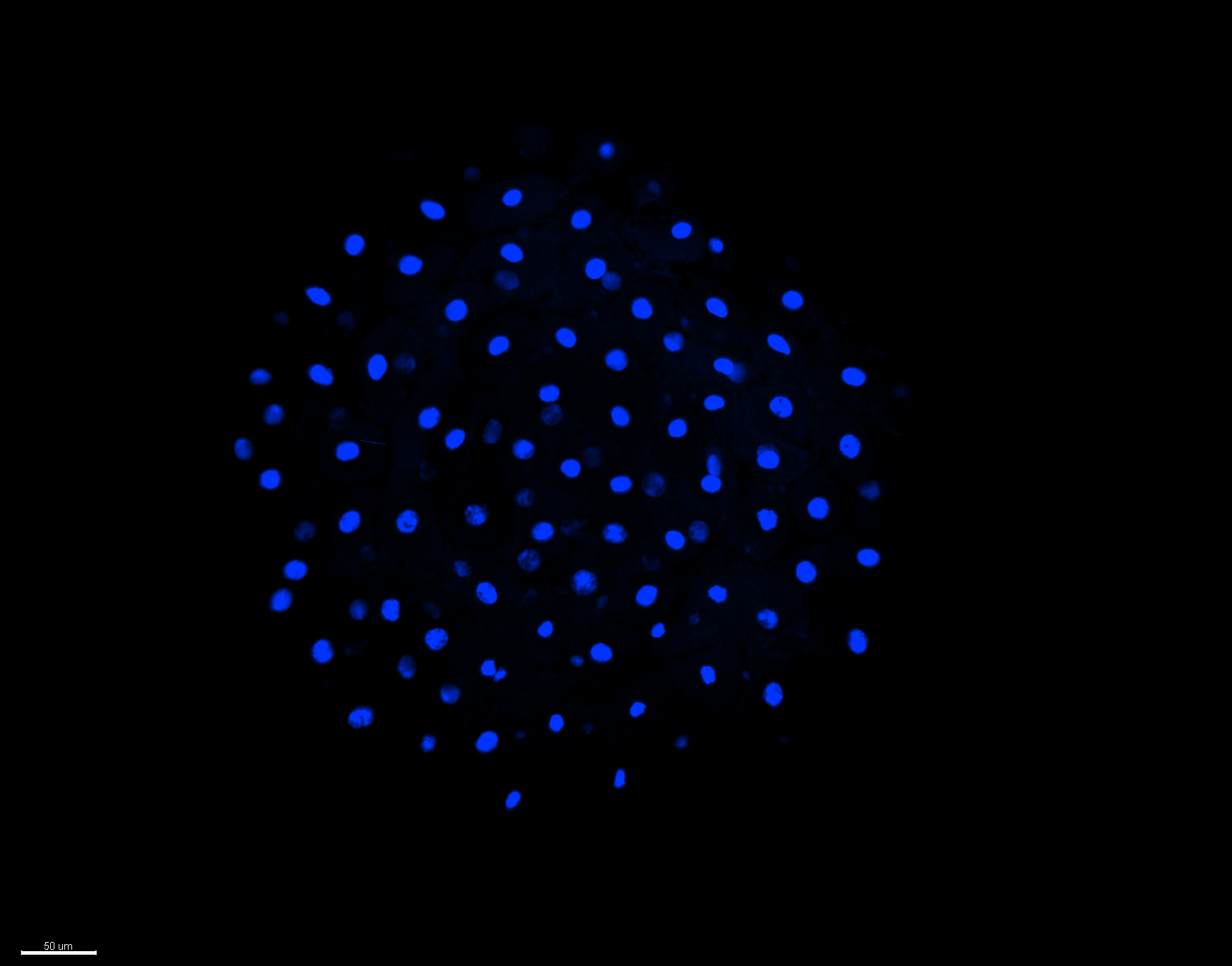

Supplement: Supplementary file 12 — Appendix Figure 3-4 Source Data [file 44319_2026_805_MOESM12_ESM.zip › Appendix Source Data 2/Appendix Fig.4/D/2. dapi 1k-cell sibling.tif]

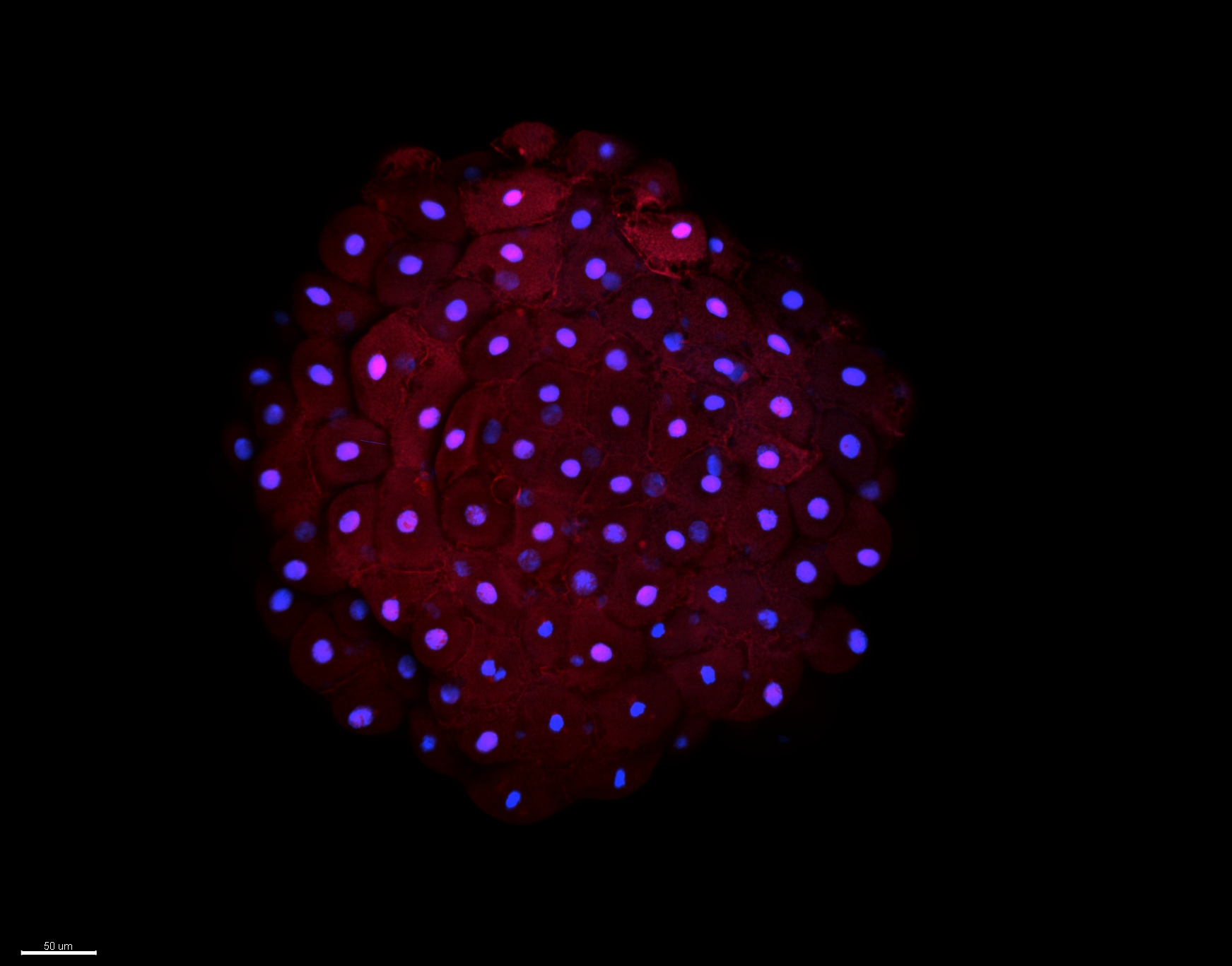

Supplement: Supplementary file 12 — Appendix Figure 3-4 Source Data [file 44319_2026_805_MOESM12_ESM.zip › Appendix Source Data 2/Appendix Fig.4/D/3. merge 1k-cell sibling.tif]

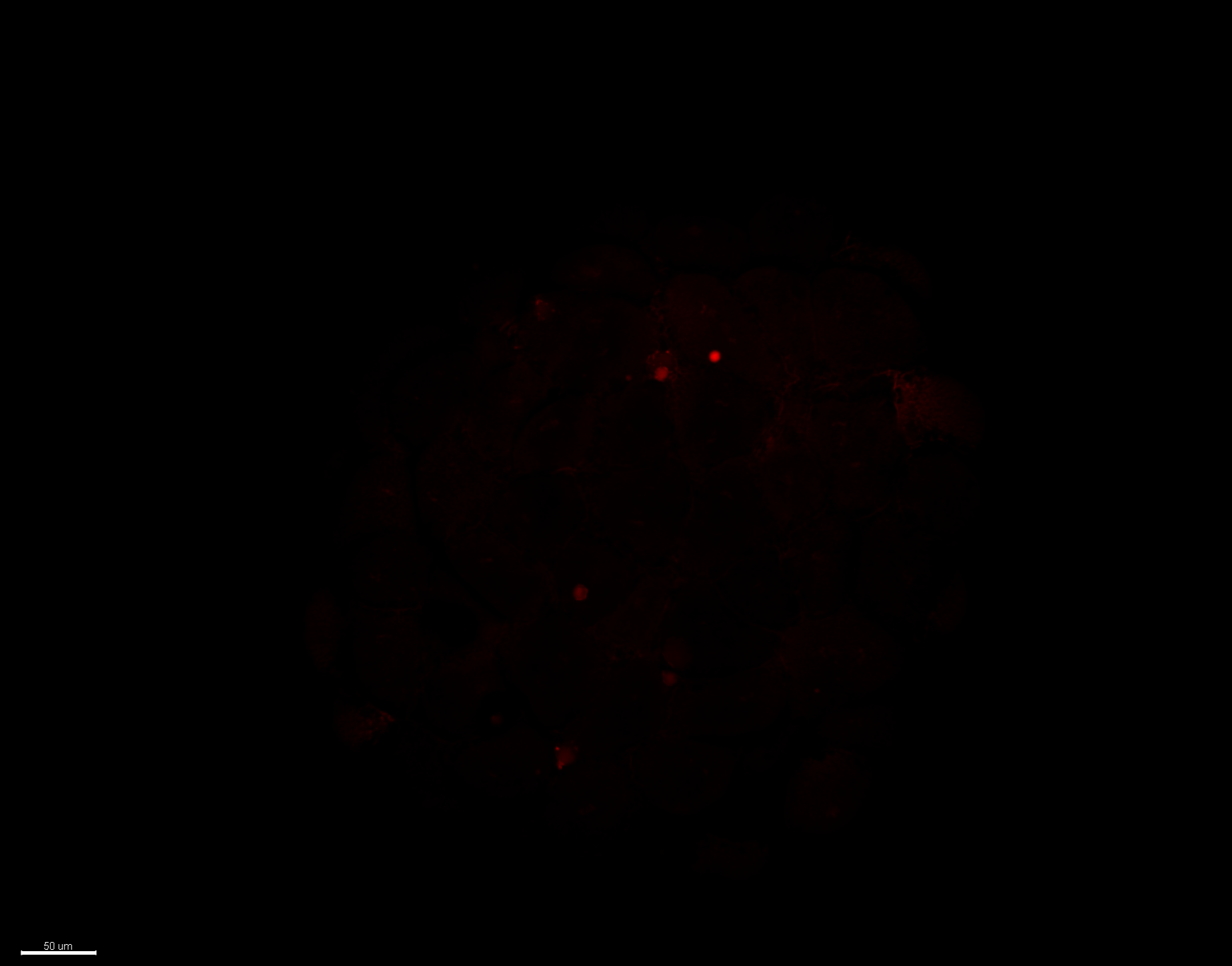

Supplement: Supplementary file 12 — Appendix Figure 3-4 Source Data [file 44319_2026_805_MOESM12_ESM.zip › Appendix Source Data 2/Appendix Fig.4/D/4. anti-trmt61a 1k-cell Mtrmt61a;trmt61a-4bp.tif]

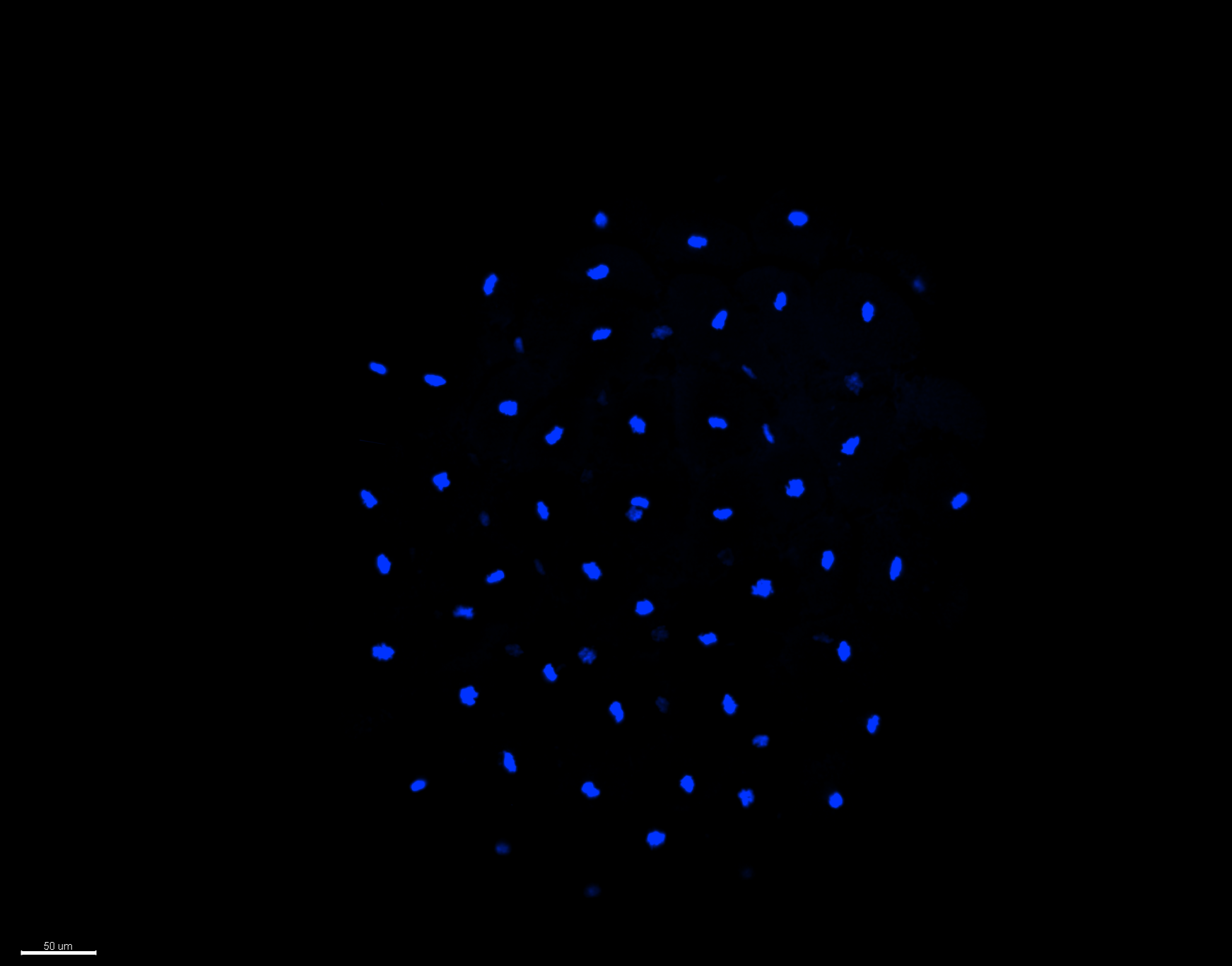

Supplement: Supplementary file 12 — Appendix Figure 3-4 Source Data [file 44319_2026_805_MOESM12_ESM.zip › Appendix Source Data 2/Appendix Fig.4/D/5. dapi 1k-cell Mtrmt61a;trmt61a-4bp.tif]

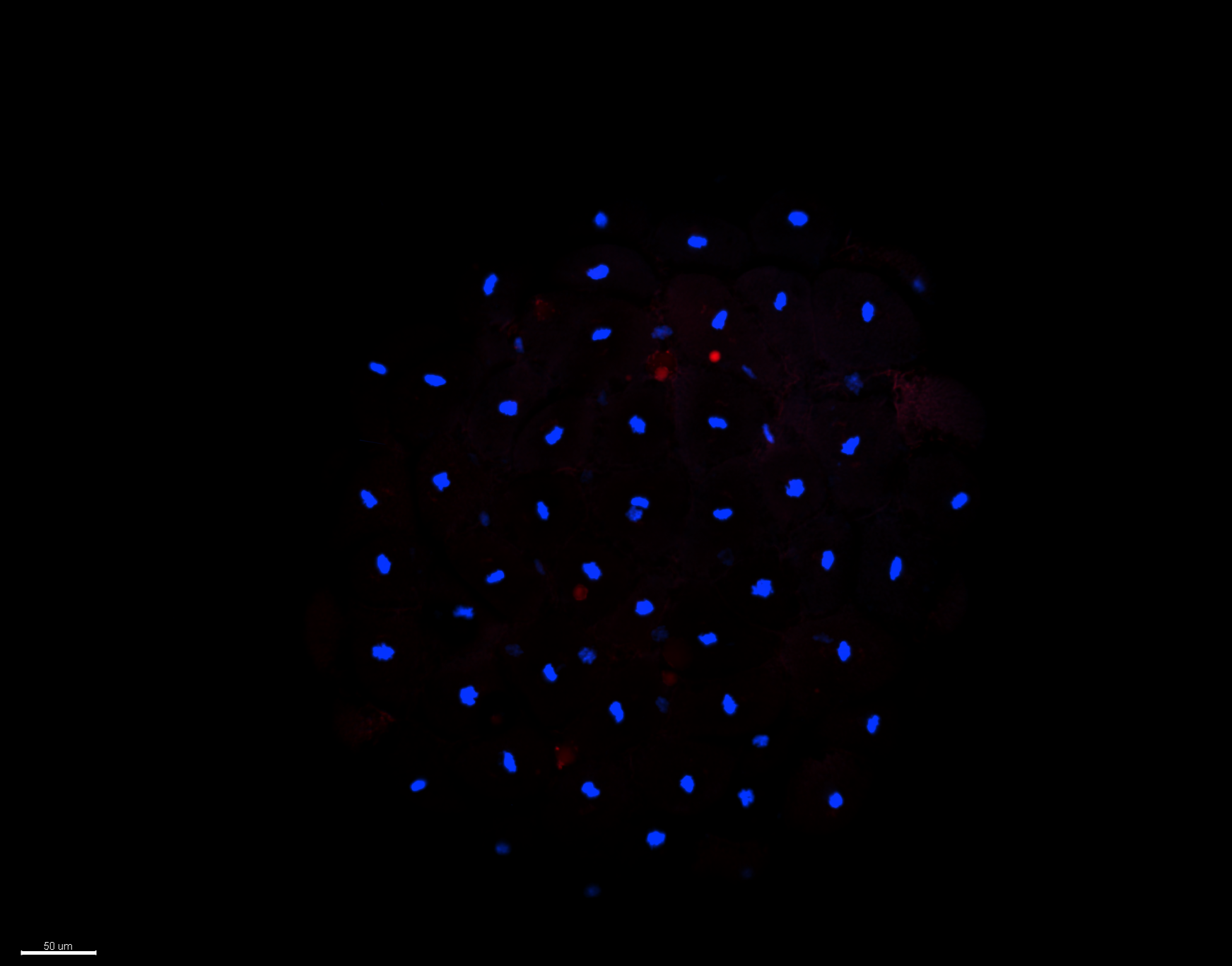

Supplement: Supplementary file 12 — Appendix Figure 3-4 Source Data [file 44319_2026_805_MOESM12_ESM.zip › Appendix Source Data 2/Appendix Fig.4/D/6. merge 1k-cell Mtrmt61a;trmt61a-4bp.tif]

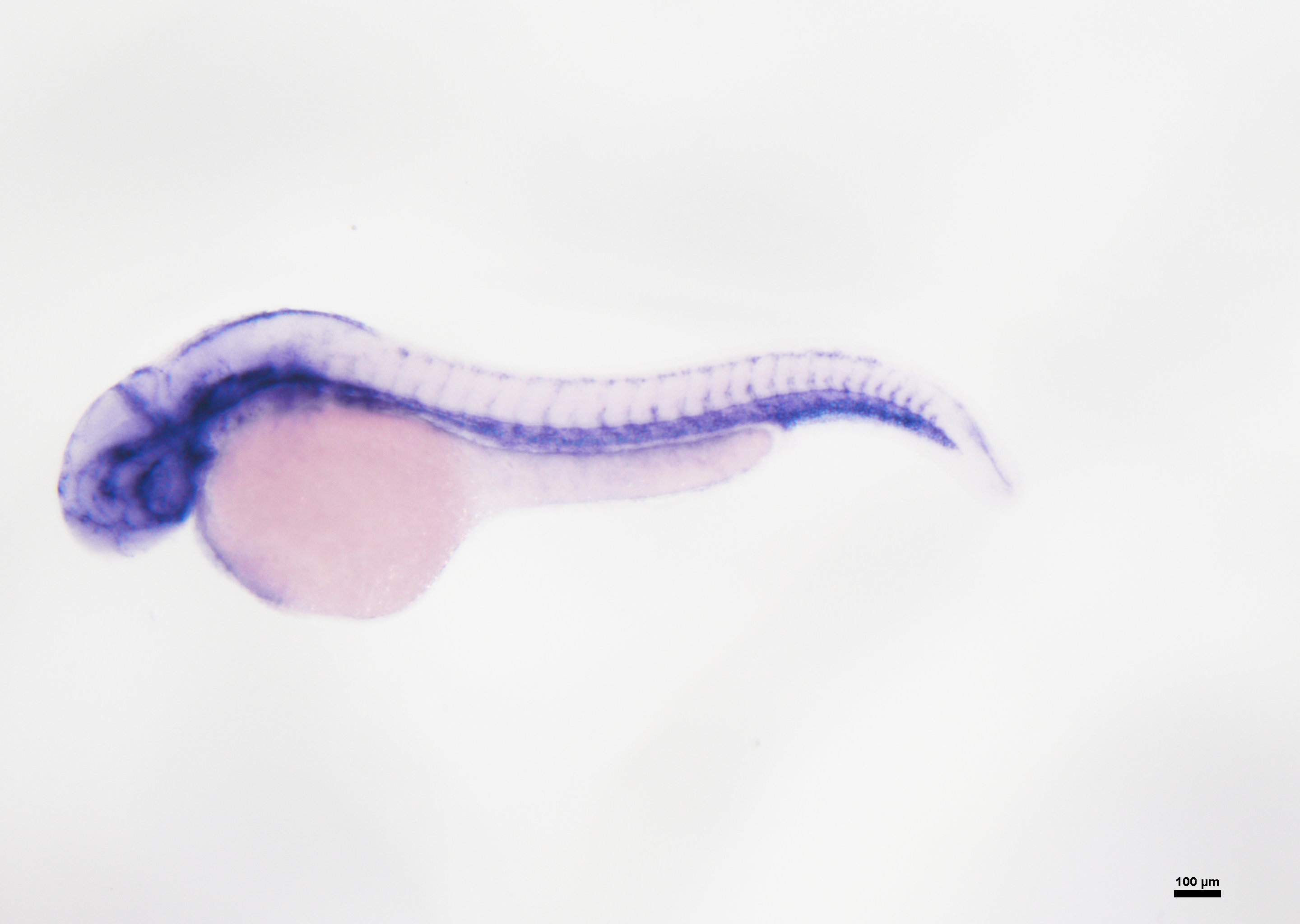

Supplement: Supplementary file 12 — Appendix Figure 3-4 Source Data [file 44319_2026_805_MOESM12_ESM.zip › Appendix Source Data 2/Appendix Fig.4/E/1. kdrl 36hpf sibling.tif]

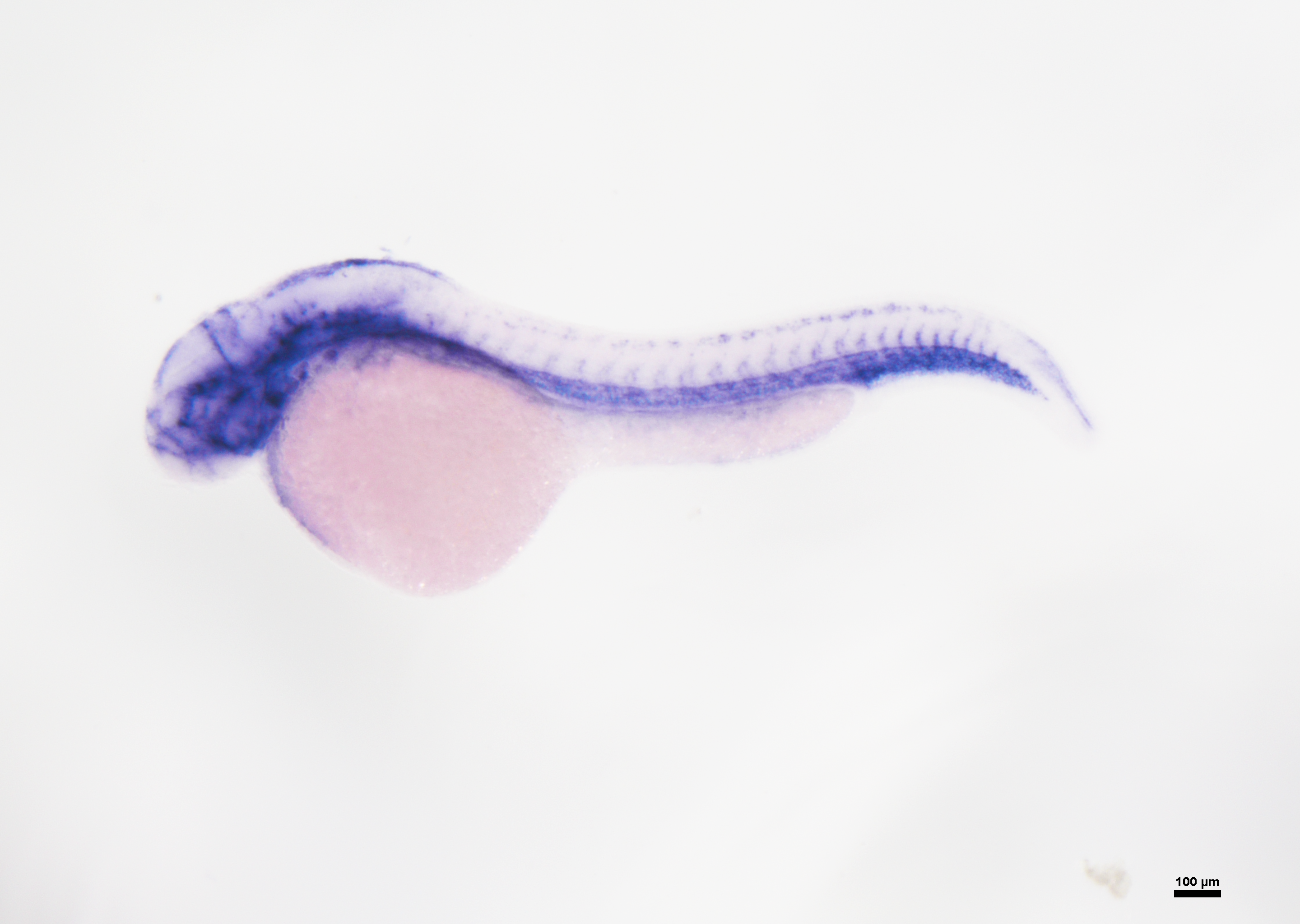

Supplement: Supplementary file 12 — Appendix Figure 3-4 Source Data [file 44319_2026_805_MOESM12_ESM.zip › Appendix Source Data 2/Appendix Fig.4/E/2. kdrl 36hpf Mtrmt61a;trmt61a-4bp.tif]

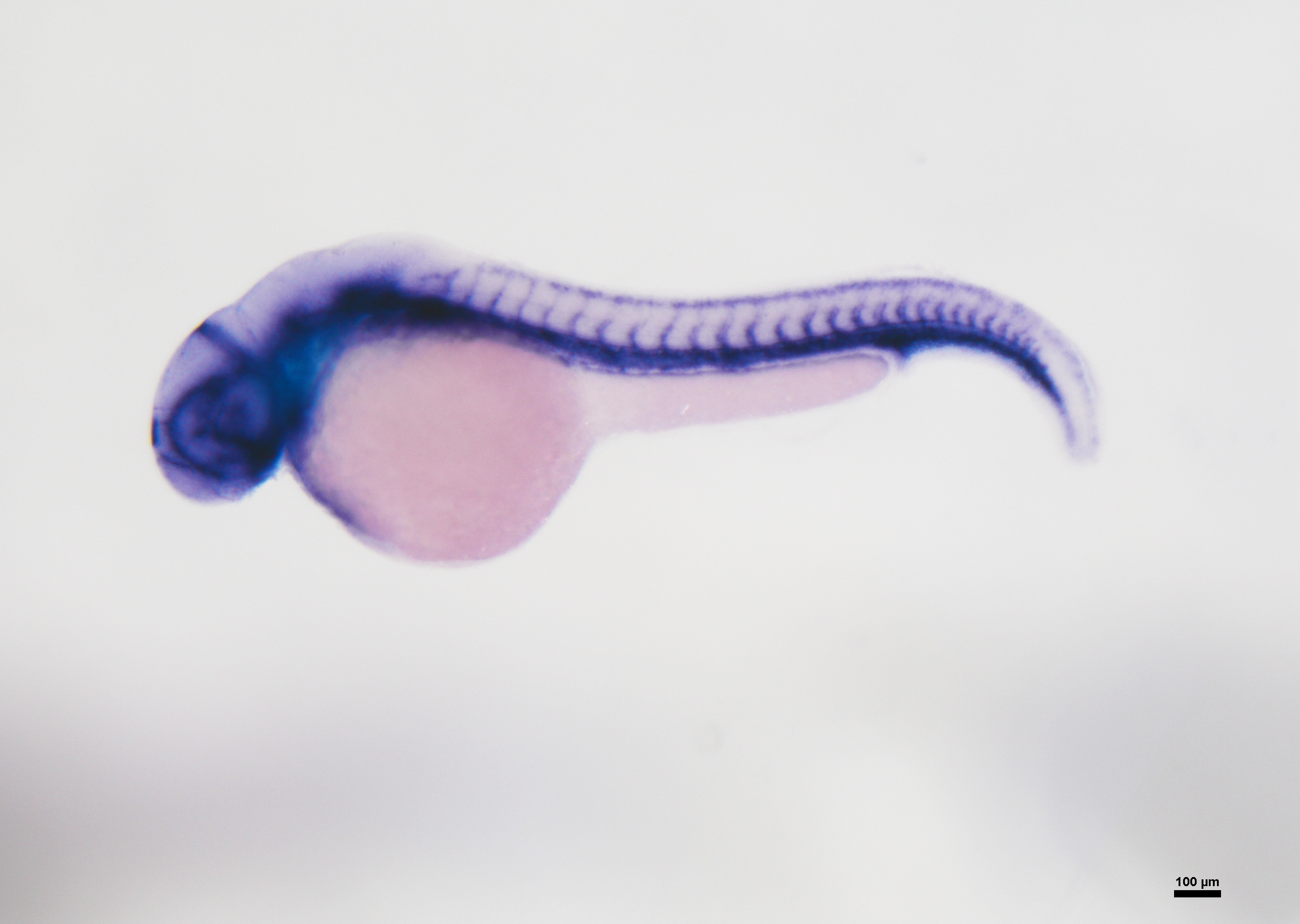

Supplement: Supplementary file 12 — Appendix Figure 3-4 Source Data [file 44319_2026_805_MOESM12_ESM.zip › Appendix Source Data 2/Appendix Fig.4/E/3. fli1a 36hpf sibling.tif]

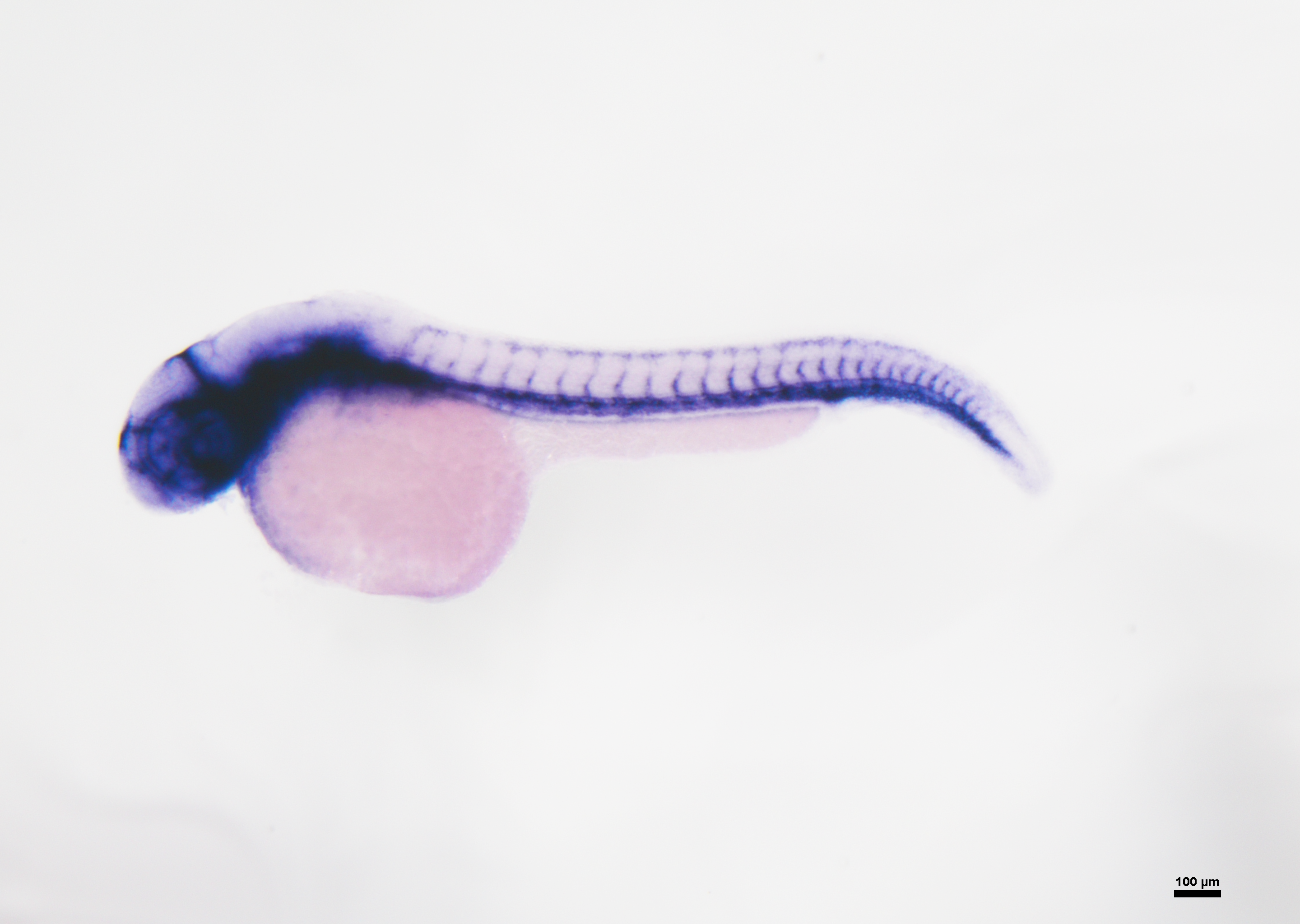

Supplement: Supplementary file 12 — Appendix Figure 3-4 Source Data [file 44319_2026_805_MOESM12_ESM.zip › Appendix Source Data 2/Appendix Fig.4/E/4. fli1a 36hpf Mtrmt61a;trmt61a-4bp.tif]

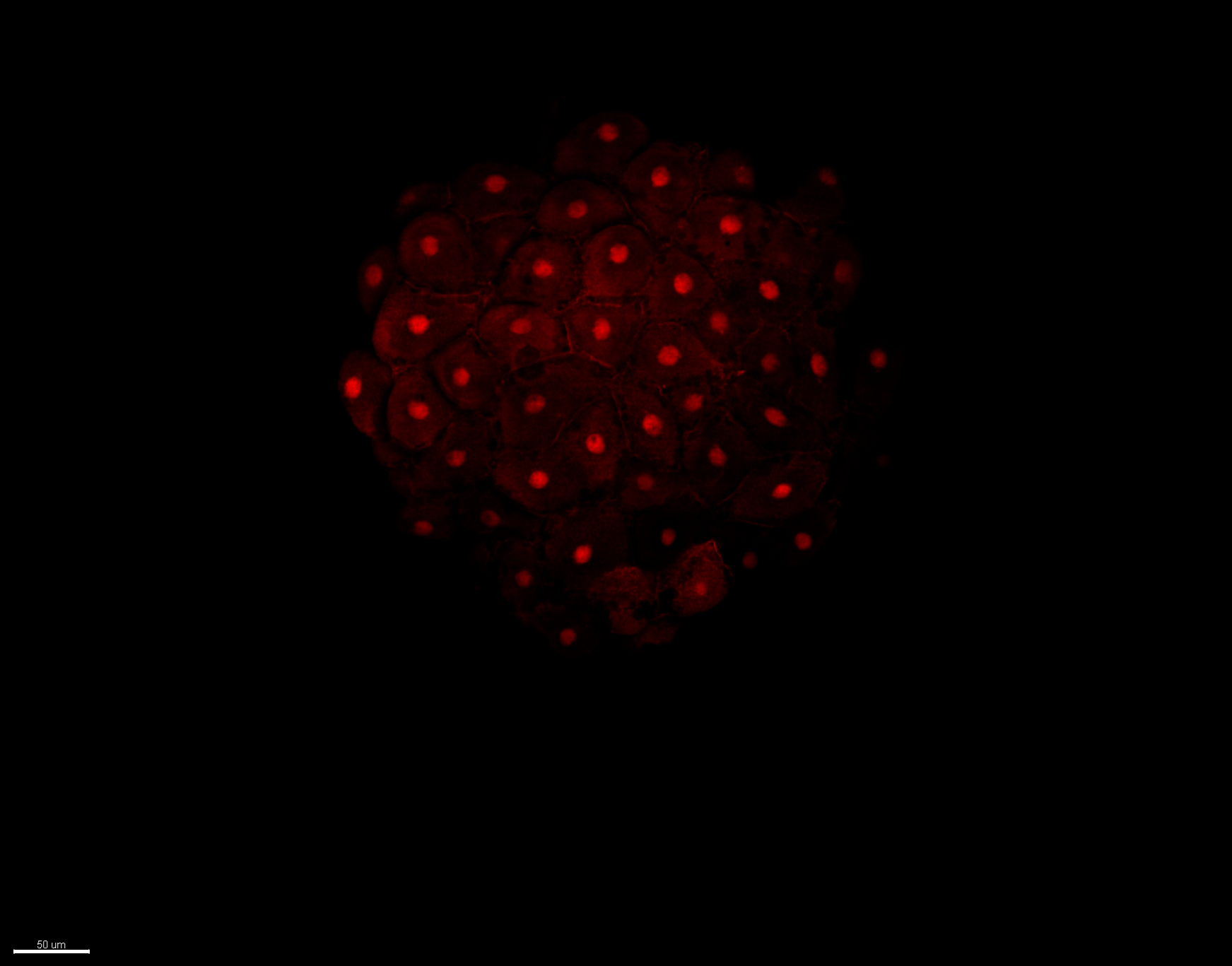

Supplement: Supplementary file 13 — Appendix Figure 5-7 Source Data [file 44319_2026_805_MOESM13_ESM.zip › Appendix Source Data 3/Appendix Fig.5/D/1. anti-trmt61a 1k-cell trmt61aD181AD181A.tif]

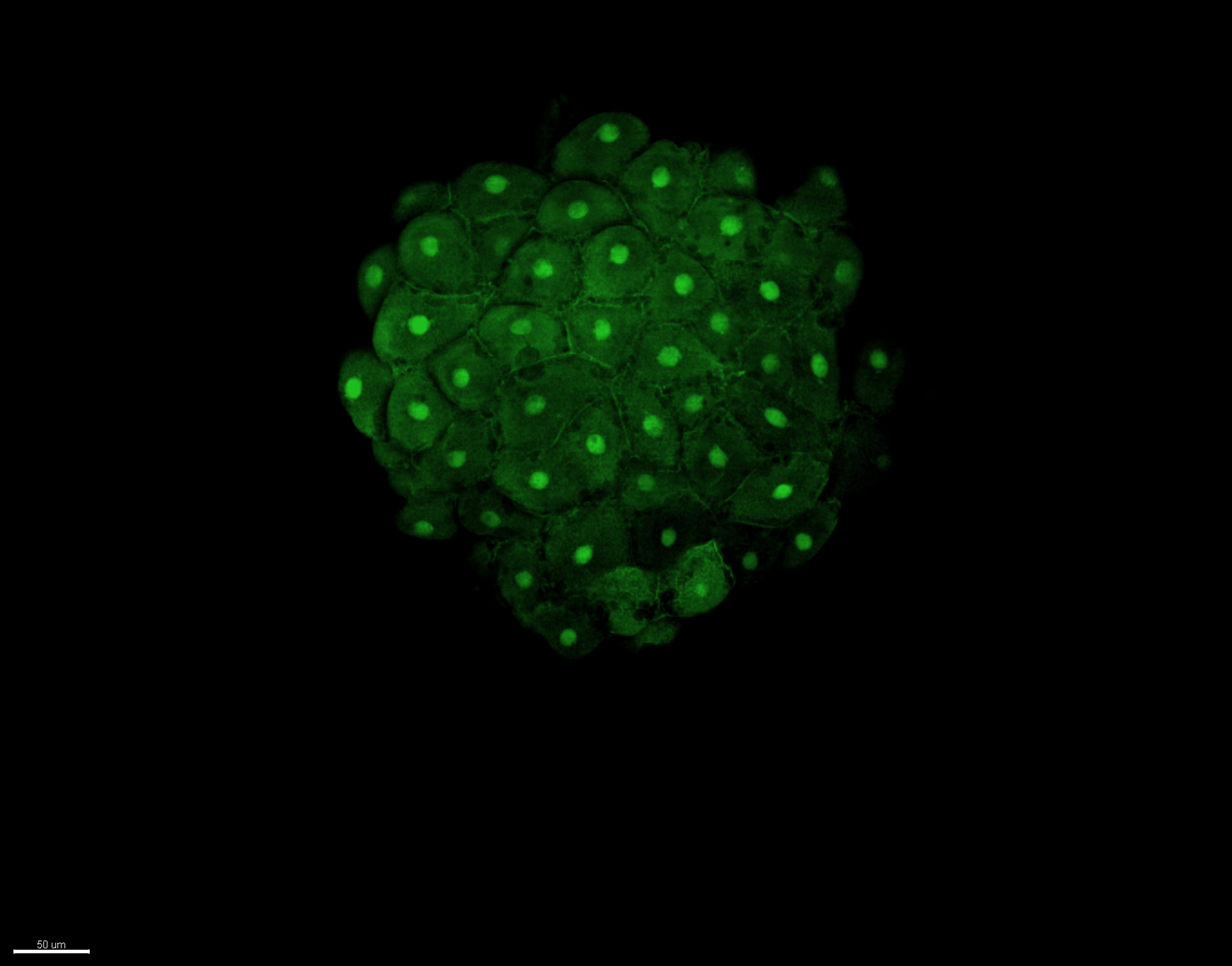

Supplement: Supplementary file 13 — Appendix Figure 5-7 Source Data [file 44319_2026_805_MOESM13_ESM.zip › Appendix Source Data 3/Appendix Fig.5/D/2. egfp 1k-cell trmt61aD181AD181A.tif]

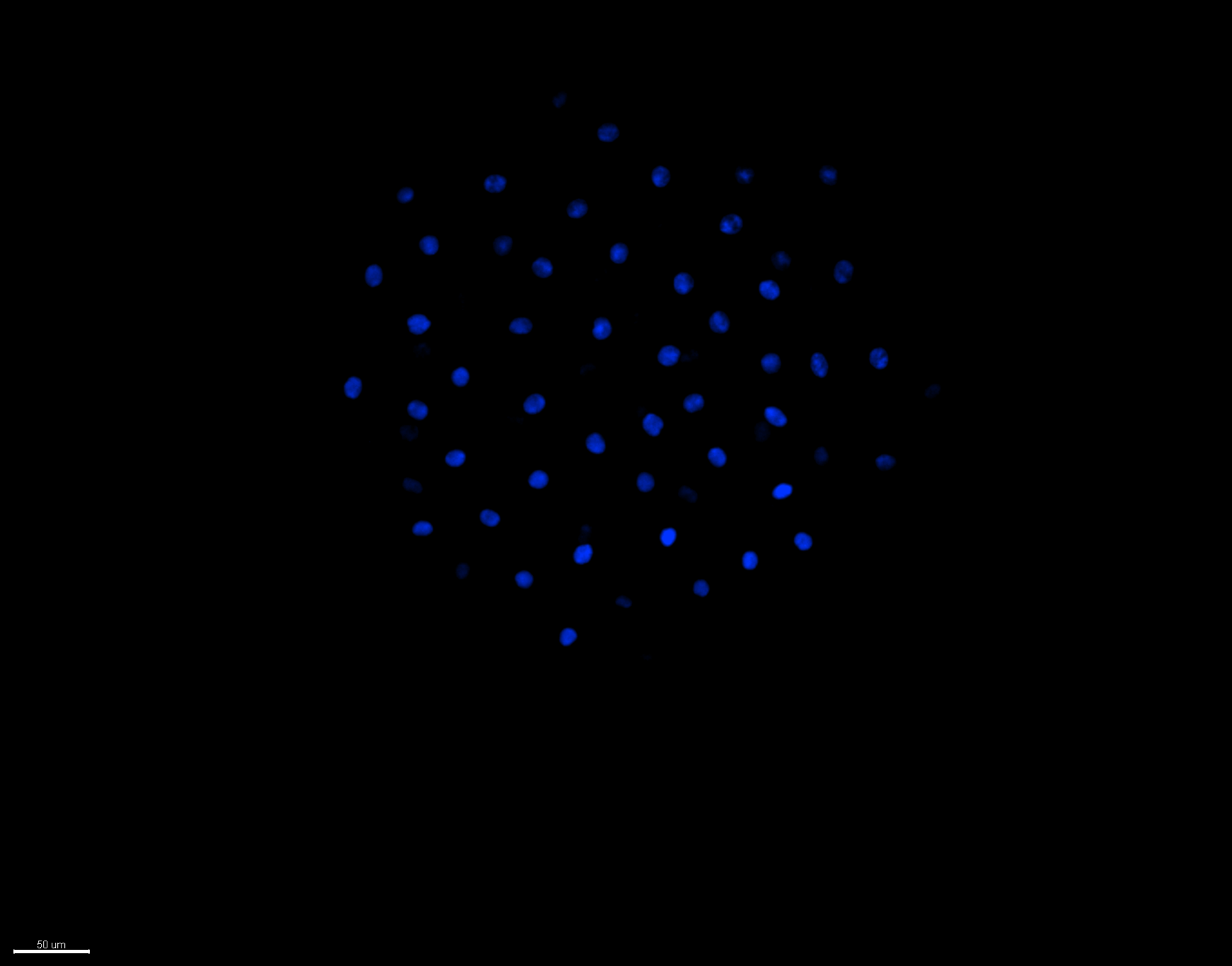

Supplement: Supplementary file 13 — Appendix Figure 5-7 Source Data [file 44319_2026_805_MOESM13_ESM.zip › Appendix Source Data 3/Appendix Fig.5/D/3. dapi 1k-cell trmt61aD181AD181A.tif]

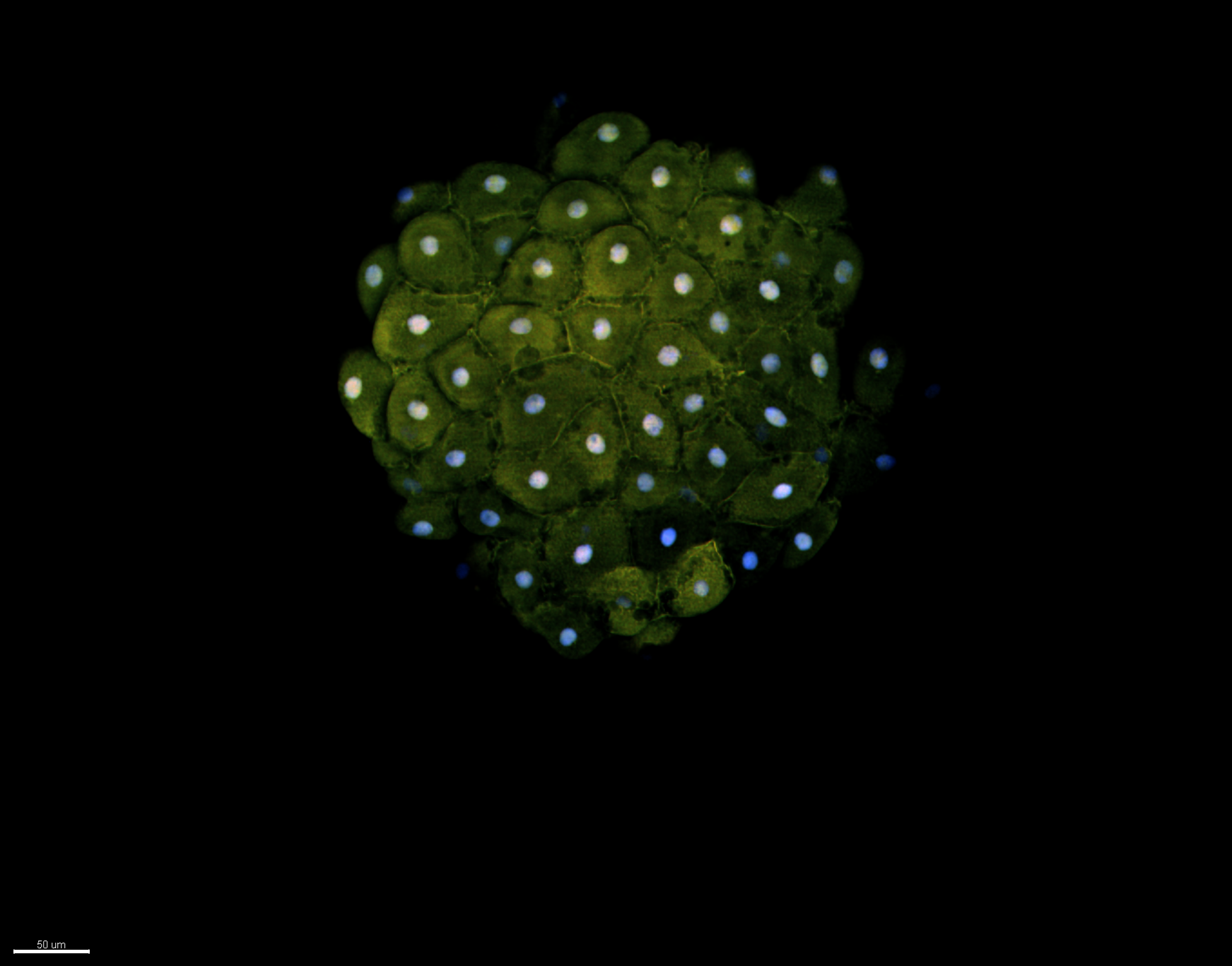

Supplement: Supplementary file 13 — Appendix Figure 5-7 Source Data [file 44319_2026_805_MOESM13_ESM.zip › Appendix Source Data 3/Appendix Fig.5/D/4. merge 1k-cell trmt61aD181AD181A.tif]

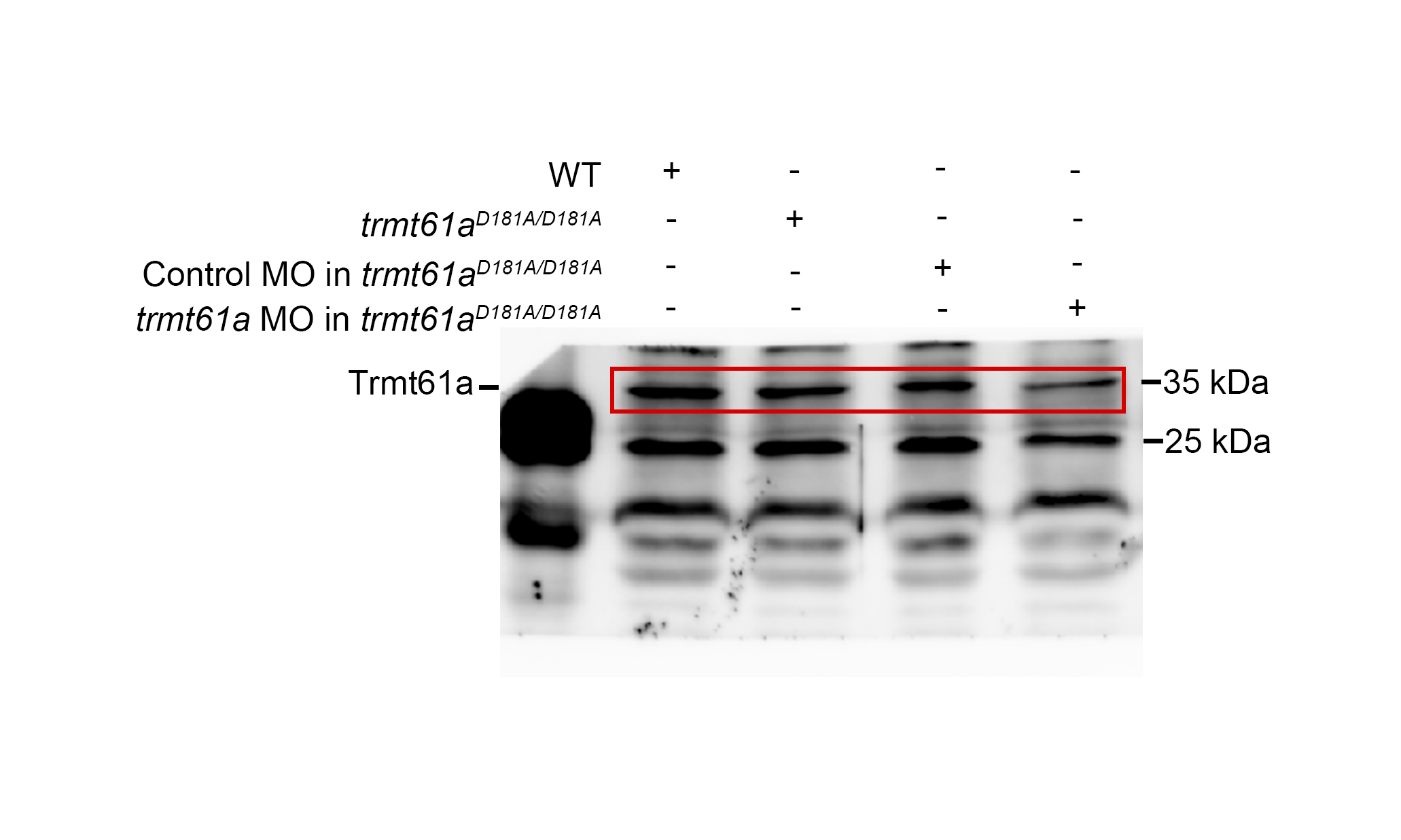

Supplement: Supplementary file 13 — Appendix Figure 5-7 Source Data [file 44319_2026_805_MOESM13_ESM.zip › Appendix Source Data 3/Appendix Fig.5/E/5E_Trmt61a WB.tif]

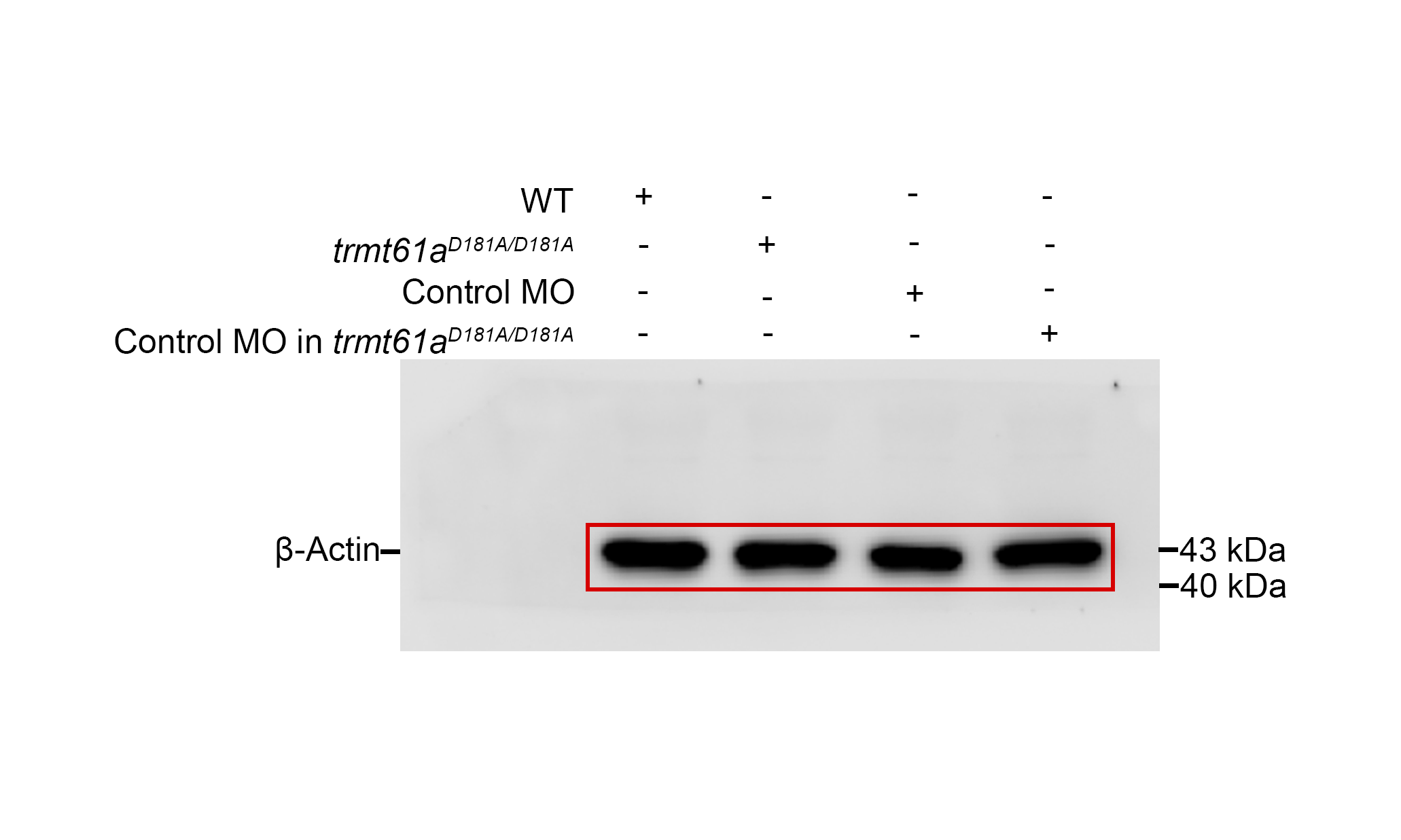

Supplement: Supplementary file 13 — Appendix Figure 5-7 Source Data [file 44319_2026_805_MOESM13_ESM.zip › Appendix Source Data 3/Appendix Fig.5/E/5E_β-Actin WB.tif]

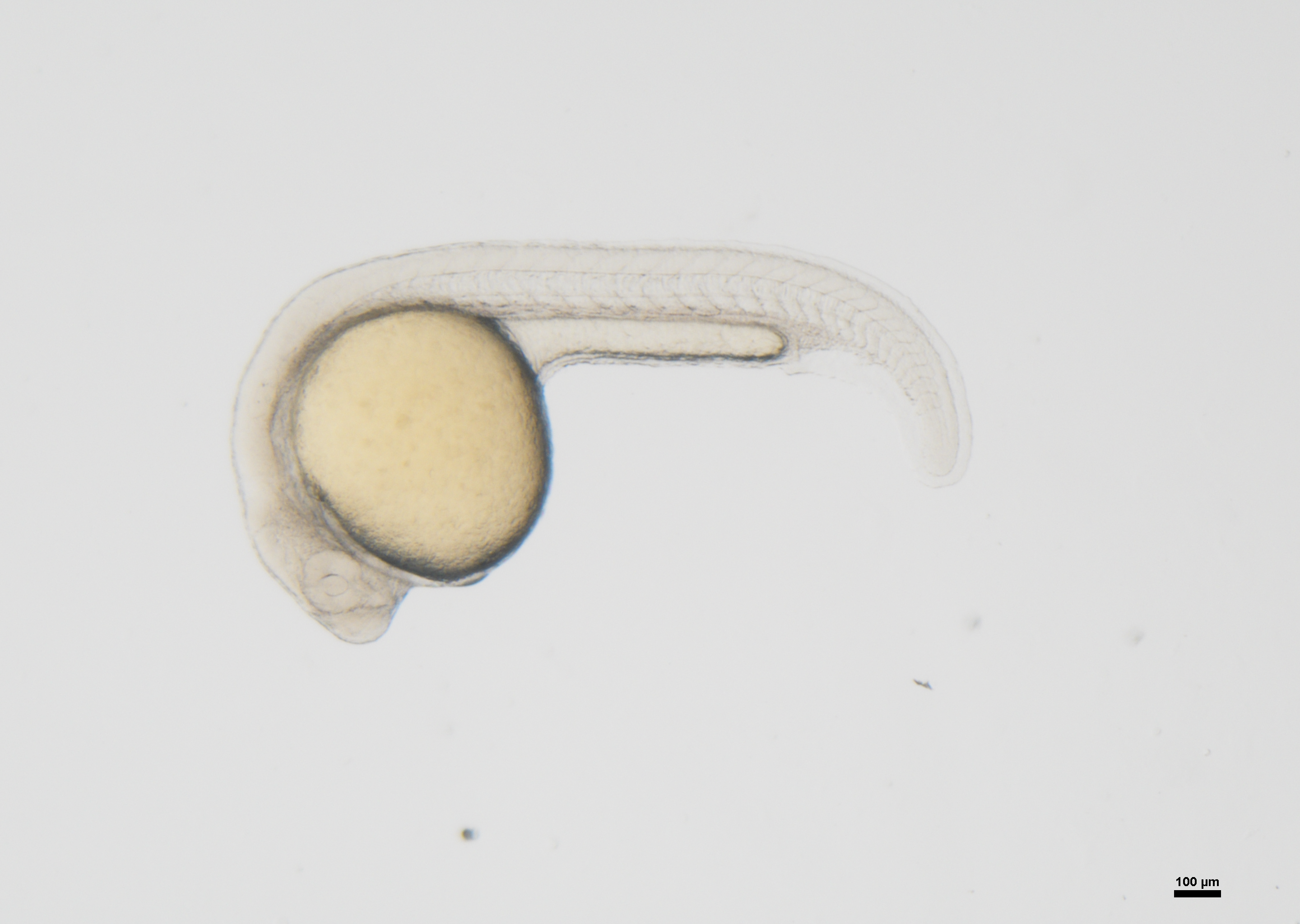

Supplement: Supplementary file 13 — Appendix Figure 5-7 Source Data [file 44319_2026_805_MOESM13_ESM.zip › Appendix Source Data 3/Appendix Fig.5/F/1. 24hpf WT.tif]

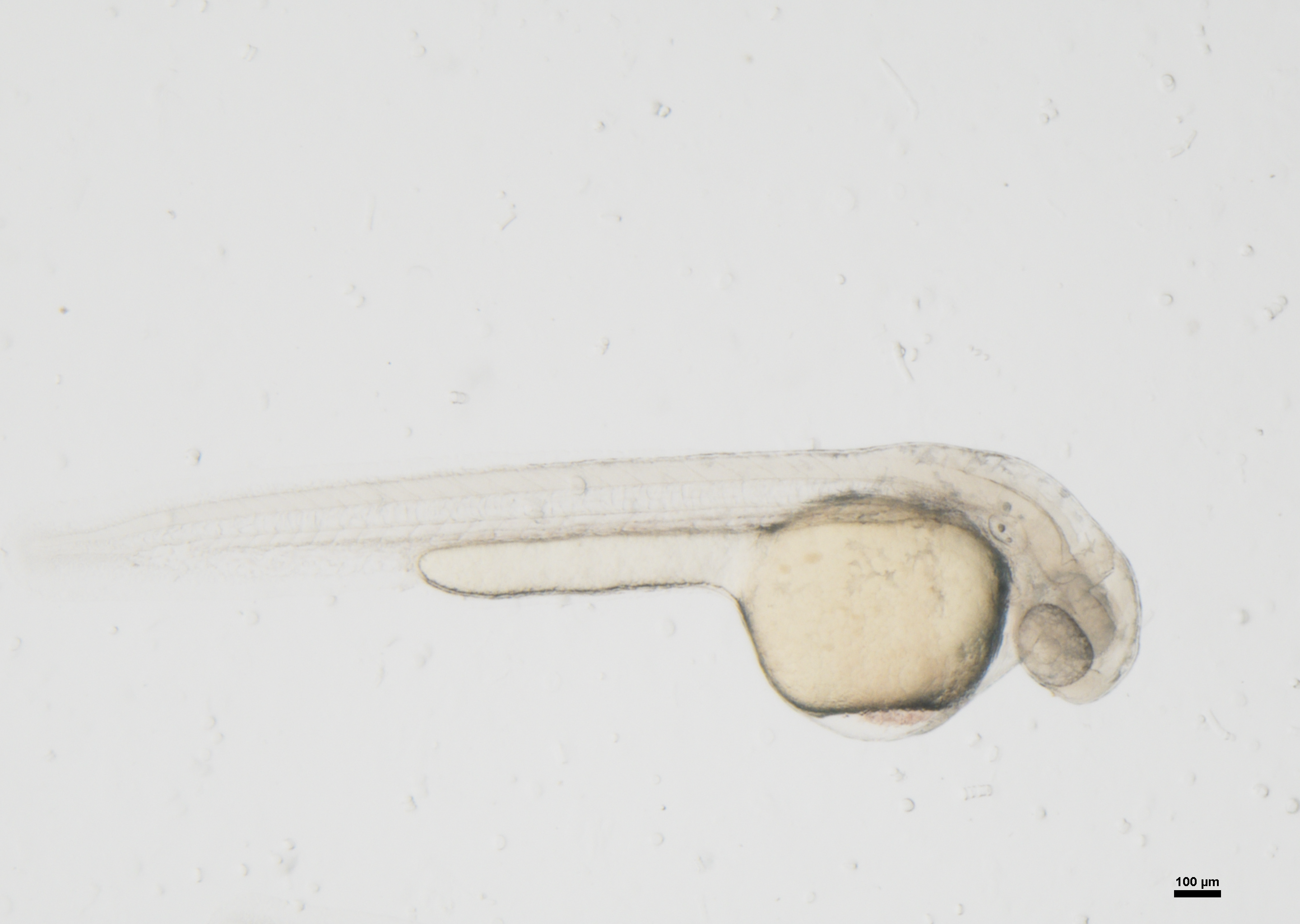

Supplement: Supplementary file 13 — Appendix Figure 5-7 Source Data [file 44319_2026_805_MOESM13_ESM.zip › Appendix Source Data 3/Appendix Fig.5/F/2. 36hpf WT.tif]

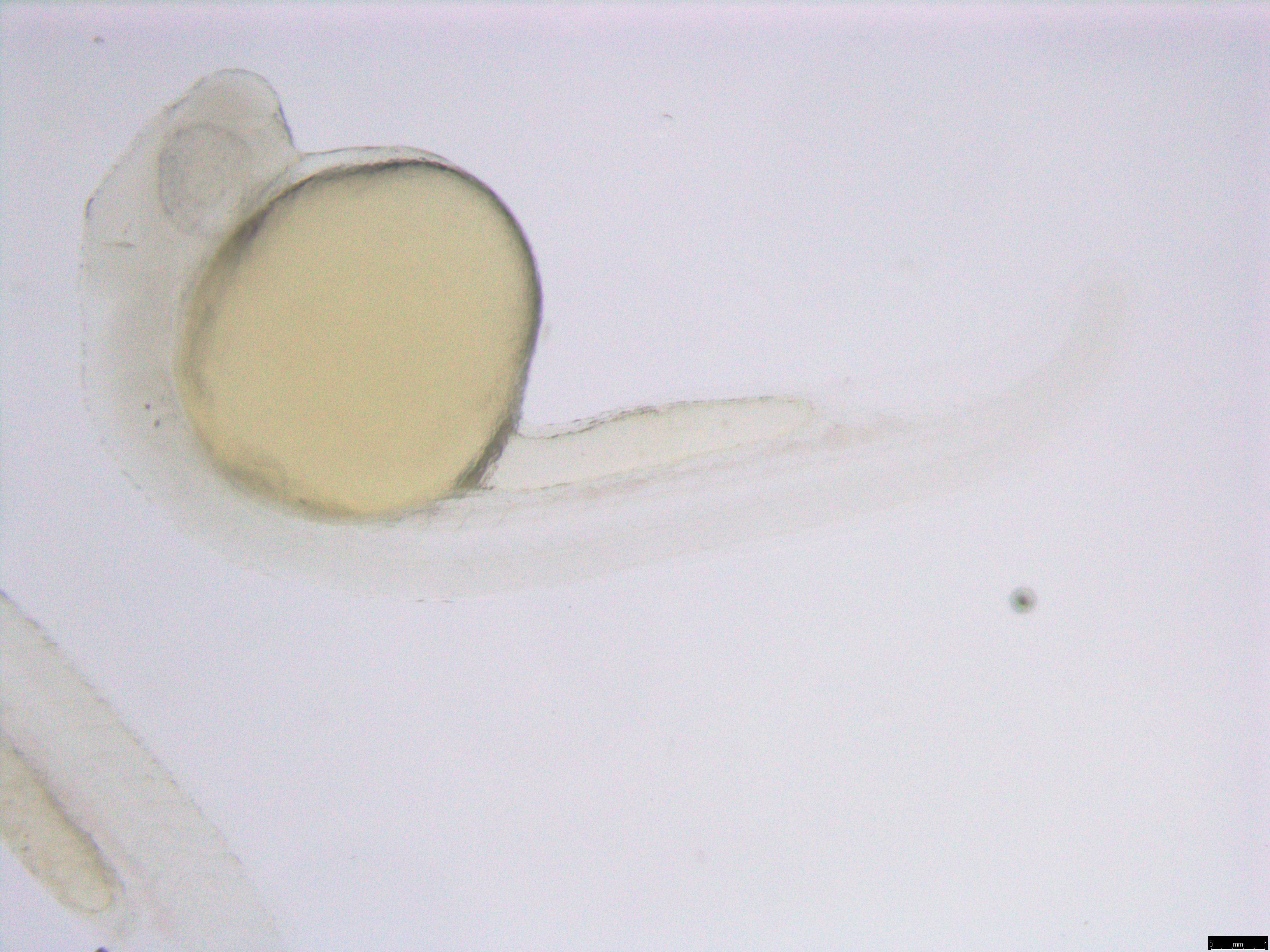

Supplement: Supplementary file 13 — Appendix Figure 5-7 Source Data [file 44319_2026_805_MOESM13_ESM.zip › Appendix Source Data 3/Appendix Fig.5/F/3. 24hpf trmt61aD181AD181A.tif]

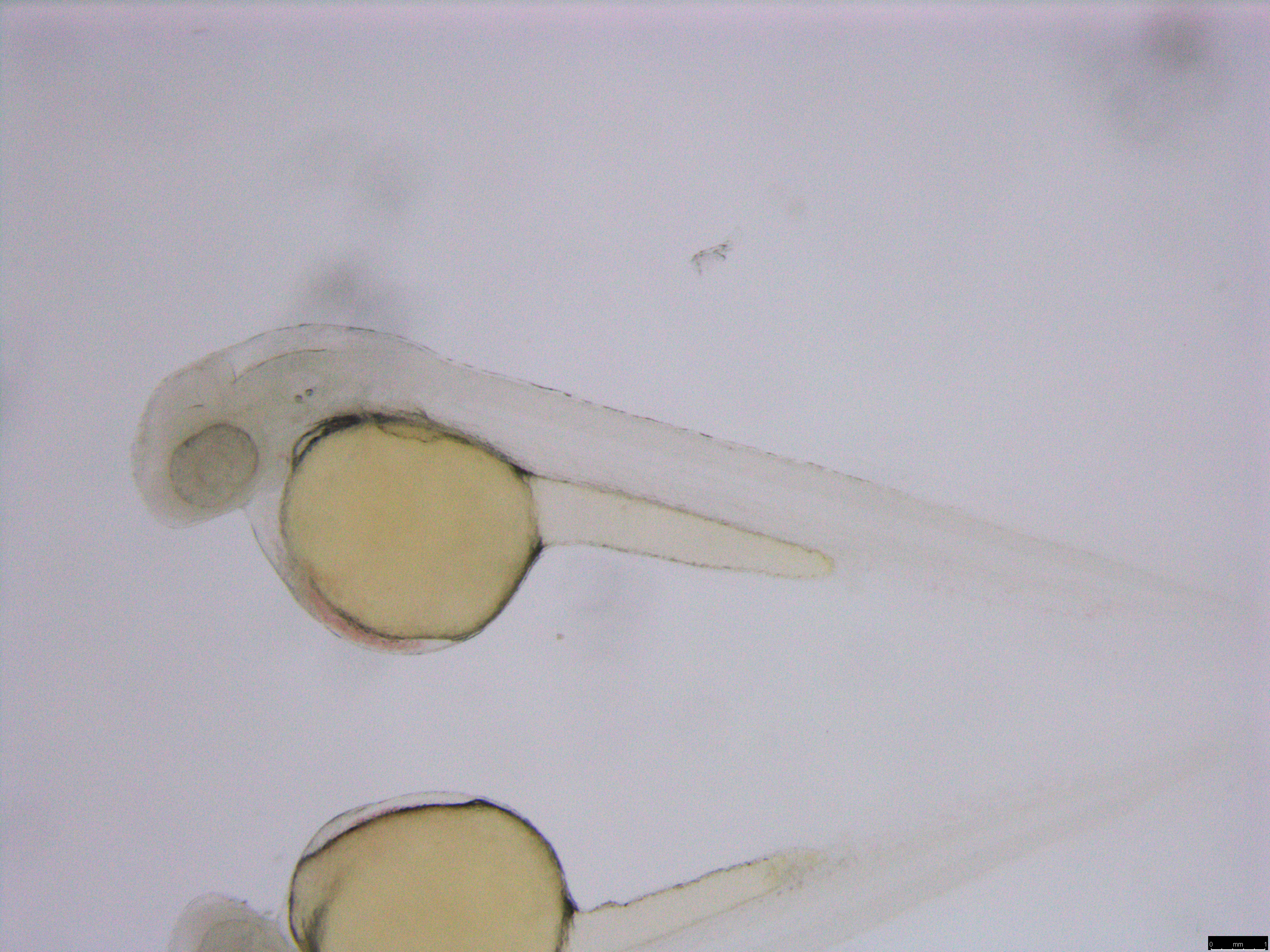

Supplement: Supplementary file 13 — Appendix Figure 5-7 Source Data [file 44319_2026_805_MOESM13_ESM.zip › Appendix Source Data 3/Appendix Fig.5/F/4. 36hpf trmt61aD181AD181A.tif]

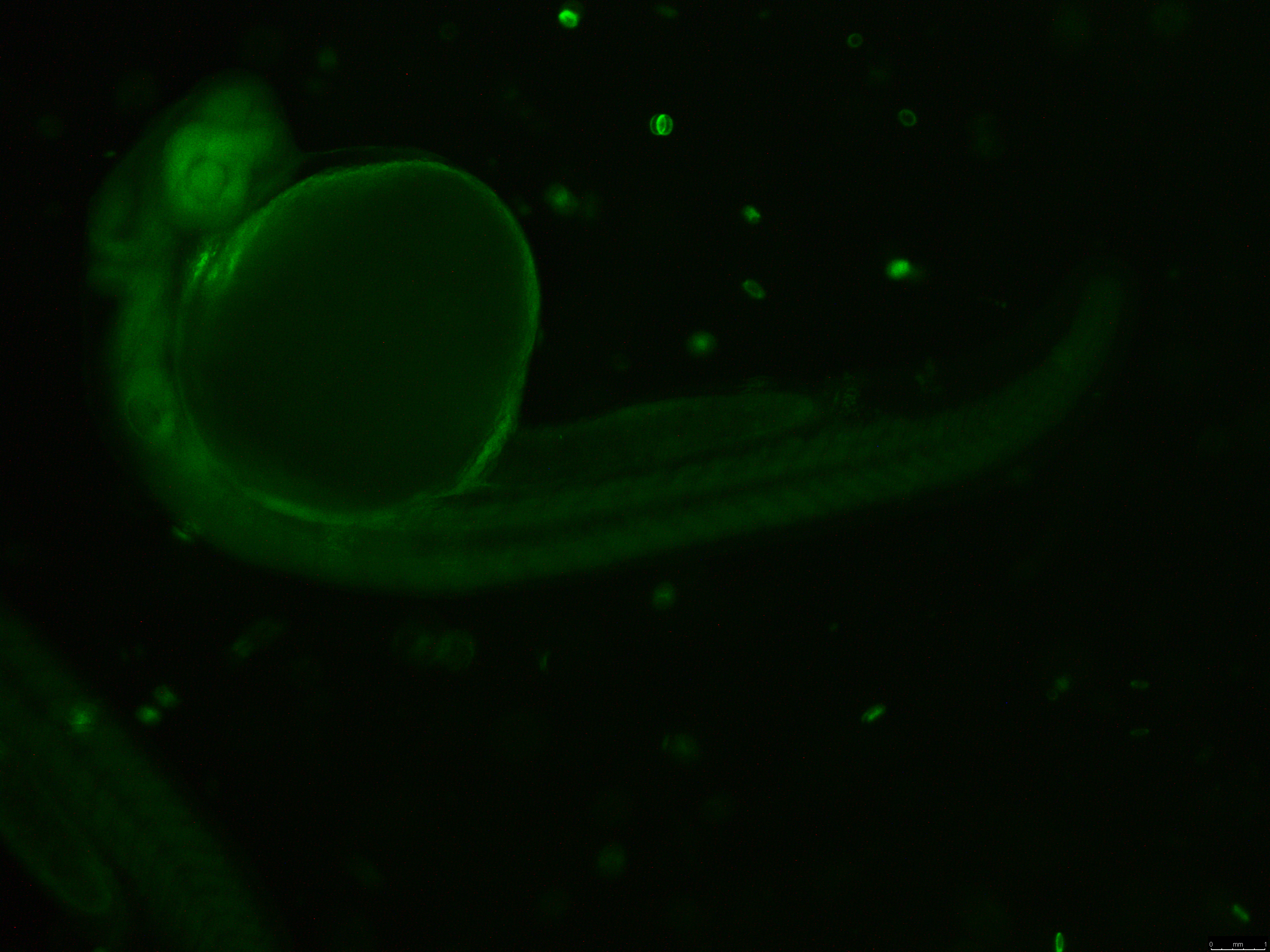

Supplement: Supplementary file 13 — Appendix Figure 5-7 Source Data [file 44319_2026_805_MOESM13_ESM.zip › Appendix Source Data 3/Appendix Fig.5/F/5. EGFP 24hpf trmt61aD181AD181A.tif]

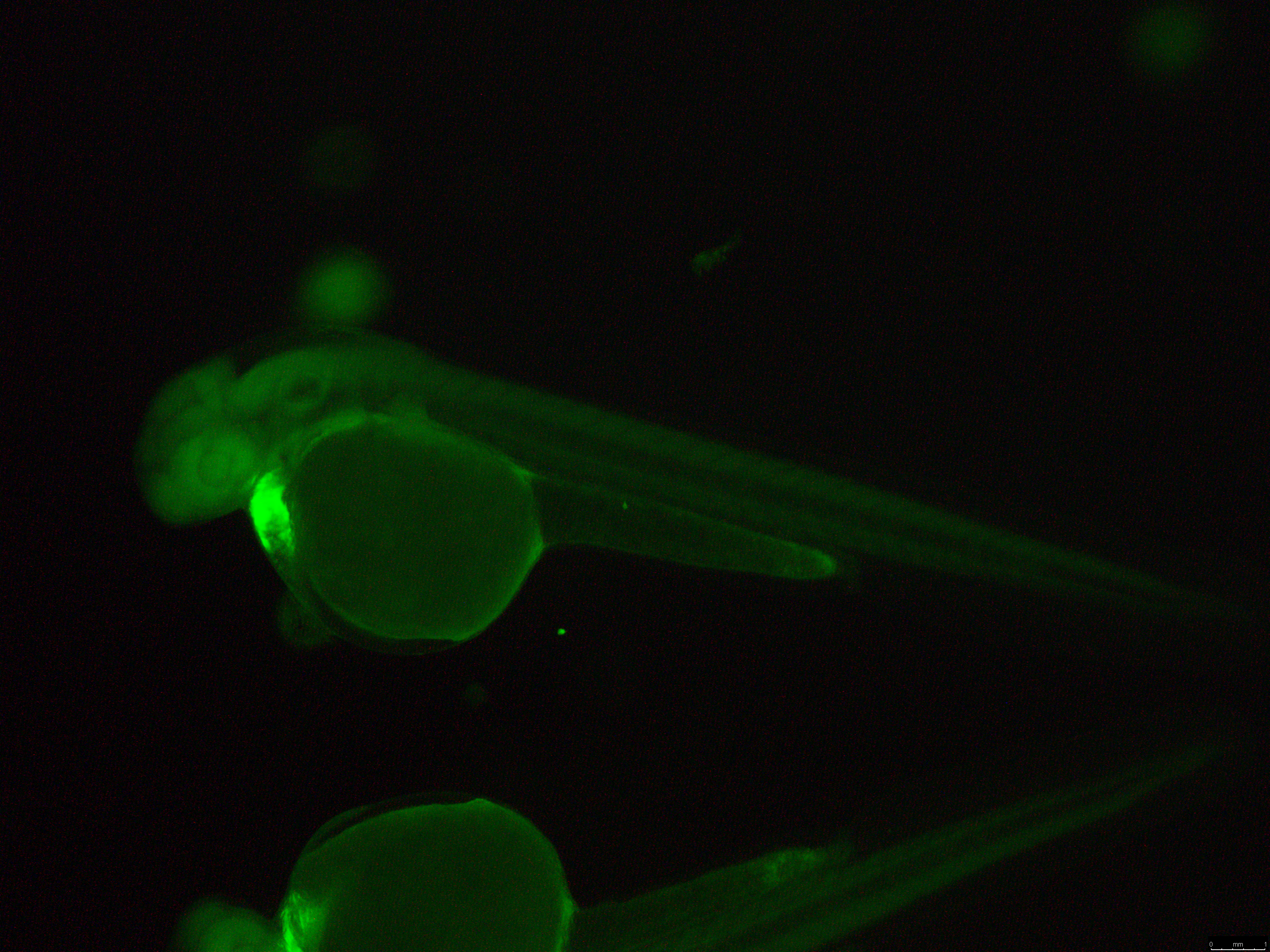

Supplement: Supplementary file 13 — Appendix Figure 5-7 Source Data [file 44319_2026_805_MOESM13_ESM.zip › Appendix Source Data 3/Appendix Fig.5/F/6. EGFP 36hpf trmt61aD181AD181A.tif]

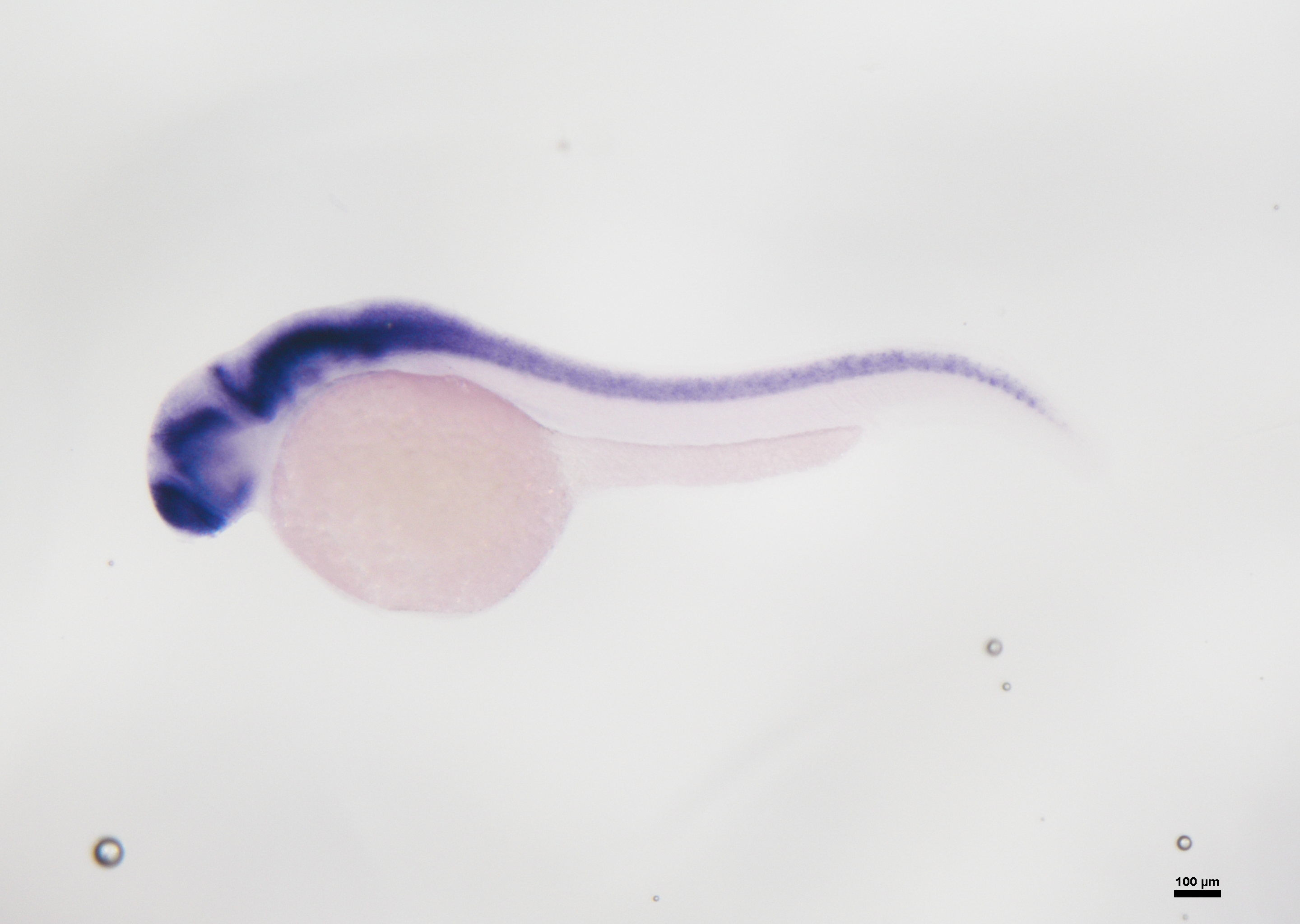

Supplement: Supplementary file 13 — Appendix Figure 5-7 Source Data [file 44319_2026_805_MOESM13_ESM.zip › Appendix Source Data 3/Appendix Fig.5/G/1. elavl3 36hpf WT.tif]

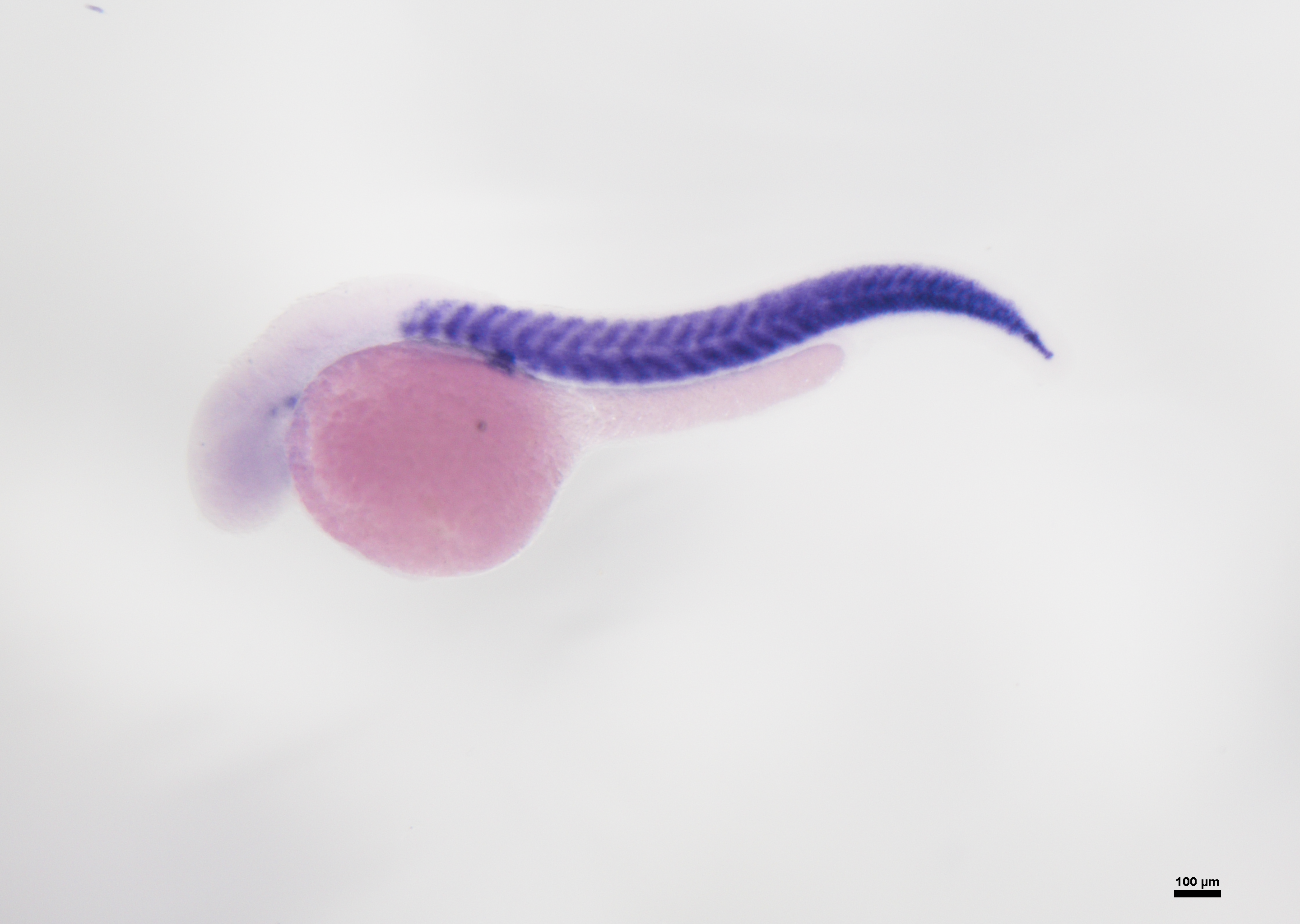

Supplement: Supplementary file 13 — Appendix Figure 5-7 Source Data [file 44319_2026_805_MOESM13_ESM.zip › Appendix Source Data 3/Appendix Fig.5/G/10. myod1 36hpf trmt61aD181AD181A.tif]

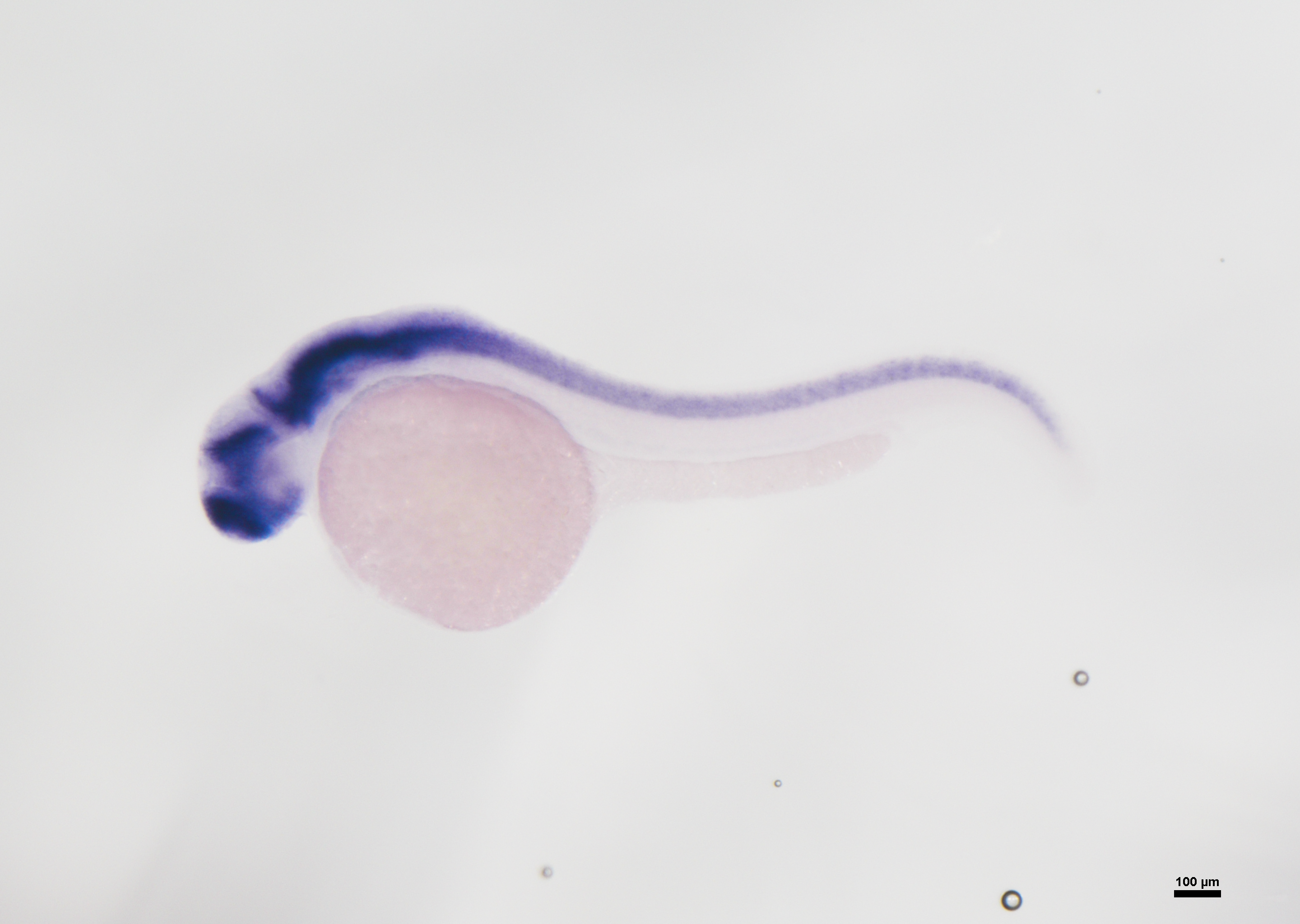

Supplement: Supplementary file 13 — Appendix Figure 5-7 Source Data [file 44319_2026_805_MOESM13_ESM.zip › Appendix Source Data 3/Appendix Fig.5/G/2. elavl3 36hpf trmt61aD181AD181A.tif]

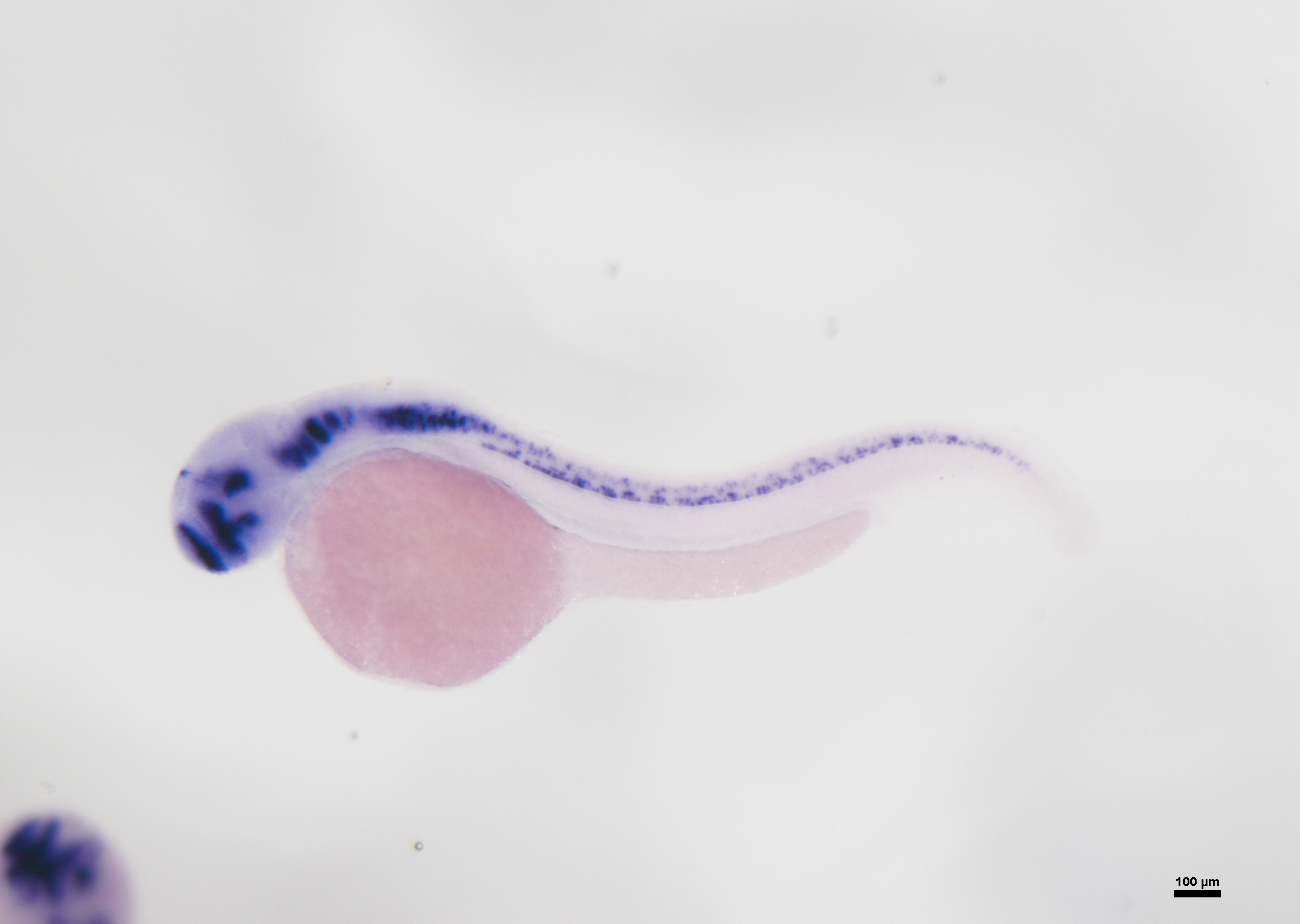

Supplement: Supplementary file 13 — Appendix Figure 5-7 Source Data [file 44319_2026_805_MOESM13_ESM.zip › Appendix Source Data 3/Appendix Fig.5/G/3. gad1b 36hpf WT.tif]

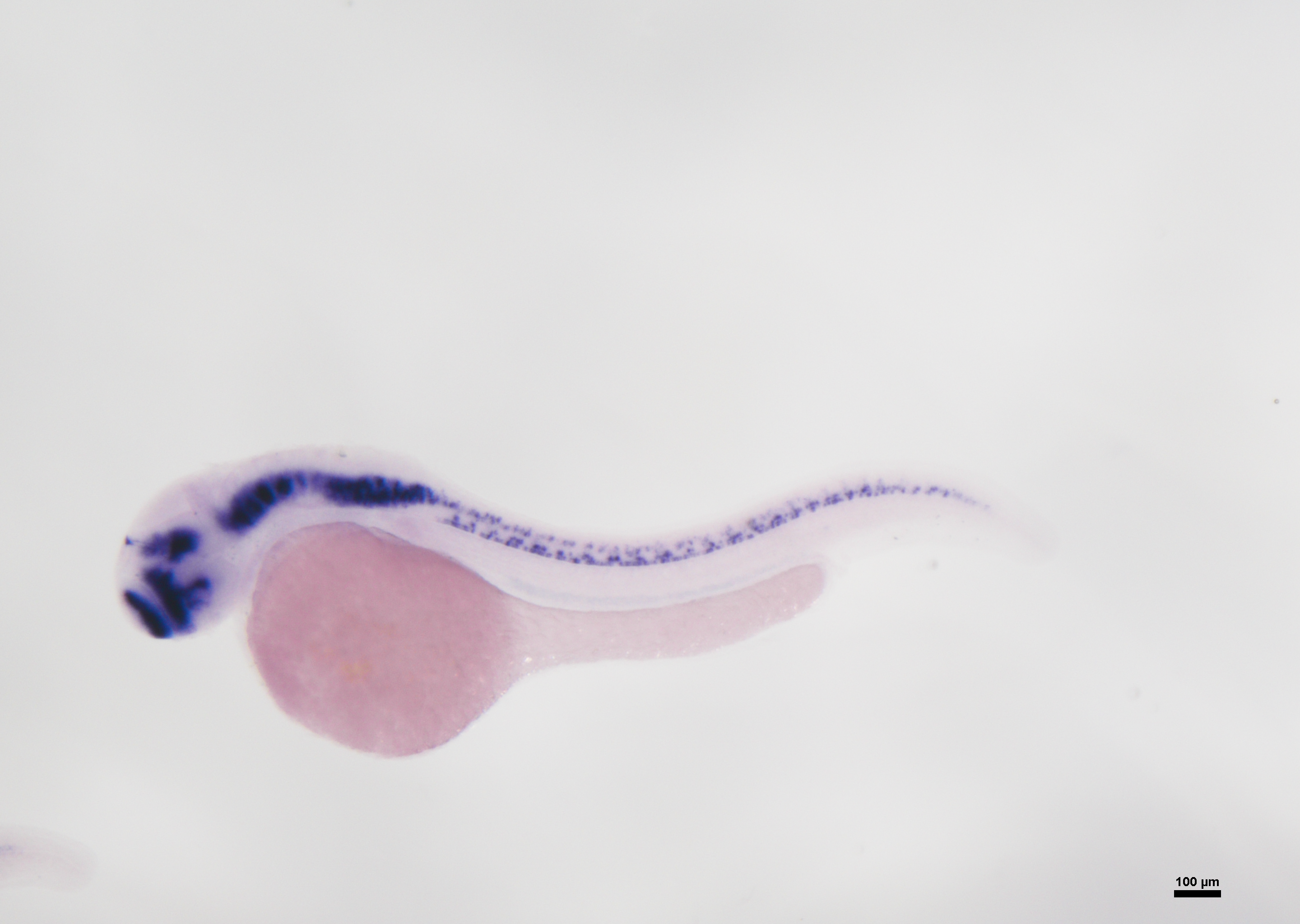

Supplement: Supplementary file 13 — Appendix Figure 5-7 Source Data [file 44319_2026_805_MOESM13_ESM.zip › Appendix Source Data 3/Appendix Fig.5/G/4. gad1b 36hpf trmt61aD181AD181A.tif]

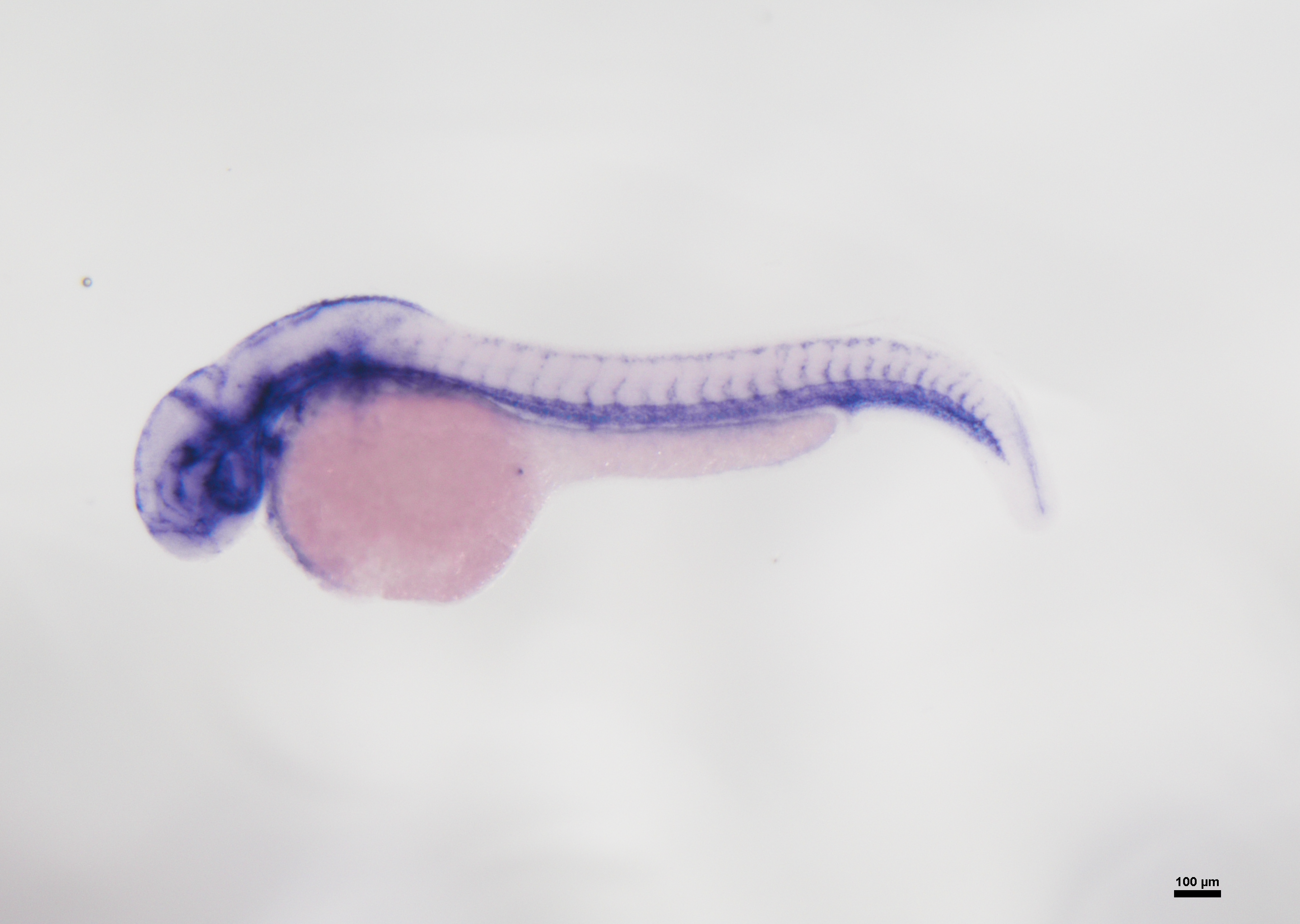

Supplement: Supplementary file 13 — Appendix Figure 5-7 Source Data [file 44319_2026_805_MOESM13_ESM.zip › Appendix Source Data 3/Appendix Fig.5/G/5. kdrl 36hpf WT.tif]

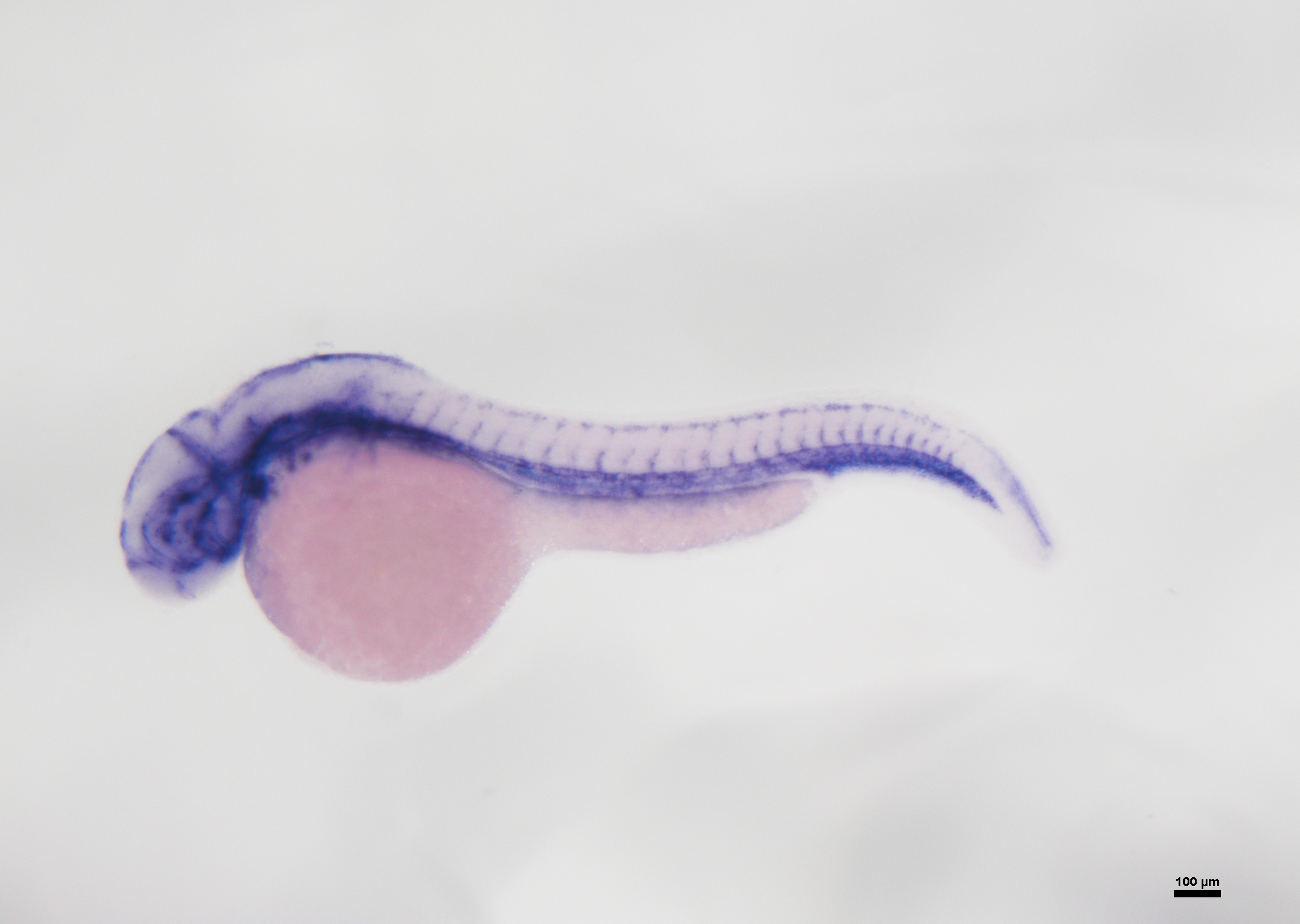

Supplement: Supplementary file 13 — Appendix Figure 5-7 Source Data [file 44319_2026_805_MOESM13_ESM.zip › Appendix Source Data 3/Appendix Fig.5/G/6. kdrl 36hpf trmt61aD181AD181A.tif]

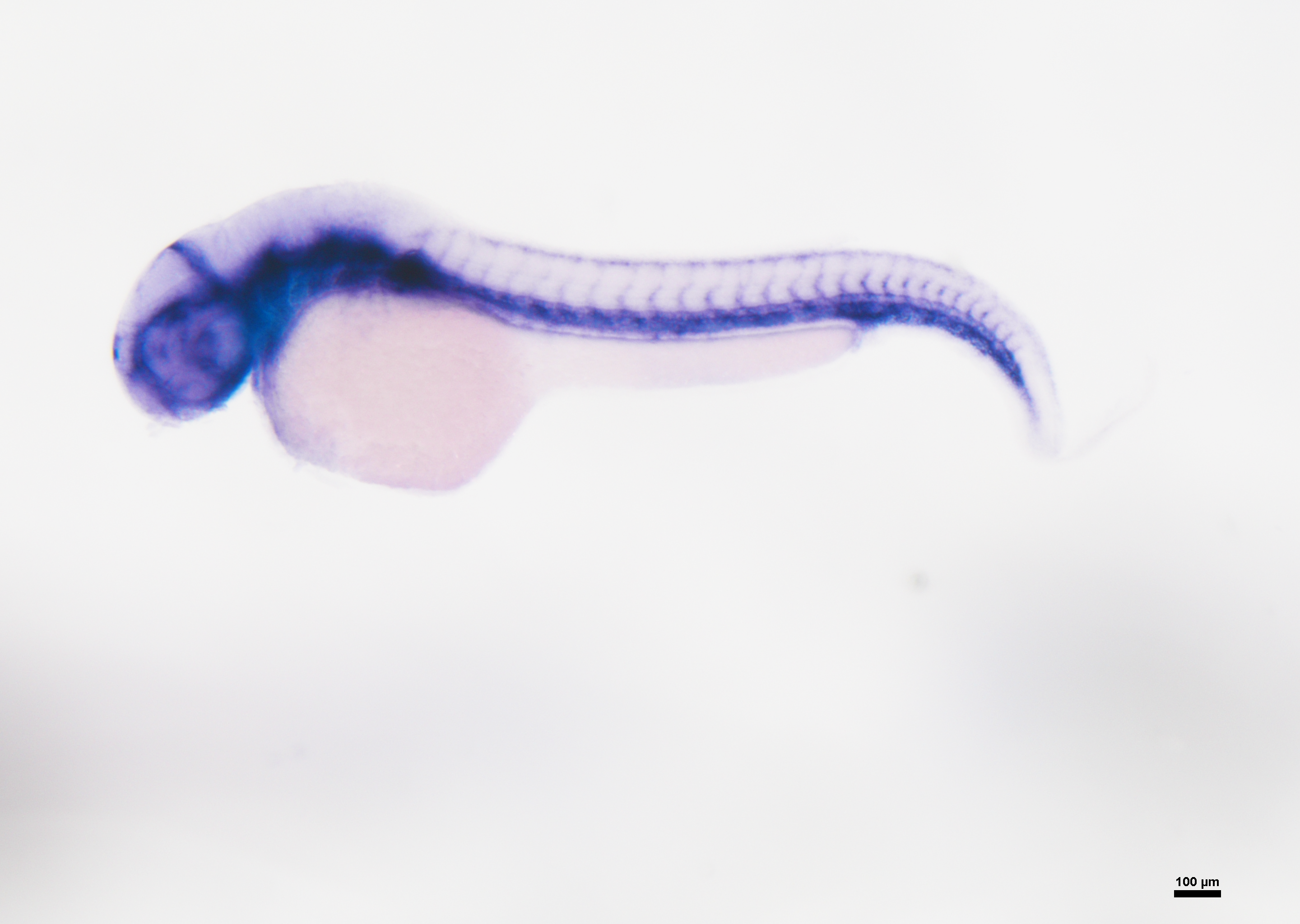

Supplement: Supplementary file 13 — Appendix Figure 5-7 Source Data [file 44319_2026_805_MOESM13_ESM.zip › Appendix Source Data 3/Appendix Fig.5/G/7. fli1a 36hpf WT.tif]

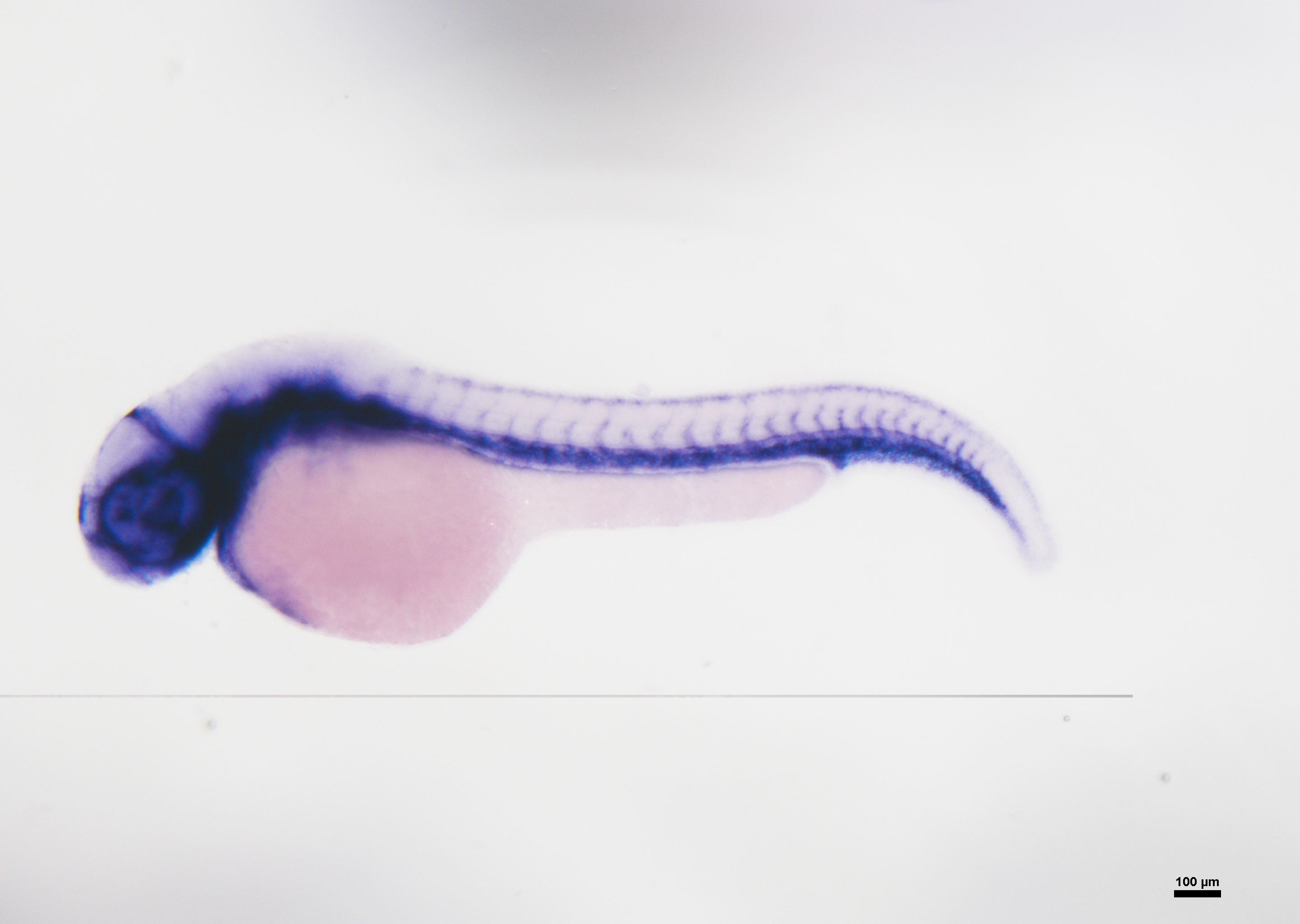

Supplement: Supplementary file 13 — Appendix Figure 5-7 Source Data [file 44319_2026_805_MOESM13_ESM.zip › Appendix Source Data 3/Appendix Fig.5/G/8. fli1a 36hpf trmt61aD181AD181A.tif]

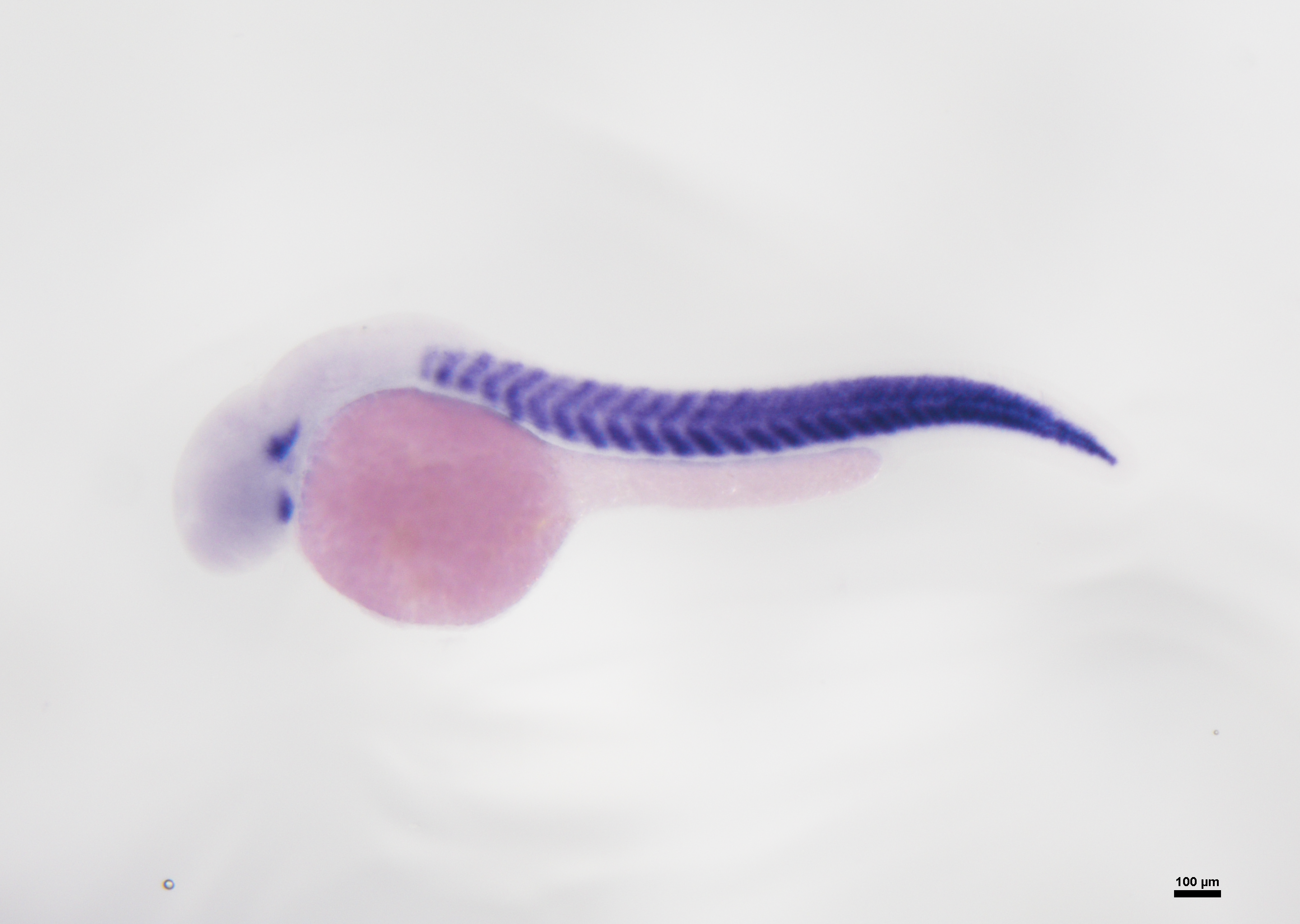

Supplement: Supplementary file 13 — Appendix Figure 5-7 Source Data [file 44319_2026_805_MOESM13_ESM.zip › Appendix Source Data 3/Appendix Fig.5/G/9. moyd1 36hpf WT.tif]

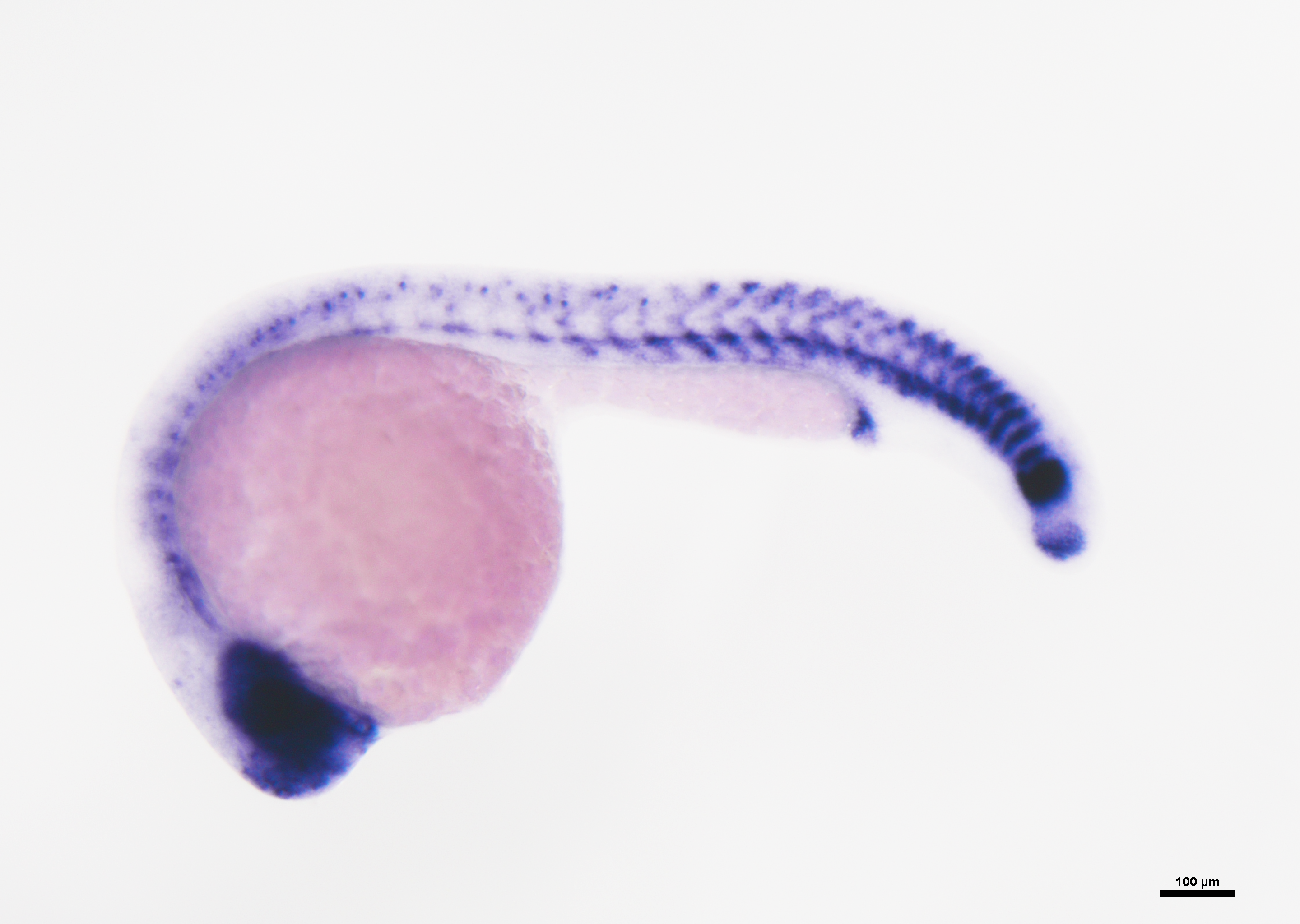

Supplement: Supplementary file 13 — Appendix Figure 5-7 Source Data [file 44319_2026_805_MOESM13_ESM.zip › Appendix Source Data 3/Appendix Fig.5/H/1. dltc 22hpf WT.tif]

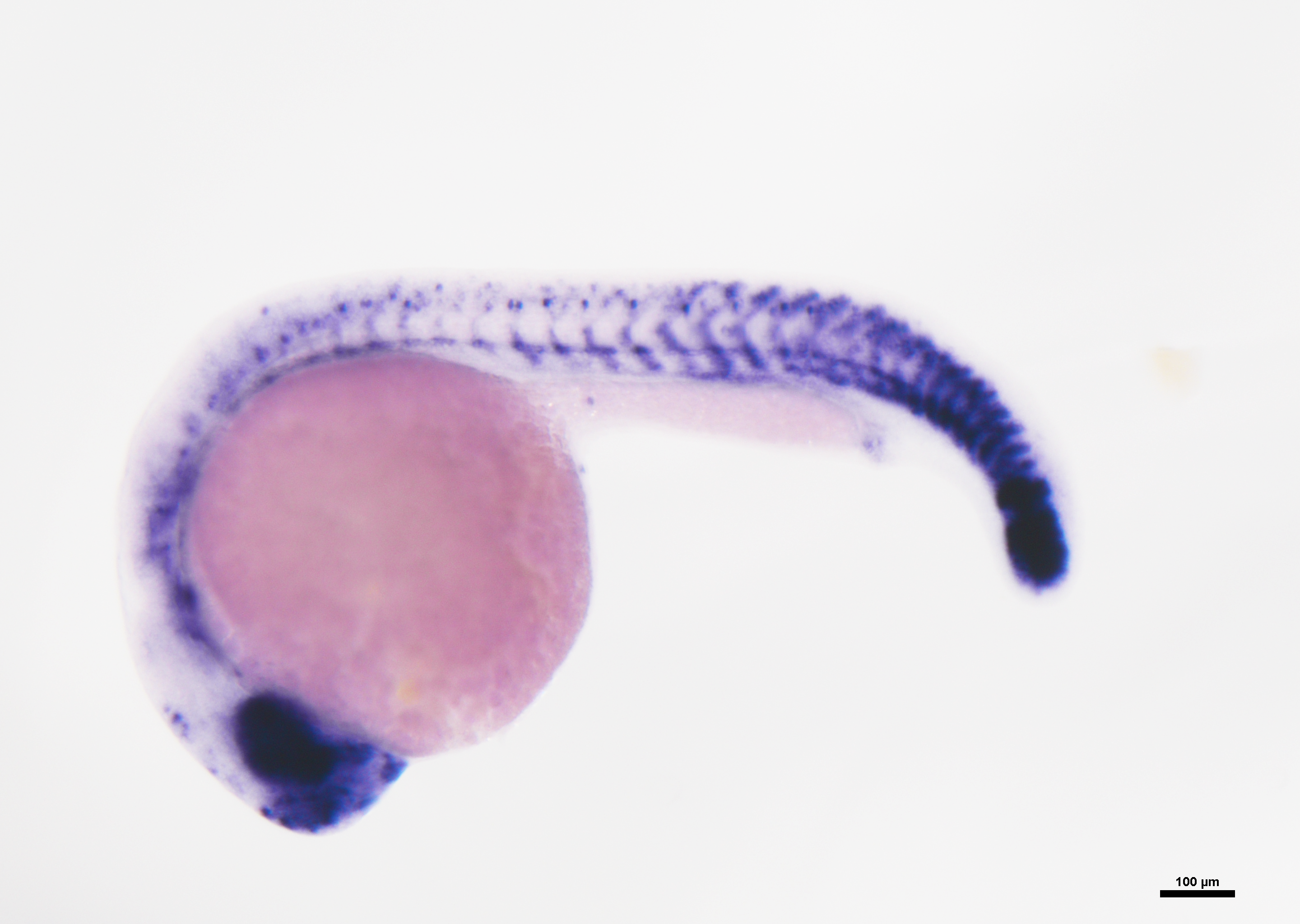

Supplement: Supplementary file 13 — Appendix Figure 5-7 Source Data [file 44319_2026_805_MOESM13_ESM.zip › Appendix Source Data 3/Appendix Fig.5/H/2. dltc 22hpf trmt61aD181AD181A.tif]

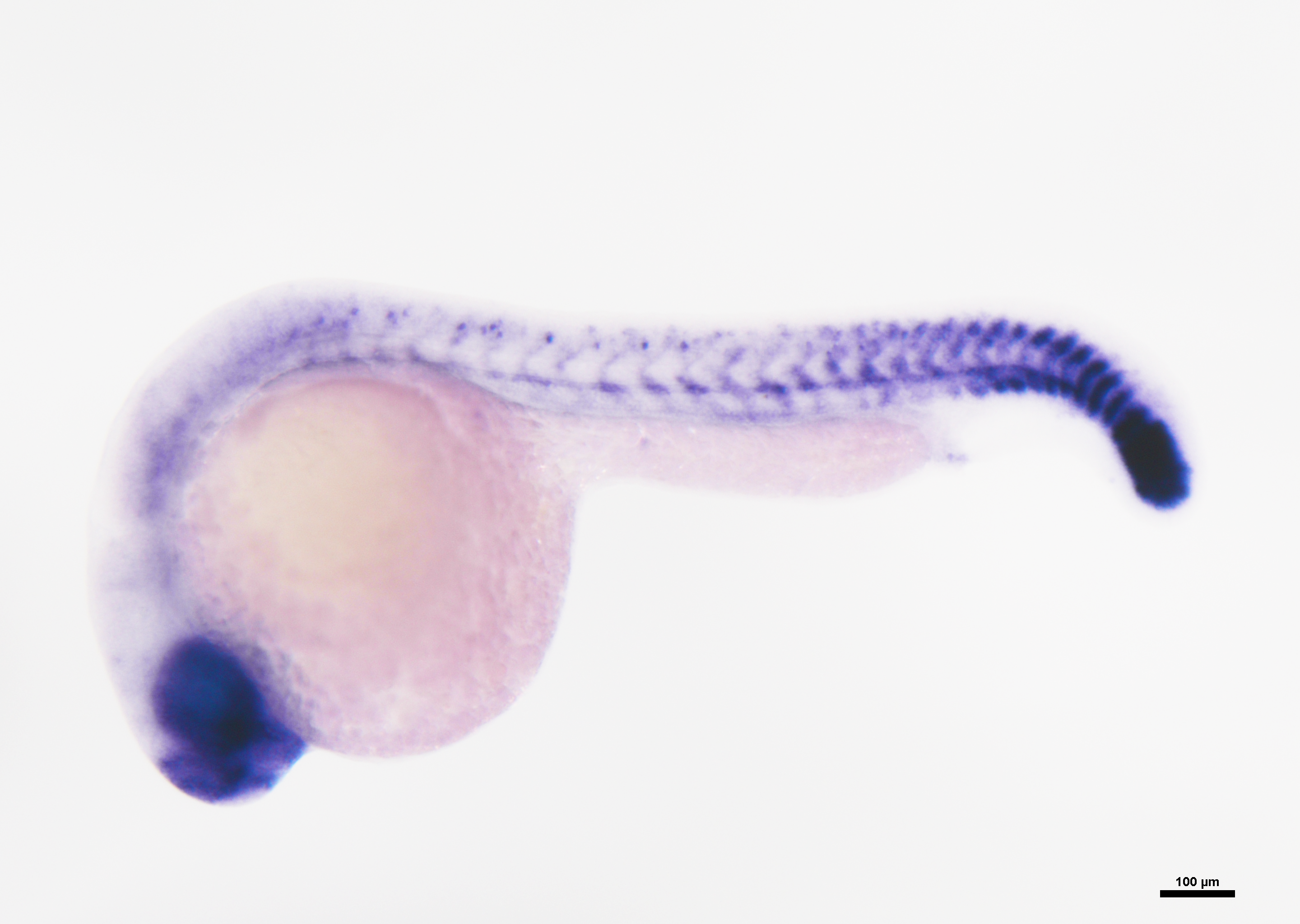

Supplement: Supplementary file 13 — Appendix Figure 5-7 Source Data [file 44319_2026_805_MOESM13_ESM.zip › Appendix Source Data 3/Appendix Fig.5/H/3. dltc 24hpf WT.tif]

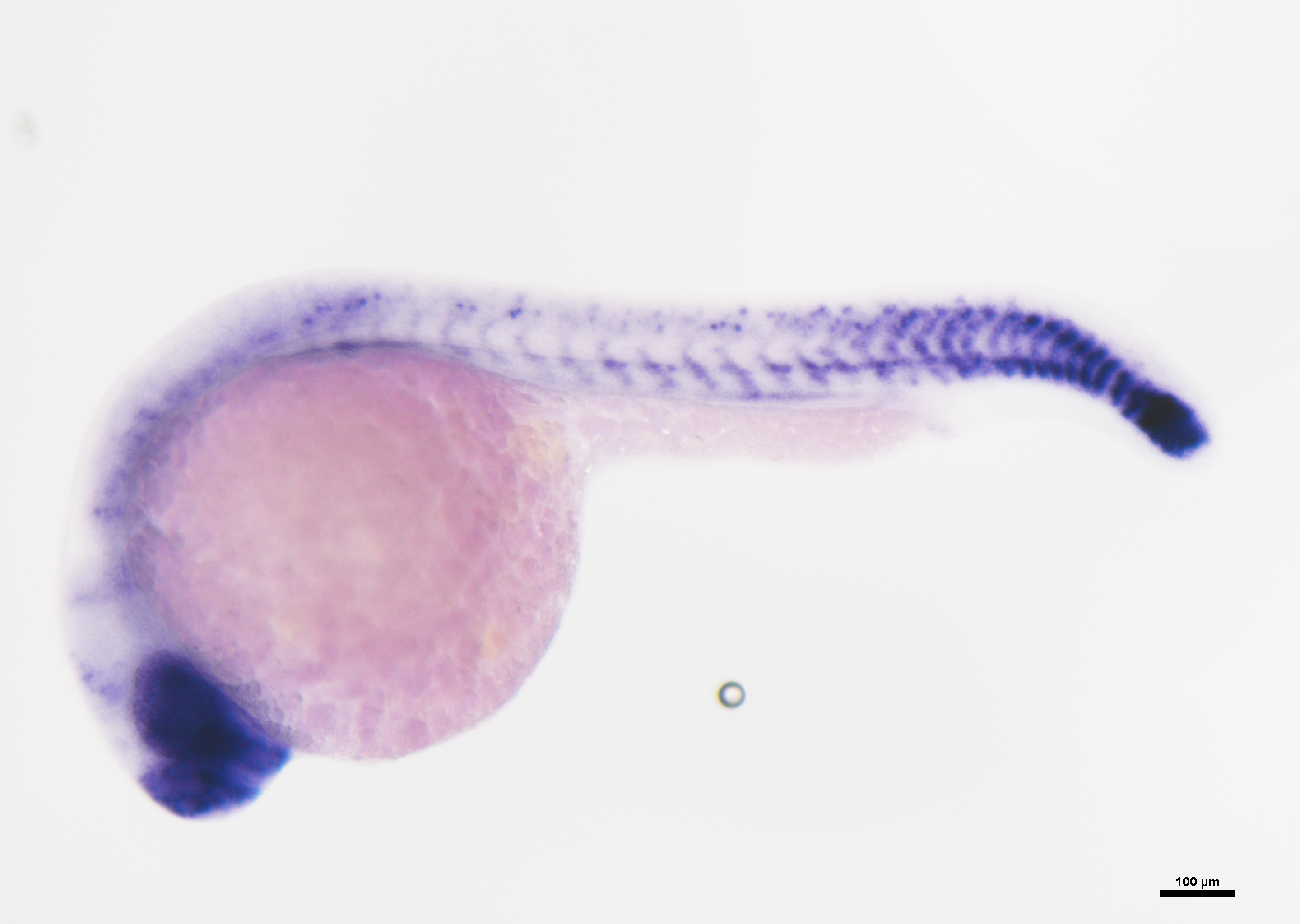

Supplement: Supplementary file 13 — Appendix Figure 5-7 Source Data [file 44319_2026_805_MOESM13_ESM.zip › Appendix Source Data 3/Appendix Fig.5/H/4. dltc 24hpf trmt61aD181AD181A.tif]

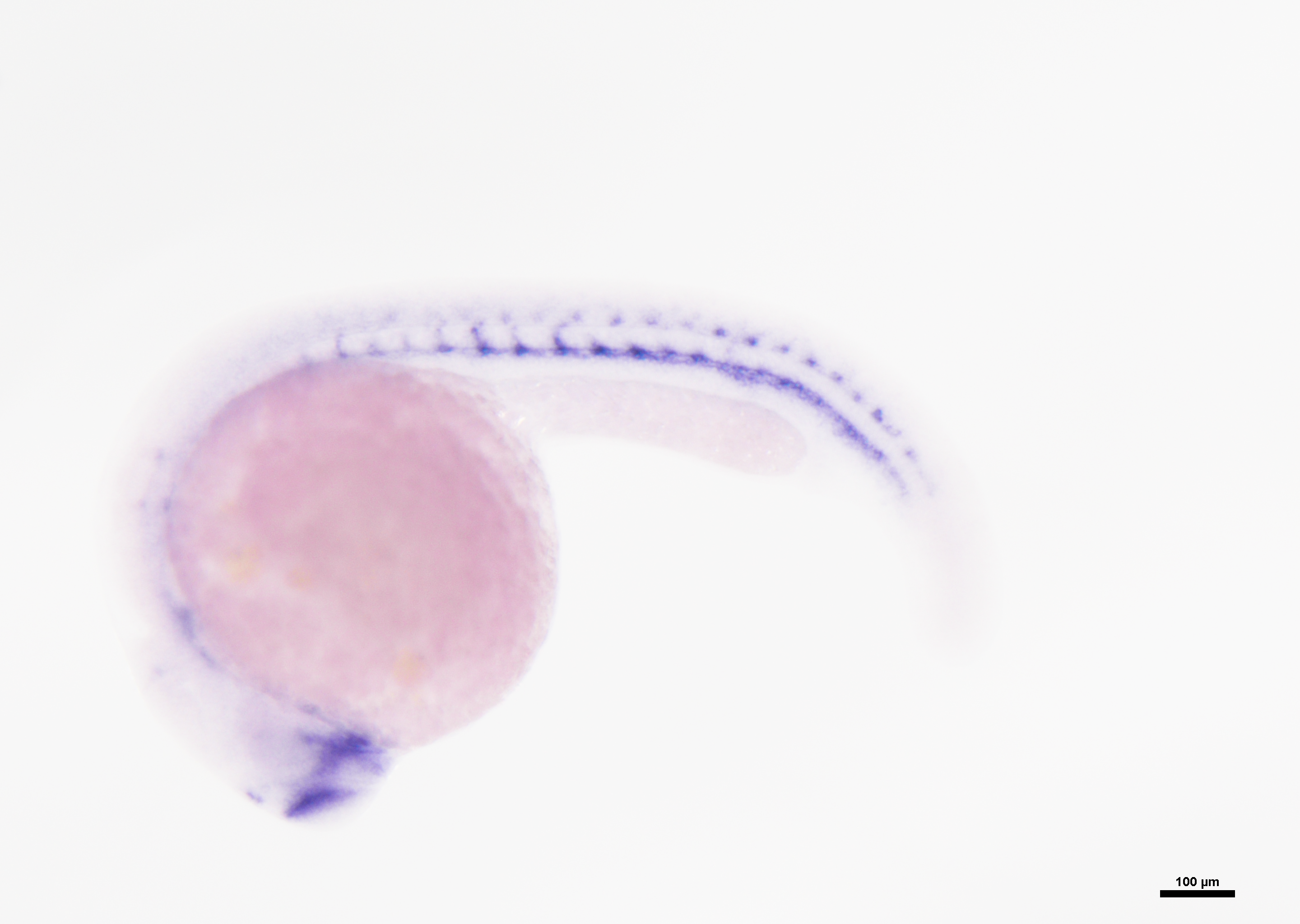

Supplement: Supplementary file 13 — Appendix Figure 5-7 Source Data [file 44319_2026_805_MOESM13_ESM.zip › Appendix Source Data 3/Appendix Fig.5/H/5. dll4 22hpf WT.tif]

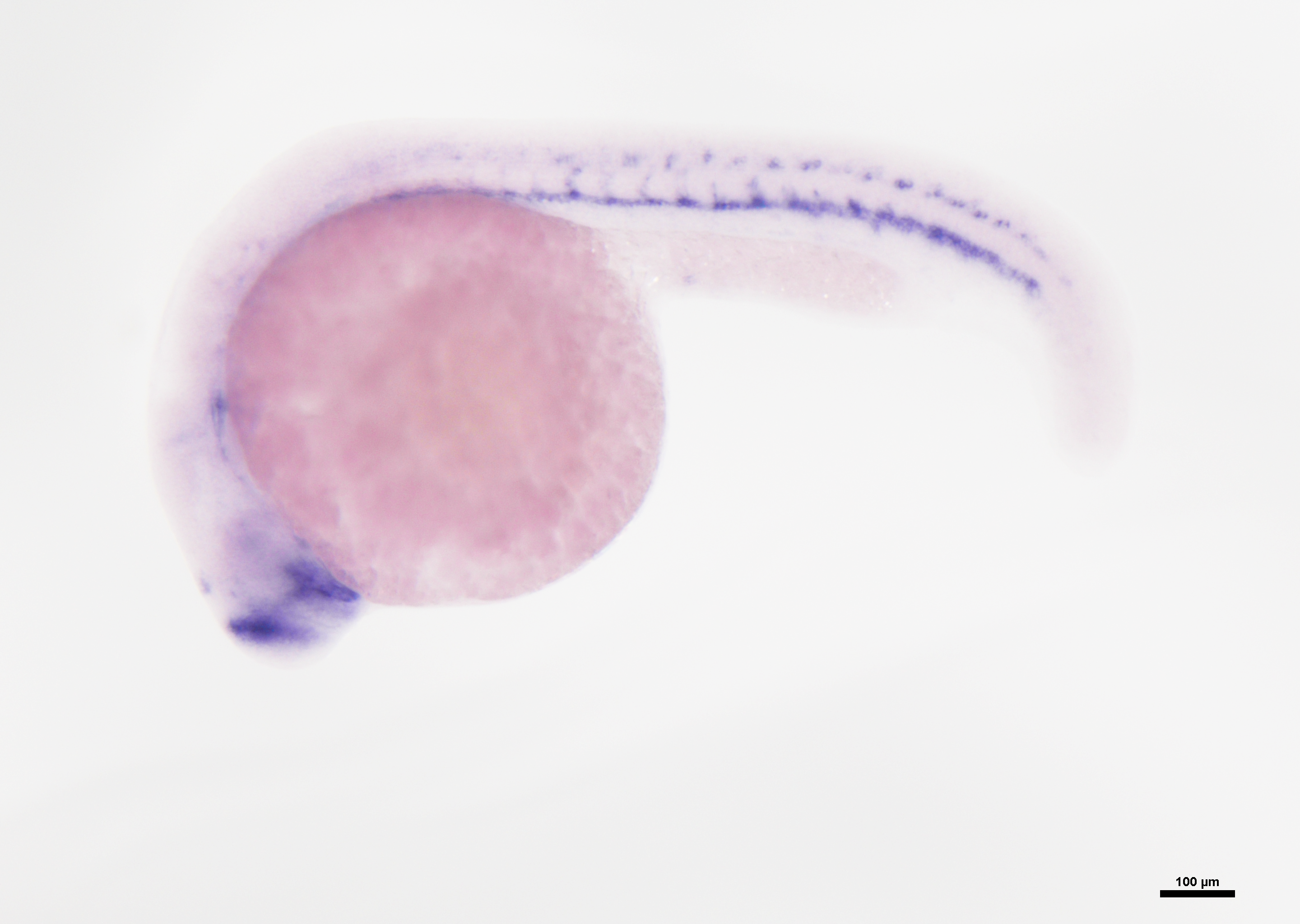

Supplement: Supplementary file 13 — Appendix Figure 5-7 Source Data [file 44319_2026_805_MOESM13_ESM.zip › Appendix Source Data 3/Appendix Fig.5/H/6. dll4 22hpf trmt61aD181AD181A.tif]

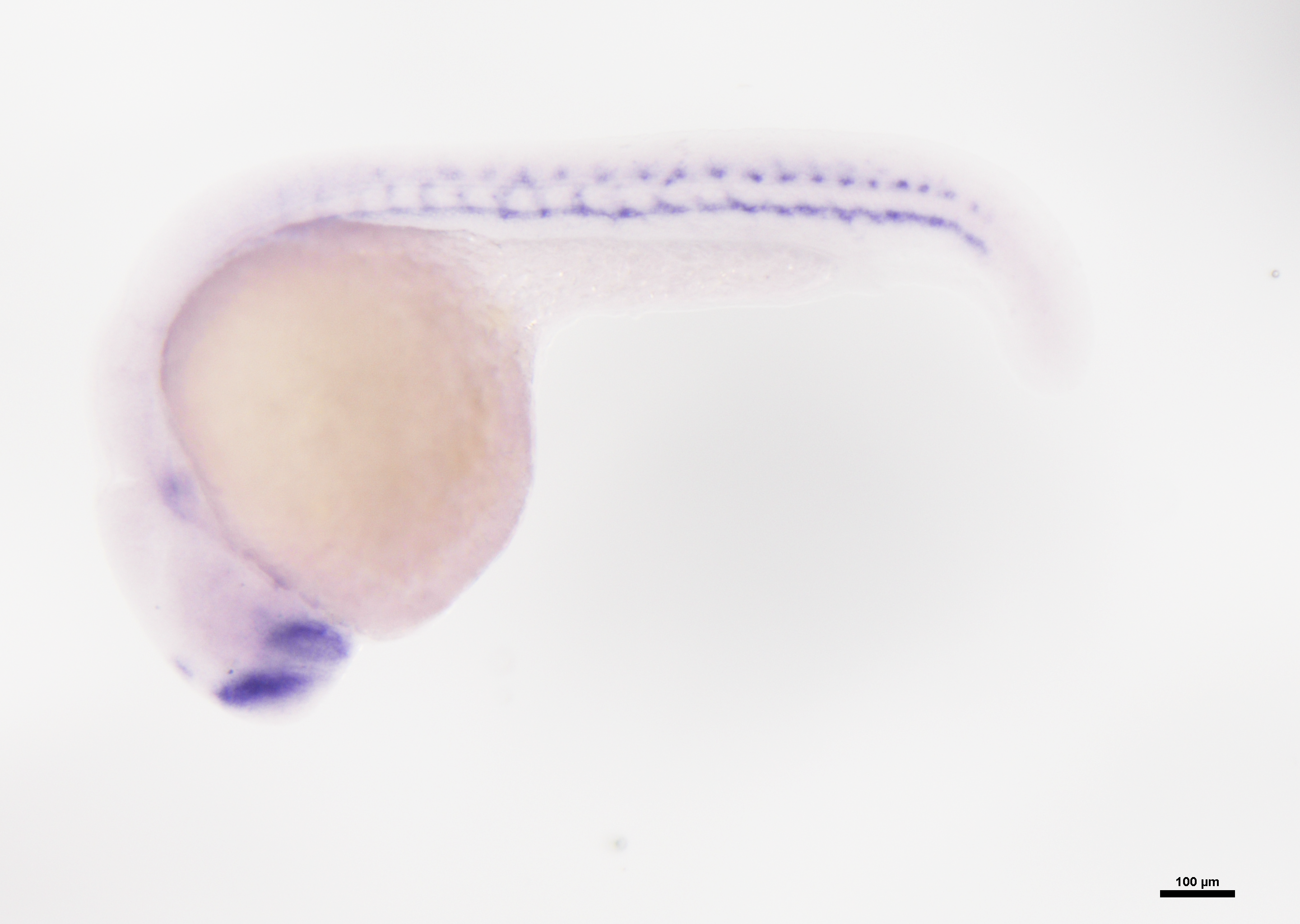

Supplement: Supplementary file 13 — Appendix Figure 5-7 Source Data [file 44319_2026_805_MOESM13_ESM.zip › Appendix Source Data 3/Appendix Fig.5/H/7. dll4 24hpf WT.tif]

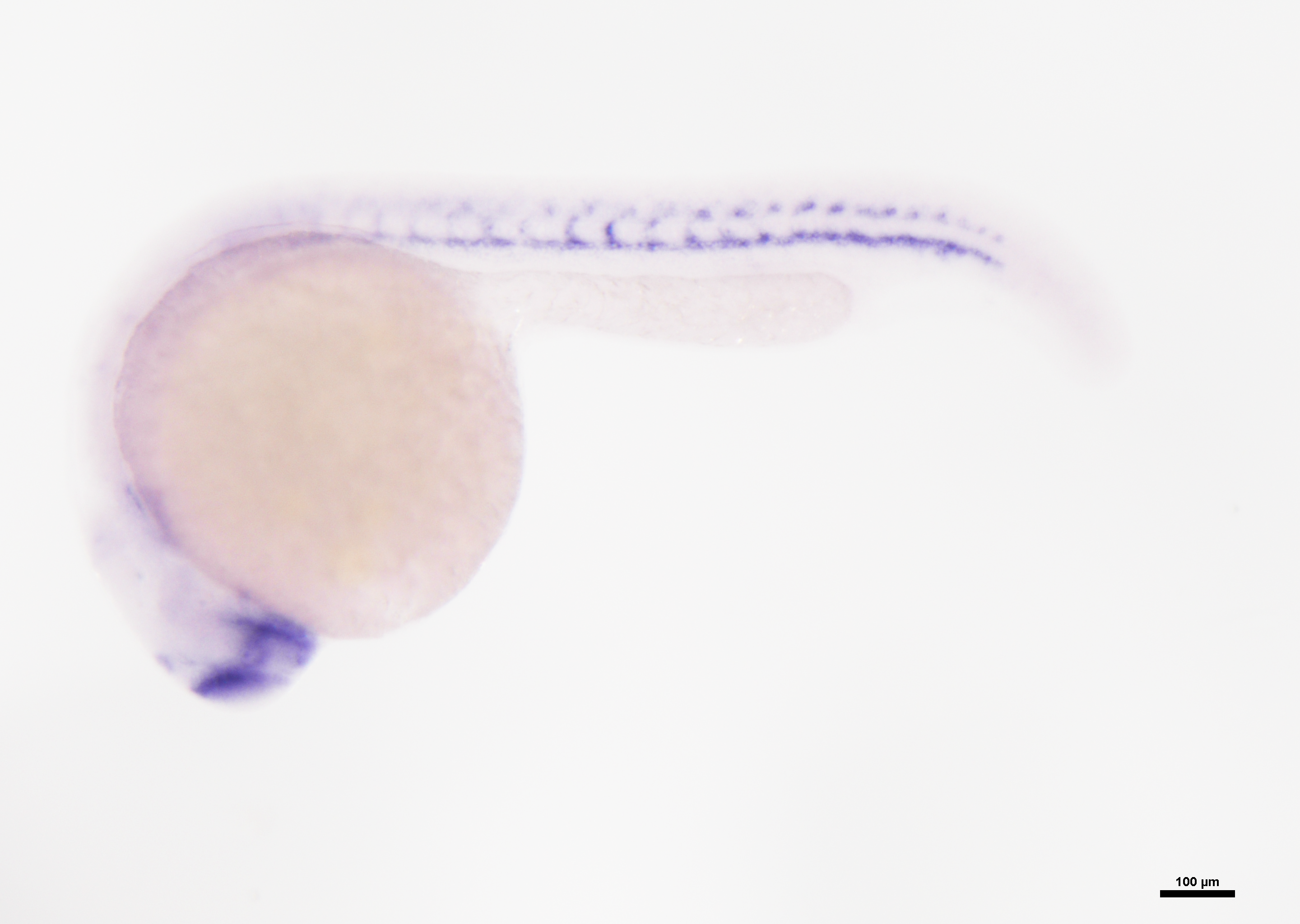

Supplement: Supplementary file 13 — Appendix Figure 5-7 Source Data [file 44319_2026_805_MOESM13_ESM.zip › Appendix Source Data 3/Appendix Fig.5/H/8. dll4 24hpf trmt61aD181AD181A.tif]

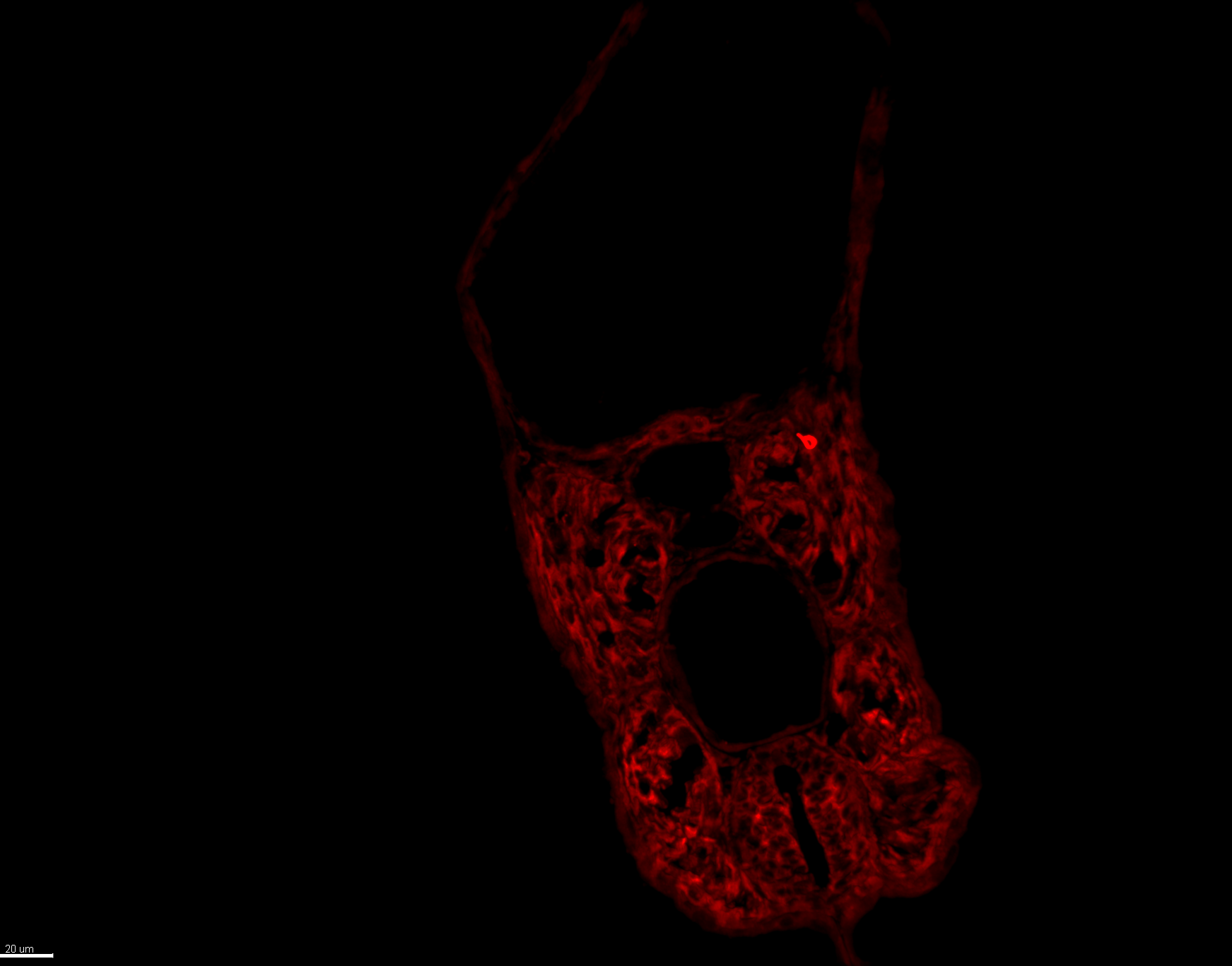

Supplement: Supplementary file 13 — Appendix Figure 5-7 Source Data [file 44319_2026_805_MOESM13_ESM.zip › Appendix Source Data 3/Appendix Fig.5/J/1. anti-m1a 36hpf WT.tif]

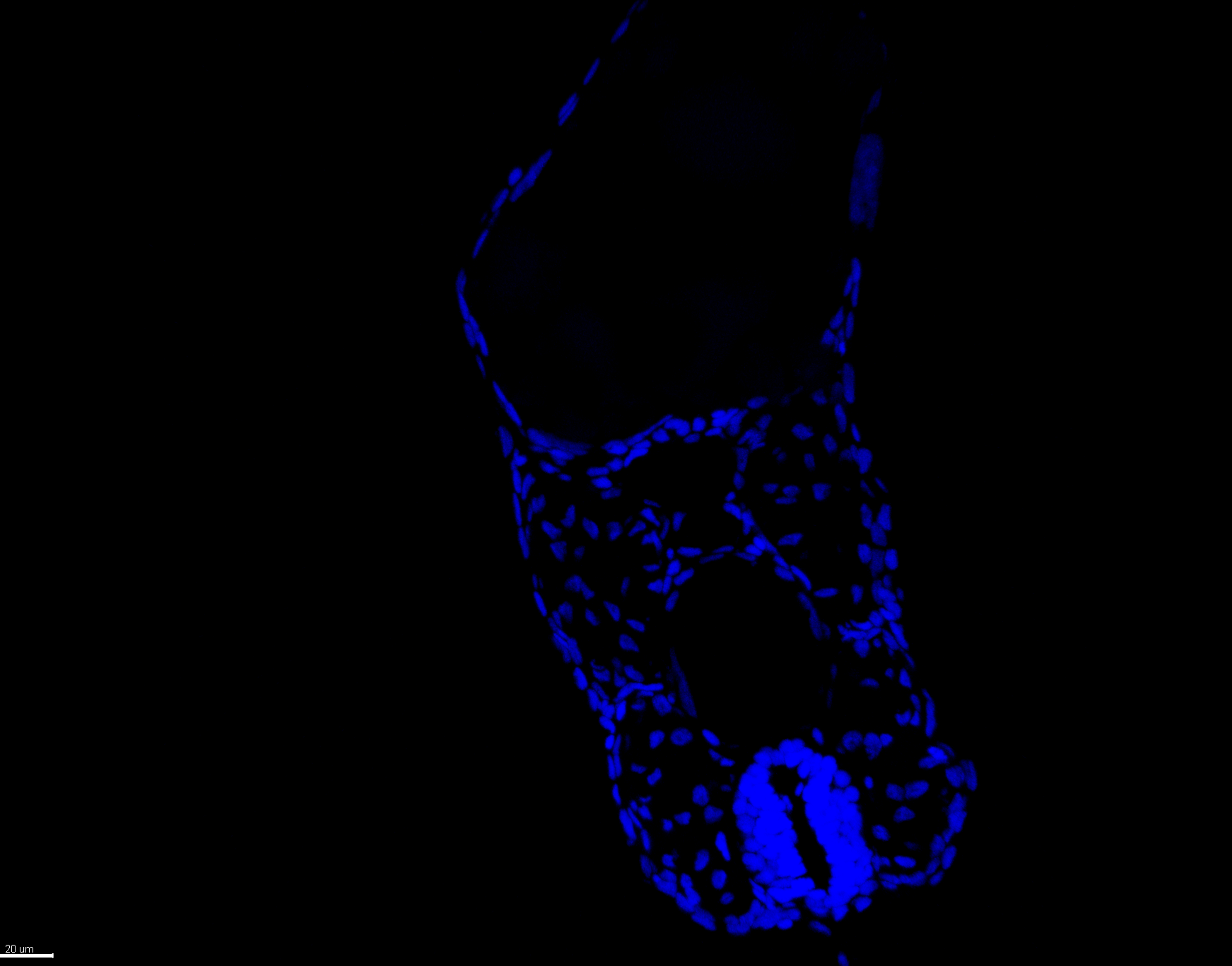

Supplement: Supplementary file 13 — Appendix Figure 5-7 Source Data [file 44319_2026_805_MOESM13_ESM.zip › Appendix Source Data 3/Appendix Fig.5/J/2. dapi 36hpf WT.tif]

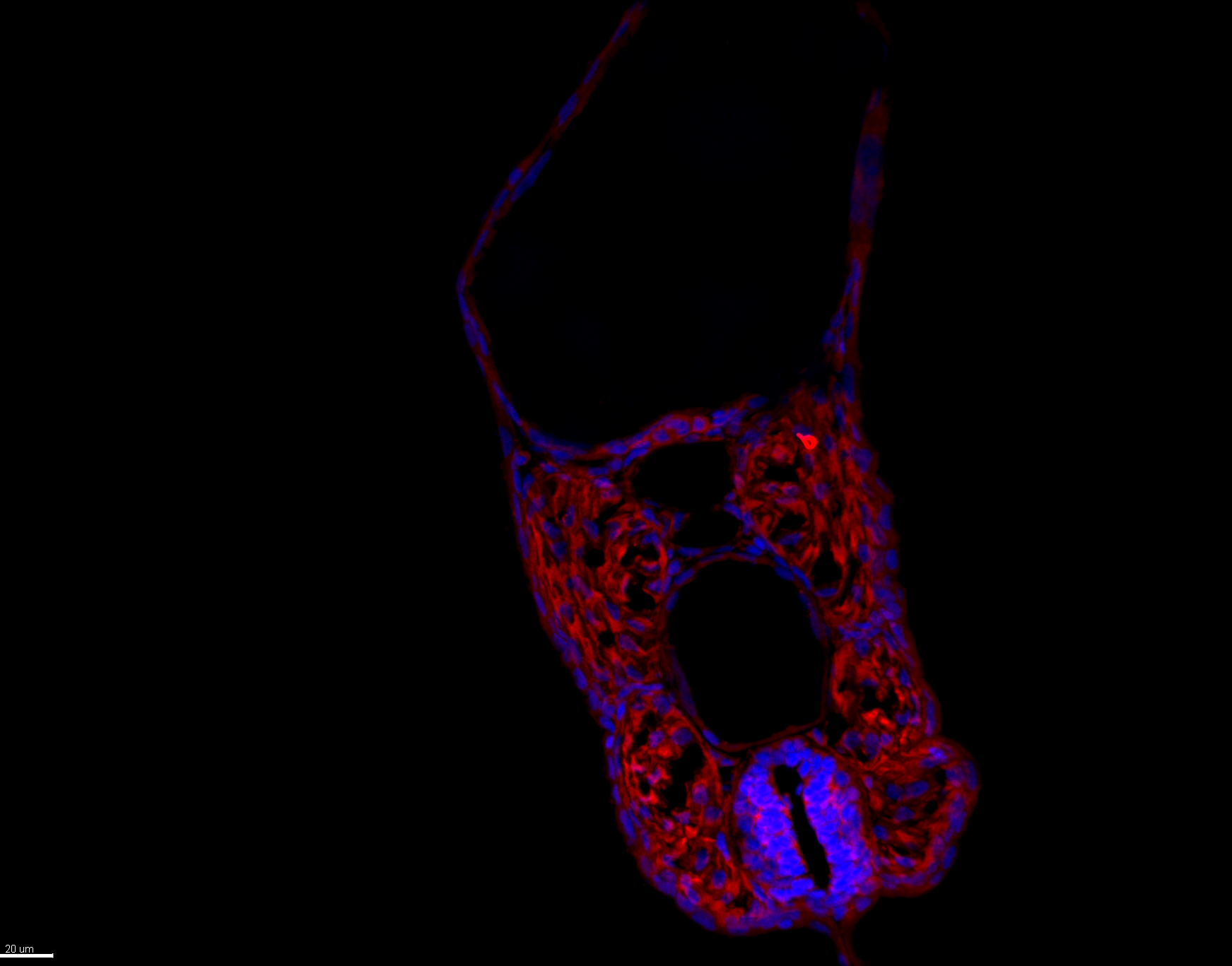

Supplement: Supplementary file 13 — Appendix Figure 5-7 Source Data [file 44319_2026_805_MOESM13_ESM.zip › Appendix Source Data 3/Appendix Fig.5/J/3. merge 36hpf WT.tif]

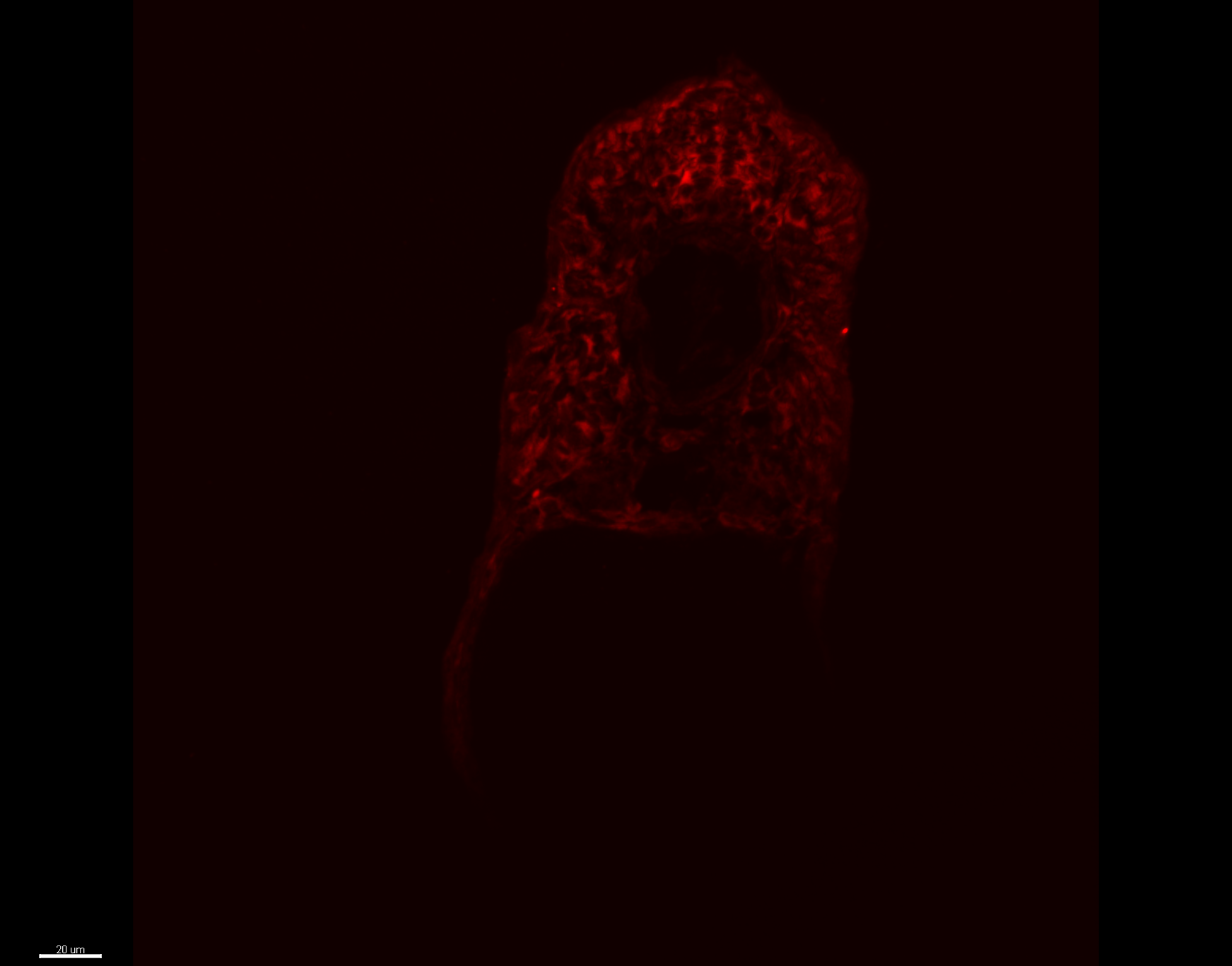

Supplement: Supplementary file 13 — Appendix Figure 5-7 Source Data [file 44319_2026_805_MOESM13_ESM.zip › Appendix Source Data 3/Appendix Fig.5/J/4. anti-m1a 36hpf Mtrmt61a;trmt61aD181A.tif]

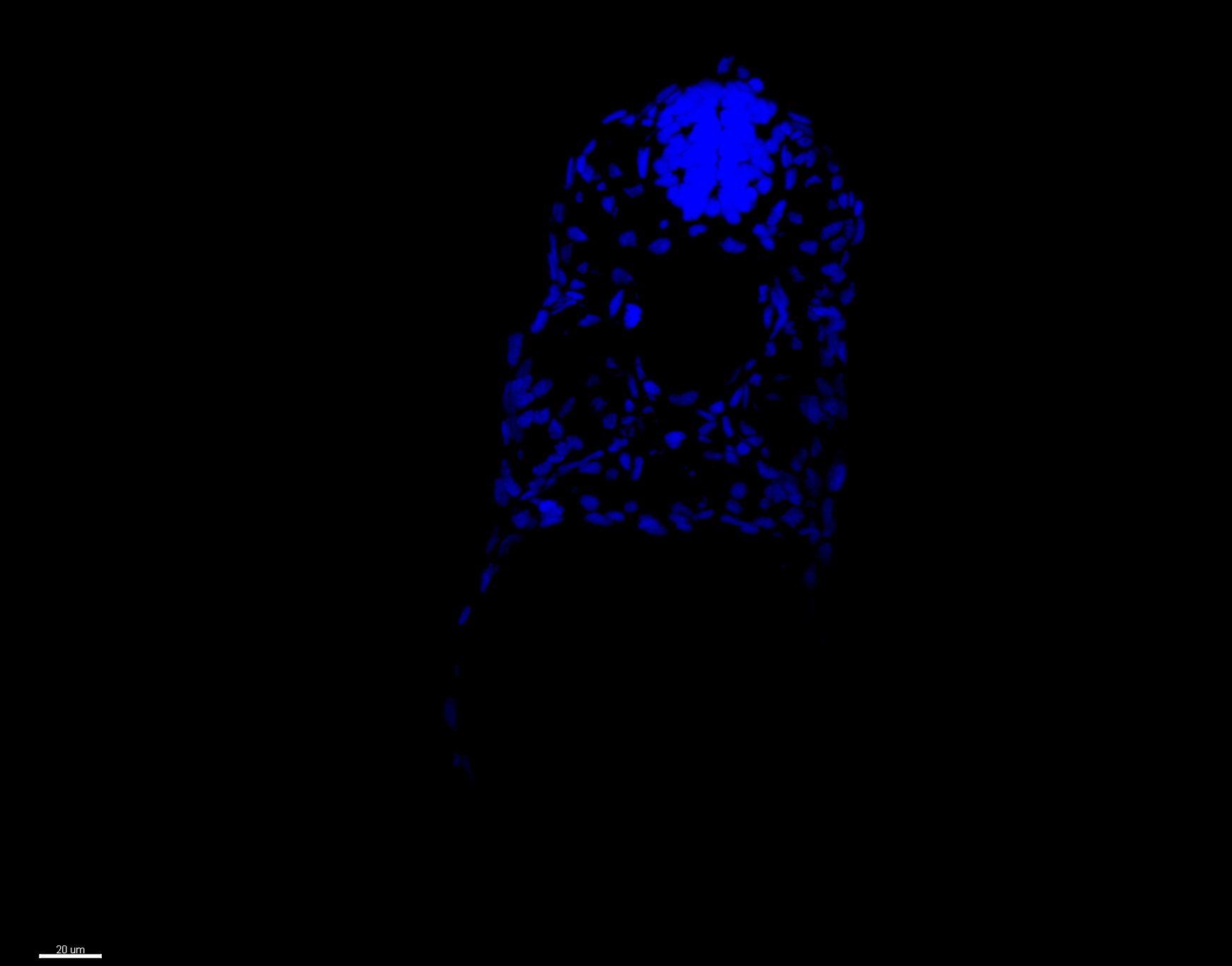

Supplement: Supplementary file 13 — Appendix Figure 5-7 Source Data [file 44319_2026_805_MOESM13_ESM.zip › Appendix Source Data 3/Appendix Fig.5/J/5. dapi 36hpf Mtrmt61a;trmt61aD181A.tif]

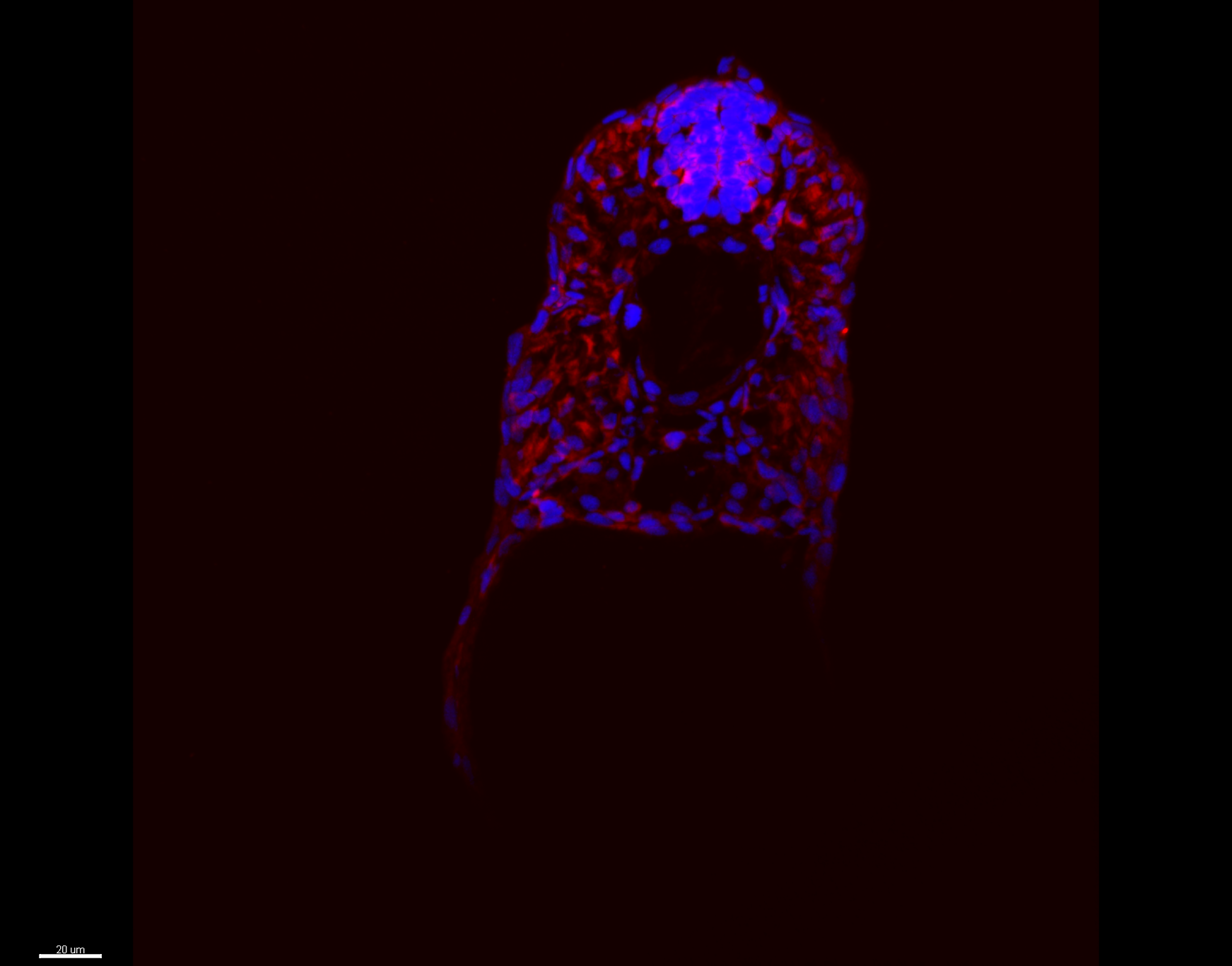

Supplement: Supplementary file 13 — Appendix Figure 5-7 Source Data [file 44319_2026_805_MOESM13_ESM.zip › Appendix Source Data 3/Appendix Fig.5/J/6. merge 36hpf Mtrmt61a;trmt61aD181A.tif]

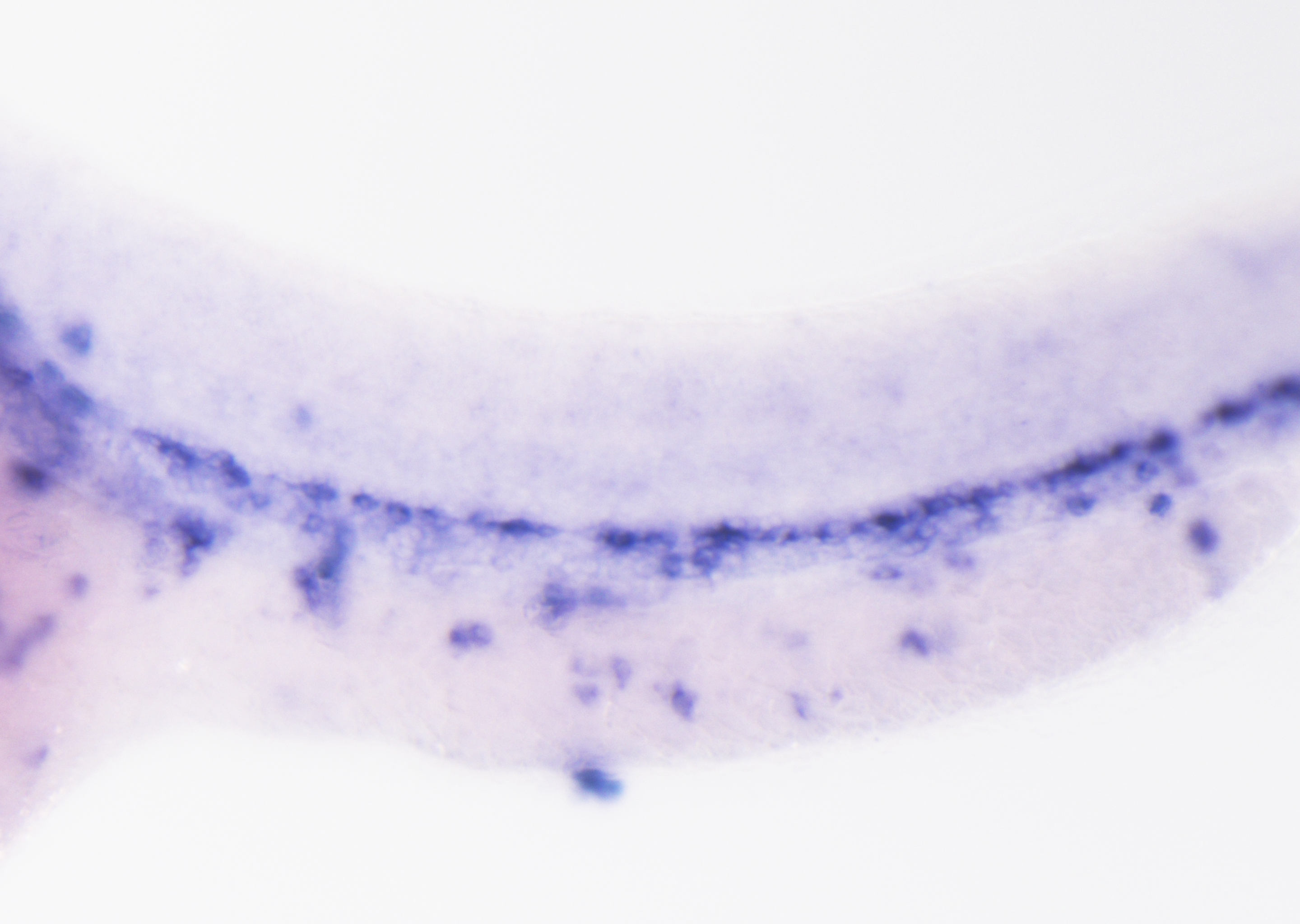

Supplement: Supplementary file 13 — Appendix Figure 5-7 Source Data [file 44319_2026_805_MOESM13_ESM.zip › Appendix Source Data 3/Appendix Fig.5/K/1. cmyb 36hpf sibling.tif]

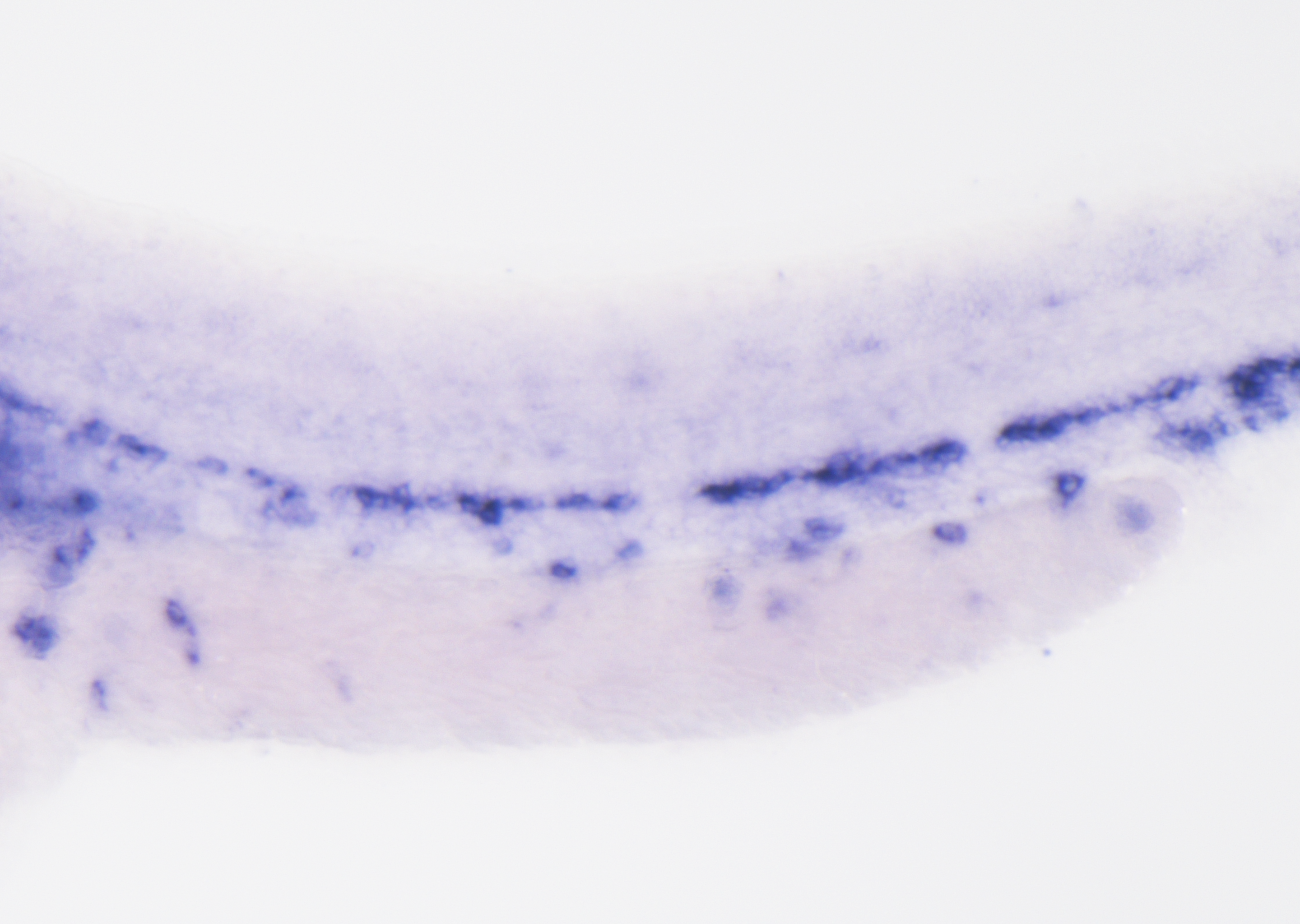

Supplement: Supplementary file 13 — Appendix Figure 5-7 Source Data [file 44319_2026_805_MOESM13_ESM.zip › Appendix Source Data 3/Appendix Fig.5/K/2. cmyb 36hpf Mtrmt61a.tif]

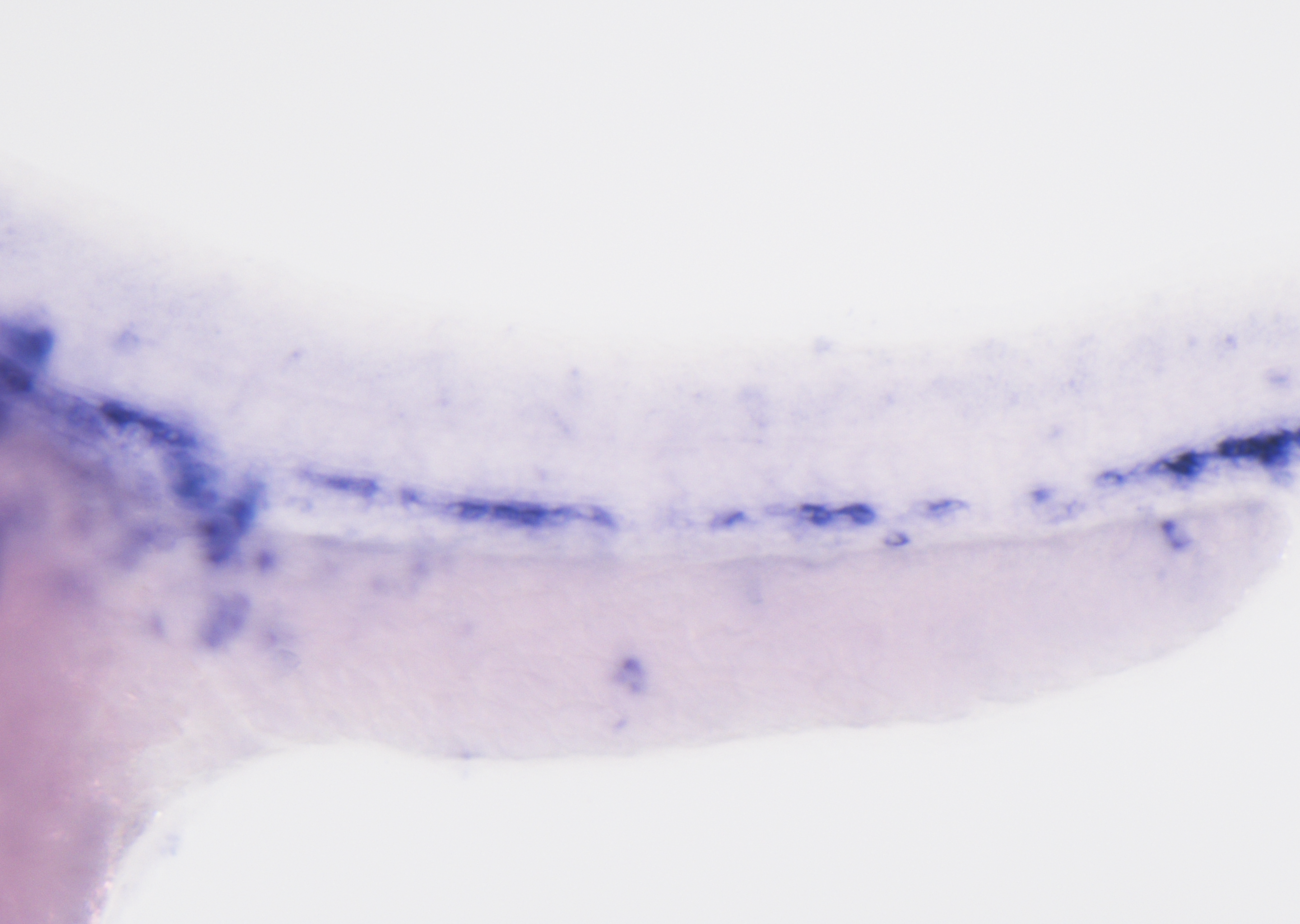

Supplement: Supplementary file 13 — Appendix Figure 5-7 Source Data [file 44319_2026_805_MOESM13_ESM.zip › Appendix Source Data 3/Appendix Fig.5/K/3. cmyb 36hpf Mtrmt61a;trmt71aD181A.tif]

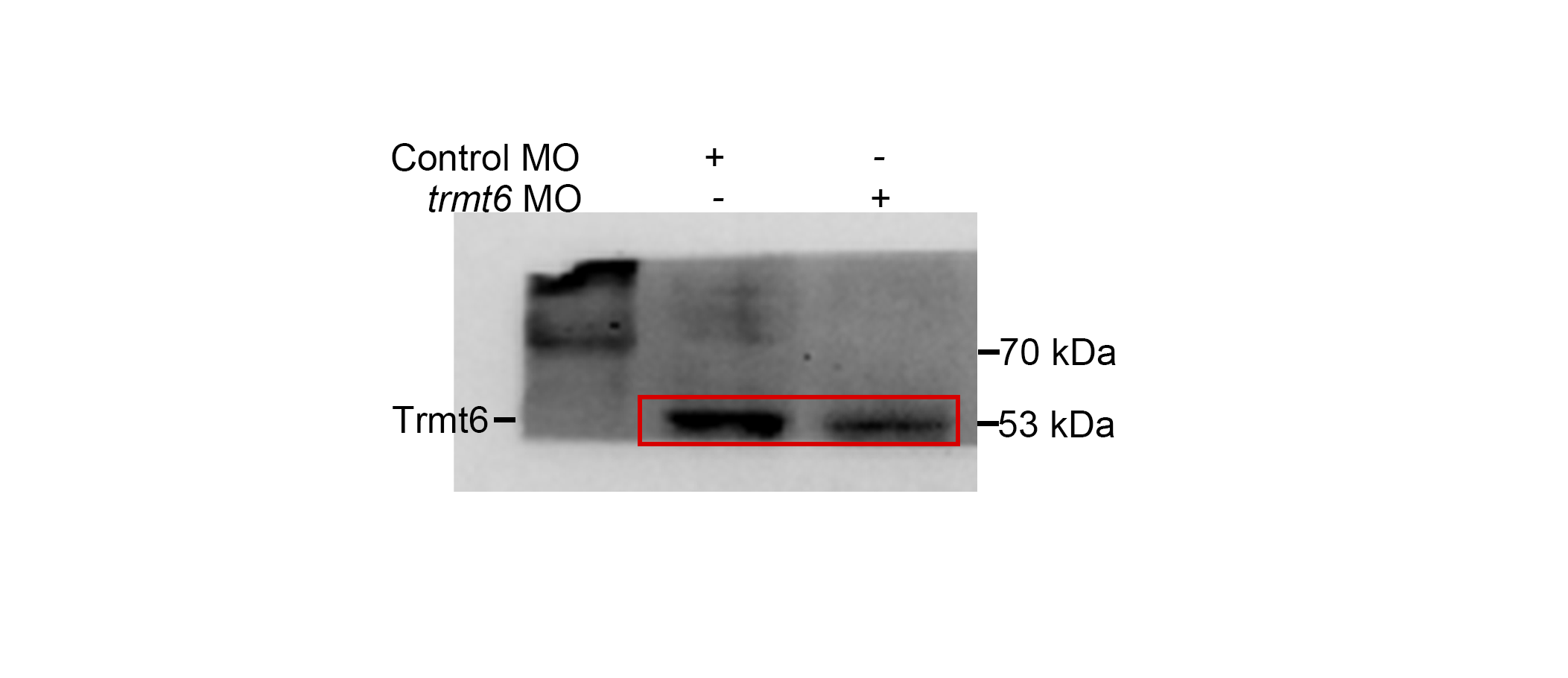

Supplement: Supplementary file 13 — Appendix Figure 5-7 Source Data [file 44319_2026_805_MOESM13_ESM.zip › Appendix Source Data 3/Appendix Fig.6/A/6A_Trmt6 WB.tif]

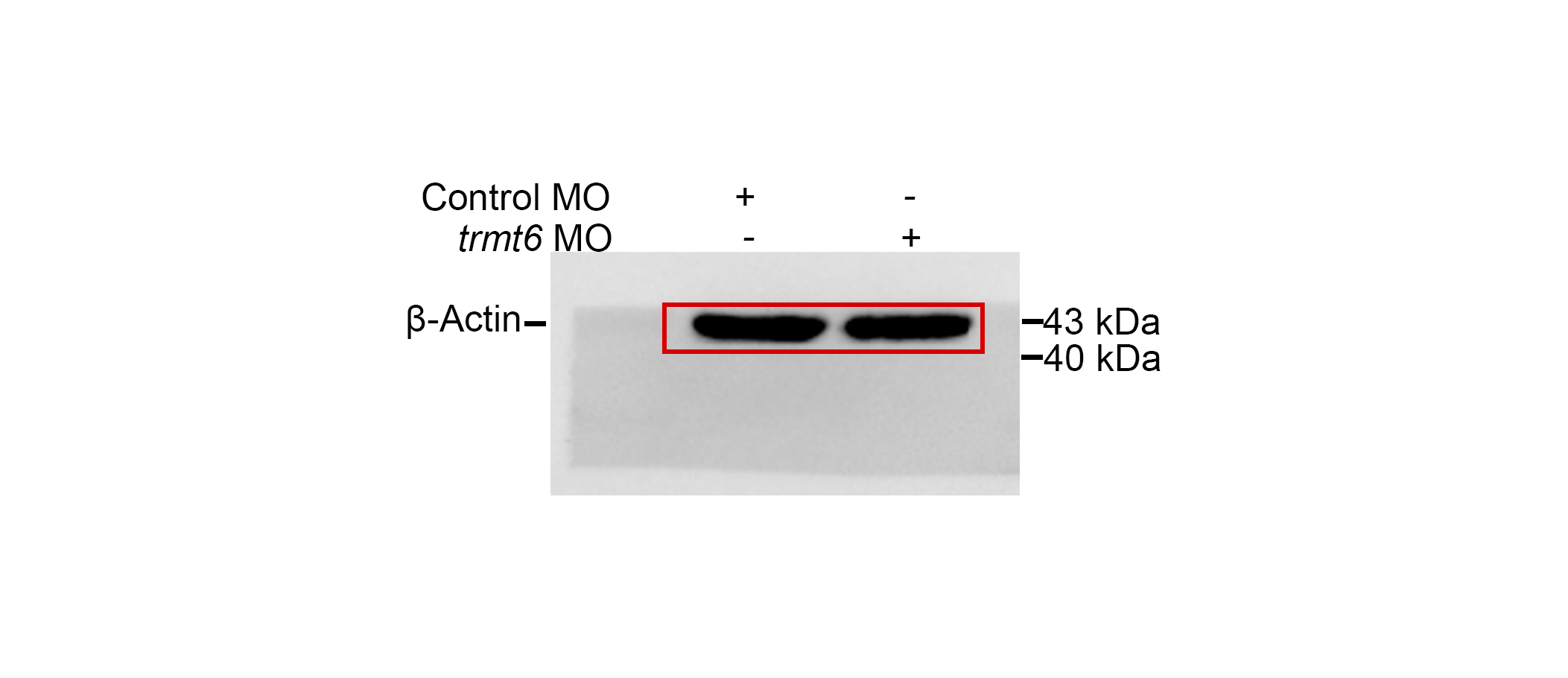

Supplement: Supplementary file 13 — Appendix Figure 5-7 Source Data [file 44319_2026_805_MOESM13_ESM.zip › Appendix Source Data 3/Appendix Fig.6/A/6A_β-Actin WB.tif]

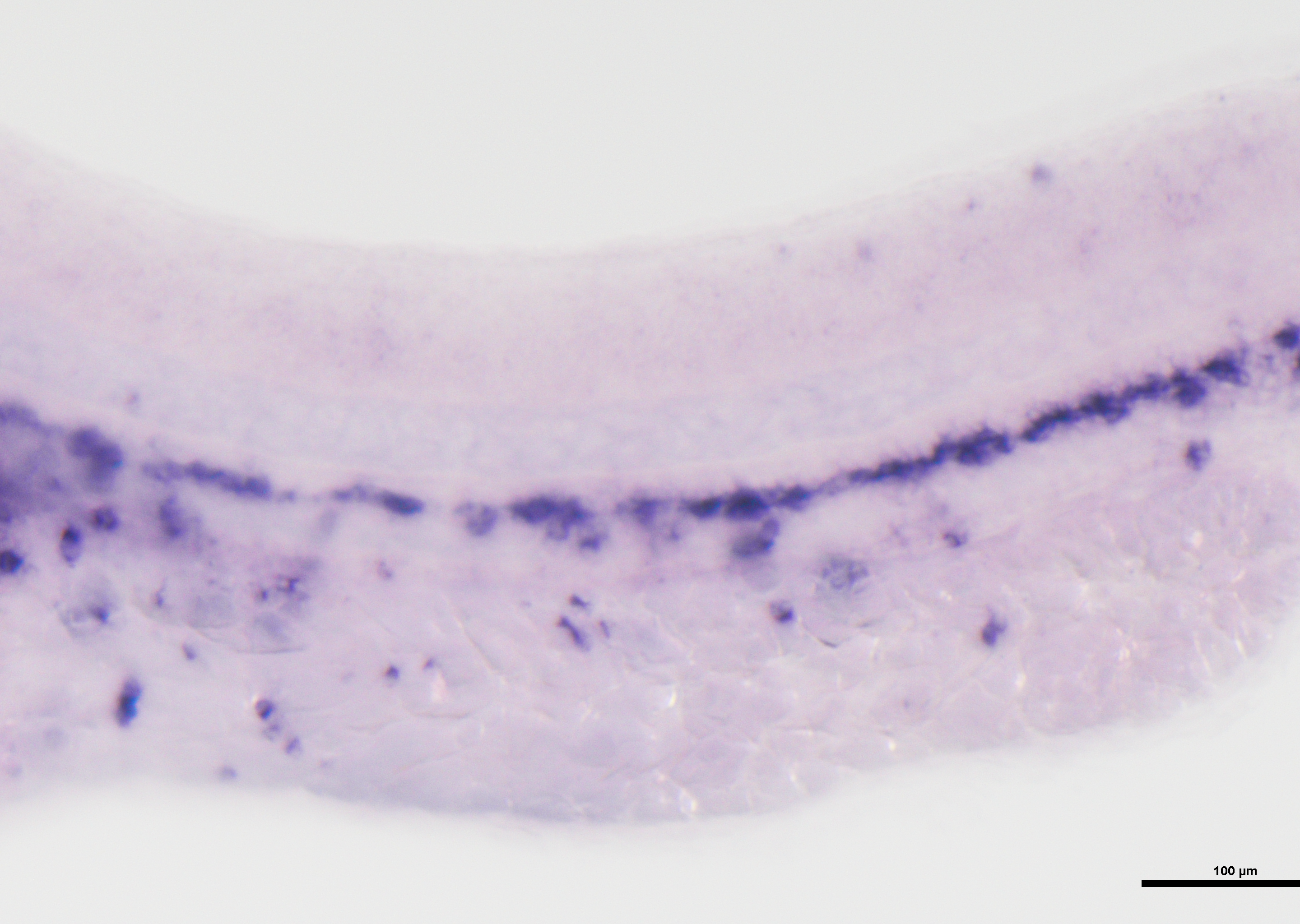

Supplement: Supplementary file 13 — Appendix Figure 5-7 Source Data [file 44319_2026_805_MOESM13_ESM.zip › Appendix Source Data 3/Appendix Fig.6/B/1. cmyb 36hpf controlMO.tif]

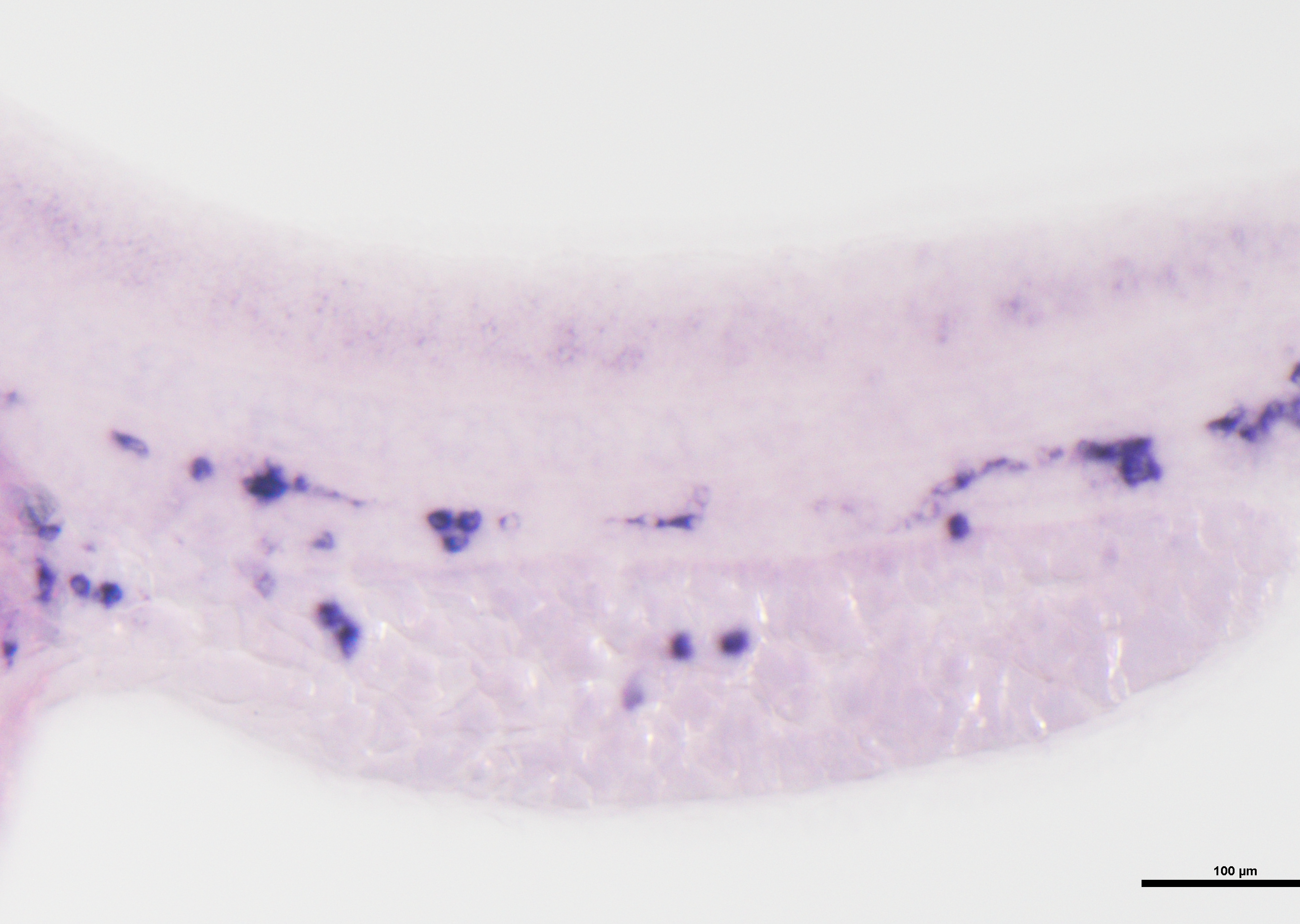

Supplement: Supplementary file 13 — Appendix Figure 5-7 Source Data [file 44319_2026_805_MOESM13_ESM.zip › Appendix Source Data 3/Appendix Fig.6/B/2. cmyb 36hpf trmt6MO.tif]

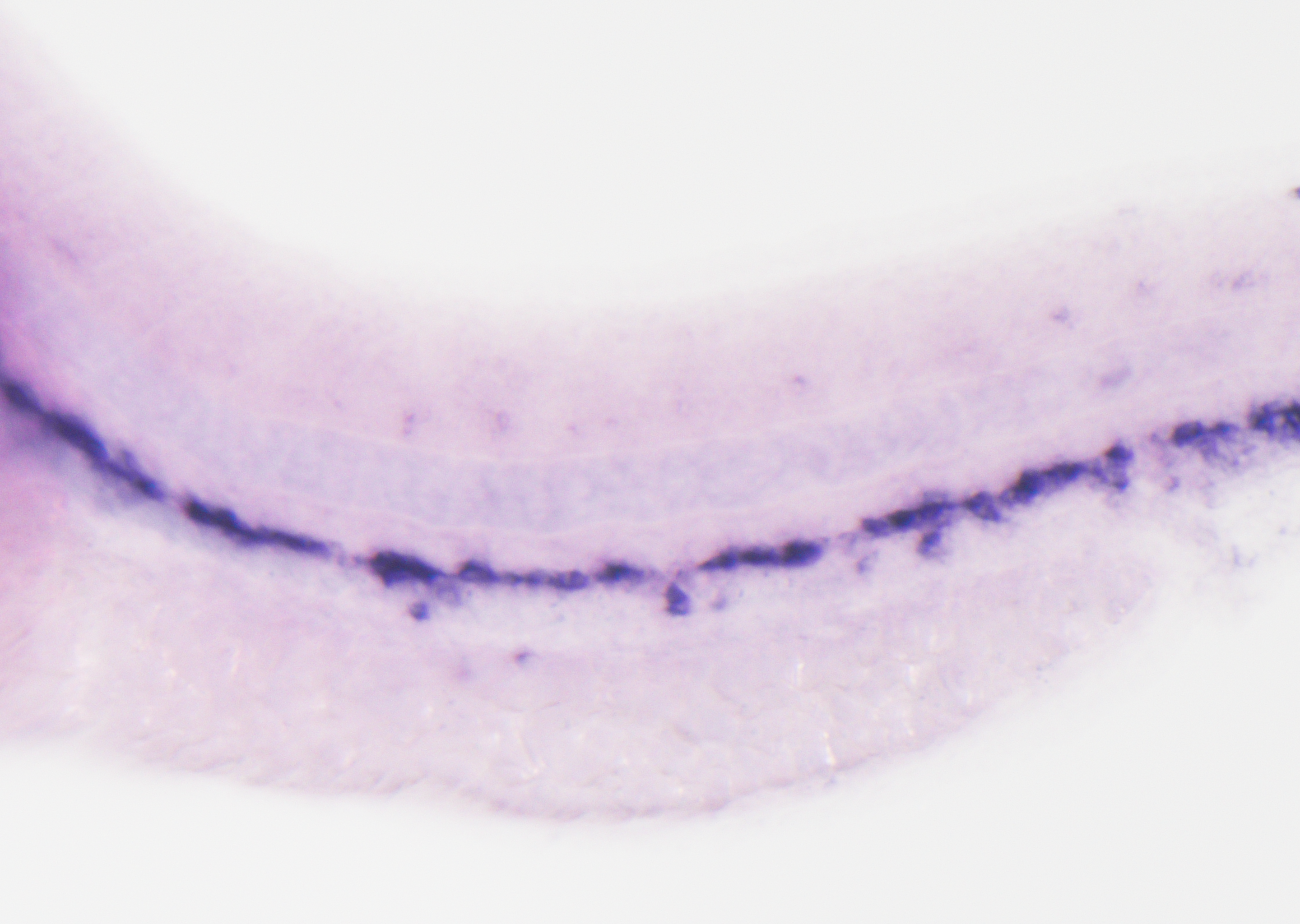

Supplement: Supplementary file 13 — Appendix Figure 5-7 Source Data [file 44319_2026_805_MOESM13_ESM.zip › Appendix Source Data 3/Appendix Fig.6/B/3. runx1 36hpf controlMO.tif]

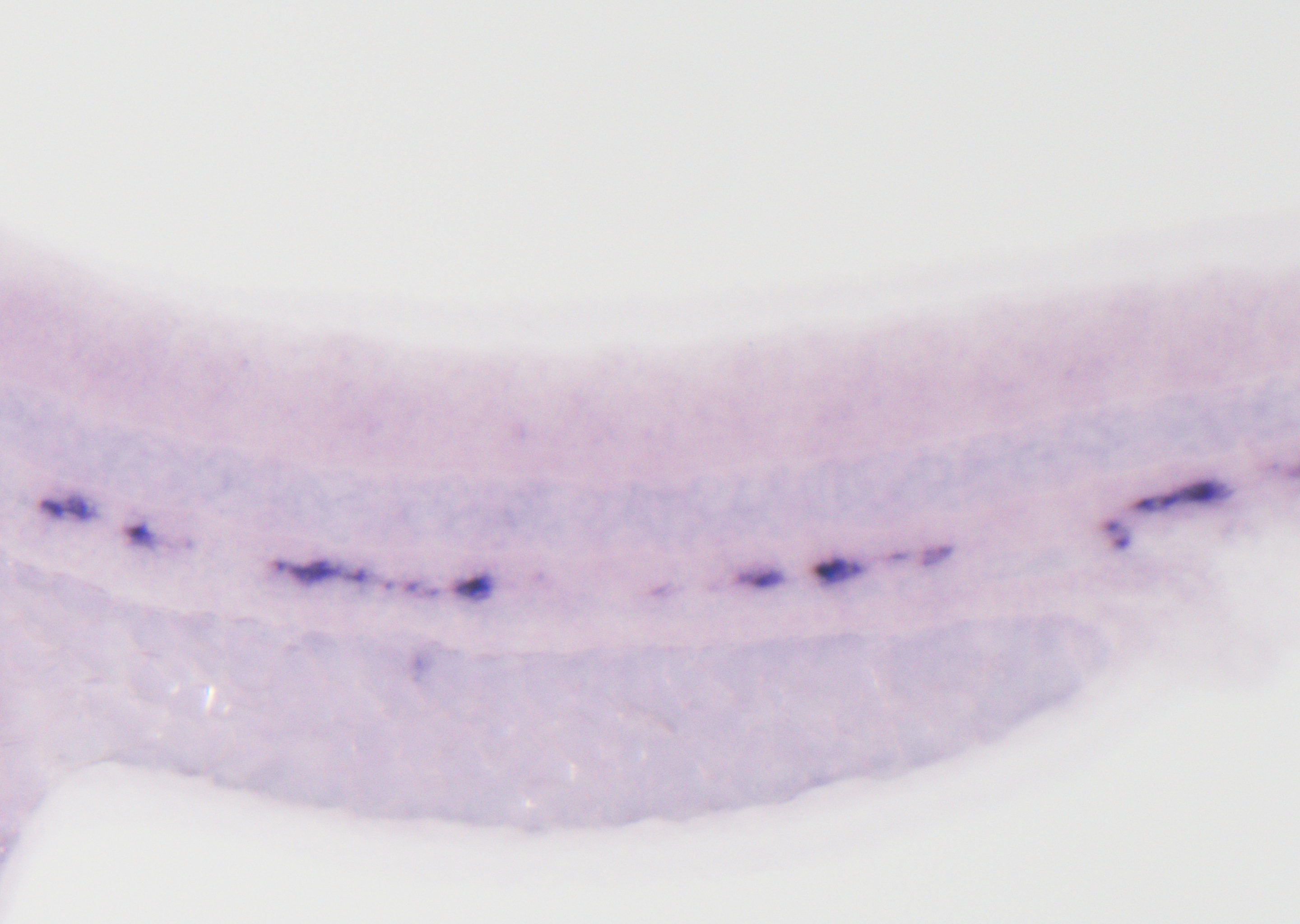

Supplement: Supplementary file 13 — Appendix Figure 5-7 Source Data [file 44319_2026_805_MOESM13_ESM.zip › Appendix Source Data 3/Appendix Fig.6/B/4. runx1 36hpf trmt6MO.tif]

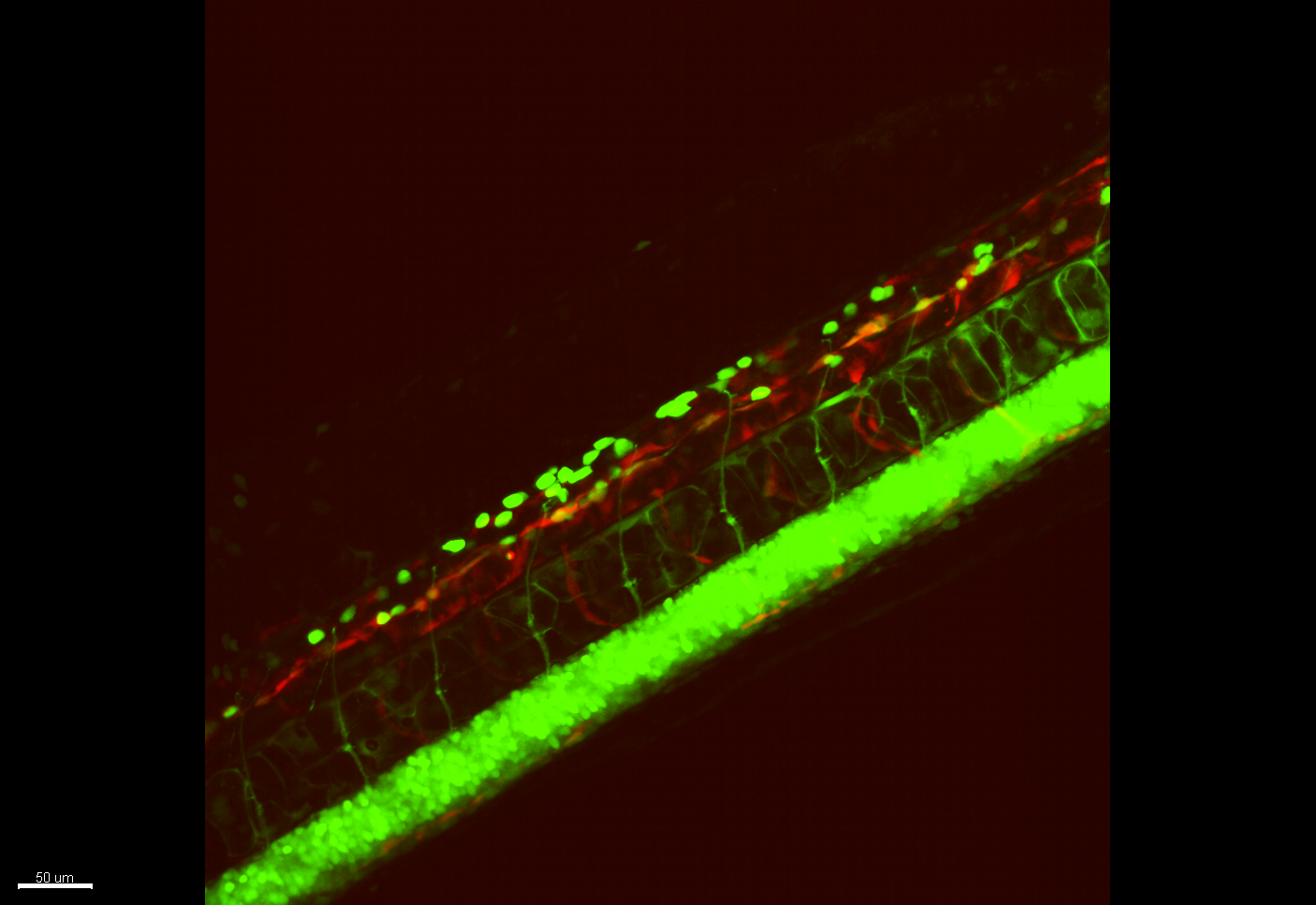

Supplement: Supplementary file 13 — Appendix Figure 5-7 Source Data [file 44319_2026_805_MOESM13_ESM.zip › Appendix Source Data 3/Appendix Fig.6/D/1. 36hpf controlMO.png]

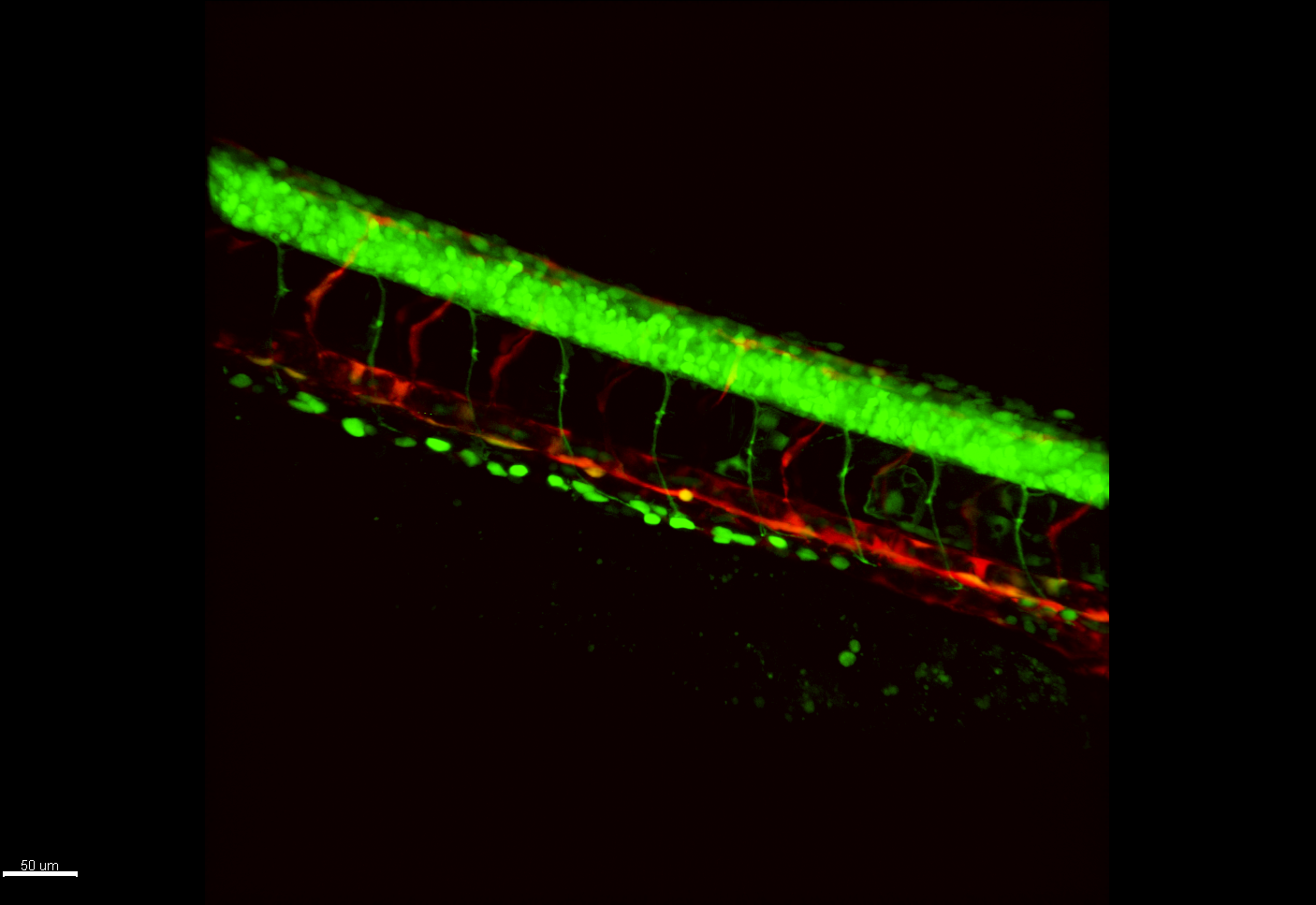

Supplement: Supplementary file 13 — Appendix Figure 5-7 Source Data [file 44319_2026_805_MOESM13_ESM.zip › Appendix Source Data 3/Appendix Fig.6/D/2. 36hpf trmt6MO.png]

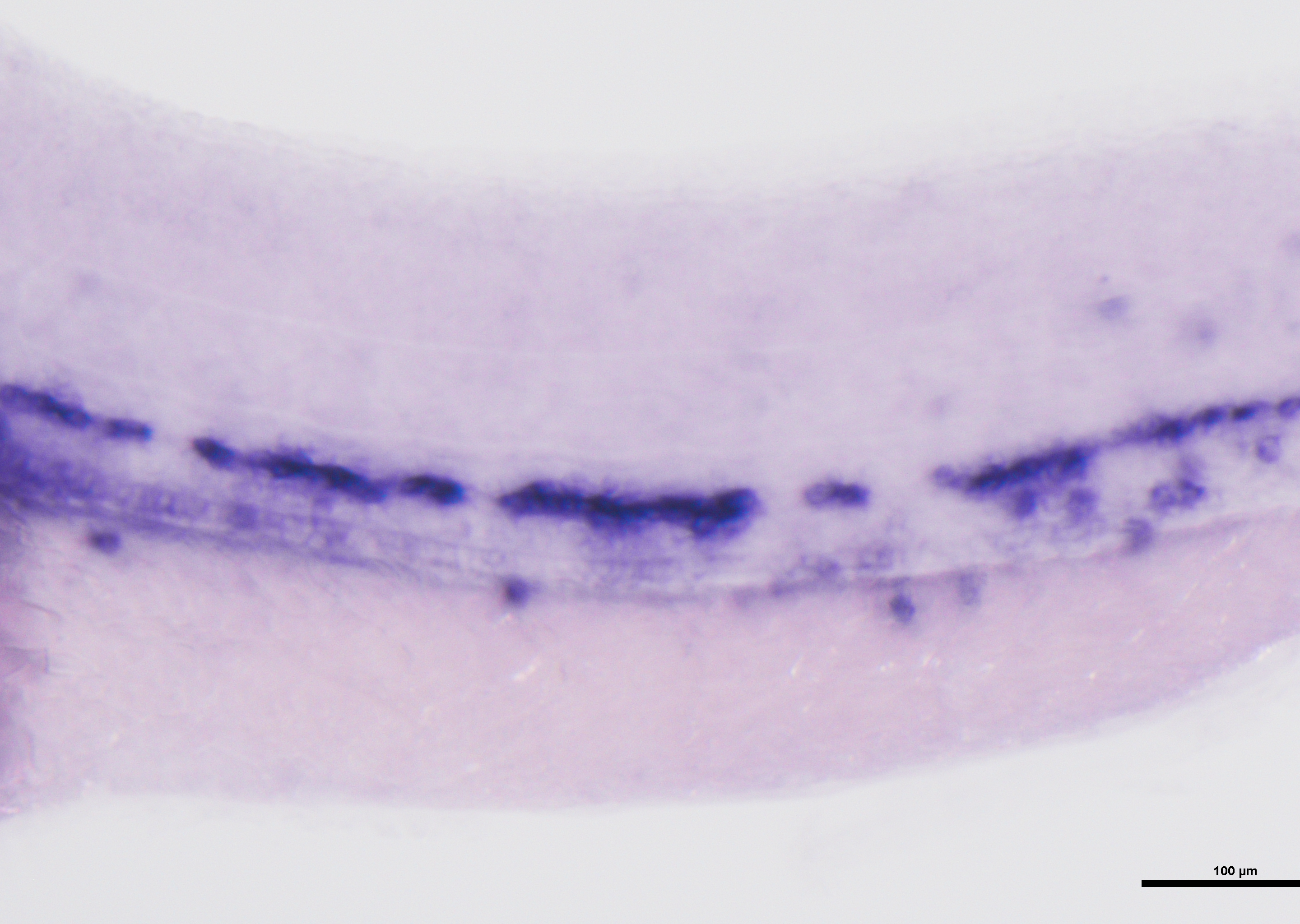

Supplement: Supplementary file 13 — Appendix Figure 5-7 Source Data [file 44319_2026_805_MOESM13_ESM.zip › Appendix Source Data 3/Appendix Fig.6/E/1. cmyb 36hpf controlMO.tif]

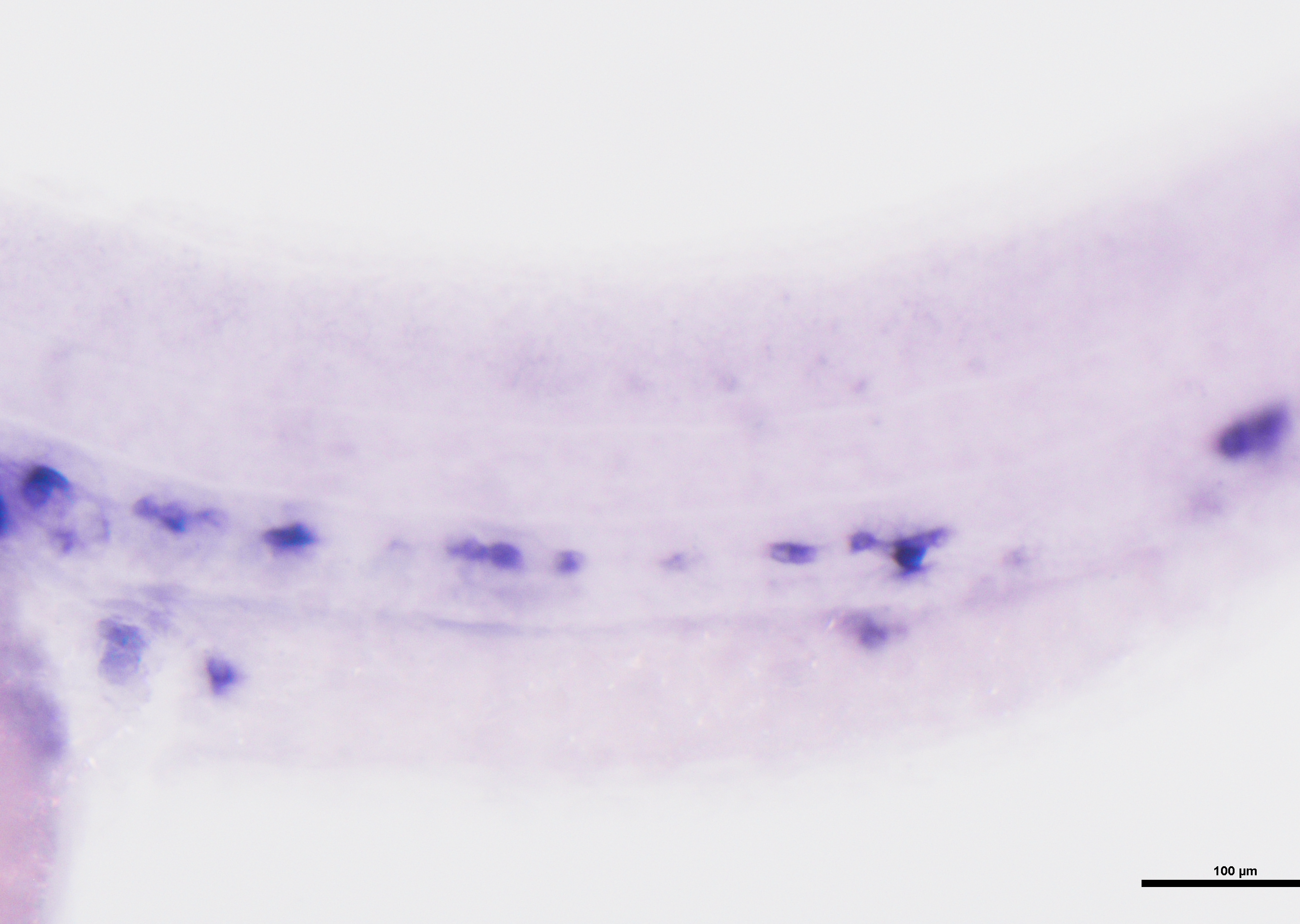

Supplement: Supplementary file 13 — Appendix Figure 5-7 Source Data [file 44319_2026_805_MOESM13_ESM.zip › Appendix Source Data 3/Appendix Fig.6/E/2. cmyb 36hpf trmt6MO.tif]

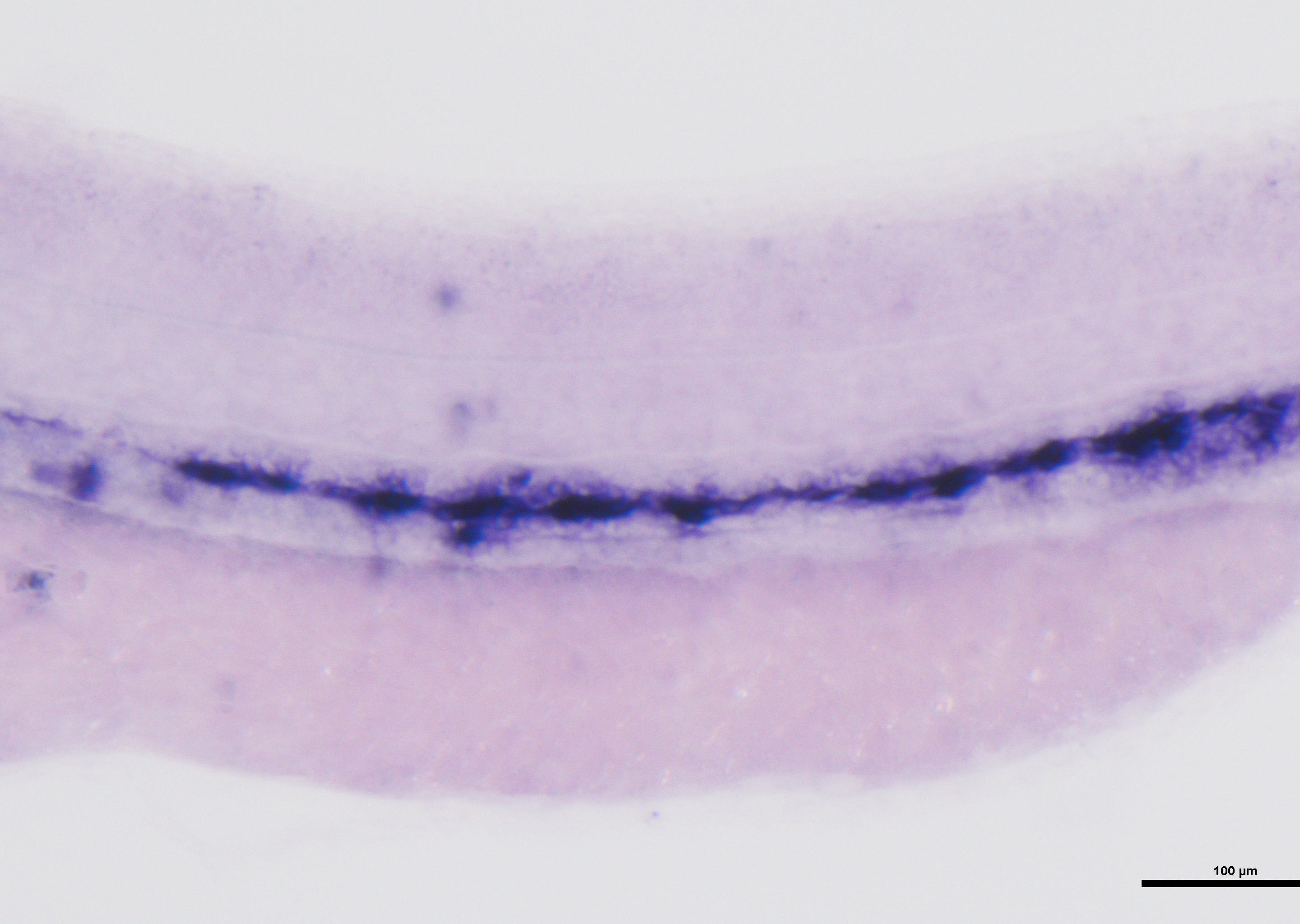

Supplement: Supplementary file 13 — Appendix Figure 5-7 Source Data [file 44319_2026_805_MOESM13_ESM.zip › Appendix Source Data 3/Appendix Fig.6/E/3. cmyb 36hpf trmt6MO+trmt6mRNA.tif]

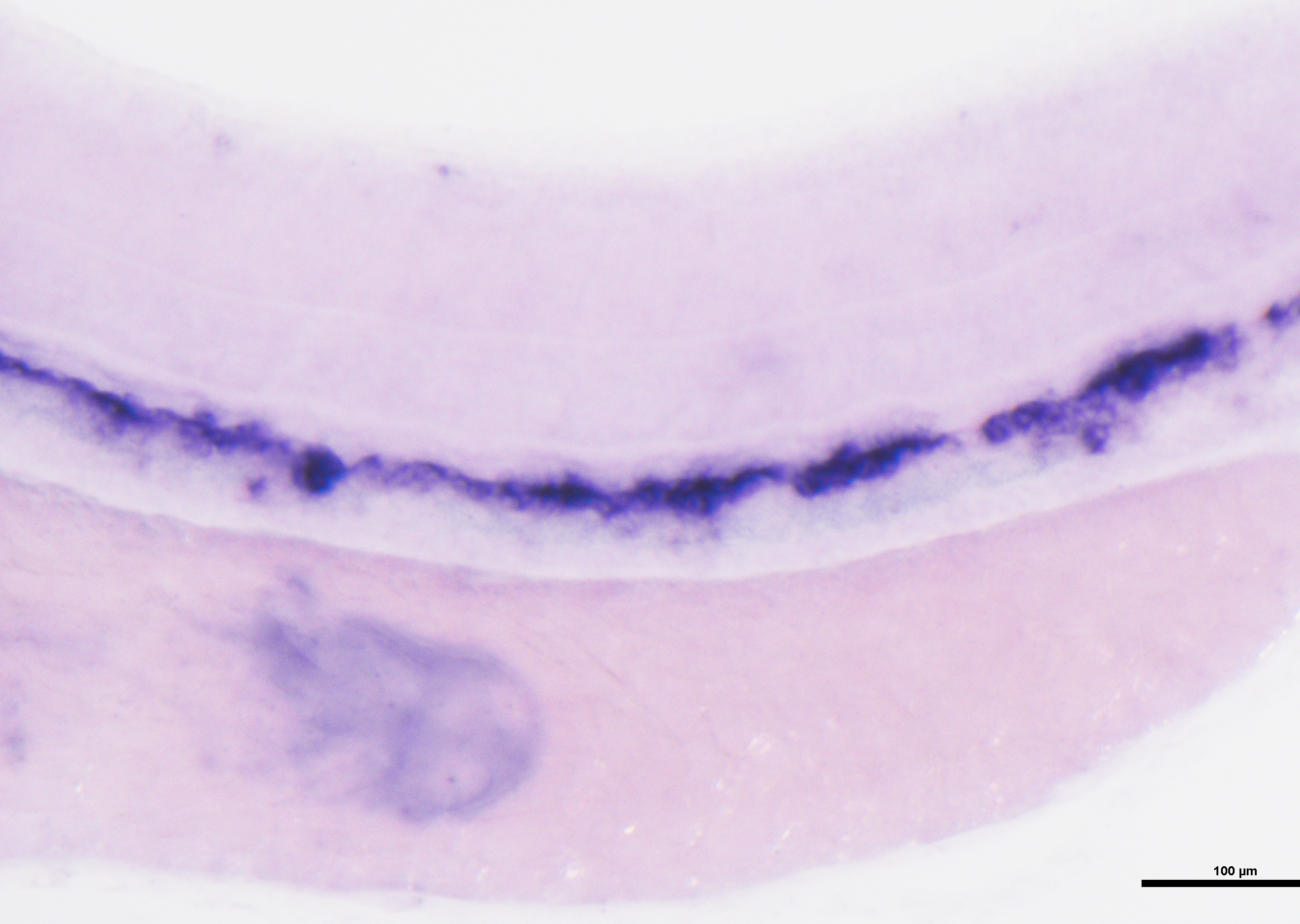

Supplement: Supplementary file 13 — Appendix Figure 5-7 Source Data [file 44319_2026_805_MOESM13_ESM.zip › Appendix Source Data 3/Appendix Fig.6/E/4. runx1 36hpf controlMO.tif]

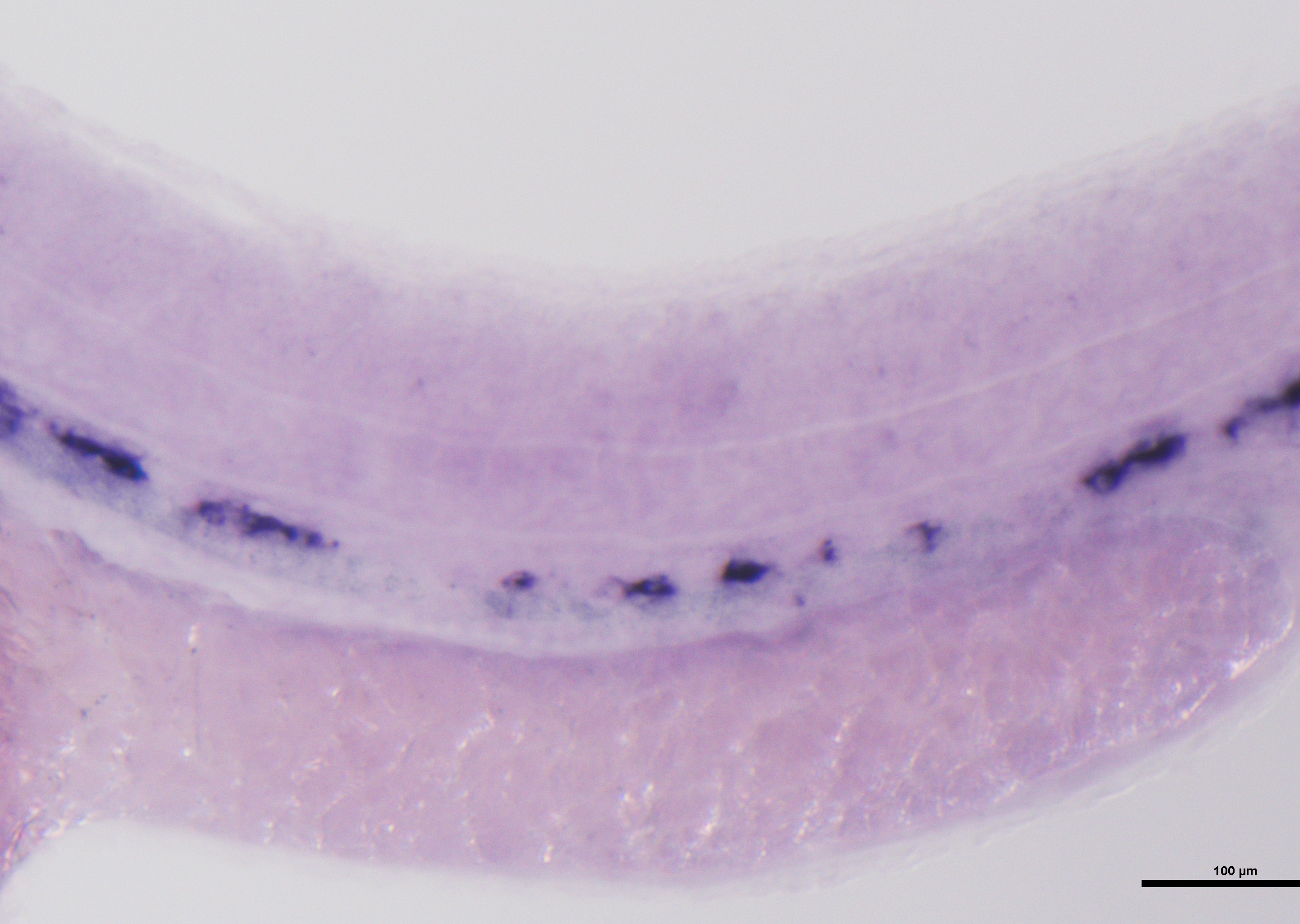

Supplement: Supplementary file 13 — Appendix Figure 5-7 Source Data [file 44319_2026_805_MOESM13_ESM.zip › Appendix Source Data 3/Appendix Fig.6/E/5. runx1 36hpf trmt6MO.tif]

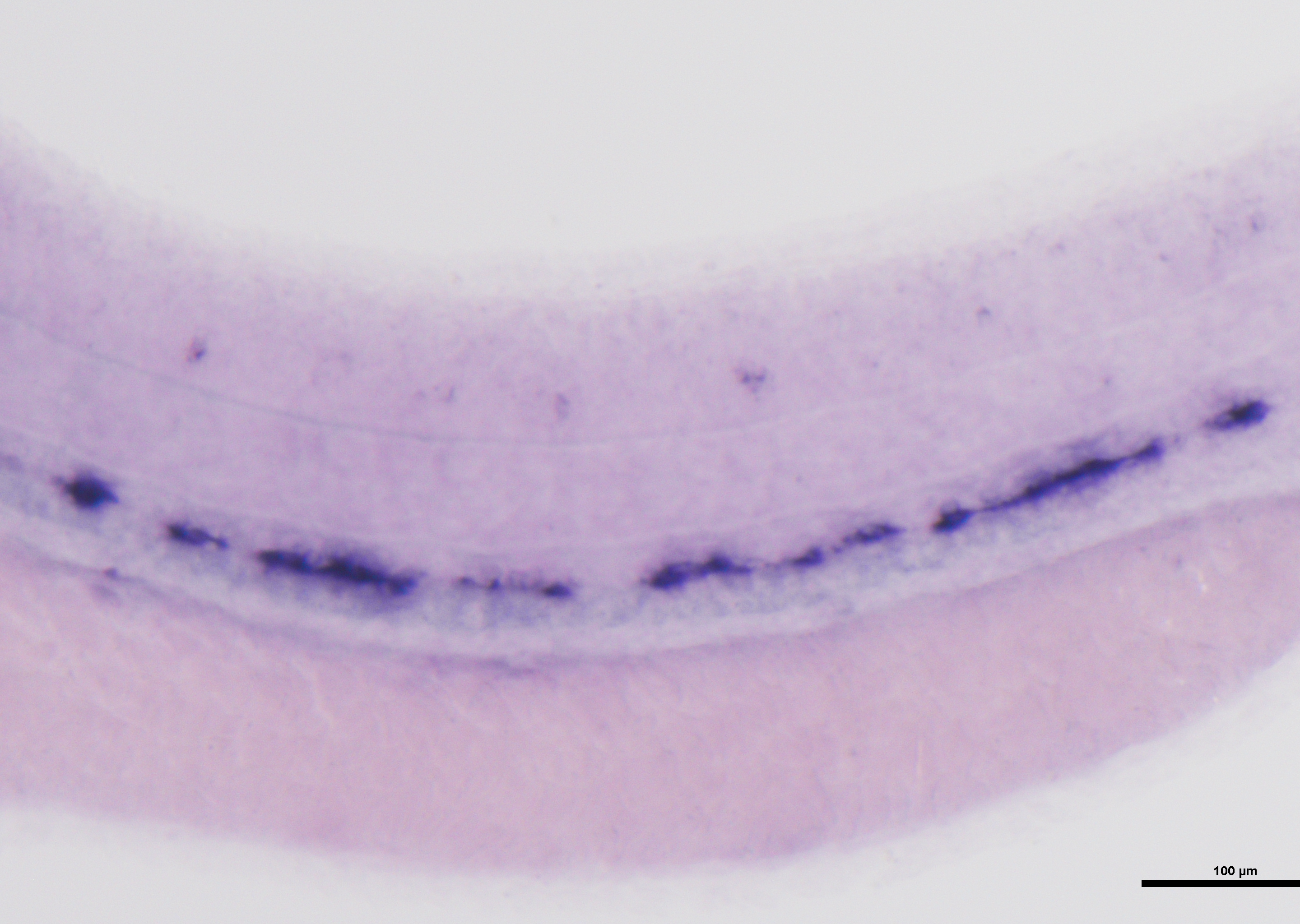

Supplement: Supplementary file 13 — Appendix Figure 5-7 Source Data [file 44319_2026_805_MOESM13_ESM.zip › Appendix Source Data 3/Appendix Fig.6/E/6. runx1 36hpf trmr6MO+trmt6mRNA.tif]

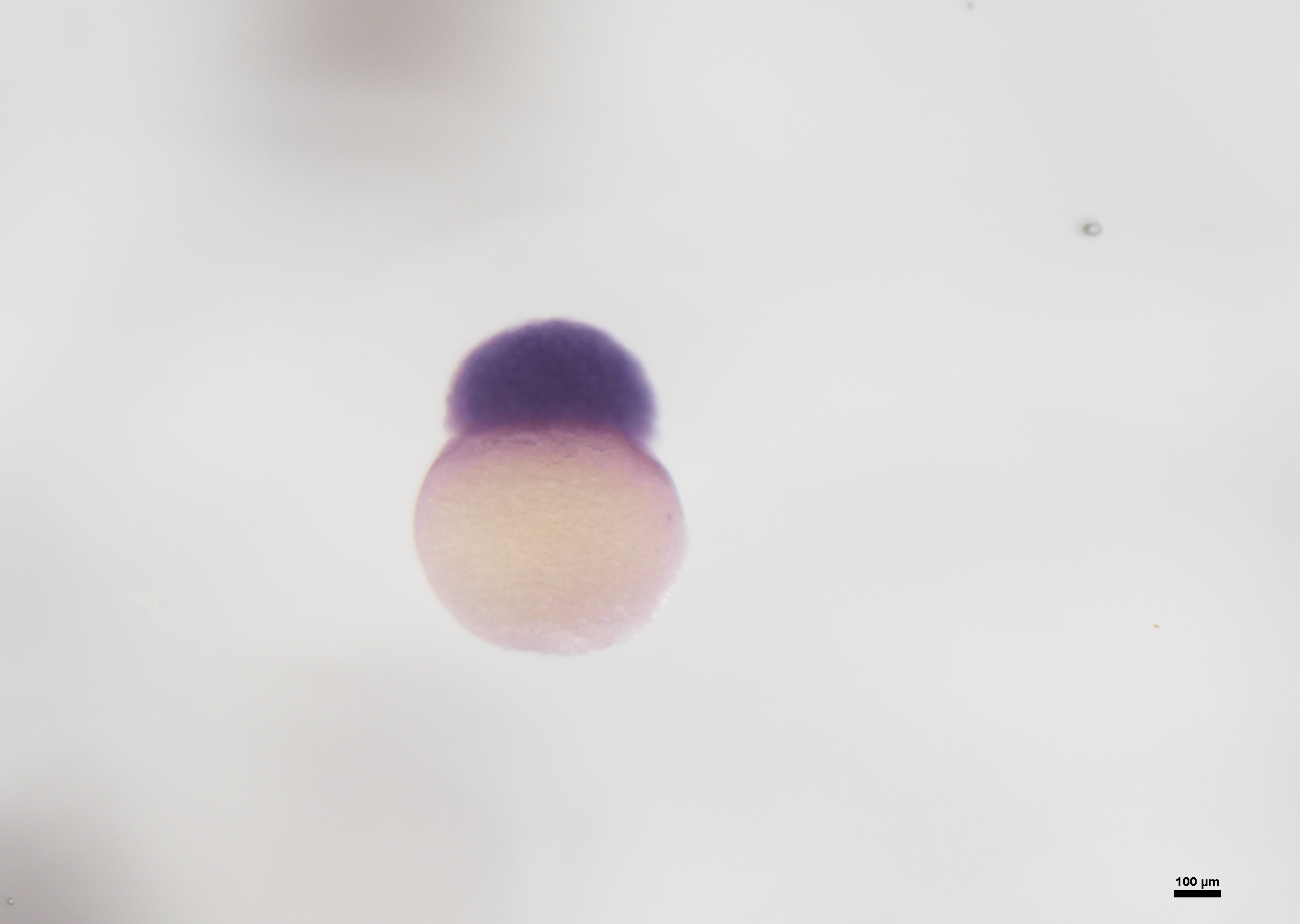

Supplement: Supplementary file 13 — Appendix Figure 5-7 Source Data [file 44319_2026_805_MOESM13_ESM.zip › Appendix Source Data 3/Appendix Fig.7/D/1. nrf1 1k-cell.tif]

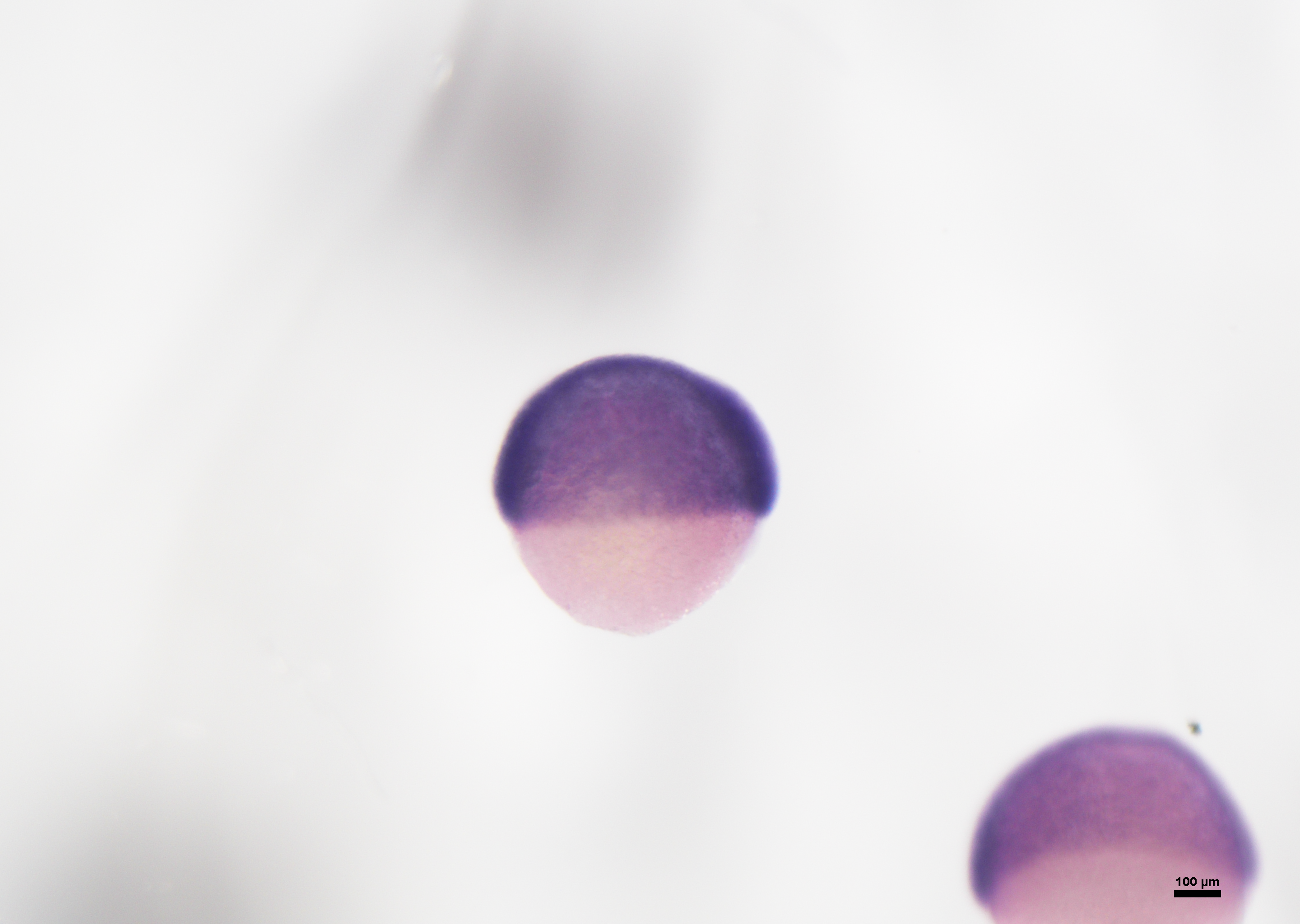

Supplement: Supplementary file 13 — Appendix Figure 5-7 Source Data [file 44319_2026_805_MOESM13_ESM.zip › Appendix Source Data 3/Appendix Fig.7/D/2. nrf1 shield.tif]

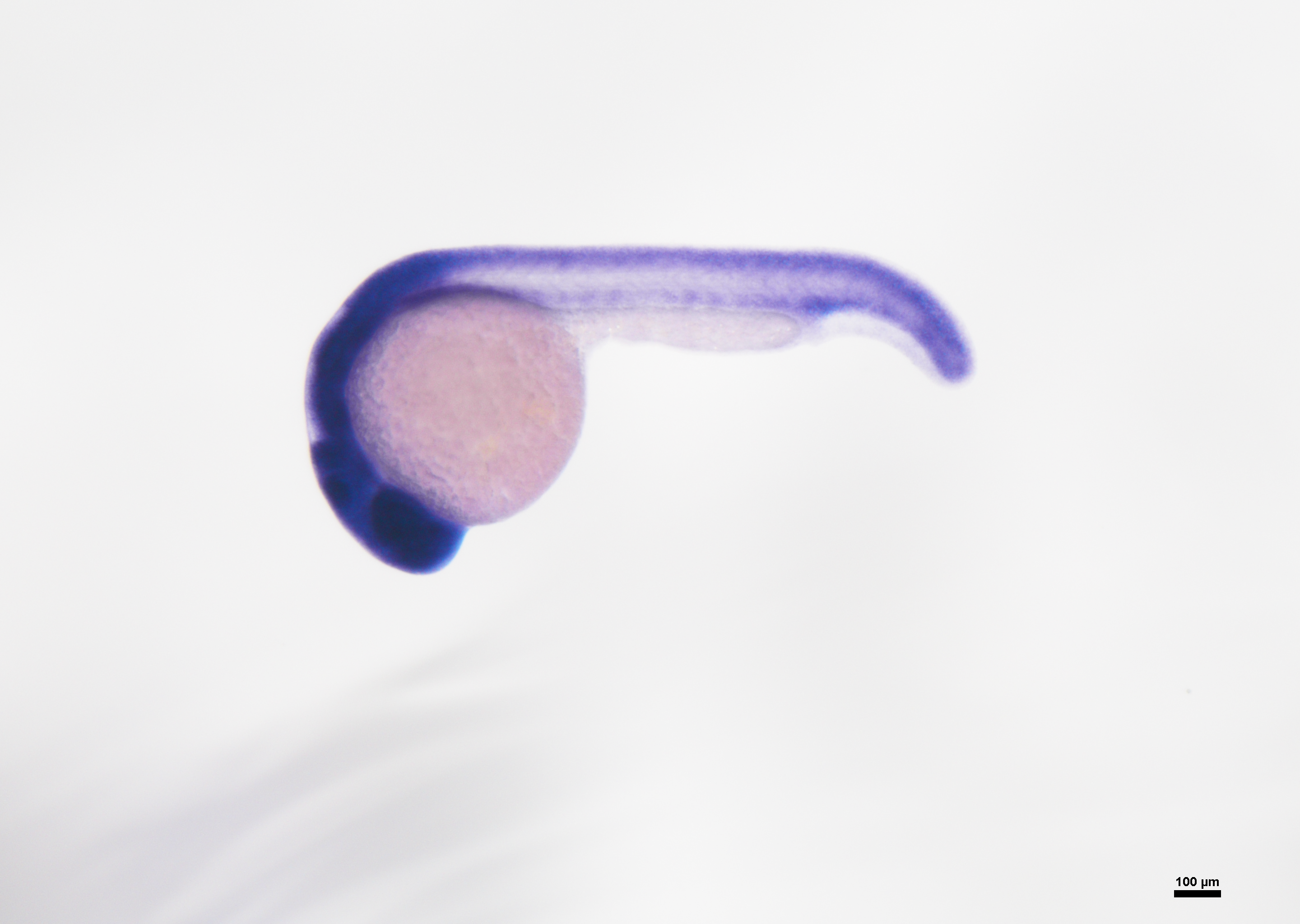

Supplement: Supplementary file 13 — Appendix Figure 5-7 Source Data [file 44319_2026_805_MOESM13_ESM.zip › Appendix Source Data 3/Appendix Fig.7/D/3. nrf1 24hpf.tif]

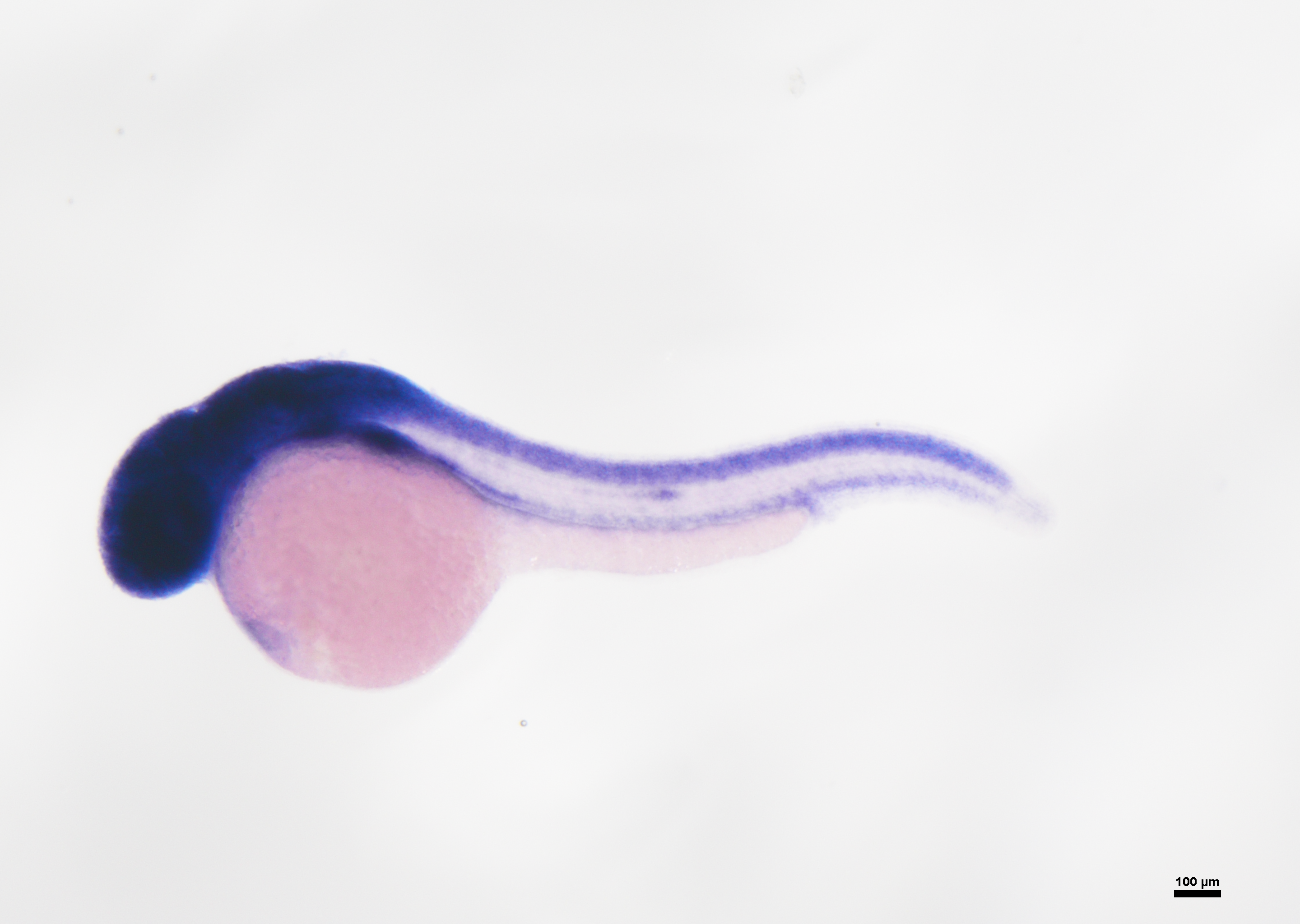

Supplement: Supplementary file 13 — Appendix Figure 5-7 Source Data [file 44319_2026_805_MOESM13_ESM.zip › Appendix Source Data 3/Appendix Fig.7/D/4. nrf1 36hpf.tif]

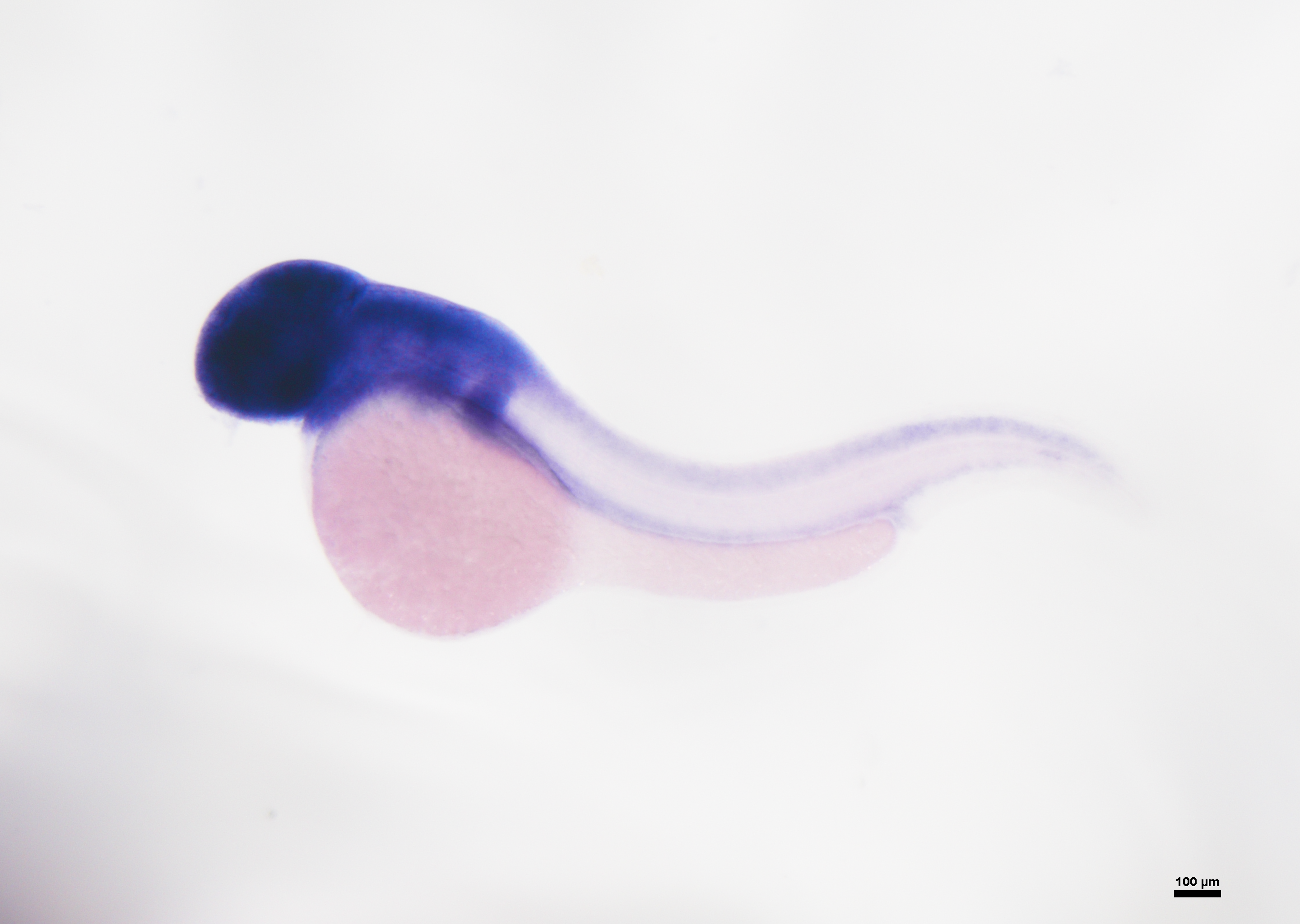

Supplement: Supplementary file 13 — Appendix Figure 5-7 Source Data [file 44319_2026_805_MOESM13_ESM.zip › Appendix Source Data 3/Appendix Fig.7/D/5. nrf1 2dpf.tif]
