# Supplementary material for: tRNA m1A modification ensures HSPC production via modulating Nrf1 translation in zebrafish
Source: EMBO Rep. 2026 May 27;27(13):3826–41. doi: 10.1038/s44319-026-00805-5 (PMC13354807; doi:10.1038/s44319-026-00805-5)
Supplement: Supplementary file 3 — Peer Review File [file 44319_2026_805_MOESM3_ESM.pdf]

EMBO reports

tRNA m1A modification ensures HSPC production via modulating Nrf1 translation in zebrafish

Zhenkun Dong, Panfeng Li, Mengyao Liu, Sifeng Wang, Weiyi Lai, Yining Liu, Hailin Wang, Ang Li, and Lu Wang

Corresponding author(s): Lu Wang (wanglu1@ihcams.ac.cn)

Review Timeline:

Submission Date:	30th Jan 26
Editorial Decision:	2nd Mar 26
Revision Received:	7th Mar 26
Editorial Decision:	26th Mar 26
Revision Received:	29th Mar 26
Editorial Decision:	24th Apr 26
Revision Received:	24th Apr 26
Accepted:	30th Apr 26

Editor: Martina Rembold

Transaction Report: This manuscript was submitted to EMBO Reports after peer review at another journal.

(Note: With the exception of the correction of typographical or spelling errors that could be a source of ambiguity, letters and reports are not edited. Depending on transfer agreements, referee reports obtained elsewhere may or may not be included in this compilation. Referee reports are anonymous unless the Referee chooses to sign their reports.)

Reviewer Comments:
Reviewer #1:
Suitable Quality? Yes
Sufficient General Interest? Yes
Conclusions Justified? Yes
Clearly Written? Yes
Procedures Described? Yes
Supplemental Material Warranted? Yes
Sufficient data/samples? Yes

Comments on Significance Statement:
None.

Comments:
The authors addressed all our comments in a satisfying way. However, it is too bad that they gave up on everything that was ROS-related.

Reviewer #2:
Suitable Quality? No
Sufficient General Interest? Yes
Conclusions Justified? No
Clearly Written? Yes
Procedures Described? No
Supplemental Material Warranted? Yes
Sufficient data/samples? No

Comments:
Summary of Revision：
This reviewer appreciates the efforts the authors made to expand the details in their methods sections and to provide additional data to support their claims. However, the authors did not sufficiently address the concerns about the trmt61a loss of function models used and thus lack rigorous evidence to support their conclusions over other interpretations. Additional concerns were also not addressed. Some of issues not addressed are noted below:
1. An area that was not sufficiently addressed was that p53-induced apoptosis in trmt61A morphants was specific to loss of trmt61A and not due to a non-specific consequence of morpholino. 15-20% of morpholinos will non-specifically induce p53 (PMID: 18403413). This reviewer appreciates that the authors attempted to address this issue by co-injecting p53 and trmt61A morpholinos. However, the rescue of HSPC number in this condition cannot distinguish between specific and non-specific effects of the morpholino. Eisen et al. 2008 (PMID: 18403413) clearly describes the limitations of using morpholinos to examine phenotypes involving p53-induced cell death. Just because the control MO doesn't induce p53, doesn't mean trmt61A will.
R1： In our initial revision, we presented data comparing *p53* expression levels between embryos injected with a Control MO and those injected with *trmt61a* MO. The results demonstrated upregulated *p53* expression in the *trmt61a* MO-injected group relative to the control (see below

Response Figure 1A). To further exclude potential non-specific effects associated with MO injection, we subsequently assessed *p53* expression in *trmt61a* mutant embryos (M*trmt61a*;*trmt61a*$^{-4bp}$) and their siblings. This analysis confirmed that the genetic ablation of *trmt61a* also leads to a marked elevation in *p53* expression (see below **Response Figure 1B and C,** also see **Fig. 3D-E**), thus reinforcing the conclusion that the observed *p53* upregulation is a specific consequence of Trmt61a deficiency.

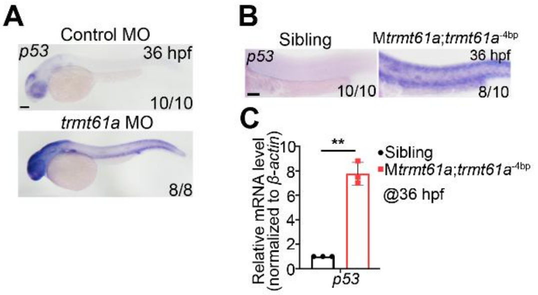

Response Figure 1. Trmt61a deficiency activates *p53* expression. (A) WISH showing the expression of *p53* in control morphants and *trmt61a* morphants at 36 hpf. (B-C) WISH (B) and qPCR (C) showing the expression of *p53* in sibling and M*trmt61a*;*trmt61a*$^{-4bp}$ embryos at 36 hpf. The numbers indicating the number of embryos with representative phenotype/total number of embryos in each group (A, B). Scale bars: 100 μm (A, B). Error bars represent mean ± SD. Two-tailed unpaired Student's t-test; ***p*<0.01. n≥3 biological replicates.

Other controls were also not conducted (dose curve, a second MO) and the authors said that the other trmt61A deficiency models were rigorous enough. Since the other models were also not fully validated, this justification is not enough. While this reviewer appreciates the difficulty in obtaining Mtrmt61a; trmt61a-4bp embryos, there is some lack of data/explanation whether Mtrmt61a embryos or Mtrmy61a;trmt61a-4bp hets have a phenotype and could be used instead. Identifying a rigorously validated genetic model is needed considering the involvement of p53-mediated cell death.

R2: While we did design and utilize a second MO, it consistently produced pronounced off-target developmental phenotypes, such as axial curvature. Although this second MO-injection recapitulated the HSPC defects observed in the AUG-targeting *trmt61a* MO-injected embryos in the manuscript (see below **Response Figure 2**), we chose not to include its data to avoid confounding interpretations.

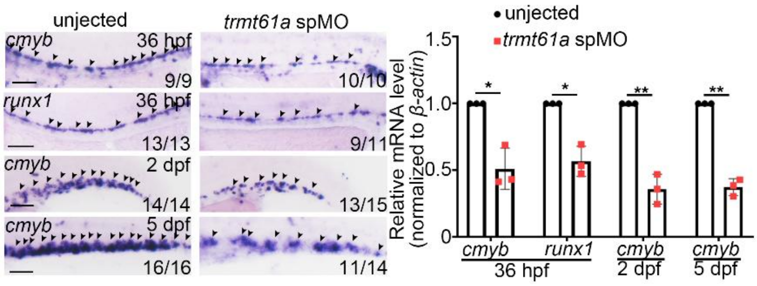

Response Figure 2. *trmt61a* spliceMO-injection leads to abnormal hematopoietic development. WISH results (left) with qPCR (right) showing *runx1* expression at 36 hpf in the AGM region, *cmyb* expression at 36 hpf in the AGM region, at 2 dpf and 4 dpf in the CHT region in uninjected- and *trmt61a* spliceMO-injected embryos. Error bars represent mean ± SD. Two-tailed unpaired Student's t-test; *p<0.05; **p<0.01. n≥3 biological replicates. Arrowheads denote marker gene-positive signals. The numbers indicating the number of embryos with representative phenotype/total number of embryos in each group. Scale bars: 100 μm.

Importantly, we contend that the evidence presented in the current manuscript is robust. By employing three distinct models—MO-mediated knockdown, a genetic knockout, and an enzyme-inactive point mutant—we have sufficiently demonstrated that the loss of *trmt61a* function leads to HSPC defects. We will elaborate on this point further in our subsequent response.

Regarding the phenotypes of *Mtrmt61a* or *Mtrmt61a;trmt61a-4bp* heterozygous embryos: in our experiments involving the *Mtrmt61a;trmt61a-4bp* mutant line, we used sibling embryos as controls. This control pool included wild-type, *Mtrmt61a;trmt61a+* (*Mtrmt61a*), and *trmt61a+/-4bp* genotypes. We found no significant differences among these sibling groups in terms of HSPC phenotypes, or *p53* expression. Therefore, these embryos were collectively pooled and served as the "sibling control" group for data presentation. For instance, we confirmed that *cmyb* expression in *Mtrmt61a* embryos and *trmt61a+/-4bp* embryos was not significantly altered when compared to control embryos (see below **Response Figure 3A-B,** also see **Fig EV.5K and 3C**).

We propose that the absence of a phenotype in these heterozygous embryos is due to the continued presence of sufficient Trmt61a protein during the critical window of HSPC emergence. We experimentally confirmed this sustained protein expression in *trmt61a+/-* embryos (see below **Response Figure 3C,** also see **Fig EV3B**). While we were unable to perform protein-level validation for the other two heterozygous genotypes due to the difficulty of obtaining sufficient numbers of these embryos, we infer that the same principle applies.

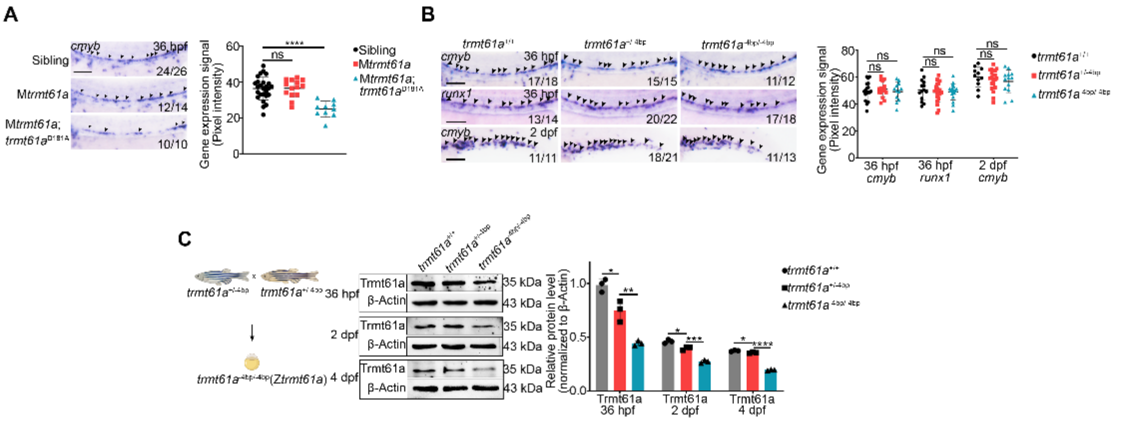

Response Figure 3. Maternal trmt61a mutants (*Mtrmt61a*) and *trmt61a+/-* embryos display unaltered HSPC generation. (A) WISH (left) and quantification (right) at 36 hpf showing the expression of *cmyb* in siblings, M*trmt61a*, and M*trmt61a*;*trmt61a*^{D181A} embryos. Arrowheads denote *cmyb* positive signals. (B). WISH results (left) and quantification (right) showing the expression of *cmyb* and *runx1* at 36 hpf and 2 dpf in *trmt61a*$^{+/+}$, *trmt61a*$^{+/-4bp}$ and *trmt61a* $^{-4bp/-4bp}$ embryos. Arrowheads denote *runx1* or *cmyb* positive signals. The numbers indicating the number of embryos with representative phenotype/total number of embryos in each group. **(C)** Generation of *trmt61a* zygotic mutant (Z*trmt61a*, left), and the protein level of Trmt61a (middle) with quantification (right) at 36 hpf, 2 dpf and 4 dpf in *trmt61a*$^{+/+}$, *trmt61a*$^{+/-4bp}$ and *trmt61a* $^{-4bp/-4bp}$ embryos. Scale bars: 100 μm. Error bars represent mean ± SD. Two-tailed unpaired Student's *t*-test; $*p<0.05$; $**p<0.01$; $***p<0.001$; $****p<0.0001$; ns, no significance. n≥3 biological replicates.

2. Another loss of function model that lacks rigor is the trmt61A D181A knock-in model. The reviewer appreciates the additional technical details and schematic that were added. However, data is lacking that validates the desired function of this model. The authors give the impression that the CRISPR/Cas9 strategy used would simply create a catalytic D181A mutation in the endogenous locus (Reviewer response R28-29). This is not how this strategy was designed.

R3: We apologize for the lack of detailed methodological information in the initial manuscript. We have now added this information into the Methods section and Figure legend. The knock-in strategy employed in this study is based on the "Intron targeting-mediated and endogenous gene integrity-maintaining knock-in method" as previously reported [1]. We designed a donor plasmid consisting of three parts: a left arm, a P2A-EGFP coding sequence, and a right arm. To preserve the full coding sequence of *trmt61a*, the left arm begins from the upstream of the sgRNA target site in intron 2, spans the whole exon3 which harbors a specific point mutation (c.542A>C; p.D181A), and terminates at the last base just before the stop codon of t*rmt61a* in exon4. The right arm contains the stop codon and 3′ untranslated region of *trmt61a* (see below **Response Figure 4** also see **Fig EV.5C**). Furthermore, to facilitate the identification of successfully edited embryos, the donor plasmid incorporates a cardiac-specific EGFP reporter, driven by the *myl7* promoter.

The donor construct was co-injected with sgRNA and Cas9 protein into 1-cell stage embryos. As both the donor plasmid DNA and the second intron of *trmt61a* contain sgRNA target site, concurrent cleavage by sgRNA/Cas9 would result in efficient and specific integration of the donor DNA into the *trmt61a* locus via non-homologous recombination (HR) (see below **Response Figure 4** also see **Fig EV.5C**). Larvae exhibiting cardiac EGFP expression were selected and raised to adulthood. Founders with germline transmission were identified through fluorescence pattern and junction PCR with sequencing verification, yielding stable *Ki*(*trmt61a*^{D181A}-EGFP) lines.

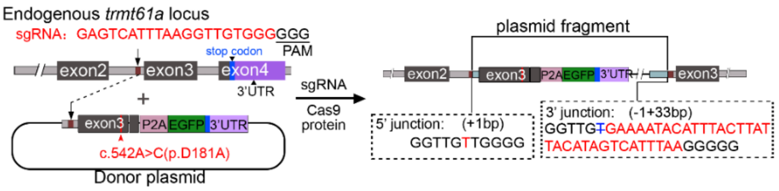

Response Figure 4. Procedure of the intron targeting-mediated strategy for generating trmt61a^{D181A} knockin zebrafish line by CRISPR/Cas9 system and 5' and 3' junction sequences of F1 progenies (right). The donor cassette was integrated into the trmt61a locus after co-injection of the donor with the sgRNA and Cas9 protein. The zebrafish trmt61a has 4 exons. The arrows in left panel indicate the sgRNA target sites.

Instead, the authors integrated a transgene that contained a whole new copy of the trmt61A coding sequence with D181A mutation (plus P2A GFP and donor backbone) into the second intron of the endogenous locus. It remains unclear how this integration affects the expression of endogenous gene and/or transgenic insert. Does the transgene get spliced out from the intron? Does the intron fail to splice out from the endogenous exons 1-2, making a chimeric protein? Experiments are lacking to validate that the transgene is working as the authors intended. Some key experiments missing are to sequence the cDNA resulting from the insertion, integrating a wild-type version of this transgene with the same method and showing rescue, etc. It is very conceivable that that the integration just made a loss of function mutant and not a catalytic dead mutant as the authors intended.

R4: To validate the knock-in (KI) line, cDNA was generated from both wild-type and *trmt61a*$^{D181A/D181A}$ embryos and used as a template to amplify the *trmt61a* coding sequence. Sanger sequencing of the PCR products confirmed the presence of the c.542A>C point mutation, along with the in-frame P2A and partial EGFP sequences, thereby verifying the correct genomic integration and transcription of the mutant allele (see below **Response Figure 5A**).

Correspondingly, Western blot analysis demonstrated that the Trmt61a antibody effectively recognized the mutant protein, which was expressed at levels comparable to wild-type. Furthermore, the mutant protein remained susceptible to knockdown by *trmt61a* MO injection (see below **Response Figure 5B** also see **Fig EV.5E**). Functionally, LC-MS analysis revealed a significant reduction in tRNA m1A levels in these KI embryos (see below **Response Figure 5C** also see **Fig. 2A**), confirming that Asp181 is the primary catalytic residue for m1A modification in zebrafish.

We acknowledge that the most stringent control would have been a KI line harboring a wild-type version of the transgene. However, based on the proven efficacy of the knock-in methodology and the multi-layered verification of the *Ki*(*trmt61a*^{D181A}-EGFP) line—at the genetic, protein, and functional levels—we conclude that this line effectively serves as an enzymatically inactive *trmt61a* mutant model for our study.

Also in Response 28, the authors did not address the concerns about subcellular localization adequately. They removed the concerning data and then failed to examine whether the construct localized properly at the appropriate time. Another experiment that would help validate the approach.

R5: We apologize for removing the immunofluorescence data for Trmt61a protein in *trmt61a*$^{D181A/D181A}$ embryos at the 256-cell stage. To address this, we performed the immunofluorescence assay at the 1k-cell stage using *trmt61a*$^{D181A/D181A}$ embryos. This analysis confirmed the specificity of the knock-in by revealing clear co-localization of EGFP with Trmt61a-positive cells (see below **Response Figure 5D** also see **Fig. EV.5D**).

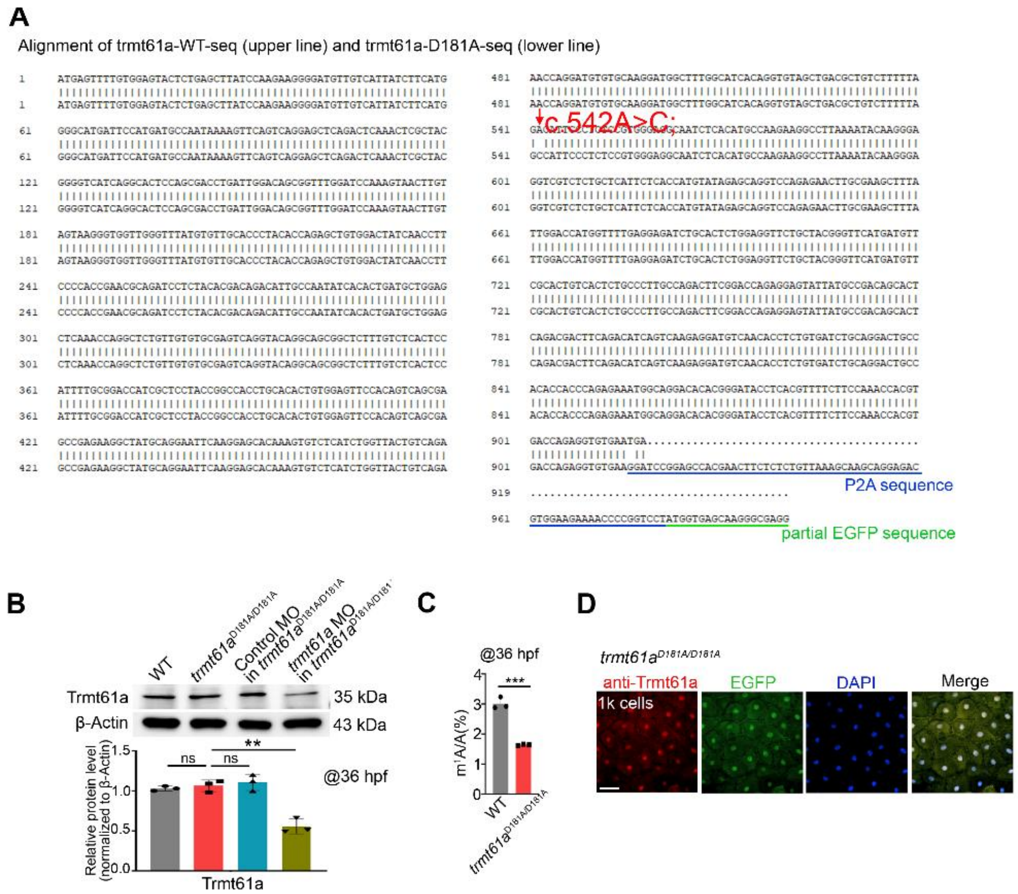

Response Figure 5. Verification of the *Ki*(*trmt61a*^{D181A}-EGFP) line (A) Alignment of wild-type *trmt61a*

sequence and *trmt61a* with point mutation in *trmt61a*$^{D181A/D181A}$ embryos. (B) Western blot analysis (upper) and quantification (lower) showed the Trmt61a protein level in wild-type embryos, *trmt61a*$^{D181A/D181A}$ embryos, and control MO-, or *trmt61a* MO-injected *trmt61a*$^{D181A/D181A}$ embryos. (C) Quantification of m^1A/A ratio in total tRNA isolated from WT and *trmt61a*$^{D181A/D181A}$ embryos at 36 hpf by UHPLC-MS/MS. (D) Immunostaining of Trmt61a and EGFP in *trmt61a*$^{D181A/D181A}$ knockin embryos. Error bars represent mean ± SD. Two-tailed unpaired Student's t-test; $^{**}p<0.01$; $^{***}p<0.001$; ns, no significance. n≥3 biological replicates. Scale bars: 20 μm (D).

3. The authors use the kdrl and runx transgenes to sort out populations of endothelial cells, (kdrl+runx1-), hemogenic endothelial cells (kdrl+runx+) and HSPCs (kdrl-runx+). This is incorrect. The runx1 transgene also labels many other cell types and at 36 hours kdrl+runx1+ cells include both hemogenic ECs and HSPCs. Dissection of just the AGM region will still include cell types not involved in EHT. The terminology should be corrected and kdrl-runx+ category removed.

R6: We have revised the manuscript in accordance with the reviewers' comments.

4. Response 14: The authors did not address the following concern: "previous evidence indicates that m1A methylated mRNAs are a small fraction of the total mRNA pool. Thus, any changes in m1A modification on relevant mRNAs would likely be masked in the complex >200nt pool the study examined." The small fraction of the total mRNA pool in reference still consists of hundreds of mRNAs and thus could have a significant impact on the phenotype.

R7: Previous studies have established that the Trmt61a/Trmt6 complex catalyzes m^1A modification on tRNAs [2-4]. However, to date, there is no direct evidence demonstrating its capacity to methylate m^1A on mRNAs. This includes the findings of the two articles cited by the reviewer 2 in the initial comments (PMIDs: 26863196, 26863410). Therefore, based on the current literature, we hypothesize that in the context of *trmt61a* loss-of-function, mRNA m^1A modifications are likely to remain unaffected. We acknowledge, however, that this possibility cannot be entirely excluded.

5. Review response 19 did not address concern 5 that was raised that wt and mutant transgenes are not expressed equivalently and thus conclusions drawn are merely do to the inequal expression levels.

R8: In this experimental series, *fli1a-nrf1*^{WT} and *fli1a-nrf1*^{MUT} expression constructs were microinjected into *trmt61a* morphants. Equimolar amounts of each construct were administered to ensure comparable expression levels. Subsequent analysis confirmed that the mRNA expression levels of *nrf1* and the co-expressed *tdTomato* reporter were consistent between the two groups (Figure 5G).

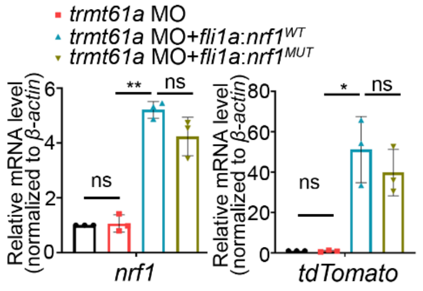

Response Figure 6. The mRNA level of *nrf1* and *tdTomato* at 36 hpf in control MO-, *trmt61a* MO-injected, *trmt61a* MO and *fli1a*:*nrf1*^{WT}-tdTomato or *fli1a*:*nrf1*^{MUT}-tdTomato co-injected embryos.

6. The authors claim that trmt61a impacts hemogenic endothelial specification, however this conclusion cannot be drawn from the data provided. The authors appropriately examined gata2b and gfiaa expression at 24 hours, a time when only hemogenic endothelial cells are present, and HSPCs haven't formed yet during EHT. However, these markers remain on once they become HSPCs around ~28-30 hours, so at later stages hemogenic endothelial cells and HSPCs cannot be differentiated by these markers. Given that the authors found no change in gata2b and gfiaa upon trmt61A loss of function at 24 hours, and changes in the expression of these genes became apparent only at 32 hours, indicates a role for trmt61a later in the process. While technically trmt61A could impact specification, the authors did not provide data that differentiated between HSPC specification and later processes during EHT (e.g. budding, survival, proliferation, etc.). Language implying that it impacts specification or fate should be removed and replaced with more accurate terms such as "HSPC formation" or "HSPC production".

R9: We have revised the manuscript based on the reviewers' feedback. Specifically, the term 'HSPC specification' has been changed to 'HSPC formation' or 'HSPC production' for greater clarity and consistency.

Reference:

[1] LI J, ZHANG B B, REN Y G, et al. Intron targeting-mediated and endogenous gene integrity-maintaining knockin in zebrafish using the CRISPR/Cas9 system [J]. Cell Res, 2015, 25(5): 634-7.

[2] OZANICK S, KRECIC A, ANDERSLAND J, et al. The bipartite structure of the tRNA m1A58 methyltransferase from S. cerevisiae is conserved in humans [J]. RNA, 2005, 11(8): 1281-90.

[3] OERUM S, DEGUT C, BARRAUD P, et al. m1A Post-Transcriptional Modification in tRNAs [J]. Biomolecules, 2017, 7(1):

[4] FINER-MOORE J, CZUDNOCHOWSKI N, O'CONNELL J D, 3RD, et al. Crystal Structure of the Human tRNA m(1)A58 Methyltransferase-tRNA(3)(Lys) Complex: Refolding of Substrate tRNA Allows Access to the Methylation Target [J]. J Mol Biol, 2015, 427(24): 3862-76.

Dear Lu,

Thank you once more for submitting your manuscript to EMBO reports. To recapitulate, your manuscript was reviewed twice at another journal and given the remaining concerns from former referee #2 the editors decided not to offer publication.

You have submitted the anonymous referee reports, a response to the remaining concerns from former referee #2 and your manuscript that had been revised accordingly. We had offered to take the earlier reports and your response/revision into account in arriving at an editorial decision and have asked an expert in the field to arbitrate based on this information. We have now received the reports from two experts.

Referee #1 is an expert in DNA and RNA methylation and has reviewed your manuscript from this angle. The referee points out some important limitations regarding the mechanistic insight provided. Please address these concerns by discussing and acknowledging these limitations and by providing potential explanations in the discussion section. The proposed pathway analysis seems straightforward and could be performed. I am happy to discuss this suggestion if you disagree. The referee furthers suggests a rewriting to make your text more accessible for non-specialists.

Referee #2 is an expert in haematopoiesis with expertise in the zebrafish model and kindly agreed to arbitrate and review your response to the previous referees. As you will see from the report below, the arbitrator considers your response adequate and supports publication after a few minor issues have been addressed.

Please also provide a point-by-point response to the referee/arbitrator comments.

Before we can proceed with official acceptance of your manuscript, I need you to format your study along our editorial and formatting guidelines. You find the general formatting guidelines below, but in order to speed up the process, I list here a few specific points that will need to be addressed:

1) Please
- write the abstract in present tense
- remove the significance statement
- remove the Author contributions from the manuscript text and make sure they are up-to-date and complete in the online manuscript tracking system
- rename Declaration of interests to Disclosure and competing interests statement
- make sure that the manuscript setions are as follows: Title page - Abstract - Introduction - Results - Discussion - Methods - Acknowledgements - Disclosure and competing interests statement - References - Figure legends - Tables and their legends (not EV tables) - Expanded View Figure legends
- format the references alphabetically instead of numerically.
- use et al if more than 10 authors
- define exact p-values in the legend or figure unless p<0.0001
- define all replicates as biological or technical and use statistics only on the former
- provide a Reagents and Tools table as a separate file
- provide an Author Checklist
- prepare for supplying Source data (point 8 below)
- enter the funding information in the online manuscript tracking system. All funders and the numbers must match between the system and the text.

2) Dataset EV1 is not a complex dataset. Some of the information could go to the Reagents and Tools table other could stay as Table EV1.

3) Expanded View information: we need individual production quality figure files for the EV figures. Their legends are part of the manuscript text (see above on sections).

==============================

GENERAL FORMATTING GUIDELINES:

When submitting your revised manuscript, please review the submission guidelines (https://link.springer.com/journal/44319/submission-guidelines#cms-Revised-submissions) and carefully review the instructions that follow below. Failure to include requested items will delay the evaluation of your revision.

1) a .docx formatted version of the manuscript text (including legends for main figures, EV figures and tables). Please make sure that the changes are highlighted to be clearly visible.

2) individual production quality figure files as .eps, .tif, .jpg (one file per figure). See hhttps://media.springernature.com/original/springer-cms/rest/v1/content/27825798/data/v1 for more info on how to prepare your figures.

3) We replaced Supplementary Information with Expanded View (EV) Figures and Tables that are collapsible/expandable online. A maximum of 5 EV Figures can be typeset. EV Figures should be cited as 'Figure EV1, Figure EV2" etc... in the text and their respective legends should be included in the main text after the legends of regular figures.

- For the figures that you do NOT wish to display as Expanded View figures, they should be bundled together with their legends in a single PDF file called *Appendix*, which should start with a short Table of Content. Appendix figures should be referred to in the main text as: "Appendix Figure S1, Appendix Figure S2" etc. See 'Expanded View' section of the submission guidelines.

- Additional Tables/Datasets should be labeled and referred to as Table EV1, Dataset EV1, etc. Legends have to be provided in a separate tab in case of .xls files. Alternatively, the legend can be supplied as a separate text file (README) and zipped together with the Table/Dataset file.

4) a .docx formatted letter INCLUDING the reviewers' reports and your detailed point-by-point responses to their comments. As part of the EMBO Press transparent editorial process, the point-by-point response is part of the Review Process File (RPF), which will be published alongside your paper.

5) a complete author checklist, which you can download from our author guidelines. Please insert information in the checklist that is also reflected in the manuscript. The completed author checklist will also be part of the RPF.

6) Please note that all corresponding authors are required to supply an ORCID ID for their name upon submission of a revised manuscript (<https://orcid.org/>), which can be linked to your profile in the manuscript submission system.

7) Before submitting your revision, primary datasets produced in this study need to be deposited in an appropriate public database. Please remember to provide a reviewer password if the datasets are not yet public. The accession numbers and database should be listed in a formal "Data Availability" section placed after Methods. Please note that the Data Availability Section is restricted to new primary data that are part of this study. * Note - All links should resolve to a page where the data can be accessed. *

If your study has not produced novel datasets, please mention this fact in the Data Availability Section.

8) At EMBO Press we ask authors to provide source data for the main manuscript figures. You will receive a separate email with instructions for providing source data with your revised manuscript, including how to upload and organize the files.

9) Our journal also encourages inclusion of *data citations in the reference list* to directly cite datasets that were re-used and obtained from public databases. Data citations in the article text are distinct from normal bibliographical citations and should directly link to the database records from which the data can be accessed. In the main text, data citations are formatted as follows: "Data ref: Smith et al, 2001" or "Data ref: NCBI Sequence Read Archive PRJNA342805, 2017". In the Reference list, data citations must be labeled with "[DATASET]". A data reference must provide the database name, accession number/identifiers and a resolvable link to the landing page from which the data can be accessed at the end of the reference.

10) Regarding data quantification, the following points must be specified in each figure legend:

- the name of the statistical test used to generate error bars and P values,

- the number (n) of independent experiments (please specify technical or biological replicates) underlying each data point,

- the nature of the bars and error bars (s.d., s.e.m.),

- If the data are obtained from n Program fragment delivered error ``Can't locate object method "less" via package "than" (perhaps you forgot to load "than"?) at //ejpvfs23/sites23b/embor_www/letters/embor_decision_revise_and_review.txt line 54.' 2, use scatter blots showing the individual data points.

Discussion of statistical methodology can be reported in the materials and methods section, but figure legends should contain a basic description of n, P and the test applied.

- Please also include scale bars in all microscopy images.

11) The journal requires a statement specifying whether or not authors have competing interests (defined as all potential or actual interests that could be perceived to influence the presentation or interpretation of an article). In case of competing

interests, this must be specified in your disclosure statement.

12) All Materials and Methods need to be described in the main text using our 'Structured Methods' format, which is required for all research articles. According to this format, the Methods section includes a Reagents and Tools Table (listing key reagents, experimental models, software and relevant equipment and including their sources and relevant identifiers) followed by a Methods and Protocols section describing the methods using a step-by-step protocol format. The aim is to facilitate adoption of the methodologies across labs. More information on how to adhere to this format as well as a downloadable template (.docx) for the Reagents and Tools Table can be found in our author guidelines.

We would also welcome the submission of cover suggestions, or motifs to be used by our Graphics Illustrator in designing a cover.

As part of the EMBO publication's Transparent Editorial Process, EMBO reports publishes online a Review Process File (RPF) to accompany accepted manuscripts. This File will be published in conjunction with your paper and will include the referee reports, your point-by-point response and all pertinent correspondence relating to the manuscript.

You are able to opt out of this by letting the editorial office know (emboreports@embo.org). If you do opt out, the Review Process File link will point to the following statement: "No Review Process File is available with this article, as the authors have chosen not to make the review process public in this case."

I look forward to seeing a revised form of your manuscript when it is ready.

Kind regards,

Martina

Martina Rembold, PhD
Senior Editor
EMBO reports

==============================

Referee #1:

In this study, the authors use zebrafish embryos as a model to reveal a role of the trmt61a tRNA methyltransferase is hematopoietic stem cell formation. Mechanistically, this role is linked to trmt61a-dependent m1A58 tRNA modification, which regulates the translation efficiency of Nrf1, a key regulator of mitochondrial biogenesis. The paper is based on an impressive array of carefully designed experiments and provides novel and convincing results. However, I also found the manuscript challenging to read, as it is very densely written and would greatly benefit from thorough editing to make it more accessible for non-specialists.

Additionally, the paper should be further developed to broaden and generalize the mechanistic insight. For example, in Fig. 3 it is not clear how differentially expressed genes in trmt61a morphants are regulated by m1A58. It is also not clear (mechanistically) why trmt61a morphants show reduced protein synthesis or how loss of m1A58 results in mitochondrial respiratory chain complex assembly (Fig. 4). Importantly, the authors provide some insight into these questions in Fig. 5B and C. However, this needs to be better developed and the analyses need to be driven further to more broadly connect the mechanism to the observed phenotypes. Possible starting points for this could be provided by pathway analyses of mRNAs with low TE and pathway analyses of mRNAs enriched for codons that are decoded by m1A58-hypomodified tRNAs.

Additional points:

Title: clarify that the work was done in zebrafish embryos.

L. 69: terminology - m1A58 is not an „epigenetic" mark.

Fig. 2A: The authors observe about 50% reduction of m1A58. How do they explain the remaining modification levels? This should be clarified in the text.

Referee #2:

Having read the responses to the reviewers, I am satisfied that the authors have addressed all of the major issues highlighted by reviewer #2, in particular the link to p53 and the use of genetic mutants to confirm/verify the MO data, as well as the details and verification of the knock-in experiments. The authors' responses are, in my view, thorough and robust. I believe no more experiments are required and the manuscript is robust and suitable for publication.

I have a few final (minor comments) detailed below.

Line 126 - in line with the response to the reviewer, change this to HSPC production instead (HECs are produced!).

Lines 210-211 - there's a reference to heat shock following fli1a-trmt61a construct overexpression - not sure where this is coming from as they do not use heat shock anywhere in the current manuscript - please check and correct the phrasing.

Fig 5D - I'd use the same colour scheme as in Fig 5G, for example, or increase the size of the symbols (they are really small in the figure, difficult to discern)

Fig 6 - The model seems to suggest ALL HSPCs undergo apoptosis - this is not the case as demostrated by their data on later embryos - perhaps change it slightly to indicate a reduction rather than a loss?

Referee #1:
In this study, the authors use zebrafish embryos as a model to reveal a role of the trmt61a tRNA methyltransferase is hematopoietic stem cell formation. Mechanistically, this role is linked to trmt61a-dependent m1A58 tRNA modification, which regulates the translation efficiency of Nrf1, a key regulator of mitochondrial biogenesis. The paper is based on an impressive array of carefully designed experiments and provides novel and convincing results. However, I also found the manuscript challenging to read, as it is very densely written and would greatly benefit from thorough editing to make it more accessible for non-specialists.
We thank the reviewer for the positive evaluation of our work and for this helpful suggestion. Accordingly, we have carefully revised the manuscript to reduce density, improve clarity, and enhance accessibility for non-specialist readers.

Additionally, the paper should be further developed to broaden and generalize the mechanistic insight. For example, in Fig. 3 it is not clear how differentially expressed genes in trmt61a morphants are regulated by m1A58. It is also not clear (mechanistically) why trmt61a morphants show reduced protein synthesis or how loss of m1A58 results in mitochondrial respiratory chain complex assembly (Fig. 4). Importantly, the authors provide some insight into these questions in Fig. 5B and C. However, this needs to be better developed and the analyses need to be driven further to more broadly connect the mechanism to the observed phenotypes. Possible starting points for this could be provided by pathway analyses of mRNAs with low TE and pathway analyses of mRNAs enriched for codons that are decoded by m1A58-hypomodified tRNAs.
R1: We thank the reviewer for this insightful comment.

First, regarding the differentially expressed genes observed in *trmt61a* morphants, we note that m^1A58 is a tRNA modification that primarily affects translation rather than transcription. Therefore, we do not expect m^1A58 to directly regulate transcriptional programs. Instead, we propose that the transcriptional alterations detected in *trmt61a*-deficient embryos arise secondarily from changes in the proteome, resulting from impaired translation of specific regulatory proteins. Consistent with this model, our ribosome profiling analysis demonstrates a global reduction in translation efficiency (TE) that preferentially affects transcripts enriched for codons decoded by m^1A58-hypomodified tRNAs (see below **Response Figure 1A, B** and **Figure 5B, C**).

To further address the reviewer's suggestion, we expanded our analysis by performing pathway enrichment analysis of transcripts with reduced TE. These analyses revealed significant enrichment of genes involved in mitochondrial respiration and protein stabilization. These findings suggest that impaired translation of codon-biased transcripts may broadly affect mitochondrial homeostasis and proteostasis (see below **Response Figure 1C** and **Appendix Fig.8E, Lines 250-253**).

Proteomic analysis revealed a subset of proteins significantly reduced in *trmt61a*-deficient embryos. Among these candidates, we identify Nrf1, a master regulator of mitochondrial biogenesis, as a representative downstream effector. We demonstrate that although *nrf1* mRNA levels are not reduced, its polysome association is significantly decreased in *trmt61a*-deficient embryos, indicating impaired translational efficiency. Furthermore, a codon-optimized *nrf1* construct that bypasses the requirement for m^1A58-modified tRNAs restores Nrf1 protein expression and significantly rescues the HSPC defects in *trmt61a* morphants. These results

provide direct evidence that m^{1}A58-dependent translational control contributes to the mitochondrial defects and hematopoietic phenotypes observed upon *trmt61a* loss.

Together, these additional analyses strengthen the mechanistic model that Trmt61a-mediated m^{1}A58 modification supports efficient translation of codon-biased transcripts, including regulators of mitochondrial function. Disruption of this translational program leads to mitochondrial dysfunction, activation of apoptotic pathways, and impaired HSPC emergence during embryogenesis.

These clarifications have now been incorporated into the revised manuscript (**Discussion**, **Lines 297-313**).

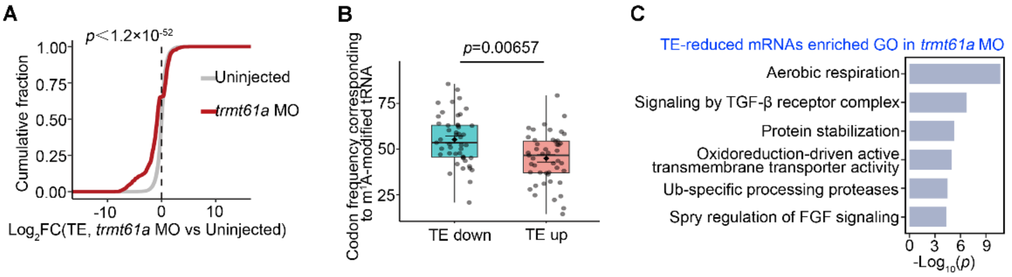

Response Figure 1. Trmt61a deficiency impairs translation efficiency. (A) Cumulative distribution of TE between *trmt61a* morphants and uninjected embryos at 36 hpf (two biological replicates). Statistics by Wilcoxon test. **(B)** Frequency of codons corresponding to m^{1}A58-modified tRNAs was higher in the genes with decreased translation efficiency (two biological replicates). Statistics by Two-tailed paired Student's *t*-test. (C) Representative gene ontology biological process categories enriched in TE-reduced genes in *trmt61a* morphants.

Additional points:

Title: clarify that the work was done in zebrafish embryos.

R2: Thank you for this suggestion. We have revised the title, as shown in "tRNA m1A modification ensures HSPC production via modulating Nrf1 translation in zebrafish".

L. 69: terminology - m1A58 is not an „epigenetic" mark.

R3: Thank you for this suggestion. We have deleted this sentence and revised the manuscript accordingly as "One of the most prevalent modifications is N1-methyladenosine (m^{1}A). Among them, m1A58 is a conserved modification located at position 58 in the T-loop of tRNAs and plays an important role in maintaining tRNA structural stability and proper function" (**Lines 56-59**).

Fig. 2A: The authors observe about 50% reduction of m1A58. How do they explain the remaining modification levels? This should be clarified in the text.

R4: We thank the reviewer for raising this important point.

Previous studies have identified Asp181 as a conserved catalytic residue that plays a major role in Trmt61a-mediated tRNA m^{1}A58 methylation (Wang et al., 2021 , Liu et al., 2022 , Miao et al., 2025). Meanwhile, other studies have further suggested that the catalytic activity of Trmt61a involves multiple residues that collectively contribute to methyltransferase activity (Finer-Moore et al., 2015 , Barraud et al., 2008 , Tao et al., 2025). Based on these observations, we speculate that

additional catalytic residues may also contribute to Trmt61a enzymatic activity in zebrafish, such that mutation of Asp181 reduces but does not completely abolish m^1A58 formation.

Importantly, despite the remaining signal, the substantial reduction of m^1A observed in the *trmt61a*^{D181A} mutants confirms that Asp181 is critical for Trmt61a catalytic activity in vivo. We have now clarified this point in the revised manuscript (**Lines 158-161**).

Referee #2:

Having read the responses to the reviewers, I am satisfied that the authors have addressed all of the major issues highlighted by reviewer #2, in particular the link to p53 and the use of genetic mutants to confirm/verify the MO data, as well as the details and verification of the knock-in experiments. The authors' responses are, in my view, thorough and robust. I believe no more experiments are required and the manuscript is robust and suitable for publication.

We thank the reviewer for the positive assessment of our study.

I have a few final (minor comments) detailed below.

Line 126 - in line with the response to the reviewer, change this to HSPC production instead (HECs are produced!).

R5: Thank you for this suggestion. We have revised the manuscript (**Line 109**)

Lines 210-211 - there's a reference to heat shock following fli1a-trmt61a construct overexpression - not sure where this is coming from as they do not use heat shock anywhere in the current manuscript - please check and correct the phrasing.

R6: We apologize for this error in the manuscript. We have checked and revised the manuscript accordingly (**Line 180**).

Fig 5D - I'd use the same colour scheme as in Fig 5G, for example, or increase the size of the symbols (they are really small in the figure, difficult to discern)

R7: We apologize for not presenting this result clearly. In the revised manuscript, we have magnified the size of this panel in the **Figure 5**.

Fig 6 - The model seems to suggest ALL HSPCs undergo apoptosis - this is not the case as demostrated by their data on later embryos - perhaps change it slightly to indicate a reduction rather than a loss?

R8: Thanks for this suggestion. We have revised the model.

References

BARRAUD P, GOLINELLI-PIMPANEAU B, ATMANENE C, et al. 2008. Crystal structure of Thermus thermophilus tRNA m1A58 methyltransferase and biophysical characterization of its interaction with tRNA. J Mol Biol [J], 377: 535-550.

FINER-MOORE J, CZUDNOCHOWSKI N, O'CONNELL J D, 3RD, et al. 2015. Crystal Structure of the Human tRNA m(1)A58 Methyltransferase-tRNA(3)(Lys) Complex: Refolding of Substrate tRNA Allows Access to the Methylation Target. J Mol Biol [J], 427: 3862-3876.

LIU Y, ZHOU J, LI X, et al. 2022. tRNA-m(1)A modification promotes T cell expansion via efficient MYC protein synthesis. Nat Immunol [J], 23: 1433-1444.

MIAO S, LI H, SONG X, et al. 2025. tRNA m1A modification regulates cholesterol biosynthesis to promote

antitumor immunity of CD8+ T cells. J Exp Med [J], 222.

TAO E W, WANG Y, TAN J, et al. 2025. TRMT6-mediated tRNA m(1)A modification acts as a translational checkpoint of histone synthesis and facilitates colorectal cancer progression. Nat Cancer [J], 6: 1458-1476.

WANG Y, WANG J, LI X, et al. 2021. N(1)-methyladenosine methylation in tRNA drives liver tumourigenesis by regulating cholesterol metabolism. Nat Commun [J], 12: 6314.

Manuscript number: EMBOR-2026-63753V2
Title: tRNA m1A modification ensures HSPC production via modulating Nrf1 translation in zebrafish
Author(s): Lu Wang, Zhenkun Dong, Panfeng Li, Mengyao Liu, Sifeng Wang, Weiyi Lai, Yining Liu, Hailin Wang, and Ang Li

Dear Lu,

Thank you for submitting your revised manuscript, which is in almost perfect format. There are only a few minor things that are still needed before I can officially accept the manuscript. I am therefore writing with an 'accept in principle' decision, which means that I will be happy to accept your manuscript for publication once the few minor concerns listed below have been addressed:

- The labels for Dataset EV1-EV2 to be corrected in the sheet with the dataset legend (currently this tab in the .xls file is named as Table S2-S3)

- The legend for Table EV1 needs to be removed from the manuscript. It is sufficient to have it in the .xls file.

- Appendix PDF: Please change the red text to black on the last page.

- Please change the label on the Appendix figures to "Appendix Fig. S#".

- Appendix figure legends:
Legend for Appendix Fig. S1B: "(three biological replicates)" is duplicated. I suggest adding that one representative image/sample is shown. The replicate description is also in duplicate for panel E.
S1C: please add the reference for the published scRNA-seq dataset, at least the dataset identifier.
S2B: please define the bars and error bars (mean/median, SEM/SD).
S2C: I suggest adding that one representative staining of three biological replicates is shown. I recommend doing this in all legends when you show one example image/staining for an experiment you repeated three times.

- Please remove the Reagents and Tools table from the manuscript and upload it as separate file.

- The source data for Figure 1G has been sorted to folder "F" instead of its own folder "G".

- This reference could have a data reference in addition to citing the paper, since you seem to have reused the scRNA seq data published by these authors.
Xia, J., Liu, M., Zhu, C., Liu, S., Ai, L., Ma, D., Zhu, P., Wang, L., and Liu, F. (2023). Activation of lineage competence in hemogenic endothelium precedes the formation of hematopoietic stem cell heterogeneity. Cell Res 33, 448-463.
The dataref would list the same authors but instead of the journal it should provide the URL that resolves to the dataset. In the reference list you need to add [DATASET]. In the text, if you chose to add a datareference, it would read (Xia et al, 2023; dataref: Xia et al, 2023)

- Please provide the specific URL for CRA028758 in the data availability statement.

- Please address the following points in the figure legends:
1)Please note that the statistical tests information for figures 2 and 3 is mislabeled in the legends. This needs to be rectified.
2) Please indicate the statistical test used for data analysis in the legends of figures 3A-C."
3) Please note that information related to n is missing in the legend of figure 5C

- Finally, EMBO Reports papers are accompanied online by
A) a short (1-2 sentences) summary of the findings and their significance,
B) 2-3 bullet points highlighting key results and
C) a schematic summary figure that provides a sketch of the major findings (not a data image).
Please provide the summary figure as a separate file in PNG or JPG format at a size of 550x300-600 pixels (width x height). Please note that the size is rather small and that text needs to be readable at the final size. Please send us this information along with the revised manuscript.

Once you have made these minor revisions, please use the following link to submit your corrected manuscript:

Link Not Available

If all remaining corrections have been attended to, you will then receive an official decision letter from the journal accepting your manuscript for publication in the next available issue of EMBO reports. This letter will also include details of the further steps

you need to take for the prompt inclusion of your manuscript in our next available issue.

Thank you for your contribution to EMBO reports.

Kind regards,

Martina

Martina Rembold, PhD
Senior Editor
EMBO reports

All minor editorial requests have been addressed by the authors.

2nd Revision - Editorial Decision 24th Apr 2026

Dear Lu,

Thank you for the submission of your revised manuscript to our offices and please apologize my delayed response. All is fine now but there are two things that still need your attention before I can officially accept it.

- We perform a routine integrity check on all figures of revised manuscripts. In this case, it appears as if two WISH images are very similar to each other or the same.
This is in Figure S2E, the staining for fli1a for the trmt61a MO and
Figure S5E, the staining for fli1a for the trmt61a[D181A/D181A] embryo.

Please double-check these images and ensure that there was no mix-up. If you are sure that these two images are from the respective stainings and mutant phenotypes, I would even suggest to choose a different example to avoid potential misunderstandings.

- In addition, I added one request for changes in the reference list, regarding the data reference and one comment in the figure legend regarding statistics. Could you please review these?

Once these two issues are sorted out, we will proceed with official acceptance of your study.

Thank you very much.

Kind regards,

Martina

Martina Rembold, PhD
Senior Editor
EMBO reports

All minor editorial requests have been addressed by the authors.

3rd Revision - Editorial Decision 30th Apr 2026

Dr. Lu Wang
State Key Laboratory of Experimental Hematology, National Clinical Research Center for Blood Diseases, Haihe Laboratory of Cell Ecosystem, Institute of Hematology & Blood Diseases Hospital, Chinese Academy of Medical Sciences & Peking Union Medical College
Stem Cells and Regenerative Medicine
Nanjing Road 288, Heping District
TIANJIN, TIANJIN 300020
China

Dear Lu,

Thank you for the final minor changes. I fixed a small error with the data reference in the manuscript (attached for your information) and uploaded the file. Therefore, all looks good and I am very pleased to accept your manuscript for publication in the next available issue of EMBO reports. Thank you for your contribution to our journal.

Your manuscript will be processed for publication by EMBO Press. It will be copy edited and you will receive page proofs prior to publication. Please note that you will be contacted by Springer Nature Author Services to complete licensing and payment information.

You may qualify for financial assistance for your publication charges - either via a Springer Nature fully open access agreement or an EMBO initiative. Check your eligibility: https://link.springer.com/journal/44319/how-to-publish-with-us

Should you be planning a Press Release on your article, please get in contact with embo_production@springernature.com as early as possible in order to coordinate publication and release dates.

If you have any questions, please do not hesitate to contact the Editorial Office. Thank you for your contribution to EMBO Reports.

Kind regards,

Martina

Martina Rembold, PhD
Senior Editor
EMBO reports

>>> Please note that it is EMBO Reports policy for the transcript of the editorial process (containing referee reports and your response letter) to be published as an online supplement to each paper. If you do NOT want this, you will need to inform the Editorial Office via email immediately. More information is available here: https://link.springer.com/partners/embo-press/editorial-policies#Peer%20review

EMBO Press Author Checklist

Corresponding Author Name: Lu Wang
Journal Submitted to: EMBO reports
Manuscript Number: EMBOR-2026-63753-T

USEFUL LINKS FOR COMPLETING THIS FORM
The EMBO Journal - Author Guidelines
EMBO Reports - Author Guidelines
Molecular Systems Biology - Author Guidelines
EMBO Molecular Medicine - Author Guidelines

Reporting Checklist for Life Science Articles (updated January
This checklist is adapted from Materials Design Analysis Reporting (MDAR) Checklist for Authors. MDAR establishes a minimum set of requirements in transparent reporting in the life sciences (see Statement of Task: 10.31222/osf.io/9sm4x). Please follow the journal's guidelines in preparing your
Please note that a copy of this checklist will be published alongside your article.

Abridged guidelines for figures
1. Data
The data shown in figures should satisfy the following conditions:
- the data were obtained and processed according to the field's best practice and are presented to reflect the results of the experiments in an accurate and unbiased manner.
- ideally, figure panels should include only measurements that are directly comparable to each other and obtained with the same assay.
- plots include clearly labeled error bars for independent experiments and sample sizes. Unless justified, error bars should not be shown for technical
- if n<5, the individual data points from each experiment should be plotted. Any statistical test employed should be justified.
- Source Data should be included to report the data underlying figures according to the guidelines set out in the authorship guidelines on Data

2. Captions
Each figure caption should contain the following information, for each panel where they are relevant:
- a specification of the experimental system investigated (eg cell line, species name).
- the assay(s) and method(s) used to carry out the reported observations and measurements.
- an explicit mention of the biological and chemical entity(ies) that are being measured.
- an explicit mention of the biological and chemical entity(ies) that are altered/varied/perturbed in a controlled manner.
- the exact sample size (n) for each experimental group/condition, given as a number, not a range;
- a description of the sample collection allowing the reader to understand whether the samples represent technical or biological replicates (including how many animals, litters, cultures, etc.).
- a statement of how many times the experiment shown was independently replicated in the laboratory.
- definitions of statistical methods and measures:
  - common tests, such as t-test (please specify whether paired vs. unpaired), simple χ2 tests, Wilcoxon and Mann-Whitney tests, can be unambiguously identified by name only, but more complex techniques should be described in the methods section;
  - are tests one-sided or two-sided?
  - are there adjustments for multiple comparisons?
  - exact statistical test results, e.g., P values = x but not P values < x;
  - definition of 'center values' as median or average;
  - definition of error bars as s.d. or s.e.m.

Please complete ALL of the questions below.
Select "Not Applicable" only when the requested information is not relevant for your study.

Materials

Newly Created Materials	Information included in the manuscript?	In which section is the information available? (Reagents and Tools Table, Materials and Methods, Figures, Data Availability Section)
New materials and reagents need to be available; do any restrictions apply?	Not Applicable	

Antibodies	Information included in the manuscript?	In which section is the information available? (Reagents and Tools Table, Materials and Methods, Figures, Data Availability Section)
For **antibodies** provide the following information:<br>- Commercial antibodies: RRID (if possible) or supplier name, catalogue number and or/clone number<br>- Non-commercial: RRID or citation	Yes	All related information are included in Methods.

DNA and RNA sequences	Information included in the manuscript?	In which section is the information available? (Reagents and Tools Table, Materials and Methods, Figures, Data Availability Section)
**Short novel DNA or RNA including primers, probes**: provide the sequences.	Yes	All related information are included in Methods and Table EV1.

Cell materials	Information included in the manuscript?	In which section is the information available? (Reagents and Tools Table, Materials and Methods, Figures, Data Availability Section)
**Cell lines:** Provide species information, strain. Provide accession number in repository **OR** supplier name, catalog number, clone number, and/**OR** RRID.	Not Applicable	
**Primary cultures:** Provide species, strain, sex of origin, genetic modification status.	Not Applicable	
Report if the cell lines were recently **authenticated** (e.g., by STR profiling) and tested for mycoplasma contamination.	Not Applicable	

Experimental animals	Information included in the manuscript?	In which section is the information available? (Reagents and Tools Table, Materials and Methods, Figures, Data Availability Section)
**Laboratory animals or Model organisms:** Provide species, strain, sex, age, genetic modification status. Provide accession number in repository **OR** supplier name, catalog number, clone number, **OR** RRID.	Yes	All related information are included in Methods.
**Animal observed in or captured from the field:** Provide species, sex, and age where possible.	Not Applicable	
Please detail **housing and husbandry conditions**.	Yes	All related information are included in Methods.

Plants and microbes	Information included in the manuscript?	In which section is the information available? (Reagents and Tools Table, Materials and Methods, Figures, Data Availability Section)
**Plants:** provide species and strain, ecotype and cultivar where relevant, unique accession number if available, and source (including location for collected wild specimens).	Not Applicable	
**Microbes:** provide species and strain, unique accession number if available, and source.	Not Applicable	

Human research participants	Information included in the manuscript?	In which section is the information available? (Reagents and Tools Table, Materials and Methods, Figures, Data Availability Section)
If collected and within the bounds of privacy constraints report on age, sex and gender or ethnicity for all study participants.	Not Applicable	

Core facilities	Information included in the manuscript?	In which section is the information available? (Reagents and Tools Table, Materials and Methods, Figures, Data Availability Section)
If your work benefited from core facilities, was their service mentioned in the acknowledgments section?	Not Applicable	

Design

Study protocol	Information included in the manuscript?	In which section is the information available? (Reagents and Tools Table, Materials and Methods, Figures, Data Availability Section)
If study protocol has been **pre-registered, provide DOI in the manuscript**. For clinical trials, provide the trial registration number **OR** cite DOI.	Not Applicable	
Report the **clinical trial registration number** (at ClinicalTrials.gov or equivalent), where applicable.	Not Applicable	

Laboratory protocol	Information included in the manuscript?	In which section is the information available? (Reagents and Tools Table, Materials and Methods, Figures, Data Availability Section)
Provide DOI OR other citation details if **external detailed step-by-step protocols** are available.	Not Applicable	

Experimental study design and statistics	Information included in the manuscript?	In which section is the information available? (Reagents and Tools Table, Materials and Methods, Figures, Data Availability Section)
Include a statement about **sample size** estimate even if no statistical methods were used.	Yes	All related information are included in Methods.
Were any steps taken to minimize the effects of subjective bias when allocating animals/samples to treatment (e.g. **randomization procedure**)? If yes, have they been described?	Not Applicable	
Include a statement about **blinding** even if no blinding was done.	Not Applicable	
Describe **inclusion/exclusion criteria** if samples or animals were excluded from the analysis. Were the criteria pre-established?<br><br>If sample or data points were omitted from analysis, report if this was due to attrition or intentional exclusion and provide justification.	Not Applicable	
For every figure, are **statistical tests** justified as appropriate? Do the data meet the assumptions of the tests (e.g., normal distribution)? Describe any methods used to assess it. Is there an estimate of variation within each group of data? Is the variance similar between the groups that are being statistically compared?	Yes	All related information are included in Figure Legends and Methods.

Sample definition and in-laboratory replication	Information included in the manuscript?	In which section is the information available? (Reagents and Tools Table, Materials and Methods, Figures, Data Availability Section)
In the figure legends: state number of times the experiment was **replicated** in laboratory.	Yes	All related information are included in Figure Legneds.
In the figure legends: define whether data describe **technical or biological replicates**.	Yes	All related information are included in Figure Legneds.

Ethics

Ethics	Information included in the manuscript?	In which section is the information available? (Reagents and Tools Table, Materials and Methods, Figures, Data Availability Section)
Studies involving **human participants**: State details of **authority granting ethics approval** (IRB or equivalent committee(s), provide reference number for approval.	Not Applicable	
Studies involving **human participants**: Include a statement confirming that **informed consent** was obtained from all subjects and that the experiments conformed to the principles set out in the WMA Declaration of Helsinki and the Department of Health and Human Services Belmont Report.	Not Applicable	
Studies involving **human participants:** For publication of **patient photos**, include a statement confirming that consent to publish was obtained.	Not Applicable	
Studies involving experimental **animals**: State details of **authority granting ethics approval** (IRB or equivalent committee(s), provide reference number for approval. Include a statement of compliance with ethical regulations.	Not Applicable	
Studies involving **specimen and field samples:** State if relevant **permits** obtained, provide details of authority approving study; if none were required, explain why.	Not Applicable	

Dual Use Research of Concern (DURC)	Information included in the manuscript?	In which section is the information available? (Reagents and Tools Table, Materials and Methods, Figures, Data Availability Section)
Could your study fall under dual use research restrictions? Please check biosecurity documents and list of **select agents and toxins** (CDC): https://www.selectagents.gov/sat/list.htm	Not Applicable	
If you used a select agent, is the security level of the lab appropriate and reported in the manuscript?	Not Applicable	
If a study is subject to dual use research of concern regulations, is the name of the **authority granting approval and reference number** for the regulatory approval provided in the manuscript?	Not Applicable	

Reporting

The MDAR framework recommends adoption of discipline-specific guidelines, established and endorsed through community initiatives. Journals have their own policy about requiring specific guidelines and recommendations to complement MDAR.

Adherence to community standards	Information included in the manuscript?	In which section is the information available? (Reagents and Tools Table, Materials and Methods, Figures, Data Availability Section)
State if relevant guidelines or checklists (e.g., **ICMJE, MIBBI, ARRIVE, PRISMA**) have been followed or provided.	Not Applicable	
For **tumor marker prognostic studies,** we recommend that you follow the **REMARK** reporting guidelines (see link list at top right). See author guidelines, under 'Reporting Guidelines'. Please confirm you have followed these guidelines.	Not Applicable	
For **phase II and III randomized controlled trials**, please refer to the **CONSORT** flow diagram (see link list at top right) and submit the CONSORT checklist (see link list at top right) with your submission. See author guidelines, under 'Reporting Guidelines'. Please confirm you have submitted this list.	Not Applicable	

Data Availability

Data availability	Information included in the manuscript?	In which section is the information available? (Reagents and Tools Table, Materials and Methods, Figures, Data Availability Section)
Have **primary datasets** been deposited according to the journal's guidelines (see 'Data Deposition' section) and the respective accession numbers provided in the Data Availability Section?	Yes	All related information are included in Data Avaliability Section.
Were **human clinical and genomic datasets** deposited in a public access-controlled repository in accordance to ethical obligations to the patients and to the applicable consent agreement?	Not Applicable	
Are **computational models** that are central and integral to a study available without restrictions in a machine-readable form? Were the relevant accession numbers or links provided?	Not Applicable	
If publicly available data were reused, provide the respective **data citations in the reference list**.	Not Applicable	
